# RNAi in *Piezodorus guildinii* (Hemiptera: Pentatomidae): Transcriptome Assembly for the Development of Pest Control Strategies

**DOI:** 10.3389/fpls.2022.804839

**Published:** 2022-04-01

**Authors:** Claudia Schvartzman, Pablo Fresia, Sara Murchio, María Valentina Mujica, Marco Dalla-Rizza

**Affiliations:** ^1^Unidad de Biotecnología, Instituto Nacional de Investigación Agropecuaria, Canelones, Uruguay; ^2^Unidad Mixta Pasteur + INIA (UMPI), Institut Pasteur de Montevideo, Montevideo, Uruguay; ^3^Unidad de Protección Vegetal, Instituto Nacional de Investigación Agropecuaria, Canelones, Uruguay

**Keywords:** stink bug, RNA-seq, dsRNA, RNAi, vATPase A, pest control

## Abstract

Red-banded stink bug *Piezodorus guildinii* (*P. guildinii*) has been described as the most damaging stink bug regarding soybean crops, leading to seed injury, low germination percentages, and foliar retention, at low population densities. In recent years, RNA interference (RNAi), a conserved eukaryote silencing mechanism has been explored to develop species-selective pesticides. In this work, we evaluated RNAi in *P. guildinii* to develop new pest-control strategies. For this, we assembled and annotated a *P. guildinii* transcriptome from a pool of all developmental stages. Analysis of this transcriptome led to the identification of 56 genes related to the silencing process encompassing siRNA, miRNA, and piRNA pathways. To evaluate the functionality of RNAi machinery, *P. guildinii* adults were injected with 28 ng/mg of body weight of double stranded RNA (dsRNA) targeting *vATPase A*. A mortality of 35 and 51.6% was observed after 7 and 14 days, respectively, and a downregulation of *vATPase A* gene of 84% 72 h post-injection. In addition, *Dicer-2* and *Argonaute-2* genes, core RNAi proteins, were upregulated 1.8-fold 48 h after injection. These findings showed for the first time that RNAi is functional in *P. guildinii* and the silencing of essential genes has a significant effect in adult viability. Taken together, the work reported here shows that RNAi could be an interesting approach for the development of red-banded stink bug control strategies.

## Introduction

Soybean (*Glycine max*) is one of the most extensively grown legume used for protein meal and vegetable oil, with an estimated 6% usage of the worlds’ arable land (Hartman et al., 2011). Soybean cultivation in the United States covers large areas, being the largest producing countries Brazil, the United States, and Argentina, which have an adoption of biotech-enhanced soybean seedstock of more than 90%.^1^ Phytophagous stink bug complex (*Pentatomidae* family) is an important sanitary problem of the crop (Zerbino et al., 2016). In particular, red-banded stink bug or small-green stink bug *Piezodorus guildinii* (Westwood) (*P. guildinii*) has a Neotropical distribution, spanning from Argentina to the southern United States (Panizzi and Slansky, 1985). Economic damage, estimated at approximately 5% of the harvest, occurs when feeding, from pod formation to maturity (Bundy et al., 2019). The magnitude of the damage depends on the population reached, and the time of exposure to the infestation (Corrêa-Ferreira and De Azevedo, 2002). *P. guildinii* has been described as one of the most damaging stink bug regarding soybean, leading to reduced yield and quality, affecting seed weight and oil content, delaying crop maturity, and reducing the germination of the harvested seed (Depieri and Panizzi, 2011; Bundy et al., 2019). Current control strategies relay on the application of insecticides based on pyrethroids and neonicotinoids. Pyrethroids act on sucking and chewing insects with tumbling power, while neonicotinoids are highly residual systemic compounds used in sucking insects (Baur et al., 2010; Sosa-Gómez et al., 2020). It has been shown that these insecticides negatively impact natural enemies, are particularly harmful on bees, and have a detrimental effect on the environment (Giorio et al., 2021). Moreover, the lack of options for the control of these insects leads to the use of active principles in the same season and for several years with a similar mode of action, favoring the emergence of resistance (Temple et al., 2013).

RNA interference (RNAi) is a natural gene regulation mechanism present in eukaryotic cells (Svoboda, 2020). In this process, small RNA molecules (sRNA) associate with Argonaute proteins, forming the RNA-induced silencing complex (RISC), which uses complementary base pairing of the sRNA to identify target RNA molecules to be silenced (Ketting, 2011). RNAi pathways differ in the proteins involved, the origin and type of sRNA, the target RNA and, thus, the biological function. Biological roles of RNA silencing pathways include the regulation of endogenous gene expression (miRNA), antiviral immunity (siRNA), and genome protection against transposable elements (piRNA) (Ketting, 2011; Dowling et al., 2016). In siRNA, the basic mechanism consists of a long double stranded RNA (dsRNA) molecule (exogenous or endogenous) which is processed by ribonuclease III type Dicer-2 (Dcr-2) to a small duplex of 21–23 nucleotides. The resulting siRNAs are then loaded into RISC where the duplex is unbounded by the action of Ago-2, the sense strand is degraded, and the antisense strand guides the RISC to the target mRNA. Finally, Ago-2 provides the endonucleolytic activity, silencing gene expression (Mello and Conte, 2004; de Andrade and Hunter, 2016; Svoboda, 2020).

Given the growing need for alternatives to chemical pesticides, the use of RNAi emerges as a highly specific strategy, low environmental impact, and non-transgenic alternative (Christiaens et al., 2020). Reports of successful progress in pest control strategies in insects has been reviewed extensively (Christiaens et al., 2020; Zhu and Palli, 2020; Nitnavare et al., 2021). Nevertheless, the development of effective RNAi strategies in insects is a complex task and there is a great variability response in different insect orders. While coleopterans has shown to be highly susceptible (Baum et al., 2007; Baum and Roberts, 2014), insects from Lepidoptera (Terenius et al., 2011; Kolliopoulou and Swevers, 2014), Diptera (Maktura et al., 2021), and Hemiptera (Jain et al., 2021) respond with greater variability (Cooper et al., 2019). Several factors play a role in this observed differential RNAi efficiency: the presence of dsRNA degrading enzymes in the hemolymph and lumen (Christiaens and Smagghe, 2014; Song et al., 2017), inefficient intracellular transport of dsRNA including entrapment in the endosomes (Shukla et al., 2016; Yoon et al., 2017), or missing core components of the RNAi machinery (Arraes et al., 2021).

Several studies have shown that RNAi is efficient in stink bugs. In the brown marmorated stink bug *Halyomorpha halys*, administration of dsRNA by injection and feeding showed a reduction in the expression levels and mortality of up to 70% targeting essential genes (Mogilicherla et al., 2018). In the Neotropical brown stink bug *Euschistus heros*, RNAi core proteins were identified in the transcriptome, and adult injection of dsRNA targeting vATPase A caused a reduction in *vATPase A* gene expression and significant mortality (Cagliari et al., 2020). Moreover, the insecticidal effect with the same target in second instar nymphs reached 80% of mortality 14 days after injection. In feeding assays, the formulation with ethylenediaminetetraacetic acid (EDTA) or Liposome encapsulation enhanced the mortality up to 45 and 51%, respectively, compared with naked dsRNA against *vATPae A*, where no significant mortality was reported (Castellanos et al., 2019). In addition, a complex of a shRNA against troponin coupled to nanoparticles showed significant mortality by oral administration (Laisney et al., 2021). Studies in southern green stink bug *Nezara viridula* showed that injected dsRNA targeting essential genes could induce significant mortality (Riga et al., 2020; Gurusamy et al., 2021). Particularly, dsRNA targeting *vATPase A* achieved more than 80% mortality in injection assays, and 45% when feeding. This response was enhanced when specific *N. viridula* dsRNases were silenced by injection before oral administration in second instar nymphs targeting the α*cop* gene (Sharma et al., 2020, 2021). In the harlequin bug, *Murgantia histrionica* (Hahn), RNAi core proteins were identified and significant mortality was shown by injection and feeding targeting several genes (Howell et al., 2020). Finally, the brown-winged green stinkbug *Plautia stali* was very sensitive to vATPase E dsRNA injection, but a lower response was reported by oral feeding (Nishide et al., 2021).

In this work, we explored RNAi in *P. guildinii* towards the development of environmentally low impact pest control strategies. For this, we generated, to our knowledge, the first transcriptome of this stink bug species. Machinery RNAi genes were annotated, finding core RNAi genes, RISC associated genes, uptake, and intracellular transport related genes as well as RNAases. We demonstrated RNAi *in vivo* by dsRNA injection targeting *vATPase A*. Gene expression was significantly reduced in treated animals, and a 51.6% mortality rate was observed after 14 days. The gene expression of core proteins after injection in dsRNA treated animals was evaluated as well, with a significant overexpression of *Dcr-2* and *Ago-2* 48 h after injection. These results showed that *P. guildinii* is susceptible to RNAi, and this approach could be exploited for the development of integrated pest control strategies.

## Materials and Methods

### Insect Rearing

Adults *P. guildinii* were collected in INIA La Estanzuela, Colonia, Uruguay (S34° 20′W 57°41′) and maintained at 26 ± 1°C, 80 ± 10% RH, and a 16:8 light:dark cycle. Insects were fed *ad livitum* with green bean pods (*Phaseolus vulgaris*), dry soybean seeds (*G. max*), and raw shelled peanuts (*Arachis hypogaea*), distilled water was supplied every day by moistened cotton. Eggs were removed to a different plastic container and nymphs were checked daily until adult emergence.

### cDNA Libraries and Sequencing

Total RNA was purified from all nymphal stages (1st–5th) and adults (male and female) using an RNeasy Mini Kit Qiagen (Hilden, Germany), according to manufacturer instructions. RNA integrity was determined with a Bioanalyzer 2100 Agilent Technologies (Santa Clara, United States). Equal RNA quantity of every stage was pooled to a 1 μg sample used for cDNA library preparation and Illumina (San Diego, United States) sequencing was conducted by Macrogen, Inc. (Seoul, South Korea). TruSeq stranded mRNA libraries were generated with a TruSeq Stranded mRNA LT Sample Prep Kit Ilumina (San Diego, United States), and sequencing was performed with Illumina HiSeq 2500 platform with a coverage of 75 G (1 lane), 150 bp paired ends reads.

### Transcriptome Assembly

The quality of raw reads from the Illumina sequencing was analyzed by FastQC software^2^ and filtered as follows: erroneous k-mers were eliminated with r-Corrector software (Song and Florea, 2015), adaptors and bases with a Phred score lower than 30 were trimmed with TrimmGalore.^3^ Additionally, reads were mapped against SILVA (LSU/SSU) Database^4^ using Bowtie2^5^ to eliminate rRNA contaminant reads. The filtered reads were *de novo* assembled using Trinity software^6^ with default parameters, using a de Bruijn graph algorithm and a k-mer length of 25. The quality of transcriptome assembly was evaluated by mapping the reads over the assembled contigs with bowtie2. Completeness was analyzed with Benchmarking Universal Single-Copy Orthologs (BUSCO) using the Arthropoda odb9 database,^7^ and the number of fully reconstructed coding transcripts was evaluated by a BLASTx search against Swiss-Prot database with a cut off E-value ≤ 1e-20. All raw reads have been deposited in the sequence reads archive (SRA) at National Center for Biotechnology Information (NCBI), and may be accessed using the access code PRJNA772728.

### Homology Search and Annotation

The generated contigs were analyzed by searching homology with a BLASTx tool against public databases, such as the non-redundant protein database (nr) filtered by Insecta Taxa (NCBI), Uni-Prot TrEMBL, and Swiss-Prot with a cut off E-value ≤ 1e-5. InterproScan available in OmicsBox software^8^ was used for conserved domains search (Zdobnov and Apweiler, 2001). Gene ontology (GO) categories were assigned from pooled BLAST and InterPro hits according to the pipeline available in the Blast2Go software (Götz et al., 2008).

### RNAi Related Genes

Genes related to RNAi machinery classified as core RNAi (Table 1), auxiliary RISC factors (Table 2), uptake, nucleases, antiviral, and intracellular transport proteins (Table 3) previously described were used as reference for the search in the *P. guildinii* transcriptome (Prentice et al., 2015; Taning et al., 2016; Cagliari et al., 2020). Homologous sequences for these proteins were obtained from NCBI and used as queries in a tBLASTn search with a cut off E-value < 1e-5. Hits with the lowest E-value were further analyzed to confirm their identity. An ORF Finder tool from NCBI^9^ was used to predict Open Reading Frames and protein domains were predicted by the NCBI Conserved Domains Database.^10^ A BLASTp search against non-redundant protein database at NCBI was performed as well. To provide additional confirmation on the identity of core RNAi proteins, a phylogenetic analysis was performed. Proteins sequences from different insect orders were aligned using MUSCLE program from MEGA 7.0.26, and neighbor-joining algorithm with 1,000 bootstrap replicates was used to predict phylogeny.

**TABLE 1:** Core RNAi related genes identified in *Piezodorus guildinii* transcriptome.

Gene ID	Homolog ID – species	*P. guildinii* ID	Comparison	% Identity
**miRNA**				
*DCR-1*	AVK59457.1 – *Nezara viridula*	TRINITY_DN8985_c0_g1_i5	E = 0.0 – bits = 1,321	93.56
*AGO-1 isoform 1*	AVK59466.1 – *Nezara viridula*	TRINITY_DN2687_c0_g1_i1	E = 0.0 – bits = 1,257	99,84
*Loquacious*	XP_014274312.1 – *Halyomorpha halys*	TRINITY_DN19426_c0_g1_i2	E = 0.0 – bits = 739	95.32
*Drosha*	XP_014278529.1 – *Halyomorpha halys*	TRINITY_DN28734_c0_g1_i1	E = 0.0 – bits = 1,442	92.75
*Pasha/DGCR8*	XP_014282581.1 – *Halyomorpha halys*	TRINITY_DN3240_c0_g1_i1	E = 0.0 – bits = 1,210	87.41
*Exportin-5*	XP_014280932.1 – *Halyomorpha halys*	TRINITY_DN9946_c0_g1_i3	E = 0.0 – bits = 2,405	85.60
**siRNA**				
*DCR-2*	XP_014275310.1 – *Halyomorpha halys*	TRINITY_DN9350_c0_g1_i2	E = 0.0 – bits = 2,808	83.81
*Ago-02*	AVK59468.1 – *Nezara viridula*	TRINITY_DN2417_c0_g1_i1	E = 0.0 – bits = 1,582	81.26
*R2D2*	XP_014288218.1 – *Halyomorpha halys*	TRINITY_DN2682_c1_g1_i1	E = 2.73e-170 – bits = 582	77.134
**piRNA**				
*Ago-3*	XP_014276831.1 – *Halyomorpha halys*	TRINITY_DN83747_c0_g1_i1	E = 0.0 – bits = 1,028	85.60
*Aubergine (AUB)*	XP_014275927.1 – *Halyomorpha halys*	TRINITY_DN5247_c0_g1_i2	E = 0.0 – bits = 1,195	65.38
*Piwi*	XP_014270559.1 – *Halyomorpha halys*	TRINITY_DN56355_c0_g1_i1	E = 0.0 – bits = 1,714	96.89
*Zucchini (Zuc)*	XP_014288409.1 *– Halyomorpha halys*	TRINITY_DN47842_c0_g2_i3	E = 3.52e-167 – bits = 468	92.41

**TABLE 2:** RISC-related genes identified in *Piezodorus guildinii* transcriptome.

Gene ID	Homolog ID – species	*P. guildinii* ID	Comparison	% Identity
*Tudor-SN*	XP_014284230.1 – *Halyomorpha halys*	TRINITY_DN7506_c0_g1_i1	E = 0.0 – bits = 1,660	75.37
*Translin*	XP_014290495.1 – *Halyomorpha halys*	TRINITY_DN9072_c0_g1_i2	E = 8.5e-162 – bits = 513	86.40
*Similar to translin associated factor-X (TRAX)*	XP_014289754.1 – *Halyomorpha halys*	TRINITY_DN5790_c0_g1_i5	E = 1.50e-160 – bits = 613	87.60
*Armitage*	XP_014289817.1 – *Halyomorpha halys*	TRINITY_DN4232_c0_g1_i1	E = 0.0 – bits = 2,098	95.52
*Homeless (spindle-E)*	XP_014286769.1 – *Halyomorpha halys*	TRINITY_DN8326_c0_g1_i2	E = 0.0 – bits = 2,285	87.77
*Maelstrom*	XP_014290039.1 – *Halyomorpha halys*	TRINITY_DN4191_c0_g1_i1	E = 0.0 – bits = 449	82.56
*HEN1*	XP_014284423.1 – *Halyomorpha halys*	TRINITY_DN3220_c0_g1_i1	E = 0.0 – bits = 1,264	67.36
*PRP16, mut6 homolog*	XP_014279344.1 – *Halyomorpha halys*	TRINITY_DN6502_c0_g1_i1	E = 0.0 – bits = 2,428	98.81
*Clp1 homolog (kinase)*	XP_014275582.1 – *Halyomorpha halys*	TRINITY_DN2409_c0_g1_i16	E = 0.0 – bits = 2,821	96.43
*Elp-1*	XP_014290480.1 – *Halyomorpha halys*	TRINITY_DN1844_c0_g1_i1	E = 0.0 – bits = 2,104	85.16
*GLD-1 homolog*	XP_014290348.1 – *Halyomorpha halys*	TRINITY_DN6361_c0_g2_i1	E = 0.0 – bits = 1,058	85.96
*ACO-1 homolog*	XP_014275296.1 – *Halyomorpha halys*	TRINITY_DN320_c0_g1_i9	E = 0.0 – bits = 1,709	94.14
*Vasa intronic gene (VIG)*	XP_014292052.1 – *Halyomorpha halys*	TRINITY_DN5670_c0_g1_i1	E = 0.0 – bits = 782	96.57
*Staufen*	XP_014282526.1 – *Halyomorpha halys*	TRINITY_DN4585_c0_g1_i5	E = 0.0 – bits = 1,323	95.83
*RNA helicase Belle*	XP_014279436.1 – *Halyomorpha halys*	TRINITY_DN10378_c0_g3_i2	E = 0.0 – bits = 340	98.11
*Protein arginine methyltransferase 7 (PRMT)*	XP_014292128.1 – *Halyomorpha halys*	TRINITY_DN4183_c0_g1_i2	E = 0.0 – bits = 749	87.11
*Gawky*	XP_014288686.1 – *Halyomorpha halys*	TRINITY_DN12193_c0_g2_i6	E = 0.0 – bits = 2,830	98.59
*Similar to fragile X mental retardation syndrome related protein 1 (FXMR1)*	XP_969396 E – *Tribolium castaneum*	TRINITY_DN1626_c0_g1_i4	E = 0.0 – bits = 613	71.18
*Gemin 3 homolog*	EFA00789 *– Tribolium castaneum*	TRINITY_DN55620_c0_g1_i1	E = 0.0 – bits = 427	49.76
*p68 Helicase*	NP_001164095 *– Tribolium castaneum*	TRINITY_DN10378_c0_gi_i5	E = 0.0 bit = 688	73.85

**TABLE 3:** Uptake, nucleases, antiviral, and intracellular transport genes identified in *Piezodorus guildinii* transcriptome.

Gene ID	Homolog ID – species	*P. guildinii* ID	Comparison	% Identity
**Uptake**				
*Scavenger*	XP_024218066.1 – *Halyomorpha halys*	TRINITY_DN11492_c2_g2_i1	E = 0.0 – bits = 1,026	95.99
*CG4966* = *orthologous to the Hermansky-Pudlak Syndrome4 (HSP4)*	XP_014288755.1 – *Halyomorpha halys*	TRINITY_DN9038_c0_g1_i1	E = 0.0 – bits = 897	89.00
*F-box protein 11 (FBX011)*	XP_014287303.1 – *Halyomorpha halys*	TRINITY_DN19510_c0_g1_i1	E = 0.0 – bits = 1,799	99.17
*Clathrin heavy chain (Chc)*	XP_014287090.1 – *Halyomorpha halys*	TRINITY_DN2469_c0_g1_i1	E = 0.0 – bits = 3,485	99.52
*AP2u (Ap50)*	NP_001280510.1 – *Tribolium castaneum*	TRINITY_DN5859_c0_g1_i3.	E = 0.0 bits = 866	94.33
*ADP-ribosylation factor-like protein 1 (Arl1)*	EFA02719.2 – *Tribolium castaneum*	TRINITY_DN8242_c0_g1_i1	E = 7.14e-115 bits = 323	85.55
*Eater*	XP_969372 – *Tribolium castaneum*	TRINITY_DN758_c1_g1_i2	E = 7.57e-47 – bits	36.79
*Epsin 2 (Epn2)*	XP_014270392.1 – *Halyomorpha halys*	TRINITY_DN2686_c2_g1_i4	E = 0.0 – bits = 1,026	91.21
*Gap Junction protein (Innexin2)*	XP_014292574.1 – *Halyomorpha halys*	TRINITY_DN2653_c0_g1_i1	E = 0.0 – bits = 1,026	99.44
**Nucleases**				
*Exoribonuclease 1 (Eri1)*	XP_014290344.1 – *Halyomorpha halys*	TRINITY_DN6568_c0_g1_i4	E = 0.0 – bits = 1,026	84.22
*DNA/RNA non-specific endonuclease isoform 1*	XP_024218583.1 – *Halyomorpha halys*	TRINITY_DN4766_c0_g1_i2	E = 1.03e-173 bits = 494	82.05
*DNA/RNA non-specific endonuclease isoform 3*	XP_014293261.1 – *Halyomorpha halys*	TRINITY_DN14109_c0_g1_i4	E = 0.0 – bits = 687	74.51
*Small RNA degrading nuclease 1 (SDN1-like)*	XP_014279339.1 – *Halyomorpha halys*	TRINITY_DN36580_c0_g1_i1	E = 0.0 – bits = 871	75.46
*Exosome*	XP_014288410.1 – *Halyomorpha halys*	TRINITY_DN30464_c0_g1_i1	E = 0.0 – bits = 1,845	94.64
*PolyApolymerase*	EFA00912 – *Tribolium castaneum*	TRINITY_DN5556_c0_g1_i1	E = 0.0 – bits = 1,026	65.37
*Nibbler*	XP_024216394.1 – *Halyomorpha halys*	TRINITY_DN76599_c0_g1_i1	E = 0.0 bit = 1,424	85.76%
**Antiviral**				
*Ars2*	XP_014277995.1 – *Halyomorpha halys*	TRINITY_DN4735_c0_g1_i1	E = 0.0 – bits = 1,507	94.90
*NinaC*	XP_014281724.1 – *Halyomorpha halys*	TRINITY_DN11848_c0_g1_i1	E = 0.0 – bits = 1,097	95.08
*Egghead Beta 1,4-mannosyltransferase (egh)*	XP_014283435.1 – *Halyomorpha halys*	TRINITY_DN10121_c0_g1_i1	E = 0.0 – bits = 918	96.94
*CG4572*	XP_014280828.1 – *Halyomorpha halys*	TRINITY_DN91529_c0_g1_i1	E = 0.0 – bits = 870	90.13
**Intracellular transport**				
*Vacuolar H* + *ATPase sub unit A (vha68)*	XP_014272529.1 – *Halyomorpha halys*	TRINITY_DN3993_c2_g1_i1.p1	E = 0.0 – bits = 1,256	99.35
*Vacuolar H* + *ATPase sub unit C (vha16)*	XP_014275063.1 – *Halyomorpha halys*	TRINITY_DN1028_c0_g3_i1.p1	1.13e-102 bits = 298	98.08
*Small Rab GTPases*	XP_014286452.1 – *Halyomorpha halys*	TRINITY_DN3137_c0_g1_i1.p1	4.24e-154 bits = 425	99.03

### dsRNA Synthesis and Purification

*Piezodorus guildinii* transcriptome was screened for *vATPAase A* nucleotide sequence using as a query *H. halys* protein sequence XM_014417043.2 (Table 3). A 300 bp sequence showing low cross-reactivity to other organisms evaluated by BLASTp against nr-NCBI database was selected. A 496 bp green fluorescent protein (GFP) dsRNA was designed from pRFHUE-eGFP plasmid Addgene (Watertown, United States). Plasmid DNA was purified from transformed *Escherichia coli* DH5α cells according to Green and Sambrook (2016) protocol. Specific primers were designed to amplify DNA templates that included T7 promoter sequence placed at the 5′-end of both forward and reverse primer to enable *in vitro* transcription (Table 4). Further, 1 μg of total RNA from adults *P. guildinii* was used as template to synthesize cDNA and oligodT primer using SuperScript™ IV Reverse Transcriptase Thermo Fischer Scientific (Waltham, United States), according to instructions from manufacturer. Templates were amplified from cDNA by PCR with 2.5 μl of cDNA template, 5 μl of 10× Buffer Thermo Scientific (Waltham, United States), 1.8 mM MgCl_2_, 0.25 mM DNTPs, 0.25 mM of each primer, and 2.5 U of Taq Polymerase Thermo Scientific (Waltham, United States) in a 50 μl reaction. PCR cycle consisted of 5 min at 94°C, 35 cycles of 30 s at 94°C, 30 s at 58°C, 45 s at 72°C, and a final extension of 10 min at 72°C. PCR products were purified by isopropanol precipitation, quantified by Nanodrop 8000 Thermo (Waltham, United States), and analyzed by agarose 1.5% gel electrophoresis. Sequences were confirmed by Sanger sequencing in Macrogen Inc. (Seoul, South Korea). DsRNAs were synthesized using a MEGAScript kit Thermo Fisher Scientific (Waltham, United States) from 1.2 μg of PCR product in a 16 h reaction and then purified by phenol:chloroform extraction following the manufacturer’s instructions. Integrity was evaluated by agarose 1.5% gel electrophoresis, and, finally, concentration was determined in a Nanodrop 8000 Thermo (Waltham, United States). Aliquots were stored at −80°C.

**TABLE 4:** Primers used for dsRNA synthesis and RT-qPCR.

Gene name	Primer	Sequence 5′–3′	Amplicon (bp)	Amplification efficiency (%)
dsvATPase A	Fw	TAATACGACTCACTATAGGGAGATATCCAGCGACCCCTGAAG	300	−
	RV	TAATACGACTCACTATAGGGAGATTAGTTTTCTCACCATCAAACTCTG		
ds GFP	Fw	TAATACGACTCACTATAGGGAGAATGGTGAGCAAGGGCGAG	496	−
	RV	TAATACGACTCACTATAGGGAGATGTTCTGCTGGTAGTGGTCG		
18S	Fw	GTGCTTTGCAGTGGTTGTGT	107	99.3
	RV	TCGGGCCGTTCGACTTAATG		
60S	Fw	GCTCCCAAGATCGGTCCTCT	119	96.8
	RV	TGCCTGTTTTGAATAGTGAGGC		
vATPase A	Fw	AATTGTGCAGCTGGTCGGTA	127	99.6
	RV	TGGGCAGAACCGATCGTAAG		
Dcr-2	Fw	ACATTGCTGATGGAACGGGAT	84	104.9
	RV	AGGCTGTTTGGTCGACTTCC		
Ago-2	Fw	TACGGCAGAGACCTCCATCA	102	102.6
	RV	GAGGAGGTCCTCTTTGTGCC		

*T7 Promoter sequence is underlined. Amplicon size is indicated, as well as primer efficiency when calculated.*

### Adult dsRNA Injection

RNA interference in *P. guildinii* was evaluated by injection of 1.2 μg of dsRNA in adults (28 ng/mg of insect, average adult weight ∼45 mg) based on previous reports (Castellanos et al., 2019; Cagliari et al., 2020; Sharma et al., 2020). A 300 bp dsRNA targeting *vATPase A* was used to evaluate silencing and a 496 bp GFP dsRNA was used as control. Adults were anesthetized by incubation on ice for 5 min and then placed with the ventral side up under a binocular loupe (2×) (Supplementary Figure 3). A volume of 0.5 μl of dsRNA (2.4 μg/μl) was injected in the ventral septum between the thoracic and abdominal segments using a 25 μl Hamilton syringe with a 33G needle coupled to a PB600 repeater Hamilton (Reno, United States). Control group was injected with water. After injections, insects were placed in plastic plates containing green beans, peanut, and water *ad libitum*, and kept in the conditions previously described. For survival assays 30 adults were used per group, and mortality was evaluated every day for 14 days. Each assay was repeated two times. Survival curves were compared using the log-rank test (*p* < 0.01). For gene silence evaluation by real-time quantitative PCR (RT-qPCR), 27 adults were injected per group, and the pools of three individuals were processed at 24, 48, and 72 h post-injection by triplicate.

### Real-Time Quantitative PCR

Total RNA was purified from insect samples corresponding to 24, 48, and 72 h post-injection using TRIzol Reagent Ambion (Austin, United States), following manufacturer’s instructions. RNA was quantified using a Nanodrop 8000 (Thermo) and quality was evaluated by 1.5% agarose gel electrophoresis. Then, 1 μg of total RNA was treated with DNAase I NZY (Lisbon, Portugal) and used as template to synthesize cDNA, as described. Primers were designed using Primer-Blast from NCBI based on Primer3 software,^11^ and were validated with a standard curve based on serial dilution of cDNA to determine amplification efficiency, calculated as E = [10^(−1/slope) − 1] × 100 (Table 4). A melting curve analysis with temperature range from 60 to 95°C and agarose gel electrophoresis confirmed primer specificity. RT-qPCRs were performed in a QuantStudioTM 3 Real-Time PCR System (Applied Biosystems) in 96 well plates. Each reaction included 2 μl of cDNA dilution, 5 μl iQ SYBR Green Supermix 2X Bio-Rad (Hercules, United States), and 0.25 mM of each primer in a 10 μl reaction. Amplification conditions were 10 min at 95°C, 40 cycles of 15 s at 95°C, and 1 min at 60°C, and a melting curve stage from 60 to 90°C. Data were analyzed using the Quant Studio design and analysis software v1.4.3. As endogenous control, *18S ribosomal protein* (*18S*) and *60S ribosomal protein* (*60S*) described previously for *H. halys* (Mogilicherla et al., 2018) were checked for stability with RefFinder software (Xie et al., 2012) and used for data normalization. Relative gene expression was calculated using the equation 2^(–ΔΔCt)^ (Livak and Schmittgen, 2001). Normal distribution of the data was checked with Shapiro–Wilk test and Levene’s test to compare variances. The statistical significance was analyzed by an un-paired *t*-test (*p* < 0.05, *n* = 3) using R-studio.

## Results

### *Piezodorus guildinii* Transcriptome

Illumina sequencing led to 520,093,434 raw reads, corresponding to 260,046,717 unprocessed pairs with a GC content of 41.88%. Through the quality filtering process, 50,599,592 reads (19.4%) were eliminated (Supplementary Table 1). The *de novo* assembly of the *P. guildinii* transcriptome was performed with filtered reads resulting in 172,298 transcripts that correspond to 119,178 uni-genes. The assembled transcripts’ GC content was 35.57% and the average contig size was 750 bp. Assembly quality, as evaluated by mapping the filtered reads against the assembled transcripts, indicated that 97.49% of the paired reads were included in the assembly. Completeness of the transcriptome evaluated by BUSCO against an Arthropoda database showed that five out of 1,066 genes (0.5%) were not identified (Supplementary Table 1). Finally, the number of fully reconstructed transcripts was evaluated by means of a BLASTx search against the Swiss-Prot database, showing that 4,499 hits presented coverage greater than or equal to 90%.

**FIGURE 1:**
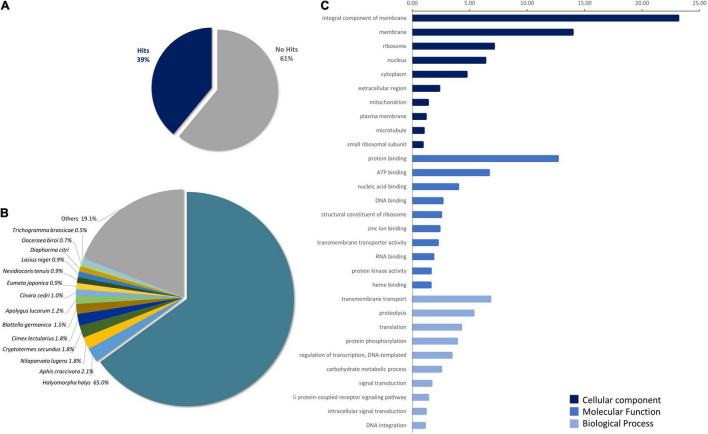
*Piezodorus guildinii* transcriptome homology analysis. **(A)** Percentage of hits identified by BLASTx in *P. guildinii* transcriptome against nr-NCBI, Swiss-Prot, and TrEMBLE databases (cutoff E-Value < 10e-5). **(B)** Species distribution of transcripts with known function, hits from nr-NCBI database. **(C)** Gene Ontology (GO) terms in *P. guildini* transcriptome. Percentage distribution of 10 principal GO terms classified in Molecular Function (MF), Biological Process (BP), and Cellular Component (CC) assigned to *P. guildinii* contigs. Analysis was performed using hits from BLASTx and InterProScan using OmicsBox software.

The functional annotation of transcripts was performed by BLASTx, 55,706 hits (36.8%) were obtained against an Insecta subset of nr-NCBI protein database, 33,490 against Swiss-Prot (22%), while the BLASTx using insect TrEMBLE database yielded 52,721 matches (36.8%). Taking together, 58,922 hits were obtained, corresponding to 39% of the transcriptome (Figure 1A). Interestingly, when databases are compared, 53.60% of transcripts were identified by all three. Analysis of the sequences identified by nr-NCBI insect database showed that 76.8% of the hits corresponded to Hemiptera order as follows; Pentatomidae: 65% (*H. halys, N. viridula*), Aphidae: 4% (*Aphis craccivora*, *Aphis glycines*), Delphacidae: 2.16% (*Nilaparvata lugens*, *Laodelphax striatellus*), Miridae: 2.08% (*Apolygus lucorum*, *Nesidiocoris tenterauis*), Hemicidae: 1.79% (*Cimex lectularius*), and Psylliae: 0.8% (*Diaphorina citri*). The remaining hits were distributed among species of the orders Hymenoptera 3.9%, Coleoptera 2.4%, Lepidoptera 2.16%, and Diptera 1.19% (Figure 1B and Supplementary Table 2). It should be noted that BLAST analysis is based on sequence homology, which is dependent on the database used and does not imply phylogenetic relationships. Additionally, InterPro search identified 41,172 hits (27.2%). Sequences identified by both BLASTx and InterPro were used for the assignment of GO terms. A total of 42,316 terms were obtained, 54.5% from the Molecular Function category (MF), 30.5% from the Biological Process category (BP), and 15.02% from the Cellular Component category (CC). The 10 main components from each category are detailed in Figure 1C.

### Identification of RNAi-Related Genes

To establish RNAi strategies as a tool to control the red banded stink bug, the assembled transcriptome was used to annotate contigs related to RNAi machinery by tBLASTn. A total of 56 contigs were identified and are detailed in Tables 1–3 and Supplementary Material.

Core RNAi machinery genes related to miRNA, siRNA, and piRNA pathways were predicted in the *P. guildinii* transcriptome (Table 1 and Supplementary Data 1). In relation to Dicer proteins, in the miRNA pathway, only a partial sequence of *Dcr-1* was found, consisting in two RNAase III domains and a Double stranded RNA-binding domain (dsRBD). On the other hand, *Dcr-2* from siRNA pathways contained all the conserved domains (an *N*-terminal helicase domain, a PAZ domain, two RNAase III domains, and a dsRBD), while Drosha protein sequence was found with two Ribonuclease III family domain and a dsRBD. To further analyze the identified sequences, a phylogenetic analysis was performed with Dicer sequences from the different orders of insects (Supplementary Figure 1). Three main clades were inferred, one containing Dcr-1, another with Dcr-2, and the third one with Drosha sequences. In all cases, *P. guildinii* proteins were grouped with Hemiptera Pentatomidae sequences from *H. halys*, *N. viridula*, and *E. heros*. Within Dcr-1 subclade, *P. guildinii* Dcr-1 was grouped with *N. viridula*, while for Dcr-2, *P. guildinii* was grouped with *E. heros.* Drosha protein from *P. guildinii* was found in a clade with *E. heros* and *H. halys.* Co-factors Pasha, Loquacious, and R2D2 were identified with two dsRBD conserved domains. Regarding Argonaute, *Ago-1, Ago-2, Ago-3, Piwi*, and *Aubergine* were identified, all presented conserved domains PAZ and PIWI (Supplementary Data 1). Argonaute protein phylogeny showed three main clades, separating AGO subfamily (Ago-1 and Ago-2), Ago-3, and another clade with Aubergine and Piwi. In Ago subfamily, *P. guildinii* Ago-1 was grouped with *E. heros, H. halys*, and *N. viridula*, but in a different final branch, showing minor differences. Regarding Ago-2, *P. guildinii* protein was clustered with *N. viridula* and *H. halys*. In the PIWI subfamily, *P. guildinii* Ago-3 was clustered with *E. heros* and *H. halys*, while Piwi and Aubergine were clustered with *E. heros* (Supplementary Figure 2). In addition, the endoribonuclease Zucchini, essential for the primary piRNA biogenesis and Exportin5, a protein related to the nuclear export of pre-miRNAs was also identified.

The presence of auxiliary factors of the RISC complex was examined. Overall, 20 sequences were identified related to intracellular factors that are associated with the activity of the RISC complex (Table 2). Holo-RISC related proteins *Tudor-SN*, *Vasa-intronic gene*, and *fragile X related protein 1* were annotated with all conserved domains predicted. *Translin* and *Translin-associated factor X* (*TRAX*) components of C3P0 were also found. Additionally, nucleases involved in piRNA biogenesis, *Armitage, Homeless (splindle-E)*, and *Maesltrom* were present as well. As detailed in Table 2, other contigs identified were *GLD-1* homolog, *Aco-1, Elp-1, RNA Belle helicase*, *Gawky, Staufen, helicase p68*, and *Clp-1.* Partial sequences were identified from *Hen-1 metyltransferase*, without a dsRBD2 nor methyltransferase conserved domains, *PRP16 helicase* sequence lacked one DEAH Helicase- domain, and *Arginine methyltransferase 7* did not present any SAM-dependent methyltransferase PRMT-type domain.

Nine proteins involved in dsRNA uptake were identified in *P. guildinii* transcriptome, as shown in Table 3. *Clathrin* heavy chain, *Clathrin adaptor AP50*, *Epsin-2*, and *HSP4* related to receptor mediated endocytosis were found. In addition, three proteins related to intracellular vesicle transport were also annotated, *Vacuolar H* + *ATPase subunit A (vha68*), *Vacuolar H* + *ATPase subunit C (vha16*), and *Small Rab GTPases*. Particularly, *Vacuolar H* + *ATPase subunit A (vATPase A)* was used as a target to evaluate RNAi response in *P. guildinii.* Putative dsRNA receptors Scavenger and Eater were described as well. Interestingly, transcripts coding for *sid-like* proteins were not found.

Regarding RNAi related nucleases, *Eri-1, SDN1-like*, and *Nibble* were found and all conserved domains were predicted. Two *DNA/RNA endonucleases* isoforms were found, but only isoform 1 contained a nuclease conserved domain (Supplementary Data 1). In addition, *Exosome complex exonuclease* and *Poly(A)polymerase* were described.

Antiviral RNAi related genes *Ars2* involved in RISC complex regulation, *Egghead*, a transmembrane-domain glycosyltransferase and *ninaC*, a protein related to vesicle transport, were identified as shown in Table 3.

### dsRNA Injection in *Piezodorus guildinii*

**FIGURE 2:**
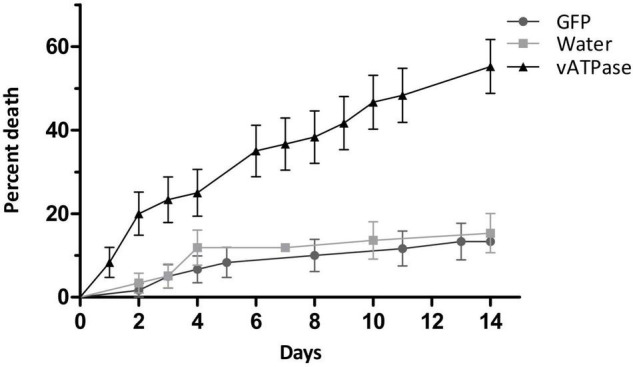
dsRNA injection in *P. guildinii.* Cumulative mortality expressed as percentage in *P. guildinii* adults after injection with dsRNA (28 ng/μl per mg of body weight) targeting *vATPase A*, green fluorescent protein (GFP), or water. Error bars represent SE from two independent assays.

The effect of dsRNA targeting *vATPase A* was studied by injection in *P. guildinii* adults. Significant differences were observed in the survival rates between insects treated with vATPase A, control GFP, or water assessed by log-rank test (χ^2^ = 42.76, df = 2 *p* < 0.0001). In vATPase A group, as shown in Figure 2, treated insects mortality was 35% after 7 days post-injection, reaching 51.6% after 14 days, while control groups behaved similarly. In GFP group, mortality ranged 10% on day 7 and 14% at the end of the assay; while in water injected insects, mortality was 11.5 and 15.5% after 7 and 14 days, respectively, as shown in Figure 2.

### RT-qPCR in Injection Assays

**FIGURE 3:**
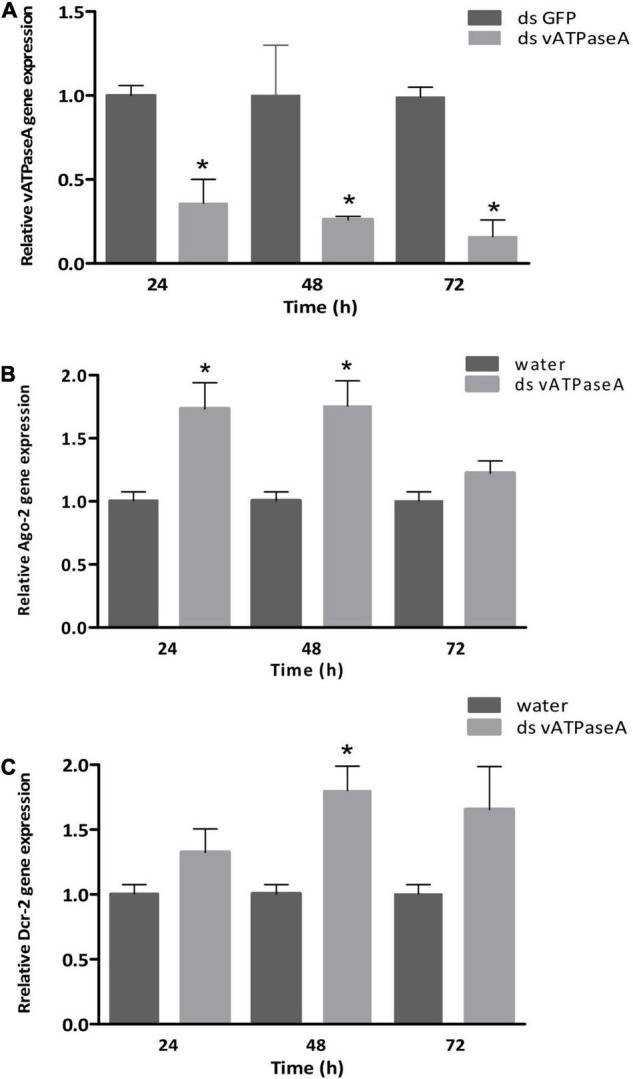
Effect of dsRNA injection on gene expression by RT-qPCR in *P. guildinii*. Adults were injected with 28 ng/μl per mg of body weight with dsRNA targeting *vATPase A* or GFP used as a negative control. Adults were sampled at 24, 48, and 72 h post-injection in both treatments. As internal control, *18S* and *60S* genes were used. **(A)** *vATPase A* relative expression, GFP treated insects were used for normalization. **(B)** *Ago-2* relative gene expression in dsRNA treated insects in comparison with water-treated insects. **(C)** *Dcr-2* relative expression of insects treated with ds-vATPase A; water-treated insects were used as control. Bars represent mean and SEM based on three biological repeats consisting of a pool of three insects each. Statistical significance was calculated by an unpaired *t*-test. *p ≤ 0.05.

To confirm *vATPase A* gene silencing after dsRNA injection, RT-qPCR analysis was performed using cDNA from treated insects. The *vATPase A* transcript level showed a significant reduction of 64.4% at 24 h after injection (*p* = 0.025), at 48 h, gene expression decreased 74% (*p* = 0.019), and 84% of reduction (*p* = 0.002) at 72 h compared with control samples injected with dsGFP (Figure 3A).

To assess the involvement of the siRNA machinery following dsRNA injection, the differential expression of the *Dcr-2* and *Ago-2* genes was analyzed by RT-qPCR. *Argonaute-2* showed a significant overexpression of 70 and 80% at 24 and 48 h, respectively, (*p* = 0.02); at 72 h, *Ago-2* expression was 1.3-fold, which was not statistically significant (Figure 3B). For *Dcr-2*, after 24 h a 30% overexpression was observed, but showed no statistical significance, while at 48 h, a 1.8-fold expression of *Dcr-2* was significantly different compared with control group (*p* = 0.04). A 1.6-fold expression was maintained at 72 h, even though this difference was not statistically significant (Figure 3C).

## Discussion

To evaluate the potential of RNAi as a tool in the control of the red-banded stink bug, we first generated a *P. guildinii* transcriptome, since little sequence information was available for this species. This approach allowed to get insight into the RNAi gene machinery and to gather information about possible target genes and housekeeping genes sequences. Recently, a complete genome assembly of *P. guildinii* has been reported (Perera, 2021-preprint). Taken together, these data will generate complementary information that will broaden the knowledge in the biology of this insect species. Homology analysis showed a 39% of contigs of known functions, revealing that *P. guildinii* transcriptome contains a high percentage of genes with non-assigned functions, a similar proportion of what was reported in *E. heros* transcriptome (Cagliari et al., 2020). The stink bug *H. halys* showed the highest homology from the identified transcripts, covering 65% of the hits; this could be explained by database coverage rather than phylogenetical homology (Ioannidis et al., 2014).

Differences in RNAi efficacy among insects can be partially explained by the diversity in the RNAi pathway genes present in different lineages (Dowling et al., 2016). To increase our understanding about *P. guildinii* RNAi response, we first analyzed the transcriptome for the annotation of genes related to this process. As shown in Tables 1–3, we were able to identify 56 sequences. Core components of the RNAi machinery with small RNA fragments are the main effectors in gene silencing. They can be divided into three pathways based on Dicers, Argonautes, and small RNAs involved. The siRNA pathway is activated by long exogenous or endogenous dsRNAs and involves Dicer-2 (Dcr-2), co-factor R2D2, and Argonaute-2 (Ago-2). The miRNA pathway involves miRNA from endogenous transcripts, nuclear Dicer, Drosha and co-factor Pasha, cytoplasmic Dicer-1 (Dcr-1), co-factor Loquatios, and Argonaute-1 (Ago-1). Finally, the piRNA pathway is germline-specific, derived from single-stranded RNA (ssRNA) and independent of Dicer proteins. It is characterized by endonuclease Zucchini (Zuc) and Argonaute proteins of the PIWI class: Aubergine, Piwi, and Argonaute-3 (Ago-3) (Ambrus and Frolov, 2009; Zhu and Palli, 2020). All the above-mentioned core proteins were identified in *P. guildinii* transcriptome (Table 1 and Supplementary Data 1). Particularly, *Dicer-1* lacked the *N*-terminal helicase and PAZ domain. Functional Dcr-1 lacking the *N*-terminal helicase domain has been reported previously in *D. melanoganster* (Lee et al., 2004; Kavi et al., 2008). In *P. guildinii*, *Dicer-1* resembled nuclear RNAase III Drosha protein (two RNAse III domains and a carboxyterminal dsRBD). It would be interesting to further study if this Dicer-1 is functional in *P. guildinii* miRNA pathway. The phylogenetic analysis of these protein sequences (Supplementary Figures 1, 2) further validated the identity of the annotated transcripts. *P. guildinii* proteins were grouped with Hempitera:Pentatomidae species *E. heros*, *N. viridula*, and *H. halys*. *Pasha*, *Loquacious*, and *R2D2* dsRNA binding proteins, co-factors of Drosha, Dicer-1, and Dicer-2, respectively, were also found with all their conserved domains. Although the structure of Ago proteins is highly conserved, the number of Ago proteins varies between different species from 1 in the fission yeast *Schizosaccharomyces pombe* to 27 in the nematode worm *Caenorhabditis elegans* (Höck and Meister, 2008; Meister, 2013). In insects, five Argonautes were reported for *D. melanoganster, T. castaneum, Cylas puncticollis*, and among others. Five members belonging to the Argonaute superfamily were identified in *P. guildinii* as well (Table 1). Argonaute subfamily: *Ago-1* and *Ago-2*; and PIWI subfamily: *Ago-3, Aubergine*, and *Piwi*. In all cases, conserved PAZ and Piwi domains were predicted. Interestingly, the phylogenetic analysis showed that identified sequences were grouped within Hemiptera:Pentatomidae species clades with a common ancestor (Supplementary Figure 2). In addition, the *Zucchini* endonuclease involved in piRNA maturation was also annotated. RdRP protein that can amplify synthesizing small RNAs or dsRNAs on targeted RNA templates was not found. This is consistent with previous reports that indicate that no homologs of this protein have been described in vertebrate or insect genomes (Maida and Masutomi, 2011).

We then analyzed auxiliary RISC factors associated with the activity of the RISC complex. In siRNA described in *D. melanoganster*, Holo-RISC or the mature form of RISC is composed by Dcr-2 and R2D2 (components of the RISC Loading Complex), proteins Ago-2, Tudor-SN (Gutierrez-Beltran et al., 2016), Vasa intronic gene, Fragile X related protein (FXMR1) (Caudy et al., 2002), and C3P0, an heterodimer of Translin and Trax proteins (Kavi et al., 2008; Liu et al., 2009). Sequences for all the proteins mentioned were identified in *P. guildinii* transcriptome with their conserved domains predicted as shown in Table 2. In addition, *Clp-1 kinase* related to siRNAs phosphorylation before RISC loading was also annotated (Davies and Samuels, 2010). Helicases play different functions in the silencing processes, from unwinding RNA duplexes to proper RNA loading to RISC (Ambrus and Frolov, 2009). Helicases *Belle*, *Armitage*, *p68 RNA helicase*, *Gemin 3*, and a partial sequence of *PRP16* were identified in *P. guildinii*. In addition, Piwi pathway related proteins *Spindle E*, *Maelstrom*, *Armitage*, *methyltransferases PRMT*, and a partial sequence of *Hen-1* were also found. Other auxiliary factors, such as *Elp-1*, a component of pol II core elongation complex, *Staufen, GLD-1*, and *ACO-1* were present as well.

Internalization of dsRNA into cells is an essential step for the generation of RNAi. Two different pathways have been described for dsRNA uptake in insects, Sid-1 channel protein-mediated pathway and the endocytic pathway (Joga et al., 2016). The first pathway, described in *C. elegans*, involves transmembrane proteins SID-1 and SID-2 (Hunter et al., 2006). Orthologous proteins to *C. elegans* SID-1, called SID-like proteins (SIL-A, SIL-B, and SIL-C), have been identified in several insect species, although their direct involvement in dsRNA uptake is still not clear (Vélez and Fishilevich, 2018). SID-2 has not been reported in any insect order (Hinas et al., 2012). In *P. guildinii* transcriptome, *SilA, SilB*, and *SilC* homologous sequences were absent (Table 3), similarly in Diptera, where no *SID-like* genes have been reported, and more recently in the Hemiptera *E. heros* transcriptome (Tomoyasu et al., 2008; Huvenne and Smagghe, 2010; Cagliari et al., 2020). The other uptake pathway described is Clathrin-mediated endocytosis. The sequences identified in the present transcriptome encompassed the endocytic pathway (Table 3 and Supplementary Material), from early vesicle formation, *Clathrin heavy chain (Chc), Clathrin adapter protein AP50*, to the late endosomal formation and release, *HPS4, Rab7*, endosome acidification proteins: *Vacuolar H* + *ATPase sub unit A (vha68)* and *Vacuolar H* + *ATPase sub unit C (vha16)*, as reported previously (Vélez and Fishilevich, 2018). These findings suggest that besides the absence of SID-like proteins, dsRNA uptake could be mediated by endocytosis in *P. guildinii*. In Drosophila S2 cells, scavenger receptors, SR-CI and Eater, account for more than 90% of dsRNA uptake (Saleh et al., 2006; Ulvila et al., 2006). A *scavenger receptor class B* and *Eater* sequence was annotated in *P. guildinii* (Table 3 and Supplementary Material); further functional evaluation of these proteins would be needed to determine if they have an active role in dsRNA uptake.

A rapid degradation of dsRNA by dsRNases causes a reduction in RNAi response (Cooper et al., 2019). We described the presence of seven nucleases in the transcriptome of *P. guildinii* (Table 3 and Supplementary Material): *Eri-1*, an evolutionarily conserved 3′–5′ exoribonuclease related in siRNA and miRNA pathways (Kennedy et al., 2004; Thomas et al., 2014), *Sdn1-like protein*, a 3′–5′ exonuclease related to the degradation of mature miRNA (Ramachandran and Chen, 2008). In addition, *Nibble*, a 3′–5′ exoribonuclease described in *Drosophila* to process 3′-end trimming in miRNA pathway (Han et al., 2011) and a *Poly(A)polymerase*, involved in the mRNA degradation (Yamanaka et al., 2013). Most insects possess 2–4 dsRNase genes, as revealed by genome-wide analysis (Cooper et al., 2019). In *P. guildinii* transcriptome, we found two sequences, but only isoform 1 showed a nuclease conserved domain (Table 3 and Supplementary Material). *Exosome complex exonuclease*, a 3′−5′ exonuclease (Morlando et al., 2008) reported in *N. viridula* saliva, was also present (Lomate and Bonning, 2016). In addition, we report here the presence of anti-viral proteins (Table 3 and Supplementary Material), such as *Ars2*, related to the regulation of the RISC complex, (Sabin et al., 2009), *Egghead* (*Egh)*, a seven transmembrane-domain glycosyltransferase, *nina C*, a protein involved in vesicle transport, and *C4572* protein, a carboxypeptidase with unknown function (Saleh et al., 2009).

Once we identified transcripts homologous to essential RNAi genes in *P. guilidinii* transcriptome, we evaluated *in vivo* whether the machinery was functional after an injection of a dsRNA targeting *vATPase A*. Vacuolar-type proton pumping ATPase (V-ATPase) is a ubiquitous enzyme responsible for proton (H^+^) transport across membranes and the acidification of cellular compartments (Utai et al., 2019). Silencing and mortality after an administration of dsRNA vATPase A have been reported in different insect orders, such as Coleoptera (Baum et al., 2007; Whyard et al., 2009; Christiaens et al., 2016), Diptera (Whyard et al., 2009), Lepidoptera (Whyard et al., 2009; Camargo et al., 2016), Homoptera (Whyard et al., 2009), and Hemiptera (Singh et al., 2015; Basnet and Kamble, 2018). In *P. guildinii*, an injection of 28 ng/mg of body weight of dsRNA targeting vATPase A showed a mortality of 35% after 7 days, and of 51.6% after 14 days of injection. These results show that *P. guildinii* is susceptible to RNAi, with a significant mortality effect. In *E. heros*, an injection of dsRNA against *vATPase A* at the same concentration caused a mortality of 35% in adults 4 days after injection (Cagliari et al., 2020), while in second instar nymph a 50% mortality was shown after 7 days, reaching 80% after 14 days (Castellanos et al., 2019). A similar observation was reported in *N. viridula*, where second instar nymphs injected with 40 ng/mg of body weight of dsRNA against *vATPase A* showed 63 and over 80% mortality after 7 and 14 days, respectively (Sharma et al., 2020), while in adults, mortality rates were not significantly different to the control group (Gurusamy et al., 2021). These reports point out a differential response to RNAi regarding developmental stages in stink bugs. Further analysis in *P. guildinii* at different life stages could show higher mortality rates.

The effect of dsRNA injection was further assessed by RT-qPCR. The relative expression of *vATPase A* at 24, 48, and 72 h after injection was significantly reduced to 84% at 72 h, which is in accordance with a functional RNAi machinery. Moreover, *Dicer-2* and *Argonaute-2*, two fundamental core components of the process, were significantly upregulated at 48 h as well. Even though at 72 h, the levels of these proteins were not significantly different from control non-treated samples, the effect in the reduction of the expression of *vATPase A* was maintained. The upregulation of core siRNA enzymes in response to exogenous dsRNA occurs in many insect species, and can be related not only to the exposure to dsRNA, but also to environmental factors, and pathogens have an influence as well (Cooper et al., 2019).

The establishment of RNAi pest control strategies is a complex task affected by several variables raging from the presence and copy number of core RNAi machinery proteins, the selection of target genes and dsRNA design to delivery strategies that must overcome nuclease degradation, dsRNA cellular uptake, and systemic spreading (Jain et al., 2021). In this context, the present work generated a dataset that will be useful for the selection and design of new target genes to be further evaluated. In addition, the direct effect of dsRNA on insect survival was evaluated bypassing the salivary system and the digestive tract, which may contain enzymes that degrade RNA and may reduce the effect observed, as reported previously in *E. heros* and *N. viridula* (Castellanos et al., 2019; Sharma et al., 2021). Delivery strategies to provide dsRNA protection to nuclease activity must be evaluated to achieve a robust *P. guildinii* control using RNAi.

## Conclusion

In this work, we described the assemble and analysis of *P. guildinii* transcriptome focusing on RNAi machinery. In this sense, we described the annotation of RNAi machinery-related genes and showed that this process is functional in *P. guildinii* by injection of dsRNA into adults. The administration of dsRNA targeting *vATPase A* showed a reduction in the expression of this gene and it had significant effect on survival rates. Taken together, these results show that RNAi could be a potential tool for the development of new control strategies in *P. guildinii* in soybean crops.

## Data Availability Statement

The datasets presented in this study can be found in online repositories. The names of the repository and accession number can be found below: https://www.ncbi.nlm.nih.gov/, PRJNA772728.

## Author Contributions

MD-R, CS, and MM conceived and designed the experiments. CS and SM performed the experiments. CS and PF analyzed the data. CS and MD-R wrote the manuscript. All authors have discussed the findings, interpreted the results, read, and approved the final manuscript.

## Conflict of Interest

The authors declare that the research was conducted in the absence of any commercial or financial relationships that could be construed as a potential conflict of interest.

## Publisher’s Note

All claims expressed in this article are solely those of the authors and do not necessarily represent those of their affiliated organizations, or those of the publisher, the editors and the reviewers. Any product that may be evaluated in this article, or claim that may be made by its manufacturer, is not guaranteed or endorsed by the publisher.

## Footnotes

1
http://soystats.com/international-adoption-of-biotech-enhanced-seedstock/

2
http://www.bioinformatics.babraham.ac.uk/projects/fastqc

3
https://www.bioinformatics.babraham.ac.uk/projects/trim_galore/

4
https://www.arb-silva.de

5
http://bowtie-bio.sourceforge.net/bowtie2/

6
http://trinityrnaseq.sourceforge.net/

7
https://busco.ezlab.org/

8
https://www.biobam.com/omicsbox

9
https://www.ncbi.nlm.nih.gov/orffinder/

10
https://www.ncbi.nlm.nih.gov/Structure/cdd/wrpsb.cgi

11
https://www.ncbi.nlm.nih.gov/tools/primer-blast/
