# Supplementary material for: RNAi in Piezodorus guildinii (Hemiptera: Pentatomidae): Transcriptome Assembly for the Development of Pest Control Strategies
Source: Front Plant Sci. 2022 Apr 1;13:804839. doi: 10.3389/fpls.2022.804839 (PMC9011191; doi:10.3389/fpls.2022.804839)
Supplement: Supplementary file 1 [file Data_Sheet_1.PDF]

Supplementary Material

Table S1. Illumina sequencing and de novo assembly statistics of *P. guildinii* transcriptome

**Sequencing and Filtering**	
Total Reads	520.093.434
Paired Reads	260.046.717
Total bases (pb)	78.534.108.534
GC (%)	41.88
Q20(%)	98.11
Q30(%)	94.44
Filtered reads	50.599.592
***de novo* assembly metrics**	
Total transcripts	172.298
Total Genes	118.178
GC %	35.57
Contig N50	1.419
Average contig length	752.84
Median contig length	373
Total assembled bases	129.713.353
**Read alignment metrics**	
Paired aligned	208.995.243
Aligned once	203.703.447
Not aligned	5.251.796
**BUSCO metrics**	%
Complete	99
Complete and single copy	50.4
Complete and duplicated	48.6
Fragmented	0.6
Missing	0.4

Table S2 Species distribution of BLASTx (e-value 1e-5) against the Nr-NCBI database filtered by Insecta (Taxonomy ID 50557), first 40 Hits are shown.

	Species	Hits	%
1	*Halyomorpha halys*	36614	65.00
2	*Aphis craccivora*	1200	2.13
3	*Nilaparvata lugens*	1032	1.83
4	*Cryptotermes secundus*	1022	1.81
5	*Cimex lectularius*	1009	1.79
6	*Blattella germanica*	840	1.49
7	*Apolygus lucorum*	671	1.19
8	*Cinara cedri*	545	0.97
9	*Eumeta japonica*	529	0.94
10	*Nesidiocoris tenuis*	490	0.87
11	*Lasius niger*	482	0.86
12	*Diaphorina citri*	447	0.79
13	*Ooceraea biroi*	375	0.67
14	*Trichogramma brassicae*	303	0.54
15	*Rhagoletis zephyria*	282	0.50
16	*Photinus pyralis*	278	0.49
17	*Aphis glycines*	268	0.48
18	*Lucilia cuprina*	267	0.47
19	*Frankliniella occidentalis*	241	0.43
20	*Ephemera danica*	237	0.42
21	*Riptortus pedestris*	236	0.42
22	*Plutella xylostella*	233	0.41
23	*Sitophilus oryzae*	222	0.39
24	*Trachymyrmex cornetzi*	196	0.35
25	*Arctia plantaginis*	177	0.31
26	*Laodelphax striatellus*	175	0.31
27	*Agrilus planipennis*	166	0.29
28	*Callosobruchus maculatus*	157	0.28
29	*Dendroctonus ponderosae*	157	0.28
30	*Hyposmocoma kahamanoa*	146	0.26

31	*Trachymyrmex septentrionalis*	140	0.25
32	*Acyrthosiphon pisum*	134	0.24
33	*Camponotus floridanus*	129	0.23
34	*Ignelater luminosus*	127	0.23
35	*Papilio xuthus*	127	0.23
36	*Diabrotica virgifera virgifera*	124	0.22
37	*Chilo suppressalis*	124	0.22
38	*Bombyx mori*	123	0.22
39	*Aedes albopictus*	116	0.21
40	*Zootermopsis nevadensis*	113	0.20

Data S1. Sequences of *P. guildinii* RNAi predicted genes classified by Core, RISC related proteins, uptake, nucleases, antiviral and intracellular transport. For each gene, Transcriptome Accession number, nucleotide sequence, translated protein sequence, conserved domains and first hit BLASTp are detailed.

Core RNAi machinery genes

miRNA

Dicer-1

>TRINITY_DN8985_c0_g1_i5 len=2422 path=[0:0-1388 3:1389-2254 4:2255-2255 7:2256-2421]

GCTAAAGATGAATTATTAGATGAAAATGAAAGTAAAATCATTAACAAAGAAAAAGAAGAATCTGATTCTG
ATGATGAAACAAACAAAGATTCTGCCAACGAAGAAGATGTTAAAGATAAGGGTGACTGGATGGAAATAGG
AACTTGGTCCAATGACATGGCTCAGCTTGATGATGAGCTTCAAAAAGACCTCCCTCCAGAACTCTCAATGG
TGCGCTATGGGTCCCCAACTAGTTGGAATGTCGGTGAAAATCAATCATTTAACTACTACAGCTCTGACTGTG
AATCTGATGATTCGAGTGAAATATATTTAGAATCTGAAACATCCGATTCATCTGATGGAGGTGCTGGAAAA
TTAAAAATCACTTTCAGGGGAGATTACCTGGCAGAAGCCATTGAAGAAAATGAGCCTGAACCTAAGAAAG
AAAACAGTAATAGCATGAAATTAGACCTTGAATTGTGGACTTGGGATACTGAAGATTTAATTTCTAATGTT
AAGGAAAGTATCCATGAAGAAACTGAAGAATTTCAGAAGCAAACTGAGCAACAAATGGAAATAATAAAAT
TCCAAAATAAACTTATTAAAAAAGAAGATCAGTTTGTAATAAATAAAGAGGATGGGAAAAAACAAGATAC
TGTTCCGGGAGAGATGAAGGATTTTGATTTCAAAGTAAGCAGCTATAGTATGCCTTCAGATATTATACCTAA
ATCATCAAAGAAAAAGTCTCTGTCCTACCATTATCCAGCTGAAACTATTAATAATGAAGAACTAGAGGAGG
GAATCACATTTAGTTTTGATCAGCAGCCAGAATTGGAAGGTCATCCAGGTCCTAGCCCTTCTATTCTACTCC
AAGCTTTGACCATGTCTAATGCTAATGACGGCATCAATCTAGAGCGTTTGGAAACTATTGGTGATTCCTTTC
TTAAGTATGCGATAACAACGTACCTATATTGTACTCATGAAAATATACATGAAGGAAAACTGAGCCATCTT
CGTTCTAAACAGGTTAGTAACTTGAAGCTTTATAGACTGGGACAAAAAAAGGTATTTGGTGAGAGTATGAT
TGCTAGTAAGTTTGAGCCACATGACAATTGGTTACCACCATGCTACTTAGTTCCTCGTGATGCTGAGCAAAG
GTTATTAGACTCAGAGCCTCAAGCTACTCTCCCATACAATCTTGTTACTCAACATTCTATTCCTGACAAAAG
TATTGCTGACTGTGTGGAGGCTTTGATAGGTGCTTATCTGATTGAATGTGGTCCGAGGGGAGCTTTGTTGTT
CATGTCTTGGCTTGGGATAAAAGTTTTACCTAGAGAGGAAATAAATATGACCACTGAATCCAGTTTTGTTCA
GCGTGTTGGAAGCATTAAAATAGATGACAATACAGTGGTGAACTTTACTAGACTTTCAGAACCGATTTCTC

CTTTGCTTCGACATGTAGAAGATCCAGAAGGGGAGTTAGCTCTTCTTATGGCTGGCTTTGAAGCTTTAGAAT
CGTCGCTGCATTATTCATTTAGAGATAAGTCATATCTACTACAAGCCATGAGCCATGCTTCTTATTCTCCAA
ATAGAGTTACAGATTGTTATCAGCGGCTTGAATTCTTGGGTGATGCAGTTCTAGATTACCTTATAACACGGC
ATCTTTTTGAGGATAAGCGTGCACATAGCCCTGGAGCATTAACAGATTTAAGGTCTGCATTAGTTAACAAC
ACTATATTTGCTTCTCTGGCTGTTAAATATGGACTCCATAAATACTTTAGGCATCTCTCACCAGGCCTTGCTG
AAGTTATTCAAAGATTTGTCACCATTCAAGAAGAAAATAGTCATCTCATTAGTGAAGAGTACTATTTAGTTG
AAGAAGAAGCTGAAGATGTAGAGGTACCAAAAGCGCTTGGTGATGTTTTTGAATCTGTTGCGGGAGCCATT
TTTCTTGATAGTGGAATGTCATTAGATACTGTTTGGAAGGTGTACTACAGAATGATGAAAAAAGAAATAGA
ACAATTTAGTACAAAAGTTCCAAAATCTCCCATCAGAGAACTGTTAGAACTGGAACCTGAAACAGCTAAAT
TCAGTAAACCTGAAAAATTAGCTGATGGAAGAAGAGTGAGAGTCACAGTTGAAGTATTTGGCAAAGGGAC
ATTCAAAGGTATAGGACGAAACTATAGAATAGCCAAATGCACTGCTGCCAAATGTGCTTTAAAACAATTGA
AGATTGGGCTATTAGCTAGAAAGTTAACTGAACACCCTAGTCAGTCATTGTTAAAAGTTCTGAGGGTTTGTC
ATGACCTGAAAAACAAGCATTAGAACTCCATTAATGTCTGTATTATATTTAAAATGACAACTATATTTGTGG
TTATTACTTAATTAATTAATTATTATGTAACATTAATTGTCATTAAAAAAAATTAGCTTTTAACTTGAG

Protein

RF: +1

ORF: 130 -> 2304

Length: 271 aa

>lcl|ORF1
MEIGTWSNDMAQLDDELQKDLPPELSMVRYGSPTSWNVGENQSFNYYSSDCESDDSSEIYLESETSDSSDGGAG
KLKITFRGDYLAEAIEENEPEPKKENSNSMKLDLELWTWDTEDLISNVKESIHEETEEFQKQTEQQMEIIKFQNKL
IKKEDQFVINKEDGKKQDTVPGEMKDFDFKVSSYSMPSDIIPKSSKKKSLSYHYPAETINNEELEEGITFSFDQQP
ELEGHPGPSPSILLQALTMSNANDGINLERLETIGDSFLKYAITTYLYCTHENIHEGKLSHLRSKQVSNLKLYRLG
QKKVFGESMIASKFEPHDNWLPPCYLVPRDAEQRLLDSEPQATLPYNLVTQHSIPDKSIADCVEALIGAYLIECGP
RGALLFMSWLGIKVLPREEINMTTESSFVQRVGSIKIDDNTVVNFTRLSEPISPLLRHVEDPEGELALLMAGFEAL
ESSLHYSFRDKSYLLQAMSHASYSPNRVTDCYQRLEFLGDAVLDYLITRHLFEDKRAHSPGALTDLRSALVNNTI
FASLAVKYGLHKYFRHLSPGLAEVIQRFVTIQEENSHLISEEYYLVEEEAEDVEVPKALGDVFESVAGAIFLDSG
MSLDTVWKVYYRMMKKEIEQFSTKVPKSPIRELLELEPETAKFSKPEKLADGRRVRVTVEVFGKGTFKGIGRNY
RIAKCTAAKCALKQLKIGLLARKLTEHPSQSLLKVLRVCHDLKNKH

Conserved Domains

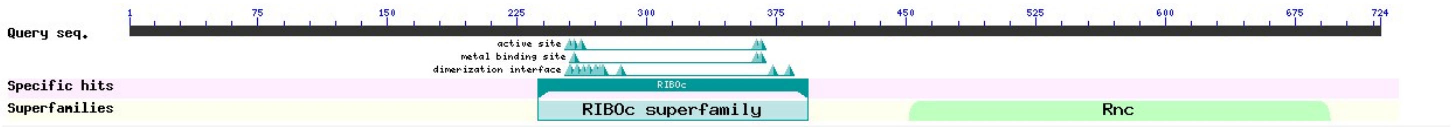

BLASTp

AVK59457.1 Dicer-1-PA *[Nezara viridula]*

Score:1321 bits

E-value: 0.0

```
Query  1347  MEIGTWSNDMAQLGDEFQKDLPPELSMVRYGSPTSWNVGENQPFNYYSSDCESDDSSEIY  1406
             MEIGTWSNDMAQL DE QKDLPPELSMVRYGSPTSWNVGENQ FNYYSSDCESDDSSEIY
Sbjct  1     MEIGTWSNDMAQLDDELQKDLPPELSMVRYGSPTSWNVGENQSFNYYSSDCESDDSSEIY  60

Query  1407  LESETSDSSDGGAGKLKITFRGDYLAEAIEDNEPEAKKENNNSMKLDLELWTWETDDLIT  1466
             LESETSDSSDGGAGKLKITFRGDYLAEAIE+NEPE KKEN+NSMKLDLELWTW+T+DLI+
Sbjct  61    LESETSDSSDGGAGKLKITFRGDYLAEAIEENEPEPKKENSNSMKLDLELWTWDTEDLIS  120

Query  1467  DVKEAILTETEEFQKQTEKQMEILKSQKKLIKKEEQFLVNKEDDKKQEIVSGEMKNFDFK  1526
             +VKE+I  ETEEFQKQTE+QMEI+K Q KLIKKE+QF++NKED KKQ+ V GEMK+FDFK
Sbjct  121   NVKESIHEETEEFQKQTEQQMEIIKFQNKLIKKEDQFVINKEDGKKQDTVPGEMKDFDFK  180

Query  1527  VRSYNMPSDIIPKSSKKKSLSYHYPAETINNQEIEEDIAFSFDHQPELEGHPGPSPSILL  1586
             V SY+MPSDIIPKSSKKKSLSYHYPAETINN+E+EE I FSFD QPELEGHPGPSPSILL
Sbjct  181   VSSYSMPSDIIPKSSKKKSLSYHYPAETINNEELEEGITFSFDQQPELEGHPGPSPSILL  240

Query  1587  QALTMSNANDGINLERLETIGDSFLKYAITTYLYCTHENIHEGKLSHLRSKQVSNLKLYR  1646
             QALTMSNANDGINLERLETIGDSFLKYAITTYLYCTHENIHEGKLSHLRSKQVSNLKLYR
Sbjct  241   QALTMSNANDGINLERLETIGDSFLKYAITTYLYCTHENIHEGKLSHLRSKQVSNLKLYR  300

Query  1647  LGQKKVFGESMIASKFEPHDNWLPPCYLVPRDAEQRLLDSEPQATLPYNLVTQHSIPDKS  1706
             LGQKKVFGESMIASKFEPHDNWLPPCYLVPRDAEQRLLDSEPQATLPYNLVTQHSIPDKS
Sbjct  301   LGQKKVFGESMIASKFEPHDNWLPPCYLVPRDAEQRLLDSEPQATLPYNLVTQHSIPDKS  360

Query  1707  IADCVEALIGAYLIECGPRGALLFMSWLGIKVLPKEEIDTTTDPSLVQRVGSIKTDENIV  1766
             IADCVEALIGAYLIECGPRGALLFMSWLGIKVLP+EEI+ TT+ S VQRVGSIK D+N V
Sbjct  361   IADCVEALIGAYLIECGPRGALLFMSWLGIKVLPREEINMTTESSFVQRVGSIKIDDNTV  420

Query  1767  VNFTSLSEPISPLLRHVDDPEGELAFLLAGFEALESSLHYSFRDKSYLLQAMSHASYSPN  1826
             VNFT LSEPISPLLRHV+DPEGELA L+AGFEALESSLHYSFRDKSYLLQAMSHASYSPN
Sbjct  421   VNFTRLSEPISPLLRHVEDPEGELALLMAGFEALESSLHYSFRDKSYLLQAMSHASYSPN  480

Query  1827  RVTDCYQRLEFLGDAVLDYLITRHLFEDKRAHSPGALTDLRSALVNNTIFASLAVKYGLH  1886
             RVTDCYQRLEFLGDAVLDYLITRHLFEDKRAHSPGALTDLRSALVNNTIFASLAVKYGLH
Sbjct  481   RVTDCYQRLEFLGDAVLDYLITRHLFEDKRAHSPGALTDLRSALVNNTIFASLAVKYGLH  540

Query  1887  KYFRHLSPGLAEVIQRFVTIQEENSHLISEEYYLVGEEAEDVEVPKALGDVFESVAGAIF  1946
             KYFRHLSPGLAEVIQRFVTIQEENSHLISEEYYLV EEAEDVEVPKALGDVFESVAGAIF
```

Sbjct 541 KYFRHLSPGLAEVIQRFVTIQEENSHLISEEYYLVEEEAEDVEVPKALGDVFESVAGAIF 600

Query 1947 LDSGMSLDTVWKVYYRMMKKEIEQFSTKVPKSPIRELLELEPETAKFSKPEKLADGRRVR 2006

LDSGMSLDTVWKVYYRMMKKEIEQFSTKVPKSPIRELLELEPETAKFSKPEKLADGRRVR

Sbjct 601 LDSGMSLDTVWKVYYRMMKKEIEQFSTKVPKSPIRELLELEPETAKFSKPEKLADGRRVR 660

Query 2007 VTVEVFGKGTFKGIGRNYRIAKCTAAKCALKQLKIGLLARKLTEHPSQSLLKVLRVCHDL 2066

VTVEVFGKGTFKGIGRNYRIAKCTAAKCALKQLKIGLLARKLTEHPSQSLLKVLRVCHDL

Sbjct 661 VTVEVFGKGTFKGIGRNYRIAKCTAAKCALKQLKIGLLARKLTEHPSQSLLKVLRVCHDL 720

Query 2067 KNKH 2070

KNKH

Sbjct 721 KNKH 724

Argonaute 1 (Ago-1)

>TRINITY_DN2687_c0_g1_i1 len=2906 path=[0:0-554 2:555-709 3:710-2905]

CGAAAACCTTTGACAGACTCTCAGAGGGTTAAATTTACTAAAGAAATAAAAGGATTAAAAATTGAAATTAC
ACATTGTGGAACAATGAGAAGAAAGTATAGAGTCTGCAATGTTACAAGGAGACCTGCCCAAATGCAGTCG
TTTCCATTACAGTTGGAAAATGGACAAACTGTGGAGTGCACAGTTGCTAAGTATTTCTTAGACAAATATAA
AATGAAATTAAGATACCCACATTTACCCTGTCTTCAAGTCGGGCAGGAACATAAGCATACATATCTTCCTCT
GGAGGTTTGTAATATAGTTGCTGGGCAGAGATGCATTAAGAAACTAACTGACATGCAAACTTCAACGATGA
TCAAAGCTACCGCTAGGTCTGCACCTGATCGAGAAAGAGAAATCAACAATCTCGTCAAGAGAGCAGATTTC
AATAATGATGCCTATGTTCAAGAATTTGGATTAGCTATATCCAATAATATGATGGAAGTTCGAGGTAGAGT
TCTACCACCACCAAAGCTGCAGTATGGCGGGCGTGCACCAAACTTGCCCAGTCAGCTCGAATACCAAGGCA
TGTTGTCTGCAAAACAACAAGCACTTCCTAACCAGGGCGTATGGGATATGCGAGGCAAACAGTTCTTTACT
GGTGTTGAAATTAGGGTCTGGGCGATCGCTTGTTTTGCACCTCAAAGGACTGTTAGAGAAGATGCCCTCAG
AAACTTTACTCAACAATTGCAAAAAATTAGTAATGATGCTGGAATGCCAATAATAGGCCAACCTTGTTTTTG
TAAATACGCTACTGGTCCAGATCAAGTAGAGCCTATGTTTCGTTACCTGAAATCTTCCTTCCAAGCATTACA
GCTTGTTGTGGTTGTGCTTCCGGGGAAAACTCCAGTTTATGCTGAAGTGAAGAGGGTTGGAGATACAGTTTT
AGGTATGGCCACACAATGCGTTCAAGCTAAAAATGTAAATAAAACTTCACCGCAGACTCTCTCCAATCTTT
GTCTCAAAATTAACGTTAAACTTGGCGGCATTAATAGCATATTAGTTCCCAGCATCAGGCCGAAGGTTTTCA
ATGAACCAGTGATATTCCTCGGTGCTGATGTGACTCACCCGCCTGCTGGAGATAATAAAAAACCTTCTATA
GCAGCTGTTGTTGGTTCCATGGATGCACATCCAAGTCGATATGCTGCTACTGTTAGGGTTCAGCAGCATCGT
CAAGAAATAATTCAAGAACTATCCTCTATGGTCAGAGAACTTCTCATAATGTTTTACAAAAGTACTGGCGG
CTACAAGCCTCACAGAATTATACTCTACCGAGATGGTGTGTCAGAGGGTCAATTTTTACATGTTTTGCAACA
CGAACTTACTGCTATAAGAGAAGCTTGTATTAAGCTAGAAGGAGATTATAAGCCTGGAATTACTTTTATTGT
GGTGCAAAAGAGGCATCATACAAGGTTATTCTGTGCTGACAAGAAAGAGCAAAGTGGTAAGTCTGGAAAT
ATACCTGCTGGTACGACTGTAGATGTGGGAATAACCCATCCTACTGAATTTGACTTCTACCTTTGCAGCCAT
CAAGGTATTCAGGGTACTAGCCGGCCAAGTCATTATCATGTATTATGGGATGACAATCATTTTGATTCTGAT
GAACTGCAATGCTTAACTTATCAGTTGTGCCATACGTATGTGAGGTGTACACGCTCAGTATCTATACCTGCT
CCTGCATATTATGCCCACCTAGTTGCATTTAGAGCGAGGTACCATCTTGTCGAGAAAGAGCATGACAGCGG
TGAGGGTTCTCACCAGTCAGGCTGCAGTGAAGACCGAACACCTGGTGCAATGGCACGAGCAATTACCGTTC
ATGCAGATACAAAGAAAGTAATGTACTTTGCATAACCTAAGTTTCATGAGAACTTCCTGCCATGTGGGATT
ATAGGAACCAGCCAGCTATTATTCAATGGTCTCTTTAATTGTTTACCCTCTTTTGAAGGGGTTTATCGCTGT
GTTGGTCGTCCTTTTTAATGCGATTTCTAATATTAAGATAATTATTTATGATTAAGGAACCTACAAAAAAA

AAGCAGTGATAGTTATATTTTCATAAGAGTCAAATGAAAACACTGATTCATATTTGTTAAGTCAAATTTCAC
TAGTTGGAAGAAAACTAATGGAGGAAAGCTGCCGTATTAATATAAGTCTTCTTTAGCTGGTGCACAAGTCT
AGGCATGTAAATAGGTTTAGGGCTGTCAAGAGAGATACATGAAGAAACCTATATAACTTAGTAGATAAGTT
GTAACTTGTTCCATTATGCTTGTCTTTTTCTTTTCTTTCCTTTTTTTATATATTTATTTTATATTTCTCTCTTTAT
TTCTCTCTTAAGCCAAGGTCTCCAAGTCAACTATTTGTTGATAGCTTTAATCTTTTTATTCTATGAGGATGAA
TATACCAAATGGTTGTGTAGTCATGTCTCCAGGCGATGTACTAAAGTCAGCTTTAAGTTAGAACAGGTCTGT
GGTGATTTAGTGTCCTCACTTAGTCGTACTTAAAAATTTTAATCTGCTGAATGAAATAATGCAAAAGAATTG
TTAATTTTAATTTCCTTCATATTAATGTTATTTTTATTTACAAGATAAATTTCACCTGTACTTGTTATTTGTTA
TATTTTAACATTTGATGAATTCTTATTTCTTTCTTTTTTATTTTTTACATGATTATTTATTATTATGTACTCAA
TATTCACAATTTTAAGAGATGCAGGTATTATTTTCTTAATAAGCTACGATTCTGTTTTAAAAATTGTATTTGC
TTATATTATTTAGAAAGCTGTCTTTTTTTTAATTATTTAATTTTATATATATATTTTTTTCTAAGGTATTTTTAT
GATCTTAAATCTATATACTTCGTGTCATTTG

Protein

RF: +1

ORF: 85 -> 1890

Length: 601 aa

>lcl|ORF1_TRINITY_DN2687_c0_g1_i1:84:1889 unnamed protein product

MRRKYRVCNVTRRPAQMQSFPLQLENGQTVECTVAKYFLDKYKMKLRYPHLPCLQVGQEHKHTYLPLEVCNI
VAGQRCIKKLTDMQTSTMIKATARSAPDREREINNLVKRADFNNDAYVQEFGLAISNNMMEVRGRVLPPPKLQ
YGGRAPNLPSQLEYQGMLSAKQQALPNQGVWDMRGKQFFTGVEIRVWAIACFAPQRTVREDALRNFTQQLQK
ISNDAGMPIIGQPCFCKYATGPDQVEPMFRYLKSSFQALQLVVVVLPGKTPVYAEVKRVGDTVLGMATQCVQA
KNVNKTSPQTLSNLCLKINVKLGGINSILVPSIRPKVFNEPVIFLGADVTHPPAGDNKKPSIAAVVGSMDAHPSRY
AATVRVQQHRQEIIQELSSMVRELLIMFYKSTGGYKPHRIILYRDGVSEGQFLHVLQHELTAIREACIKLEGDYKP
GITFIVVQKRHHTRLFCADKKEQSGKSGNIPAGTTVDVGITHPTEFDFYLCSHQGIQGTSRPSHYHVLWDDNHFD
SDELQCLTYQLCHTYVRCTRSVSIPAPAYYAHLVAFRARYHLVEKEHDSGEGSHQSGCSEDRTPGAMARAITVH
ADTKKVMYFA

Conserved Domains

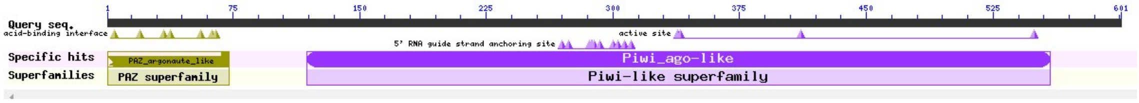

BLASTp

AVK59466.1 Ago-1 *[Nezara viridula]*

Score: 1257 bits

E-value: 0.0

Query 321 MRRKYRVCNVTRRPAQMQSFPLQLENGQTVECTVAKYFLDKYKMKLRYPHLPCLQVGQEH 380
MRRKYRVCNVTRRPAQMQSFPLQLENGQTVECTVAKYFLDKYKMKLRYPHLPCLQVGQEH
Sbjct 1 MRRKYRVCNVTRRPAQMQSFPLQLENGQTVECTVAKYFLDKYKMKLRYPHLPCLQVGQEH 60

```
Query  381  KHTYLPLEVCNIVAGQRCIKKLTDMQTSTMIKATARSAPDREREINNLVRRADFNNDAYV  440
            KHTYLPLEVCNIVAGQRCIKKLTDMQTSTMIKATARSAPDREREINNLV+RADFNNDAYV
Sbjct  61   KHTYLPLEVCNIVAGQRCIKKLTDMQTSTMIKATARSAPDREREINNLVKRADFNNDAYV  120

Query  441  QEFGLAISNNMMEVRGRVLPPPKLQYGGRAPNLPSQLEYQGMLSAKQQALPNQGVWDMRG  500
            QEFGLAISNNMMEVRGRVLPPPKLQYGGRAPNLPSQLEYQGMLSAKQQALPNQGVWDMRG
Sbjct  121  QEFGLAISNNMMEVRGRVLPPPKLQYGGRAPNLPSQLEYQGMLSAKQQALPNQGVWDMRG  180

Query  501  KQFFTGVEIRVWAIACFAPQRTVREDALRNFTQQLQKISNDAGMPIIGQPCFCKYATGPD  560
            KQFFTGVEIRVWAIACFAPQRTVREDALRNFTQQLQKISNDAGMPIIGQPCFCKYATGPD
Sbjct  181  KQFFTGVEIRVWAIACFAPQRTVREDALRNFTQQLQKISNDAGMPIIGQPCFCKYATGPD  240

Query  561  QVEPMFRYLKSSFQALQLVVVVLPGKTPVYAEVKRVGDTVLGMATQCVQAKNVNKTSPQT  620
            QVEPMFRYLKSSFQALQLVVVVLPGKTPVYAEVKRVGDTVLGMATQCVQAKNVNKTSPQT
Sbjct  241  QVEPMFRYLKSSFQALQLVVVVLPGKTPVYAEVKRVGDTVLGMATQCVQAKNVNKTSPQT  300

Query  621  LSNLCLKINVKLGGINSILVPSIRPKVFNEPVIFLGADVTHPPAGDNKKPSIAAVVGSMD  680
            LSNLCLKINVKLGGINSILVPSIRPKVFNEPVIFLGADVTHPPAGDNKKPSIAAVVGSMD
Sbjct  301  LSNLCLKINVKLGGINSILVPSIRPKVFNEPVIFLGADVTHPPAGDNKKPSIAAVVGSMD  360

Query  681  AHPSRYAATVRVQQHRQEIIQELSSMVRELLIMFYKSTGGYKPHRIILYRDGVSEGQFLH  740
            AHPSRYAATVRVQQHRQEIIQELSSMVRELLIMFYKSTGGYKPHRIILYRDGVSEGQFLH
Sbjct  361  AHPSRYAATVRVQQHRQEIIQELSSMVRELLIMFYKSTGGYKPHRIILYRDGVSEGQFLH  420

Query  741  VLQHELTAIREACIKLEGDYKPGITFIVVQKRHHTRLFCADKKEQSGKSGNIPAGTTVDV  800
            VLQHELTAIREACIKLEGDYKPGITFIVVQKRHHTRLFCADKKEQSGKSGNIPAGTTVDV
Sbjct  421  VLQHELTAIREACIKLEGDYKPGITFIVVQKRHHTRLFCADKKEQSGKSGNIPAGTTVDV  480

Query  801  GITHPTEFDFYLCSHQGIQGTSRPSHYHVLWDDNHFDSDELQCLTYQLCHTYVRCTRSVS  860
            GITHPTEFDFYLCSHQGIQGTSRPSHYHVLWDDNHFDSDELQCLTYQLCHTYVRCTRSVS
Sbjct  481  GITHPTEFDFYLCSHQGIQGTSRPSHYHVLWDDNHFDSDELQCLTYQLCHTYVRCTRSVS  540

Query  861  IPAPAYYAHLVAFRARYHLVEKEHDSGEGSHQSGCSEDRTPGAMARAITVHADTKKVMYF  920
            IPAPAYYAHLVAFRARYHLVEKEHDSGEGSHQSGCSEDRTPGAMARAITVHADTKKVMYF
Sbjct  541  IPAPAYYAHLVAFRARYHLVEKEHDSGEGSHQSGCSEDRTPGAMARAITVHADTKKVMYF  600
```

Query 921 A 921

A

Sbjct 601 A 601

Loquacious

>TRINITY_DN19426_c0_g1_i2 len=2159 path=[0:0-201 2:202-953 3:954-1100 4:1101-2158]

CTTGGATGGCGCTTCCTTGTGGCCGAAAATACAGTTGATTATCATACCCATCCTCCCCTCCCTGGT
AGTGGTAGTGGCAGAAATCAATTTCATCTGTGCCGTACGTTCGTCGCCATTTTCTCGAGATAAGT
TAAAGAAACCGGCAAGATTACAAGGACAACAGCTGGACTATCAAATATTGTAGAGGATTGATGG
CAGAAGGGAATTTAAAAATTGAAGAAAGTTTATTGGGACATGTGGGTTTGTCAAGGCATGCCCC
TTCAATTCGTGGTGGACGTCCTAGGATGAGAAGTAATACTCATAATACCAGAATACAACCTCCAG
ATCCCATTCCTCTTGAAGAGGCTGCAAAAAGGGAGCTGCAGGCCATGCCTACCAAAACACCTGT
GTCCATGCTGCAAGAGTTGCTTTCCAGGAGAGGGACAACTCCAAAATATGAGCTGGTTCAGGTG
GAGGGAGCAATACATGAACCCACTTTTAGGTATACTGTCTCCGTAGGAGAAACTATAGGATCCA
CTAGATTTGCTGCTATGGGTACTGGCAGGTCTAAGAAAGAAGCGAAGCATGCTGCTGCGAAGGC
AATTTTAGATAAGATCACAGGAAATACAGAACCTCTAGATATCTCTAGTCCAACCTCTAATACGG
ATAGCCTAGGCCCCAATATTGGGGCAGTCGAGGAGCGAATGATGGGCAACCCTATCGGTCTTCT
GCAAGAGTTGTGCATGACAAGGCGATGGCCTCCACCTGCCTACGAAACCGAAAATGAAGAGGGC
CTTCCGCATGAAAGACAGTTCACCATTTCCTGCCTCGTATTCCGACACAAAGAAATAGGTACTGG
TAAATCGAAAAAAATTGCCAAGAGATTAGCAGCACATAAAATGTGGATGAGACTGAAAGACTTA
CCCTGCGAAAGCAACACTATTCATTATGGCTTGGATGATGAAGATGAAATAAATCAGAGAGTTC
CTCAAGGTGAGTTTGACTCATTGAAGATGGTCAAAATCCCTACCCTTGCCACGGCGCCTTACAAC
ATCAAAGTGTCACAATTCCACAAGAACCTCAAATCATCGGTCGGGCCGAAGCTTGATGAGCTCC
AGATAATTTCTTTAAAGGATTCAGATTTTAATGCAGTCCAGTTCCTGCAGGAGATAGCCACTGAG
CAACAGTTTGAAGTGACATTCGTTGACGTTGAAGAAAAATCAGTCTCAGGAAAATGCCAGAGCT
TGGTGCAGCTGTCGACACTGCCCGTAGCAGTTTGTTACGGCACAGGAATGTCCTCTTCAGAGGCA
CAAACTTGTGCTGCCTACAATGCACTACAGTATCTCAAACTAATGACCAAGAAATAGAATCGGG
TGACTCAAGACCGGCTCAGAACTGCAACCGTGTACCTCACTTCCTTTACTTGCTTTGTTATCATGA
CCTTCTGACAAGGTAGCCTCCGCACCAGGCATCAGATGGTGTTGCAGCTCCTCTTTCTGTTAATTT
ATTTTAAATATTAACTTTTAATTGTTTTTAAAGAATTGCCATTTGTTGGTTTTGAAAACCCGAAAA
TGGGAAACGTTTTTACAAGTAGAGCCAGATGATATATGTTTTTATTCGATAATCAGAGTGAGTTA
TGGCCCATTGTTAGTTGTAACCCTATTTTACTTTTAATATAAAATAAAACAGTTTTTTTCTGGTTG
AACATTATTATAAGAGCTTCTGGAAACAGAAAGAAAAGTTAAATTATTTTTAATTTTAAATATTT
TATTAATTACTGTTTAACATAAAAGAAAAATTATGTTGTTATATTTGTTATTTTTTGCTTCTGTCTT
TCAGTTTTAAAACTTTTTATTTTGTTTATTATGTGTGTTTTTATTTTTGAAATAGGTATAGAATTAA
TACCTATCTACTTTCATGAACTGCATTGTTTTTCAGAAGCTCTACAAACAATTATTATTAATGTAG
GATAGCAGCTACAATGTGCCAAGCGTAATAGAAATAGAGTGACAATTATTTTTATTTTAATTCAA
ATCGACACAATGTATCCTAGGTTCTTTTAAAGTATTTTGAGCTTATTTTGTTTTATGTTGACCTTGC
TTTAAGAATTTGACACTTATTTAGTTGAAGTCTTTTGTTTGATAATATTAACAATAAAGTGTTCAA
TTTGAAAAAAAAAA

Protein

RF: +3

ORF: 192 -> 1346

Length: 384 aa

>lcl|ORF2_TRINITY_DN19426_c0_g1_i2:191:1345 unnamed protein product

MAEGNLKIEESLLGHVGLSRHAPSIRGGRPRMRSNTHNTRIQPPDPIPLEEAAKRELQAMPTK
TPVSMLQELLSRRGTTPKYELVQVEGAIHEPTFRYTVSVGETIGSTRFAAMGTGRSKKEAKH
AAAKAILDKITGNTEPLDISSPTSNTDSLGPNIGAVEERMMGNPIGLLQELCMTRRWPPPAYE
TENEEGLPHERQFTISCLVFRHKEIGTGKSKKIAKRLAAHKMWMRLKDLPCESNTIHYGLDD
EDEINQRVPQGEFDSLKMVKIPTLATAPYNIKVSQFHKNLKSSVGPKLDELQIISLKDSDFNA
VQFLQEIATEQQFEVTFVDVEEKSVSGKCQSLVQLSTLPVAVCYGTGMSSSEAQTCAAYNA
LQYLKLMTKK

Conserved Domains

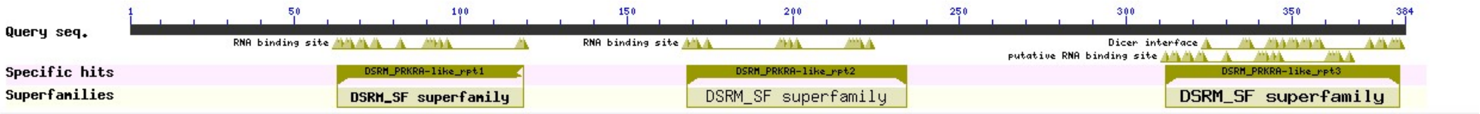

BLASTp

XP_01442734312.1 RISC-loading complex subunit tarbp2-like isoform X1 [*Halyomorpha halys*]

Score:739 bits

E-value: 0.0

Query 1 MAEVNIKIEENLLGHVGLSRHPPSIRGGRPRMRSNTQSTKVQPPEPIPLEEAAKRELQAM 60
MAE N+KIEE+LLGHVGLSRH PSIRGGRPRMRSNT +T++QPP+PIPLEEAAKRELQAM
Sbjct 1 MAEGNLKIEESLLGHVGLSRHAPSIRGGRPRMRSNTHNTRIQPPDPIPLEEAAKRELQAM 60

Query 61 PTKTPVSMLQELLSRRGTTPKYELVQVEGAIHEPTFRYTVSVGETIGSSRFAAMGTGRSK 120
PTKTPVSMLQELLSRRGTTPKYELVQVEGAIHEPTFRYTVSVGETIGS+RFAAMGTGRSK
Sbjct 61 PTKTPVSMLQELLSRRGTTPKYELVQVEGAIHEPTFRYTVSVGETIGSTRFAAMGTGRSK 120

Query 121 KEAKHAAAKAILDKITGNTESLDISSPTSSNTESLGPNIGTVEERMMGNPIGLLQELCMT 180
KEAKHAAAKAILDKITGNTE LDISSPTS NT+SLGPNIG VEERMMGNPIGLLQELCMT
Sbjct 121 KEAKHAAAKAILDKITGNTEPLDISSPTS-NTDSLGPNIGAVEERMMGNPIGLLQELCMT 179

Query 181 RRWPPPAYETENEEGLPHERQFTISCLVFRHKEIGTGKSKKIAKRLAAHKMWMRLKDLPC 240
RRWPPPAYETENEEGLPHERQFTISCLVFRHKEIGTGKSKKIAKRLAAHKMWMRLKDLPC
Sbjct 180 RRWPPPAYETENEEGLPHERQFTISCLVFRHKEIGTGKSKKIAKRLAAHKMWMRLKDLPC 239

Query 241 ESTTIHYGLDDEDEINQRVPQSEFDSLKMVKIPTLATAPYNIKVSQFHKNLKSSVGPKLD 300
ES TIHYGLDDEDEINQRVPQ EFDSLKMVKIPTLATAPYNIKVSQFHKNLKSSVGPKLD
Sbjct 240 ESNTIHYGLDDEDEINQRVPQGEFDSLKMVKIPTLATAPYNIKVSQFHKNLKSSVGPKLD 299

Query 301 ELQIISLKDSDFNAVQFLQEIAAEQQFEVTFVDVEEKSVTGKCQSLVQLSTLPVAVCYGT 360
ELQIISLKDSDFNAVQFLQEIA EQQFEVTFVDVEEKSV+GKCQSLVQLSTLPVAVCYGT
Sbjct 300 ELQIISLKDSDFNAVQFLQEIATEQQFEVTFVDVEEKSVSGKCQSLVQLSTLPVAVCYGT 359

Query 361 GMSSSEAQTCAAYNALQYLKLMTKK 385
GMSSSEAQTCAAYNALQYLKLMTKK
Sbjct 360 GMSSSEAQTCAAYNALQYLKLMTKK 384

Drosha

>TRINITY_DN28734_c0_g1_i1 len=2761 path=[0:0-2760]

CTTTTAATACCAAATGATGAACTCAAAGAAATTATAAGTTTGCCTAATTACAAGTGGCAAAAACTTGTGGA
CGAGGTTAAAGGAATGGTGGTTACATATCCTGGAAAAAAACCTTGTTCTATCAGGGTGGACCAACTTGACA
AAGATCAAAGTAATGCCAGTGATGGGAATCCTAAATACCCTGAAATAGTTCATTTGGAATTCGTCCACCT
CAATTGAGCTATGCTGGAAATCCAGAATATCAGAAAGCATGGAGGGAATATGTAAAGTTTCGTCATCTCCT
TGCTAATATGCCAAAATCGTCCTATGAAGACAAAAGAAAATTAGAGGCAAAAGAAAACAAACTTCAAGAA
ATGAGAAGTCAAAGTAAAATGAAACGAGATGTTACCATTGCTGTTTCCAGTGAAGGATTTTACAGAACAGG
TATTATGTGTGATATTGTCCAACATGCTATGCTTCTACCAGTTTTAGTATCCCATTTAAGATTTCATCGGTCT
TTAGATGTACTAGAAGGCAAAATAAATTATAAATTTAAAAATAGAAGCTTATTACAACTTGCTTTAACTCAT
CCATCTTATAGAGAAAATTTTGGGACCAATCCAGATCATGCTCGAAACTCTCTTACAAACTGTGGCATTCGC
CAGCCAGAATATGGAGACAGGCGAATTCATTACATGAATACAAGAAAGAGAGGTATAAACACACTTATTA
ATATAATGTCCAGGTTTGGTAAGAAGCGAGAAACTGAATCGAATATTACTCACAATGAACGTTTAGAATTC
CTTGGAGATGCTGTAGTGGAATTTGTTTCATCAATTCATTTATTTCACCTTTTTCCTAGGCTTGAAGAAGGAG
GTCTTGCTACTTATAGGGCAGCTATCGTTCAAAATCAACATCTAGCAGTTTTAGCAAAAACATTAGGCTTAG
ATGGATATATGTTGTATGCCCATGGTTCAGACTTATGCCATGATCTTGAGTTGAGACATGCAATGGCCAACT
GTTTTGAGGCTTTAATGGGTGCACTTTTTCTGGATGGTGGCATAGAAGCAGCTGATAAAGTTTTTGGTGAAG
TACTTTTTGAAGACAATAAAGTTTTACAGAATGTTTGGAGGAATTATCCACCACACCCTTTACAAGAAGAA
GAACCAGCTGGTGATAGAAAGTGGATTGAATCTTACCAGATGCTACAAAATCTTACAAAATTTGAAGATTC
TATTGGTGTTAAGTTTAATCATATTCGTCTTTTAGCTCGTGCTTTTACTGACAGAAGTATGGGTTATAATAAC
TTAACACTTGGGTCAAATCAGAGATTAGAATTTCTCGGTGATACTGTTCTTCAGTTGATTGCATCAGAATAT
TTATATAAATATTTTCCAGAACATCATGAAGGTCATCTTTCATTGCTAAGAAGTTCTTTAGTAAACAATAGA
ACACAAGCAGTGGTATGTGATGATCTTGGTATGGCTGCTTATGCAGTATATTCTAATCCTAAAGCTGAACTG
AAGACTAAAGATCGAGCTGATCTTTTAGAAGCTTTTCTTGGCGCTCTTTATGTTGATAAAGGTTTACTTTATT
GTCAAACCTTCTGCAATGTTTGTTTCTTTCCGAGACTTCATGACTTCATTATGCATCAAGATTGGAATGACC
CAAAATCCAAGTTACAACAATGTTGTCTTACATTGAGAACAATGGAAGGAGGTGAACCAGAAATACCAGT
GTATAAAGTCATTCAGTGTATGGGGCCAACAAACACTCGTTTATACACTGTTGCCGTTTACTTTAAAGGAAA
GCGTTTAGCTGAAGCTTCTGGTCATAGCATTCAGCAAGCTGAAATGAATGCAGCGAAAGTAGCTTTAGAGC
ACTCACAAAGTTTGTTTCCTCAACTAGATCATCAAAAACGAGTAATTGCTAAAAGTATGAAACAAGAACCT

CTGAACTCGGCAGCATCGAAGAAGAAACAAAACAAAGATCATTACGATGATAAAAAATATAGTAAATATG
ATTATAGCAATTTACATAAAAAAATTAAAAGGGAAAATATTAAACTATCTTCATCCAAAGAATCATCACCC
ATTTCCAGGAGATTTTCTTCTAGCAATGATAGGTCAGATTCTGATGAAGAGAATCTTGGAAAAATTAAAAA
ATACAAACACAAGAAATATCATGAATATTCTTCAAGTAGAATGTCGAGTTCTGATGAGTATTGTTCAGATC
GAGAATTCAACAGGAAAAAAAATTCGTTATTATAGTAGTTCTTCCACTAGTTCTTCTGAAGATGAAAGCAGT
ATTGGACAAAGTCCTAATAGCTCAAAAAAATCAATGCAGAAGTATGAACCCGATGAAGAACTATCAAAAA
AATATTTGTTTTACACAAATGAACTGGAATGTATGAAAAATGAGTTAGATGAAGAAGGAAAAAGTAATGTA
TTAAAAGAAATTGATATAAAAACAGAAGTGGATACAGTATCTGAAGACGAAAACAGTTTATCATCATTCGA
TGACCAGAATAAGCGTGTGAAGTTGGAAACTTCATAATTTTATTATTCATTTTAGTTGCATAAATGTATTGT
ACATTATTTATGATAATTTTTTTATATTACAATTGAATTATTTGTTGTTTTTACTGTGTAAAATATTTATAAAT
TTATTTTATTTTATTTTAACACTAGATATTCTGTCAACATGGCTGTGTTTATGATAAAAATATGTATGGAAAT
ATCTTAGTATTGTAAAATATGACTGGTAATTTTTTAATCTAACA

Protein

RF: +1

ORF: 85 -> 2451

Length: 816 aa

>lcl|ORF1_TRINITY_DN28734_c0_g1_i1:84:2534 unnamed protein product

MVVTYPGKKPCSIRVDQLDKDQSNASDGNPKYPEIVHFGIRPPQLSYAGNPEYQKAWREYVKFRHLLANMPKS
SYEDKRKLEAKENKLQEMRSQSKMKRDVTIAVSSEGFYRTGIMCDIVQHAMLLPVLVSHLRFHRSLDVLEGKIN
YKFKNRSLLQLALTHPSYRENFGTNPDHARNSLTNCGIRQPEYGDRRIHYMNTRKRGINTLINIMSRFGKKRETE
SNITHNERLEFLGDAVVEFVSSIHLFHLFPRLEEGGLATYRAAIVQNQHLAVLAKTLGLDGYMLYAHGSDLCHD
LELRHAMANCFEALMGALFLDGGIEAADKVFGEVLFEDNKVLQNVWRNYPPHPLQEEEPAGDRKWIESYQML
QNLTKFEDSIGVKFNHIRLLARAFTDRSMGYNNLTLGSNQRLEFLGDTVLQLIASEYLYKYFPEHHEGHLSLLRS
SLVNNRTQAVVCDDLGMAAYAVYSNPKAELKTKDRADLLEAFLGALYVDKGLLYCQTFCNVCFFPRLHDFIM
HQDWNDPKSKLQQCCLTLRTMEGGEPEIPVYKVIQCMGPTNTRLYTVAVYFKGKRLAEASGHSIQQAEMNAA
KVALEHSQSLFPQLDHQKRVIAKSMKQEPLNSAASKKKQNKDHYDDKKYSKYDYSNLHKKIKRENIKLSSSKE
SSPISRRFSSSNDRSDSDEENLGKIKKYKHKKYHEYSSSRMSSSDEYCSDREFNRKKIRYYSSSSTSSSEDESSIGQS
PNSSKKSMQKYEPDEELSKKYLFYTNELECMKNELDEEGKSNVLKEIDIKTEVDTVSEDENSLSSFDDQNKRVK
LETS

Conserved Domains

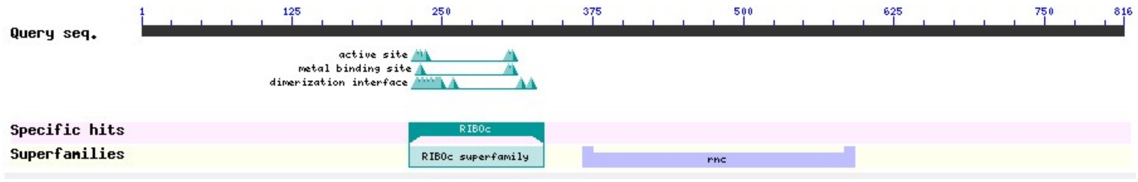

BLASTp

XP_014278529.1 Dicer-1-PA ribonuclease 3 [*Halyomorpha halys*]

Score:1442 bits

E-value: 0.0

```
Query  524   MVVTYPGKKPCSIRVDQLDKDQSNASDGNPKYPEIVHFGIRPPQLSYAGNPEYQKAWREY  583
             MVVTYPGKKPCSIRVDQLDKDQSNASDGNPKYPEIVHFGIRPPQLSYAGNPEYQKAWREY
Sbjct  1     MVVTYPGKKPCSIRVDQLDKDQSNASDGNPKYPEIVHFGIRPPQLSYAGNPEYQKAWREY  60

Query  584   VKFRHLLANMPKSSYEDKRKLEAKENKLQEMRSQSKMKRDVTIAVSSEGFYRTGIMCDIV  643
             VKFRHLLANMPKSSYEDKRKLEAKENKLQEMRSQSKMKRDVTIAVSSEGFYRTGIMCDIV
Sbjct  61    VKFRHLLANMPKSSYEDKRKLEAKENKLQEMRSQSKMKRDVTIAVSSEGFYRTGIMCDIV  120

Query  644   QHAMLLPVLVSHLRFHRSLDVLENKINYKFKNRSLLQLALTHPSYRENFGTNPDHARNSL  703
             QHAMLLPVLVSHLRFHRSLDVLE KINYKFKNRSLLQLALTHPSYRENFGTNPDHARNSL
Sbjct  121   QHAMLLPVLVSHLRFHRSLDVLEGKINYKFKNRSLLQLALTHPSYRENFGTNPDHARNSL  180

Query  704   TNCGIRQPEYGDRRIHYMNTRKRGINTLINIMSRFGKKRETESNITHNERLEFLGDAVVE  763
             TNCGIRQPEYGDRRIHYMNTRKRGINTLINIMSRFGKKRETESNITHNERLEFLGDAVVE
Sbjct  181   TNCGIRQPEYGDRRIHYMNTRKRGINTLINIMSRFGKKRETESNITHNERLEFLGDAVVE  240

Query  764   FVSSIHLFHLFPRLEEGGLATYRAAIVQNQHLAVLAKTLGLDGYMLYAHGSDLCHDLELR  823
             FVSSIHLFHLFPRLEEGGLATYRAAIVQNQHLAVLAKTLGLDGYMLYAHGSDLCHDLELR
Sbjct  241   FVSSIHLFHLFPRLEEGGLATYRAAIVQNQHLAVLAKTLGLDGYMLYAHGSDLCHDLELR  300

Query  824   HAMANCFEALMGALFLDGGIEAADKVFGEVLFEDNEILQNVWRNYPPHPLQEEEPAGDRK  883
             HAMANCFEALMGALFLDGGIEAADKVFGEVLFEDN++LQNVWRNYPPHPLQEEEPAGDRK
Sbjct  301   HAMANCFEALMGALFLDGGIEAADKVFGEVLFEDNKVLQNVWRNYPPHPLQEEEPAGDRK  360

Query  884   WIESYQMLQNLTKFEDSIGVKFNHIRLLARAFTDRSMGYNNLTLGSNQRLEFLGDTVLQL  943
             WIESYQMLQNLTKFEDSIGVKFNHIRLLARAFTDRSMGYNNLTLGSNQRLEFLGDTVLQL
Sbjct  361   WIESYQMLQNLTKFEDSIGVKFNHIRLLARAFTDRSMGYNNLTLGSNQRLEFLGDTVLQL  420
```

```
Query  944   IASEYLYKYFPEHHEGHLSLLRSSLVNNRTQAVVCDDLGMAAYAVYSNPKAELKTKDRAD  1003
             IASEYLYKYFPEHHEGHLSLLRSSLVNNRTQAVVCDDLGMAAYAVYSNPKAELKTKDRAD
Sbjct  421   IASEYLYKYFPEHHEGHLSLLRSSLVNNRTQAVVCDDLGMAAYAVYSNPKAELKTKDRAD  480

Query  1004  LLEAFLGALYVDKGLLYCQTFCNVCFFPRLHDFIMHQDWNDPKSKLQQCCLTLRTMEGGE  1063
             LLEAFLGALYVDKGLLYCQTFCNVCFFPRLHDFIMHQDWNDPKSKLQQCCLTLRTMEGGE
Sbjct  481   LLEAFLGALYVDKGLLYCQTFCNVCFFPRLHDFIMHQDWNDPKSKLQQCCLTLRTMEGGE  540

Query  1064  PEIPVYKVIQCMGPTNTRLYTVAVYFKGKRLAEASGHSIQQAEMNAAKVALEHSQSLFPQ  1123
             PEIPVYKVIQCMGPTNTRLYTVAVYFKGKRLAEASGHSIQQAEMNAAKVALEHSQSLFPQ
Sbjct  541   PEIPVYKVIQCMGPTNTRLYTVAVYFKGKRLAEASGHSIQQAEMNAAKVALEHSQSLFPQ  600

Query  1124  LDHQKRVIAKSMKQEPLNSTASKKKQSKDYYEEKSYKKYDYSNSRKKIKKGNTK-ASSKE  1182
             LDHQKRVIAKSMKQEPLNS ASKKKQ+KD+Y++K Y KYDYSN  KKIK+ N K +SSKE
Sbjct  601   LDHQKRVIAKSMKQEPLNSAASKKKQNKDHYDDKKYSKYDYSNLHKKIKRENIKLSSSKE  660

Query  1183  SSPTSRRFSSSNDKSDSDSDSHRKIKKYKHKKSHE--NSLDRSSDEYYSDREIKKKKIRY  1240
             SSP SRRFSSSND+SDSD ++  KIKKYKHKK HE  +S   SSDEY SDRE  +KKIRY
Sbjct  661   SSPISRRFSSSNDRSDSDEENLGKIKKYKHKKYHEYSSSRMSSSDEYCSDREFNRKKIRY  720

Query  1241  YSSSSTSSSEGESSVDQSTNSSNKSMKGYESDEELTKKYSFYTNELKSLKDQLAQEGKSN  1300
             YSSSSTSSSE ESS+ QS NSS KSM+ YE DEEL+KKY FYTNEL+ +K++L +EGKSN
Sbjct  721   YSSSSTSSSEDESSIGQSPNSSKKSMQKYEPDEELSKKYLFYTNELECMKNELDEEGKSN  780

Query  1301  LLNQVD-KKEVDSLSEDENS  1319
             +L ++D K EVD++SEDENS
Sbjct  781   VLKEIDIKTEVDTVSEDENS  800
```

Pasha

>TRINITY_DN3240_c0_g1_i1 len=2585 path=[0:0-609 2:610-741 4:742-2584]

CTTTGGTTTACGCTTTTAGCCTCAGCTGGCTGAGGTTTCATCGTGTTAAGGAGTCAATTATTTCGTATTTATT
TTTTATATAATTAGGAAATGCATTTCGATTTTGATACGGTTCCATAATATTTTAAATATCTTCTATAAAATCG
GTGTTAAGTTAGTTCCGAAAGATTCCTTATAAAATATCTAAGTCATAAAATGGAGTCATTAGGGAAAAGGA
AATGCCCCTATGCAAGAATAAATGAACCATTTTCAGGTTCTGCCTCCTGTAACATGAAATCGAATGCAAAA
AAGCCTTGTTTATTGCAGAATGTCTCTGAAAATAAAGTTTCTATGAGTAAGAGCACTTCATCAGAAGTTACA
AATTATGAAAGTAATCCAGACTCAAATCCTTATTCGCTGGATAACCTCATTGATGTATCAGCATCAATGGA
ATCTGTGGAATCATGGAATTGCTCCTATCCTGAGTACAATGAACAATACGCTGATACCTCATCTTATTGTGT
GAATAATCAAACAGAACCTGAAAATGTACAATCTACCAGTACTGGCTTCTCAAAAGAAGTTGATAATAATA
AATGTAACACTGGTACCAATCCTTATTCCTTTGAAGGAAATGAAGACCTAAGGGTATTCCAAGTTCTCGAT
GAATGTGAAGGTTCAGATGTCGATGAAAGCATAGAACATCAGTCGGATATAGAATTTGACATAAGCGATA
ATGAAATTGAAGCTATGCTGGAAGAAGGTTTTGAAAAAGGAGTGAAAAACAGTAAGACTGAATCTGATGG
TAACAGCCAGGGCGAAAATGGTATTAGATATACAGAACATAAAACATTAATATTAGAAGAACTAGGATAT
AACCATTTTGAAATGCTACCAGAAGGTTGGATCAAAATCATTCACAAAAGTGGGATGCCAATATATTTGCA
TAAGGCAACTAGAGTATGTACTTTTAGCCGTCCTTACTATTTGGGGCAAGGAAGTGTTAGGAAGCATAATG
TGCCCTTAAGTGCCATACCTTGTTTGCAATATCGCCGTGCTCTTGATGAGGAAGAAATTAGGAAAACAGAA
GAAGAAGATGCTTCAAATGTAAAAAGTGATGGTCCGCAATATTTGCATACTGCTAGAATTGAAACTACGGA
AGAAAATAAATTGTCACAAAGCCTTGGCGCTTTTACATTACAACAGTATTGTAAAAAATTATTCCGTTTCAA
AAAAGAGCAGAGGTTACATTTTGTTTCATGGGCTGATAGACGTAAGTATGCAAAAGCGAAAAAAATTGAA
AAAGAACAACATAGACCATCTTTTCCTGATGGAACAAAGCTTATCAAGTTTCCTATACAAAATCCAGATGA
TGGTCCTAAAGCAAAACCTAGGGGAGAATGGATAATGAATCCCAATGGGAAAAGCTATATTTGCATTTTGC
ATGAATATGTTCAACATGCTCTTAAAAAACAACCAACTTATGAATTCAAGGCTTTAGAAAATCCTTCAACTC
CTTACTCAGCAACAGTGATTATCAATGACATGAAATATGGTTATGGAACAGGAACAAGTAAGAAACAAGC
AAAACTTGCTGCTGCAAAGGCTTCTTTGGAAATATTAATTCCTGAAATGAGAGAAAAAATAAGAAGTGATA
ATAAAAATGGAAAAAGGAGTGGAATAACTAATTTCCCAGATGAAGAAGAAACAAGTTTATCAATATTTGA
TGAAATACGGATAGAAGATCCAAGGGTTGCTGAGTTTTGTGCCAAAACCACTGAGCCTTCCCCTTATTCTAT
TTTATTAACTTGCTTACAAAGAAATTTTGGACTCAAAGAAATGCCGATTGATTATAAAGTTAATCCTCTTCA
TCGGCAATCAAATGAATTTATTATGACTGTTGGAAAGCACACTGCACGCATTATTTGCAAAAATAAGAAAG
AAGGAAAACAAAGAGCTTCTCAAGCTATATTGCAGGATCTTCACCCTCATATAACAAGTTGGGGTGCCCTC
CTAAGGCTATATGGCAACCGTTCTGTGAGAAATGTTAAAGAAAAAAAACAAGAAGAACAAGAGATTACAA
TGTTACAGAGTAAAGCTTCTTTGAATTCTCCAAATTATTCTATTATTAACAAATTAAGAAGTGAAATGAATA
AGCTTCATGAGCAACAGATGATGGTGAAACCTATAGGTTTGTTTATACCTCCTGCTGGAACATCGTTGCCTA
GTTCAGGAACAGAACTGAATAAAGTAGATTTTTAGGTTTTCGTGAATACGTCATTAAGTACACTTCTGATCA
ATGAAGTTGTTAATATATAATTACAGAATAGTAAATTCTGTCAGCATATTAATTTAAGGAACTATTAAACAT
TAAGTGCCTGAAAGACAATTTAAAATACTGTAATGGTTGAATAATCCATAAACAATTTTTGTTAGTGTGTAA
CAAAGTGAGTATGATTCTTAAAACACAGAAAATTCACTCATAATTAAATTTAAAAGCATGCAATTCTGTGTT
GACTGAGTATAGTTTTTAAGTAGAAAATAGTAACTTCTAAGCTGTTTTTGTAATTTATTAAACAATAAAGCA
ATTTTGTATAAGAAAAAAA

Protein

RF: +1

ORF: 196 -> 2241

Length: 681 aa

>lcl|ORF1_TRINITY_DN3240_c0_g1_i1:195:2240 unnamed protein product

MESLGKRKCPYARINEPFSGSASCNMKSNAKKPCLLQNVSENKVSMSKSTSSEVTNYESNPDSNPYSLDNLIDVS
ASMESVESWNCSYPEYNEQYADTSSYCVNNQTEPENVQSTSTGFSKEVDNNKCNTGTNPYSFEGNEDLRVFQV
LDECEGSDVDESIEHQSDIEFDISDNEIEAMLEEGFEKGVKNSKTESDGNSQGENGIRYTEHKTLILEELGYNHFE
MLPEGWIKIIHKSGMPIYLHKATRVCTFSRPYYLGQGSVRKHNVPLSAIPCLQYRRALDEEEIRKTEEEDASNVK
SDGPQYLHTARIETTEENKLSQSLGAFTLQQYCKKLFRFKKEQRLHFVSWADRRKYAKAKKIEKEQHRPSFPDG
TKLIKFPIQNPDDGPKAKPRGEWIMNPNGKSYICILHEYVQHALKKQPTYEFKALENPSTPYSATVIINDMKYGY
GTGTSKKQAKLAAAKASLEILIPEMREKIRSDNKNGKRSGITNFPDEEETSLSIFDEIRIEDPRVAEFCAKTTEPSPY
SILLTCLQRNFGLKEMPIDYKVNPLHRQSNEFIMTVGKHTARIICKNKKEGKQRASQAILQDLHPHITSWGALLR
LYGNRSVRNVKEKKQEEQEITMLQSKASLNSPNYSIINKLRSEMNKLHEQQMMVKPIGLFIPPAGTSLPSSGTEL
NKVDF

Conserved Domains

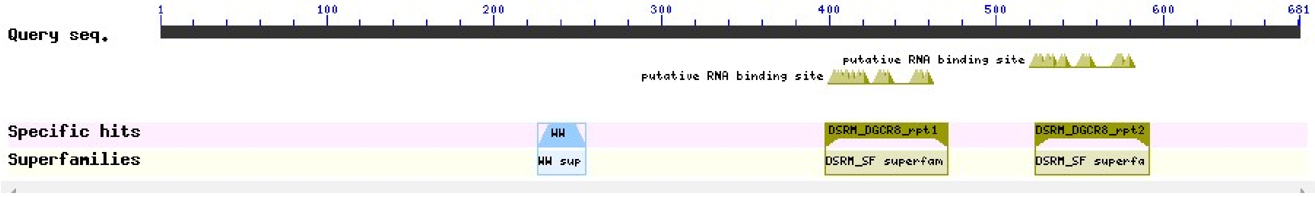

BLASTp

XP_014282581.1 PREDICTED: microprocessor complex subunit DGCR8 [*Halyomorpha halys*]

Score:1210 bits

E-value: 0.0

```
Query  42   MESLGKRKCMYGALNESFTGSASCSIKVNPKKPCFDQNLSENKIPMSKSLTSEVTNYESN  101
            MESLGKRKC Y  +NE F+GSASC++K N KKPC  QN+SENK+ MSKS +SEVTNYESN
Sbjct  1    MESLGKRKCPYARINEPFSGSASCNMKSNAKKPCLLQNVSENKVSMSKSTSSEVTNYESN  60

Query  102  PATDPYSFDNLINVSKSKMESVGTWKYTYQELNKQFTDSPSCSVKGTVENPCLDQKDPEN  161
            P ++PYS DNLI+VS S MESV +W  +Y E N+Q+ D+ S  V        +Q +PEN
Sbjct  61   PDSNPYSLDNLIDVSAS-MESVESWNCSYPEYNEQYADTSSYCVN--------NQTEPEN  111

Query  162  VPSTSREFTKEEVDIDISNMISNPYSFDENEDLRVFQVLDEWGCEGSDVDESIDQPSEVE  221
            V STS  F+KE VD +  N  +NPYSF+ NEDLRVFQVLDE  CEGSDVDESI+  S++E
Sbjct  112  VQSTSTGFSKE-VDNNKCNTGTNPYSFEGNEDLRVFQVLDE--CEGSDVDESIEHQSDIE  168

Query  222  FDISDNEIEAMLEEGFEKGVKKTKAESDVNSQGDNGIRYTEHKTLILEELGYNHFEMLPE  281
            FDISDNEIEAMLEEGFEKGVK +K ESD NSQG+NGIRYTEHKTLILEELGYNHFEMLPE
Sbjct  169  FDISDNEIEAMLEEGFEKGVKNSKTESDGNSQGENGIRYTEHKTLILEELGYNHFEMLPE  228
```

Query 282 GWIKIIHKSGMPIYLHKATRVCTFSRPYYLGQGSVRKHNVPLSAIPCLQYRRALDEEEIR 341
GWIKIIHKSGMPIYLHKATRVCTFSRPYYLGQGSVRKHNVPLSAIPCLQYRRALDEEEIR
Sbjct 229 GWIKIIHKSGMPIYLHKATRVCTFSRPYYLGQGSVRKHNVPLSAIPCLQYRRALDEEEIR 288

Query 342 KKEEKDSSNVKSDGPQYLHTAKIETTEENKLSQSLGAFTLQQYCKKLFRFKKEQRLHFVS 401
K EE+D+SNVKSDGPQYLHTA+IETTEENKLSQSLGAFTLQQYCKKLFRFKKEQRLHFVS
Sbjct 289 KTEEEDASNVKSDGPQYLHTARIETTEENKLSQSLGAFTLQQYCKKLFRFKKEQRLHFVS 348

Query 402 WADRRKYAKAKKIEKEQHRPSFPDGTKLIKFPIQNPDDGPKAKPRGEWIMNPNGKSYICI 461
WADRRKYAKAKKIEKEQHRPSFPDGTKLIKFPIQNPDDGPKAKPRGEWIMNPNGKSYICI
Sbjct 349 WADRRKYAKAKKIEKEQHRPSFPDGTKLIKFPIQNPDDGPKAKPRGEWIMNPNGKSYICI 408

Query 462 LHEYVQHALKKQPTYEFKALENPSTPYSATVIINDMKYGYGTGTSKKQAKLAAAKASLEI 521
LHEYVQHALKKQPTYEFKALENPSTPYSATVIINDMKYGYGTGTSKKQAKLAAAKASLEI
Sbjct 409 LHEYVQHALKKQPTYEFKALENPSTPYSATVIINDMKYGYGTGTSKKQAKLAAAKASLEI 468

Query 522 LIPEMREKIRSDNKNGKRSGITNFPDEEETSLSIFDEIRIEDPRVAEFCAKTTEPSPYSI 581
LIPEMREKIRSDNKNGKRSGITNFPDEEETSLSIFDEIRIEDPRVAEFCAKTTEPSPYSI
Sbjct 469 LIPEMREKIRSDNKNGKRSGITNFPDEEETSLSIFDEIRIEDPRVAEFCAKTTEPSPYSI 528

Query 582 LLTCLQRNFGLKEMPIDYKVNPLHRQSNEFIMTVGKHTARIICKNKKEGKQRASQAILQD 641
LLTCLQRNFGLKEMPIDYKVNPLHRQSNEFIMTVGKHTARIICKNKKEGKQRASQAILQD
Sbjct 529 LLTCLQRNFGLKEMPIDYKVNPLHRQSNEFIMTVGKHTARIICKNKKEGKQRASQAILQD 588

Query 642 LHPHITSWGALLRLYGNRSVRNVKEKKQEEQEITMLQSKASLNSPNYSIINKLRSEMNKL 701
LHPHITSWGALLRLYGNRSVRNVKEKKQEEQEITMLQSKASLNSPNYSIINKLRSEMNKL
Sbjct 589 LHPHITSWGALLRLYGNRSVRNVKEKKQEEQEITMLQSKASLNSPNYSIINKLRSEMNKL 648

Query 702 HEQQMMVKPIGLFIPPAGTSLPSSGTELNKVDF 734
HEQQMMVKPIGLFIPPAGTSLPSSGTELNKVDF
Sbjct 649 HEQQMMVKPIGLFIPPAGTSLPSSGTELNKVDF 681

<u>Exportin 5</u>

>TRINITY_DN9946_c0_g1_i3 len=5845 path=[0:0-426 3:427-432 4:433-447 6:448-2273 7:2274-3587 8:3588-5844]

GAAAGGTTAGACCGCATACATGTTCAGGATGACTTAAATTGTTTTAAATTTCTTCTATTAAACGTCGATCCA
TTGGATCTGCGGACAGTGCTTTAACATTATTTAAGTTTATTTCTCATTCTCATTCCTTGACAAATAGAAAAA
AAAATTACATCAACCAGTCTCCATTGCCTACAGCTTCATTGTTTTTAATTGGAAACCATTATTTGGAATGGA
TTTTTGAGTTTATTCATTAATAACAATAGTTCAAGTTCGTTGTTTTTTCAATATACGTGAATCGAAGTCCTTG

ATTGTTGTGATCTTTTATTTATATAATGAAATGTGAAGGCATGGTTCTCACATGCTTTTGGAACTAATGTCA
AGAAGTGTAATCTATGAAAGTACAAAGACATCTGTTGTTTCATCTCCTGGACTAGTCATTTAGTTTAATTTT
GAAGAGAAGAAAAAATCTTAAAAATGGATAACGAAATAAGCTCTGTAGCTACACAATTGGCAGCCGCAGT
GGAATTGACAATGAACCCACTCGCGTCGCAGGCTGAAAGATTAGAAGCTTACAATGCGTGTGAAGTTTTCA
AAGAAAAGAGTCCATTGTGCGTCCAGTGCGGTTTGTATTTAGCACAAAGAACTGATTATTCACATTTCGTCA
GGCACTTCGGATTACAACTGATGGAGCACTGTATCAAATATCGTTGGTACAATATGACTCAGGCCGAAAAA
CTATTCATAAAGGAAAACGCAATGAAATTAGTTGAACGTGGTATGGATACAACTTTAGAAGATAAGGCTCA
TATGAGAGATGCATTGTCTCGTGTAGTTGTGGAAATGATTAAAAGGGAATGGCCACAACAGTGGAATACGT
TGTTAGCGGAACTGAATCAATGTGCTTCACAAGGGAACATTCAGACTGAAATGGTTTTGTTGGTTTTACTCA
GACTTGTTGAAGATGTTGCTGTATTACAGACATTGGAGTCCAACCAACGGAGGAAAGATTTGTATCAGGCA
TTAACGACAAACATGTCTGATTTGTTTATGTTTTTTCGAGGACTGATCGGGAATCATTATGAGCAATATCGT
TCATGTCTCGAAGTAGGAAATGTAGCTATGGCCAACTCCCATGCTAGGGTTGTACAGGTTGTATTATTAACT
TTGAGCGGTTTTGTCGAGTGGGTTTCCATGACCCATATCATGGCAGAAGATGGAAAATTACTCCAAATATTC
TGTGTTTTATTGAACGATGAGAACTTTCAAGATGGATCTGCCGAATGCTTACTTCAGATTGTATCGAGAAAA
GGCAAACTTGATGAAAGGAGGCCCCTGTTGATATTATTTAGCGAAGACGCTATGAGGTGTATCTTTCAATC
AGCTGAGAACAAGAATTACCCGTTTTTAAAAAAACTAACTCGTGTGCTGACAGGGCTTGGAACACAGCTAT
GCTCGCTGTGGAGCAAGGACGGACAGTCCCGCCCTCCCAACTTCCAGATCTACTTAGAGGCCATTGTAACG
TTCACTCGCCACCCCTCCCTCAGCCTAGTCTGCTACGCCAACTCCCTCTGGCTGGCGTTGATGAAGCACGAT
CAGATCTGTCGGGATGAAGTGTTTTTAAGTTTTGTTCCAAAATGGGTCGAAGCTGCAGGGCCTAAAATCAT
GAAGGTACCTTTCCCATCATCCAAGTCGAATTTGGAACCTAACACTCCAGAGTCCTACGCTGTTCAAGATTT
TGATGCGGAAGATGAATATAACGCTTTTTTCCATAAACATCGCGTAGAGCTCCTTGATACGTTTAAACAGG
CTACATTAGTCGCACCATTGGTTGCGTATTCTTACGTTGAAAGGTGGTTGGAAGTGCAGATCCAGAAGACT
GTGACGCAAAACAATATGGAGCCATGCACTTTAACTTCTGTCGCTTATCTGGAATGGGAAGCTCTATCGCT
GGCCTTGGACGCGGTGCTTGGTAAGCTGGTGATGGCTAAGGAGAGGCCGGATGTGAACAACGGCCTCAAG
CTGCTGGTGCTCTGCCTGCAGCTGGAGCCGACCGACCCCCTCATCCTGTCGGCCATGTTGTCCTGCATATCT
GCCCTCTTTGTCTTCCTCAGCATGGCTCCACCGGAAACAACTGCGATCTATTTACCAAAAGTGCTGGACAAG
ATCTTCGCGACCTTGGTCTTCACCATCCCCGGAGAGCCTAAGAACCAGAGATCGCGTGGGGTGAAGAACCT
GCGGCGCCATGCAGCCTCCTTGATGGTCAAGATTGCCCACAAGTACCCTCTTCTGCTCCTTCCTGTCTTCTCC
AGGATACACGGTATTGTCGTCAGCCTCCAGGCCAAGCCTCACACCTTGTCAACGATGGAGGCCTTGTGTTT
ACAGGAAGCCCTGCTCCTGATCAGTAACCACTTGTGCGATTATGACCGAGAGTGCAGGTTCATCGGGGAAG
TGATGGCCCCTGTGATTGGCCCATGGAACAACATGGCCTCGACGGCGTTCTTGTCCACCCCCGCCTTCATGT
CCTACGTGGGGTTGGACAAGCCTCCAGTAGAGCCCAGTTCTGACGATATCAACGGCCAGAACAGATCCCAG
ATAATGTGGTGCTTGGACGTGATCCTGGCCGTGGTGAAGCGAGTGATGTGGCCGAGCGACCCAGAGCTGGC
CGCTCGAGGAGGGTTCCTGGTCTGTACGACGGAGGCGGGGAATCCTATCTACAGGAACCCTGCCACCCCGC
ACGTCCTCCCTCTCCTCCCGGGCCTCTTCAGCCTTGCGTTTGTCATCAACGGTCTATCGTCACCAGCTGCTCT
TGCCAATTTATCAGAGGGATATAGAGGAGCTTACATGATGCTTGAACTGGAGAGACAGAACTTGCTGGGGA
TCGGGAGCACCAGCGAAGTGATGAACAATGAGTCCAAAGGAGAGACAACCCCGCTGCAAAGGATGCAGA
ACTTCATCTCCAATGTTCACGAGGACTGCTGCCATGCCCTAGCTCACGCCTTCCACGCCCTCAGCCACGACA
TGTATCATGTACCAAACCTGGCCCACTCCCTCATAGCCACGGTCTTCTCAAATCTAGAGTTTGTACCTGACC
ATAGGATGAGGCCTTTTGTTCGCGTGTTTTTGAAATCTTTTGTCACGAGCTGCCCACCGTCTTGCTACGATA
CAGTCCTAACTCCTGTTCTGGCCAACTTTACTCCTTACATGTTTGCCAGACTGAATGCGAGATGGCAGCATT
TATCCGAATTAAGCGAAAATGGTAATGCAGATGACCATGAAGAGAATACAGACACTCAGGAGATGCTGGA
AGACATGCTGAACCGCACCCTGACCAGAGAGTACTTGGACGTGCTGAAGGTGGTGCTGTACGGAGGAGCG
GAGGCAGGCAGTGGGGCGGGGAGCATGGAGGAGGAGGGCCAGGACGTCAAGCCTCAATCGGACGTCATC
AGCGAGCTAGGGATGAGGGTCTTGGCCTTTGATCCAACATCCCAGCCCATAGTACTCTGTCTGTTGAGAGC
CCTGTACTGGGGAGACAGCACGGCCAGCTTGAAGTGCACCTCCCTCCTGGGCGGGGTGATGCTGCACTTGA
CGTCGACCGGGAAGATGAGCGCCGAGCTGGCCTGCCACGCCCTCACGTCAATCCTCTTCGGCCTCCAGTGC
CACGGCCAGCACGACGCCAACCAGGGCTCTCTTCTCGTCTTGGGGACTCTGATGTACGGCATCCTGAGGCC
TATAGCCCCGGAGATACTTCAAGTGATGAAGCAGATACCAGATGTGAATATGATCGATCTACAGAAACTGG
ACGACAGGATGTTGAAGGAGGTTCAGAAGGGAACTAAAGTAGACAAAGCAAAAAAAGAAATGTTCAAAA
AAGTTACTAGCCCCTTGATAGCAAGAAATGTCGGACAACTGTTCAGGAAAAAAGTTGCCATTCGAGATCTG
CCGAAAATTGAAAGTATGAAGCCGAAATTCAATTCAGATCTTCTTTTCAACAACGACGAGAACGGTTTAGT

GAAGCTGTTCAAGACGTAGCATCGTATATCTCTTCAACGCTTCTTGTACTGTGTCGTATAGTTCTAGTCCCA
AGTGTTGTCATGCGACAGGTTTCCTACCCCTTCATATCCTATCGTTTAATTTTAATTTTCGCAAAATTTGATG
TGATCAAAATTAACTTATTTTTTAGTTTATAAGCATTTGTTTTTCATTTTCTTTATTACTATTATTAATTTTTTT
TTTTACATTCCATCATGAATGTAATACAAAAACACTATTGTCTCAATATGATCGATCTTTAAAAGAATGAAA
TAGACTTGTTATAAATTTTGGACTCTTAATCGTTAATTATTATTATAATTTAATTAAATTAAAAAAATATCTT
AGTAATACTCCAGAATGCCAGCTCATAAGTTATAATATATAAAAATTAAAACAGGCGATCAGTAGTAAAGG
CTTCTGTCTGAAGGAAATTCTTATTCCCACTACTCAATCTGTTTCCTGATGTCTTTATGGTTATTTTCTGGTTT
TGAACGTATCTTCAATTCTTGCCAGCAGCATGTATGGTGAAGAAATTATAAAGACAGTTCAAAACCGGAAA
AATGCTATGAAGATAGCAAGGATATTTGCTTTCAAGCCTTAAAGACTAAGTGGCTGCATGAATATTTTGTTT
TCTTTCTTATCAGCTGTATATGATTTTTAAACTAAAATTATAATAGATCGGAAATATTTTTTCCTCTCAGTTA
ATAATTTTTCATTTATAAAATCAAATAATGGTCTCTAAACAATTTATCAATTAATTTCAAATTTAAGTTGTCC
CTTTCATGTATTCTGGCGTTGACCAATTATAACATTATAAAAAGTAAGAAAATTATGGAATTATCATTAGTT
TTATATATTAGATATAATAATACTAAGAGTGTTTCGACGGTAAATGGGCGATTCCTAATATAGAAAACTAT
ATTTTGGAATGTTGACTGAAAGTCTACAAGTAAGACATTTATATATAAGAGGTGTGATTTGAACACACTAA
TTCTAAAACTTTTCTTGGGTTGCTGAAAAATAGTAGAAAAGTGTATTTAAGCCAGTGATTTCATTTTAATTC
CAAAAATAAAAATTTGGTTAAAATGTTTAATAAAAAAATTTATTTTTATTTTTATTATTTTTTAGTTGTAATG
ATTTAAAATTCTACTAGTTTTTGGCCCTGAGAAATGTATTTTTTAGTTAGTGTGTTAAGTCTTTAAAAAAACA
CAAAAAAAAACAACATTTCTTTCCACTAAATAAAATTGTAAATGTTTATTTTGTATATAGTACGTACATTAT
TATTATTATTATTTTTGTTTTATTGTTGTAATATATAATGGATGAAATTATGATGGTGTGTTATGTAGGCGAA
TACGGTTGAATGTTATTAACTGTAATACGTGTATATGTATTGTACATTTGTGTTGATTCAGTGTTGTTATATG
TATATATATTATAATCGTGGGTCATTTGTCCAATGAACGAACTTGTTATATTAGATATAATTGAATAATAAA
GATGATTGAAAAAAAAAAAATTAAATACAAAAAAAAAAATAACAAAATACTGAATGTGTTTCCGGTTTTTAAA
AATTGTAATTATAAAAAAAAAAAAGTTTTGAGATTTTGAAGAAAAAGGGTAATTTTTTTATATGATTTGTTTT
ATAAGATTGACAAAGGTTGCTTATGCTTGTTTTTGTTTGTTTAGTTTTTTTAATATTGAAGAATTGTACTGAG
AAATTATTATTTTTTATTGTTATTCTAGAATAATAAATAAAAAAATATATACTTTCATTAAACTGTAATACAT
AGCTATACAAACCAGAATAAACGTATCATCTATCAAAAAAAAA

Protein

RF: +1

ORF: 457 -> 4011

Length: 1184 aa

>lcl|ORF1_TRINITY_DN9946_c0_g1_i3:456:4010 unnamed protein product

MDNEISSVATQLAAAVELTMNPLASQAERLEAYNACEVFKEKSPLCVQCGLYLAQRTDYSHFVRHFGLQL

MEHCIKYRWYNMTQAEKLFIKENAMKLVERGMDTTLEDKAHMRDALSRVVVEMIKREWPQQWNTLLAELNQ
CASQGNIQTEMVLLVLLRLVEDVAVLQTLESNQRRKDLYQALTTNMSDLFMFFRGLIGNHYEQYRSCLEVGNV
AMANSHARVVQVVLLTLSGFVEWVSMTHIMAEDGKLLQIFCVLLNDENFQDGSAECLLQIVSRKGKLDERRPL
LILFSEDAMRCIFQSAENKNYPFLKKLTRVLTGLGTQLCSLWSKDGQSRPPNFQIYLEAIVTFTRHPSLSLVCYAN
SLWLALMKHDQICRDEVFLSFVPKWVEAAGPKIMKVPFPSSKSNLEPNTPESYAVQDFDAEDEYNAFFHKHRVE
LLDTFKQATLVAPLVAYSYVERWLEVQIQKTVTQNNMEPCTLTSVAYLEWEALSLALDAVLGKLVMAKERPD
VNNGLKLLVLCLQLEPTDPLILSAMLSCISALFVFLSMAPPETTAIYLPKVLDKIFATLVFTIPGEPKNQRSRGVKN
LRRHAASLMVKIAHKYPLLLLPVFSRIHGIVVSLQAKPHTLSTMEALCLQEALLLISNHLCDYDRECRFIGEVMA
PVIGPWNNMASTAFLSTPAFMSYVGLDKPPVEPSSDDINGQNRSQIMWCLDVILAVVKRVMWPSDPELAARGG
FLVCTTEAGNPIYRNPATPHVLPLLPGLFSLAFVINGLSSPAALANLSEGYRGAYMMLELERQNLLGIGSTSEVM
NNESKGETTPLQRMQNFISNVHEDCCHALAHAFHALSHDMYHVPNLAHSLIATVFSNLEFVPDHRMRPFVRVFL
KSFVTSCPPSCYDTVLTPVLANFTPYMFARLNARWQHLSELSENGNADDHEENTDTQEMLEDMLNRTLTREYL
DVLKVVLYGGAEAGSGAGSMEEEGQDVKPQSDVISELGMRVLAFDPTSQPIVLCLLRALYWGDSTASLKCTSL
LGGVMLHLTSTGKMSAELACHALTSILFGLQCHGQHDANQGSLLVLGTLMYGILRPIAPEILQVMKQIPDVNMI

DLQKLDDRMLKEVQKGTKVDKAKKEMFKKVTSPLIARNVGQLFRKKVAIRDLPKIESMKPKFNSDLLFNNDEN GLVKLFKT

Conserved Domains

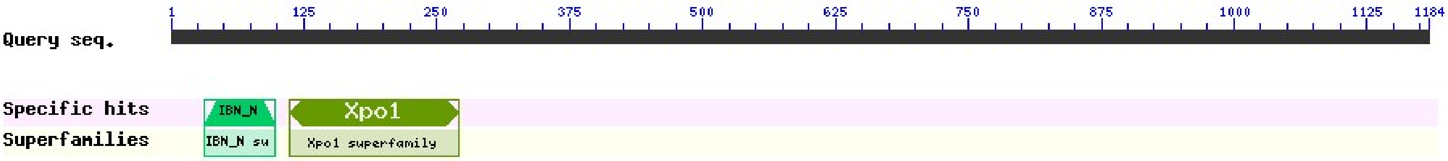

BLASTp

XP_014280932.1 exportin-5 [*Halyomorpha halys*]

Score:2405 bits

E-value: 0.0

Query 1 MDNEISSVATQLAAAVELTMNPLASQAERLEAYNACEVFKEKSPLCVQCGLYLAQRADYS 60
MDNEISSVATQLAAAVELTMNPLASQAERLEAYNACEVFKEKSPLCVQCGLYLAQR DYS
Sbjct 1 MDNEISSVATQLAAAVELTMNPLASQAERLEAYNACEVFKEKSPLCVQCGLYLAQRTDYS 60

Query 61 HFVRHFGLQLMEHCIKYRWYNMTQAEKLFIKENAMKLVESGMDTTLEDKAHMRDALSRVV 120
HFVRHFGLQLMEHCIKYRWYNMTQAEKLFIKENAMKLVE GMDTTLEDKAHMRDALSRVV
Sbjct 61 HFVRHFGLQLMEHCIKYRWYNMTQAEKLFIKENAMKLVERGMDTTLEDKAHMRDALSRVV 120

Query 121 VEMIKREWPQQWNTLLAELNQCASQGNIQTEMVLLVLLRLVEDVAVLQTLESNQRRKDLY 180
VEMIKREWPQQWNTLLAELNQCASQGNIQTEMVLLVLLRLVEDVAVLQTLESNQRRKDLY
Sbjct 121 VEMIKREWPQQWNTLLAELNQCASQGNIQTEMVLLVLLRLVEDVAVLQTLESNQRRKDLY 180

Query 181 QALTTNMSDLFMFFRGLIGNHYEQYRSSLELGNVAMANSHARVVQVVLLTLSGFVEWVSM 240
QALTTNMSDLFMFFRGLIGNHYEQYRS LE+GNVAMANSHARVVQVVLLTLSGFVEWVSM
Sbjct 181 QALTTNMSDLFMFFRGLIGNHYEQYRSCLEVGNVAMANSHARVVQVVLLTLSGFVEWVSM 240

Query 241 GHIMAEDGKLLQIFCVLLNDENFQDGSAECLLQIVSRKGKLDERRPLLILFSEDAMRCIF 300
HIMAEDGKLLQIFCVLLNDENFQDGSAECLLQIVSRKGKLDERRPLLILFSEDAMRCIF
Sbjct 241 THIMAEDGKLLQIFCVLLNDENFQDGSAECLLQIVSRKGKLDERRPLLILFSEDAMRCIF 300

Query 301 QSAENKNYQFLKKLTRVLTGLGTQLCSLWSKDGQSRPPNFQIYLEAIVTFTRHPSLSLVC 360
QSAENKNY FLKKLTRVLTGLGTQLCSLWSKDGQSRPPNFQIYLEAIVTFTRHPSLSLVC
Sbjct 301 QSAENKNYPFLKKLTRVLTGLGTQLCSLWSKDGQSRPPNFQIYLEAIVTFTRHPSLSLVC 360

```
Query  361   YANSLWLALMKHDQICRDEVFLSFVPKWVETAGPKIMKVPFPSSKSNLEPNTPEMYAVQD  420
             YANSLWLALMKHDQICRDEVFLSFVPKWVE AGPKIMKVPFPSSKSNLEPNTPE YAVQD
Sbjct  361   YANSLWLALMKHDQICRDEVFLSFVPKWVEAAGPKIMKVPFPSSKSNLEPNTPESYAVQD  420

Query  421   FDAEDEYNAFFHKHRVELLDTFKQATLVAPLVAYSYVERWLEVQIQKTVTQNNLEPCTLT  480
             FDAEDEYNAFFHKHRVELLDTFKQATLVAPLVAYSYVERWLEVQIQKTVTQNN+EPCTLT
Sbjct  421   FDAEDEYNAFFHKHRVELLDTFKQATLVAPLVAYSYVERWLEVQIQKTVTQNNMEPCTLT  480

Query  481   SVAYLEWEALSLALDAVLGKLVMAKERPDVNNGLKLLVLCLQLEPTDPLILSAMLSCISA  540
             SVAYLEWEALSLALDAVLGKLVMAKERPDVNNGLKLLVLCLQLEPTDPLILSAMLSCISA
Sbjct  481   SVAYLEWEALSLALDAVLGKLVMAKERPDVNNGLKLLVLCLQLEPTDPLILSAMLSCISA  540

Query  541   LFVFLSMAPPETTAVYLPKVLDKIFATLVFTIPGEPKNQRSRGVKNLRRHAASLMVKIAQ  600
             LFVFLSMAPPETTA+YLPKVLDKIFATLVFTIPGEPKNQRSRGVKNLRRHAASLMVKIA
Sbjct  541   LFVFLSMAPPETTAIYLPKVLDKIFATLVFTIPGEPKNQRSRGVKNLRRHAASLMVKIAH  600

Query  601   KYPLLLLPVFSRIHGIVISLQAKPHTLSTMEALCLQEALLLISNHLCDYDRECRFIGEVM  660
             KYPLLLLPVFSRIHGIV+SLQAKPHTLSTMEALCLQEALLLISNHLCDYDRECRFIGEVM
Sbjct  601   KYPLLLLPVFSRIHGIVVSLQAKPHTLSTMEALCLQEALLLISNHLCDYDRECRFIGEVM  660

Query  661   SPVIGPWNNMASTAFMSTPAFMSYVGLDKPPVEPSSDDINGQNRSQIMWCLDVILAVVKR  720
             +PVIGPWNNMASTAF+STPAFMSYVGLDKPPVEPSSDDINGQNRSQIMWCLDVILAVVKR
Sbjct  661   APVIGPWNNMASTAFLSTPAFMSYVGLDKPPVEPSSDDINGQNRSQIMWCLDVILAVVKR  720

Query  721   VMWPSDPELAARGGFLVCTTEAGNPIYRNPATPHVLPLLPGLFSLAFVINGLSSPAALAN  780
             VMWPSDPELAARGGFLVCTTEAGNPIYRNPATPHVLPLLPGLFSLAFVINGLSSPAALAN
Sbjct  721   VMWPSDPELAARGGFLVCTTEAGNPIYRNPATPHVLPLLPGLFSLAFVINGLSSPAALAN  780

Query  781   LSEGYRGAYTMLELERQNLLGIGSTSEVMNSESKGETTPLQRMQNFISNVHEDCCHALAH  840
             LSEGYRGAY MLELERQNLLGIGSTSEVMN+ESKGETTPLQRMQNFISNVHEDCCHALAH
Sbjct  781   LSEGYRGAYMMLELERQNLLGIGSTSEVMNNESKGETTPLQRMQNFISNVHEDCCHALAH  840

Query  841   AFHALSHDMYHVPNLAHSLIATVFSNLEFVPDHRMRPFVRVFLKSFVTSCPPSCYDTVLT  900
             AFHALSHDMYHVPNLAHSLIATVFSNLEFVPDHRMRPFVRVFLKSFVTSCPPSCYDTVLT
Sbjct  841   AFHALSHDMYHVPNLAHSLIATVFSNLEFVPDHRMRPFVRVFLKSFVTSCPPSCYDTVLT  900

Query  901   PVLAHFTPYMFARLNARWQHLSELSENGNTDDHEENTDTQEMLEDMLNRTLTREYLDVLK  960
             PVLA+FTPYMFARLNARWQHLSELSENGN DDHEENTDTQEMLEDMLNRTLTREYLDVLK
Sbjct  901   PVLANFTPYMFARLNARWQHLSELSENGNADDHEENTDTQEMLEDMLNRTLTREYLDVLK  960

Query  961   VVLYGGTETGNGSGSMEEEGQEVKPQSDVISELGMRVLAFDPTSQPIVLCLLRALYWGDS  1020
             VVLYGG E G+G+GSMEEEGQ+VKPQSDVISELGMRVLAFDPTSQPIVLCLLRALYWGDS
Sbjct  961   VVLYGGAEAGSGAGSMEEEGQDVKPQSDVISELGMRVLAFDPTSQPIVLCLLRALYWGDS  1020
```

Query 1021 TASLKCTSLLGGVMLHLVSAGKMTADLACHALTSILFGLQCHGQHDANQGSLLVLGTLMY 1080
TASLKCTSLLGGVMLHL S GKM+A+LACHALTSILFGLQCHGQHDANQGSLLVLGTLMY
Sbjct 1021 TASLKCTSLLGGVMLHLTSTGKMSAELACHALTSILFGLQCHGQHDANQGSLLVLGTLMY 1080

Query 1081 GILRPIAPEIVQVMKQIPDVNMIDLQKLDDRMLKEIQKGTKVDKAKKEMFKKVTSPLIGR 1140
GILRPIAPEI+QVMKQIPDVNMIDLQKLDDRMLKE+QKGTKVDKAKKEMFKKVTSPLI R
Sbjct 1081 GILRPIAPEILQVMKQIPDVNMIDLQKLDDRMLKEVQKGTKVDKAKKEMFKKVTSPLIAR 1140

Query 1141 NVGQLFRKKVAIRDLPKIESLKPKLNSDLLFNNDENGLVTLFKT 1184
NVGQLFRKKVAIRDLPKIES+KPK NSDLLFNNDENGLV LFKT
Sbjct 1141 NVGQLFRKKVAIRDLPKIESMKPKFNSDLLFNNDENGLVKLFKT 1184

siRNA

Dicer-2 (Dcr-2)

>TRINITY_DN9350_c0_g1_i2 len=5494 path=[1:0-1632 2:1633-5170 4:5171-5493]

CGTAATATTAAGTGCCATTATTATTAATAACAATCAGTGTACTTTAAATGGACATTTAGTTTGTGTCATAG
CTTACAAAAATGAGTGACAAAATAGAAGATTTTAATCCCAGGGAGTACCAAGTGGAACTCTTTGAAAGAGT
GATGAGGGAGAACACCATAATATTCTTACCTACAGGCTCTGGAAAGACATTTATTGCTGTTATGCTTATTAA
GCATAAATCCAACGAATTGAAACTATTATATTCTGAAGGCGGTAAAAGAACATTCTTTCTTGTGGATACAG
TCGCATTAGTTGCTCAGCAGGCTCAAAGTATTCGACAGCAAGCTCCCCTAACGGTTGGAGAATATAGTGGA
GACAAAGGTGTTGACCACTGGGATTTTAAAAAGTGGTATGAAGAGCTTGAAAATAATCAGGTTTTAGTTAT
GACGGCTGCTATATTTTATAATATGATTCTCCATAAGTATGTCTTATTCCCTCGTGTCAATCTTATAGTTATG
GATGAATGTCATCATGCCGTAAATAAACATCCAATGAGGCTGATCATGGATCAGTTTAAAGATGTTCCTGC
AGAGGATAGGCCGCATGTCCTAGGTCTAACTGCAACACTGTTAAATTCTAATTGCAAACCAGAACAAATCT
CTAAGGAAATTCACAACTTGGAAATCACAATGCATGCAAAAATAGCTAGAGCTGACAATGAAGAAATGGT
CCAAGCATTTTCAACTAATCCAAAAGAAGAAATCATTAAGTACGATGTAGACGAAGTGGATGAATGTTACC
AAGTTGTTTCTACTTCAATTGGCGAAATTTACCTGATTTTGAAGTCGATAGTTCTTCTGGATAATTCACCGA
AACCAGCCAGCAATCCTCCGAATGCAGTCTTGATTGACAATAAATCAGATGCTATCAATGAAATCAAAAAT
ATTCTGAATGAAATAACTCTCATCACTGGAGAATTAGGAGGATATGCTTCTACTTATGCATTACTGTTAAGA
ATAGTCCATTTTGAAAGAATGAGAATTTCATCCAATGATCCAACGTTAACAAAACTGTTAGGTCATATTATA
ATACAACTTCATATAATTCGAAAAAAACTCGATGTGATCATGAAGACAACACCTAATAAAATACTTAAATA
TTCAACTTCTAAGATGTTAAAATTGTTCGAATTGCTGAAGAACTTTCGAGAGGATAATTTAGCTCTGGTTTT
TGTTGAAAGAAAGACGACTGCCAAAGTTTTGTACTATGTACTTGATGATTTGGCAGCTCATTTTGAGGAATA
TAGAAATATAAAGCCTGATTTTGTCGTTGGTGTAAACAGCAACCCATATCATTTAACTCGAGAATATATTCT
TGAAAGGAAAAAAAATCAAAAGATTTTAAAAAAATTTAATATGGGTGAGATCAACATATTGGTTGCTTCTG
ATGTGATTGAAGAAGGCATTGATGTAAAGAATTGCAATTACGTCATAAAATTTGATCATCCTAAAACAACA
CGATCTTACATTCAATCCAAAGGCCGTGCAAGGCACAGAGACAGTAAATACATTCTGTTCTACCCCAATTC
AAATTCTGACTCATTTCAGAAAAAGTATATTAATTTTAAACTAACTGAAGAAACCCTAAAACAGGAACTAT
GTTATGGCAGAGATCTTCATAGAAGGGGTGGTACGGAAGCAGAAGCTGAAGCGTTTCTCTATTCCAAAGAA
GTGGAACCTTTCTACATCGACGGGCCTGCTGGTCCAAAAGTGACTGTTGATTCTGCTATTTCTTTATTAAAT
AGGTATTGCTTTTCTTTGCCATGTGATAAATTTACTAAGCTTACTGTGTTTTTTTGGAAGAAGAATGTCATTG
TAGATGGTGTAAATAAAGTTATCTGTTTTCTTCAACTACCAATAAATTGTCCACTGAAAGGGATAATTAAGG
GTCAACCATGTGAAAATCTTGTCACTGCCAAGGGTGCGGTTGCGTTAAACGCTTGTAAAATGTTGTATGAG
ATCGGTGAACTTAATGATCACTTACTTCCTGTAGGAAATGAAGTTCAGAATGTTGAAGAAAAGATACTCTTT

CCTCTATGGGAAAATGAAGATGAAGAAAAGTCCATGTACAATACTGGAACTAAAAAATGTCGCAGGATTT
ATGATAAGCAGCTTCCCAATTGGATCTCTGGTGGTTTTCCAAAACCTGGCCAGCCTGTCTACATTCATGTTC
TAAAAATATTACCTAACTATCCAAAACCGGATAATGACAGGTTATTGGCTTTTTATAATTTACTACAGTCGA
AAAATAATTTTGCAGTTATAACTTCAAAAAAGTGGCCGAAGCTTTGTGATTTTCCGTTATTCATGAACGTCG
GAGAAGTGAGTATAAATATACTACAAAATTCTCGAAAAATTACTCTGAGCCAGGAACAGTGTGAAAAAATT
ATGGCTTTCCATTCTTTGTTATTTACTGAAGCTTGGTTATTGAAGAAAGAATTTTTAGTTAAAGACTATGAA
AACAAAGAAAACTCTTATTATGTTGTCCCGACCATTGTAGATAATGATAATATTGTCATCGATTGGGAAACT
ATAAAGAATCATAAGGAAATTCCTCCAGTGAAAGCGGTACCTCTGGATTCAAGAGGGAATATTGATGTGAC
AAAGGATAGATATCTTCATAAGATAGTTATACCATGGTACCGACCAAAAGGATATATGCTGAAATATGTTG
TAACAAGAGTGTGTGAAGAACAAACACCTATGTCACCCTTTCCGTCAGAGCAATACAGTTCATATTCGGAT
TATTTCTTCAAACGTTACAATCAATCTATCATTAATAAAAATCAGAAATTGATAGAAGTGAGAGCTATTTCT
GGAAAAAACAACTGCTTATTACCGAGAGGAAAATACCGCTTTGGTAAAAAAAGACGTCGTGATGAAGAAG
ATTTTGAAGAGACACTAGTGCCCGAGCTTTGTACCCTTATAGAATTCCCTGCAGTTTACATGCTCAAGGTTA
CTTTGTTACCATCAATCTTGCATCGTATCACTATGCTACTCAATGCTGAAGAATTGCGTCAGAACATTGCTG
ATGGAACGGGATTAGGTGTAGTAAAGCTTCCACAAGGTGTTTATTGGCAGCCTTTGGAAGTCGACCAAACA
GCCTTAATTGGTGATGAAAGTTCAAATGCAAGAAGTACAATCGAGGTGAGTAACATGTTGGCGGATAAGAT
GGTGGAATTGCAGCCTAAAGATTGCACATGGGATAAAGAAGAAGAGCCGATAGACATTGAAAGAAACTTG
AGGAATCTAGATCGCCTTTCAGTTCTCCATTATGAAAGATTTGCAAAAATTGAAGTTACTCTCGACCAACTT
AATAGATCGAATGGTCTGATTTTTGGAACTGGATCATCTTACATGAGAAGCAATATTATAAGGCCACCAAA
AGTTAACGCTCCTGATATTCCTATTTTGTCTAATGTAAGCAGTGTTTTTGGGCCACAACTAAGTTCTGTATTA
AAGGCTCTTACTGCTAGTTCTTCTAATGACGTAATGAACTATGAAAGGCTAGAAACATTGGGTGATTCCTTT
TTGAAGTTTTAGTTTCTTTGATATTATTTATTTACTTCAAAAACAAGGATGAAGGAAAATTAAGCAGTATC
AAAGGGAAAATAATCGGGAATAGAAATTTGTACTACAGTGGAAAACATTTGAACCTTGGAAAAATGTTGA
AGGTCAATGACTTTATTCCTGATGACTGGGAAGTTCCTGGATTTACAATTTTGGAAAGCATTAAAAAAGTAC
TAAAAGATGCTAAAATCCCGCCTTCGGTGCTCTACCAAATAAGCCTCTCAAAAAGGGAACGGGATACTGCA
GTTTTGGAAGAAGAAACGAGGTTGAATATTCAGGAGTTGTTCTGCAATTCCTCACTTGCTGATGAAAGTTCT
TACAGCAGCTTGGGAATTATAGGTCAGCAGTGCGTTGCTGACAAAGTAATTTCTGATTCTGTCGAAGCTCTC
ATAGGAGTCTATCTTGAGGCATGTGGTGTTGAAGGAGCATTTAAATTATGCAAATGGTTGAGCATACTTCCC
GAAAATATGTCTGATATAAACAAAGTATTACATGAACCAGCACCTACTGCTCAGATATCCGATTGCGGTAA
CATGAATGACATACTGATCATGCCAGAGGTTGTAGAATCCATTTTGGACTACAGATTCAAAAACCGAAGTT
TCCTCCTTCAAGCTCTTACTCATTGTTCCTACAGACAGAACATTACCGATTGCTATCAAAGATTGGAATTCC
TCGGTGATGCAATTTTAGACTTCTTGATAACTTCCCACATATATGATAGATGCAAAAATATAACACCTGGTG
AATTAACTGATCTGAGGTCTGCCTTAGTTAATAACGTCACTTTTGCATGTCTGACTGTAAGATATGGCTTCC
ATAAATTTATGCTTACTAAGGCTTGTAAACTGACTGATATTATCAAAAGGTTTGTTGAACACCAAGAGAAA
CGGAACCACAAAATTGGTGCTGAGATATTATTTCTTCTGAGTGAAAACGATGTAAAAGCTGCGGAGATTGT
GGATGTGCCTAAGGTTCTTGGTGATCTATTTGAATCATTAGCTGCGGCTGTTTACTTAGATAGTGGAAAATC
TCTTACTACTGTTTGGAAAGTGTTTTATAAACTGATGCACAATGAAATAGAGGAATTTGTCGGAAACATTCC
TAAGAACAGCATCAGACTCCTTTATGAAAAATTTCCTGTACCAGCACCGTGTTTTGAGAAACCGCAATTCCT
CGATGAAAACAAAACGACAGTCATGGTGACTCTTGTTGTCACCACCAAGAATGAGAGGATGGCCTTCGTTG
GAGTGGGAGAGAACAAGAACCAAGCGAAACTGGCTGCTGCCAAAGTAGCACTCAGGCATTATTATCTAGA
TACTATATGATTAAACAGTCAATAATATTTCTTTTATTATATTATCTTTTTATTGTAAATAATCTTTATTTTTA
TTTTTAAAATGAAAAGAGAAATCATTTTTATTTTCAAAATATACGTAGAAGCTATATTTTAAGAGAATTTTT
TTTTAGTGTGTTTATATTTAAAAATATATCACTAATTGTTTTGTTTTTTTAAATAAAACTGTACAGTATGGAT
TAATGTTATGTACAGTATGGACAGCTAACTGACTGAACAATATTAAAAGACTGAATATGAAAAGGAAACCA
TGTGATGTTATTTATTGCAATTAATGTTGTTTTTATACATATTTTTATATATATGTGTTGCATTTTGCATTATT
TATGTTATTCCTTCTGATAGGATAGTATAAATATTTGAAAGGTGTCATATGTTACTTTATAATTATTCTTTTA
ATTTTATTATGTTTGTTAATGTATTGAACTGTTATTTTGCTTAAACATTACATTTATGTCATAATAATTATTG
GAAGTTTCATATATTGTTTAAATATTTTAGAAATAAATCAATTTATTGAGAGC

Protein

RF: +1

ORF: 82 -> 4941

Length: 1619 aa

>lcl|ORF1

MSDKIEDFNPREYQVELFERVMRENTIIFLPTGSGKTFIAVMLIKHKSNELKLLYSEGGKRTFFLVDTVALVAQQ
AQSIRQQAPLTVGEYSGDKGVDHWDFKKWYEELENNQVLVMTAAIFYNMILHKYVLFPRVNLIVMDECHHAV
NKHPMRLIMDQFKDVPAEDRPHVLGLTATLLNSNCKPEQISKEIHNLEITMHAKIARADNEEMVQAFSTNPKEEI
IKYDVDEVDECYQVVSTSIGEIYLILKSIVLLDNSPKPASNPPNAVLIDNKSDAINEIKNILNEITLITGELGGYAST
YALLLRIVHFERMRISSNDPTLTKLLGHIIIQLHIIRKKLDVIMKTTPNKILKYSTSKMLKLFELLKNFREDNLALVF
VERKTTAKVLYYVLDDLAAHFEEYRNIKPDFVVGVNSNPYHLTREYILERKKNQKILKKFNMGEINILVASDVIE
EGIDVKNCNYVIKFDHPKTTRSYIQSKGRARHRDSKYILFYPNSNSDSFQKKYINFKLTEETLKQELCYGRDLHR
RGGTEAEAEAFLYSKEVEPFYIDGPAGPKVTVDSAISLLNRYCFSLPCDKFTKLTVFFWKKNVIVDGVNKVICFL
QLPINCPLKGIIKGQPCENLVTAKGAVALNACKMLYEIGELNDHLLPVGNEVQNVEEKILFPLWENEDEEKSMY
NTGTKKCRRIYDKQLPNWISGGFPKPGQPVYIHVLKILPNYPKPDNDRLLAFYNLLQSKNNFAVITSKKWPKLCD
FPLFMNVGEVSINILQNSRKITLSQEQCEKIMAFHSLLFTEAWLLKKEFLVKDYENKENSYYVVPTIVDNDNIVID
WETIKNHKEIPPVKAVPLDSRGNIDVTKDRYLHKIVIPWYRPKGYMLKYVVTRVCEEQTPMSPFPSEQYSSYSD
YFFKRYNQSIINKNQKLIEVRAISGKNNCLLPRGKYRFGKKRRRDEEDFEETLVPELCTLIEFPAVYMLKVTLLPSI
LHRITMLLNAEELRQNIADGTGLGVVKLPQGVYWQPLEVDQTALIGDESSNARSTIEVSNMLADKMVELQPKD
CTWDKEEEPIDIERNLRNLDRLSVLHYERFAKIEVTLDQLNRSNGLIFGTGSSYMRSNIIRPPKVNAPDIPILSNVSS
VFGPQLSSVLKALTASSSNDVMNYERLETLGDSFLKFLVSLILFIYFKNKDEGKLSSIKGKIIGNRNLYYSGKHLN
LGKMLKVNDFIPDDWEVPGFTILESIKKVLKDAKIPPSVLYQISLSKRERDTAVLEEETRLNIQELFCNSSLADESS
YSSLGIIGQQCVADKVISDSVEALIGVYLEACGVEGAFKLCKWLSILPENMSDINKVLHEPAPTAQISDCGNMNDI
LIMPEVVESILDYRFKNRSFLLQALTHCSYRQNITDCYQRLEFLGDAILDFLITSHIYDRCKNITPGELTDLRSALV
NNVTFACLTVRYGFHKFMLTKACKLTDIIKRFVEHQEKRNHKIGAEILFLLSENDVKAAEIVDVPKVLGDLFESL
AAAVYLDSGKSLTTVWKVFYKLMHNEIEEFVGNIPKNSIRLLYEKFPVPAPCFEKPQFLDENKTTVMVTLVVTT
KNERMAFVGVGENKNQAKLAAAKVALRHYYLDTI

Conserved Domains

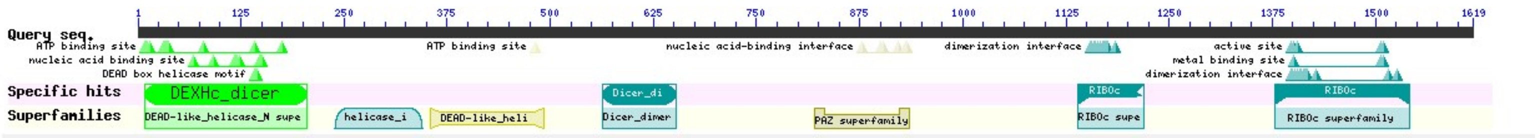

BLASTp

XP_014275310.1 endoribonuclease Dicer isoform X2 [*Halyomorpha halys*]

Score:2808 bits

E-value: 0.0

Query 5 IEDFNPREYQVELFERVMRENTIIFLPTGSGKTFIAVMLIKHKSNELKLLYSEGGKRTFF 64

IEDF PREYQVELFERVM+ NTIIFLPTGSGKTFIAVM+IKHKSN+LKLLYSEGGKRTFF

Sbjct 17 IEDFKPREYQVELFERVMKHNTIIFLPTGSGKTFIAVMVIKHKSNDLKLLYSEGGKRTFF 76

Query 65 LVDTVALVAQQAQSIRQQAPLTVGEYSGDKGVDHWDFKKWYEELENNQVLVMTAAIFYNM 124

LV+TVALVAQQAQSIRQQ PLTVGEYS +KGVDHWD +W+EELEN+QVLVMTAAIF+ M

Sbjct 77 LVNTVALVAQQAQSIRQQTPLTVGEYSAEKGVDHWDINQWHEELENHQVLVMTAAIFHQM 136

Query 125 ILHKYVLFPRVNLIVMDECHHAVNKHPMRLIMDQFKDVPAEDRPHVLGLTATLLNSNCKP 184

ILH Y+ PRVNL+V+DECHHAVNKHPM+L+M++FK VP +DRPHVLGLTATLLNSNCKP

Sbjct 137 ILHDYITLPRVNLLVIDECHHAVNKHPMKLVMEEFKGVPTKDRPHVLGLTATLLNSNCKP 196

Query 185 EQISKEIHNLEITMHAKIARADNEEMVQAFSTNPKEEIIKYDVDEVDECYQVVSTSIGEI 244

E+I EIHNLEITMHAKIARA+NEEM+QAFSTNPKEE++ Y+++E DEC+ VV +SI EI

Sbjct 197 ERIPTEIHNLEITMHAKIARAENEEMIQAFSTNPKEEVVIYNIEEDDECFAVVKSSIDEI 256

Query 245 YLILKSIVLLDNSPKPASNPPNAVLIDNKSDAINEIKNILNEITLITGELGGYASTYALL 304

Y ILK IVL+D+SPKP S+PPNA L+DNKSD I EI NIL EI LIT ELGGYASTY LL

Sbjct 257 YPILKLIVLVDSSPKPPSSPPNATLVDNKSDTIIEITNILKEIVLITRELGGYASTYVLL 316

Query 305 LRIVHFERMRISSNDPTLTKLLGHIIIQLHIIRKKLDVIMKTTPNKILKYSTSKMLKLFE 364

LRIVHFERMRIS++D L KLLGHIIIQLH+IRKKL++IMK P KI YSTSKM++LF+

Sbjct 317 LRIVHFERMRISASDQALVKLLGHIIIQLHLIRKKLELIMKNRPGKIFHYSTSKMIELFK 376

Query 365 LLKNFREDNLALVFVERKTTAKVLYYVLDDLAAHFEEYRNIKPDFVVGVNSNPYHLTREY 424

LL+NF E +LALVFVERKTTAKVLYYVLDDLAA EEY+ IKPDF+VGVNSNPYH+TREY

Sbjct 377 LLQNFEEKSLALVFVERKTTAKVLYYVLDDLAASIEEYKYIKPDFIVGVNSNPYHITREY 436

Query 425 ILERKKNQKILKKFNMGEINILVASDVIEEGIDVKNCNYVIKFDHPKTTRSYIQSKGRAR 484

ILERKKN KILKKFNMGEINILVASDVIEEGIDVKNCN+VIKFDHPKT RSYIQSKGRAR

Sbjct 437 ILERKKNHKILKKFNMGEINILVASDVIEEGIDVKNCNFVIKFDHPKTARSYIQSKGRAR 496

Query 485 HRDSKYILFYPNSNSDSFQKKYINFKLTEETLKQELCYGRDLHRRGGTEAEAEAFLYSKE 544

HRDSKYI+F P+SNS++F + YI FK +EETLKQELCYGRD RRGGTEAEAE FLYSKE

Sbjct 497 HRDSKYIVFAPSSNSNAFHRNYITFKQSEETLKQELCYGRDQSRRGGTEAEAELFLYSKE 556

Query 545 VEPFYIDGPAGPKVTVDSAISLLNRYCFSLPCDKFTKLTVFFWKKNVIVDGVNKVICFLQ 604

VEPF+IDGP GPKVTV+SAISLLNRYCF+LPCDKFT+LTVFFWKK VIVD K +CFLQ

Sbjct 557 VEPFFIDGPIGPKVTVESAISLLNRYCFTLPCDKFTRLTVFFWKKEVIVDNKPKTLCFLQ 616

Query 605 LPINCPLKGIIKGQPCENLVTAKGAVALNACKMLYEIGELNDHLLPVGNEVQNVEEKILF 664

LPINCPLKGII+GQPCENLVTAKGAVALNACKMLY+IGELN+HLLPVGNEVQNVEEKIL

Sbjct 617 LPINCPLKGIIEGQPCENLVTAKGAVALNACKMLYKIGELNEHLLPVGNEVQNVEEKILC 676

Query 665 PLWENEDEEKSMYNTGTKKCRRIYDKQLPNWISGGFPKPGQPVYIHVLKILPNYPKPDND 724

PLWE+E ++KS++ GTKK RRIYDKQ P WI GGFP+ GQPVYIHVLKILPNYPKP+ND

Sbjct 677 PLWEDEKDQKSLHKPGTKKSRRIYDKQFPRWICGGFPQVGQPVYIHVLKILPNYPKPEND 736

Query 725 RLLAFYNLLQSKNNFAVITSKKWPKLCDFPLFMNVGEVSINILQNSRKITLSQEQCEKIM 784

RLLAFYNLL S NFAV++SKKWPKLC+FPLFMNVGEV +NI QN+RKITL+QEQ +KI+

Sbjct 737 RLLAFYNLLLSNKNFAVLSSKKWPKLCNFPLFMNVGEVRVNIQQNARKITLNQEQYKKIL 796

Query 785 AFHSLLFTEAWLLKKEFLVKDYENKENSYYVVPTIVDNDNIVIDWETIKNHKEIPPVKAV 844

AFHSLLFTEAW LKKEFLV++YENKENSYYVVP IVDND+I IDWETIKNH EIPPVKAV

Sbjct 797 AFHSLLFTEAWSLKKEFLVREYENKENSYYVVPAIVDNDSITIDWETIKNHGEIPPVKAV 856

Query 845 PLDSRGNIDVTKDRYLHKIVIPWYRPKGYMLKYVVTRVCEEQTPMSPFPSEQYSSYSDYF 904

PLDSRG+I VTKD+YL+KIVIPWYRPKG+MLKYVVTRVCEEQTPMSPFPSEQYSSYSDYF

Sbjct 857 PLDSRGDIIVTKDKYLNKIVIPWYRPKGFMLKYVVTRVCEEQTPMSPFPSEQYSSYSDYF 916

Query 905 FKRYNQSIINKNQKLIEVRAISGKNNCLLPRGKYRFGKKRRRDEEDFEETLVPELCTLIE 964

FKRYNQS++NKNQKLIEVRAISGKNNCLLPRGKYR GKKRRRDEEDFEETLVPELCTL+E

Sbjct 917 FKRYNQSVVNKNQKLIEVRAISGKNNCLLPRGKYRLGKKRRRDEEDFEETLVPELCTLLE 976

Query 965 FPAVYMLKVTLLPSILHRITMLLNAEELRQNIADGTGLGVVKLPQGVYWQPLEVDQTALI 1024

FPAVYMLK+TLLPSILHRI +LLNAEELRQ IA TGLGVVKLP GVYW LEVD TAL

Sbjct 977   FPAVYMLKITLLPSILHRINILLNAEELRQKIAAETGLGVVKLPSGVYWSSLEVDDTALA 1036

Query 1025 GDESSNARSTIEVSNMLADKMVELQPKDCTWDKEEEPIDIERNLRNLDRLSVLHYERFAK 1084

GDESSNARSTIEVSNMLADKMVELQPKDCTWDKEEEPIDIERNLRNLD LSVLHYE+FAK

Sbjct 1037 GDESSNARSTIEVSNMLADKMVELQPKDCTWDKEEEPIDIERNLRNLDLLSVLHYEKFAK 1096

Query 1085 IEVTLDQLNRSNGLIFGTGSSYMRSNIIRPPKVNAPDIPILSNVSSVFGPQLSSVLKALT 1144

IE++ DQ++RSNG++   GSS +SNI+RPPKVNAP+IPILS+ + V GP+L S+LKALT

Sbjct 1097 IEISADQIDRSNGMVSERGSSVKKSNIMRPPKVNAPEIPILSDKNCVLGPELGSILKALT 1156

Query 1145 ASSSNDVMNYERLETLGDSFLKFLVSLILFIYFKNKDEGKLSSIKGKIIGNRNLYYSGKH 1204

ASSSNDVMNYERLETLGDSFLKFLVSLILF++F+NKDEGKLSSIKGKIIGNRNLYYSGK+

Sbjct 1157 ASSSNDVMNYERLETLGDSFLKFLVSLILFVHFENKDEGKLSSIKGKIIGNRNLYYSGKY 1216

Query 1205 LNLGKMLKVNDFIPDDWEVPGFTILESIKKVLKDAKIPPSVLYQISLSKRERDTAVLEEE 1264

L+LGK+LKVNDFIPDDWEVPGFTILESIKKV+ +AKIPPSVLYQISL+K+ERDTAVL EE

Sbjct 1217 LSLGKILKVNDFIPDDWEVPGFTILESIKKVIINAKIPPSVLYQISLTKKERDTAVLNEE 1276

Query 1265 TRLNIQELFCNSSLADESSYSSLGIIGQQCVADKVISDSVEALIGVYLEACGVEGAFKLC 1324

TR I ELFCNSS ADES++SSLG+IGQQCV DKVI+D+VEALIGVYLEACG+EGAFKLC

Sbjct 1277 TRSKIHELFCNSSPADESTHSSLGVIGQQCVVDKVIADAVEALIGVYLEACGIEGAFKLC 1336

Query 1325 KWLSILPENMSDINKVLHEPAPTAQISDCGNMNDILIMPEVVESILDYRFKNRSFLLQAL 1384

KWL ILPENM DI+KVL+EPAP+AQI D G++ND+L+MPEV+ESIL YRF+NRS+LLQAL

Sbjct 1337 KWLKILPENMCDIDKVLYEPAPSAQILDSGDVNDVLVMPEVIESILGYRFRNRSYLLQAL 1396

Query 1385 THCSYRQNITDCYQRLEFLGDAILDFLITSHIYDRCKNITPGELTDLRSALVNNVTFACL 1444

THCS+RQN TDCYQRLEFLGDAILDFLITSHIY++CKNI+PGELTDLRSALVNNVTFACL

Sbjct 1397 THCSFRQNFTDCYQRLEFLGDAILDFLITSHIYNKCKNISPGELTDLRSALVNNVTFACL 1456

Query 1445 TVRYGFHKFMLTKACKLTDIIKRFVEHQEKRNHKIGAEILFLLSENDVKAAEIVDVPKVL 1504

TVRYGFHKFMLTK CKLTDIIKRFVEHQEKRNHKIGAEILFLLSENDV+AAEIVDVPKVL

Sbjct 1457 TVRYGFHKFMLTKTCKLTDIIKRFVEHQEKRNHKIGAEILFLLSENDVEAAEIVDVPKVL 1516

Query 1505 GDLFESLAAAVYLDSGKSLTTVWKVFYKLMHNEIEEFVGNIPKNSIRLLYEKFPVPAPCF 1564

GDLFESLAAAVYLDSGKSL TVWK+FYKLMHNEIEEF+ NIPKNSIRLLYEKFP+PAPCF

Sbjct 1517 GDLFESLAAAVYLDSGKSLNTVWKIFYKLMHNEIEEFMRNIPKNSIRLLYEKFPIPAPCF 1576

Query 1565 EKPQFLDENKTTVMVTLVVTTKNERMAFVGVGENKNQAKLAAAKVALRHYYLD 1617

EKP+FL+EN+T VMV L+VT KN+RM F+GVGENKNQAKLAAAKVALRHYYLD

Sbjct 1577 EKPRFLNENQTIVMVPLIVTAKNDRMRFIGVGENKNQAKLAAAKVALRHYYLD 1629

Argonaute-2 (Ago-2)

>TRINITY_DN2417_c0_g1_i1 len=4003 path=[0:0-22 2:23-79 3:80-113 4:114-170 5:171-227 6:228-250 7:251-274 9:275-325 10:326-4002]

CCTCAACAGCAACAACCACCCCAGCAGCAGCAAAGACCTCAACAGCAACAACCACCTCAGCAGCAGCAAA
GGCCTCAACAGCAGCAGTGGCCTCAACAACAGCAGCAACCACCCCAGCAGCAGCAAAGGCCTCAACAGCA
GCAGTGGTCTCAACAACAGCAGCAACCACCCCAGCAGCAGCAAAGGCCTCAACAGCAGCAGTGGCCTCAA
CAACAGCAGCAACCACCCCAGCAGCAGCAAAGGCCTCAACAGCAGCAGTGGTCTCAACAGCAGCAGCAAC
AGAGAACACAAGATGTTTCTGAAAATTTCCCACCACCTGGAGCAGGTGATTTTGGCTCTAAAAAGAACAAA
AAAAAGGGTGGAAGGCAAGGACAAACTCTTCCTCCACAGGATTCTGGACCAGGGCCAAGTGCTGCACCTC
CTCAGCAGGCTTTACAACATAGTCAGCCTACACCTCCACAGCAATTTCCCCAAGAACCAAGGATGCAGCAA
CCACCAGGGTTTGTAACCGGCCCTGTAAGACAAACAGCGCCCCCCGGAATGGTACGGCAGAGACCTCCATC
ACAGACATGGGCCCAGCAGCAACAAGCCGGTCCACCTGGTCCCAGAGGGCAGCCTTCTCCTTGGGCACAA
AGAGGACCTCCTCCTCAACAGGTTTCTCAACCACCGTCTAGAGAAACTACCCCTGTTCAACAACAGCCGGT
ATCATCACCCGTGAGCAGCGCACCTCCTGCTAGGCAGCAACCCCACCCCGCTGACCAGCAGCAGGTCAGGC
AACAGCCTTCCCAGCCTCCTGTACAAACCCAAGTGCCGGCTCAGCCAAGATCTGCCCCAGGAGGAAGTGCT
TTAGTAGAAAAAATGAAAGCTTTATCTACTAAGTCAACGTTTATTCCTCCCAAAAGGAAAGATAGACCCGC
TGACCCTAAAAAGGGAAGAGAAATAATTGTCGAAACTAATCATCTTAATTTAAACATTAAGAATAAAAATA
TGGTAATTCATCATTATGATGTGTCCATTACCCCCGAGAAACCGTATCGCAATTATAGACAAGCTGTGGAA
GCTGTGCGGCAGAGATGTTTCAAAGATCGATTCCCTGCCTTTGATGGAAAGAAGAACCTATACAGTTATCC
TGAGTTGCCTATTCTAAAATCGGAACTTGAGGAGACTGTGACAATCTATGATAATGAGAGGGACCAAGAAA
AGGAAATGACGGTTACCATTAAGTACGCCACCCAAGTCAATGTCTCAATGATATGGGACTATCTCAACTAC
GGAACTTCAACTAATGGTCCACAGGAAGCAATTCAAGCTCTTGACATTGTCTTAAGGCAACCAGCTGCCAA
CAGGTTTGTGAATGTTGGAAGATCATTTTTCTCACCTCCACCTGGAAGAGTAATTGATCTCGGTTTTGGTCT
AGATCTTTGGTATGGTTTCTTCCAGTCAGCAATTATTGGTTGGAAACCATACCTTAACATTGATGTGGCTCA
CAAAGGGTTTCCCGCTGGTGACAATTGCATTGTTGCTCTTGGAAAATTTATCCGTGGAGATATTAACGATCC
ACGCTTGCAACTGAAACCTTATGAACTTGAGACTTTTGTTGCATACATTAAGGATTTAAAAGTTGTTTATGA
AATAAAAAATAAAGGTGTTAAAAGAACATATAAAGTAACTTCTACCACAACTTGCCCTAGAGATAACAGG
TTTGACATGATTGATAAAGACACCAATATTAAAACTGAAACGACTGTTGAAAATTATTTTTTCACCCAGTAT
GGTGTTAGGTTGAGATATCCTCATCTCCCGTGCCTGGTCGTTGGTTCACGTGAGAAGCCTTTGTCCCTTCCA
ATGGAGTTGTGTACAATTGTACCGGGTCAAGTTACAATGAAGAAAATGAATGATACTCAGACTCGTACGAT
GGTAAAAGAAGCTGCTGTTGATACTGAAAGGAGGAAACAAAAGATTGTTGAATCTATAAGCAAGATCAAG

TTTAATAGTGACCCTTGCTTGAGGGAGTTTGGGCTTTCAGTTGATGAAAATTTCACTAAAGTGAAGGCTAGA
ATAATGAATGCACCAACTGTCAAATATCGAGAACAACCGGTTACTGTTCGACAAGGCACATGGAGGAGTG
AAAAGTTTGTTCAAGGTGCTGAGTTGATCCAATGGAGAATTGTAAATACTAATCGAATGGTCAGAGATGGA
GATTTAAGAAAACTCGCAACAGATCTGATACAGCATGGAAAGGATTGCGGCATGAATATATCTAATAATTA
TAGAATTGTCTATAAGGACAGTCCTCGTGACCTTGAGCAATATTTTGGTCAATGTCTTCAAGAAGATGTCAA
GCTTGTAATTGTTATTTTACCCGATAGAGGAATGACATATGCTTCCATTAAAAAGAGTGCTGAACTGCAAGT
TGGTGTACTTACACAATGCCTGAAATCGATCACAGTACAGAGGAGGCTTAATGCTGCGACATTCATTAATA
TATTACAGAAAATAAATGCAAAGCTGAATGGTGTCAACCATCACATCACTGATAGTTTTTGGCCAAAAATA
TTCCAAGATCCTACCATTATTGTTGGTGCTGATGTCACTCATCCGGCTCCTGACCAGATCAATGTTCCTTCCA
TCGCAGCCGTGGCAGCTTCTTATGACCCGAAGGCCTTCAGGTACAACATGATATGGAAGCTTCAGGCGCCG
AGGGAGGAAGTGATCAGGGACCTTGAGAGCATCATGAAGGAACAATTACTCATGTTTTATAGGAATACCA
GGCAGAAGCCTCAGACTATCTTCTTTTATAGGGATGGAGTGTCAGAAGGACAGTTTAAGATAATCTTGGAT
CAAGAATTGATGGCAATCAGGAGGGCTTGCCAGAGCCTTAGCCAAGATTACAAACCTCCGATTACATTTAT
TGTCGTACAGAAACGACATCATACAAGATTCTTTCCTGACAGGAGGGACGCAGATGGTCGTAATTTCAACG
TGCCAGCTGGTACAGTCGTTGACACCGAGATCACTCACCCGACTGAATTAGACTTCTATTTAGTTAGTCATG
CTAGTATACAGGGTACAGCGCGACCCACGAAGTATCACCTGCTATGGGATGATTCTAATCTATCAGAGCAG
ACTTTAGAAGAGATAACTTACTATCTTTGTCATCTCTTCACCAGATGCACCCGTTCAGTCTCCTACCCCGCA
CCTACTTATTATGCTCACCTCGCCGCATTCCGTGCTAGGGCATATACAGATGCAGATAGGCTGCAACTGAAC
AAACTAAAAGATGAACAGGTTAAAAGGACGGTGAAGGATTCGGTCTGTAAAAATAATCCAATGTACTATG
TTTAAAAACAAAAGTTTGAAATGGTGTCCTAATACATACTAACTTTTATCATGATATATCTTTATAGAGTTT
TATATATATTTTAAAAAAGTCTGTTCTAATTATTTTTTGTAACATTGTATATATTTCAACTTTCTGGTGTTGA
AGAAATATCAATGTGATATTAAACTTAAGAAGTTTTGTATTATTATTATTTTATTTTATTATTATTAATGTAG
AAGTACCTACTTCATTTATGTATATAAATTTATTCTATTTGGAAAAAAAAAAAGTTGATATATGGAAGTAGAA
GAATTGCCTTTATCTGTTATGCAAAAATATATGTAATAAATCTTAGAACAATCATTATGTAACTAGTAGTTG
GTTGTATAACTTGAAATGCTGGTTTTATTGATTAATTGATTTTAGTTTATTTTTTTAAACGTTTTATCAGTTTT
TATATCCCTGTAAGGAAAGGAATCTTACATGGCACAGACATTTTTAATTTTTTCATAAAGTATTCCAAGCTC
ATAGAAAAAAAGAAAGAAGGTAATTAATTATCTATTAATTAAAAAATTAATAATTTCTATTTTGTACAAAG
TATGCATATTTTATTTATTTTACATCTTAACCCACATTTTATGTTATACAACCAGATATGGGTTCTATCAACA
GTTAAAATGCC

Protein

RF: +1

ORF: 484 -> 3345

Length: 953 aa

>lcl|ORF1_TRINITY_DN2417_c0_g1_i1:483:3344 unnamed protein product

MQQPPGFVTGPVRQTAPPGMVRQRPPSQTWAQQQQAGPPGPRGQPSPWAQRGPPPQQVSQPPSRETTPVQQQP
VSSPVSSAPPARQQPHPADQQQVRQQPSQPPVQTQVPAQPRSAPGGSALVEKMKALSTKSTFIPPKRKDRPADPK
KGREIIVETNHLNLNIKNKNMVIHHYDVSITPEKPYRNYRQAVEAVRQRCFKDRFPAFDGKKNLYSYPELPILKS
ELEETVTIYDNERDQEKEMTVTIKYATQVNVSMIWDYLNYGTSTNGPQEAIQALDIVLRQPAANRFVNVGRSFF
SPPPGRVIDLGFGLDLWYGFFQSAIIGWKPYLNIDVAHKGFPAGDNCIVALGKFIRGDINDPRLQLKPYELETFVA
YIKDLKVVYEIKNKGVKRTYKVTSTTTCPRDNRFDMIDKDTNIKTETTVENYFFTQYGVRLRYPHLPCLVVGSR
EKPLSLPMELCTIVPGQVTMKKMNDTQTRTMVKEAAVDTERRKQKIVESISKIKFNSDPCLREFGLSVDENFTKV
KARIMNAPTVKYREQPVTVRQGTWRSEKFVQGAELIQWRIVNTNRMVRDGDLRKLATDLIQHGKDCGMNISN
NYRIVYKDSPRDLEQYFGQCLQEDVKLVIVILPDRGMTYASIKKSAELQVGVLTQCLKSITVQRRLNAATFINILQ
KINAKLNGVNHHITDSFWPKIFQDPTIIVGADVTHPAPDQINVPSIAAVAASYDPKAFRYNMIWKLQAPREEVIR

DLESIMKEQLLMFYRNTRQKPQTIFFYRDGVSEGQFKIILDQELMAIRRACQSLSQDYKPPITFIVVQKRHHTRFF
PDRRDADGRNFNVPAGTVVDTEITHPTELDFYLVSHASIQGTARPTKYHLLWDDSNLSEQTLEEITYYLCHLFTR
CTRSVSYPAPTYYAHLAAFRARAYTDADRLQLNKLKDEQVKRTVKDSVCKNNPMYYV

Conserved Domains

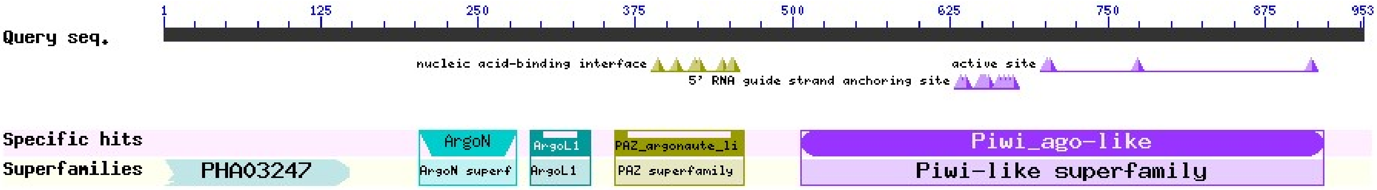

BLASTp

AVK59468.1Argonaute 2-PC [Nezara viridula]

Score:1585 bits

E-value: 0.0

```
Query  85   VRQAAPITRPQAMEQQGPPRQPWGQQQQQQQQQQQQARFPLQTGPRGQ-TPWAQRGPPPQ  143
            VRQ AP     M +Q PP Q W QQQQ         A  P   GPRGQ +PWAQRGPPPQ
Sbjct  12   VRQTAP----PGMVRQRPPSQTWAQQQQ--------AGPP---GPRGQPSPWAQRGPPPQ  56

Query  144  QVSQPQSRETTPTHQTQLPPQQQPVS-PVTSAPPVRQQP--SDQQPSRPPSSQPQVQQQA  200
            QVSQP SRETTP       QQQPVS PV+SAPP RQQP  +DQQ  R   SQP VQ Q
Sbjct  57   QVSQPPSRETTPV-------QQQPVSSPVSSAPPARQQPHPADQQQVRQQPSQPPVQTQV  109

Query  201  -AKPRSPAEGSALAEKMRNIDIKSSSYIPPKRGDRPSDPKKGREIVVETNHLNLNIKNKN  259
             A+PRS   GSAL EKM+ +  KS+ +IPPKR DRP+DPKKGREI+VETNHLNLNIKNKN
Sbjct  110  PAQPRSAPGGSALVEKMKALSTKST-FIPPKRKDRPADPKKGREIIVETNHLNLNIKNKN  168

Query  260  MVIHHYDVSINPEKPYRNYRQAVEAVRQRCFKNRFPAFDGKKNLYSYPELPLQKTELVET  319
            MVIHHYDVSI PEKPYRNYRQAVEAVRQRCFK+RFPAFDGKKNLYSYPELP+ K+EL ET
Sbjct  169  MVIHHYDVSITPEKPYRNYRQAVEAVRQRCFKDRFPAFDGKKNLYSYPELPILKSELEET  228

Query  320  VTIYDNERDQEKEMTVTIKYATQVNVSMIWDYLRAGTSTNGPQEAIQALDIVLRQPAANR  379
            VTIYDNERDQEKEMTVTIKYATQVNVSMIWDYL  GTSTNGPQEAIQALDIVLRQPAANR
Sbjct  229  VTIYDNERDQEKEMTVTIKYATQVNVSMIWDYLNYGTSTNGPQEAIQALDIVLRQPAANR  288

Query  380  FVTVGRSFFSPPPGRVIDLGYGLDLWYGFFQSAIIGWKPFLNIDVAHKGFPAADNCVVAL  439
            FV VGRSFFSPPPGRVIDLG+GLDLWYGFFQSAIIGWKP+LNIDVAHKGFPA DNC+VAL
Sbjct  289  FVNVGRSFFSPPPGRVIDLGFGLDLWYGFFQSAIIGWKPYLNIDVAHKGFPAGDNCIVAL  348
```

Query 440 GKFVQGDLGDPRFQMRPYERESFVAYIKDLKVVYEIPSKSVKRTYKVTNITTCPRDNRFE 499

GKF++GD+ DPR Q++PYE E+FVAYIKDLKVVYEI +K VKRTYKVT+ TTCPRDNRF+

Sbjct 349 GKFIRGDINDPRLQLKPYELETFVAYIKDLKVVYEIKNKGVKRTYKVTSTTTCPRDNRFD 408

Query 500 ITDKETNNKSETTVERYFVSQYNIRLKYPHLPCLTVGSREKPLALPMELCTIVAGQVTMK 559

+ DK+TN K+ETTVE YF +QY +RL+YPHLPCL VGSREKPL+LPMELCTIV GQVTMK

Sbjct 409 MIDKDTNIKTETTVENYFFTQYGVRLRYPHLPCLVVGSREKPLSLPMELCTIVPGQVTMK 468

Query 560 KMNDAQTRTMVKEAAVDTERRKEKIVKSIRQINFNSDPCLKEFGLSVDDQFTKVKARIMN 619

KMND QTRTMVKEAAVDTERRK+KIV+SI +I FNSDPCL+EFGLSVD+ FTKVKARIMN

Sbjct 469 KMNDTQTRTMVKEAAVDTERRKQKIVESISKIKFNSDPCLREFGLSVDENFTKVKARIMN 528

Query 620 APTLDYRERPVPVRQGVWRNEKFIQGADLVQWRIVTTNQMIREPDLRKLASGLINHGMEC 679

APT+ YRE+PV VRQG WR+EKF+QGA+L+QWRIV TN+M+R+ DLRKLA+ LI HG +C

Sbjct 529 APTVKYREQPVTVRQGTWRSEKFVQGAELIQWRIVNTNRMVRDGDLRKLATDLIQHGKDC 588

Query 680 GMNISNNYKIVSKNTPRELEQYFSQCLKEEVKLVIVVLPDRGMTYASIKKIAEIQVGILT 739

GMNISNNY+IV K++PR+LEQYF QCL+E+VKLVIV+LPDRGMTYASIKK AE+QVG+LT

Sbjct 589 GMNISNNYRIVYKDSPRDLEQYFGQCLQEDVKLVIVILPDRGMTYASIKKSAELQVGVLT 648

Query 740 QCLKTVTVQRRLNAATFVNILQKVNAKLNGINHHITHSYWPKFFQKPVIVVGADVTHPAP 799

QCLK++TVQRRLNAATF+NILQK+NAKLNG+NHHIT S+WPK FQ P I+VGADVTHPAP

Sbjct 649 QCLKSITVQRRLNAATFINILQKINAKLNGVNHHITDSFWPKIFQDPTIIVGADVTHPAP 708

Query 800 DQINVPSIAAVAASHDPKAFRYNMIWKLQPPREEVIRDLESIMKEQLLAFYRSTRYKPQA 859

DQINVPSIAAVAAS+DPKAFRYNMIWKLQ PREEVIRDLESIMKEQLL FYR+TR KPQ

Sbjct 709 DQINVPSIAAVAASYDPKAFRYNMIWKLQAPREEVIRDLESIMKEQLLMFYRNTRQKPQT 768

Query 860 ILFYRDGVSEGQFKMILNQELQAIRRACSSLSSDYTPPITFIVVQKRHHTRFFPEKKDAD 919

I FYRDGVSEGQFK+IL+QEL AIRRAC SLS DY PPITFIVVQKRHHTRFFP+++DAD

Sbjct 769 IFFYRDGVSEGQFKIILDQELMAIRRACQSLSQDYKPPITFIVVQKRHHTRFFPDRRDAD 828

Query 920 GRNMNVPAGTVVDTEITHPTELDFYLVSHASIQGTARPTKYHLLWDDSNLSEQNLEEITY 979

GRN NVPAGTVVDTEITHPTELDFYLVSHASIQGTARPTKYHLLWDDSNLSEQ LEEITY

Sbjct 829 GRNFNVPAGTVVDTEITHPTELDFYLVSHASIQGTARPTKYHLLWDDSNLSEQTLEEITY 888

Query 980 YLCHLFTRCTRSVSYPAPTYYAHLAAFRARAYTDADRLQLNQLQEEQVRRTVKDSVCKNN 1039

YLCHLFTRCTRSVSYPAPTYYAHLAAFRARAYTDADRLQLN+L++EQV+RTVKDSVCKNN

Sbjct 889 YLCHLFTRCTRSVSYPAPTYYAHLAAFRARAYTDADRLQLNKLKDEQVKRTVKDSVCKNN 948

Query 1040 PMFFV 1044

PM++V

Sbjct 949 PMYYV 953

R2D2

>TRINITY_DN2682_c1_g1_i1 len=1789 path=[1:0-151 2:152-234 4:235-236 5:237-1298 6:1299-1299 7:1300-1788]

AAACAAACAACTGTATATCATGGATTCCTATTTGGAATCACTTGTTATGCTGTGGGAAGGTTATGTTGCTGT
TTACTGTTGTCGTTATTCTCTTAACTTCAAATGTAGTGAATTGCTGAAGTGCCTGTTTTCTTACCAATACTAG
ATATTGGGGTTTAGTGAACCAATTGTGTTATTTTTATAATGATATATAGTAATTATATAAACGTGTAACTAA
GAATTTGTTTTGTTTTTTAGTATTTAATTATTATGGATGGAAAAACTCCTGTCACCTTACTCCATGAGTATTT
AATGAAAGGTGGAGAAGTTCCTGATTACAAGTTAGTGTACAATGGAGTAGGAACTCATGATCCTCTGTTCC
AGTATGAAGTTAGTGCCAAAGGTATGGAGGCCATAGGGAAGGGAAAATCAAAAAAAGAAGCCAAACATG
ATGCAGCTCGTGCCCTTCTTCTAAAGCTGAAAGATAAACATGCCTTGGAAGAGGAGGTTGAAGTTGTTTCA
CCTTACGAACATTCTCTCAAAGAAAATGCAGTAGGACAACTTCAAGATTTTTGTTTACAGCACAACACTGCT
TTCCCTACTTATGAATTAATCAGAGACGAAGGATTAGCTCATGCCAAAGTTTTTGGCATACGCTGTAGGGTT
TCTTCATTTTGCACCGAAGCTGAGGCTCGGACTAAGAAACAAGCAAAGCAACAAGCTTCACATTTAATGTT
ATTAAAGCTGGAAAAATGTCTTAGCGAAGGAAACTTTGAGGTGAACATTGCTAAACCAGATTCGGAAAAA
CCAGAAGCAGACGAATTACTTGAAAAGGCTAGTGAGAAGACCAAGGAAGCCTATTCTAAAGTTTTACTGG
AAAGGTCAAATGAAAAAGATGAAATTGCTAGTTGTAGTCTGGGAACTTCAATGAGTAATTTTTCTGATCAC
TTTTTAGAAAATGTTTTGCCTTTTTCCGAAACTCTTAAAAGCATTGCTGATAACAATAATGAATACTTCGAG
AACCATCCTGATCCAGAAGAATTGGTTGCAACGATAATGACCCAATTAGATTACCATCACAGTTATGAATT
TCTACCAACTAAGAAGGACACTGATAATTTGTGCTTTTTACAGATTAGAGAGTTGAACTGTGCCAACTTTGT
TGGACTAAAGGAAAGTAGAGACCTTGCTAAGAAAACTGCAGCTATTAAAGCTCTTCAATTTTTATCATGCA
TGTCAAAACCATGATTTTACACACTGTTTTTTTTTTTTTTTTTTTTTAATTTTTTGAGAGAAATGTATATCAG
TTTTTGTTCTTTTGCAAATTTTACTTATTCTTCTTCATTTTGATTATTTTGTAAAACGATTTTTCTCTCTTGAAA
TACAATTCTTTGTTATTTGTCTAGATCAATTTTTGAGACAAACACAAATAACTAGTTTGTGATTGTCTTAAAC
CGATAGTTGCAGGATTGTATCGATGTTGATAAATTTATTAGTCAATGTAACATGAGTATGATGATATGTGAT
GAGTTATAGAAATTAATTTTATTGAATATGGTTTTATTTTAACTTTGTATGTAAATATGTTGTATATTATGTT
ATCTTTGTTATTAATAAATTACTAATGATTTTTTAAAAAATATTTTTAACTTTTTTATAAAGTTATATAATTTC
AGAAACAATCCTCTTCTATTTATTAAATTATAACATTCTGCCAACTTTGTTTGTATTAAAAAAAATACCACTT
TTTTTTCAAAATATTGTTAAATATTTTTTTTAATAAAGAGTCGTTATACCGTTAAATTGTCA

Protein

RF: +1

ORF: 250 -> 1227

Length: 325 aa

>lcl|ORF1_TRINITY_DN2682_c1_g1_i1:249:1226 unnamed protein product

MDGKTPVTLLHEYLMKGGEVPDYKLVYNGVGTHDPLFQYEVSAKGMEAIGKGKSKKEAKHDAARALLLKLK
DKHALEEEVEVVSPYEHSLKENAVGQLQDFCLQHNTAFPTYELIRDEGLAHAKVFGIRCRVSSFCTEAEARTKK
QAKQQASHLMLLKLEKCLSEGNFEVNIAKPDSEKPEADELLEKASEKTKEAYSKVLLERSNEKDEIASCSLGTS
MSNFSDHFLENVLPFSETLKSIADNNNEYFENHPDPEELVATIMTQLDYHHSYEFLPTKKDTDNLCFLQIRELNC
ANFVGLKESRDLAKKTAAIKALQFLSCMSKP

Conserved Domains

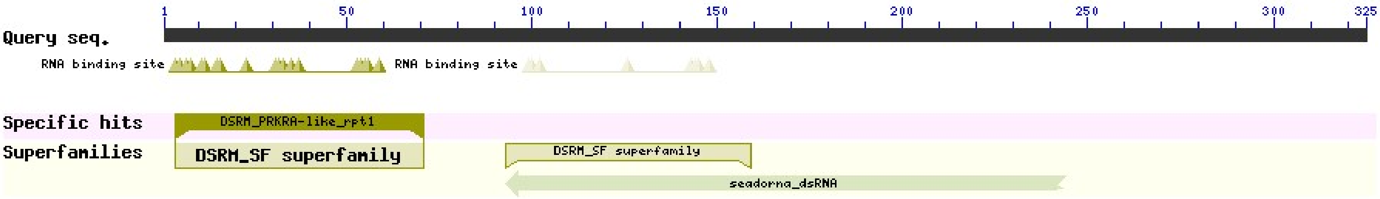

BLASTp

XP_014288218.1 interferon-inducible double-stranded RNA-dependent protein kinase activator A-like isoform X1 [*Halyomorpha halys*].

Score:582 bits

E-value: 0.0

Query 10 MDGKTPVTLLHEYLMKGGEVPDYKLVYNGVGTHDPLFQYEVSAKGMEAVGKGKSKKEAKH 69

MDGKTPVTLLHEYLMKGGEVPDYKLVYNGVGTHDPLFQYEVSAKGMEA+GKGKSKKEAKH

Sbjct 1 MDGKTPVTLLHEYLMKGGEVPDYKLVYNGVGTHDPLFQYEVSAKGMEAIGKGKSKKEAKH 60

Query 70 DAARALLLKLKDEKALEQEVEVVSPYEHSLKENAVGQLQDFCSQHSTALPRYELIRDEGL 129

DAARALLLKLKD+ ALE+EVEVVSPYEHSLKENAVGQLQDFC QH+TA P YELIRDEGL

Sbjct 61 DAARALLLKLKDKHALEEEVEVVSPYEHSLKENAVGQLQDFCLQHNTAFPTYELIRDEGL 120

Query 130 AHAKVFGIRCRVSSFITEAEARTKKQAKQQASHLMLLKLEKCLSEGDF-ISTSETEKQQQ 188

AHAKVFGIRCRVSSF TEAEARTKKQAKQQASHLMLLKLEKCLSEG+F ++ ++ + ++

Sbjct 121 AHAKVFGIRCRVSSFCTEAEARTKKQAKQQASHLMLLKLEKCLSEGNFEVNIAKPDSEKP 180

Query 189 ETNGLTQKACDETKEAYQQVVQERTNEKDKISCCRLGTSMSNFSGQFSDNVLPLSETLRT 248

E + L +KA ++TKEAY +V+ ER+NEKD+I+ C LGTSMSNFS F +NVLP SETL++

Sbjct 181 EADELLEKASEKTKEAYSKVLLERSNEKDEIASCSLGTSMSNFSDHFLENVLPFSETLKS 240

Query 249 IADKDDCFFENLEDPEELLITIMTQLDYHYSYDFLQTMTETDHLCFLQIRELNCANFVGL 308

IAD ++ +FEN DPEEL+ TIMTQLDYH+SY+FL T +TD+LCFLQIRELNCANFVGL

Sbjct 241 IADNNNEYFENHPDPEELVATIMTQLDYHHSYEFLPTKKDTDNLCFLQIRELNCANFVGL 300

Query 309 STSKIIAQKNASVKALQFLSYMSR 332

S+ +A+K A++KALQFLS MS+

Sbjct 301 KESRDLAKKTAAIKALQFLSCMSK 324

piRNA

Argonaute-3 (Ago3)

>TRINITY_DN83747_c0_g1_i1 len=2934 path=[0:0-2933]

CCGGATTCTTATCCTGGTGATTCGTTTTTCAGTTAGCTTTGCAGATTAATAACATGTCTTTAACTTGAATAT
TTTTTGGGTTGTCGTTCGACGGAACACAATAGCTCCTCCTTAAACTGATTTTTCTGCCTTAAAAATGGAGGG
TAGACGTCTTGGGAGAGGAGAAGCTTTAAGAAAGCTTCTCGAATCCCAACCCCAGTCTTTGCCTCATGCTC
AATCATTCTCCCAGCCAGAGCCATCATCTCAACGCTCACCCCCAACACATGAACAAGATCCATATTCTTATG
GCCAGGATGTAGCAGCTCATACATCAGGTCCAGTATTGAGAAAACCAATGGGCAGAAGTGCATTGCTGGC
GAGTGAGCTGAGGAGAAGTGAAGAATCAAGAGAGGAGGCGGGAGAAGCCCCGAAACCTACGGGAAGAGG
AAGAGCCCTGTTGGCTTCCTTGGCTGCCAGGGAAGTGACACCCGAAGTCGAACAAATCTCTCGACAGTTGT
CTAGAACTTCATTGACTGAATCCTCTGAAGCAGATAAGGAACCTGTTGTCATGAAAGGAACATTTGGAAGG
GGAATACCTATAGGTGTGAACTACGTCAAGCTAAAAGTAGAAAAGGGCAAAGGTCTGTTTCAGTATGATGT
TCAATATGATCCGCCAGTAGAATCAAGGAGTCTCAAGTTCGGGATATTGAATAATGTCAAAAATGTGATCG
GAGAGACCAGGCTCTTTGACGGAATGGTTCTTTATTTACCAATTAAATTACCTCAGAAGGTTACGATTGTAA
AAACAACTGTAGCTCATGATAATAGTGTTGTTACCGTTAGAATTACATTTACAAAAGAAAGGAAATTAGGC
GATCCAGAAGCGATTCACCTCTACAACGTGTTGTTTAGAAGGATTATGGTTATTTTAGGTTTAGCCCTTCAT
GGAAAAAATTTTTATGATCCGAAATCAGCCATGCCTATTCCATTACATAAGTTGGAAGTATGGCCTGGCTA
CATCACCGCCATTGATGAATTTGAAGGAGGCATTATGTTATGCTGTGATGCTTCACACAGAGTTTTAAGAAA
TCAAACTGTGCTTCAGTTAATGGAGGATATGCAGAACTCAAACCATAAGCACTGGAAAGATGAATTCGTTC
AGTTAATACTAGGGCAGTCTGTACTTACGAAGTATAACAACAAGGTTTATAGAATTGACGATTTATCGTTCG
ATGAATGTCCAAATGATTGTTTCGAAAAAAGCAACGGCGAAAGAGTTAAATATGTCGACTATTACAGGCTT
CAGTATAACGTAAGAGTTACAGATCTCAATCAACCGCTTCTTAAAAGTAGAGTAAAAACACGTGTTAAAGG
AAAAGAAGAAAGACAGCTGGTTTCTCTTGTTCCTGAACTGTGCTTCTTAACAGGAATAACGGACACAATGA
GAGCCGATAATAGGGTTATGAAAGATATCACTCAGTGCATCAGTATTTCACCTAACCAAAGGCACTATGCC
ATAAACCAATTTGTCAACAACATTAAAGGTTCTCCCGAGGCAACCAAAGTTCTTTCTGACTGGGGTATCTCG
CTGGATCACGCTTATATGATTCTTGAGGGTCGTAATATACCTCCTATTGATATAATATTTGGTGGAGGAGTA
ACGGTACCAGGTAACCGAGAAGCTAATTGGTCTGGTGCGTCTAATAAACACAAAGCATTATCTGTCATTGA
TTTTTCCTCTTGGTCGGTGGTCTGTACAAGAAGAGATATGGATATGGTTTCATCATTCGTGGACCAGATGAT
AAAAATAGGACCTCAGATGGGTATTAAAATCATGAAACCGAGAATGGTTCCCATTCCAGACGACAGAACA
GATAATTACATCAGGTCTCTAAGGTCGTCGATCAGGGAGAAAGTACAGATTGTTGTTATCATCTTTCCATTG
GCCAGAACTGACAAATATAGTGCTGTTAAGAAGCTGTGCTGCATTGAAGAGCCTGTTCCTTCTCAGGTAAT
TCTGTCGAGGACAGTCAGAAGAGCCGGTACTTCTAAAGCTATCACTTCAAAAATTGCTCTACAAATGAATT
GTAAACTCGGTGGAACGCTTTGGGCAGTAAATGTACCTTTGAAATACACAATGGTATGCGGATTGGATACC
TATCACGACCCCAAGAGGAGAGCGAACAGCGTAGGAGCTCTGGTCAGCTCGTTGAACCAGCCGCTTACGC
GATGGTATTCCAAAATTTACAGTCAGGCTGCAGGTCTAGAGTTTGTGGACGGACTCCTGGTCAGCATGATT
GCATCTCTGCAAAAGTATAGAGAGGTAAATGGACATTATCCTGAGCAAATCACAATATTTAGAGATGGAGT
GTCTGACGGTCAGCTGCGTCTTTGTGAAGAGTACGAATTACCTCAAATAACGAATGCATGTAGAAAAATTT
CACCTGAATACACACCAAAGATCCTTTTTGTGGTAGTACAGAAGAGGATTAACACGAGGCTGTTCGGAATT
GAACGTGATAAGAGTTTTTCCAATCCATTGCCAGGAACTGTTATGGATCACACTATTACCCGTCGTTATCTT

TATGATTTCTTCTTGGTATCTCAACACGTTAATCAGGGAACAGTCTCTCCAACCCATTACATTGTTGTAAAA
AATACGACCAGTATGTCTCCAGATCAAGTTCAGCGATTCACGTATATGCTGACCCATCTGTATTATAACTGG
CCAGGAACGATTAGGGTTCCTGCTCCTTGTCAGTATGCTCACAGGCTTGCTTATTTAGTAGGCGAAAATATT
CATAAAGAAGCATCTGAATCTCTGTCAGACAAACTGTTTTATTTGTAAATTTATGTATACTTTCATACTATA
CATATATATATGTATATAAAATAATGGCATTATTTTGTACTATAAAAAATATATTTTTATTTGTTATTTTTTT
TGTAAAAAA

Protein

RF: +3

ORF: 138 -> 2828

Length: 896 aa

lcl|ORF6_TRINITY_DN83747_c0_g1_i1:137:2827 unnamed protein product

MEGRRLGRGEALRKLLESQPQSLPHAQSFSQPEPSSQRSPPTHEQDPYSYGQDVAAHTSGPVLRKPMGRSALLA
SELRRSEESREEAGEAPKPTGRGRALLASLAAREVTPEVEQISRQLSRTSLTESSEADKEPVVMKGTFGRGIPIGV
NYVKLKVEKGKGLFQYDVQYDPPVESRSLKFGILNNVKNVIGETRLFDGMVLYLPIKLPQKVTIVKTTVAHDNS
VVTVRITFTKERKLGDPEAIHLYNVLFRRIMVILGLALHGKNFYDPKSAMPIPLHKLEVWPGYITAIDEFEGGIML
CCDASHRVLRNQTVLQLMEDMQNSNHKHWKDEFVQLILGQSVLTKYNNKVYRIDDLSFDECPNDCFEKSNGE
RVKYVDYYRLQYNVRVTDLNQPLLKSRVKTRVKGKEERQLVSLVPELCFLTGITDTMRADNRVMKDITQCISIS
PNQRHYAINQFVNNIKGSPEATKVLSDWGISLDHAYMILEGRNIPPIDIIFGGGVTVPGNREANWSGASNKHKAL
SVIDFSSWSVVCTRRDMDMVSSFVDQMIKIGPQMGIKIMKPRMVPIPDDRTDNYIRSLRSSIREKVQIVVIIFPLAR
TDKYSAVKKLCCIEEPVPSQVILSRTVRRAGTSKAITSKIALQMNCKLGGTLWAVNVPLKYTMVCGLDTYHDPK
RRANSVGALVSSLNQPLTRWYSKIYSQAAGLEFVDGLLVSMIASLQKYREVNGHYPEQITIFRDGVSDGQLRLC
EEYELPQITNACRKISPEYTPKILFVVVQKRINTRLFGIERDKSFSNPLPGTVMDHTITRRYLYDFFLVSQHVNQGT
VSPTHYIVVKNTTSMSPDQVQRFTYMLTHLYYNWPGTIRVPAPCQYAHRLAYLVGENIHKEASESLSDKLFYL

Conserved Domains

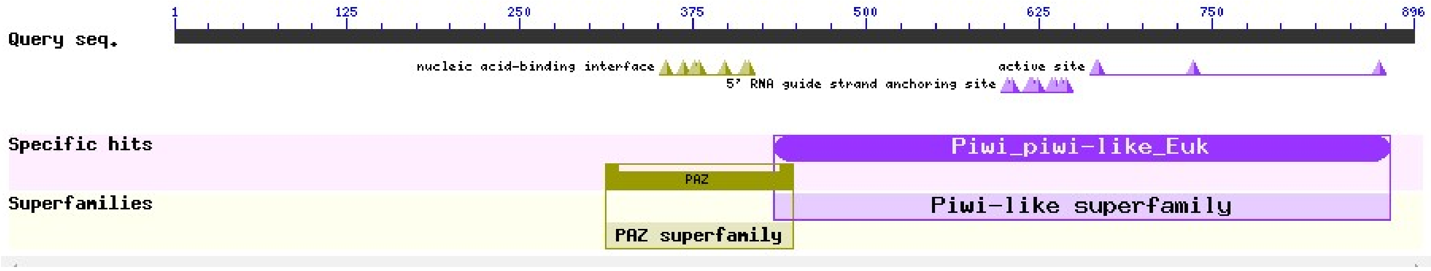

BLASTp

XP_014276831.1 1 piwi-like protein Ago3 [*Halyomorpha halys*]

Score:1628 bits

E-value: 0.0

Query 1 MDGRRLGRGEALRKLLESQPH-LSHAQSYSQHEPSSHRSSSSLSSAAQEQDPYTYRQDVA 59

M+GRRLGRGEALRKLLESQP L HAQS+SQ EPSS RS + EQDPY+Y QDVA

Sbjct 1 MEGRRLGRGEALRKLLESQPQSLPHAQSFSQPEPSSQRSPPT-----HEQDPYSYGQDVA 55

Query 60 AHTSESQGVKKPMGRSALLLGGEMRKSEELKEEI---PKPSGRGRALLASLVAREGSPSV 116

AHTS ++KPMGRSALL E+R+SEE +EE PKP+GRGRALLASL ARE +P V

Sbjct 56 AHTS-GPVLRKPMGRSALL-ASELRRSEESREEAGEAPKPTGRGRALLASLAAREVTPEV 113

Query 117 EKITRHLSRTSLAGSTVEEKEPVVMKGSFGREISVGVNYVKLKVEKGKGLFQYDVQYDPP 176

E+I+R LSRTSL S+ +KEPVVMKG+FGR I +GVNYVKLKVEKGKGLFQYDVQYDPP

Sbjct 114 EQISRQLSRTSLTESSEADKEPVVMKGTFGRGIPIGVNYVKLKVEKGKGLFQYDVQYDPP 173

Query 177 VESRSLKFGILNSVKDVIGDTKLFDGMVLYLPFKLNQKVTILKTTVAHDNSVVTVKITFT 236

VESRSLKFGILN+VK+VIG+T+LFDGMVLYLP KL QKVTI+KTTVAHDNSVVTV+ITFT

Sbjct 174 VESRSLKFGILNNVKNVIGETRLFDGMVLYLPIKLPQKVTIVKTTVAHDNSVVTVRITFT 233

Query 237 KERKLGDPEAIHLYNVLFRRIMVILGLALHGKNFFDPKSAMPIPLHKLEVWPGYITAIDE 296

KERKLGDPEAIHLYNVLFRRIMVILGLALHGKNF+DPKSAMPIPLHKLEVWPGYITAIDE

Sbjct 234 KERKLGDPEAIHLYNVLFRRIMVILGLALHGKNFYDPKSAMPIPLHKLEVWPGYITAIDE 293

Query 297 YEGGIMLCCDASHRVLRNQTVLQLMDDLQNSNQKHWRDEFVQLILGQSVLTKYNNKVYRI 356

+EGGIMLCCDASHRVLRNQTVLQLM+D+QNSN KHW+DEFVQLILGQSVLTKYNNKVYRI

Sbjct 294 FEGGIMLCCDASHRVLRNQTVLQLMEDMQNSNHKHWKDEFVQLILGQSVLTKYNNKVYRI 353

Query 357 DDVSFEECPNDCFEKSNGEKVRYVDYYRLQYNLRLTDSNQPLLKSRVKMRVRGKEEKQLV 416

DD+SF+ECPNDCFEKSNGE+V+YVDYYRLQYN+R+TD NQPLLKSRVK RV+GKEE+QLV

Sbjct 354 DDLSFDECPNDCFEKSNGERVKYVDYYRLQYNVRVTDLNQPLLKSRVKTRVKGKEERQLV 413

Query 417 SLVPELCFLTGITDTMRADARVMKDITQCISISPNQRHYAINQFTNNIRASPEATKVLSD 476

SLVPELCFLTGITDTMRAD RVMKDITQCISISPNQRHYAINQF NNI+ SPEATKVLSD

```
Sbjct  414  SLVPELCFLTGITDTMRADNRVMKDITQCISISPNQRHYAINQFVNNIKGSPEATKVLSD  473


Query  477  WGISLDDAYMVLPGRNIPPIDVMFGGGVTIPGSREANWSGASNKNKAISVVNFVTWSVIC  536
            WGISLD AYM+L GRNIPPID++FGGGVT+PG+REANWSGASNK+KA+SV++F +WSV+C
Sbjct  474  WGISLDHAYMILEGRNIPPIDIIFGGGVTVPGNREANWSGASNKHKALSVIDFSSWSVVC  533


Query  537  TRRDMDMVTSFIEQMIKIGPQMGIKINKPDIVSIPDDRTDNYIRSLRSSIKNNIQIVVVI  596
            TRRDMDMV+SF++QMIKIGPQMGIKI KP +V IPDDRTDNYIRSLRSSI+  +QIVV+I
Sbjct  534  TRRDMDMVSSFVDQMIKIGPQMGIKIMKPRMVPIPDDRTDNYIRSLRSSIREKVQIVVII  593


Query  597  FPLARTDKYSAVKKLCCVEEPIPSQVILARTVRRAGTSKAITLKIALQMNCKLGGTLWAV  656
            FPLARTDKYSAVKKLCC+EEP+PSQVIL+RTVRRAGTSKAIT KIALQMNCKLGGTLWAV
Sbjct  594  FPLARTDKYSAVKKLCCIEEPVPSQVILSRTVRRAGTSKAITSKIALQMNCKLGGTLWAV  653


Query  657  NVPLKYTMVCGLDTYHDPKRRADSVGALVSSLNQPLTRWYSKIYSQSTGLEFVAGLQVSM  716
            NVPLKYTMVCGLDTYHDPKRRA+SVGALVSSLNQPLTRWYSKIYSQ+ GLEFV GL VSM
Sbjct  654  NVPLKYTMVCGLDTYHDPKRRANSVGALVSSLNQPLTRWYSKIYSQAAGLEFVDGLLVSM  713


Query  717  IASLQKYREVNGSYPEQITIFRDGVSDGQLRLCEDYELPQIMNACQRISPEYMPKILFVV  776
            IASLQKYREVNG YPEQITIFRDGVSDGQLRLCE+YELPQI NAC++ISPEY PKILFVV
Sbjct  714  IASLQKYREVNGHYPEQITIFRDGVSDGQLRLCEEYELPQITNACRKISPEYTPKILFVV  773


Query  777  VQKRINTRLFGVERDKSFSNPMPGTVMDHTITRRYLHDFFLVSQHVSQGTVSPTHYIVVR  836
            VQKRINTRLFG+ERDKSFSNP+PGTVMDHTITRRYL+DFFLVSQHV+QGTVSPTHYIVV+
Sbjct  774  VQKRINTRLFGIERDKSFSNPLPGTVMDHTITRRYLYDFFLVSQHVNQGTVSPTHYIVVK  833


Query  837  NTTSMSPDQIQRFTYMLTHLYYNWPGTIRVPAPCQYAHRLAYLIGENIHKEAAESLSDKL  896
            NTTSMSPDQ+QRFTYMLTHLYYNWPGTIRVPAPCQYAHRLAYL+GENIHKEA+ESLSDKL
Sbjct  834  NTTSMSPDQVQRFTYMLTHLYYNWPGTIRVPAPCQYAHRLAYLVGENIHKEASESLSDKL  893


Query  897  FYL  899
            FYL
```

Sbjct 894 FYL 896

Aubergine (Aub)

>TRINITY_DN5247_c0_g1_i2 len=3987 path=[0:0-798 1:799-1291 3:1292-3986]

TTGGAATATGGCGAGGTGTTACTGATTCGGCATTCGTTTGTTTACCTTTAAGGAAGTTGGCTCTTCGTGTTGT
TACTTGTGTGTTATTTTAAACCGCCTGATTAGATAACAATATTTGATCTTGCAGAAACCAAACTTAAGACAA
GATGTCTGAAGGACAGCAACCTAAGGGTCGAGCTAGGGGGAGAGCGTGGGGCAGGGCCCGTGGGGTCCCA
ACAGCTGCGCAACAGCAGCAACAATCCCAGCAACGGCTGCAACCCCAACAACAGCAGTCTCCCCAGCAAC
GGCTGCAACCCCAACAACAGCAGTCTCCCCAACAACTGCAGTCACCTCAACCACAATATCAGCTGCCCGGC
ACTTCGTCCCAGCAGCTTCCTCTCCTTCCCCAGCCACAACAGCAGCCGAGAGCTTCAGCTCGAGCGAGGGG
GCACGTGCCTAAGCAAGTGCAACCTGGTCCTAGTGGTGTTCAACAGATGAGGCAGCAGCAGGAACAGCAA
CAGATAATGCAGCAGCAGGAACAGCAACAGCAAGCACCGGAAGAAGGGGCATGTGCGTTCGATCCCGAGA
TAGCATTGTTATCTGGAAATTTCAACGTGTTGACAATTGGTGATGCTAAGCCAGGGAAACGTAGAGGGGCT
CTGAGGGGAAGGCTTGATATAAACGCGGAACCTCCGATCAAAACTAGACCAGAGAATTTGAGTGAAAAAA
CAGGAACCATGGGAACGAAGATAAAATTTGTAACCAACTATTTTAGATTGACCCAAGTAACCAGATGGTCT
TTATATCAATATAGAGTCGATTTTAGTCCCGAGGAGGAAAGAACTAAAGTCCGTAGAGAACTTATGGAATA
TCATAAGGATACGTTAGGAGAGGCGTTTATCTTTGATGGATCAATGTTGTTCTGTATTCAAAAGCTTCATCC
CAATGTTAATAATAGAATCGAATTATTTACTAGAAGAGGACCTGAACAACCTATCATAAGAATAACAATAA
GGTTTACCAATGAGTTATTATTTGGGGATTATCAATATATTCAATTATATAACATAATCCTTCGGAACTGTTT
AGGACTCATGCAATTTCAACTTGTAGGGCGTGATTACTTTGATCCTGATAATTCTATGTCTATCAAGGGTTA
TGGACTCGAAATTTGGCCAGGCTATAGTACTTCAATTTTACAATTTGAAAACAACATTATGATGGGAATTGA
TGTATCACATAAGGTCTTACGGTGTGATAATGCTTTGGACATTCTTAAGGAATGTAAAAAAAGATACGGAT
CTGAGTGGAAGAAATACTACTTGGACACCATGTTAGGTGCTGTTGTTTTTACCTTTTACAACAATAAGCCTT
ATCGGGTGGATGATGTAGCATTTGATATGGCTCCAACCGATACATTCAGCTTTAAGGGCAGAGACATAACT
TATGTAGAATACGTTAAGGAGAAATACGGCGTAGTAGTAAATGACTTCACACAACCATTACTTATATCAAG
GCCCAAAGCAAGAGATATTCGAGGTGGAAGGGATCAAAATATCATTCTTATTCCCCAATTTTGTAGTATGA
CTGGGCTTACCGATGAGATGAGGAGTAATTTCAACTTAATGAGGACAGTTGCGGATCATACTCGGGTAGTT
CCAGGCAAGCGAATTGAGTCTTACAATGCGTTTATGAGGAGGCTTACGCAATGCTCTGAAGCTATGGAAAA
ACTTGTCGCATGGAACATCCAGTTCTCTCAAAAATTGGAAGAAGTGCCAGGCAGAGTGTTTGGTTCAGAAA
TTCTCTATATGGCGAGGAATCAAACTGTAACTCCACGTGACGGGGATTGGTCCCGTGAAATACGCCATGTT
AGCATGTTTTCATCTGTTCGTTTAGATAGGTGGGTATTAGTAATTAGCAGGCAAGCAATGTTTGATAAGAGG
AACAATAGAGATAACGTCAAATACTTCACTAATCAACTCATCACAGCAGCCAACAGTTTGCTTTTTGATAT
GAATAAACCTGAAGTGGTTCCTGTTGAGGTTGATAAGATATCGAGCTATATTTCCGCCATTGACCAAGGTGT
GGGTAAAGGAGGGATGCGATTGGTGATGTGTGTTATCAATGGAAACAGGTCAGACTTGTACGCTGCCATTA
AAAAGAAATGTGTCTGTGATAGGCCAATTCCTTCTCAAGTTATAGCGTCTAAAACACTAGGGCACAAAAAT
CTGCTGTCAGTTTGCACAAAAATTGTCATACAGTTAAACTGTAAATTGGGCGGAAGTCCTTGGTTCACCCCA
GTCCCATTTAAGGATACAATAGTGTTGCCGGATGGTACTTTACAACCGGATACGCAACATGCTACAATGAT
AGTTGGATTTGATGTATGCCATGATACAAGATCCAAGGGGAAATCGGTAGGTGCAATGGTAGCCTCCCTTG
ATTACAATTTCTCAAGGTTTATTCTTCCGTGAGTAGGCACGGTTTAGGAGAGGAATTGTCAAATGACATCG
CCACCCACATTTCCCATGCTGTCATAAGATTTCAAGAAATCAACAAGTACCCACCTCCTAAAATAGTCATTT
TTAGAGACGGTGTTGGAGAATCCAACATAAAATATGTGAAGGAACATGAAATTTTGACAATAAAGGGTAG
ACTTTGTCAAATGTTTCCTAATAATACTCCTAAATTAACTGTCATCATTGTATCAAAGAGAATTCAGACCAG
ACTTTTTAAGGTAAAGGAGAACAAGTATATCAACCCTGAACCTGGTTCTATTGTAGATGATGTGATTACCCA
ACCTGAAAAGTATGACTTTTTCTTGGTGTCACAATCAGTTCGACAGGGTACCGTGACTCCTACAAACTACAA
TGTCATCTTTGATGATAGTAAAATGAAGCCTGATCATGTTCAGAGGATGGCGTATAAAATGTGCCATCTTTA
TTACAATTGCACCACTACGGTGAGAGTTCCGTGCCAGGTGCAGTACGCTCATAAGCTTGCGTTCCTGATCGG
CCAGGTCATCCACATTCCTCCTGGCCCAGAGATGGACCATCTTTTGTACTTCCTTTAACAACTTTTATCCGTT
TCTCAATCTTCTGAGAAAGAATTTGTGAATTTTGTAGATTAGCAGTTTTTTTTTTTTTTTTTTATATATATAT
ATATATATATATATATATATATATTTTCTTTTTTTTAATTTTATATTCATTAAATATGGAAATTTTTTGTTATC
TTTTTAAAAGTATTATCTTTACTTTAGTAATTTTTAAGTGAGTCCTGTATTTTTTAGCTGTTTATTATAAAAA
ATTACACAGTATTTGTGTAAATCTTTTTCCCCTTGAAATTGAATTATTTTTTGTTTGTATTACTGTGTTAATTA

AATAATATTATGGCTGTCGAGAGTTACATTAATTTTTGCTTTGTATTCAGGCACGCTTTTATTTAACTTAACG
TGCCCTGAAATTGTTTTGGTTATTTTAAGTTGGGATATCAAATTAGTTTAGTTGTTTTCCTTCCTTTAACATA
TATGTGTAATATTTGTTCATTTTTCTATAAAAAATTTCATTGGGCTGTTATATCATAACACCTTTGATAAATC
TTGTTCCTCTTATCATTGTTGGGGAAGGATTCAGCAGTTCCTCATTATTAATGGTTTTATATATATATTCTTTCA
TATGTGTATTTTTTTAAGTTATGTATTCATTGAGTTTTATAGGAATATATATTTTTTTTAAATATTTGTAAATA
ATGTTTATATAAATGTTAATACATTTTTTTAATTCTAAAAAACCTGAGACTGAGAGGCGAGGAGGTAAATTG
TACAATAATTTAGGAGTTTTTATCTAAAATTGACTCAATATTGAGAAAAAAAAAAGATTTATTTTTTTATTA
TAAATTACATTTTTGTGATAATGTTACTTCTGTACATTAAATTCTATAATATGTAAACAACACTGCCTTTTTT
TATTTTCTGTTAGTAAAATATAGATATCATTTATCTAAAAA

Protein

RF: +3

ORF: 147 -> 3053

Length: 968 aa

>lcl|ORF7_TRINITY_DN5247_c0_g1_i2:146:3052 unnamed protein product

MSEGQQPKGRARGRAWGRARGVPTAAQQQQQSQQRLQPQQQQSPQQRLQPQQQQSPQQLQSPQPQYQLPGTS
SQQLPLLPQPQQQPRASARARGHVPKQVQPGPSGVQQMRQQQEQQQIMQQQEQQQQAPEEGACAFDPEIALLS
GNFNVLTIGDAKPGKRRGALRGRLDINAEPPIKTRPENLSEKTGTMGTKIKFVTNYFRLTQVTRWSLYQYRVDFS
PEEERTKVRRELMEYHKDTLGEAFIFDGSMLFCIQKLHPNVNNRIELFTRRGPEQPIIRITIRFTNELLFGDYQYIQL
YNIILRNCLGLMQFQLVGRDYFDPDNSMSIKGYGLEIWPGYSTSILQFENNIMMGIDVSHKVLRCDNALDILKEC
KKRYGSEWKKYYLDTMLGAVVFTFYNNKPYRVDDVAFDMAPTDTFSFKGRDITYVEYVKEKYGVVVNDFTQ
PLLISRPKARDIRGGRDQNIILIPQFCSMTGLTDEMRSNFNLMRTVADHTRVVPGKRIESYNAFMRRLTQCSEAM
EKLVAWNIQFSQKLEEVPGRVFGSEILYMARNQTVTPRDGDWSREIRHVSMFSSVRLDRWVLVISRQAMFDKR
NNRDNVKYFTNQLITAANSLLFDMNKPEVVPVEVDKISSYISAIDQGVGKGGMRLVMCVINGNRSDLYAAIKK
KCVCDRPIPSQVIASKTLGHKNLLSVCTKIVIQLNCKLGGSPWFTPVPFKDTIVLPDGTLQPDTQHATMIVGFDVC
HDTRSKGKSVGAMVASLDYNFSRFYSSVSRHGLGEELSNDIATHISHAVIRFQEINKYPPPKIVIFRDGVGESNIK
YVKEHEILTIKGRLCQMFPNNTPKLTVIIVSKRIQTRLFKVKENKYINPEPGSIVDDVITQPEKYDFFLVSQSVRQG
TVTPTNYNVIFDDSKMKPDHVQRMAYKMCHLYYNCTTTVRVPCQVQYAHKLAFLIGQVIHIPPGPEMDHLLYF

Conserved Domains

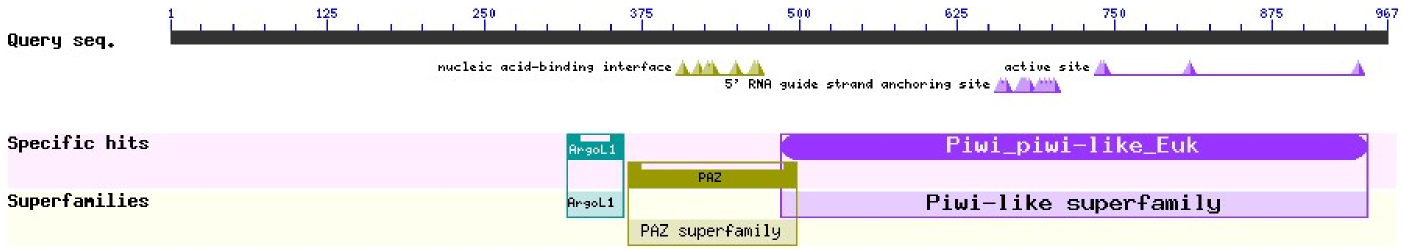

BLASTp

XP_014275927.1 PREDICTED: protein aubergine-like isoform X3 [*Halyomorpha halys*]

Score:1195 bits

E-value: 0.0

Query 145 RSAGRARGIVPKQVQPGASGVQQMLQQQPPQSQSQPTPQPQQERHEEGACAIDPEIKSV 204

R++ RARG VPKQVQPG SGVQQM QQQ+ Q Q Q Q EEGACA DPEI +

Sbjct 87 RASARARGHVPKQVQPGPSGVQQMRQQQEQQQIMQQQEQQQQAP--EEGACAFDPEIALL 144

Query 205 SGAFQGLKVGESKPGNLRRGMMRGRLTV-VTPPPKTRPDNLSVKLGVSGTPVKLVTNYYR 263

SG F L +G++KPG RRG +RGRL + PP KTRP+NLS K G GT +K VTNY+R

Sbjct 145 SGNFNVLTIGDAKPGK-RRGALRGRLDINAEPPIKTRPENLSEKTGTMGTKIKFVTNYFR 203

Query 264 LTKSTNWSLYQYRVDI-PNEERTKVRKGLISCHKDRIGDAYLFDGTMLFVIQKLHPDRNA 322

LT+ T WSLYQYRVD P EERTKVR+ L+ HKD +G+A++FDG+MLF IQKLHP+ N

Sbjct 204 LTQVTRWSLYQYRVDFSPEEERTKVRRELMEYHKDTLGEAFIFDGSMLFCIQKLHPNVNN 263

Query 323 VIELCSKRHDDVKIT-MTIRFTNEMLVGDYQYIQLFNIILRNCMDDMKYQLVGREYFDAR 381

IEL ++R + I +TIRFTNE+L GDYQYIQL+NIILRNC+ M++QLVGR+YFD

Sbjct 264 RIELFTRRGPEQPIIRITIRFTNELLFGDYQYIQLYNIILRNCLGLMQFQLVGRDYFDPD 323

Query 382 KSIKMPNYHLEIWPGYSTSILQFENHIMMGIDVSHKVLRSDNVWEFLRACRERHGTDYEK 441

S+ + Y LEIWPGYSTSILQFEN+IMMGIDVSHKVLR DN + L+ C++R+G++++K

Sbjct 324 NSMSIKGYGLEIWPGYSTSILQFENNIMMGIDVSHKVLRCDNALDILKECKKRYGSEWKK 383

Query 442 HFQDTMLGAVVFTFYNNKPYRVDDVAFDETPISTFTTKKGQNTSYFDYYHTKYGVEIKDL 501

++ DTMLGAVVFTFYNNKPYRVDDVAFD P TF+ K G++ +Y +Y KYGV + D

Sbjct 384 YYLDTMLGAVVFTFYNNKPYRVDDVAFDMAPTDTFSFK-GRDITYVEYVKEKYGVVVNDF 442

Query 502 NQPLLVSRPKPRDIRAGRSNNIILIPELCQMTGINDEMRNNFTLMKAVAAHTRVEPEKRI 561

QPLL+SRPK RDIR GR NIILIP+ C MTG+ DEMR+NF LM+ VA HTRV P KRI

Sbjct 443 TQPLLISRPKARDIRGGRDQNIILIPQFCSMTGLTDEMRSNFNLMRTVADHTRVVPGKRI 502

Query 562 QSYYNFMKDLGSCEKAQEKLEVWNVQFSQELETVQGRILHPEDLHVGGNKMIRPESGDWS 621

+SY FM+ L C +A EKL WN+QFSQ+LE V GR+ E L++ N+ + P GDWS

Sbjct 503 ESYNAFMRRLTQCSEAMEKLVAWNIQFSQKLEEVPGRVFGSEILYMARNQTVTPRDGDWS 562

Query 622 RPLRAASMLRIVKLKNWIVIASKQATYDKRVRGNPLDNFLSDLYKAAGTLKFKFDEPEVI 681

R +R SM V+L W+++ S+QA +DKR + + F + L AA +L F ++PEV+

Sbjct 563 REIRHVSMFSSVRLDRWVLVISRQAMFDKRNNRDNVKYFTNQLITAANSLLFDMNKPEVV 622

Query 682 IIDQDKISNYLGAIDKAMSASKLQLVMCIINYSRNDLYAAIKKKCLCDRPIPSQVIATKT 741

++ DKIS+Y+ AID+ + ++LVMC+IN +R+DLYAAIKKKC+CDRPIPSQVIA+KT

Sbjct 623 PVEVDKISSYISAIDQGVGKGGMRLVMCVINGNRSDLYAAIKKKCVCDRPIPSQVIASKT 682

Query 742 LGHKNLLSVCTKIAIQINCKLGGSPWFTPVPFKENIVRNDGTVLENMIHAIMIVGFDVCH 801

LGHKNLLSVCTKI IQ+NCKLGGSPWFTPVPFK+ IV DGT+ + HA MIVGFDVCH

Sbjct 683 LGHKNLLSVCTKIVIQLNCKLGGSPWFTPVPFKDTIVLPDGTLQPDTQHATMIVGFDVCH 742

Query 802 DTRLKGQSVGAMVASLDYMFSQFYSSVSRHNLGEELSNDIANHISCAVMRFQELNQYLPP 861

DTR KG+SVGAMVASLDY FS+FYSSVSRH LGEELSNDIA HIS AV+RFQE+N+Y PP

Sbjct 743 DTRSKGKSVGAMVASLDYNFSRFYSSVSRHGLGEELSNDIATHISHAVIRFQEINKYPPP 802

Query 862 KIVVFRDGVGESNINYVKEHEIKRIKERLLEMFPNRTPRLTVIIVSKRIQARFFMEDRKG 921

KIV+FRDGVGESNI YVKEHEI IK RL +MFPN TP+LTVIIVSKRIQ R F + ++

Sbjct 803 KIVIFRDGVGESNIKYVKEHEILTIKGRLCQMFPNNTPKLTVIIVSKRIQTRLF-KVKEN 861

Query 922 KYLNPLPGTVVDDVITQPEKYDFFLVSQSVRQGTVTPTNYNVIFDECNMKPDHVQRLAYK 981

KY+NP PG++VDDVITQPEKYDFFLVSQSVRQGTVTPTNYNVIFD+ MKPDHVQR+AYK

Sbjct 862 KYINPEPGSIVDDVITQPEKYDFFLVSQSVRQGTVTPTNYNVIFDDSKMKPDHVQRMAYK 921

Query 982 MCHLYYNCTSTVRVPCQVQYAHKLAFLVGQVIHIPPNPVLDHLLYF 1027

MCHLYYNCT+TVRVPCQVQYAHKLAFL+GQVIHIPP P +DHLLYF

Sbjct 922 MCHLYYNCTTTVRVPCQVQYAHKLAFLIGQVIHIPPGPEMDHLLYF 967

Piwi

>TRINITY_DN56355_c0_g1_i1 len=2826 path=[0:0-2825]

CTGTAGCCTCTCAATAGCCATTTTGAAGAATGGCTTCTGCTTAGTTTTATCTTCATTGTTTACTGTTATAAGT
CCATATATTTATTGGTTTTATTTTGGAAACATGGAAGAGGGAAGAGCTCGAGGAAGGGCCAGGGGCAGAGC
TAGACAGGATCCTAAGAAAACCGAGGTGACTCAGAATATAATTGATTCCAGAGGAGTTAGGTCAAAGCCA

CAGCATGTACCTCCTACAGTAGCTGCTGTAGTTGGCGATATGAAACAGCTTTCAGTTACACCTAGAAGACC
TGGAGGTAGAAAAATGATAGACAAATTGGATTTTGTGTCTATTAGATCAAGGCCCAAGGAACTTGTCACTA
AACAGGGAACAACAGGTCAGAAGGTAAAACTTCAAGCCAATTATTTTAAATTAGAAACCCACCCTGATTGG
GCCTTATACCAATATAGAGTTGATTTTTCTCCTGAAGAAGAGAGAACTTCTGTGAAAAAAGCACTGCTGAA
ACCACACAAGGACATTTTTAACTGTGCATATCTCTTTGATGGCACTGTGTTGTATGTTTGTAACAGATTAAA
AACTGATCCTATTGAACTTTTCTCCCTTAGAGACACAGATAGAAAGAAAATTAGGATAACCATTAAATTTGT
AGGGGATGTAGTTATGGGCGATTATCAATATCTACAAGTTTTCAATATCATAATGAGAAAATGCTTAGATA
ACTTAAAACTACAAATGGTTGGCCGTAATTTCTTTGATGCGAAAGCAAGAATCGAAATTCGTGAGTACAGG
ATGGAACTTTGGCCAGGTTATTTAACATCTATCAGACAGTGTGAAGATCATATTCTTATGAATGCTGAAATA
ACGTATAAAGTGATGCGTTGTGAGACTGTTCTTGATTTAATTGTTAAGTGTAGATCATCTGAAGATTGGGAG
AAGACATTTGAGAATGCAATCATAGGTACTATTGTTCTGACTGATTATAACAATCGTACCTATCGGATTGAT
GATGTGAATTTCAATTGTACACCCATGTCTAAATTTAAAATTAAGAATGGAGAGTCGTCTACTTATTGTGAA
TACTATAAGTCGAGATATGGTATAAGAATTAGAGAACCAAATCAACCGATGCTGGTTTCTAAAGCTAAACC
ACGGGAAGTCCGTGCTGGAATGACTGAAATTATATATTTGGTCCCAGAACTGTGTCGATTAACCGGATTAA
CTGATGACATGAGAACAAACTTTCAGCTCATGAGGGCACTTGCTGAGCATACTCGAGTCACACCTAAGCTA
AGAATTGAGAAACTTTTGAAGTTTAATCACCGTTTAAAAGAGAGTAAAGAAATCACTAAAGATTTGGATTC
ATGGAATATGAAGTTAGCTGATTCATTGATAACATTTGATGGAAGGGTTCTTCCAATGGAAAAGATCTATTT
TGGAGACAATAGAGCTGTTGCTGCTGGTAATGATGCTGATTGGACTCGAAGCATGAGAGATCATTCTATGT
TAACTAGTGGAAATTTTAAATCTTGGTCCATCGTTTATTTAGGGAGAACCAAGAGTGAAGTTAACAGCTTCA
TTCACACCCTTGCAAAGGCAGCAGAGAGTTTAAACTTTCGGGTTCCAACACCTAGAATGTCTGAAGTCATT
AATGACAGATGTGGTAGTTTTGTTGAAGCATTAGACTCAGTGATTTCCATGCATAATCCACAATTAATATTA
TGTATTGTACCAAACAGTAGAGCTGATCGGTATGCAGCGATAAAGAAGAAATGTTGTGTTGATAGGGCAGT
CCCCACCCAAGTTGTTGTGGCAAAACATTTTAATTCCAAAAATCTTATGGCAATTTCTACTAAAATCGCCAT
TCAAATAAATTGTAAACTTGGTGGCATCCCATGGACCATAGCAAACCCTCTTAAGGGGTTGATGGTAGTGG
GATATGATGTCTGTCATGACGCTTCACGAAAAGACATGTCTTATGGAGCTATGGTGGCTTCGTTGAATCCTT
CTCTTTCAAGGTATTATTCATCTGTGACTCCTCATTCTCATGGAGAAGAACTGTCTAATGACTTAGCCATGA
ATATATTCAAAGCAATTATTCGTTATAAAGAAAACAATGAAGGTAATATTCCTTCAATGATAGTACTTTATC
GAGATGGTGTAGGAGAGGGTCAGATACCTTTTGTTTATAATCATGAAGTAAGAATTGTCAAAGAACGACTC
TCAGAAATATATAAGGATAAACCTCCTAAGTTGGGATTTGTGATAGTCACCAAGAGACTCAACACCCGATT
ATTCCTTAATGGCAGTAATGCTACCCCCGGTACAGTTGCAGACGACTGTATTACATCTCCTGATCGATATGA
TTTCTTTCTCGTTTCACAGTCTGTACGTCAAGGCACAGTAAGTCCTACATCCTACAATGTTATTGATGATTCA
ACAGGTCTGGATGCAGACAAAATGCAACGCCTCGCTTACAAAATGACCCATTTGTATTATAACTGGAGTGG
AACAGTAAGAGTTCCTGCTCAATGTCAGTATGCACATAAGTTGGCTTTTCTTGTAAGCCAATGTTTGCATAG
AGCTCCGAATCCTGATCTTGATGATCTTTTATATTTTCTTTAAAAAGTAATTTATTCAGAACTGATGTTCTCT
ATTATAATCGTTAGTGTAGGTTGAATAATGATTTTAGAGGTGTAATTCTTTATTTTAAGTTTTGATACTTTTT
AATCTAATTTCTTATTTAAATTTGCAAAAGAAAAAAATCTTAATATTTTTACTTTATTTTCAGAGTTATTCAT
TTTAACAAGTTAATTAATTTCTAACAAGTTAGT

Protein

<u>RF:</u> +2

<u>ORF:</u> 104 -> 2617

<u>Length</u>: 837 aa

>lcl|ORF1_TRINITY_DN56355_c0_g1_i1:103:2616 unnamed protein product

MEEGRARGRARGRARQDPKKTEVTQNIIDSRGVRSKPQHVPPTVAAVVGDMKQLSVTPRRPGGRKMIDKLDFV
SIRSRPKELVTKQGTTGQKVKLQANYFKLETHPDWALYQYRVDFSPEEERTSVKKALLKPHKDIFNCAYLFDGT
VLYVCNRLKTDPIELFSLRDTDRKKIRITIKFVGDVVMGDYQYLQVFNIIMRKCLDNLKLQMVGRNFFDAKARIE

IREYRMELWPGYLTSIRQCEDHILMNAEITYKVMRCETVLDLIVKCRSSEDWEKTFENAIIGTIVLTDYNNRTYRI
DDVNFNCTPMSKFKIKNGESSTYCEYYKSRYGIRIREPNQPMLVSKAKPREVRAGMTEIIYLVPELCRLTGLTDD
MRTNFQLMRALAEHTRVTPKLRIEKLLKFNHRLKESKEITKDLDSWNMKLADSLITFDGRVLPMEKIYFGDNRA
VAAGNDADWTRSMRDHSMLTSGNFKSWSIVYLGRTKSEVNSFIHTLAKAAESLNFRVPTPRMSEVINDRCGSFV
EALDSVISMHNPQLILCIVPNSRADRYAAIKKKCCVDRAVPTQVVVAKHFNSKNLMAISTKIAIQINCKLGGIPW
TIANPLKGLMVVGYDVCHDASRKDMSYGAMVASLNPSLSRYYSSVTPHSHGEELSNDLAMNIFKAIIRYKENNE
GNIPSMIVLYRDGVGEGQIPFVYNHEVRIVKERLSEIYKDKPPKLGFVIVTKRLNTRLFLNGSNATPGTVADDCIT
SPDRYDFFLVSQSVRQGTVSPTSYNVIDDSTGLDADKMQRLAYKMTHLYYNWSGTVRVPAQCQYAHKLAFLV
SQCLHRAPNPDLDDLLYFL

Conserved Domains

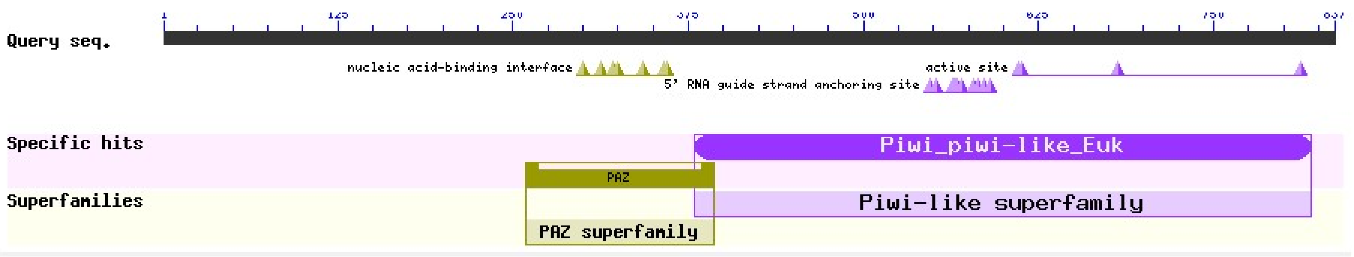

BLASTp

XP_014270559.1piwi-like protein Siwi [*Halyomorpha halys*]

Score:1714 bits

E-value: 0.0

Query 1 MEEGRARGRARGRSRQDPKKTEVTHNVIEARGVRSKPQHASPTVATVVGDMKQLAVTPRR 60

MEEGRARGRARGR+RQDPKKTEVT N+I++RGVRSKPQH PTVA VVGDMKQL+VTPRR

Sbjct 1 MEEGRARGRARGRARQDPKKTEVTQNIIDSRGVRSKPQHVPPTVAAVVGDMKQLSVTPRR 60

Query 61 PGGRKMMDKLDFVSIRSRPKELLTKQGTTGQKVKLQANYFKLETHPDWALYQYRVDFSPE 120

PGGRKM+DKLDFVSIRSRPKEL+TKQGTTGQKVKLQANYFKLETHPDWALYQYRVDFSPE

Sbjct 61 PGGRKMIDKLDFVSIRSRPKELVTKQGTTGQKVKLQANYFKLETHPDWALYQYRVDFSPE 120

Query 121 EERTSVKKALLKPHKDIFNCAYLFDGTVLYVCNRLKTDPIELFSLRDTDRKKIRITIKFV 180

EERTSVKKALLKPHKDIFNCAYLFDGTVLYVCNRLKTDPIELFSLRDTDRKKIRITIKFV

Sbjct 121 EERTSVKKALLKPHKDIFNCAYLFDGTVLYVCNRLKTDPIELFSLRDTDRKKIRITIKFV 180

Query 181 GDVVMGDYQYLQVFNIIMRKCLDNLKLQMVGRNFFDARARIEIREYRMELWPGYLTSIRQ 240

GDVVMGDYQYLQVFNIIMRKCLDNLKLQMVGRNFFDA+ARIEIREYRMELWPGYLTSIRQ

Sbjct 181 GDVVMGDYQYLQVFNIIMRKCLDNLKLQMVGRNFFDAKARIEIREYRMELWPGYLTSIRQ 240

Query 241 CEDHILMNAEITYKVMRCETVLDLIVKCRSSEDWEKTFENAIIGTIILTDYNNRTYRIDD 300

CEDHILMNAEITYKVMRCETVLDLIVKCRSSEDWEKTFENAIIGTI+LTDYNNRTYRIDD

Sbjct 241 CEDHILMNAEITYKVMRCETVLDLIVKCRSSEDWEKTFENAIIGTIVLTDYNNRTYRIDD 300

Query 301 VNFNCTPMSKFKIKNGESSTYCEYYKTRYGIKIKEPNQPMLVSKAKPREVRAGMTEIIYL 360

VNFNCTPMSKFKIKNGESSTYCEYYK+RYGI+I+EPNQPMLVSKAKPREVRAGMTEIIYL

Sbjct 301 VNFNCTPMSKFKIKNGESSTYCEYYKSRYGIRIREPNQPMLVSKAKPREVRAGMTEIIYL 360

Query 361 VPELCRLTGLTDDMRTNFQLMRALAEHTRVTPKLRIEKLLKFNHRLKESKDITKDLDSWN 420

VPELCRLTGLTDDMRTNFQLMRALAEHTRVTPKLRIEKLLKFNHRLKESK+ITKDLDSWN

Sbjct 361 VPELCRLTGLTDDMRTNFQLMRALAEHTRVTPKLRIEKLLKFNHRLKESKEITKDLDSWN 420

Query 421 MKLAESLITFDGRVLPMEKIYFGENRVVTAGNDADWTRSMRDHSMLSSGNFKSWSIVYLG 480

MKLA+SLITFDGRVLPMEKIYFG+NR V AGNDADWTRSMRDHSML+SGNFKSWSIVYLG

Sbjct 421 MKLADSLITFDGRVLPMEKIYFGDNRAVAAGNDADWTRSMRDHSMLTSGNFKSWSIVYLG 480

Query 481 RTKSEVNSFIHTLAKAAESLNFRVPTPRLTEVVNDRCGSFVEALDSVISMHNPQLILCIV 540

RTKSEVNSFIHTLAKAAESLNFRVPTPR++EV+NDRCGSFVEALDSVISMHNPQLILCIV

Sbjct 481 RTKSEVNSFIHTLAKAAESLNFRVPTPRMSEVINDRCGSFVEALDSVISMHNPQLILCIV 540

Query 541 PNSRADRYAAIKKKCCVDRAVPTQVVVAKHFNSKNLMAISTKIAIQINCKLGGIPWTIAN 600

PNSRADRYAAIKKKCCVDRAVPTQVVVAKHFNSKNLMAISTKIAIQINCKLGGIPWTIAN

Sbjct 541 PNSRADRYAAIKKKCCVDRAVPTQVVVAKHFNSKNLMAISTKIAIQINCKLGGIPWTIAN 600

Query 601 PLKGLMVVGYDVCHDASRKDMSYGAMVASLNPSLSRYYSSVTPHSHGEELSNDLAMNIFK 660

PLKGLMVVGYDVCHDASRKDMSYGAMVASLNPSLSRYYSSVTPHSHGEELSNDLAMNIFK

Sbjct 601 PLKGLMVVGYDVCHDASRKDMSYGAMVASLNPSLSRYYSSVTPHSHGEELSNDLAMNIFK 660

Query 661 AIIRYKENNEGNIPSMIILYRDGVGEGQIPFVYNHEVRIVKERLSEIYKDKPPKLGFVIV 720

AIIRYKENNEGNIPSMI+LYRDGVGEGQIPFVYNHEVRIVKERLSEIYKDKPPKLGFVIV

Sbjct 661 AIIRYKENNEGNIPSMIVLYRDGVGEGQIPFVYNHEVRIVKERLSEIYKDKPPKLGFVIV 720

Query 721 TKRLNTRLFLNGSNATPGTVADDCITSPDRYDFFLVSQSVRQGTVSPTSYNVIDDSTGLD 780

TKRLNTRLFLNGSNATPGTVADDCITSPDRYDFFLVSQSVRQGTVSPTSYNVIDDSTGLD

Sbjct 721 TKRLNTRLFLNGSNATPGTVADDCITSPDRYDFFLVSQSVRQGTVSPTSYNVIDDSTGLD 780

Query 781 ADKMQRLAYKMTHLYYNWSGTVRVPAQCQYAHKLAFLVSQCLHRAPNPDLDDLLYFL 837

ADKMQRLAYKMTHLYYNWSGTVRVPAQCQYAHKLAFLVSQCLHRAPNPDLDDLLYFL

Sbjct 781 ADKMQRLAYKMTHLYYNWSGTVRVPAQCQYAHKLAFLVSQCLHRAPNPDLDDLLYFL 837

Zucchini (Zuc)

>TRINITY_DN47842_c0_g2_i3 len=1252 path=[1:0-262 2:263-714 3:715-716 4:717-1251]

TATAGATTAAGTGTTACAGTTTTCGAAGATAGCCTCATTGTTCTAGGTTAGTAAAGACAATTGTTCATAAAA
GGAAATTAGTAATTATTTTTGAAAGTAGAAATGGCTGCTCCACTCCCGCGAATGTTTTCTGTGAATCCCAGA
ACAGTTAGCTGCTTACCAAGCGTTGCAAATATCTCATTCCTTACATCTTATAAATAGCATCCCGTAGGCATC
ATTATTTTAAAATTACTTTTTATAAAGTAAACTTCCACTGTTTGGGGGGCATTGGTTGGTGAAGTGAGAAAG
GTGATAGCTGTACTCTGACCTCTATGGACAGAATGGATACGAGCTCTTTGGCTGTGTAAGTTCTTTGTAACC
TTTTTCCGCCTTAGTTTGATTTAGGGAATGAATTAGTTGTGCTTAGGAATTGAGAATTGACTAGTTTAGTTG
AAGTGGATGTTTCGGATATATTTTCAATCAAATGAGTATCACAAAAGGCAGCTTATGGCTTGTAGGACTTTC
CACGTTCGGTACGGTATGCCTCGTTGGTATTGCGAATTACAAGTTTAATCAATGGATGAGAAAACAAATCA
ACAAAATAAAAAAAGACAACCAGATTGATATATTAAATAAAAACCGCTTGTTAACTTTTGGTGTGAGTAGC
GAGCAGTGTGATGGTCACATGAAGAGAGAATTCGATTGTGGCAATTTAGATTGTAACTATGGACGTTTAAG
TTATATTCTAGGATTTATTAATAATTGCGAGAGAACCCTGGACGTATGTATCTATATAATCACGGCTAAACT
GTTTGGAGATTGCATCATCAAGGCACACCAGCGCGGCGTGAAGGTGCGGGTGATAGCAGACAGCGACATG
TCCTTCTCAGCGCAGTCCTTGATCAACACCTTCAGGGCGGCAGATATCCCGACAAGGCAGAGAATGTCACC
ATTCATCATGCACCACAAGTTCGTGATTGTGGACAGCGAGGTGCTAATCAACGGGAGCATGAACTTCACGA
TGACGGGTGCGTTCTGTAATTGGGAGAACGTGATGATCACGACCCAGCCGCAGCTAGTCAATTCCTTCCAG
AACGCTTTCGATTCTCTCTGGCAAGACTTTTCCCCTCGTAATTATTCTCCTTTAGACGGGATCGTAAAAGCC
CAAGCGCCTCAGCACCAAGTCGCTAATGTATAATTGATTGTAAATATATCCTAGATATAATATACTTTTTAA
AAATAAATTTTATTGAATTTTAAAAAAAAAAAAAAA

Protein

<u>RF:</u> +2

<u>ORF: </u>464 -> 1177

<u>Length</u>: 237 aa

>lcl|ORF3_TRINITY_DN47842_c0_g2_i3:463:1176 unnamed protein product

MSITKGSLWLVGLSTFGTVCLVGIANYKFNQWMRKQINKIKKDNQIDILNKNRLLTFGVSSEQCDGHMKREFDC
GNLDCNYGRLSYILGFINNCERTLDVCIYIITAKLFGDCIIKAHQRGVKVRVIADSDMSFSAQSLINTFRAADIPTR
QRMSPFIMHHKFVIVDSEVLINGSMNFTMTGAFCNWENVMITTQPQLVNSFQNAFDSLWQDFSPRNYSPLDGIV
KAQAPQHQVANV

Conserved Domains

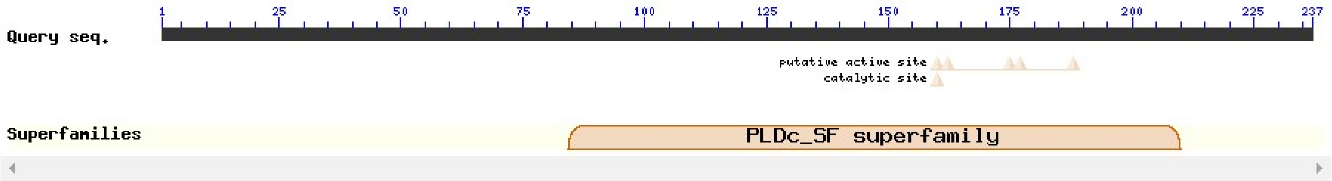

BLASTp

XP_014288409.1 PREDICTED: mitochondrial cardiolipin hydrolase [*Halyomorpha halys*]

Score:468 bits

E-value4e-175

Query 1 MSDTKGSLWLLGLSTFGTVCVIGIFNYKFNQWMRKQINKIKKDNQLDILNKNRLLTFGVS 60

MS TKGSLWL+GLSTFGTVC++GI NYKFNQWMRKQINKIKKDNQ+DILNKNRLLTFGVS

Sbjct 1 MSITKGSLWLVGLSTFGTVCLVGIANYKFNQWMRKQINKIKKDNQIDILNKNRLLTFGVS 60

Query 61 SEQCDGHMKREFNCGNLDCNYGRLSYILGFINNCEKSLDVCIYIITAKLFGDCIIKAHQR 120

SEQCDGHMKREF+CGNLDCNYGRLSYILGFINNCE++LDVCIYIITAKLFGDCIIKAHQR

Sbjct 61 SEQCDGHMKREFDCGNLDCNYGRLSYILGFINNCERTLDVCIYIITAKLFGDCIIKAHQR 120

Query 121 GVKVRVIADSDMSFSAQSLINTFRAADIPTRQRMSPFIMHHKFVIVDNEVLINGSMNFTM 180

GVKVRVIADSDMSFSAQSLINTFRAADIPTRQRMSPFIMHHKFVIVD+EVLINGSMNFTM

Sbjct 121 GVKVRVIADSDMSFSAQSLINTFRAADIPTRQRMSPFIMHHKFVIVDSEVLINGSMNFTM 180

Query 181 TGAFCNWENVMITSQPQLVNSFEDAFNSLWEDFSPHNYSPLDGIIRAQAPQHQVANV 237

TGAFCNWENVMIT+QPQLVNSF++AF+SLW+DFSP NYSPLDGI++AQAPQHQVANV

Sbjct 181 TGAFCNWENVMITTQPQLVNSFQNAFDSLWQDFSPRNYSPLDGIVKAQAPQHQVANV 237

RISC related genes

Tudor- SN

>TRINITY_DN7506_c0_g1_i1 len=4169 path=[0:0-4168]

GAACCATTGTGGTTTGGAATTAGTTGAAGAACCTCGGTATCAATTGGTCACAAAAGACAAGGTACCGATCG
ACTATCCGGTCTCACCCACGAAAGATCTTACCAGAGAGGAAGTTCTCAAATTACTTACAAAACCATATCTT
ATTGTTGATTCTTCATCCACTACTCATTCCGAAGGAACAGTCAGTCCATCACCTTCTTTAGCCAGTGAAAAA
TCTTACTCATCTCAGACTCGTGATCATCCATCAAATGCGCTGTTTTATCCTGAAGATATAGCTGTGGATTGC
TCGCTCAGTGTCCGTATCAGTCATCTAATATCTCCATCGCACTTTTATGTTCAGAGGGAGAACGCACAGAAG
AAATTACTTGAATTCCAAGCATCTTTAAGGACAGCACAGGTCCAACATTCTAGACCTCCATATAATCTAGA
AAAAGGCAAACTTTATTTGACGCTTTATACGGCTGACAGCAAGTGGTACAGAGCAAGGATAACTAATATTA
TAGATGCTAAGCAATTTGAAGTATTTTATGTAGATTATGGAAATTCTGAAGTTGTCGATAGATCAAGGATA
AGAAATATTCCCAAATCATCTTTGAACATGCCTTGTTTGGCTTATAGATGTGAGTTATCTGATTGCGTGCCT
AGGTATGGAGATGAATGGGATGCCCAAGCCATTTCTCTGATGACTGAAATAATGGACGATGACTTTGTAAC
TATTACTGTCGTTGGCAAAACAAGTGTCAGTTATTTAGTTGATCTACATAAAGTTACTGAAAATTCTTTTAT
CAATCTTCGCCAGTCATTAGTCTTTCATGAATTAGCCTCTATGTCTAAAATGCCTAGTCAGACAACTGATAT
AAGTGTGCAGCAGATTCTGACAACACCACCTCAACCTAGTTATACTGAAGGAAGTGTCTTTGAAGGGCATG
TTTCACACGTTGATTCTCCTCATACCATTTATATTCAAGATTTAACAAACTATAGGAAACTTGCACAATTGG
CCGAGGACTTGCAAAGAGCCTATAATATTAAGAATCCGCAGAAGAATGCCATCTATGATCCTAAAAAAGG
CATGCTTGTTGCTGCTCTTTATAGTAAAGATTCAAAGTGGTATCGAGCGAAAATAGTAGACTATCTTGAGGG
AAGACAAGTTAAAATATTTTTTATTGATTTTGGAAATGAAGAGACATTAATTTGTGATAATATTAAATTATT
ATCGCAAAGATTTTGCATTCTTCCTGCACAGGCTCTCAAGTGCACGCTATCTGATGTGTACCCGCTTGATGG
GACAGATTGGTGGAATGAAGACATTTGTTCTAAATTTCATGAATTGGTCTTTGATAAGCAGTTTAAATTACT
TGTTGAAAATGTAGGAAATGGGGAACTAAGTGTCGTTCTGTATGAAGTCACTAAGGAGGTGGATATATGCA
TTAATGCTAAGATTGTAAAAGAAGGTTTGGCCATGAGTACTGGCCCCAACTCTGTTCTAGTGGAGTTCCCTA
AACTTTCAGCCAATCCAATAGATCCAACTTTTCAGATTCAGGCAGTCGAAAAGAAAAAAGTTAATAAAAAA
GTGGCAAAACCATATAAATTTGCTGGTGATTCTTCTCATATGATGGACCATTTTGTTATGACGCCTGATGGT
GATTACAGAAGCAAAGTAATCGTAAAACAAATTTATAATCCTGGAGAATTCTTTGTCCTTCCTTGCTGCTTT
GAGGATATGATCAACCGGTTAAAGTTTGCCCTCCAGGGATTTTATGAAAATAGAAGATTGAACAATGGTCA
AAAGATGTGGATGATTGGTGAAAGATGTGTCATCAATTGCGACAACGTCTGGCATAGGGCCACGATATTAG
AATTTTTGCCAAATAATTTATTTGTTAAGGTTATCTTAATCGATGAAGGCTGTGAAAAAATAGTAGATTATA
GAAATATGGACATCTTGGATAACAGTTTTTCCGACATCCCAGACGGTGCCGTAAAATGTCATCTCGGTGGA
CTAGAACCTCCTCTCAAACAGTGGTCAGCACTTTCTGTGTCTGAGTTTGAAGAGTTCGTGAATAACAGAAA
AGATAGCCTCTTCATAAGCCAAATAGGGAAAATAAGAGGTAATTCTCTACCTGTCGAACTGTTTTCTAGAA
CAGCTTTAGATGTTGGCCCTACTGAACCTATGAAAGATGACTGGAAAAGTCTGAATCATTATCTTAGATTTA
TTGGATTAGCCAAAGCAGATGGAATGATCGAATGGGACAGTGATGGAAAAATTTTGAATAAGCCTGATCTC
ACTGATGATGACGGTACATCAGTTTTAGAAGCAATGTTTAGAGATTCCTTGAATTTTAATGAGGAATTCATT
GTCGAGGAAAATTCAAGTATGGCTAAAATTGTTGATGAACCTGATGTAACGATGCCTAGTACCTGGTTGCC
TCCAGAGCCACTCTCTAACTTCCAATTCATCGCAGCTCCTACTTACGTTGATGAAAATTGTATTGTGTACCTT
CACGATTTTCATAAGAGTGAAAAATTGTTGCAAGAAATCAGTACAGCTCTTGGCACAAAATATGACAATAG
CGTACCTCACCTTCACGATTCGACTATAGAAGTGGGTGATATATGCGTTGCTAAGTACCACCTCGACAATA
AATGGTACAGAGCTGTACTTTTAGGGAAATGTCTAGAAAAAAGTGAATACATAATACAATTTGTCGATTAT
GGAAATGTTGAGACGTGTAATTTGGGAGAGTTGAGGAAAATTCCTGTCGCACAGCACATTCCGATTCAATG
TTATAAGTGTTGTTTTCATAAACTGAAGCCCATTGACCCTTCAGGTATGTGGGGAGAGAGGCACATTGAATT
AATTCAATACACTATCGTTGATAAAAATTGTCGGATAAAACTGGTTCAAATCCCTGATTCTGATTTATATGG
GATTGAAGAACTTCAAATGCCAGACGGGTCTGACTACGTAGATGAAATAGTTAAACTGGGTTTCGCTGAGT
ACAAAGATACAGTCAAAACTAATCCAGAAGATGTAGTCGACCATAGCAGTGAAGTGGTAGATATGAAATC
AGCATTGGAGAGCGACTTGAAATTAGAATTGGAGAGCGAGGTGAAATTAGAATTGGAGAGTGAGAGTAGC
TCTGTTGAATTAAAACCAGTTATTGAGGCAATCATCAGTGAAGAATCACAGGCTTTTGAAAATGATGTCAA
GAAGGCTCCAATCCTACATTGGTCTTCGGTTTTAAGTACTTTATCGAAGAAAGACATGCCAAAGTTGATATA

TAAAGACTTTGAAATTCCACCCGTCGTGAAATCGTTGATAGTTGATGTCACAGCTTTCTTATCTCCTATTCA
ACTTATTTTACATGTTGAAGATGTCGAAGAAGATCCTGTGGTACTCAAGATGCTGGAAGACTTCAAGGAAC
TCTCGAATGAAATGCAAATCGAAGGACCGAACCAACCACTGTTGCAACATCCTTATAAAAACAAGGCTTGC
TGTGCCATGTTCTCTTTGGATAAGAAGTGGTACCGTGGATTCGTTTTGGAAGAATTACCCGATGACTTGTTG
TTAGTGCAATATGTTGATTATGGAAACGTAGAAGTTGTTCCAGGTTCTTGCGTTCATGAGCTCAAGGAAGA
GTGGGTTAACCTTGAAGTGCAGGGAATACTTTGCACCTTGTATAATGTTGCAATGAATGAGAATTTGAATA
CTTCTGAAATTCTACATGCCATGCAGGACTGTTTGGCAGAGGGGACAGTAAAAGCTGACATTGTAGAACGA
AACCCTGAGCTGACGGTAGAATTGGTGGTTGACGAGAAATTGGCTTATCAGCAACTTATCGATCTAAAAAT
GCTGAACTTAATCAACTAACGAGATGTGTTTTGTTGCTCTGTAATTATTTTAAAATTGAATTACCTTATCGT
TCCTAACATACAATTTTACAGTTTTATTCATATAGATATTTTAGTTTGTCAAATTGTATTTAAGATTATAAAT
TTGTTCTATAAAGTGAAATTTAAGTTTATTTTTTTGTGTAGATATATCAAATTGTATTTTTTTTACCTTTAATA
TTGTTTTTAATGTTTAAAAGTTAGTATTAATTGGTATGAAAAATTTATTTTATACTCTAAGTTCATTCCAAAA
ATCATCTATGGTTGTTCCATC

Protein

RF: +2

ORF: 599 -> 3874

Length: 1091 aa

>lcl|ORF1_TRINITY_DN7506_c0_g1_i1:598:3873 unnamed protein product

MPCLAYRCELSDCVPRYGDEWDAQAISLMTEIMDDDFVTITVVGKTSVSYLVDLHKVTENSFINLRQSLVFHEL
ASMSKMPSQTTDISVQQILTTPPQPSYTEGSVFEGHVSHVDSPHTIYIQDLTNYRKLAQLAEDLQRAYNIKNPQK
NAIYDPKKGMLVAALYSKDSKWYRAKIVDYLEGRQVKIFFIDFGNEETLICDNIKLLSQRFCILPAQALKCTLSD
VYPLDGTDWWNEDICSKFHELVFDKQFKLLVENVGNGELSVVLYEVTKEVDICINAKIVKEGLAMSTGPNSVL
VEFPKLSANPIDPTFQIQAVEKKKVNKKVAKPYKFAGDSSHMMDHFVMTPDGDYRSKVIVKQIYNPGEFFVLPC
CFEDMINRLKFALQGFYENRRLNNGQKMWMIGERCVINCDNVWHRATILEFLPNNLFVKVILIDEGCEKIVDYR
NMDILDNSFSDIPDGAVKCHLGGLEPPLKQWSALSVSEFEEFVNNRKDSLFISQIGKIRGNSLPVELFSRTALDVG
PTEPMKDDWKSLNHYLRFIGLAKADGMIEWDSDGKILNKPDLTDDDGTSVLEAMFRDSLNFNEEFIVEENSSMA
KIVDEPDVTMPSTWLPPEPLSNFQFIAAPTYVDENCIVYLHDFHKSEKLLQEISTALGTKYDNSVPHLHDSTIEVG
DICVAKYHLDNKWYRAVLLGKCLEKSEYIIQFVDYGNVETCNLGELRKIPVAQHIPIQCYKCCFHKLKPIDPSGM
WGERHIELIQYTIVDKNCRIKLVQIPDSDLYGIEELQMPDGSDYVDEIVKLGFAEYKDTVKTNPEDVVDHSSEVV
DMKSALESDLKLELESEVKLELESESSSVELKPVIEAIISEESQAFENDVKKAPILHWSSVLSTLSKKDMPKLIYKD
FEIPPVVKSLIVDVTAFLSPIQLILHVEDVEEDPVVLKMLEDFKELSNEMQIEGPNQPLLQHPYKNKACCAMFSLD
KKWYRGFVLEELPDDLLLVQYVDYGNVEVVPGSCVHELKEEWVNLEVQGILCTLYNVAMNENLNTSEILHAM
QDCLAEGTVKADIVERNPELTVELVVDEKLAYQQLIDLKMLNLIN

Conserved Domains

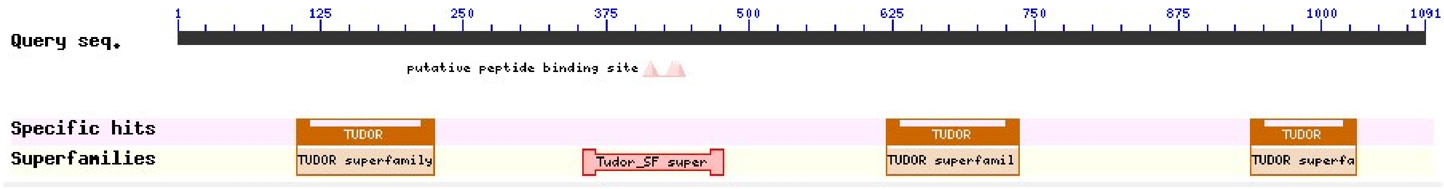

BLASTp

<u>XP_014284230.1Tudor domain-containing protein 1 isoform X2 [*Halyomorpha halys*].</u>

<u>Score</u>:1660 bits

<u>E-value</u>: 0.0

Query 480 MSCLAYRCQLSDCVPRYGDDWDAEAISLMTEIMDEDFVTITVLDKTNVAYLVDLHKVTEN 539

M CLAYRC+LSDCVPRYGD+WDA+AISLMTEIMD+DFVTITV+ KT+V+YLVDLHKVTEN

Sbjct 1 MPCLAYRCELSDCVPRYGDEWDAQAISLMTEIMDDDFVTITVVGKTSVSYLVDLHKVTEN 60

Query 540 SFINLRQSLVFHELASMTKMLSQTTDINVQQTLTTPSQHKYTEGSVFEGHISHVESPHAF 599

SFINLRQSLVFHELASM+KM SQTTDI+VQQ LTTP Q YTEGSVFEGH+SHV+SPH

Sbjct 61 SFINLRQSLVFHELASMSKMPSQTTDISVQQILTTPPQPSYTEGSVFEGHVSHVDSPHTI 120

Query 600 YIQDITNCRKLAQMTEDLQRTYNIKNKQNNAIYDPKKGMLVAACYSKDSKWYRGKIVDYL 659

YIQD+TN RKLAQ+ EDLQR YNIKN Q NAIYDPKKGMLVAA YSKDSKWYR KIVDYL

Sbjct 121 YIQDLTNYRKLAQLAEDLQRAYNIKNPQKNAIYDPKKGMLVAALYSKDSKWYRAKIVDYL 180

Query 660 EGRKVKIFFIDFGNEETLICDNIKLLSQRFCSLPAQALKCTLSDVYPLNGKDWWDEEICT 719

EGR+VKIFFIDFGNEETLICDNIKLLSQRFC LPAQALKCTLSDVYPL+G DWW+E+IC+

Sbjct 181 EGRQVKIFFIDFGNEETLICDNIKLLSQRFCILPAQALKCTLSDVYPLDGTDWWNEDICS 240

Query 720 KFHELIFDKQFKLLVDSIANGELAVVLYQVSKELDTCINAKIVKEGLAMSTGPNSVLVEF 779

KFHEL+FDKQFKLLV+++ NGEL+VVLY+V+KE+D CINAKIVKEGLAMSTGPNSVLVEF

Sbjct 241 KFHELVFDKQFKLLVENVGNGELSVVLYEVTKEVDICINAKIVKEGLAMSTGPNSVLVEF 300

Query 780 PKISGEPIDPTFQIMAVEKKRVNKKIVKPNKLDSESPLSDDFFVMMPEGVVRTKVIVKTI 839

PK+S PIDPTFQI AVEKK+VNKK+ KP K +S D FVM P+G R+KVIVK I

Sbjct 301 PKLSANPIDPTFQIQAVEKKKVNKKVAKPYKFAGDSSHMMDHFVMTPDGDYRSKVIVKQI 360

Query 840 HNPGEFYVMPTCFRERTTKLKFDLQEFYGNRRLNHVQKEWNIDDRCVVNCDGIWHRAIIS 899

+NPGEF+V+P CF + +LKF LQ FY NRRLN+ QK W I +RCV+NCD +WHRA I

Sbjct 361 YNPGEFFVLPCCFEDMINRLKFALQGFYENRRLNNGQKMWMIGERCVINCDNVWHRATIL 420

Query 900 ELLPNDFLIKVNLIDEGCEKIVDCKNIEIMDNIFAEIPDGVVKCHLGGIEPTLNTWSALS 959

E LPN+ +KV LIDEGCEKIVD +N++I+DN F++IPDG VKCHLGG+EP L WSALS

Sbjct 421 EFLPNNLFVKVILIDEGCEKIVDYRNMDILDNSFSDIPDGAVKCHLGGLEPPLKQWSALS 480

Query 960 VSEFEEFVNNRKESLFISQLGKIKNESLPVELFSRIPLEVGPTEPMKDDWKSVNHYLRFI 1019

VSEFEEFVNNRK+SLFISQ+GKI+ SLPVELFSR L+VGPTEPMKDDWKS+NHYLRFI

Sbjct 481 VSEFEEFVNNRKDSLFISQIGKIRGNSLPVELFSRTALDVGPTEPMKDDWKSLNHYLRFI 540

Query 1020 GLAKADGMIEWDSDGKILNMPE--DNDCLSILDRMFKDSMNLFEENV--ENSYMANIVEH 1075

GLAKADGMIEWDSDGKILN P+ D+D S+L+ MF+DS+N EE + ENS MA IV+

Sbjct 541 GLAKADGMIEWDSDGKILNKPDLTDDDGTSVLEAMFRDSLNFNEEFIVEENSSMAKIVDE 600

Query 1076 GGLMLKS-WLPPEPLTNLEFIAAPTYVDENGIVYLHDFHKSEKILQEISSALGTKYDNSV 1134

+ + S WLPPEPL+N +FIAAPTYVDEN IVYLHDFHKSEK+LQEIS+ALGTKYDNSV

Sbjct 601 PDVTMPSTWLPPEPLSNFQFIAAPTYVDENCIVYLHDFHKSEKLLQEISTALGTKYDNSV 660

Query 1135 PRLHDSTIEEGDICVAKYHLDNKWYRAVLLGKCLETSEYTIQFVDYGNVETCKLGELRKI 1194

P LHDSTIE GDICVAKYHLDNKWYRAVLLGKCLE SEY IQFVDYGNVETC LGELRKI

Sbjct 661 PHLHDSTIEVGDICVAKYHLDNKWYRAVLLGKCLEKSEYIIQFVDYGNVETCNLGELRKI 720

Query 1195 PVAQHIPIQSYRCCFHTVKPMDPSGMWKQRDVEIIQYYIVDKNCQIKLVKIPGSDLFGIE 1254

PVAQHIPIQ Y+CCFH +KP+DPSGMW +R +E+IQY IVDKNC+IKLV+IP SDL+GIE

Sbjct 721 PVAQHIPIQCYKCCFHKLKPIDPSGMWGERHIELIQYTIVDKNCRIKLVQIPDSDLYGIE 780

Query 1255 QLKLPEGCDYVAEMVRDGLAIYRNFFKNTKDVSDEIGDNNDEVVNTKSESET-------- 1306

+L++P+G DYV E+V+ G A Y++ K +++ D++ EVV+ KS E+

Sbjct 781 ELQMPDGSDYVDEIVKLGFAEYKDTVKTN---PEDVVDHSSEVVDMKSALESDLKLELES 837

Query 1307 --------VCDTIELKPVMETVKIEELNTIENEVKKTPILHWSSILNSLLKKEMPKLVFK 1358

++ELKPV+E + EE EN+VKK PILHWSS+L++L KK+MPKL++K

Sbjct 838 EVKLELESESSSVELKPVIEAIISEESQAFENDVKKAPILHWSSVLSTLSKKDMPKLIYK 897

Query 1359 NFEIPPVVKSLIVDVTAFLSPIQLILHIEDVEEDPVVLKMLEEFKELSNEMQIEGPNQPL 1418

+FEIPPVVKSLIVDVTAFLSPIQLILH+EDVEEDPVVLKMLE+FKELSNEMQIEGPNQPL

Sbjct 898 DFEIPPVVKSLIVDVTAFLSPIQLILHVEDVEEDPVVLKMLEDFKELSNEMQIEGPNQPL 957

Query 1419 LQHPYKYKACCAKFSLDKKWYRGFVLEELPDDMMLVQYVDYGNVEVVPGSCVHELKEEWT 1478

LQHPYK KACCA FSLDKKWYRGFVLEELPDD++LVQYVDYGNVEVVPGSCVHELKEEW

Sbjct 958 LQHPYKNKACCAMFSLDKKWYRGFVLEELPDDLLLVQYVDYGNVEVVPGSCVHELKEEWV 1017

Query 1479 NIEVQGILCTLHNVAMNENYNASEILHSVQDCLEEGTVKANIIDRNPDLSVELLIDEKLA 1538

N+EVQGILCTL+NVAMNEN N SEILH++QDCL EGTVKA+I++RNP+L+VEL++DEKLA

Sbjct 1018 NLEVQGILCTLYNVAMNENLNTSEILHAMQDCLAEGTVKADIVERNPELTVELVVDEKLA 1077

Query 1539 YQKLIDLKMFNLIN 1552

YQ+LIDLKM NLIN

Sbjct 1078 YQQLIDLKMLNLIN 1091

Translin

>TRINITY_DN9072_c0_g1_i2 len=1155 path=[0:0-332 2:333-1154]

AAATTTGACTACTTTTTTAGGTATTTATTGCCATAATAGGTATTTATGTTTCCCTAAATATTTATTAGATTCA
TATGTAAACATAAAATAAGCGCAAAGCGTTCTTATCACGCTGAACAACTTGAACTATCAACAAACAGCTGT
TTTTGTGATTCACACTCCCGCTTAAACCTACTGTGTTGATCGTTGTTTGGTGATTGATAAACTTACGGCCTGA
CAAGCATAGTATTGAACCCTTATCAGCAAAAATGACAACTAACCCTGAAGATATGTTAAGTCTTTTTGAAA
AATTTCAAAAGTATGTAGAAGAGGAGCAGGCTCGACGTGAGGTAATTCAAGAGTCAGCTCGTGAATTAGA
ACTACATGTAAGACAAATGATGACAATTCTTCAAGGAATTCATCAAGAATCTGGAATCAAAGATAGTCAAA
AAGTGGTCAACAGAGCTAGAGAACTGGTTGGTCCCTGTAGTCAAGTTTACCAGAAATTAAGCTCTGGAATC
CCACCTAAAGAGTTTTATAAATATCATGAAATATGGCGAGGTGTTACTCAAAAAGCTGCTTTTTGTATTGCA
TTGATGGAATTCCTTCAATCTGGCAAACTTGCATCCAGGGAACAGGTTGCTACTGAACTCATTTTAAAAACC
GACCAATCCGAAGGATTTCATCTAGATTTGGAAGATTATTTACATGGTCTTCTGCAGTTGGTTTCAGAATTG
TCTAGATTGGCTGTAAATAGCGCTGCTTGTGGGGACTATGAACGTCCTGTTGCGATATCACAGTTCGTTAAT
GAGCTTAATGCAGGATTTCGTTTACTTAACTTCAAAAATGATTCTCTCAGAAAACATTATGATTGTTTTAAG
TATGATTTGAAAAAAATAGAAGAGGTTGTGTATGATCTTTCTATTCGAGGACTAAAGTCTTCAGAAGTCAA
TCCAAATGTTGGTTCCTGTCAAACATCTAATATTCCAGAAAATGCTCAAATGACCTCTTAAATGTTCAGCTT
AGAAATAAGTTGCCAAAATTCATTTTGATACCAAGAAATTTTGATATAATGTAAATGAATATTCATGTGTAC
ATTTTTATCTTTTTTATTTAAATTTTCTTGTAATTTATTAAAATTCCTCTAATAAACTTCCAAATAAGTTAAAA
AAAAAA

Protein

RF: +3

ORF: 249-> 992

Length: 247 aa

>lcl|ORF3_TRINITY_DN9072_c0_g1_i2:248:991 unnamed protein product

MTTNPEDMLSLFEKFQKYVEEEQARREVIQESARELELHVRQMMTILQGIHQESGIKDSQKVVNRARELVGPCS
QVYQKLSSGIPPKEFYKYHEIWRGVTQKAAFCIALMEFLQSGKLASREQVATELILKTDQSEGFHLDLEDYLHGL
LQLVSELSRLAVNSAACGDYERPVAISQFVNELNAGFRLLNFKNDSLRKHYDCFKYDLKKIEEVVYDLSIRGLKS
SEVNPNVGSCQTSNIPENAQMTS

Conserved Domains

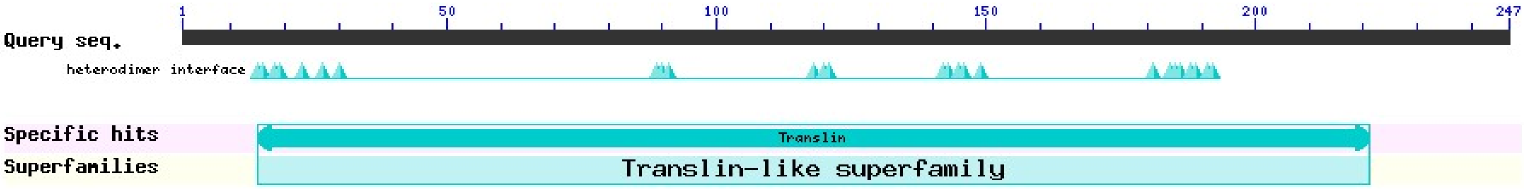

BLASTp

XP_014290495.1 translin [*Halyomorpha halys*]

Score:454 bits

E-value: 3e-169

Query 1 MTTSTEDMLSLFEKFQKYVEEEQARREVIQESARDLEQHIRQMMTIIQGIHQESGIKDSP 60

MTT+ EDMLSLFEKFQKYVEEEQARREVIQESAR+LE H+RQMMTI+QGIHQESGIKDS

Sbjct 1 MTTNPEDMLSLFEKFQKYVEEEQARREVIQESARELELHVRQMMTILQGIHQESGIKDSQ 60

Query 61 KVISRAKDLVGPCGQVYQKLSSGIPPKEYYKYHEIWRGTTQKAVFCIALIEFLQSGKLAS 120

KV++RA++LVGPC QVYQKLSSGIPPKE+YKYHEIWRG TQKA FCIAL+EFLQSGKLAS

Sbjct 61 KVVNRARELVGPCSQVYQKLSSGIPPKEFYKYHEIWRGVTQKAAFCIALMEFLQSGKLAS 120

Query 121 REQVAAELTLKIDQAEGFHLDLEDYLHGLLQLVSELSRLAVNSAACGDYDRPVAISQFVN 180

REQVA EL LK DQ+EGFHLDLEDYLHGLLQLVSELSRLAVNSAACGDY+RPVAISQFVN

Sbjct 121 REQVATELILKTDQSEGFHLDLEDYLHGLLQLVSELSRLAVNSAACGDYERPVAISQFVN 180

Query 181 ELNAGFRLLNFKNDSLRKHYDCFKYDLKKIEEVVYDLSIRGLKSSENKSSIDVCPTSNIP 240

ELNAGFRLLNFKNDSLRKHYDCFKYDLKKIEEVVYDLSIRGLKSSE ++ C TSNIP

Sbjct 181 ELNAGFRLLNFKNDSLRKHYDCFKYDLKKIEEVVYDLSIRGLKSSEVNPNVGSCQTSNIP 240

Query 241 ENSEMAA 247

EN++M +

Sbjct 241 ENAQMTS 247

<u>Similar to translin associated factor-X (TRAX)</u>

>TRINITY_DN5790_c0_g1_i5 len=1675 path=[2:0-106 5:107-1674]

AAACAAGGGTTACTGTTAACAAATTTATTTTAAATATCTATCATAATTTTTACTGTTTGGTCTTGGCATACTC
TTCAATATCTAAAAATATAATTTTCTTAATATGGGTTACGATGAGAAAAAGAGAAAATTTCAGAAACCACC
TCAGGAGAAAAAAGCAGTTCTCCAATACAACAAAACTCCGGTTACTGAAGCATTTGTAAAATATGCAATGG
AATTGAATTCGAAACAAGATAAATCTGAAAGAATTGTAAAACTTAGCAGAGATGTAACTATTGCGAGTAAA
CGTGCTATATTTGCCCTCCATGCTTTGATGAGATCTGGAAATTATGAAGAAGACTTGTCAAAAATTACTGAA
ACTTTGAAAACAGTGAGAGAAGATCTATTAAAGAAGATTGCATTTGAACTCGCTGGTGATGATCCCTATCA
ATTTTGCAGAAATTATACTGCAGGACTACAAGAGTACATAGAAGCTATAACATTTTATCATTTTCTAACTAA
AAATGGGCTGTTTAATCTTGAAGTAATTCAAGATGAATTAACGTTCGATAAACAAGTTATGGATCAAGGAG
AGCCTCCTCGTGAGAAAGTTCCGCTACTTGTATCTTTCTTCGATTACATTCTTGGAGTACAGGATCTCACCG
GTGAAATTATGAGGCACTGCATCAACAGCTTTAGTGCTCGGAATTTAGAAGCAGGCAACAAGGATTGCAAT
TTCGTCAAAAACCTCTATATGGGAATGGTATTGTTGAATTCTTATCGAAACGTTATGGGCCTAGGGGTGCAA
GGGAGAGATTTTGCTAAGAAAATGTCTGTAACTCTTCAATCATTGAAAAAAATGGAAATGGCCTGCTATGC
TGCACATATTCGGGGATCAGAAGCTGTTGATTTTGGATTCAATACAGAGGAATTCGAAAGTTATAATTTTGG
TGATTAATTTGTAATATAAATTGAAAACTGATTTTTTCTTCATATTATTAAGGCTATGTGATTATTTATTTTG
TACAAAATCGTGGTTTTTATTTTTATATCTTACCTTTTCCTTGATTTTATTTATTTTTAGTTCCTTGGAATTGTT
CAGGTCTAAATTTTTATCAGAAAAAAAAAAAAAATCCTTCCAATTTAAAACTTTTATAGGGGATTAGTTGAT
TCCGGATGCATTCATCAAAACGCTTAAGTAGAAAACTAAATTTATTTGTGTCAACATTTTAGCCTATATTAT
AACCCTTCTTCAGGGCGATTCAAGGAGGAAGATGACAAAAAACCAACATTGGCCCAAATAAATTAAGTTTT
CCACTGAAGTGTTTCGTTGACTGCACCCGGAATCAACTAATCTCCTATATATACACACTATCAACCATTCTC
ACTTCACTTAAAACTTTTATAATGATCAAAACAGTACTAATTTAAAAGTAAGATATTTTAATTTTTTTTTTG
TTATTATTATTCATCAGTCCATTACCTAGAGTAGTGTTTTAAAAATAATTCTGTTTTGGTGTTCTATGCAGAT
TATTTTTAGAACCCAAAACAAATGTTCAGCTAAAACATTTCTTTGAACTTTCGTATACCAGTATTATTTTTAA
CTGATTTCAATTTTTCATTCCTTTTATTTTATGTATAATTTTTAAAATTTTGTTAACATTATAATTTATATAAT
GTGATGATTATTTCACT

Protein

<u>RF:</u> +2

<u>ORF:</u> 104 -> 937

Length: 277 aa

>lcl|ORF1_TRINITY_DN5790_c0_g1_i5:103:936 unnamed protein product

MGYDEKKRKFQKPPQEKKAVLQYNKTPVTEAFVKYAMELNSKQDKSERIVKLSRDVTIASKRAIFALHALMRS
GNYEEDLSKITETLKTVREDLLKKIAFELAGDDPYQFCRNYTAGLQEYIEAITFYHFLTKNGLFNLEVIQDELTFD
KQVMDQGEPPREKVPLLVSFFDYILGVQDLTGEIMRHCINSFSARNLEAGNKDCNFVKNLYMGMVLLNSYRNV
MGLGVQGRDFAKKMSVTLQSLKKMEMACYAAHIRGSEAVDFGFNTEEFESYNFGD

Conserved Domains

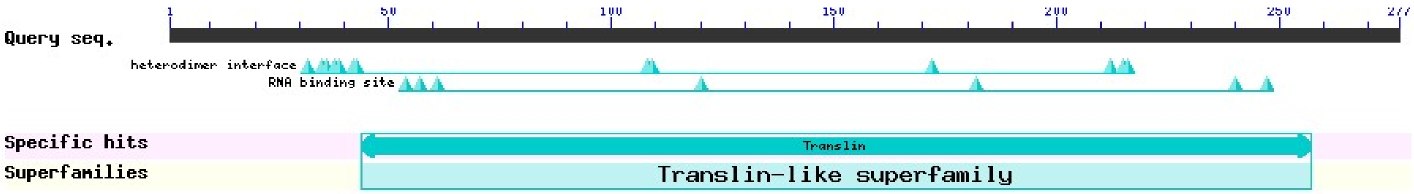

BLASTp

XP_014289754.1 translin-associated protein X isoform X2 [*Halyomorpha halys*]

Score:513 bits

E-value: 0.0

Query 1 MGYEEKKKKYQKPPQPKKVCLEYDKTPVTEAFEKYAMELNSKQDKSERIVKLSRDVTIAS 60

MGY+EKK+K+QKPPQ KK L+Y+KTPVTEAF KYAMELNSKQDKSERIVKLSRDVTIAS

Sbjct 1 MGYDEKKRKFQKPPQEKKAVLQYNKTPVTEAFVKYAMELNSKQDKSERIVKLSRDVTIAS 60

Query 61 KRAIFSLHALMRSGNYEEDLAKITETLETVRENLLKNIALELAGDDPYQFCRNYTAGLQE 120

KRAIF+LHALMRSGNYEEDL+KITETL+TVRE+LLK IA ELAGDDPYQFCRNYTAGLQE

Sbjct 61 KRAIFALHALMRSGNYEEDLSKITETLKTVREDLLKKIAFELAGDDPYQFCRNYTAGLQE 120

Query 121 YVEAITFYHFITKGGLFNIEVIQDELTFQKKVMEQGEPPQEKVPLLVPFFDYILGIQDLT 180

Y+EAITFYHF+TK GLFN+EVIQDELTF K+VM+QGEPP+EKVPLLV FFDYILG+QDLT

Sbjct 121 YIEAITFYHFLTKNGLFNLEVIQDELTFDKQVMDQGEPPREKVPLLVSFFDYILGVQDLT 180

Query 181 GEIMRHCINCFSARNLEAGNKDCNFVKNLYTGLLLLNTYRNAMSHGMQGRDFTKKMSVTL 240

GEIMRHCIN FSARNLEAGNKDCNFVKNLY G++LLN+YRN M G+QGRDF KKMSVTL

Sbjct 181 GEIMRHCINSFSARNLEAGNKDCNFVKNLYMGMVLLNSYRNVMGLGVQGRDFAKKMSVTL 240

Query 241 QSLKKMEMACYAAHIRGSEALDFGFNTDEFESYNFGD 277

QSLKKMEMACYAAHIRGSEA+DFGFNT+EFESYNFGD

Sbjct 241 QSLKKMEMACYAAHIRGSEAVDFGFNTEEFESYNFGD 277

Armitage

>TRINITY_DN4232_c0_g1_i1 len=3618 path=[1:0-184 2:185-309 4:310-337 5:338-533 6:534-537 7:538-567 9:568-3617]

CTGTCATTGACAATAAGTACTATTTTCATCTAAAAAATGCCGTTGCGCCTGCCTTAAAATTAAATGATTTAG
TGACCTGCACCTTATATACTAATGAATTGTATACTTTGGTAAAAAATGTTCAGAAAGGTGAAGGAACTGAA
GAAGAAACAGAAAGCTTCATTGAACCTGTCACTAAAATAGAAAGGAAACTTGATGGCATGACTGAATTGA
AAATATCTGGAAAGATAATTGATAAAGTAGCCAGAAAATTGACTGTTGAAGTTGATTATGAAGATCCTAAA
GTTTTAAATTTTAATTTAGACAATGTCAGCGCAGAGTTTATTCCTTTAACAGGTGATAATGTAGAGTTGGAA
TGCCTCTCCCCAGTTGAGGATTGTAAGAAAATTATTGTAGAAAAAGTAAATCCCAAGAAAACAGCTGTCAG
AATGGGGACTGTGACCGATTGGAATGGTGCACGTGGTGTAATTAGTAATTTTGCAGAATTTACATCGAATA
CATGTGAGCCAGGTTACCAACCAACCAAAGGAGATAAGGTTTTGGCAAGTGTTATTAGAAGTGATTCAGAT
GCTGGGTACAAATGGCGAGCGATTCAAGTGGTCGCTCAACTACGCCAGTTGGGTGTTCGTCTGTCCAAAAA
AGCTGAAGAAGCCCGACGGGAGCTATTGCTTGACAAGCAAGGAGTCTTTATTTCTGTCTGTTCCAAGTTTAC
TGTTAATCTAAATTCGTCTCTTGAGTTTGTTGTTGAGGTAGTGAACAAGAGCTGCATCGAAAAAGATCTTGA
AAGAGTGAGCCTTTTGTCGAAGGCTGATTTATGCCAGATAACTTTGATAAACCCGGAGGATCCTGCTATGA
CCATACAAGCAGGGGAGGTGGTCCCCTTCACATTCAGAATAGACGGAAAGTTTGTCGGGCGATCTTCGGAA
CAATATGTTTGGGTGTTTTCAGAATTCACAATCGGAAGAATATTCGATTTGGAAGTAGTCGATTTATCTCTA
GCCGAAGCTGAACAGGAATGTTCGACTTCACAATCACTTATTCCTAGGAAAGAAGTTACTGCAACACAACT
GTTGGATCGTGCTTCTGGTGTTATTATGCCTGGACGCAGATCTTTTTCACCTGCTGCATTTGTCCCTGTGAAA
CTTGGACAGTTCCCGTTGTCAGAAAGGATTCTTATATCAGCTCTACCTGATGTAAAAATGAACCTAGATGAT
TTGATGATACGTGTTGAGAAGGTCTTACCTTGCTTAAAAGAAGATTTATCTCCAAAAACATATTTAGCTCGT
ATGCATGGCCTTCTTTATCTCGAGGAAGTGGCTATGCTTAAGCAGGTTGGTGAACTTCAAATGGACAGAGC
ATCGTTCAGAAAAGAAAGAGATTGCCTGGTGTTAGACGTGCCACCCCTTTCACCGTACACAAGTAAACTAG
TCGCTGGGGACATGCTGATAGCGTCCCTACCAGGGGCAGCTGATGAAAGGAAGTACGAGGGAGTCATTCA
TCGGGTAACCCAGACAGAGCTGTGGCTTCAGTTCGCCAAAGATTTCCATGACTCTTACGGAGAAGGAGTAA
AGTACTCAGTGTCTTTCGTGACATCAAGAGCTTCCCTGCGTCGGATGCATCAGGCTATTAACCTTGCTGCAA
AACACCTTGGCTACGGTTGGCTCTTTCCTACGGGAGTGACGCCTAGGTTACCTCAAGTGGTTGTTGAAGAG
GAAATCGAAGAGAACGATTCTATGAAGAGTACGAAGAAAGAGAGGGGTTCCAAAAAAGTCAATAGTTTAT
TGGCGCTTGAAAGTAAAGAGTTAGCTTACAGCCCTGATCCAAGGAAAATATCCATTGCCGGCCAGGCCAAT
CTAAGTATTGACCATAAGTTCCAGGCTTCTTTATTAAAAGCAGGATACCGTGGTAGAGGCAGAAGATACAA
TAACCGTGATATTCAATGGAACAGGCAAGGTAGTGGTTATGGTATGCATTGTACCATCGACAACGAGGTTC
GTGTCAATATTGGCTTAGAGAGAATTAAAAAAATTAGATGGTTCAACAAGGGTCTCAATAGGCAGCAAAA
AGAAGCAGTAAAGAACGTGCTCTTAGGAGAAGCAAGGCCTTTGCCATACGTTATATTTGGTCCTCCAGGCA
CTGGTAAAACAGTCACTGTTGTTGAAACCATTTTGCAGTTGCATGCTCTCATTCCCGAAAGTAGATTACTTG
TTGCCACTCCCTCCAATTCTGCTGCAGACCTTATTACTGAACGTCTGTTGGATGCTGGTGATTTAGAACAAG
GGGATTTGTTGAGGATGGTTGGTTATCATTATTTAGAACAAGGAAGAATCGCTGCATCTATTGTGCCCTATG
CAGCTGTTCCAGATGTTAAAGCTATAAATGTTGCTGGTCTTTCCGGTTCTTCTCACGAAGGAGTACAAATGT
GTGGGAGAGAACTTCTTGGCCAACACCGAGTTACCGTGGGTACCCTTGGCTGCCTTGGATTGCTCTACAAC
ATGGGCTTCCCGAGGGGCCATTTCACACACGTCATTGTTGATGAAGCTGGTCAGGCTACTGAACCAGAGCT
CCTCATTCCCATGGTGTTTTTGCACATGGAATATGGGCAGGTGGTTCTAGCTGGTGATCCTCTGCAGCTTGG

GCCAGTTGTCACTTCACGCCTTGCATCAAGGTGTGGGTTACAGGATTCTCTCCTTGCAAGATTTCTTAACCG
ATTCCCATATACCAGAGATCCTCATGGCTTTCCAGAGAGTTCAGGCTATGATCCACGCTTAGTCACCAAACT
GGTGAACAATTACCGATCACTTCCGACTATACTGGAATTACCTAGCATGCTTTTTTACGATAATGATCTTAT
TCCAAATGTATCCCAGGATTCCAGTGAAGAAGCAGCTTTATTACGAGCATTGGCCCCGTTGCTCCCTTGTCG
TATATTCGGTGGTCATGCACCTCCTCTTCTGTTTCATGGAGTGCGTGGCACTAACTGCCAGGAGACTGAATC
ACACTCCTGGTATAATCCTCAAGAAGTATTTCAAGCATTTGTTTACTTGAACTTATTGTATAAAGCTGGACT
TCGCCCTGATCAAGTTGGAATCATCACTCCCTATCAACTCCAGTCCAATAAAATCAGATTTATGTTGGAACG
AATTAATATAGAACCTCCAAAAGTTGGTTCTGTGGAAGAATTTCAAGGGCAAGAAAAGATGGCAATTATTG
TTAGTGTCGTTCGAAGTAGCCCAGATTTGATCAGCTACGACATGCAAAGAGCGCTCGGCTTCGTTGCTAAT
GCTAGAAGATTGAACGTTGCGTTATCAAGAGCAAGGGCCATTTTGATTATTCTAGGAAATCCTCACCTGCTC
TACCTCGATATGCATTGGAGAAGTGTTTTGAGACATTGTGTTAAGAAGAAATTTTATACTGGTTGTGATCTT
CCACCAGAATTTACTTTAGAAAGAACAAATGCTGACCCATCACTCAACTTGGAGCTGTAACTACATTTTATT
TTAAAAAATATGTATTTTTTATCAAGATTTACTGTTGTTTCATGTTTTATATTAGTCTATGCTAATGTTTGTAT
TAAAAATAACCAATAAATTATGTTTTATATTGGTTTAAAAAAAA

Protein

<u>RF:</u> +3

<u>ORF:</u> 201 -> 3488

<u>Length</u>: 1095 aa

>lcl|ORF7_TRINITY_DN4232_c0_g1_i1:200:3487 unnamed protein product

MTELKISGKIIDKVARKLTVEVDYEDPKVLNFNLDNVSAEFIPLTGDNVELECLSPVEDCKKIIVEKVNPKKTAV
RMGTVTDWNGARGVISNFAEFTSNTCEPGYQPTKGDKVLASVIRSDSDAGYKWRAIQVVAQLRQLGVRLSKK
AEEARRELLLDKQGVFISVCSKFTVNLNSSLEFVVEVVNKSCIEKDLERVSLLSKADLCQITLINPEDPAMTIQAG
EVVPFTFRIDGKFVGRSSEQYVWVFSEFTIGRIFDLEVVDLSLAEAEQECSTSQSLIPRKEVTATQLLDRASGVIMP
GRRSFSPAAFVPVKLGQFPLSERILISALPDVKMNLDDLMIRVEKVLPCLKEDLSPKTYLARMHGLLYLEEVAML
KQVGELQMDRASFRKERDCLVLDVPPLSPYTSKLVAGDMLIASLPGAADERKYEGVIHRVTQTELWLQFAKDF
HDSYGEGVKYSVSFVTSRASLRRMHQAINLAAKHLGYGWLFPTGVTPRLPQVVVEEEIEENDSMKSTKKERGS
KKVNSLLALESKELAYSPDPRKISIAGQANLSIDHKFQASLLKAGYRGRGRRYNNRDIQWNRQGSGYGMHCTID
NEVRVNIGLERIKKIRWFNKGLNRQQKEAVKNVLLGEARPLPYVIFGPPGTGKTVTVVETILQLHALIPESRLLV
ATPSNSAADLITERLLDAGDLEQGDLLRMVGYHYLEQGRIAASIVPYAAVPDVKAINVAGLSGSSHEGVQMCG
RELLGQHRVTVGTLGCLGLLYNMGFPRGHFTHVIVDEAGQATEPELLIPMVFLHMEYGQVVLAGDPLQLGPVV
TSRLASRCGLQDSLLARFLNRFPYTRDPHGFPESSGYDPRLVTKLVNNYRSLPTILELPSMLFYDNDLIPNVSQDS
SEEAALLRALAPLLPCRIFGGHAPPLLFHGVRGTNCQETESHSWYNPQEVFQAFVYLNLLYKAGLRPDQVGIITP
YQLQSNKIRFMLERINIEPPKVGSVEEFQGQEKMAIIVSVVRSSPDLISYDMQRALGFVANARRLNVALSRARAIL
IILGNPHLLYLDMHWRSVLRHCVKKKFYTGCDLPPEFTLERTNADPSLNLEL

Conserved Domains

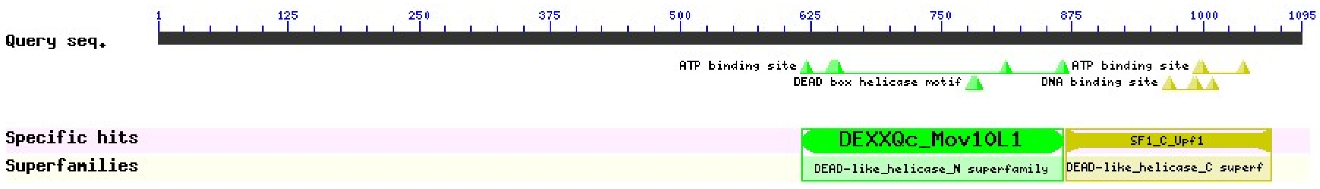

BLASTp

XP_014289817.1 probable RNA helicase armi [*Halyomorpha halys*]

Score:2098 bits

E-value: 0.0

uery 133 MTELKISGKIIKKEARKLTVEIDYEELEVLIFNLDNVSAEFIPLRGDIVELECLSPIDDC 192

MTELKISGKII K ARKLTVE+DYE+ +VL FNLDNVSAEFIPL GD VELECLSP++DC

Sbjct 1 MTELKISGKIIDKVARKLTVEVDYEDPKVLNFNLDNVSAEFIPLTGDNVELECLSPVEDC 60

Query 193 QKIIVEKVNPKKTAVKMGTVTDWNATRGVISNFAEFTSNTCEPGYQPTKGDKVLASVIRS 252

+KIIVEKVNPKKTAV+MGTVTDWN RGVISNFAEFTSNTCEPGYQPTKGDKVLASVIRS

Sbjct 61 KKIIVEKVNPKKTAVRMGTVTDWNGARGVISNFAEFTSNTCEPGYQPTKGDKVLASVIRS 120

Query 253 DSEAGYKWRAIQVVAQLRQLGVRLSKKAEEARRELLLDKQGVFISVCSKFTVDLNSSLEF 312

DS+AGYKWRAIQVVAQLRQLGVRLSKKAEEARRELLLDKQGVFISVCSKFTV+LNSSLEF

Sbjct 121 DSDAGYKWRAIQVVAQLRQLGVRLSKKAEEARRELLLDKQGVFISVCSKFTVNLNSSLEF 180

Query 313 VVEVVNKSCIEKDLERVSLLSKADLCQITLINPTDPAMTIQAGQVVPFTFRIDGKFVGRS 372

VVEVVNKSCIEKDLERVSLLSKADLCQITLINP DPAMTIQAG+VVPFTFRIDGKFVGRS

Sbjct 181 VVEVVNKSCIEKDLERVSLLSKADLCQITLINPEDPAMTIQAGEVVPFTFRIDGKFVGRS 240

Query 373 SEQYVWVFSEFIIGRIFDLEVVDLSLAEAEQECSTSQSLIPRKEVTATQLLDRASGVIMP 432

SEQYVWVFSEF IGRIFDLEVVDLSLAEAEQECSTSQSLIPRKEVTATQLLDRASGVIMP

Sbjct 241 SEQYVWVFSEFTIGRIFDLEVVDLSLAEAEQECSTSQSLIPRKEVTATQLLDRASGVIMP 300

Query 433 GRRSFSPAAFVPVKLGQFPLSERILISALPDVKMNLDDLMIRVEKVLPCLKEDLSPKTYL 492

GRRSFSPAAFVPVKLGQFPLSERILISALPDVKMNLDDLMIRVEKVLPCLKEDLSPKTYL

Sbjct 301 GRRSFSPAAFVPVKLGQFPLSERILISALPDVKMNLDDLMIRVEKVLPCLKEDLSPKTYL 360

Query 493 ARMHGLLYLEEVAMLKQVGELQMDRASFRKQKDCLVLDVPPLSPYTSKLVAGDMLIASLP 552

ARMHGLLYLEEVAMLKQVGELQMDRASFRK++DCLVLDVPPLSPYTSKLVAGDMLIASLP

Sbjct 361 ARMHGLLYLEEVAMLKQVGELQMDRASFRKERDCLVLDVPPLSPYTSKLVAGDMLIASLP 420

Query 553 EAADERKYEGVIHRVTQTELWLQFAKDFHDSYGEGVKYSVSFVTSRASLRRMHQAINLAA 612

AADERKYEGVIHRVTQTELWLQFAKDFHDSYGEGVKYSVSFVTSRASLRRMHQAINLAA

Sbjct 421 GAADERKYEGVIHRVTQTELWLQFAKDFHDSYGEGVKYSVSFVTSRASLRRMHQAINLAA 480

Query 613 KHLGFGWLFPTGVIPRLPQVVVEEEIEESGSTKTVRKDRGSKKVNSLPALESKDLLYSPD 672

KHLG+GWLFPTGV PRLPQVVVEEEIEE+ S K+ +K+RGSKKVNSL ALESK+L YSPD

Sbjct 481 KHLGYGWLFPTGVTPRLPQVVVEEEIEENDSMKSTKKERGSKKVNSLLALESKELAYSPD 540

Query 673 PRKISIAGRTNLSIDHKFQASLLKAGQRGRARRYNNRDIQWNRQGSGYGMHCTIDNEVRV 732

PRKISIAG+ NLSIDHKFQASLLKAG RGR RRYNNRDIQWNRQGSGYGMHCTIDNEVRV

Sbjct 541 PRKISIAGQANLSIDHKFQASLLKAGYRGRGRRYNNRDIQWNRQGSGYGMHCTIDNEVRV 600

Query 733 NIGLERIKKIRWFNKGLNRQQKEAVKNVLLGEARPLPYVIFGPPGTGKTVTVVETILQLH 792

NIGLERIKKIRWFNKGLNRQQKEAVKNVLLGEARPLPYVIFGPPGTGKTVTVVETILQLH

Sbjct 601 NIGLERIKKIRWFNKGLNRQQKEAVKNVLLGEARPLPYVIFGPPGTGKTVTVVETILQLH 660

Query 793 ALIPESRLLVATPSNSAADLITERLLDAGDLEQGDLLRMVGYHYLEQGRIAAAIVPYAAV 852

ALIPESRLLVATPSNSAADLITERLLDAGDLEQGDLLRMVGYHYLEQGRIAA+IVPYAAV

Sbjct 661 ALIPESRLLVATPSNSAADLITERLLDAGDLEQGDLLRMVGYHYLEQGRIAASIVPYAAV 720

Query 853 PDVKAINVAGLSGSSHEGVQMCGRELLGQHRVTVGTLGCLGLLYNMGFPRGHFTHVIVDE 912

PDVKAINVAGLSGSSHEGVQMCGRELLGQHRVTVGTLGCLGLLYNMGFPRGHFTHVIVDE

Sbjct 721 PDVKAINVAGLSGSSHEGVQMCGRELLGQHRVTVGTLGCLGLLYNMGFPRGHFTHVIVDE 780

Query 913 AGQATEPELLIPMVFLHMEYGQVVLAGDPLQLGPVVTSRLASRCGLQDSLLARFLNRFPY 972

AGQATEPELLIPMVFLHMEYGQVVLAGDPLQLGPVVTSRLASRCGLQDSLLARFLNRFPY

Sbjct 781 AGQATEPELLIPMVFLHMEYGQVVLAGDPLQLGPVVTSRLASRCGLQDSLLARFLNRFPY 840

Query 973 TRDPHGFPDSSGYDPRLVTKLVNNYRSLPTILELPSMLFYDNDLIPNVSEDSSEEAGLLR 1032

TRDPHGFP+SSGYDPRLVTKLVNNYRSLPTILELPSMLFYDNDLIPNVS+DSSEEA LLR

Sbjct 841 TRDPHGFPESSGYDPRLVTKLVNNYRSLPTILELPSMLFYDNDLIPNVSQDSSEEAALLR 900

Query 1033 ALAPLLPCRIFGTRAPPLLFHGVRGTNCQETESHSWYNPQEVFQAFVYLNLLYKAGLRPD 1092

ALAPLLPCRIFG APPLLFHGVRGTNCQETESHSWYNPQEVFQAFVYLNLLYKAGLRPD

Sbjct 901 ALAPLLPCRIFGGHAPPLLFHGVRGTNCQETESHSWYNPQEVFQAFVYLNLLYKAGLRPD 960

Query 1093 QVGIITPYQLQSNKIRFMLERINIEPPKVGSVEEFQGQEKMAIIVSVVRSSPDLISYDMQ 1152

QVGIITPYQLQSNKIRFMLERINIEPPKVGSVEEFQGQEKMAIIVSVVRSSPDLISYDMQ

Sbjct 961 QVGIITPYQLQSNKIRFMLERINIEPPKVGSVEEFQGQEKMAIIVSVVRSSPDLISYDMQ 1020

Query 1153 RALGFVANARRLNVALSRARAILIILGNPHLLYLDMHWRSVLRHCVKKKFYTGCDLPPEF 1212

RALGFVANARRLNVALSRARAILIILGNPHLLYLDMHWRSVLRHCVKKKFYTGCDLPPEF

Sbjct 1021 RALGFVANARRLNVALSRARAILIILGNPHLLYLDMHWRSVLRHCVKKKFYTGCDLPPEF 1080

Query 1213 TLERTNADPSLNLE 1226

TLERTNADPSLNLE

Sbjct 1081 TLERTNADPSLNLE 1094

Homeless (spindle-E)

>TRINITY_DN8326_c0_g1_i2 len=4080 path=[0:0-632 2:633-4079]

CCAATGAGCGAGGGTGGCCGTTAGGCACCATTGTTGGATATCAGATCGCTTTGGAACGTAACTGTTCTCAA
GACACCCGCCTCATGTATTGTACAACGGGCGTTCTTCTTCAGCAGCTTGTGAAGAAGCAGTCGCTGTCAGAT
TACACTCATATCATTGTAGACGAGGTTCATGAAAGAGATAATGAAACTGATTTCCTTCTGATCATATTGAAA
AAACTGCTGAGGAGTGCTCAGTGCAGAACAAAGGTTATTTTGATGTCGGCAACAATGAATGTGAACCAATT
TTCAGATTACTTTTCAAGAACTGTAAATGGTCTTCCTATTGAGCCTCCTATTGTCGAATTAGCCCACATCGC
CAAATTCCCAGTTCAGTATTACTACCTCGACAGCCTTGCTAATAGGCTGCCAGGTATAAAGGTACCTGATAT
TGATATTAATACTCCTTCGATTTCAACTTATACCTACGACTTAGCTAGAGCTCTTATCGAGGTCTTCAGAGT
AATAGATGAGCATGAACATCATGATACTAATTTCATTGGATCTGTTCTAATATTTTTACCTGGCATCGCTGA
AATTGAAACAATGTATTCTAGACTCAATAATTACAAGGGTTCTGCTGAAAAGTGGTGGCTGTGCCCACTCC
ACTCATCTGTCACTTACGATGAACAAATGAAAGCCTTTCAGCCCGCTCCTAAGGGTCATCGTAAGATCATCC
TTGCCACCAACATTGCTGAAAGTTCAATTACTGTGCCTGACATTAAGTATGTGATAGATTTCTGTCTTGCGA
AGCAACAGGTACTGGAACCTGAAACAAGCTACTCTTGCCTACAGCTGACCTGGGTCTCCAAATCACAAGGG
ACTCAGAGAGCCGGCAGGGTTGGAAGAGTCATGCCAGGCCGTGTTTACCGTCTCATCCCAAAGGATTATTA
TATGGAGTTACCCGAGGATATAACTCCAGAGATTATGAGATGCCCTCTTGATCAGCTGGTCTTGAAGGCAA
AGCAGCTGAGGATGGGTAACCCAGCAACACTGCTAGGATTGGCCATTGATCCTCCCGATCTATCAAACATA
CATAAGACTATCCTTCATTTAAAAGAGGCTGGTGCTCTCCTCCTCACCTCTAATGGAGAATACAAAGATAA
CGATGGCGACTTGACTTTTATCGGTCAAATCATGGCATCGCTTCCCCTGGACATCCATCTTTCCAAGTTGAT
AATCCTCGGTCATATGTTTTCTTGCCTCTCAGACGCCATAGTTATGGCTTGTGCAATGTCCGTAAAGAGTAT
CTTTAGCACACCTTTCAGGCAGCAGTTGGAAGCTTACAACTCAAAGCTTACCTGGGCGGATTCCTCGTGTAG
CGATCCCATCGCTTATCTTCACGCTTATTCGCTTTGGAAATTCAAATCGAAAATGGGTTACTTCAAGAGATC
AGGTGGAGAGAGCGAACTTGATTGGTGTCGAAAATACTTCATTCAGGGAAATCTTATCAGAGAGGTTTCTC
GATTAGAAAATGAAATCATACAACGTTTGAGGGGATTAGGGGTTGAAGAAATGAAAGGAGAAACGAGTGT
TTCTTGGACTGAAGCAGAAAAGCCTATTATTCTTAAGCTTATTATTGCTGGAGCATTTTATCCAAACTATTTC
ATTCCCTTTGGCTCTGATGAGAAGGATTCTGTTAAAATTCTTGGTGGGCGAGATCCACACTCAACAGTATAT

TTAACTGGATTGCCAAACAACCAACCTGGTCCCCTCTACACTCATTCTATACGAAGCCATTTTCAACATTGC
GGATCAAATATTGAAGTTTCATTTGATGGTAGCAGCAAAATTTACCTAACTTTTGGAAGTAGTGTATCTGCT
AACCGAGAACCTGAAAAAACTCCTTTGATGCCAGGAAAAACTAGCATGGCTGTTTACAGGAGCATTAAATT
GCGCCAGCTTCAGGTCCCGATCACTGTACCAGTACTATTACCTCATGAAGCCCGTAAAAGGGCTCAAGAAG
TTTTTGGTGATAGGTTGACACCGAGCTTGTTTAATGTTAACAAAAGGAAAGCAGTAACACCTACTAAAAAG
ACCTATATGCCCTCTCTTGCAACAAGCATTCTTCCAGTTGTCATCACTAACGTTGAAAGTCCCTCCAAGTTTT
GGGTGAACATAAACGAGCCAGTAAACACTAACAGGCTTTTTTGGATCCAATCTTCACTTAACAAAATAAGC
GAACCCCTGCCGCCATATACGGGTCCCTATGACGATGGCACTCCTTGTATTGCTCCGTACAGAGATAAGAA
AGGACCTCCGGAATATTACAGAGCTCGTATCATCAGCAGCATTGAGATAGATCAAAGGGGAACAGTTGTA
AGAGTTCAGGTGTACTTTGTCGATTATGGTAATCAAGAATCTGTCTACACCAAGGATCTGAGAACCTATCCT
GAATCTTTAAACAACGTGAAAGAGGAGCCAGATTTAGCGCTGGAAGCAAGCCTTGCCGAAATTGGTCCGTG
TTTTGCTAAGAACCCTAAGGGTGGTTGGGCCCCTGAGCACTGCAGGGAATTTTATAAGTTCTTAAAGGATA
ATACTGGTGTAGCCAAAATTTTTTCTGTCGTCAATAATGTAATGGCAGTTACATTGTTTTCATCAGAGCTAA
TGAAGAGAAAAACGGAGGATATTCCTTTTGAAATGTCCTTTAATTACTACCTGATAAAGAATGGATTTGCT
GAGCCAGTTGACGAGCCTTACCTTTCAAGAGAAAATCACATGTTACGAGAATCTAGCAGCTGTAGGGAAAA
CATAAATGACTGCATTATTGATTTATTGAAAGGCAACCTGTATAATGATAATTTGTCTGATATTAATTTTGC
TCCTCCTAAACCTGAAGAATGTCGAAGTAAGATAACTTTGAAAGGTCCTCGTTCACCTTTGGAGATGCATCT
GTATTCTCTTCCCAAAAAGTGCCAAGGAAAAGAAACAATCATTGAATGGAATTCCGTGAATTCCGTTCTCTT
GGATACTTATCCGTTGGATCCACATAGCAGGCTTGTTGTCGCAGCTTCAGTGACACAGAATGCTGGCTCGA
ATCGGTTAACCTTAAGGAACACAACAATCACGCCTAACATCCATGGTTTAAGCTCTTTGATATGTCTCATTT
TTGCTCCGAGGGTAGAACTCAGAGCAGATAATGAAAGGCGGCAGCTAACGGGAGCCCTCTGTGGGCTAGG
GTTCAATCCAGAAACTGGAGTATCTTTTTATCCTGAAAATGATTTCGAATTTATGTTTGATACAAAACTGAC
ACTCCTGGATTTGGAAATGGTAAATAAGTTGAGGTTTTGGATGGACTATATCATGGGGGGTGGAGAAGGAC
CACACAGCGAGCTGTCGAGGCCTGGCATTATAAAGTCCCAAGAGAGGATAAGGACTTACATCATAGATTTG
GTGTTCAAGAAACGCCCTTGCATCACACCCCAGCCAGTCTGCAACCCTCACGAGTGGGACCAGCTGGAGCC
AGACGAGCTGCTGGACCCCCAGGGCAGTGACACCAGCCTCTACCCCCTCATCTGGGGTATCTCACTCGTCG
GGGAAGGAGCTAGGGATAATGCCGTTCTGTCCAGGCTGGAGACACTTAATATGATTGCTCAAGGGAAAGA
GTCTTTCAAGACGGCTGCTCAGTGTGAGCTATGCCAAGTGTACAACGAAACATTGCAGGAGCTGAGACTGC
ACCTCACTACGACGCTGCACACGATGAAGTTGGCCGATTTTAAGGAGATGATGAGAGCCGAAAAAAGGAA
ATGACGACGCAAAGGAAAGCCCTCTCGATAAGTCATCTTACCAAGTCGCCTTTCAAAGTTTAATAGGATTT
ATTCCTTTCTTAAATTTACTTTACTGATTTTTATTTTTTGTTGAAAAATTTTGACATTTTATGGTAACCTATAT
TTTTTACAAGGGTTTCATTAGAGTGTTTTATTTCTACTTTTTAAAGGAAGTATTTTGGTTGTTACCATTATTTT
CATTTTATATGTATAGTTTATTGTAGATCTTTTTAATTATTATTTTAAAAAAATCATATCTACCTCTGATTAC
AGTG

Protein

RF: +3

ORF: 84 -> 3788

Length: 1234 aa

>lcl|ORF5_TRINITY_DN8326_c0_g1_i2:83:3787 unnamed protein product

MYCTTGVLLQQLVKKQSLSDYTHIIVDEVHERDNETDFLLIILKKLLRSAQCRTKVILMSATMNVNQFSDYFSRT
VNGLPIEPPIVELAHIAKFPVQYYYLDSLANRLPGIKVPDIDINTPSISTYTYDLARALIEVFRVIDEHEHHDTNFIG
SVLIFLPGIAEIETMYSRLNNYKGSAEKWWLCPLHSSVTYDEQMKAFQPAPKGHRKIILATNIAESSITVPDIKYVI
DFCLAKQQVLEPETSYSCLQLTWVSKSQGTQRAGRVGRVMPGRVYRLIPKDYYMELPEDITPEIMRCPLDQLVL
KAKQLRMGNPATLLGLAIDPPDLSNIHKTILHLKEAGALLLTSNGEYKDNDGDLTFIGQIMASLPLDIHLSKLIILG
HMFSCLSDAIVMACAMSVKSIFSTPFRQQLEAYNSKLTWADSSCSDPIAYLHAYSLWKFKSKMGYFKRSGGESE
LDWCRKYFIQGNLIREVSRLENEIIQRLRGLGVEEMKGETSVSWTEAEKPIILKLIIAGAFYPNYFIPFGSDEKDSV
KILGGRDPHSTVYLTGLPNNQPGPLYTHSIRSHFQHCGSNIEVSFDGSSKIYLTFGSSVSANREPEKTPLMPGKTS

MAVYRSIKLRQLQVPITVPVLLPHEARKRAQEVFGDRLTPSLFNVNKRKAVTPTKKTYMPSLATSILPVVITNVE
SPSKFWVNINEPVNTNRLFWIQSSLNKISEPLPPYTGPYDDGTPCIAPYRDKKGPPEYYRARIISSIEIDQRGTVVR
VQVYFVDYGNQESVYTKDLRTYPESLNNVKEEPDLALEASLAEIGPCFAKNPKGGWAPEHCREFYKFLKDNTG
VAKIFSVVNNVMAVTLFSSELMKRKTEDIPFEMSFNYYLIKNGFAEPVDEPYLSRENHMLRESSSCRENINDCIID
LLKGNLYNDNLSDINFAPPKPEECRSKITLKGPRSPLEMHLYSLPKKCQGKETIIEWNSVNSVLLDTYPLDPHSRL
VVAASVTQNAGSNRLTLRNTTITPNIHGLSSLICLIFAPRVELRADNERRQLTGALCGLGFNPETGVSFYPENDFE
FMFDTKLTLLDLEMVNKLRFWMDYIMGGGEGPHSELSRPGIIKSQERIRTYIIDLVFKKRPCITPQPVCNPHEWD
QLEPDELLDPQGSDTSLYPLIWGISLVGEGARDNAVLSRLETLNMIAQGKESFKTAAQCELCQVYNETLQELRLH
LTTTLHTMKLADFKEMMRAEKRK

Conserved Domains

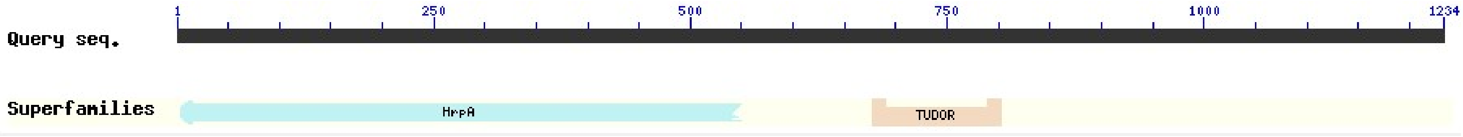

BLASTp

XP_014286769.1 probable ATP-dependent RNA helicase spindle-E [*Halyomorpha halys*]

Score:2285 bits

E-value: 0.0

Query 211 MYCTTGVLLQQLVKKQSLSDYTHIIVDEVHERDNETDFLLIILKKLLRSAQCRTKVILMS 270

MYCTTGVLLQQLVKKQSLSDYTHIIVDEVHERDNETDFLLIILKKLLRSAQCRTKVILMS

Sbjct 1 MYCTTGVLLQQLVKKQSLSDYTHIIVDEVHERDNETDFLLIILKKLLRSAQCRTKVILMS 60

Query 271 ATMNVTQFSDYFSRTVNGVSIEPPVVELAHISKFPVQYYYLDSLTNRLPGIKIPEINLNN 330

ATMNV QFSDYFSRTVNG+ IEPP+VELAHI+KFPVQYYYLDSL NRLPGIK+P+I++N

Sbjct 61 ATMNVNQFSDYFSRTVNGLPIEPPIVELAHIAKFPVQYYYLDSLANRLPGIKVPDIDINT 120

Query 331 PSISTYSYDLARALIEVFRVIDEHEHHDNNFIGSVLVFLPGIAEIETMYARLQSYAGSAE 390

PSISTY+YDLARALIEVFRVIDEHEHHD NFIGSVL+FLPGIAEIETMY+RL +Y GSAE

Sbjct 121 PSISTYTYDLARALIEVFRVIDEHEHHDTNFIGSVLIFLPGIAEIETMYSRLNNYKGSAE 180

Query 391 MWWLCPLHSSITYDEQMKAFQPAPRGHRKVILATNIAESSITVPDIKYVIDFCLAKQQVL 450

WWLCPLHSS+TYDEQMKAFQPAP+GHRK+ILATNIAESSITVPDIKYVIDFCLAKQQVL

Sbjct 181 KWWLCPLHSSVTYDEQMKAFQPAPKGHRKIILATNIAESSITVPDIKYVIDFCLAKQQVL 240

Query 451 EPETGYSCLQLTWITKSQGTQRGGRVGRVMPGRVYRLIPKDYYMELSEDITPEIMRCPLD 510

EPET YSCLQLTW++KSQGTQR GRVGRVMPGRVYRLIPKDYYMEL EDITPEIMRCPLD

Sbjct 241 EPETSYSCLQLTWVSKSQGTQRAGRVGRVMPGRVYRLIPKDYYMELPEDITPEIMRCPLD 300

Query 511 QLVLKAKQLRMGNPATLLGLAIDPPDLSNIHKTILHLKEAGALLMTANGQYKDNDGDLTF 570

QLVLKAKQLRMGNPATLLGLAIDPPDLSNIHKTILHLKEAGALL+T+NG+YKDNDGDLTF

Sbjct 301 QLVLKAKQLRMGNPATLLGLAIDPPDLSNIHKTILHLKEAGALLLTSNGEYKDNDGDLTF 360

Query 571 IGQIMASLPLDIHLSKLIILGHMFSCLSDAIIMASAMSVKSIFSTPFRQQLDAYNSKLTW 630

IGQIMASLPLDIHLSKLIILGHMFSCLSDAI+MA AMSVKSIFSTPFRQQL+AYNSKLTW

Sbjct 361 IGQIMASLPLDIHLSKLIILGHMFSCLSDAIVMACAMSVKSIFSTPFRQQLEAYNSKLTW 420

Query 631 ADSSCSDPIAYLHAYSLWKFKSKMGFFKRSGGESEIDWCRKVFIQGNLIREASRLENEIV 690

ADSSCSDPIAYLHAYSLWKFKSKMG+FKRSGGESE+DWCRK FIQGNLIRE SRLENEI+

Sbjct 421 ADSSCSDPIAYLHAYSLWKFKSKMGYFKRSGGESELDWCRKYFIQGNLIREVSRLENEII 480

Query 691 QRLRGLGVEEMKGETSVSWTAAEKPVILKLIIAGAFYPNYFIPFGSDEKDAVKLLGGRDP 750

QRLRGLGVEEMKGETSVSWT AEKP+ILKLIIAGAFYPNYFIPFGSDEKD+VK+LGGRDP

Sbjct 481 QRLRGLGVEEMKGETSVSWTEAEKPIILKLIIAGAFYPNYFIPFGSDEKDSVKILGGRDP 540

Query 751 LSTVYLTGLPNNQPGPLYTHAIRNHFLHCGSNIEVSFDGSSKIYLSFGSSVPTNREPEKA 810

STVYLTGLPNNQPGPLYTH+IR+HF HCGSNIEVSFDGSSKIYL+FGSSV NREPEK

Sbjct 541 HSTVYLTGLPNNQPGPLYTHSIRSHFQHCGSNIEVSFDGSSKIYLTFGSSVSANREPEKT 600

Query 811 DLMPGKISMAVYRSIKLRQLQVPITVPVLLPHEARKRAQEVFGDRLTPSLFNVNKRKAVM 870

LMPGK SMAVYRSIKLRQLQVPITVPVLLPHEARKRAQEVFGDRLTPSLFNVNKRKAV

Sbjct 601 PLMPGKTSMAVYRSIKLRQLQVPITVPVLLPHEARKRAQEVFGDRLTPSLFNVNKRKAVT 660

Query 871 PIKKTNMPSLGTSILPIVFTHVESPSKFWVNVNEPVNSNRIYWIHSSLNRISEPLPLYTG 930

P KKT MPSL TSILP+V T+VESPSKFWVN+NEPVN+NR++WI SSLN+ISEPLP YTG

Sbjct 661 PTKKTYMPSLATSILPVVITNVESPSKFWVNINEPVNTNRLFWIQSSLNKISEPLPPYTG 720

Query 931 PYDDGSPCIAPFNDKNGPSEYYRARIISSIDVDQSGAVTRVQVIFIDYGNQESICTKELR 990
PYDDG+PCIAP+ DK GP EYYRARIISSI++DQ G V RVQV F+DYGNQES+ TK+LR
Sbjct 721 PYDDGTPCIAPYRDKKGPPEYYRARIISSIEIDQRGTVVRVQVYFVDYGNQESVYTKDLR 780

Query 991 DYPKSLTNVKEEPDLALEASLAEVGPSFTKNPRGGWSPESCKEFSRFFKNNTGIAKIFSV 1050
YP+SL NVKEEPDLALEASLAE+GP F KNP+GGW+PE C+EF +F K+NTG+AKIFSV
Sbjct 781 TYPESLNNVKEEPDLALEASLAEIGPCFAKNPKGGWAPEHCREFYKFLKDNTGVAKIFSV 840

Query 1051 VNNVMAVTLFSSDLLSKKSEAHIPFEMSFNYYLIKNGFAEPIDEPYLSRENHMLREASER 1110
VNNVMAVTLFSS+L+ +K+E IPFEMSFNYYLIKNGFAEP+DEPYLSRENHMLRE+S
Sbjct 841 VNNVMAVTLFSSELMKRKTE-DIPFEMSFNYYLIKNGFAEPVDEPYLSRENHMLRESSSC 899

Query 1111 SSSTNHC-LEVLNADLYNDNLSDINFEPPRPEECRSKVTLKGPRSPLEMNLYSLPKKCQG 1169
+ N C +++L +LYNDNLSDINF PP+PEECRSK+TLKGPRSPLEM+LYSLPKKCQG
Sbjct 900 RENINDCIIDLLKGNLYNDNLSDINFAPPKPEECRSKITLKGPRSPLEMHLYSLPKKCQG 959

Query 1170 KETIIEWNSVNSVLLDTYPMDPHSRLVIAASVTQNAGSNRLTLRNTTIMPNIHGLSSLVC 1229
KETIIEWNSVNSVLLDTYP+DPHSRLV+AASVTQNAGSNRLTLRNTTI PNIHGLSSL+C
Sbjct 960 KETIIEWNSVNSVLLDTYPLDPHSRLVVAASVTQNAGSNRLTLRNTTITPNIHGLSSLIC 1019

Query 1230 LIFAPRVELRADSERRQLTGALCGLGFEPETGAAFYPENDLEVLFDTKITLMDLEVVNKL 1289
LIFAPRVELRAD+ERRQLTGALCGLGF PETG +FYPEND E +FDTK+TL+DLE+VNKL
Sbjct 1020 LIFAPRVELRADNERRQLTGALCGLGFNPETGVSFYPENDFEFMFDTKLTLLDLEMVNKL 1079

Query 1290 RFWMDYIMGGGEGPHSELSRPGIIKSQERIKSYITDLLFKKRPCVTPQPVSYPHEWDQLE 1349
RFWMDYIMGGGEGPHSELSRPGIIKSQERI++YI DL+FKKRPC+TPQPV PHEWDQLE
Sbjct 1080 RFWMDYIMGGGEGPHSELSRPGIIKSQERIRTYIIDLVFKKRPCITPQPVCNPHEWDQLE 1139

Query 1350 PDELLDPQGCDTSLYPLIWGISLVGEGARDNAILSRLETLNMIAEGKEIFKTAVQCELCQ 1409
PDELLDPQG DTSLYPLIWGISLVGEGARDNA+LSRLETLNMIA+GKE FKTA QCELCQ
Sbjct 1140 PDELLDPQGSDTSLYPLIWGISLVGEGARDNAVLSRLETLNMIAQGKESFKTAAQCELCQ 1199

Query 1410 VYNETLQELRLHLTTTLHTIKLADFKESMKAERRK 1444

VYNETLQELRLHLTTTLHT+KLADFKE M+AE+RK

Sbjct 1200 VYNETLQELRLHLTTTLHTMKLADFKEMMRAEKRK 1234

Maelstrom

>TRINITY_DN4191_c0_g1_i1 len=1979 path=[0:0-940 1:941-1092 2:1093-1978]

TGTATTGATTTTATCTCTTGTCCTTAATTTTCTGAAATTCACACAGTTGGCAGTAATGATAATCCTCTTATAG
AAGTCCTGTTTTGGCACGAAACCTTTCTCTTATCTATTCTCTTGGTTGAAACCTCTTAGCTTGACAGACGTTT
GTGGCCGCTTTCTTTAAATTTGTTGATTCTTTCATTCCAGATAGCTGACAATGCCACAAAAGAAGAAACAGA
AAGCTGGGAAAAATGGGTTTTATTTTTTTATGTTAGAGGTTCAAAAGCAAGAAGCTCAAAAGGGAAATCGA
CATACTTTACCTGAAATTTCTGAAATTGCAAATCCCTTGTGGTCGGCCATGACACCAGAAGAGCGTAAGCC
ATATAATGACAAGGCATTAATGAATGTTTCCAAAAATAACGAAGATAACTCTAAAAAATACACATCTCTTG
GTATCAGTTATGCTGAGCTAGATGCTGAACAGAGAGAGTTAGATGAAGCTCGTAACCTTATGATAGCGACT
ATTAAAAATACAGTAGCTAACCTTGACATCAAAACTTCTTTGAAGACTCATAGATTCTTTGTGTGTCATGTC
AGCTATTTCTACAAGTCTGACCATCACTTTTATTGTCCAGCTGAAGTTGCTCTTGCAGCATTTTCTTTGGAAT
TTGGAGTTCTAGGCACCATTCATTTCTTCGTTGACCCTGGAAAGATTCCTCTTGGTTTTAAATACGAAGCTG
CTGAATGGTCAGCTAGAACTCATGGAATTCCAATAAACGATCCGAGTTGGAATGAAGGGATCAAAGATCCT
CTAGAAATGTTTAGAAAAGTTAAGGATTTCATAAAGAAATTTGCTGGCCCTTCTGAAGTACCTCCAATCTAC
ACATTGTTTGATACTTTAAATGCTAACAGTCGTGTTTCTGTTGTGAAATCTGCTTTGAATATGATGTGTGATG
CTGCTAATGAAGACTCCTCTCTATTCCGTGTTTATTGTTTATCACACTTATTCTTTGAAATTAGAAATAAATG
TTCTGCAGAAATACCAAGTGTAGCCATTATGAAAAGTGAATTAGATAAAGATGTTTTTTCATATGCGAGGG
ACCTAGGTTGTTATTATCACGAAGAGAAAGATCTTTCTGTGTATTGTTCACTCTCTATTGTGACAAGGTGGG
TATTCACCATTTGTGATCATTGTTGCAAGCATCTTGGTATAAAGCTTATTCTTGGTAGGCACGTGCCTAAAG
ATACAGACCTTTCAGAAAGTGCTCATTTCATAGAAGAAAAATACTCTTCAAATAAACTTGATAATTGTGGTT
CTTTTAGTAAACCTTTGACGATCATTGACCATGGGCGTTTAAAAGAACAAAGAGCAGAACAAAAACGAGCC
CAGGATATGCACTTGCGTGAAAGTGAAAAAATTAGGCTTCCTAAATCCAGTTATAGCAACAAAATTGGTGC
ACGATTCAGTCAAGACCTGGGTGCTAGTTGTTCCCAAAGATCTTTGGAAACCAAACAGGAATCCAATGAAA
ATGAATGGTGCATGGTAAAAGGAAAAGGATTTGGACGGGGTCGTGGTTTTATTACAGAAGAGCATTCACCT
GAAGAGCCTATCTGGCCAGTTTCTGGAAGGGGAAGAGCATTTCAACAATGAATTTAAGAAAATAATTTCAT
GTTGACAAATAAAATTTTAATATTTTTTATGAAAGTTTTTTATACTGTTTGATAAGTTAAATAAAGATTAGA
TAAAATATTTCCAAAAAAATGTTTTCACCAAGTTGTAATCATATTTTGTTATCAATGTGTATTATTTATTGAC
GTTCTGAACAAGGTTTAAAGATCTCCCTTTTTTCTCCACAGATTTTATACAACTAAGTTATTTTGGTAAATTT
CACTGGTTATATATAAGTTTACATTGTATAATTAAATTGACAACTTGTTTTGTTTTATGATTAATCAGTCATC
ACTTCAATTTGTTATTTTAATAAATTATCTATTACAAG

Protein

<u>RF:</u> +2

<u>ORF:</u> 197 -> 1630

<u>Length</u>: 477 aa

>lcl|ORF1_TRINITY_DN4191_c0_g1_i1:196:1629 unnamed protein product

MPQKKKQKAGKNGFYFFMLEVQKQEAQKGNRHTLPEISEIANPLWSAMTPEERKPYNDKALMNVSKNNEDNS
KKYTSLGISYAELDAEQRELDEARNLMIATIKNTVANLDIKTSLKTHRFFVCHVSYFYKSDHHFYCPAEVALAAF
SLEFGVLGTIHFFVDPGKIPLGFKYEAAEWSARTHGIPINDPSWNEGIKDPLEMFRKVKDFIKKFAGPSEVPPIYTL
FDTLNANSRVSVVKSALNMMCDAANEDSSLFRVYCLSHLFFEIRNKCSAEIPSVAIMKSELDKDVFSYARDLGC
YYHEEKDLSVYCSLSIVTRWVFTICDHCCKHLGIKLILGRHVPKDTDLSESAHFIEEKYSSNKLDNCGSFSKPLTII
DHGRLKEQRAEQKRAQDMHLRESEKIRLPKSSYSNKIGARFSQDLGASCSQRSLETKQESNENEWCMVKGKGF
GRGRGFITEEHSPEEPIWPVSGRGRAFQQ

Conserved Domains

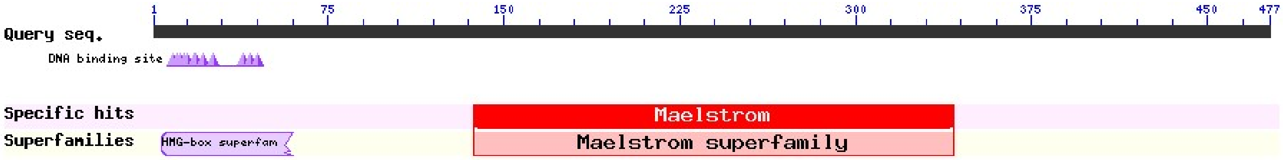

BLASTp

XP_014290039.1 protein maelstrom homolog isoform X1 [*Halyomorpha halys*]

Score: 449 bits

E-value: 0.0

Query 18 MTPEERKPYNDKALTSVSKDHEDNTKKFTSLGISYAELDAEQRELAEAHNLMLATIKNTV 77

MTPEERKPYNDKAL +VSK++EDN+KK+TSLGISYAELDAEQREL EA NLM+ATIKNTV

Sbjct 48 MTPEERKPYNDKALMNVSKNNEDNSKKYTSLGISYAELDAEQRELDEARNLMIATIKNTV 107

Query 78 ANLDINSSLKSHKFFVCYVNYFYKNDHHIYYPAEVALAAFSLESGVLGTIHFFVDPGKIP 137

ANLDI +SLK+H+FFVC+V+YFYK+DHH Y PAEVALAAFSLE GVLGTIHFFVDPGKIP

Sbjct 108 ANLDIKTSLKTHRFFVCHVSYFYKSDHHFYCPAEVALAAFSLEFGVLGTIHFFVDPGKIP 167

Query 138 LGYKFEAADWSTRTHGIPVNDPSWNEGIKDHFEMFKKVKDFLKSFSDSNEVPPIYTMFDS 197

LG+K+EAA+WS RTHGIP+NDPSWNEGIKD EMF+KVKDF+K F+ +EVPPIYT+FD+

Sbjct 168 LGFKYEAAEWSARTHGIPINDPSWNEGIKDPLEMFRKVKDFIKKFAGPSEVPPIYTLFDT 227

Query 198 LNANSRLSIVKSALNLMCDAANEDPDLFRVYCLPHLFFEIRNKCSKEIPSVAIMKSELDK 257

LNANSR+S+VKSALN+MCDAANED LFRVYCL HLFFEIRNKCS EIPSVAIMKSELDK

Sbjct 228 LNANSRVSVVKSALNMMCDAANEDSSLFRVYCLSHLFFEIRNKCSAEIPSVAIMKSELDK 287

Query 258 DVFSYARDLGCYYHEEKDLSMYCSLSVVTRWVFTICDHCCKHLGIKLILGRHVPKDTDLS 317

DVFSYARDLGCYYHEEKDLS+YCSLS+VTRWVFTICDHCCKHLGIKLILGRHVPKDTDLS

Sbjct 288 DVFSYARDLGCYYHEEKDLSVYCSLSIVTRWVFTICDHCCKHLGIKLILGRHVPKDTDLS 347

Query 318 ESTRFMDDKYSPNKLDHCGSHSKPLTIIDHGRLKEQRAEQKRAQDRHLRESEKIRLPKSS 377

ES F+++KYS NKLD+CGS SKPLTIIDHGRLKEQRAEQKRAQD HLRESEKIRLPKSS

Sbjct 348 ESAHFIEEKYSSNKLDNCGSFSKPLTIIDHGRLKEQRAEQKRAQDMHLRESEKIRLPKSS 407

Query 378 YSNKIGARFSNDLGASSSRSSVEKKEESSENEWRTVRGKGFGRGRGFLVQEPSAKAPVSA 437

YSNKIGARFS DLGAS S+ S+E K+ES+ENEW V+GKGFGRGRGF+ +E S + P+

Sbjct 408 YSNKIGARFSQDLGASCSQRSLETKQESNENEWCMVKGKGFGRGRGFITEEHSPEEPI-- 465

Query 438 WPVTGRGRAF 447

WPV+GRGRAF

Sbjct 466 WPVSGRGRAF 47529

HEN1

>TRINITY_DN3220_c0_g1_i1 len=3255 path=[0:0-3254]

ATAACCGGTAAAGCAGTGAATAGCCGAAGTCTTTTTGTTTGTTTACAAACATATTTATCTAAGTTTTATGGC
TTAAAGATTAATAGTTCGCTATAATGCTTTGAGATGCACAACATAACCGCCTTTTAATTGTTACTGCTTCCT
ATAATGATTTTTTCCTTTTTATTTTTCATTAATTACCTTTATAAAGTTATCCAAACCAGGATACATAAAAGTA
CCCAATCCAATAATGATTTATGTGAACCTGTTGCAATTCAAATACTGAAGAGTATAGAATTTGATGGGATTA
CTCTTGAAGATGATGATGTGGGTGTTAGATTTATGCCTCCAGCTTACAATCAAAGATATCTGGCTGTATGCA
ACGTTTTGGTTCAGTTACAATCAGTATATGACATTAAAAAGGTTGTCGACTTAGGCTGCTCAGAAATAGGAT
TCTTCTCACATATTAAAAGAATTGAAACTATAGAGGAATACATAGCTGTGGATATTGATCTATTTACACTTG
AAAGAAGTTCGTGTAGAGTTACGCCACTCCACGCAGATTACCTATCAAGAAGATGTACTCCTCTGAAAGTG
AGTGTTTTACAAGGGTGTGCTACAGAGCCTGATAATAGATTGAAAGATACGAATGCCATTGTAGCTATTGA
ACTTATTGAACACTTGTATCCTTGGGACTTGGAGATTCTGCCTTATAATATCTTCAGTATTATTCAACCAGAT
GTTGCAATATTTACTACACCTAATGCAGACTTCAATGTGCTATTCAAAAGTCTGCATCAAAATACATTTAGG
CATGATGATCATAAGTTTGAATGGACTCGGGAGCAGTTTGAAGATTGGGGTCACAATCTAACTTTGAGGTA
TCCTGGTTATGAAGTTTGGTTTAATGGAATCGCTAAGGGACCAGCTGGAACTGAAGAATTAGGCTGTTGTTC
TCAAATAGCTGTTTTTGTAAGAATAGAAGCAAAAACTGAAAGCAATGAATTCTATGATTGTTATCATTCTGT
TCACGAAGTAGATTATCCTTATCAAACGAAAGAAGAAAAGTATAACACCATTTTGCAAGCTGATGTTCAAC
AATTTATTGACCGTTTTTCTCAAGATGAAAAGTACCTAGTCGGAGACGAAATTGAGATACCTCTAGTTGACA
TTATTAATTACATTAGTGTTCAATGCTCAGAAGAAAGGTTGAAGAATTATCTGGAAGAATCTGAAAAAGAA
GTTGAACTTAGGGATAACCAACTCAAGTTAGTTCTGCCTTATTCTTCAGATAATGATGATGAGGCTCTTTCT
TCATATGATGATATCATTGAAGAAATTTATGATGGTGAAGAAAGCTGGATCATTGACAACAATGAGTTTTTT
AATGAAGAGCACTGGAGCAATGTTTTAGAAGATGTGAATGAAATGCAACCTGAGCCAGATTCCAATTTTGA
TCAAACCGATGATGTTGTTAGGAATTTGGCAGACCAGTTAGAATGTTTAATCAGAAGTACTAGAGGAGAAG

CGCAAAATGAAAATGAACAGAATAACTCTGGTACGGATGTACCCTACGAGCCAAAAGACACGAGTGTCAA
TGATTTGGTATTGCCAGATGAAGAGGATCTTAGGTCTGATTCTGTAACTCCAGTTGGAGAAAATCAGGTTCA
GTATCTCAATGTCGATAGTATTCTTCAACAATCAGGTTCAACTGGATCTGGTAATACATCTAAATCAAGTGA
TACTCTTGTGGACAACTCTTTATCACCAACGAATACAATAAAACACTGTATGAATAACGAATTCAGTGAAA
ATTTTGCTCTCATTTCACCTTTAAAAGATTCTTGTTCAGATACAGAACAATATCCAGGTCCATCTTCTTCTAA
TGAGGTTGTCCAAATTCCAGAAAACAAACCTCAGAATAATCATTCAAATGAGGTTGTTGAAATTCTAGAAA
ACAAACCTCTGAATAATCATTCAAATGATGTTGTCGAAATTCCAGAAAACAAGCCTTATCAGAATAATCAA
CAATCCTCATTTGATGTTAAGCCAAGTAAAGAGCAAAAAGAAATCAATGAAAATATAGACATTCCATCATC
TAGTTCAGGGTCCCTTGCCAGAGAGCCTGGTTATGTTGCAATAGAAGAAAGTAGTAATGTGAAAGAAGTTC
AGTCGGACCAGTCTTCATCTCTTGTAACTAGGAGTCAATCTTTAGAAAATGTAAATATGTACAAATGTAAA
GACAAACGTTTTAGGTATAGTTTAAATATAGATAATAAAATACTCGACTGTGATACTTCAGTTATAGATATG
GATCTAGATACGTGTAAGGGGTCAAGCCTTCCTAGTCTTACAGAAACTTATGGTAGTCACAGTAAGGCGGT
AGATAGTGGCTATCCTAATTCGACGCAAGATATGGAACTCGATTTGACTCCTGAACAGGTAGACGAAATTA
TCACTGAAACAGAATCTAGTTTAGAAGATGAAGAGTCTTCTGCTGACGAAAGGATCGAGCCTGTTCTTGGC
AACCCTCCTATTGTGTTTGCCGACAATGTAGAAAATGGGGACGTAGCCAACAATAATAGGGATGGCGAAG
GCAACAATATGGAAGGAGGTGAAGTACTACCAGAGGAGTTGGTTTTCGAGGATTTAGTGATAGGCGGTGA
AGAAGTGGAAGCTGCGGGAGAGCACGAATCAGACGACGATGGACTTTTCCTCTCGGCTCTAGATGTATCGA
ATGATACCATTGTGCCTGAAGAAATGTCATCGAATGAAGACACTATTTTCCTCTTCGACTTGCCATCTCCGA
CTTCATTTGGTAGCCTTCACGTGGAAGAATCTATTAGTGTCCAGGATGAGAACCAATTCCCCTCCTGGCTGA
TGAACATGGACGTGTGGGATGATGACAACGGGAGTGGTGATGCAGGCCCTGATAGAGGTGAAGACAGTAG
TGACCTTCTTGTAGACCGCGAAGGACAAGTTGACTTTTACCCAGGGGACGCCAGGGCTGCGGGGGAGCCGA
CTCTTCCTCATGACTGAGCCACTCATTATGTTTTATTATTCAACATATCATTTGTAAAAAAATGTATTATAAG
TATATTATAGTTAAGCAATGCTTTCATTATGTGAAGTGCTTAATTGGAAGTTTGAATCTCCCATGTGCCAAA
GCTGGATGATTGGAAATAATGTAATTAAGACTGATGTTACCATATTAGTCATCTAAAATTTTTATTTTTGTT
ATTACTAAAAATAAATTTTTTACAAAATCAAAAAAAA

Protein

<u>RF:</u> +1

<u>ORF:</u> 148 -> 3018

<u>Length</u>: 956 aa

>lcl|ORF1_TRINITY_DN3220_c0_g1_i1:147:3017 unnamed protein product

MIFSFLFFINYLYKVIQTRIHKSTQSNNDLCEPVAIQILKSIEFDGITLEDDDVGVRFMPPAYNQRYLAVCNVLVQL
QSVYDIKKVVDLGCSEIGFFSHIKRIETIEEYIAVDIDLFTLERSSCRVTPLHADYLSRRCTPLKVSVLQGCATEPD
NRLKDTNAIVAIELIEHLYPWDLEILPYNIFSIIQPDVAIFTTPNADFNVLFKSLHQNTFRHDDHKFEWTREQFEDW
GHNLTLRYPGYEVWFNGIAKGPAGTEELGCCSQIAVFVRIEAKTESNEFYDCYHSVHEVDYPYQTKEEKYNTIL
QADVQQFIDRFSQDEKYLVGDEIEIPLVDIINYISVQCSEERLKNYLEESEKEVELRDNQLKLVLPYSSDNDDEAL
SSYDDIIEEIYDGEESWIIDNNEFFNEEHWSNVLEDVNEMQPEPDSNFDQTDDVVRNLADQLECLIRSTRGEAQN
ENEQNNSGTDVPYEPKDTSVNDLVLPDEEDLRSDSVTPVGENQVQYLNVDSILQQSGSTGSGNTSKSSDTLVDN
SLSPTNTIKHCMNNEFSENFALISPLKDSCSDTEQYPGPSSSNEVVQIPENKPQNNHSNEVVEILENKPLNNHSND
VVEIPENKPYQNNQQSSFDVKPSKEQKEINENIDIPSSSSGSLAREPGYVAIEESSNVKEVQSDQSSSLVTRSQSLE
NVNMYKCKDKRFRYSLNIDNKILDCDTSVIDMDLDTCKGSSLPSLTETYGSHSKAVDSGYPNSTQDMELDLTPE
QVDEIITETESSLEDEESSADERIEPVLGNPPIVFADNVENGDVANNNRDGEGNNMEGGEVLPEELVFEDLVIGGE
EVEAAGEHESDDDGLFLSALDVSNDTIVPEEMSSNEDTIFLFDLPSPTSFGSLHVEESISVQDENQFPSWLMNMD
VWDDDNGSGDAGPDRGEDSSDLLVDREGQVDFYPGDARAAGEPTLPHD

Conserved Domains

No putative conserved domains have been detected

BLASTp

XP_014284423.1 uncharacterized protein LOC106685926 [*Halyomorpha halys*]

Score:1264 bits

E-value: 0.0

```
Query  1    MIVPFLFLINYLLKVIQSRIRKSTTTLQNNVRKPDAIQLLNSLEFEGTAI-NDAVGIRFL  59
            MI  FLF INYL KVIQ+RI KST +  N++ +P AIQ+L S+EF+G  + +D VG+RF+
Sbjct  1    MIFSFLFFINYLYKVIQTRIHKSTQS-NNDLCEPVAIQILKSIEFDGITLEDDDVGVRFM  59

Query  60   PPAFSQRYTAVYNILVQLQSIYDIKKIVDLGCSEISFFTHIKGLESIEEYIGVDIDEATL  119
            PPA++QRY AV N+LVQLQS+YDIKK+VDLGCSEI FF+HIK +E+IEEYI VDID  TL
Sbjct  60   PPAYNQRYLAVCNVLVQLQSVYDIKKVVDLGCSEIGFFSHIKRIETIEEYIAVDIDLFTL  119

Query  120  LRHSCRAKPLNYDYLSRRCSPLKVSIFHGSATEPDVRLKYTNAVVAIELIEHMYPPELDD  179
             R SCR  PL+ DYLSRRC+PLKVS+  G ATEPD RLK TNA+VAIELIEH+YP +L+
Sbjct  120  ERSSCRVTPLHADYLSRRCTPLKVSVLQGCATEPDNRLKDTNAIVAIELIEHLYPWDLEI  179

Query  180  LPYNIFGVIQPDVAIFTTPNADFNVLFTTLKPNTFRHYDHKFEWTRQQFEDWGHNLALRY  239
            LPYNIF +IQPDVAIFTTPNADFNVLF +L  NTFRH DHKFEWTR+QFEDWGHNL LRY
Sbjct  180  LPYNIFSIIQPDVAIFTTPNADFNVLFKSLHQNTFRHDDHKFEWTREQFEDWGHNLTLRY  239

Query  240  PNYEVWFNGIGRGPVGTEEFGCCSQIAVFVRKEVKTESSDFNVYYKTVHEVEYPFQTREE  299
            P YEVWFNGI +GP GTEE GCCSQIAVFVR E KTES++F   Y +VHEV+YP+QT+EE
Sbjct  240  PGYEVWFNGIAKGPAGTEELGCCSQIAVFVRIEAKTESNEFYDCYHSVHEVDYPYQTKEE  299

Query  300  KLNDILEYDVYQFIDHNSQNDEYLVGNKIEIPLVDILKYISIPCPEEKLKTFLEKADKHV  359
            K N IL+ DV QFID  SQ+++YLVG++IEIPLVDI+ YIS+ C EE+LK +LE++K V
Sbjct  300  KYNTILQADVQQFIDRFSQDEKYLVGDEIEIPLVDIINYISVQCSEERLKNYLEESEKEV  359
```

Query 360 ELKNNELILVLPFSSE-DNNSLSDHNEVIEEICDGEESWINANNDVLNDFVNNDETWSYV 418

EL++N+L LVLP+SS+ D+ +LS ++++IEEI DGEESWI NN+ N+ E WS V

Sbjct 360 ELRDNQLKLVLPYSSDNDDEALSSYDDIIEEIYDGEESWIIDNNEFFNE-----EHWSNV 414

Query 419 LEDVNEMKPQSKPNYDPMDDV-NDLVDELDNVLRNPENDV-TESGQNNSAMNVMCDPKQS 476

LEDVNEM+P+ N+D DDV +L D+L+ ++R+ + E+ QNNS +V +PK +

Sbjct 415 LEDVNEMQPEPDSNFDQTDDVVRNLADQLECLIRSTRGEAQNENEQNNSGTDVPYEPKDT 474

Query 477 GVNDLLLSDEEDLRADSVTPVGENQVQYLNIDSILQHSGSTGSGNTSKSSDTLVDHTLSP 536

VNDL+L DEEDLR+DSVTPVGENQVQYLN+DSILQ SGSTGSGNTSKSSDTLVD++LSP

Sbjct 475 SVNDLVLPDEEDLRSDSVTPVGENQVQYLNVDSILQQSGSTGSGNTSKSSDTLVDNSLSP 534

Query 537 MNKIKNCTNDDYSEKNAL-SPLKNSFSDKEEYPCSSTINDAVEISLNI------------ 583

N IK+C N+++SE AL SPLK+S SD E+YP S+ N+ V+I N

Sbjct 535 TNTIKHCMNNEFSENFALISPLKDSCSDTEQYPGPSSSNEVVQIPENKPQNNHSNEVVEI 594

Query 584 -----------------SNPFHENDKQFSCDVLQSESQKEINENKTKNLENSLSSAIPVG 626

N ++N++Q S DV S+ QKEINEN +S S A G

Sbjct 595 LENKPLNNHSNDVVEIPENKPYQNNQQSSFDVKPSKEQKEINENIDIPSSSSGSLAREPG 654

Query 627 ESNRVTDTVVEEVSNSKEVHYDQSTSIVTRSQSLENINLYKCKEKCFRYSLNIDNKLLDC 686

+EE SN KEV DQS+S+VTRSQSLEN+N+YKCK+K FRYSLNIDNK+LDC

Sbjct 655 Y------VAIEESSNVKEVQSDQSSSLVTRSQSLENVNMYKCKDKRFRYSLNIDNKILDC 708

Query 687 DTAVVDMDLETCKGSSIPSLTETYGSHSKAVDSGYPNSTQDMDLDLTPEQVDEIITETES 746

DT+V+DMDL+TCKGSS+PSLTETYGSHSKAVDSGYPNSTQDM+LDLTPEQVDEIITETES

Sbjct 709 DTSVIDMDLDTCKGSSLPSLTETYGSHSKAVDSGYPNSTQDMELDLTPEQVDEIITETES 768

Query 747 SLEDEDSSADERVEPVHGNPPIVFADNVENGDVANNNRDGEGNNMEGGQVLPEELIFEGL 806

SLEDE+SSADER+EPV GNPPIVFADNVENGDVANNNRDGEGNNMEGG+VLPEEL+FE L

Sbjct 769 SLEDEESSADERIEPVLGNPPIVFADNVENGDVANNNRDGEGNNMEGGEVLPEELVFEDL 828

Query 807 VIGGEEVEAADEHESDDDGLFLSALDVSNDTIVPEEMSSNEDTVYLFDLPSPSSFGSLQV 866

VIGGEEVEAA EHESDDDGLFLSALDVSNDTIVPEEMSSNEDT++LFDLPSP+SFGSL V

Sbjct 829 VIGGEEVEAAGEHESDDDGLFLSALDVSNDTIVPEEMSSNEDTIFLFDLPSPTSFGSLHV 888

Query 867 EEPTSFHNQNQFPSWLLNMEVWEDDSGSGDAGPDRDEDSSDLVVDREGQVDFYPGDARAG 926

EE S ++NQFPSWL+NM+VW+DD+GSGDAGPDR EDSSDL+VDREGQVDFYPGDARA

Sbjct 889 EESISVQDENQFPSWLMNMDVWDDDNGSGDAGPDRGEDSSDLLVDREGQVDFYPGDARAA 948

Query 927 GEPTLPDD 934

GEPTLP D

Sbjct 949 GEPTLPHD 956

PRP16, mut6 homolog

>TRINITY_DN6502_c0_g1_i1 len=3862 path=[0:0-3861]

TTTATGTTGTTTGTTTACTTGAAGCAGCTGATAAACATGTTATTGATACTTAGATAAGAAACTTGCAATTTTT
GTTCGTTTAATATCGAAATGGAAGCTGAAGATGATGAGGGTGTTTATAGATTGGAAGGAGTTGAAGAAAAA
CAAGGTGGCCTCATAATTAAAAAGAAACCAGAACCCCAAGGAAGTTTTCAATTTAAAGTTCCCCAAACTTC
CTTGCTTGGTTTAGACAGGCTTGCAGCACTACGTAGAAAAGAAAGGGAGAATGATGAAAAGAAGGAAGCT
TCAAAGAAATTAAAAGAAGAGGAAGAAAATAAAGCTAGTTTCGTAAAACCTGAAAAACATGGAGATAGGC
ATTATAGAGCTAAATATGATGAAACTCCTACTCATACTGGAGGTGTCACGAGAGAGGCCAAAGAAAGAAT
GGAAGAAAGGCGAAGGAAAGAAGAAAAGAAGAGAGGAATTCATGTTGCATCAGATGTAAAGGAACGTAA
CGATAACAATGATTCCTACAGAAAGGACTACAGGCGTGAGAAAGATAAGGAAAGAGAAAGGGACAGACA
TCGTGATAGATACAGGGCGAGAGAGAGGGAGCACGAGAGAGACAGAGAACGCAGTAGAGGAGACAAGGA
TCGCGACAGGAGAAACGATCGACGCAATGATGAAAGGAACGAGTATAGAAGTGAGCGAGGCTTCAACAG
GTCTCAAAGGTCTTGGTCAGAATCTCCAAGGTTCAAAGATGAGCCTCTGACGCCAATGTTTTCTGTTAAAGA
TACTCCATCCAGGACTGGTTGGGAAGAAGATGAAGTTTCTTCTACGCCCGCTGCAAAGTCTTCCTGGGAAC
ACCCAACACCAAAGACAGAACCGAGGGAAGATGACTGGTCCATGAGGAGACCTACTCCTGCACACAGATT
CAACAGTTGGGCGCCAGAGAGGTCACGAACTGGAGCTACACCTTTAGCTGGCAAAGAAGAAGAAGTCCCA
TGGGGCTCTGAAAAAGACCGCCTGCTCTGGGAGGAGGAGCAGAAGAGACTGGATCGAGAGTGGTACGGGC
TGGACCAGGGGATAGAGGAGGGGAGGGACCCCTTTGGCGCCGTGAGCCAGGAATACATCACTAAGAGAGA
GCGTCAGCTGGAGCGCTCCAAGAAGAAGAGGATGTCCGCGCAGCAGCGGCAGATCAACAAAGATAATGAG
CTTTGGGAAAGGAACAGGATGCTGACATCCGGGGTGGTTCAAAATCTTGACCTGGATGAAGATTTTGATGA
GGAAGCTGAAGCGAGGGTCCACCTCTTGGTCCACAACATCGTACCTCCGTTCTTGGACGGTCGTATTGTATT
TACCAAACAACCTGAACCAGTCATACCTGTCAAGGACCCGACATCAGACATGGCGTTGGTGTCGAGGAAG
GGCTCGGCGCTGGTGAGGGCCTACAGGGAACAGAAGGAGAGGAAGAGAGCCCAGAAGAAGCACTGGGAG
CTCTCGGGAACTACCATTGGTAACATCATGGGAGTGCCAAAGAAAAAAGATAAGGAGGAAGATAAAATAG

ACGGTGAAGTTGATGCCGACTATAAGACGAGTCAGAAATTCGCCGAACACATGAAAGACATGGCTCCTGC
CGCCAGCGAGTTCGCCAAGAAGAAGACCATTCAGCAGCAGAGGCAATACCTTCCGGTCTTCGCAGTCAGG
GAAGAGCTACTGAATATAATACGTGAAAACAGTGTTGTGGTTGTCGTCGGTGAAACAGGTTCTGGTAAGAC
GACCCAGCTGACCCAATACCTACACGAGGACGGCTACTCCAAGTCAGGGATGATAGGGTGCACCCAGCCC
AGGAGGGTGGCAGCCATGTCGGTTGCCAAGAGAGTGTCTGACGAGATGGCCTCTCCGTTAGGCAAGACAG
TGGGCTACGCCATTCGATTTGAAGACTGCACATCGGAGGACACCGTGATAAAATATATGACAGACGGTATC
CTGTTGAGAGAGTCACTAAGAGAGCCCGACTTGGACAACTACTCAGCCATCATTATGGACGAAGCCCACGA
AAGGTCACTTAACACAGATGTTCTCTTCGGGCTTTTGCGACAGGTGGTGGCTCGGAGGCAAGACCTGAAGC
TGATAGTCACTTCGGCCACGATGGACTCCTCAAAGTTTTCCATGTTCTTCGGACATGTTCCAGTCTACACCA
TTCCCGGAAGGACTTTCCCCGTTGAAGTACTCTTTGCCAGAAATGCAGTCGAGGACTACGTTGAAGCGTCT
GTCAAGCAAGCGTTACAGATACATCTTCAGCCAACAAAGGGAGACATTCTGATCTTCATGCCCGGTCAAGA
AGACATAGAAGTGACCTGCGAGGTGCTGGGAGAGAGGCTGGCCGAGATAGATGCAGCCCCTCCATTGTCC
ATCCTGCCTATCTACTCACAGCTGCCGTCCGACCTACAGGCAAAGATATTCCAAGCCTCACCCGATGGAGT
ACGCAAGTGTGTAGTAGCTACCAACATTGCTGAAACATCTTTAACAGTTGACGGTATTATGTACGTCGTGG
ACTCTGGTTACTGCAAGCTGAAAGTTTACAATCCAAGGATTGGTATGGACGCCTTGCAAATCTACCCCATCT
CTCAGGCCAACGCCAACCAGAGATCCGGAAGAGCTGGTAGGACAGGGCCTGGCCACGCTTATAGGCTATA
TACTGAACGCCAGTACAAGGATGAATTGTTAACTTCAACCGTGCCTGAGATTCAGAGGACTAATTTGGCAA
ACACCGTTCTGCTGCTCAAATCATTGGGTGTGCAAGATCTGTTACAGTTCCATTTTATGGACCCTCCTCCTC
AGGATAACATCCTCAATTCTCTCTATCAGTTATGGATCCTTGGTGCTCTAGACCACACAGGATCGTTGACGC
CACTTGGAAGGCAGATGGCGGAATTCCCTTTGGATCCACCGCAGTGCCAAATGTTGATAGTATCCAATCAA
ATGGGATGTACAGCCGAAATTCTAATTATAGTCTCGATGTTGTCTGTACCAACGATATTCTATCGACCTAAA
GGGAGAGAAGATGAAGCAGACAGCGTTAGAGAGAAGTTCCAAGTCCCAGAATCCGATCACTTGACCTATT
TAAACGTCTACAATCAATGGAAGCAAAACAAATACTCTTCGACTTGGTGTAACGACCACTTCATTCATGCC
AAAGCGATGAGGAAGGTGCGTGAGGTTCGCCAGCAGCTCAAAGACATCCTGGACCAACAGAAGATGGAAG
TAGTGTCCTGCGGTACTGACTGGGACATTGTCAGGAAGTGTATTTGCTCAGCCTACTTTCACCAAGGTGCTC
GCCTCAAGGGGATAGGAGAGTACGTGCACCTGAGAACAGGAATGCCCTGCCATCTTCACCCAACCTCTGCA
CTCTTTGGGATGGGTTTCACTCCCGACTATGTTGTCTACCACGAGCTGGTTATGACATCAAAGGAGTACATG
CAGTGCGTGACCGCAGTGGACGGGGTCTGGCTCGCTGAACTAGGTCCGATGTTCTTCTCCATCAAAGAGAC
CGGACGCTCGGGGTCCTCTAAGAGACGAGAGGCGATGGAGGGCATGGAGGCTGAGATGGAGGCGGCCCAG
AAGGAGATGAAGGAGAGGGAGCGGCAGGCGGAGGAGGAGCGAGGGAGGTGGTCCAAGAGGAGCAGTCA
GGTCGCAGAGCCAGGGGCCCCGGGAAGCACGCCTCTCAGGACCCCGCTCAGGCTGGGCCTTTAAGGGCTAT
CTGTACTACCTCTTAGCACAACAATCTTTGACTTCTGTCTTTGTATATACTTTTATTTAATATTAACTGTTGTA
AATAGAGTCTCTTTTAAAATAAACCCTAGTATAAGTTAAAAAAAAA

Protein

RF: +1

ORF: 91-> 3735

Length: 1214 aa

>lcl|ORF1_TRINITY_DN6502_c0_g1_i1:90:3734 unnamed protein product

MEAEDDEGVYRLEGVEEKQGGLIIKKKPEPQGSFQFKVPQTSLLGLDRLAALRRKERENDEKKEASKKLKEEEE
NKASFVKPEKHGDRHYRAKYDETPTHTGGVTREAKERMEERRRKEEKKRGIHVASDVKERNDNNDSYRKDYR
REKDKERERDRHRDRYRAREREHERDRERSRGDKDRDRRNDRRNDERNEYRSERGFNRSQRSWSESPRFKDEP
LTPMFSVKDTPSRTGWEEDEVSSTPAAKSSWEHPTPKTEPREDDWSMRRPTPAHRFNSWAPERSRTGATPLAGK
EEEVPWGSEKDRLLWEEEQKRLDREWYGLDQGIEEGRDPFGAVSQEYITKRERQLERSKKKRMSAQQRQINKD
NELWERNRMLTSGVVQNLDLDEDFDEEAEARVHLLVHNIVPPFLDGRIVFTKQPEPVIPVKDPTSDMALVSRKG
SALVRAYREQKERKRAQKKHWELSGTTIGNIMGVPKKKDKEEDKIDGEVDADYKTSQKFAEHMKDMAPAASE
FAKKKTIQQQRQYLPVFAVREELLNIIRENSVVVVVGETGSGKTTQLTQYLHEDGYSKSGMIGCTQPRRVAAMS
VAKRVSDEMASPLGKTVGYAIRFEDCTSEDTVIKYMTDGILLRESLREPDLDNYSAIIMDEAHERSLNTDVLFGL
LRQVVARRQDLKLIVTSATMDSSKFSMFFGHVPVYTIPGRTFPVEVLFARNAVEDYVEASVKQALQIHLQPTKG

DILIFMPGQEDIEVTCEVLGERLAEIDAAPPLSILPIYSQLPSDLQAKIFQASPDGVRKCVVATNIAETSLTVDGIMY
VVDSGYCKLKVYNPRIGMDALQIYPISQANANQRSGRAGRTGPGHAYRLYTERQYKDELLTSTVPEIQRTNLAN
TVLLLKSLGVQDLLQFHFMDPPPQDNILNSLYQLWILGALDHTGSLTPLGRQMAEFPLDPPQCQMLIVSNQMGC
TAEILIIVSMLSVPTIFYRPKGREDEADSVREKFQVPESDHLTYLNVYNQWKQNKYSSTWCNDHFIHAKAMRKV
REVRQQLKDILDQQKMEVVSCGTDWDIVRKCICSAYFHQGARLKGIGEYVHLRTGMPCHLHPTSALFGMGFTP
DYVVYHELVMTSKEYMQCVTAVDGVWLAELGPMFFSIKETGRSGSSKRREAMEGMEAEMEAAQKEMKERER
QAEEERGRWSKRSSQVAEPGAPGSTPLRTPLRLGL

Conserved Domains

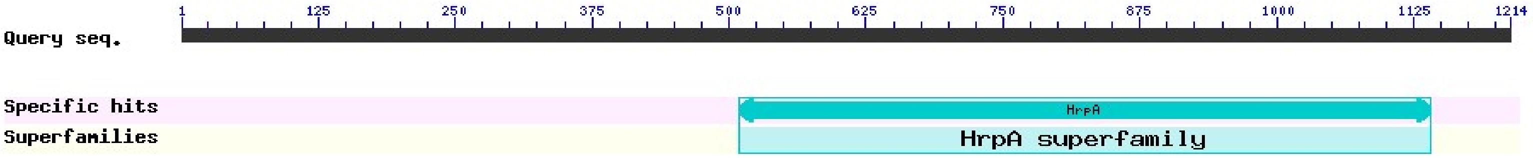

BLASTp

XP_014279344.1 pre-mRNA-splicing factor ATP-dependent RNA helicase PRP16 [*Halyomorpha halys*]

Score:2428 bits

E-value: 0.0

Query 1 MEAEDDEGVYRLEGVEQKQGGLIIKKKPEPQGSFQFKVPQTSLLGLDRLAALRRKEREND 60

MEAEDDEGVYRLEGVE+KQGGLIIKKKPEPQGSFQFKVPQTSLLGLDRLAALRRKEREND

Sbjct 1 MEAEDDEGVYRLEGVEEKQGGLIIKKKPEPQGSFQFKVPQTSLLGLDRLAALRRKEREND 60

Query 61 EKKEASKKLKEEQENKASFVKPDKHGDRHYREKYDETPTHTGGVTREAKERMEERRRKEE 120

EKKEASKKLKEE+ENKASFVKP+KHGDRHYR KYDETPTHTGGVTREAKERMEERRRKEE

Sbjct 61 EKKEASKKLKEEEENKASFVKPEKHGDRHYRAKYDETPTHTGGVTREAKERMEERRRKEE 120

Query 121 KKRGIHVTSDIKEHNENNDSYIKDFRRDRDKGN--DRHRNR--QRERDRDRDRERRRGDR 176

KKRGIHV SD+KE N+NNDSY KD+RR++DK DRHR+R RER+ +RDRER RGD+

Sbjct 121 KKRGIHVASDVKERNDNNDSYRKDYRREKDKERERDRHRDRYRAREREHERDRERSRGDK 180

Query 177 DRDRRSERRNDDRNDRRSDRSYNRSQRSWSESPRFKDEPLTPMFSVKDTPSRTGWEEDEV 236

DRDRR++RRND+RN+ RS+R +NRSQRSWSESPRFKDEPLTPMFSVKDTPSRTGWEEDEV

Sbjct 181 DRDRRNDRRNDERNEYRSERGFNRSQRSWSESPRFKDEPLTPMFSVKDTPSRTGWEEDEV 240

Query 237 S-TPAAKSSWEHPTPKTEPREDDWSMRRPTPGHRFNSWAPERSRTGATPLAGKEEEVPWG 295

S TPAAKSSWEHPTPKTEPREDDWSMRRPTP HRFNSWAPERSRTGATPLAGKEEEVPWG

Sbjct 241 SSTPAAKSSWEHPTPKTEPREDDWSMRRPTPAHRFNSWAPERSRTGATPLAGKEEEVPWG 300

Query 296 SEKDRLLWEEEQKRLDREWYGLDQGIEEGRDPFGSVSQEYITKRERQLERSKKKRMSAQQ 355

SEKDRLLWEEEQKRLDREWYGLDQGIEEGRDPFG+VSQEYITKRERQLERSKKKRMSAQQ

Sbjct 301 SEKDRLLWEEEQKRLDREWYGLDQGIEEGRDPFGAVSQEYITKRERQLERSKKKRMSAQQ 360

Query 356 RQINKDNELWERNRMLTSGVVQNLDLDEDFDEEAEARVHLLVHNIVPPFLDGRIVFTKQP 415

RQINKDNELWERNRMLTSGVVQNLDLDEDFDEEAEARVHLLVHNIVPPFLDGRIVFTKQP

Sbjct 361 RQINKDNELWERNRMLTSGVVQNLDLDEDFDEEAEARVHLLVHNIVPPFLDGRIVFTKQP 420

Query 416 EPVIPVKDPTSDMALVSRKGSALVRAYREQKERKRAQKKHWELSGTTIGNIMGVPKKKDK 475

EPVIPVKDPTSDMALVSRKGSALVRAYREQKERKRAQKKHWELSGTTIGNIMGVPKKKDK

Sbjct 421 EPVIPVKDPTSDMALVSRKGSALVRAYREQKERKRAQKKHWELSGTTIGNIMGVPKKKDK 480

Query 476 EDDKIDGDAEADYKTSQKFAEHMKDMAPASSEFARKKTIQQQRQYLPVFAVREELLNIIR 535

E+DKIDG+ +ADYKTSQKFAEHMKDMAPA+SEFA+KKTIQQQRQYLPVFAVREELLNIIR

Sbjct 481 EEDKIDGEVDADYKTSQKFAEHMKDMAPAASEFAKKKTIQQQRQYLPVFAVREELLNIIR 540

Query 536 ENSVVVVVGETGSGKTTQLTQYLHEDGYSKSGMIGCTQPRRVAAMSVAKRVSDEMASPLG 595

ENSVVVVVGETGSGKTTQLTQYLHEDGYSKSGMIGCTQPRRVAAMSVAKRVSDEMASPLG

Sbjct 541 ENSVVVVVGETGSGKTTQLTQYLHEDGYSKSGMIGCTQPRRVAAMSVAKRVSDEMASPLG 600

Query 596 KTVGYAIRFEDCTSEDTVIKYMTDGILLRESLREPDLDNYSAIIMDEAHERSLNTDVLFG 655

KTVGYAIRFEDCTSEDTVIKYMTDGILLRESLREPDLDNYSAIIMDEAHERSLNTDVLFG

Sbjct 601 KTVGYAIRFEDCTSEDTVIKYMTDGILLRESLREPDLDNYSAIIMDEAHERSLNTDVLFG 660

Query 656 LLRQVVARRQDLKLIVTSATMDSSKFSMFFGHVPVYTIPGRTFPVEVLFARNAVEDYVEA 715

LLRQVVARRQDLKLIVTSATMDSSKFSMFFGHVPVYTIPGRTFPVEVLFARNAVEDYVEA

Sbjct 661 LLRQVVARRQDLKLIVTSATMDSSKFSMFFGHVPVYTIPGRTFPVEVLFARNAVEDYVEA 720

Query 716 SVKQALQIHLQPTKGDILIFMPGQEDIEVTCEVLGERLAEIDAAPPLSILPIYSQLPSDL 775

SVKQALQIHLQPTKGDILIFMPGQEDIEVTCEVLGERLAEIDAAPPLSILPIYSQLPSDL

Sbjct 721 SVKQALQIHLQPTKGDILIFMPGQEDIEVTCEVLGERLAEIDAAPPLSILPIYSQLPSDL 780

Query 776 QAKIFQASPDGVRKCVVATNIAETSLTVDGIMYVVDSGYCKLKVYNPRIGMDALQIYPIS 835

QAKIFQASPDGVRKCVVATNIAETSLTVDGIMYVVDSGYCKLKVYNPRIGMDALQIYPIS

Sbjct 781 QAKIFQASPDGVRKCVVATNIAETSLTVDGIMYVVDSGYCKLKVYNPRIGMDALQIYPIS 840

Query 836 QANANQRSGRAGRTGPGHAYRLYTERQYKDELLTSTVPEIQRTNLANTVLLLKSLGVQDL 895

QANANQRSGRAGRTGPGHAYRLYTERQYKDELLTSTVPEIQRTNLANTVLLLKSLGVQDL

Sbjct 841 QANANQRSGRAGRTGPGHAYRLYTERQYKDELLTSTVPEIQRTNLANTVLLLKSLGVQDL 900

Query 896 LQFHFMDPPPQDNILNSLYQLWILGALDHTGSLTPLGRQMAEFPLDPPQCQMLIVSNEMG 955

LQFHFMDPPPQDNILNSLYQLWILGALDHTGSLTPLGRQMAEFPLDPPQCQMLIVSN+MG

Sbjct 901 LQFHFMDPPPQDNILNSLYQLWILGALDHTGSLTPLGRQMAEFPLDPPQCQMLIVSNQMG 960

Query 956 CTAEILIIVSMLSVPTIFYRPKGREDEADSVREKFQVPESDHLTYLNVYNQWKQNKYSST 1015

CTAEILIIVSMLSVPTIFYRPKGREDEADSVREKFQVPESDHLTYLNVYNQWKQNKYSST

Sbjct 961 CTAEILIIVSMLSVPTIFYRPKGREDEADSVREKFQVPESDHLTYLNVYNQWKQNKYSST 1020

Query 1016 WCNDHFIHAKAMRKVREVRQQLKDILDQQKMEVVSCGTDWDIVRKCICSAYFHQGARLKG 1075

WCNDHFIHAKAMRKVREVRQQLKDILDQQKMEVVSCGTDWDIVRKCICSAYFHQGARLKG

Sbjct 1021 WCNDHFIHAKAMRKVREVRQQLKDILDQQKMEVVSCGTDWDIVRKCICSAYFHQGARLKG 1080

Query 1076 IGEYVHLRTGMPCHLHPTSALFGMGFTPDYVVYHELVMTSKEYMQCVTAVDGVWLAELGP 1135

IGEYVHLRTGMPCHLHPTSALFGMGFTPDYVVYHELVMTSKEYMQCVTAVDGVWLAELGP

Sbjct 1081 IGEYVHLRTGMPCHLHPTSALFGMGFTPDYVVYHELVMTSKEYMQCVTAVDGVWLAELGP 1140

Query 1136 MFFSIKETGRSGSSKRREAMEGMEAEMEIAQKQMKERERQAEEERGRWSKRSSQVAEPGA 1195

MFFSIKETGRSGSSKRREAMEGMEAEME AQK+MKERERQAEEERGRWSKRSSQVAEPGA

Sbjct 1141 MFFSIKETGRSGSSKRREAMEGMEAEMEAAQKEMKERERQAEEERGRWSKRSSQVAEPGA 1200

Query 1196 PGSTPLRTPLRLGL 1209

PGSTPLRTPLRLGL

Sbjct 1201 PGSTPLRTPLRLGL 1214

***Clp1 homolog* (kinase)**

>TRINITY_DN2409_c0_g1_i16 len=5290

GAACTATTGTATCTGATATTAACAGTTTACTCCCTGAGAATGTTTAACAATTGTCGTGTGTTTTATAAATGAT
TTTAATTATGACTACAAGTGTTTTCAACATTGTATGTAGTAGAATACGTCTATAATTTTTAAAAATAAATAG
CCGTCGTTCGTCTACCTTTTAATTATTTATTGCTTCAATTGTAAAAAGTTTGAGTATCAACCCACTTATACGG
CTATATGCAGCTTCTGACGAATTTTCTTTTGCTTGATGGAATAGAGACATGTTTTCATCAATTAAGGGTGAA
TCCTTAGTTTCAGTATCACCCGAGACATCACAGCCTATTGGTTTATACGAAAATGTATAGTGTTAGTTTAAA
AGTAGTGTAGTGCGCATTTGAAAATCCTCTACATTTTGTTATAAATCAGTTGAAAGTATTCATGCAGATGTG
AAAGAAAGAACATTAATGGAACAAAATGTCAGCAAAAGATATGGATGGTTTTATGGGCCTTCTGTCCACAA
CAGATACAAAACAAAAGCTCGTTATTGGGCAGGATCTTTTGAACTATTTGCAGAATGGATCATCCATAGAA
TGTTCTGATATTGGTATGGTAGTTGACAGCCTTCTGCCATGGGTACAGAGCAGCAATTTCAAGGTGTCACAA
ATGGGGCTGGACCTGATGACAGAGCTGGTGCGGAGGATGGAAGGCTATTTCAAGCCGTATATTCCGACTGT
CATACCTCCTGTAGTCGACCGACTAGGTGATAGTAAGGAATTGGTTCGCGATCGCTGCCAGCTTCTATTGAG
TACATTAATGGAAGTAGGCGCGATAACTCCGCAACACCTCTGGGATCGACTAGCCCCAGCTTTTGCTCATA
AGAATTCAAACGTACGAGATGAAATAATGAAATGCCTCACTACAACATTAAACCAACATGGTGCGAATACT
GTGTGTGTTTCCCGCTTGGTGCCATCGTTAGTGAAGCTGTTGTCCGATCCCAACGCTGCCGTGAGGGACAAC
GCTCTGAACACTTTGGCCAATGTGTACAGGCATGTCGGCGAACGGCTTAGGGCAGACTTAGTCAAGAAACA
TCAGTTACCACCTACCAAAGCTCCTAACCTGATGGCGAAATTGGATGAAATTAAGGCAGCTGGAGACTTTT
TCCCTACTGCATTACAAACAACTAGTTTGTTGGATGAAGATGAGACTGATAGGAGTGCGCCGCCGTCAGCT
AGCAAACGGTCAGCCAGTGTTACACGGAAAAATGTCTCCTCTTCTGCATCAAAGCCCACGTCTCACATGCC
AGCCCCCTCTTCTTGTACTGCTACCCCAAGTTCAGTACCTAATTCAGCATCGAAATTCAGCCGGACTGGTTC
CCTGAGAAAGCCAAGTGCAGGCGGATCAGGTCCAGCCGGCGCAGTCGACGAAGATGGCTTCATCAAAGCG
TTCCACGATGTACCAAATGTACAAATATTCAGCGTAAGAGACCTGGAAGACACCCTCAATAAAGTTAGAAC
TATTATAGCGGACCCGAACCAAGAGTGGAATAAGCGTGTGGATGCTATAAAGAAGATTCGCTCTCTCCTAA
TAGCTGGTGCTAGCGGTTATGAAGAATTTTATAATCATTTAAGATTACTTGATGTTCCTTTCCAAATATCTGT
CAAGGAATTAAGATCTCAAGTAGTTAGGGAAGCGTGTGTAACGTTGGCATTTATGTCTCAACACCTGACCA
ACAGGTTTGAACATTTTGCCGAAAATCTTGTTCCAGTTTTAATAAATTTAATACAAAATTCAGCCAAGGTTA
TAGCTAGTGCTGGCCAAGTATGTATAGGTTTCATCGTACGCTATACTCACTCTAGTCGGTTAGTACCACACA
TTTGCTCAGGACTGTCATCGAAATCTCGCGAGATAAGGAGAGCTGCGTGCAAGTTCCTCCATATAATCCTTT
CCACCTGGTCTGGCCATATTCTTACCAAACACTCCACCTTACTGCAAAATGCAATAGCCTCTGCGATCGCCG
ACGCAGATCAGGAAGTTCGTGGAACTGCAAGGAAGGCTTATTGGTCATTTAAAGATCATTTTCCCAATCTT
GCCGAAAATCTTTTGGACAATTTAGATGCCACTTACAAACGAAGCCTTCACTCTGACATGTCCAATTCTAGT
TCTTCCAATTCCCTTCATCATCAACCAACTTCTGCCACCCGAAGACAAGGAAGCACTGAAAATCTCACAAA
CGGAAGGAGAAGCAGTAGTTCATCTATACCGGCAGCAAGGCGCATAGAATCAGAAGCAGTTACTCCTCTCT
CAAAGAGAATTCCCATAGGGTCTCCGAGAACAGCTTCCCCTCGCTCTAACTCAGCTATAGATCTCCAAGCG
GCTCAGCGAGCCAAAGCAAGAGCACAATACGCAGCTTTAGCTAGGCAAAAAGTCGGATCCAACACTAGTC
TTCCTCGCCCAAAGAAAACAGGTACCGAATCTGGGCCGAGTTCCTTAGCATTTTCTCCTGAACACACGAGG
CAGGGAAGGAGTAGGACAACGGGAATCAGCCAGTCACAGCCCTCAAGCAGGTCTGGATCACCCTCATCAC
GATTGAATTACGCAACTTATTCATCTTGCTTGGGAGAGCGATCCAGGCGGTCCCCGTCGTATACGACACGCT
CAACTCAAGGCTCTCGAGAAACGTCACCTTCTCGATACAACCCGCTTCCTCCCCTCGGAAAGCTGAGGGGC
GTTCGTCCTCCTCTTTCCAGGCCTGTCATGGCTCACAAGATTTTACAACAGTCTTTGGAAGCCGAATCAGCT
CTAGCGGATGCTTTAGGATACACGGATGGACGAGGTGACACCAGTGGTAGCTGCCTGACCTCCCCTCGTAG
GTTGAGATTGGACGATCATTCGGACGATTCTGAGACATCCAGTGTTTGTTCAGAACGATCTTTTGAATCTCA
TCGGAGACCCTCTGATTCCTACTCCTGGAGTGGTTCGCAGCAAAGGCTCTACCGAGACATGTGGGAACCAG
CCATTAAGGACATAAATGAAATAATGGAAGCTTGCGAGTCTACCCATTGGTCCGATAGAAAGGATGGTTTA

GTCTCTTTATCCGCTTATCTTCAAGCTGGTAACACTTTGTCACCACACCAGCTCAATAGAATCACTGAAAAT
TTTACAAAAATGCTCTCCGATACACATACCAAGGTTTTCTCCCTTTTCCTGGATACAGTATCAGACCTCGTTT
CGACTCACTCCGCCGACCTCCATCCCTGGCTTTACATTCTGTTAACTAGGCTCTTTAATAAACTCGGAGGAG
ATTTGTTATCCTCAATACAGACTAAAATTCAGAAAACCCTCGGAGTCGTCAAGTCAAGCTTTGGTCCCGAA
CCCCTTTTACATTGGGCTTTGAGATTCCTGGTAGATCCGACGCAGACTCCGAACACTCGAGTGAAGTTAGCT
GTTCTAGGTTTCATCGCCAAGCTTGCTCCTGCTGCCGATCCATCTTCTGCCTTCCCTCCCGCCTCGCCCTCTT
CATCAGGACAATCCAAAGATGTTACTACGACTGCTCTGACAAAAATGATCGGTTGGACAATGGGAGAAGG
AATCAAACAAGGAGGAGAGCTGAGGCGTGGAGCTCAGGAAGCCATCTTAGCCCTTTTTAATTTGAACACTC
CGCAAGTAACGTTGAGGCTGTCGCAGCTACCTAAAGAGTACCAGGAAGCTGCCGGAAGTTTGATACGAAT
GAGGCGTGGCAGCGGAGGCGGAGGAGACTCGCTTAGCCCTGATCAGGTTTATCGCTCTCTACGTCGCACTA
CCGCCGAAATACAAAGTTACTCTTACGACACTAACGGAGGGAACAAAGTTGCGGATACTGCTTCTCACGAC
TCGGGCATTAGTCAAATGTCGGATAAGCATGATGACATTCTTTGGGGCATGAGTTCTCTGTCACTATCATCA
CCTACAAGGTCCGGCAAAGACTACAACGGCCTAGAAACTACTGATTCACCCACCTCACTTGGTAACGGTTT
TTCCAAAGAGGGTGGTAGTAGTGAAGCCGGGGATGCTTTAAGCAAGCTGCTAGATACGATAGAGAACAGC
AGCGGAGCAGAGAGGAAGGCTGCATTCGATCAACTTGCCACGCTTATCAGGGAGTCGGATCCCATCACGTT
AAATACACATTTCAGGAAGATACTGAAGGGTGTGCTGGGGAAATCCGGATTGTTAGGAGAAGCAGATTCTA
GATTGGGCGGACTGACGGCGCTGTCGGAGGTAATGAAGAGGAAGTGCCTCTGGTCCTCGCTCCAGACCTAC
GTCGAGCTCATCCTCTTGAGGGTGATTGACGCGTACTCGGACGGGAACAAGGAGGTGTCCAAACTCGCAGA
GCAGGCCGCGCTTCTCGTTATTTCAGCTTTAGATCCAGCAGATGTCATTAAGGTATTAGTTCCATTGATGAG
GGCAGAAGAATTTCCTAAAAATCTTATGGCCATTAAAACTCTGACGAAACTTGTCGAAATGGAAACAAAAG
AAGTTATTATGCCGTTTCTTCCCGACATAATGCCAGGACTTGTTCAGGCCTATGCTAACGAAGAGAGCTTAG
TTAGAAAATCAACTGTGTTTTGTATGGTCGCACTTCATAACAAACTCGGAGGAGAGACGCTTAAGCCATAC
CTGGCTTCTCTTAACGCAAGCAAACTCAAGCTTTTACATTTGTATATAAATAGGACTCAAACAGATCAGTCG
CCCAGTTCCCCCAAGCCTGCTGCAGCCGTGTAATGTGCCATCCCAATTATTTCGCTGATATATATTTTTATAT
TCATCGTAATTGCTTTGTAAAAAACAAAAATTGGAAAAAAAAAAATATTAGGGAAGATACATTTAGTAATAC
AATATAACATAATAACACTTTTCATTATTTGTAGAATCAGTTCTCAGTTGTACAGCTTATTATGCACATATTT
AATTTGTAAGTATATTTACTTTTTATTAATAAAAGTCTTATCGTAACTAATGTCGACATGTATTTAATTAAAT
ATGTTTAATACGAACAAAGTTGGATACATTATTTATTGGCTTTTTTTTCTCTTGTTATATATGAATTATAATG
TTGAAAGACTAATCTATGCCTTATTTATAACATTTATAAGATCTTAACGTGGCCAATCGTTGAGCAAATACT
CATCCACAAATATGCTATATGTAGAATAAAACCTGGATTAATTTATTTTTGGCCAGAATGCTTCTCTGTTAT
CTTTTTTTTTTTTTTTTTTTTAATGGATTAACCAAATTATTCATTATAAAATTTAACTATTTACTTACTA

Protein

RF: +1

ORF: 460 -> 4746

Length: 1428 aa

>lcl|ORF1_TRINITY_DN2409_c0_g1_i16:459:4745 unnamed protein product

MSAKDMDGFMGLLSTTDTKQKLVIGQDLLNYLQNGSSIECSDIGMVVDSLLPWVQSSNFKVSQMGLDLMTELV
RRMEGYFKPYIPTVIPPVVDRLGDSKELVRDRCQLLLSTLMEVGAITPQHLWDRLAPAFAHKNSNVRDEIMKCL
TTTLNQHGANTVCVSRLVPSLVKLLSDPNAAVRDNALNTLANVYRHVGERLRADLVKKHQLPPTKAPNLMAK
LDEIKAAGDFFPTALQTTSLLDEDETDRSAPPSASKRSASVTRKNVSSSASKPTSHMPAPSSCTATPSSVPNSASKF
SRTGSLRKPSAGGSGPAGAVDEDGFIKAFHDVPNVQIFSVRDLEDTLNKVRTIIADPNQEWNKRVDAIKKIRSLLI
AGASGYEEFYNHLRLLDVPFQISVKELRSQVVREACVTLAFMSQHLTNRFEHFAENLVPVLINLIQNSAKVIASA
GQVCIGFIVRYTHSSRLVPHICSGLSSKSREIRRAACKFLHIILSTWSGHILTKHSTLLQNAIASAIADADQEVRGTA
RKAYWSFKDHFPNLAENLLDNLDATYKRSLHSDMSNSSSSNSLHHQPTSATRRQGSTENLTNGRRSSSSSIPAAR
RIESEAVTPLSKRIPIGSPRTASPRSNSAIDLQAAQRAKARAQYAALARQKVGSNTSLPRPKKTGTESGPSSLAFSP
EHTRQGRSRTTGISQSQPSSRSGSPSSRLNYATYSSCLGERSRRSPSYTTRSTQGSRETSPSRYNPLPPLGKLRGVR
PPLSRPVMAHKILQQSLEAESALADALGYTDGRGDTSGSCLTSPRRLRLDDHSDDSETSSVCSERSFESHRRPSDS

YSWSGSQQRLYRDMWEPAIKDINEIMEACESTHWSDRKDGLVSLSAYLQAGNTLSPHQLNRITENFTKMLSDT
HTKVFSLFLDTVSDLVSTHSADLHPWLYILLTRLFNKLGGDLLSSIQTKIQKTLGVVKSSFGPEPLLHWALRFLVD
PTQTPNTRVKLAVLGFIAKLAPAADPSSAFPPASPSSSGQSKDVTTTALTKMIGWTMGEGIKQGGELRRGAQEAI
LALFNLNTPQVTLRLSQLPKEYQEAAGSLIRMRRGSGGGGDSLSPDQVYRSLRRTTAEIQSYSYDTNGGNKVAD
TASHDSGISQMSDKHDDILWGMSSLSLSSPTRSGKDYNGLETTDSPTSLGNGFSKEGGSSEAGDALSKLLDTIEN
SSGAERKAAFDQLATLIRESDPITLNTHFRKILKGVLGKSGLLGEADSRLGGLTALSEVMKRKCLWSSLQTYVEL
ILLRVIDAYSDGNKEVSKLAEQAALLVISALDPADVIKVLVPLMRAEEFPKNLMAIKTLTKLVEMETKEVIMPFL
PDIMPGLVQAYANEESLVRKSTVFCMVALHNKLGGETLKPYLASLNASKLKLLHLYINRTQTDQSPSSPKPAAA
V

Conserved Domains

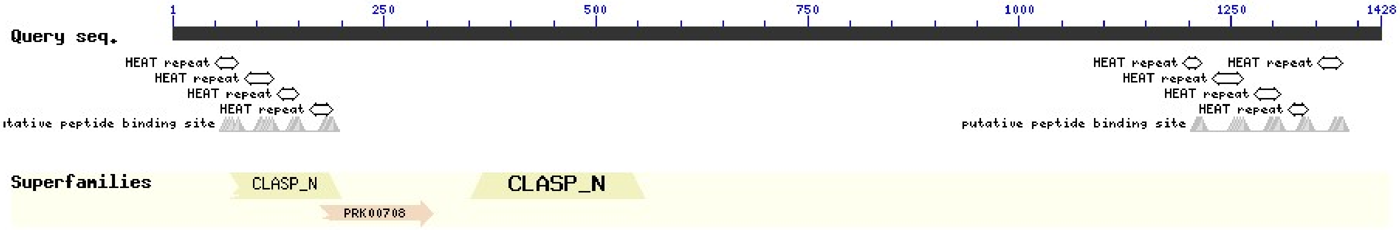

BLASTp

XP_014275582.1PREDICTED: CLIP-associating protein [*Halyomorpha halys*]

Score:2821 bits

E-value: 0.0

Query 1 MSAKDMDGFMGLLSTTDTKQKLIIGQDLLNYLQNGSSIECSDIGMVVDSLLPWIQSSNFK 60

MSAKDMDGFMGLLSTTDTKQKL+IGQDLLNYLQNGSSIECSDIGMVVDSLLPW+QSSNFK

Sbjct 1 MSAKDMDGFMGLLSTTDTKQKLVIGQDLLNYLQNGSSIECSDIGMVVDSLLPWVQSSNFK 60

Query 61 VSQMGLDLMTELVRRMEGYFKPYIPTVIPPVVDRLGDSKELVRDRCQLLLSTLMEVGAIT 120

VSQMGLDLMTELVRRMEGYFKPYIPTVIPPVVDRLGDSKELVRDRCQLLLSTLMEVGAIT

Sbjct 61 VSQMGLDLMTELVRRMEGYFKPYIPTVIPPVVDRLGDSKELVRDRCQLLLSTLMEVGAIT 120

Query 121 PQHLWDRLAPAFAHKNSNVRDEIMKCLTTTLNQHGANTVCVSRLVPSLVKLLSDPNAAVR 180

PQHLWDRLAPAFAHKNSNVRDEIMKCLTTTLNQHGANTVCVSRLVPSLVKLLSDPNAAVR

Sbjct 121 PQHLWDRLAPAFAHKNSNVRDEIMKCLTTTLNQHGANTVCVSRLVPSLVKLLSDPNAAVR 180

Query 181 DNASGTLANVYRHVGERLRADLTKKHQLPPTKAPNLMAKLDEIKAAGDFFPTAHHATSEI 240

```
           DNA  TLANVYRHVGERLRADL KKHQLPPTKAPNLMAKLDEIKAAGDFFPTA   TS +
Sbjct  181   DNALNTLANVYRHVGERLRADLVKKHQLPPTKAPNLMAKLDEIKAAGDFFPTALQTTSLL  240

Query  241   KC--LILFQPPSATKRSASVTRKNVSSSASKPTSGMPAPSSCTATPSSVPNSASKFSRTG  298
                   PPSA+KRSASVTRKNVSSSASKPTS MPAPSSCTATPSSVPNSASKFSRTG
Sbjct  241   DEDETDRSAPPSASKRSASVTRKNVSSSASKPTSHMPAPSSCTATPSSVPNSASKFSRTG  300

Query  299   SLRKPSAAGSGPAGAVDEDGFIKAFHDVPNVQIFSVRDLDDTLNKVRAVIADPNQEWNKR  358
             SLRKPSA GSGPAGAVDEDGFIKAFHDVPNVQIFSVRDL+DTLNKVR +IADPNQEWNKR
Sbjct  301   SLRKPSAGGSGPAGAVDEDGFIKAFHDVPNVQIFSVRDLEDTLNKVRTIIADPNQEWNKR  360

Query  359   VDAIKKIRSLLIAGASSYEEFYNHLRLLDVPFQISVKELRSQVVREACVTLAFMSQHLTN  418
             VDAIKKIRSLLIAGAS YEEFYNHLRLLDVPFQISVKELRSQVVREACVTLAFMSQHLTN
Sbjct  361   VDAIKKIRSLLIAGASGYEEFYNHLRLLDVPFQISVKELRSQVVREACVTLAFMSQHLTN  420

Query  419   RFEHFAENLVPVLINLIQNSAKVIASAGQVCIGFIVRYTHSSRLVPHICSGLSSKSREIR  478
             RFEHFAENLVPVLINLIQNSAKVIASAGQVCIGFIVRYTHSSRLVPHICSGLSSKSREIR
Sbjct  421   RFEHFAENLVPVLINLIQNSAKVIASAGQVCIGFIVRYTHSSRLVPHICSGLSSKSREIR  480

Query  479   RAACKFLHIILSTWSGHILTKHSALLQSAIASAIADADQEVRGTARKAYWAFKDQFPNLA  538
             RAACKFLHIILSTWSGHILTKHS LLQ+AIASAIADADQEVRGTARKAYW+FKD FPNLA
Sbjct  481   RAACKFLHIILSTWSGHILTKHSTLLQNAIASAIADADQEVRGTARKAYWSFKDHFPNLA  540

Query  539   ESLLDNLDANYKRSLHSDMSNSSSSNSLHHQPTTATRRLGSTENLTNGRRSSSSSITTSR  598
             E+LLDNLDA YKRSLHSDMSNSSSSNSLHHQPT+ATRR GSTENLTNGRRSSSSSI  +R
Sbjct  541   ENLLDNLDATYKRSLHSDMSNSSSSNSLHHQPTSATRRQGSTENLTNGRRSSSSSIPAAR  600

Query  599   RIESEAVTPLSKRIPIGSPRTASPRSNSAIDLQAAQRAKARAQYAALARQKVGSNTSLPR  658
             RIESEAVTPLSKRIPIGSPRTASPRSNSAIDLQAAQRAKARAQYAALARQKVGSNTSLPR
Sbjct  601   RIESEAVTPLSKRIPIGSPRTASPRSNSAIDLQAAQRAKARAQYAALARQKVGSNTSLPR  660
```

Query 659 PKKTGTESGPSSLAFSPEHTRQGRSRTTGISQSQPSSRSGSPSSRLNYATYSSCLGERSR 718
PKKTGTESGPSSLAFSPEHTRQGRSRTTGISQSQPSSRSGSPSSRLNYATYSSCLGERSR
Sbjct 661 PKKTGTESGPSSLAFSPEHTRQGRSRTTGISQSQPSSRSGSPSSRLNYATYSSCLGERSR 720

Query 719 RSPSYTTRSTQGSRETSPSRYNPLPPLGKLRGVRPPLSRPVMAHKILQQSLEAESALADA 778
RSPSYTTRSTQGSRETSPSRYNPLPPLGKLRGVRPPLSRPVMAHKILQQSLEAESALADA
Sbjct 721 RSPSYTTRSTQGSRETSPSRYNPLPPLGKLRGVRPPLSRPVMAHKILQQSLEAESALADA 780

Query 779 LGYTDGRGDTSGSCVTSPRRLRLDDHSDDSETSSVCSERSFESHRRPSDSYSWSGSQQRL 838
LGYTDGRGDTSGSC+TSPRRLRLDDHSDDSETSSVCSERSFESHRRPSDSYSWSGSQQRL
Sbjct 781 LGYTDGRGDTSGSCLTSPRRLRLDDHSDDSETSSVCSERSFESHRRPSDSYSWSGSQQRL 840

Query 839 YRDMWEPAIKDINEIMEACESTHWSDRKDGLVSLSAYLQAGNTLSPHQLNRITENFKSMF 898
YRDMWEPAIKDINEIMEACESTHWSDRKDGLVSLSAYLQAGNTLSPHQLNRITENF M
Sbjct 841 YRDMWEPAIKDINEIMEACESTHWSDRKDGLVSLSAYLQAGNTLSPHQLNRITENFTKML 900

Query 899 ILFFFQVFSLFLDTVSDLVSTHSADLHPWLYILLTRLFNKLGGDLLSSIQTKIQKTLGVV 958
+VFSLFLDTVSDLVSTHSADLHPWLYILLTRLFNKLGGDLLSSIQTKIQKTLGVV
Sbjct 901 SDTHTKVFSLFLDTVSDLVSTHSADLHPWLYILLTRLFNKLGGDLLSSIQTKIQKTLGVV 960

Query 959 KSSFGPEPLLHWALRFLVDPTQTPNTRVKLAVLGFIAKLAPAADPSSAFPPASPSTSGQS 1018
KSSFGPEPLLHWALRFLVDPTQTPNTRVKLAVLGFIAKLAPAADPSSAFPPASPS+SGQS
Sbjct 961 KSSFGPEPLLHWALRFLVDPTQTPNTRVKLAVLGFIAKLAPAADPSSAFPPASPSSSGQS 1020

Query 1019 KDVTTTALTKMIGWTMGEGIKQGGELRRGAQEAILALFNLNTPQVTLRLSQLPKEYQEAA 1078
KDVTTTALTKMIGWTMGEGIKQGGELRRGAQEAILALFNLNTPQVTLRLSQLPKEYQEAA
Sbjct 1021 KDVTTTALTKMIGWTMGEGIKQGGELRRGAQEAILALFNLNTPQVTLRLSQLPKEYQEAA 1080

Query 1079 GSLIRMRRGSGGGGDSLSPDQVYRSLRRTTAEIQSYSYDTNGGNKVADTASHDSGISQMS 1138
GSLIRMRRGSGGGGDSLSPDQVYRSLRRTTAEIQSYSYDTNGGNKVADTASHDSGISQMS
Sbjct 1081 GSLIRMRRGSGGGGDSLSPDQVYRSLRRTTAEIQSYSYDTNGGNKVADTASHDSGISQMS 1140

Query 1139 DKHDDILWGMSSLSLSSPTRSGKDYNGLETTDSPTSLGNGFSKEGGSSEAGDALSKLLDT 1198

DKHDDILWGMSSLSLSSPTRSGKDYNGLETTDSPTSLGNGFSKEGGSSEAGDALSKLLDT

Sbjct 1141 DKHDDILWGMSSLSLSSPTRSGKDYNGLETTDSPTSLGNGFSKEGGSSEAGDALSKLLDT 1200

Query 1199 IENSSGAERKAAFDQLATLIRESDPITLNTHFRKILKGVLGKSGLLGEADSRLGGLTALS 1258

IENSSGAERKAAFDQLATLIRESDPITLNTHFRKILKGVLGKSGLLGEADSRLGGLTALS

Sbjct 1201 IENSSGAERKAAFDQLATLIRESDPITLNTHFRKILKGVLGKSGLLGEADSRLGGLTALS 1260

Query 1259 EVMKRKCLWSSLQTYVELILLRVIDAYSDGNKEVSKLAEQAALLIISALDPADVIRVLVP 1318

EVMKRKCLWSSLQTYVELILLRVIDAYSDGNKEVSKLAEQAALL+ISALDPADVI+VLVP

Sbjct 1261 EVMKRKCLWSSLQTYVELILLRVIDAYSDGNKEVSKLAEQAALLVISALDPADVIKVLVP 1320

Query 1319 LMRAEEFPKNLMAIKTLTKLVEMETKEVILPFLPDIMPGLVQAYANEESLVRKSTVFCMV 1378

LMRAEEFPKNLMAIKTLTKLVEMETKEVI+PFLPDIMPGLVQAYANEESLVRKSTVFCMV

Sbjct 1321 LMRAEEFPKNLMAIKTLTKLVEMETKEVIMPFLPDIMPGLVQAYANEESLVRKSTVFCMV 1380

Query 1379 ALHNKLGGETLKPYLASLNASKLKLLHLYINRTQTDQSPSSPKPAAAV 1426

ALHNKLGGETLKPYLASLNASKLKLLHLYINRTQTDQSPSSPKPAAAV

Sbjct 1381 ALHNKLGGETLKPYLASLNASKLKLLHLYINRTQTDQSPSSPKPAAAV 1428

Elp-1

>TRINITY_DN1844_c0_g1_i1 len=3901 path=[0:0-3900]

CTCCCATCACGTTTGAAATTTACCACAATGAAGAATTTAAAACTTTTGCTAAATTTGAAAATACACTGTGAT
GAATTTGTGGACGCTTCTCTAATAGTTTGTGGACAAACATCTTCTGACAATGATTTGTCTTCTAAACTCAAA
AATGTTTTTGTGATATCGAATAATATATTGTATTCTGTCGATTTGATCTCTGAAAAACCCTCGAAATATTAC
GATTTAGCCAGTCTATTTCCTGATGAAAATGTCCCAAAGGCAGTTAGTATGTGGTACAGAGATTTGTATAAT
ACAATTTATGTATGTTTTGAAAATGGGGACTTATACTCACTCATATTAGATGATAATGGAGTGCAACATTGT
AGTTTCAACCAGCAGTTGGGATGCAAACCTATTGATATTCATTGGAGTCCTGACGAAGAACTTGTAGTGGTT
GTCTCAGTAAGTGGGTACTTGATTTTAACAAATTCAGATTTTTTTATAGTTGATGAGGTGGATCTTCAAGAT
TCCTCTCGTGGAGAAAATGAACTGATTAGTGTTGGTTGGGGGAAAAAAGAAACCCAGTTTCATGGATCTGA
AGGAAAGAATGCAGCTAAATTAAAACCTACTACTGTCGATCCTTCATCTATTGAAGACGATCATTCCGTCC
ATGTGACTTGGAGAGGAGACAGTAATTTATTTGCTGCTAGTTATTGGTGCGCTCAAACGAATATGAGGAAA
GTGAAAATTTTTAATAGGGATGGAGATTTACAGAGCACAAGTGAAGAAGTTCCTGGTTTAAATGAACCTAT
CTGTTGGAGGCCGAGTGGAAATTTAATCGCTCTTCCTCAACAGTTACCGAACAAAAAAGTAGTTTCTTTTTT
GGAAAAAAATGGACTCAAGCATGGAGACTTCACTTTACCACCTGGCTTAAAAGTCAAACACATATCTTGGA
ATGAAGGATCAAACATTCTTTGTACGATTTGTGAAAATCAACAAAACCTAGGTGAAGAAGTGATGTTATGG

ACCACAGGAAATTATCATTGGTATATAAAACAGAAGTTCAAATTTGTTTCCTTGGCTCGTTACGTTTGGTGG
GATAAAGAACGCCCCAACAGACTTTATGCTCTGTGTGAAGGAGGAATTTTGCAGATATTGGAATGGACGTT
TATTGTAGATACTAGTTATTATTCACGATGTGAGGACAAATCGTATGTCAGTGTTATTGATAACGATTCTAT
TTTGCTTACATCATTCAAAGAAGCAGTTATACCACCGCCAATGTCTTCGTACAAACTGAAATGTCCAGATGC
TGTAAATCATGTGATGTTTGCTCCACTTGATGGAGATGGACACGATCTTCATATGTTATGTGCGATTCTATTT
AATGGTGATGTTGCATTTTTCAAAGACCCACTGTCAAGTCCTACAATTTTTTCAGATTTGGTAAAGAAGCAC
AATTTTTCGCAGGAGATGATTTCGCTATCTCATTGGGATTGGGTTAGCTCAGATTCTATAGTTTGTTGTTCTT
CAGTTAAACGGGAAACTCATATACATCAATTCTCATTATCCCAAGATGGTCTGGAATTAAGAAATGTTACA
AAGTTAGAAGGAAATATCATAACATGCAACCGAAGAGCAGAAAAACTTGTAATTCAAACAACTAATGGAA
AGTTATTCAATTACAATACAAAGTCTTTAGTTGTTGTCGAGACTTTATCTCTAATAGAACCATGTTACGACA
TCAAAGTGCATTCTTCCGGGCTGTTCTGTTTGTCAGAATTTTCCAGACTATACCTAAATGACACCCCTATCA
ACTTGCCTTCGATAACTTGCAAGGTCACCTCTTTTATCCTAAAGGATCCTTATTTATTAGTGACAACATCATC
TCACAAACTAGTGATTTTACAGTGCTCTGATTCAGAGAATGTAGAAATATCTGAACGGAAGTTGGAAAGAG
GTTCTAGACTGGTTACTACTTTTGATAACTCAGTCGTATTGCAAGCACCAAGGGGGAACTTGGAAACTATA
CAACCGCGAGCCCTTACCATTCTTACTCTTGGTACACTCATCGATTCGAAACAGTTCAAAACCGCATTCTCT
CTTATGAGGAAGCAGAGGATTGACCTCAATCTTATCATCGACCATAACCAAAAACTTTTCTTAAATGAAGT
TAACAATGTTGTGAACCAAATCGACCCTCAGTGGATTACTCTTCTAATAACTGAACTTTCAGCCGAGGATGT
TACTACAAATTTGTACAAGCAATATTATAAGAAAAAGGATACTTCCTTTCTAAATGGTTCAAAAGTGAAAT
CTGTCTGCGAAGCTCTGCTATCAGCCTTGTCAGTTGAGGATTTAAAGAAGAAATACATTTTTCCGATTCTGA
GCGCTTTGGTTAAAATTGGTGAAATGAGTAAAGCTATTCAGCTTGCTAGCAATGAAGTCGCAATGCAACAT
TTAATGTTCATTGTCGATTCTAATAAATTGTATATGGAAGCGCTAGGCGTTTATGATTTGAATGCTGCCTTA
AAAATCGCTGAAAAATCGCAGAAAGATCCTAAGGAATATATCCCTTACTTAAATTCACTGAAATCCTTGGA
ATCCAATTACATGAGATTTACAATAGATAAAAAATTAAAGAGAAACGAATCTGCTTTAAATCATATCAGCA
AATGTTGTGACGATCATGAAGAAGAATGTCTAAAGTTTATCGAGGAAAATGCATTGTATCCTGCTGCATTG
AACATATTTAAAAATGATTTGAAAAGGTATTCAATTATTGCTTTCAAGTACGCCGATTACTTATTCAAGCAG
AGGCATTATGAGGAATCCGGTATAATGTACCTGCGTAGTGATGATAATGAGAAAGCTCTTCAGTCATTTAC
GAGAGCTGGAAACTGGAGGCAGTGTATGATCATCGTTATGGATTCTAATTATAGTGATGAAGAGCAGAGAA
AACATGCTGAAAATGTTTGTAATATGTTAATATCTGCACATAAATATTCAGAAGCCGCAATCCTGTACACA
GAATGGTTATTAGATGCTGATAAAGCTGTCGAAATGTATACTCAAGCAAGATGTTGGAAAGAAGCAATCTA
TACTGCTAAGAAATTTAAAAAGCCTAGTTTGATTGACTCCATTATCAAAGATGCTTTAATTGAAACTGCTAA
TATCCTAGTTGAAGACATTCATTCTCAATCAGAGAAAACCAAGAAGTACGTTGAGAGGTTAAAAACTGTCA
GAATTGAGAAAGAAAATCGTGCTGCTTATATTTTTGATGACATGAGTGATACTGCTTCTGATACTAGCAGCA
CTTCTTCAGTTCGAACTAAATCATTCCAATCGAGGTCTAGCAAAAACACCAAAAAGATGAGCAGAAAAATG
TGGAGTTTAAAAGAAGGCAACCCAAGGGAAGAAGAAGCCCTTGTTGCAACATTGAGTAGTATTATTTCTTC
TACAGAAAAATATGTTGGTGAAATTAACTCAGCATGCTGCACACTGCTGGTGTTCCGAGAGGATCCACTTG
CTGCGAAACTACAATCAGCACTAGAAGAATGGCTATCTATAATCGATAATTCTAAAAACATCATTTGGCCT
AAGGAACCTGGAACAAATGAAGCATCTGTTTTGGATGAGAAATTCAAGTTCCCACCTGAAGTCAGTATAAA
ACAAGACTGGAAATTTAATTTGCTAAAGTCATAGCCCATCTTTTAAAAGAGTTTTATTATTTAAAAAAATAA
TTATTCTCACCTATTCATTGGAATGAATAATTAGAGGAGAATAAAATGTACATACTAAAATTTAATTCATAG
AGAAGAAAATAATTTGTTATAATTCTTGTTGAATGTTGTAAATTTTTTTATGTATGATTTTTTCCAATAAT
GTAAATTTAAATAAAAACTTATATTTTTAAAAAAAAA

Protein

RF: +1

ORF: 28 -> 3681

Length: 1217 aa

>lcl|ORF1_TRINITY_DN1844_c0_g1_i1:27:3680 unnamed protein product

MKNLKLLLNLKIHCDEFVDASLIVCGQTSSDNDLSSKLKNVFVISNNILYSVDLISEKPSKYYDLASLFPDENVPK
AVSMWYRDLYNTIYVCFENGDLYSLILDDNGVQHCSFNQQLGCKPIDIHWSPDEELVVVVSVSGYLILTNSDFFI

VDEVDLQDSSRGENELISVGWGKKETQFHGSEGKNAAKLKPTTVDPSSIEDDHSVHVTWRGDSNLFAASYWCA
QTNMRKVKIFNRDGDLQSTSEEVPGLNEPICWRPSGNLIALPQQLPNKKVVSFLEKNGLKHGDFTLPPGLKVKHI
SWNEGSNILCTICENQQNLGEEVMLWTTGNYHWYIKQKFKFVSLARYVWWDKERPNRLYALCEGGILQILEWT
FIVDTSYYSRCEDKSYVSVIDNDSILLTSFKEAVIPPPMSSYKLKCPDAVNHVMFAPLDGDGHDLHMLCAILFNG
DVAFFKDPLSSPTIFSDLVKKHNFSQEMISLSHWDWVSSDSIVCCSSVKRETHIHQFSLSQDGLELRNVTKLEGNII
TCNRRAEKLVIQTTNGKLFNYNTKSLVVVETLSLIEPCYDIKVHSSGLFCLSEFSRLYLNDTPINLPSITCKVTSFIL
KDPYLLVTTSSHKLVILQCSDSENVEISERKLERGSRLVTTFDNSVVLQAPRGNLETIQPRALTILTLGTLIDSKQF
KTAFSLMRKQRIDLNLIIDHNQKLFLNEVNNVVNQIDPQWITLLITELSAEDVTTNLYKQYYKKKDTSFLNGSKV
KSVCEALLSALSVEDLKKKYIFPILSALVKIGEMSKAIQLASNEVAMQHLMFIVDSNKLYMEALGVYDLNAALK
IAEKSQKDPKEYIPYLNSLKSLESNYMRFTIDKKLKRNESALNHISKCCDDHEEECLKFIEENALYPAALNIFKND
LKRYSIIAFKYADYLFKQRHYEESGIMYLRSDDNEKALQSFTRAGNWRQCMIIVMDSNYSDEEQRKHAENVCN
MLISAHKYSEAAILYTEWLLDADKAVEMYTQARCWKEAIYTAKKFKKPSLIDSIIKDALIETANILVEDIHSQSEK
TKKYVERLKTVRIEKENRAAYIFDDMSDTASDTSSTSSVRTKSFQSRSSKNTKKMSRKMWSLKEGNPREEEALV
ATLSSIISSTEKYVGEINSACCTLLVFREDPLAAKLQSALEEWLSIIDNSKNIIWPKEPGTNEASVLDEKFKFPPEVS
IKQDWKFNLLKS

Conserved Domains

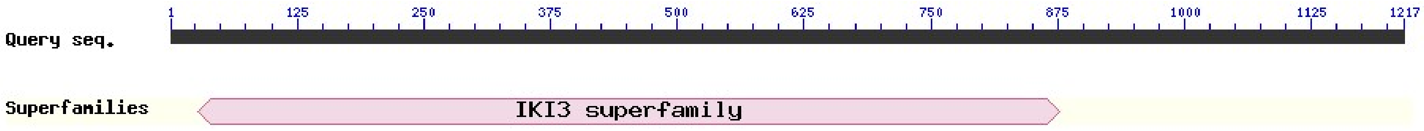

BLASTp

XP_014290480.1 elongator complex protein 1 [*Halyomorpha halys*]

Score: 2104 bits

E-value: 0.0

Query 12 VHCDVLADTSLIVGGQTSIDNDLSVKLKNVFVISKNTLYSVDLISGIASKYYDLASLFSD 71

+HCD D SLIV GQTS DNDLS KLKNVFVIS N LYSVDLIS SKYYDLASLF D

Sbjct 12 IHCDEFVDASLIVCGQTSSDNDLSSKLKNVFVISNNILYSVDLISEKPSKYYDLASLFPD 71

Query 72 KDVPKAMSMWYRDLYNTIYICFENGEIFSLILDNEKVEHSSIIHDLECKPLDIQWSPDEE 131

++VPKA+SMWYRDLYNTIY+CFENG+++SLILD+ V+H S L CKP+DI WSPDEE

Sbjct 72 ENVPKAVSMWYRDLYNTIYVCFENGDLYSLILDDNGVQHCSFNQQLGCKPIDIHWSPDEE 131

Query 132 HAAVVCGNDSLILLNSEFFILAEIDLNDSLPGENELISVGWGKKETQFHGSEGKNAAKSK 191

VV + LIL NS+FFI+ E+DL DS GENELISVGWGKKETQFHGSEGKNAAK K

Sbjct 132 LVVVVSVSGYLILTNSDFFIVDEVDLQDSSRGENELISVGWGKKETQFHGSEGKNAAKLK 191

Query 192 PSSIDPSFIEDNHSLRVTWRGDGNLFAASYWCSKTSMRRVKIFNMDGVLLSTSEDIPGLN 251

P+++DPS IED+HS+ VTWRGD NLFAASYWC++T+MR+VKIFN DG L STSE++PGLN

Sbjct 192 PTTVDPSSIEDDHSVHVTWRGDSNLFAASYWCAQTNMRKVKIFNRDGDLQSTSEEVPGLN 251

Query 252 EPICWRPSGNLIALPQQLPNKKVVSFLEKNGLKHGDFTLPSDVKVKHISWNEGSTILCTI 311

EPICWRPSGNLIALPQQLPNKKVVSFLEKNGLKHGDFTLP +KVKHISWNEGS ILCTI

Sbjct 252 EPICWRPSGNLIALPQQLPNKKVVSFLEKNGLKHGDFTLPPGLKVKHISWNEGSNILCTI 311

Query 312 CENNEYLGEEVMLWVTGNYHWYIKQKFKFVSQTRNVWWDKERPNRMYALCEGGILQILEW 371

CEN + LGEEVMLW TGNYHWYIKQKFKFVS R VWWDKERPNR+YALCEGGILQILEW

Sbjct 312 CENQQNLGEEVMLWTTGNYHWYIKQKFKFVSLARYVWWDKERPNRLYALCEGGILQILEW 371

Query 372 TFVVNNSYHSHCEDKSFVSVIDNDSILLTSFKEAVIPPPMSSYKLICPSAVNHVMFAPFD 431

TF+V+ SY+S CEDKS+VSVIDNDSILLTSFKEAVIPPPMSSYKL CP AVNHVMFAP D

Sbjct 372 TFIVDTSYYSRCEDKSYVSVIDNDSILLTSFKEAVIPPPMSSYKLKCPDAVNHVMFAPLD 431

Query 432 KDQPSLHMLCAVLFNGDVAFFKDPLSSPTILSDLVQKYNFSEEIISLSHWDWVSSDTFIF 491

D LHMLCA+LFNGDVAFFKDPLSSPTI SDLV+K+NFS+E+ISLSHWDWVSSD+ +

Sbjct 432 GDGHDLHMLCAILFNGDVAFFKDPLSSPTIFSDLVKKHNFSQEMISLSHWDWVSSDSIVC 491

Query 492 CSSVNRETHIHQFSISKDGLELKNVTKLLGNIITCNRSGNKLVIQTTDGKLFNYNTKSLV 551

CSSV RETHIHQFS+S+DGLEL+NVTKL GNIITCNR KLVIQTT+GKLFNYNTKSLV

Sbjct 492 CSSVKRETHIHQFSLSQDGLELRNVTKLEGNIITCNRRAEKLVIQTTNGKLFNYNTKSLV 551

Query 552 VTESFSLIEPCYDIKVHPSGLFCLSELSRLYLNNVPIDFPSITCKVTSFILKDPYLLVTT 611

V E+ SLIEPCYDIKVH SGLFCLSE SRLYLN+ PI+ PSITCKVTSFILKDPYLLVTT

Sbjct 552 VVETLSLIEPCYDIKVHSSGLFCLSEFSRLYLNDTPINLPSITCKVTSFILKDPYLLVTT 611

Query 612 SSHKLVILQCCDSENVEISDRKLERGCRLVTTFDNSVVLQAPRGNLETIQPRALTILTLG 671

SSHKLVILQC DSENVEIS+RKLERG RLVTTFDNSVVLQAPRGNLETIQPRALTILTLG

Sbjct 612 SSHKLVILQCSDSENVEISERKLERGSRLVTTFDNSVVLQAPRGNLETIQPRALTILTLG 671

Query 672 SLIDSKQFKSALSLMRKQRIDLNLIIDHNQKLFLDEVNNFVNQIDPQWITLLITELSGED 731

+LIDSKQFK+A SLMRKQRIDLNLIIDHNQKLFL+EVNN VNQIDPQWITLLITELS ED

Sbjct 672 TLIDSKQFKTAFSLMRKQRIDLNLIIDHNQKLFLNEVNNVVNQIDPQWITLLITELSAED 731

Query 732 VTANLYKQYYKKKDSSFLNGSKVKSVCEALLSVMSIEDLKKKYMFPILSALVKLGEMSKA 791

VT NLYKQYYKKKD+SFLNGSKVKSVCEALLS +S+EDLKKKY+FPILSALVK+GEMSKA

Sbjct 732 VTTNLYKQYYKKKDTSFLNGSKVKSVCEALLSALSVEDLKKKYIFPILSALVKIGEMSKA 791

Query 792 IQLASNEVAMQHLMFIVDSNKLYMEALGVYNLNAALKIAEKSQKDPKEYIPYLNSLKSME 851

IQLASNEVAMQHLMFIVDSNKLYMEALGVY+LNAALKIAEKSQKDPKEYIPYLNSLKS+E

Sbjct 792 IQLASNEVAMQHLMFIVDSNKLYMEALGVYDLNAALKIAEKSQKDPKEYIPYLNSLKSLE 851

Query 852 FNYMRFVIDKKLKRYESALTHISKCCDEHEEECLKFIEDNSLYPGALKIFKNDLKRSSII 911

NYMRF IDKKLKR ESAL HISKCCD+HEEECLKFIE+N+LYP AL IFKNDLKR SII

Sbjct 852 SNYMRFTIDKKLKRNESALNHISKCCDDHEEECLKFIEENALYPAALNIFKNDLKRYSII 911

Query 912 ASKYANYLFKQRHYEESGIMYLRSDDTEKALQSFTRAGNWRQCMIIVMDSNFSNEEQRKY 971

A KYA+YLFKQRHYEESGIMYLRSDD EKALQSFTRAGNWRQCMIIVMDSN+S+EEQRK+

Sbjct 912 AFKYADYLFKQRHYEESGIMYLRSDDNEKALQSFTRAGNWRQCMIIVMDSNYSDEEQRKH 971

Query 972 AESVCNMLISAHKYSEAAILYTEWLLDTDKAVEMYVQARCWKEAIYTAKKFNKSSLIDTI 1031

AE+VCNMLISAHKYSEAAILYTEWLLD DKAVEMY QARCWKEAIYTAKKF K SLID+I

Sbjct 972 AENVCNMLISAHKYSEAAILYTEWLLDADKAVEMYTQARCWKEAIYTAKKFKKPSLIDSI 1031

Query 1032 VKDAVIETANILAEDIHSQSDKTKKYVERLKNVRIEKENRATYIFDDMSDSASDTSSTSS 1091

+KDA+IETANIL EDIHSQS+KTKKYVERLK VRIEKENRA YIFDDMSD+ASDTSSTSS

Sbjct 1032 IKDALIETANILVEDIHSQSEKTKKYVERLKTVRIEKENRAAYIFDDMSDTASDTSSTSS 1091

Query 1092 VRTKSFQSRSSKNTKKMSRKMWSLKEGNPREEEALVATLSSSISSTEKYVGEVNSACSTL 1151

VRTKSFQSRSSKNTKKMSRKMWSLKEGNPREEEALVATLSS ISSTEKYVGE+NSAC TL

Sbjct 1092 VRTKSFQSRSSKNTKKMSRKMWSLKEGNPREEEALVATLSSIISSTEKYVGEINSACCTL 1151

Query 1152 LVFREDQLAAKLQSALAEWLSIIDKSKNVIWPKEAGTNEASVLDEKFKYPPEVNIKQDWK 1211

LVFRED LAAKLQSAL EWLSIID SKN+IWPKE GTNEASVLDEKFK+PPEV+IKQDWK

Sbjct 1152 LVFREDPLAAKLQSALEEWLSIIDNSKNIIWPKEPGTNEASVLDEKFKFPPEVSIKQDWK 1211

Query 1212 FNFLMS 1217

FN L S

Sbjct 1212 FNLLKS 1217

GLD-1 homolog

>TRINITY_DN6361_c0_g2_i1 len=2042 path=[0:0-2041]

CCAATACTTCAACATAGTAGCAGTTTTGCTCATGAAAGAGAAAAGCTACAATAGAACTTAGCTATCCATTT
ACATTGGCGGATTGCGTTGAGATTCAAAACCATGCAGCCTCCAAACATTGTCAACTTTTTATAATACTCAT
GGCAAGTGTTGCGTACTACAATTATCCTAACATCGACCCAAACCTTCGTGCTATAAATGTACCAACGCCAC
AGCTATCAGAACAATATGACTTCATCATTGTAGGTGCTGGAAGTGCAGGCTGTGTACTGGCTAATAGGCTG
ACGGAAATTGCCAATTGGACTGTACTATTGTTAGAAGTTGGCGGTGAAGAAACTATTGTAAGTGATACTCC
ATTGATGGCAGAAACTTTATGGACCTCACCCTTAAATTGGAATTATACAACAACAAGACAGACTAGGGCGT
GTTTGGCAACTGATGGGATTTGTCCTTGGCCAGGAGGAAGAGTAATAGGAGGAAGCAGCACGATCAATTA
CATGGTGTACGCAAGAGGCAACAGAAGAGATTATGACGGATGGGCAGCTCTAGGAAATCCTGGCTGGTCT
TATAATGAAGTTCTGCCATACTTCCGACTATCAGAGGACAACAGGAATCCAGTATATGCAAATGACACAAC
TTACCATTCTACCGGAGGTTATCAGACAGTCACAGACCTGAATTATCAGACACCTCTTGTCAGTGGATACCT
AGCGGCTGGAGAAGAGTTAGGTTTTAAAGTTCGAGACCTAAATGCAGAATACCAAACCGGTTTTATGCCCG
TACAAGGAACATTAAGAAATGGTTCCAGGTGTTCGACCGGAAAAGCATTTCTAAGACCAGTAAGGAACAG
AACTAACCTTAATGTTGCAGAAGGATCATTTGTGTCTAAAATAAACTTCATCAACAACAGGGCCGTAGGTG
TAACATTTATAAGGAACAACCAACAAATAAATGTCACAGCAACAAAGGAAGTCATAATATCAGCAGGGTC
GATCAGATCACCACACCTATTGATGCTTTCGGGTATCGGTCCAGCTGATGCATTAAACGGATTTGGAATTCC
TGTGATTCAAAATTTGAGCGTTGGCTACAATCTTCAAAATCATATAGGAGCCCCATTTTATTATCAAACCAA
TCCTCCAGTAGCAATTACTGCCTCGTCTTATGAAAATGTAGAAGCTATAGTGGAATATTCGCAGCCTAATGC
AGCTGGCCCACTCACATCTCCGGTCGGTATAGAAACAATTGCTTTCCTTAATTCTACTTTTTCAAATTCATCT
ATTGATTATCCAGACATTCAAATTCATTTCACTTCTTATGTGGATAATTTAGAAAACAATAACAGCATCTGG
TTTGGTGTCGGGCTGGCGATCCACCCACGAAGCAGGGGTAGGATAACATTACAATCAATTGACCCGTATCA
ATATCCTTTGATAGACCCCAATTACTTCAGTGAACCCGAGGACATGCAAGTACTAATGCAATCTCTAAAAT
ATGTGTCATCTGTAGCAAATTCGACAGCAATGCAAATATATAATAATGTTTTTCAAGAGCAATTATTCACCC
TTTGTAATAATTATACTATTTACAGTGATGAATTTTATAACTGTGTAATAAAAACTTACACAACAATTATTT
ATCACCCTGTTGGTACCTGCAAAATGGGACCGAGTACAGATACAGAAGCTGTTGTCAATTCTAATCTCCAA
GTATACGGCGTTGAGAATTTAAGAATCATTGATGCTTCCATCATGCCATTTGTACCCGGAGGAAATACAAA
TGCTCCAACAATAATGATTGCTGAACATGGGGCTGATATTATAAAAGCACATTATAACCAGCCAACACATA
TCCTATAGATAACAAATTAATTGAACCACTAAAATCACTATCACTGAATCATTATACATCTGCACTGGCACT
ATGGATATTCAACATATCTTAGAAGAGAATATTTTGTTAGATTAATATATTCAATATCTATTATATTGTTAA
ACATTATATGTTTGAATAAAGGATATAATTAGAATTAGAAAAAAA

Protein

RF: +1

ORF: 103 -> 1860

Length: 585 aa

>lcl|ORF1_TRINITY_DN6361_c0_g2_i1:102:1859 unnamed protein product

MQPPNIVNFFIILMASVAYYNYPNIDPNLRAINVPTPQLSEQYDFIIVGAGSAGCVLANRLTEIANWTVLLLEVGG
EETIVSDTPLMAETLWTSPLNWNYTTTRQTRACLATDGICPWPGGRVIGGSSTINYMVYARGNRRDYDGWAAL
GNPGWSYNEVLPYFRLSEDNRNPVYANDTTYHSTGGYQTVTDLNYQTPLVSGYLAAGEELGFKVRDLNAEYQ
TGFMPVQGTLRNGSRCSTGKAFLRPVRNRTNLNVAEGSFVSKINFINNRAVGVTFIRNNQQINVTATKEVIISAGS
IRSPHLLMLSGIGPADALNGFGIPVIQNLSVGYNLQNHIGAPFYYQTNPPVAITASSYENVEAIVEYSQPNAAGPL
TSPVGIETIAFLNSTFSNSSIDYPDIQIHFTSYVDNLENNNSIWFGVGLAIHPRSRGRITLQSIDPYQYPLIDPNYFSEP
EDMQVLMQSLKYVSSVANSTAMQIYNNVFQEQLFTLCNNYTIYSDEFYNCVIKTYTTIIYHPVGTCKMGPSTDT
EAVVNSNLQVYGVENLRIIDASIMPFVPGGNTNAPTIMIAEHGADIIKAHYNQPTHIL

Conserved Domains

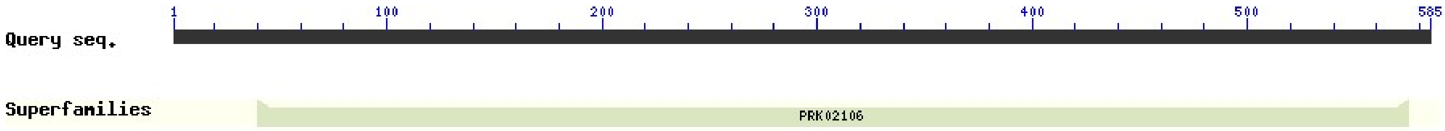

BLASTp

XP_014290348.1 glucose dehydrogenase [FAD, quinone] isoform X1 [*Halyomorpha halys*

Score:1058 bits

E-value: 0.0

Query 1 MQPPNIVNFFIILMASVAYYNYPSIDPNLRAINVPTPELSQQYDFIIVGAGSAGCVLANR 60

MQPPNIVNFFIILMASVAYYNYP+IDPNLRAINVPTP+LS+QYDFIIVGAGSAGCVLANR

Sbjct 1 MQPPNIVNFFIILMASVAYYNYPNIDPNLRAINVPTPQLSEQYDFIIVGAGSAGCVLANR 60

Query 61 LTEISNWTVLLLEVGGEETIISDTPLMAVTLWNSSLNWNYTTTRQARACLVTDGICPWPR 120

LTEI+NWTVLLLEVGGEETI+SDTPLMA TLW S LNWNYTTTRQ RACL TDGICPWP

Sbjct 61 LTEIANWTVLLLEVGGEETIVSDTPLMAETLWTSPLNWNYTTTRQTRACLATDGICPWPG 120

Query 121 GRVIGGSSTLNFMAYVRGNRRDYDGWAALGNPGWSYEEVLPYFLLSEDNRNPLYAMDTIY 180

GRVIGGSST+N+M Y RGNRRDYDGWAALGNPGWSY EVLPYF LSEDNRNP+YA DT Y

Sbjct 121 GRVIGGSSTINYMVYARGNRRDYDGWAALGNPGWSYNEVLPYFRLSEDNRNPVYANDTTY 180

Query 181 HSTGGYQTVSDLSYQTSLVSGYLAAGEEIGFQIRDINAEYQTGFMPVQGTLRNGSRCSTG 240

HSTGGYQTV+DL+YQT LVSGYLAAGEE+GF++RD+NAEYQTGFMPVQGTLRNGSRCSTG

Sbjct 181 HSTGGYQTVTDLNYQTPLVSGYLAAGEELGFKVRDLNAEYQTGFMPVQGTLRNGSRCSTG 240

Query 241 KAFLRPVRNRTNLYVAEGSFVTKINFMNNKAVGVTFVRNNQQINVTAKKEVIISAGSINS 300

KAFLRPVRNRTNL VAEGSFV+KINF+NN+AVGVTF+RNNQQINVTA KEVIISAGSI S

Sbjct 241 KAFLRPVRNRTNLNVAEGSFVSKINFINNRAVGVTFIRNNQQINVTATKEVIISAGSIRS 300

Query 301 AQLLMLSGVGPANELNRFGIPVIKNLSVGYNLQDHVGAPLFYKTNPSVAITASSYENIEA 360

LLMLSG+GPA+ LN FGIPVI+NLSVGYNLQ+H+GAP +Y+TNP VAITASSYEN+EA

Sbjct 301 PHLLMLSGIGPADALNGFGIPVIQNLSVGYNLQNHIGAPFYYQTNPPVAITASSYENVEA 360

Query 361 ILEYSQPNATGPLTSPVGIETIAFLNSTLANSSIDYPDIEIHFTSYVSYLENNDSIWFGV 420

I+EYSQPNA GPLTSPVGIETIAFLNST +NSSIDYPDI+IHFTSYV LENN+SIWFGV

Sbjct 361 IVEYSQPNAAGPLTSPVGIETIAFLNSTFSNSSIDYPDIQIHFTSYVDNLENNNSIWFGV 420

Query 421 GLVIHPQSSGRITLQSTDPYQHPLIDPNYFSEPQDLQTLMESLKYVSLIANSTAMQKYNS 480

GL IHP+S GRITLQS DPYQ+PLIDPNYFSEP+D+Q LM+SLKYVS +ANSTAMQ YN+

Sbjct 421 GLAIHPRSRGRITLQSIDPYQYPLIDPNYFSEPEDMQVLMQSLKYVSSVANSTAMQIYNN 480

Query 481 VFQDQFFTLCNNYTTYSDEFYNCVIKTYTTTIFHPVGTCKMGPSTDTEAVVNPNLQVHGI 540

VFQ+Q FTLCNNYT YSDEFYNCVIKTYTT I+HPVGTCKMGPSTDTEAVVN NLQV+G+

Sbjct 481 VFQEQLFTLCNNYTIYSDEFYNCVIKTYTTIIYHPVGTCKMGPSTDTEAVVNSNLQVYGV 540

Query 541 ENLRVIDASIMPFVTGGNTNAPVIMIAEHGADIIKAYYNQPTQI 584

ENLR+IDASIMPFV GGNTNAP IMIAEHGADIIKA+YNQPT I

Sbjct 541 ENLRIIDASIMPFVPGGNTNAPTIMIAEHGADIIKAHYNQPTHI 584

ACO-1 homolog

>TRINITY_DN320_c0_g1_i9 len=3642 path=[1:0-3232 17:3233-3254 19:3255-3286 20:3287-3290 23:3291-3641]

GTGTGATAGTAAATTATTACCTTCTCCAGTCTTATCAAGCTTTCTACTGTTGGAATTTATCGTTCGCGGTCTA
GTATTTTAGTGAATATTTTTCTGCCATGTCAGGTCCAAACCCATACAACAAAATTCTGAAAAAATTGGAGGT

TGCTGGCGTGTCTTATAATTATTACAATCTTCCTGAGCTAGGTCCTCAATATGGAAAACTTCCATTTTCAATC
AGAGTGCTTCTGGAATCAGCAGTCAGAAACTGCGACAACTTTGAGGTTAAGCAAACTGATGTGGAAAACAT
TTTGAATTGGGAAAAGACTGGAAAAGATGACAGTAATGTTGAAGTGGCTTTCAAACCAGCCAGAGTTTTGT
TACAGGATCTGACAGGCGTGCCAGCGGTTGTTGACTTTGCTGCAATGCGTGATGCCGTCAAAACACTTGGA
GGCAATCCAGATAAAATTAATCCCATTTGTCCCTCTGATCTTGTTATAGATCATTCTGTTCAGGTGGATTTTG
CTCGTATTGACAATGCTCTACAGAAAAATGAAGAAATTGAATTTGAAAGAAATAAGGAGAGATTTACATTT
CTTAAGTGGGGTGCTAAAGCATTCCGAAACATGCTTATCGTTCCTCCAGGCTCAGGAATCGTCCATCAGGT
GAATCTCGAATATTTAGCAAGAGTTGTCTTCACTGATAAACTACTTTATCCTGACAGTGTGGTCGGAACCGA
CTCTCATACGACCATGATCAATGGATTGGGTGTTGTAGGATGGGGAGTGGGAGGAATTGAGGCAGAAGCTG
TTATGCTTGGCCAAGCTATAAGTATGCTGATCCCTCAGGTTATTGGGTACAAGCTCGTTGGAAAACTCAACC
AATACGCAACATCTACTGATCTCGTCCTAACCATCACCAAGAATTTGAGGCAGTTGGGAGTTGTGGGCAAG
TTTGTTGAGTTCTTCGGCCCTGGAGTTGTTGAGCTGTCTATCGCCGATAGGGCTACTATCTCCAACATGTGC
CCTGAATATGGTGCTACTGTTGGATTTTTCCCAGTCGATGAAAACAGTCTTTATTATCTTTCTCAGACAAATC
GAGATCCGGCCAAAATAGATGCTATCAGGAAGTACCTTCAGTCAGTGGGAATGTTGAGAGATTATTCTAAC
GCTTCGCAAGACCCTGTCTATTCCCAGGTCGTGGAACTTGACTTGTCAACAGTGGTCAGTTGCATTTCTGGA
CCAAAGAGGCCTCAGGACAGGGCCTCGGTGACTGAAGTCAAGTCACAATTTTTAGCAGCTCTAACTCATAA
AGTTGGTTTCAACGGATACGGCCTGAAACAAGAAGCCGTGGGTGCGAGCGGCAACTTCACCCATGAAGGG
AAGGAATACACGCTGAGGCACGGCTCTGTCGTGATAGCTGCTATCACATCATGTACCAACACCTCCAACCC
TTCTGTCATGTTGGGAGCTGGTCTTTTGGCAAAGAAGGCAGTTGAAGCAGGCCTCTCTGTGTTACCTTACAT
AAAGACGTCTCTATCTCCTGGTTCCGGAGTGGTGACTCATTACCTCCGCGAGTCAGGTGTTATACCTTACCT
GGAAAAGCTTGGCTTCTCCATCGTTGGCTATGGATGTATGACCTGTATTGGAAATTCTGGTCCTTTGCCAGA
TGTTATGATAGAAGCCATTGAAAAGAATGAACTTGTCTGCTGTGGTGTGCTGAGTGGAAACAGAAATTTTG
AAGGACGTATCCATCCAAACACTAGGGCTAACTATTTGGCCTCACCTCTGCTTGTTGTAGCCTACGCAATCG
CTGGTAGAATTGATATAGACTTTGAAACTGAACCTCTTGGCAAAAGACCTGATGGAAGCAATGTTTTCTTG
AGAGATATCTGGCCCCTGCGTACAGAAATCCAGGAAGTAGAAAAGAAGACAGTAATACCTGCCATGTTTA
GGGATGTCTACGCAAGAATAGAGAATGGTTCAAACAGCTGGAGGTCCTTAAACGCTCCCGATGACCAGCTC
TATCCGTGGGACCCTTCATCCACTTACATCAGGAGGCCTCCATTTTTTGATGGAATGACAAGAGACTTACCC
GAGATTAAGAAGATCAAAAATGCCAAAGTTCTGCTTTTCCTTGGTGATTCAGTAACGACAGATCACATTTC
GCCAGCTGGTAGTATCTCCAGGAAGAGCCCTGCTGCTCGTTACCTTGCTTCTAAAGGACTTACACCTCGTGA
ATTCAATTCTTACGGTTCAAGAAGAGGAAATGATGAAATCATGGCTCGTGGAACGTTTGCAAACATTAGGC
TTGTCAATAAATTCCTCAATCACGCTGGCCCTCAGACAATTCACTTCCCATCAGGAGAAGAGATGGACGTG
TTCGATGCTGCCGAAAGATATAAGAGTGAAGGAGTACCACTGATCGCCATCGTTGGAAAAGATTACGGTTC
TGGTTCATCAAGGGATTGGGCTGCTAAGGGGCCATTGCTTCTTGGTATTAAAGCTGTTATTGCTGAATCGTA
TGAACGTATCCATCGCTCCAACTTAGTCGGAATGGGAATTGTTCCTCTGCAGTTCCTTGAAGGACAGACAG
CTGACTCTCTTGGCCTCACTGGAAAGGAAAACTACACCATCAACTTGCCAACTGACCCCAGGCCACACCAG
AAAATCACTGTTCAGACTGATGATGGAAAGTCTTTCGAAGTGCTCGTGAGGTTTGATACTGAAGTTGACCTC
CTCTACTTCAAGCACGGAGGAATCCTCAACTGCATGGTCAGGAGAATGCTTAGCTAAATTTATTTATGTGA
AATATAATACAATGTTTTTCATGACAATAAATTTTGTTGGAAAAATAATATGTAGTGCATAATGCATAATGA
TTAATACGGTCACGGTCTGATGTTTTTTGTATTGTATTATACTAGATATATATTTTTATATTTGTATGATCTG
AATATAAAAGGGATTGTTAATTGTTATTATTATTCAGGTTTTATTTTTTTCTTAAATATTAATGATATTTGGT
TATTTGATATTACCTCAATATGGATAGTCATTATACCATTCAATCTACTCGATGTTATAATGAAATTATTTTT
TTCAATAAAATAAAGGATTTAAAATTTATTTTTTATTTAAAATATAAGTTAACGCTTAGCTTCCTTATTTTGA
TATGATGTAGAATACATTAGAACAAAAAAATGTTTTTGAAGGATGAGAGGGGTGGTGGTAAAAAAAAAAT
CTGGTGCGGGTAACACACGACTTTCACTTACCTCCATTTTTAAAATCAATGAAAGAATTTTCAAATACACTT
AACGATAAAAATATGTGAAACTATTAAAGAATTAAGTAAATTGAATTTATATAGAAAGATGTGTTTTATTA
AAAACTCTTTTACAAGCTCTTGTGTATTAGATAATTTTTTTTTTCTAAATTCTTTAGCTCTCTAACGGTATATT
ATAGGTGTATATTACTGTTGCTATGTTCAAAATAAAAAAAATGCATTAAAAAAATATTTTTCAGTTTTCATC
TTTAGTTGTTTTTTAAATTCGCACAACTTACATACTAAGCTGTTATTTATCAATGCTAACAAGATAATTTGGT
TATTTTTATAGAATCAACAAAATTAAAGATGATATAAAAAATATCGAAAAAAGGTTGG

Protein

RF: +3

ORF: 99 -> 2774

Length: 891 a.a.

>lcl|ORF7_TRINITY_DN320_c0_g1_i9:98:2773 unnamed protein product

MSGPNPYNKILKKLEVAGVSYNYYNLPELGPQYGKLPFSIRVLLESAVRNCDNFEVKQTDVENILNWEKTGKD
DSNVEVAFKPARVLLQDLTGVPAVVDFAAMRDAVKTLGGNPDKINPICPSDLVIDHSVQVDFARIDNALQKNEE
IEFERNKERFTFLKWGAKAFRNMLIVPPGSGIVHQVNLEYLARVVFTDKLLYPDSVVGTDSHTTMINGLGVVGW
GVGGIEAEAVMLGQAISMLIPQVIGYKLVGKLNQYATSTDLVLTITKNLRQLGVVGKFVEFFGPGVVELSIADRA
TISNMCPEYGATVGFFPVDENSLYYLSQTNRDPAKIDAIRKYLQSVGMLRDYSNASQDPVYSQVVELDLSTVVS
CISGPKRPQDRASVTEVKSQFLAALTHKVGFNGYGLKQEAVGASGNFTHEGKEYTLRHGSVVIAAITSCTNTSN
PSVMLGAGLLAKKAVEAGLSVLPYIKTSLSPGSGVVTHYLRESGVIPYLEKLGFSIVGYGCMTCIGNSGPLPDVM
IEAIEKNELVCCGVLSGNRNFEGRIHPNTRANYLASPLLVVAYAIAGRIDIDFETEPLGKRPDGSNVFLRDIWPLR
TEIQEVEKKTVIPAMFRDVYARIENGSNSWRSLNAPDDQLYPWDPSSTYIRRPPFFDGMTRDLPEIKKIKNAKVL
LFLGDSVTTDHISPAGSISRKSPAARYLASKGLTPREFNSYGSRRGNDEIMARGTFANIRLVNKFLNHAGPQTIHF
PSGEEMDVFDAAERYKSEGVPLIAIVGKDYGSGSSRDWAAKGPLLLGIKAVIAESYERIHRSNLVGMGIVPLQFL
EGQTADSLGLTGKENYTINLPTDPRPHQKITVQTDDGKSFEVLVRFDTEVDLLYFKHGGILNCMVRRMLS

Conserved Domains

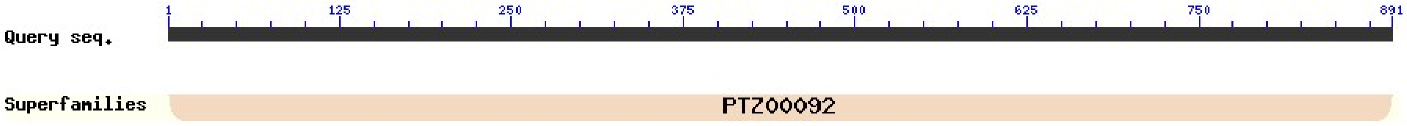

BLASTp

XP_014275296.1 cytoplasmic aconitate hydratase-like [*Halyomorpha halys*]

Score:1709 bits

E-value: 0.0

Query 5 GPNPYDKILKKLD-SGVSYNYYNLPELGPQYEKLPFSIRVLLESAVRNCDNFEVKEADVQ 63

GPNPY+KILKKL+ +GVSYNYYNLPELGPQY KLPFSIRVLLESAVRNCDNFEVK+ DV+

Sbjct 3 GPNPYNKILKKLEVAGVSYNYYNLPELGPQYGKLPFSIRVLLESAVRNCDNFEVKQTDVE 62

Query 64 NILNWEKTGKDSSSVEVYFKPARVLLQDLTGVPAVVDFAAMRDAVKSLGGNPDKINPICP 123

NILNWEKTGKD S+VEV FKPARVLLQDLTGVPAVVDFAAMRDAVK+LGGNPDKINPICP

Sbjct 63 NILNWEKTGKDDSNVEVAFKPARVLLQDLTGVPAVVDFAAMRDAVKTLGGNPDKINPICP 122

Query 124 SDLVIDHSVQVDFARVDNALQKNEEIEFERNKERFTFLKWGAKAFRNMLIVPPGSGIVHQ 183

SDLVIDHSVQVDFAR+DNALQKNEEIEFERNKERFTFLKWGAKAFRNMLIVPPGSGIVHQ

Sbjct 123 SDLVIDHSVQVDFARIDNALQKNEEIEFERNKERFTFLKWGAKAFRNMLIVPPGSGIVHQ 182

Query 184 VNLEYLARVVFTDKLLYPDSVVGTDSHTTMINGLGVVGWGVGGIEAEAVMLGQAISMLIP 243

VNLEYLARVVFTDKLLYPDSVVGTDSHTTMINGLGVVGWGVGGIEAEAVMLGQAISMLIP

Sbjct 183 VNLEYLARVVFTDKLLYPDSVVGTDSHTTMINGLGVVGWGVGGIEAEAVMLGQAISMLIP 242

Query 244 QVIGYKLVGKLNQYATSTDLVLTITKHLRQLGVVGKFVEFFGPGVVELSIADRATISNMC 303

QVIGYKLVGKLNQYATSTDLVLTITK+LRQLGVVGKFVEFFGPGVVELSIADRATISNMC

Sbjct 243 QVIGYKLVGKLNQYATSTDLVLTITKNLRQLGVVGKFVEFFGPGVVELSIADRATISNMC 302

Query 304 PEYGATIGFFPVDENSLYYLSQTNRDEAKIDAIRKYLKAVGMLRDYANAAQDPVFSQVVE 363

PEYGAT+GFFPVDENSLYYLSQTNRD AKIDAIRKYL++VGMLRDY+NA+QDPV+SQVVE

Sbjct 303 PEYGATVGFFPVDENSLYYLSQTNRDPAKIDAIRKYLQSVGMLRDYSNASQDPVYSQVVE 362

Query 364 LDLATVVSSISGPKRPQDRAAVTEVKSQFLSALTHKVGFNGYGLKSEAVNATGSFSYEGK 423

LDL+TVVS ISGPKRPQDRA+VTEVKSQFL+ALTHKVGFNGYGLK EAV A+G+F++EGK

Sbjct 363 LDLSTVVSCISGPKRPQDRASVTEVKSQFLAALTHKVGFNGYGLKQEAVGASGNFTHEGK 422

Query 424 EYTLKHGSVVIAAITSCTNTSNPSVMLGAGLLAKKAVEAGLSVLPYIKTSLSPGSGVVTH 483

EYTL+HGSVVIAAITSCTNTSNPSVMLGAGLLAKKAVEAGLSVLPYIKTSLSPGSGVVTH

Sbjct 423 EYTLRHGSVVIAAITSCTNTSNPSVMLGAGLLAKKAVEAGLSVLPYIKTSLSPGSGVVTH 482

Query 484 YLQESGVIPYLEKLGFSIVGYGCMTCIGNSGPLPDNMIEAIEKNELVCCGVLSGNRNFEG 543

YL+ESGVIPYLEKLGFSIVGYGCMTCIGNSGPLPD MIEAIEKNELVCCGVLSGNRNFEG

Sbjct 483 YLRESGVIPYLEKLGFSIVGYGCMTCIGNSGPLPDVMIEAIEKNELVCCGVLSGNRNFEG 542

Query 544 RIHPNTRANYLASPLLVVAYAIAGRIDIDFETEPLGKKADGSNVFLRDIWPLRSEIQEVE 603

RIHPNTRANYLASPLLVVAYAIAGRIDIDFETEPLGK+ DGSNVFLRDIWPLR+EIQEVE

Sbjct 543 RIHPNTRANYLASPLLVVAYAIAGRIDIDFETEPLGKRPDGSNVFLRDIWPLRTEIQEVE 602

Query 604 KKTVIPAMFRDVYARIENGSNNWRSLNAPSDQLYPWDSKSTYIKKPPFFDGMTRDLPEIK 663

KKTVIPAMFRDVYARIENGSN+WRSLNAP DQLYPWD STYI++PPFFDGMTRDLPEIK

Sbjct 603 KKTVIPAMFRDVYARIENGSNSWRSLNAPDDQLYPWDPSSTYIRRPPFFDGMTRDLPEIK 662

Query 664 KITNAKVLLFLGDSVTTDHISPAGSISRKSPAARYLASKGLTPREFNSYGARRGNDEIMA 723

KI NAKVLLFLGDSVTTDHISPAGSISRKSPAARYLASKGLTPREFNSYG+RRGNDEIMA

Sbjct 663 KIKNAKVLLFLGDSVTTDHISPAGSISRKSPAARYLASKGLTPREFNSYGSRRGNDEIMA 722

Query 724 RGTFANIRLVNKFLNHAGPQTLHFPSGEEMDVFDAAERYKSEGVPLIAIVGKDYGSGSSR 783

RGTFANIRLVNKFLNHAGPQT+HFPSGEEMDVFDAAERYKSEGVPLIAIVGKDYGSGSSR

Sbjct 723 RGTFANIRLVNKFLNHAGPQTIHFPSGEEMDVFDAAERYKSEGVPLIAIVGKDYGSGSSR 782

Query 784 DWAAKGPLLLGIKAVIAESYERIHRSNLVGMGIVPLQFLEGQTADSLGLTGKENYTINLP 843

DWAAKGPLLLGIKAVIAESYERIHRSNLVGMGIVPLQFLEGQTADSLGLTGKENYTINLP

Sbjct 783 DWAAKGPLLLGIKAVIAESYERIHRSNLVGMGIVPLQFLEGQTADSLGLTGKENYTINLP 842

Query 844 ADPRPHQKINVQTCNGKSFEVIVRFDTEVDILYFKHGGILNCMVRRML 891

DPRPHQKI VQT +GKSFEV+VRFDTEVD+LYFKHGGILNCMVRRML

Sbjct 843 TDPRPHQKITVQTDDGKSFEVLVRFDTEVDLLYFKHGGILNCMVRRML 890

Vasa intronic gene (VIG)

>TRINITY_DN5670_c0_g1_i1 len=1531 path=[0:0-772 2:773-1530]

CCTTAGTCATGATTTCAAATTTTTAGCTATCAGAAACGATAATAGATATTACAGGGGGCGCTGATGGTTAAC
CTCGTGGGATTTTTAGATTGTCGCCATTTTGCTCTGTAGCACATTTCAAATCTATTAGACGAAGTTTTCAAAC
TTATTATTCTTACTGTTCCGAATTTAACCATGTCTACAACACAGTACGGAATCGGTGTCACTAAGAACCGAT
TCGAGTTATTCGACATTGATGATGAGGATCCATTAGAAGTTTTGAAAATACGTGAACTGGAAAGGGAGGCC
CGAAAGAAAACCAAGCTTTCTGAAAAAGAAAATAAAGGGAAAGAACTTGCACCAAAACCAAAGGTTAGC
ATTCAAAGGAAAGGTATCAAGGAAACTCAAAATCTTAAACCCTTGGAAGGACAAAAACCAAAGGAGGAAG
TGAAAGGTAGACCTGCAAGAATTGAGAGACCAGAACGTAAATTTACCGGAACTGATCCTAGAGAAGTTCA
AAATAATCGCCGTAATCGTGTGGAGGATCGTCCATCTACTGATTTTCAGCCTAGGGAAGAAAGAGGTGGTG
AAAGGTTTGATCGACGTGAATATCGATCAGATAGGACCACTAGTGGTTTTTATGGAGAAGGTGGTGATGGA
AGAGGCCGAGGTAGGAGTGGACCCCAGCGTGGAAATTTCATTCGTGGTGGTAGAGGTGGAAGAGGAAGTC
AACGTCCAACATTTGATGTTAGGGGAAAACGGGAATATGATCGTCAATCCGGCTCTGATAAAACTGGTGTG
AAACATGTAGACAAGAGGGAAGGTGCTGGCGCACACAACTGGGGCAACCTTCGCGATGATATTGTTGATA
TTCAAAATCCCCCTGTGTCTGATGAAGCTACATGGACTGTTGAAAAAACTGAAGAACCTGCTGTTGAAACA
AATGGAGCAATAGTTGAAACTGAGGAAGTTGCTCAGAACACTGCTGAAGAAGAACTTAAAGAATTGACTTT
AGATGAATGGAAAGCATTGAAAGCTCCGAGACAAAAACCTACCTACAACATTAGAAAGGCTGGTGAAGGA

GAAGACCCTACTCAATGGAAAAAAATGTATGCATTGCAGAAAAAGAAAGATGGTGAAGAAGAAGAAGAG
GATGATGAGTTTGAATATGAAAGTTTTGATTATCCCCAAAGAGTCGGACGTCAAAAGCATGTTTTGGATATT
GATATACATTTTAAAGATACTCGGGGTGGAGGTAGAGGTCGTGGTGGAAGAGGAATGGGTCGTGGAGGCC
CCCGTATGGGTTTAAGAGGAACAAATGGACCACCAGCTGAAAAAGTTCATCTTGCTCGTGATACTGTTATT
CCAAAAATTGCCCCAAAAGTTGATGATGAGCATGATTTTCCTTCTCTGGGTTGAATGACTAGTCTTGAATCA
ATAATTGTTAAATTCAATTAAAACTCCTTCTGAATTTAGACTGACATTTATTTATAGGGAAAAAAAAAAAA
AAAAAAAACCTTTCCTATTTCCATTGCAGGAGTCTAGGTTACTT

Protein

RF: +1

ORF: 175 -> 1398

Length: 407 aa

>lcl|ORF1_TRINITY_DN5670_c0_g1_i1:174:1397 unnamed protein product

MSTTQYGIGVTKNRFELFDIDDEDPLEVLKIRELEREARKKTKLSEKENKGKELAPKPKVSIQRKGIKETQNLKPL
EGQKPKEEVKGRPARIERPERKFTGTDPREVQNNRRNRVEDRPSTDFQPREERGGERFDRREYRSDRTTSGFYGE
GGDGRGRGRSGPQRGNFIRGGRGGRGSQRPTFDVRGKREYDRQSGSDKTGVKHVDKREGAGAHNWGNLRDDI
VDIQNPPVSDEATWTVEKTEEPAVETNGAIVETEEVAQNTAEEELKELTLDEWKALKAPRQKPTYNIRKAGEGE
DPTQWKKMYALQKKKDGEEEEEDDEFEYESFDYPQRVGRQKHVLDIDIHFKDTRGGGRGRGGRGMGRGGPR
MGLRGTNGPPAEKVHLARDTVIPKIAPKVDDEHDFPSLG

Conserved Domains

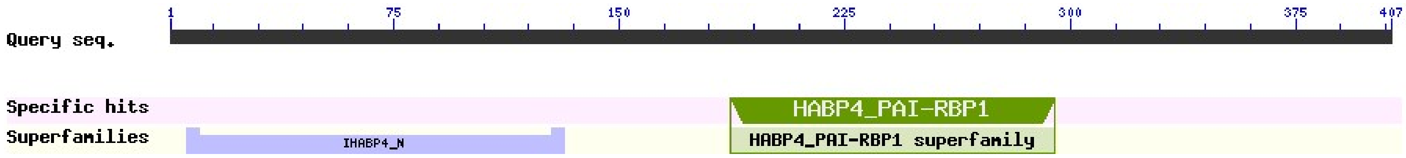

BLASTp

XP_014292052.1 plasminogen activator inhibitor 1 RNA-binding protein isoform X3 [*Halyomorpha halys*]

Score:782 bits

E-value: 0.

Query 1 MSTTQYGIGVTKNRFELFDIDDEDPLEVLKLREQEREARKKTKLSEKENKGKELAPKPKV 60

MSTTQYGIGVTKNRFELFDIDDEDPLEVLK+RE EREARKKTKLSEKENKGKELAPKPKV

Sbjct 1 MSTTQYGIGVTKNRFELFDIDDEDPLEVLKIRELEREARKKTKLSEKENKGKELAPKPKV 60

Query 61 SIQRKGIKETQNLKPLEGQKPKEAEVKGRPARIERPERKFTGTDPREVQNNRRNRVEDRA 120

SIQRKGIKETQNLKPLEGQKPKE EVKGRPARIERPERKFTGTDPREVQNNRRNRVEDR

Sbjct 61 SIQRKGIKETQNLKPLEGQKPKE-EVKGRPARIERPERKFTGTDPREVQNNRRNRVEDRP 119

Query 121 STDFQPREERGGERFDRREYRSDRTTSGFYGEGGDGRGRGRSGPQRGNFIRGGRGGRGSQ 180

STDFQPREERGGERFDRREYRSDRTTSGFYGEGGDGRGRGRSGPQRGNFIRGGRGGRGSQ

Sbjct 120 STDFQPREERGGERFDRREYRSDRTTSGFYGEGGDGRGRGRSGPQRGNFIRGGRGGRGSQ 179

Query 181 RPTFDVRGKREYDRQSGSDKTGVKHVDKREGAGAHNWGNLRDDIVDIQNAPVPDETTWTV 240

RPTFDVRGKREYDRQSGSDKTGVKHVDKREGAGAHNWGNLRDDIVDIQN PV DE TWTV

Sbjct 180 RPTFDVRGKREYDRQSGSDKTGVKHVDKREGAGAHNWGNLRDDIVDIQNPPVSDEATWTV 239

Query 241 EKTEEPAVETNGAIVETEEVLPNTAEEELKELTLDEWKALKAPRQKPTYNIRKAGEGEDP 300

EKTEEPAVETNGAIVETEEV NTAEEELKELTLDEWKALKAPRQKPTYNIRKAGEGEDP

Sbjct 240 EKTEEPAVETNGAIVETEEVAQNTAEEELKELTLDEWKALKAPRQKPTYNIRKAGEGEDP 299

Query 301 TQWKKMYALQKKKDGEEEEDDDEFEYESYEYPQRVGRQKHVLDIDIHFKDTRGGGRGRGG 360

TQWKKMYALQKKKDGEEEE+DDEFEYES++YPQRVGRQKHVLDIDIHFKDTRGGGRGRGG

Sbjct 300 TQWKKMYALQKKKDGEEEEEDDEFEYESFDYPQRVGRQKHVLDIDIHFKDTRGGGRGRGG 359

Query 361 RGMGRGGPRMGLRGTNGPPAEKVHIARDPVIPKIAPKVDDEHDFPSLG 408

RGMGRGGPRMGLRGTNGPPAEKVH+ARD VIPKIAPKVDDEHDFPSLG

Sbjct 360 RGMGRGGPRMGLRGTNGPPAEKVHLARDTVIPKIAPKVDDEHDFPSLG 407

Staufen

>TRINITY_DN4585_c0_g1_i5 len=2849 path=[1:0-70 5:71-279 6:280-280 7:281-2848]

GATGAACATTCTGATGACAAAACAAGAGTAGGAGTTTTGTGGTTAATTAAGAATAACAGAAGAAGACTTTA
TCTTGTAACTGGAAAGTTATCACCGATTTATTAGAACCTGATTCTGATAATATTTCTTGAGGAAATGAAGAT
ATTCCTTATCTTACACGAGATTGATACAATTTGTGAATTGAAGATCACACAGACTTGTCGGCACGCGGTTTG
GTTTTTACGCTAGAAGATGGTGGTGGTTCTCGGAAACTGGAAAAGACCCTCCTGGACTAGTTTAGGCCATTT
GAGTATGGCAGGAAGCGCGCCAGTGCCTGTAGGTACTGACAATGAGGGGTCCTCCGCGGGCGGCAGTCC
ACCCAGGATGAACCCGCCACGTCTGACGCCAGTCAATCCGTCTTAGGCAAAGAGAAGACTCCGATGTGCCT

CGTCAATGAGTTGGCGAGGTATAACAAGATAGAGCATCAGTACCGCCTGACAAGTGAAGATGGCCCACCG
CATAAAAAGAAGTTTACGGTAACGTTGAAGCTCGGTAATGAGGAGTACACTGCGGAGGGAGCCAGTATCA
AGAAGGCACAGCATGCGGCAGCTGGCATCGCTCTTAAGAGTACCACCTACAAGCATCCGCCTCCTAAATCT
TCGAGGCAGTCCCGAGGTTCAAAAAGTAATATCACCCCTACCGTAGAACTTAATGCTTTGGCGATGAAGAG
GGGAGAACCCACGGTTTATACCCTAGTCGAATCTCCCCAGTTTCATCAATACGCGTACCATCACTCCCACAG
GGGAATGTACACTCAGCCGTACAACGACCACTTCCTCCCCTATGGAAGAGGGAAGCTCAATTTCAGAGCCG
GTTACAATAGAATCGATCCAAGGTACTATGGGCCAGGAAATAAAGTGCCGCTGTACAAGGTGGTGGTTAA
AGTTGGCGAGCGTGAGTTCTCCGGGGAGGGCAACTCGGCGCAGGCCGCCCGCCACGACGCCGCCTCCAAG
GCGCTGCAACTGCTGCATAGTCTACCTGTCGACGACACCTGTCCTGCCAACTCTACCATCTGCGGTGGACTT
ACTGAACCGGAGTCTGAACTGAAATCACCGATATCGTTAGTTCACGAGATAGCATTAAAGAGGAATCTTCC
TGTTCTTTTTGAAGTTATCTCCGAAAAGGGTCAGCCTCATATGAAAACTTTCGTAACGAGGTGTTGTGTAGG
CGATAAGTTTGAAACGACCGGCGAAGGAAACGGAAAGAAGATTTCGAAGAAACGCGCTGCCGAGAAAAT
GCTTGAACAACTTCGAACCCTCCCACCGACAACAACTGTGACAGCGCCGGGTTGTGCTTCAGCGCTCGCTC
GTGCCAAGCGTAAGGCTACTAGTGGGAAAAAGAAGTCTAGGAATATCATCAAGGAAACTCCCGTCTCTCA
GGAAACCAAGTATGATGAGCGTGATAACAGCGATGAAATGAACCCCATCTCGCGTTTGATCCAAATCCAGC
AGGCTAAGAAGGAGCGTGAGCCGGTTTATACTTTAAGGGAAGAGCGAGGCCTTCCGAGGAGAAGAGAGTT
CATCATGGAAGTTACCGTTGGTAAGCACTCTTACATCGGATCTGGCCCTAACAAGAAGGTGGCCAAGCGCA
ACGCGGCCGAGGGCCTCCTCCAGCAGCTCGGCTACGCCAGCCCGACCCTCAGCAGGACTGGGCACCCGCTT
CCTCAGCTGCAGTCGCAGTCGCAGCCTTCGCACAAAGTAAGATTTAGCGATGAAAAACCAGAAGGCGGGG
TTTCAGGGCGACAGCTTGTACCCGGTCTAATCCTTATGAAGGACAACAAGGCCACTAATAATGGCCACAAG
CCTGGCACAGGTGTCAACTTGAAAGCAACCATTGCCAAAGAGTACCTGAGCGAAGGTTCTTCGCCGACGGC
TGATGCTCTGGCTGGAGTCAAACGCCAGACCAAAAACTCGTCAGTTAGGCCGACCGAACAGTTGGCATACT
TAGCTTCTGTATTAAATTTTCAAGTCCAGTTTTCGGATTTCCCTAAGGGCAATCATAGTGAATTCTTAACTCT
AGTCTCTCTCGACACGGATCCTCCCCAAGTCTGCCACGGAGCTGGAGCTACAATAGATCTCTCCCACGAGC
AAGCTGCTTTAAATGCTCTTAGATCTCTCTCAAAGGTCGGCCTCGATTCGATCACGCCCGCCAAGAAGGAG
GCCACCACCACGCCGCAGCCAGTTCCAAAACCTGAACCTGGGCTGTACATTGATCTTAAAGAGGAAACTCC
CTACGTCAACGGGAAGTGAAAAATAGGTTTTATTTTAATACTGTAAAAAACAAGAAAAAAAAAGCCAAAA
AAAAAAAAAAGATTCCTTTAGTTTTAAATGTTTATTTAACACAAAAATTTATTTAAATACATTATTAATTTA
ATCGAATGATAATCCAACAATAAATCAGAGAGATCTGACGTTAATATGTTTACCCTACTGTGAAATAATCTT
TGCTGGTCATATTTTTATTATTATTGTTATTTTGTTTTAGTTATGTTTTATTATTATTTTTTTTTTTTTGTTTGTA
ATATTTTGTAAATGAACGTGACTGATAAAAAGAAAGAAAACAAAAACATAAATACAGGCCACTTATAGCA
TTAGACGGTTTAAATCTGTTATAGGTTCTGATATGTTTATATAAATTATTAATTAAGAAGAATAGATGGAAA
AAAGTTTATTAGTAATAAAAACCGAGTGTTACAAGTGGGCCACGATTTAATTTTTTTTGTTTTGTTTTTTTAA
AAAAAATTAATTATTAAATGTGTAAAGTTTTGGCAGTTTTTGTCGGAACGGTCGCTATTTATATATTTCATC
GC

Protein

RF: +1

ORF: 232 ->2289

Length: 686 aa

>lcl|ORF2_TRINITY_DN4585_c0_g1_i5:231:2288 unnamed protein product

MVVVLGNWKRPSWTSLGHLSMAGSAPVPVGTDNEGSSAGGQSTQDEPATSDASQSVLGKEKTPMCLVNELAR
YNKIEHQYRLTSEDGPPHKKKFTVTLKLGNEEYTAEGASIKKAQHAAAGIALKSTTYKHPPPKSSRQSRGSKSNI
TPTVELNALAMKRGEPTVYTLVESPQFHQYAYHHSHRGMYTQPYNDHFLPYGRGKLNFRAGYN

RIDPRYYGPGNKVPLYKVVVKVGEREFSGEGNSAQAARHDAASKALQLLHSLPVDDTCPANSTICGGLTEPESE
LKSPISLVHEIALKRNLPVLFEVISEKGQPHMKTFVTRCCVGDKFETTGEGNGKKISKKRAAEKMLEQLRTLPPT
TTVTAPGCASALARAKRKATSGKKKSRNIIKETPVSQETKYDERDNSDEMNPISRLIQIQQAKKEREPVYTLREE
RGLPRRREFIMEVTVGKHSYIGSGPNKKVAKRNAAEGLLQQLGYASPTLSRTGHPLPQLQSQSQPSHKVRFSDE
KPEGGVSGRQLVPGLILMKDNKATNNGHKPGTGVNLKATIAKEYLSEGSSPTADALAGVKRQTKNSSVRPTEQ

LAYLASVLNFQVQFSDFPKGNHSEFLTLVSLDTDPPQVCHGAGATIDLSHEQAALNALRSLSKVGLDSITPAKKE
ATTTPQPVPKPEPGLYIDLKEETPYVNGK

Conserved Domains

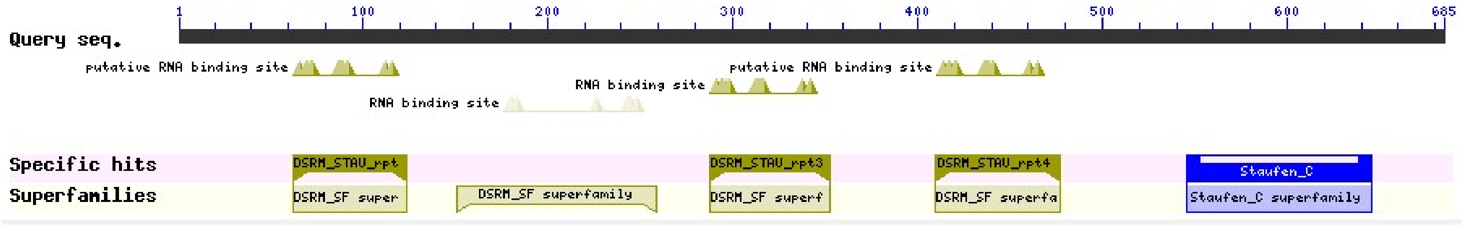

BLASTp

XP_014282526.1 PREDICTED: double-stranded RNA-binding protein Staufen homolog 2 isoform X5 [*Halyomorpha halys*]

Score:1323 bits

E-value: 0.0

Query 1 MAGSAPVPVGTDNEGSSAGGQSTQDEPATSDGSQSVLGKEKTPMCLVNELARYNKIEHQY 60

MAGSAPVPVGTDNEGSSAGGQSTQDEPATSD SQSVLGKEKTPMCLVNELARYNKIEHQY

Sbjct 21 MAGSAPVPVGTDNEGSSAGGQSTQDEPATSDASQSVLGKEKTPMCLVNELARYNKIEHQY 80

Query 61 RLTSEDGPPHKKKFTVTLKLGNEEYTAEGASIKKAQHAAAGIALKSTTYKHPPPKSSRQS 120

RLTSEDGPPHKKKFTVTLKLGNEEYTAEGASIKKAQHAAAGIALKSTTYKHPPPKSSRQS

Sbjct 81 RLTSEDGPPHKKKFTVTLKLGNEEYTAEGASIKKAQHAAAGIALKSTTYKHPPPKSSRQS 140

Query 121 RGSKSNITPTVELNALAMKRGEPTVYTLVESPQFHQYTYNHSHRGMYTQPYNDHFTFPYG 180

RGSKSNITPTVELNALAMKRGEPTVYTLVESPQFHQY Y+HSHRGMYTQPYNDHF PYG

Sbjct 141 RGSKSNITPTVELNALAMKRGEPTVYTLVESPQFHQYAYHHSHRGMYTQPYNDHF-LPYG 199

Query 181 RGKPNFRGGFNRVDPRFYGPGNKAPLYKVVVKVGEREFSGEGNSAQAARHDAASKALQLL 240

RGK NFR G+NR+DPR+YGPGNK PLYKVVVKVGEREFSGEGNSAQAARHDAASKALQLL

Sbjct 200 RGKLNFRAGYNRIDPRYYGPGNKVPLYKVVVKVGEREFSGEGNSAQAARHDAASKALQLL 259

Query 241 HSLPLDDTCPANSTICGGLTEQETELKSPISLVHEIALKRNLPVLFEVISEKGQPHMKTF 300

HSLP+DDTCPANSTICGGLTE E+ELKSPISLVHEIALKRNLPVLFEVISEKGQPHMKTF

Sbjct 260 HSLPVDDTCPANSTICGGLTEPESELKSPISLVHEIALKRNLPVLFEVISEKGQPHMKTF 319

Query 301 VTRCCVGDKFETTGEGNGKKISKKRAAEKMLEQLRTLPPTTTVTAPGCASALARAKRKAT 360

VTRCCVGDKFETTGEGNGKKISKKRAAEKMLEQLRTLPPTTTVTAPGCASALARAKRKAT

Sbjct 320 VTRCCVGDKFETTGEGNGKKISKKRAAEKMLEQLRTLPPTTTVTAPGCASALARAKRKAT 379

Query 361 TGKKKSRNIIKETPISQETKYDERDNSDEMNPISRLIQIQQAKKEREPVYTLREERGLPR 420

+GKKKSRNIIKETP+SQETKYDERDNSDEMNPISRLIQIQQAKKEREPVYTLREERGLPR

Sbjct 380 SGKKKSRNIIKETPVSQETKYDERDNSDEMNPISRLIQIQQAKKEREPVYTLREERGLPR 439

Query 421 RREFIMEVTVGKHSYIGSGPNKKVAKRNAAEGLLQQLGYASPTLSRSGHQLPQSQSQQSQ 480

RREFIMEVTVGKHSYIGSGPNKKVAKRNAAEGLLQQLGYASPTLSR+GH LPQ   QSQ

Sbjct 440 RREFIMEVTVGKHSYIGSGPNKKVAKRNAAEGLLQQLGYASPTLSRTGHPLPQ---LQSQ 496

Query 481 SQPSQQQHKVRFSDEKPEGGVSGRQLVPGLILMKDNKATNNGHKPGTGVNLKATIAKEYL 540

SQPS   HKVRFSDEKPEGGVSGRQLVPGLILMKDNKATNNGHKPGTGVNLKATIAKEYL

Sbjct 497 SQPS---HKVRFSDEKPEGGVSGRQLVPGLILMKDNKATNNGHKPGTGVNLKATIAKEYL 553

Query 541 SEGSSPTADALAGVKRQTKNSSVRPTEQLAYLASVLNFQVQFSDFPKGNHSEFLTLVSLD 600

SEGSSPTADALAGVKRQTKNSSVRPTEQLAYLASVLNFQVQFSDFPKGNHSEFLTLVSLD

Sbjct 554 SEGSSPTADALAGVKRQTKNSSVRPTEQLAYLASVLNFQVQFSDFPKGNHSEFLTLVSLD 613

Query 601 TDPPQVCHGAGATIDLSHEQAALNALRSLSKVGLDSITPAKKEATTTPQPVPKPEPGLYI 660

TDPPQVCHGAGATIDLSHEQAALNALRSLSKVGLDSITPAKKEATTTPQPVPKPEPGLYI

Sbjct 614 TDPPQVCHGAGATIDLSHEQAALNALRSLSKVGLDSITPAKKEATTTPQPVPKPEPGLYI 673

Query 661 DLKEETPYVNGK 672

DLKEETPYVNGK

Sbjct 674 DLKEETPYVNGK 685

RNA helicase Belle

>TRINITY_DN10378_c0_g3_i2 len=4495 path=[0:0-2105 2:2106-2245 4:2246-4494]

ACTGTTTTCTTATAAGAAAAATATTCTAGAAAGGAACAAATTTTAAGTAAGAATTATTTATTGGCCTTGGAG
GGGTTGTTGACATAATCGAAAGCTTCCGGTAAAATCGACTTATCGATGCTTAGTGTACATGTCCTCTGTATA
ATCCCGTGTACATCATTCCAGTTCTCTTTGTGTTTTACTTTTATTGGAAAAGCACTTGAAAGGGCTTACTTTT
AAAGTACTGATTTATAGTAATATGAGTAATGTACCCAACCAAAATGGATCAGGTCTAGAGCAGCGATTTGC
TGGTCTGGACTTGGAGGGTAGCCGTAAAGAAACCGGCCCCTACATTCCTCCCCATCTCCGAAACAAACAGG
CTGGAGAATCTCGTGGAGAACAACCACCCTCGACTAACTTCTCTTCTTACAACGACAGTCGTGAGTTTCCAA
GTAGAGGAGGTAGCGGTGGTGGATTCCGTAGTGGCGGCGGCAGCGGTGGTGGTAGTGGTGGAGGAGGCAG
CGCTGGTGGCGGTGGAGGTAGTAGAAGCGACAGTTATAGAGGAGGGAGTAGGAGAAATGGCGATTTTGAC
TCACAGAATGGAGACCGATCATGGGGTGATAGGGGAGGCTCTTCATTCAGAAGCAGCAGAGAAAAAGATA
GGGAACGTGATCGTGATGATTGGGGCAGTAGAAGAGGAGGTGGGAACACACCCACTGCAGGTAACACAGG
CAATGATAGATGGCAGGAGAAGCCCCAGGGGTCCTCTGCTGGCTGGACCACCCTTCTCCCCCGCGATGAGC
GACTTGAGTTTGAGCTCTTTGGCAATGCCAATACTGGTATTAACTTTAATAAGTATGAGGATATCCCTGTCG
AAGCAACTGGTGTCAAAGTACCACCCCAAATCCAAACGTTCGATGATTTGCAGATGAATGAAATAATATCG
AATAATATACGTCTAGCTCGTTATGATAAACCCACCCCAGTGCAGAAAAATGCCATACCTATCATCATGGG
CAAAAGAGATCTGATGGCTTGTGCCCAGACTGGAAGTGGCAAAACAGCTGCTTTTCTCATTCCTATCCTCA
ATCAAATTCTTGAAAAGGGACCACAGAATACTAGGTTTCCAGGAAAACGTAAGCAGTTCCCAATGGGCCTT
GTTCTTGCACCTACCAGGGAATTAGCTACCCAGATTTATGATGAAGCTCGCAAGTTTGCTTATAGATCTCGC
CTTCGACCTTGTGTTGTGTATGGAGGTGCTCATGTTGCCGATCAAATGAGAGATCTTGAGCGTGGATGTCAT
TTATTAGTTGCTACACCAGGACGTTTAGTCGACATGCTTTCAAGAGGAAGAATAGGCTTAGAAAATTGCAG
GTTCTTGGTGCTTGATGAAGCTGATAGAATGTTGGATATGGGTTTTGAACCTCAGATTAGAAGAATTGTTGA
AAAGAATTTCATGCCTCCAACTGGGGAAAGACAAACTCTGATGTTTTCTGCTACTTTCCCGAAAGAAATTC
AGATATTGGCATTGGATTTCTTGAATGACTATATATTTTTGGCCATTGGCCGTGTTGGATCTACCTCAGAAA
ATATTACCCAACAAATCATCTGGGTGGAAGAACATGACAAAAGAGGATTTCTGTTGGATCTTCTTAATGCTT
CGAAAAATGCTGAAGAAGGTTCATTGACTCTAGTATTTGTTGAAACAAAGAAGGGTGCAGATTCACTCGAA
GATTTCCTCTACAGCGTTGGTTACCCTGTGACTTCCATCCATGGTGATAGGACCCAGAAGGAAAGAGAAGA
AGCTCTGAGAAACTTCCGAACAGGTCAAACCCCTGTTCTTGTTGCTACTGCTGTTGCTGCCAGAGGTTTAGA
CATCCCTCATGTGAAACATGTCATCAATTTTGATTTACCTAATGATGTTGAAGAATATGTTCATAGAATTGG
ACGTACTGGCCGTATGGGAAATTTAGGGTTGGCAACATCCTTCTTCAACGATAAAAATAGGAATTTAGTTA
GGGATCTGTTCGATCTCATACAAGAAACCAAGCAAACCATGCCTTCATGGATGCAGAGAATGGCTGCGGAA
ACTAAACAGCCACTTTCTTCTCGCCGAGGCGGAAAAGGATTCTCCAGTGGGTTCGGTGCTCGGGACTACCG
TCAGCAGGGAGGAAGCAGTGGTGGCGGCGGAGGTGGTGGCCCTTCACGCAATCCCTCAAGGTCTGGAGGA
GGATCATCAGGATATGGTGGATACTCAAGTTCCAACAGTTACTACGGAGGGGGTAGCGGCAACGCAGGCT
ATGGTGGTTCCTATTCATCCAACCAGAATAATAGCTCCTCCGACCCCGATTGGTGGGGGCAATAATTCCTCA
CCGATTTCCCTGCCTCTCTCAGCCAATCGATCTCGCCCCCGCTCTTCTTCAGCCTAGCCACAAACTACAACT
ATGCACATATGGGCGTATACACAACTGTTATATAATGTTGTATGTACATATATAGATGTGTAAATATTATAT
ATGATCCCATACAACTCGAATGTTTATACGCCTAAAGAATATTAAAAATTCAGTATGTGTATTTTTAATAAG
TTAGGGGCATTAACACTGTGATTTTATTCTTACTGAATATATTTAATAAAGTCATAATGTGATTGTATATAT
ACATATTGGTAGGCCTTTTATAGGATCATTATGAGGGCCAACAACGATCGGATCGCTTTCAGGGAGGACAT
AACCAAAGATTCTCCAGTTAAGGACCCCTTTAGATTTGATCAAATAAGGTCTTTAGCACAGCTTTAAGGCTA
TCCGATGCATATACCTGTTTATTTATGTGTATATATATCACAGAGATATGTGCCACACCTAGACACGGCACT
CCAGTCTTCAGTTTAAGGAAGTAAATTGAGAAAAAAAAAATGTTGAGTTAAATGTAATTTTTTTAAGGGATG
ACAAATAATTTTTGTAAATATAAATGACAACTATCATAAGTTAGTGCCCATGATCTAACTTGGGGAAAAGT
TAATTGATTTATTGTGTACAAATATTTTCCTTTTGAGTAAATGAAAGCTAATAAATGCATATACCATTCTGA
ATTATTTATTATTAGTTTCACTCAAGGAGTGCTTGTATCAAAGGGATTGAACATCAGATACTCAATCTCAAG
TTAAAACTGAATTGATTTTTCCTTTTTTTTGTTTTTCTTTCTTTTTTTATTATTTTTCTTTCTTTATTTTGTTCCT
GTTTTATTTTTGTATATAAATAAAATTCGAACTGAAACTACTTACAATGTATTAGCTGTTAAAATGATCTGC
TAATATTTTATCATTATTAGGATATATATAATAAATATATATATTGCAAGTGCCTTATCTGGGGTAGAAGTA
TTGATTTTAGGCTCCAATAAGACAGTTTGCAACAACTTGAAAAAACGTAAAACAGCATTATTGTTTTTCTTC
TCACTTGTTTCTCTGTATTTATAAATTTCAGTATGTATTTTATCATTTTATTTCATCTTTTATAATATTTTAACT
GTATACAGATTGTTTATCTATAATAAAAATAATTTCTTCTTTGTCAAAATCTATGCCTTGTAAAGCTGCAGA
CGGGCGGACATCTTTGTGGGATATTTATATGATATAAGAAAATGAAAAAAGAAGAAATAATTATGAAAAA
TCCAAGATTCGTCTGAGAGAGGATTTTGAAAAAAGAAATATGTATTAATTACTATTCTATATTATAGCTGTC

TCCCAGTTTTCATTGTATGATTCTATATTATGTAGACATTAGGTCAAGTTTACCTATGTTTTAAATTACTGGC
AAGATGTAATGGTCATTTATGACTTTTGTATCTTAAAACAGCATCTATAGAACAATTCTCACTGCGATAGTT
TGAGATTGTGTTTAAGAAGAATGTTTTGATTAATTTTTTTTTCTTTTTTGTTAATGTTTGTATTTAATCATTTG
TCAGTTAACAACCATCCACCACTTTTATCCCAAAAGTTCATACATTTTAGAAAGTTTATGTGGAAAATCAAA
TTTCCTTTTGTAATGCTTTAAATGTTCAAATAAACAGATAGTTTTAATTGATATTTTAAAACAGTTTATTATT
GTCAGACACTGACCTTCATCCTTCACTAGTTCATGGTGGTAAAATAAATTAAATTATTCAAGATAAATACAA
TTTATTACAATAAACAACTATAAATATTTTAATAAGTAATTTGAATGTAAAAATATAACATTTATAATTAAT
ATTTAACATGAACTTTAATCAGAAGACAACAAATAAATATATTTTTATTTCTCTAACATAAGTAAGGACATA
TAAAAGTGTGTACATAGTACACATATATCAGTCAGCTAAGTATTTCTAAAAATTTTGAGATTCAAGCTAATA
ATTACTATGTATATATATATAAAACTAATCCTAGGTAAAAAAGTATATTGTCAGCTTTAAAGTAAAAGTCAT
TCATCTTTAATTTTATATTTATTAACATGAATGAAAGAATTAGAATGGTG

Protein

RF: +2

ORF: 239 -> 2344

Length: 702 aa

>lcl|ORF6_TRINITY_DN10378_c0_g1_i5:137:2995 unnamed protein product

MGGRDGRRGGARGGRGGRGAGSGGGRGGRDFGRGGFDRRDGFGSLKGKQPGGRLRKPSWNMANLEPFKKDF
YVPHPNVLNRTEDDIEKFRESKSITIKGDRIPPPIMRFEDVNFPDYVMNEVKKQGYDEPTPIQAQGWPIALSGRD
MVGIAQTGSGKTLAYILPAIVHINNQPRLRPRDGPIALILAPTRELAQQIQQVANDFGSHTSVRNTCIFGGAPKGG
QARDLERGVEIVIATPGRLIDFLEKNTTNLKRCTYLVLDEADRMLDMGFEPQIRKIIEQIRPDRQVLMWSATWPK
EVRNLAEEFLHNYIQLNIGSLELSANHNIQQYVDICDEYEKPDKLCDLLTDICREAESKTIIFTETKRSVDEIVRTIN
RRGWKASGIHGDKSQQDRDMVLNDFRTGRVNILVATDVAARGLDVEDVKFVVNYDYPSSSEDYVHRIGRTGR
SQRTGTSYTFFTTGNARQAKDLISVLQEAKQTLNPRLLELCEMAKCGVFGKPRSTRFAGKERGSKGGRGGRGG
RGGSERRGRPERRGPSSGSERRGSDRSGGGRGDRSSERRGGGSSDRWGRDSAPPSGRGGGFGNRIGSGGGFDSG
GRGALGGSAGFGERDPPRNSGFGNDGGRAGGFRPKPPSLMQAETFGDRNGPRGGNYSEGFGSDSGRGFGDAGG
GRGFGGDSNRGGFATENGGRRGFGGESRGGFGGDSGRGGFGGDEDDGFGRGRDSSSSKIGGRGGGSGAGGRG
GMSRGMSGSSGGAPPRGGGRGGGGLDRGGRGAPAGAAGRNSYSSAPAPTSRGTYGQDRLPSGGYETRQANSN
NLGYDRRSDRRSDWASESPVPEKRSRFSDNTSSTAAPPPPPLMSTTSAVTTKAAQMPANFSQPPPNYSKPPPNYH
TAVPQPAQAYQAAQQQPTNMMQYPQHMYQQQNYMMYQQMYQHQMNGYQQQQQTQQQTYQQQPPLPK

Conserved Domains

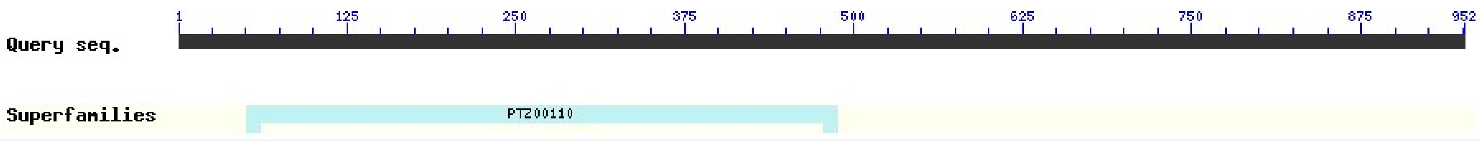

BLASTp

XP_014279436.1 ATP-dependent RNA helicase bel isoform X2 [*Halyomorpha halys*]

Score:340 bits

E-value: 0.0

Query 205 VEATGVQVPPQIQTFDDLQMNEIISNNIRLARYDKPTPVQKNAIPIIMAKRDLMACAQTG 264

\+ G ++PP I F+D+ + + N ++ YD+PTP+Q PI ++ RD++ AQTG

Sbjct 95 ITIKGDRIPPPIMRFEDVNFPDYVMNEVKKQGYDEPTPIQAQGWPIALSGRDMVGIAQTG 154

Query 265 SGKTAAFLIPILNQILERGPQNTRFPGKRKQFPMGLVLAPTRELATQIYDESRKFAYRSR 324

SGKT A+++P + I N + ++ P+ L+LAPTRELA QI + F +

Sbjct 155 SGKTLAYILPAIVHI------NNQPRLRPRDGPIALILAPTRELAQQIQQVANDFGSHTS 208

Query 325 LRPCVVYGGAHVAEQMRDLERGCHLLVATPGRLVDMLARGRIGLENCRYLVLDEADRMLD 384

+R ++GGA Q RDLERG +++ATPGRL+D L + L+ C YLVLDEADRMLD

Sbjct 209 VRNTCIFGGAPKGGQARDLERGVEIVIATPGRLIDFLEKNTTNLKRCTYLVLDEADRMLD 268

Query 385 MGFEPQIRRIVEKNSMPPTGERQTLMFSATFPKEIQILALDFLNDYIFLAIGRVG-STSE 443

MGFEPQIR+I+E+ + P +RQ LM+SAT+PKE++ LA +FL++YI L IG + S +

Sbjct 269 MGFEPQIRKIIEQ--IRP--DRQVLMWSATWPKEVRNLAEEFLHNYIQLNIGSLELSANH 324

Query 444 NITQQIIWVEEHDKRGFLLDLL-NASKNAEEGSLTLVFVETKKGADSLEDFLYGVGYPVT 502

NI Q + +E++K L DLL + + AE S T++F ETK+ D + + G+ +

Sbjct 325 NIQQYVDICDEYEKPDKLCDLLTDICREAE--SKTIIFTETKRSVDEIVRTINRRGWKAS 382

Query 503 SIHGDRTQKEREEALRNFRTGQTPVLVATAVAARGLDIPHVKHVINFDLPNDVEEYVHRI 562

IHGD++Q++R+ L +FRTG+ +LVAT VAARGLD+ VK V+N+D P+ E+YVHRI

Sbjct 383 GIHGDKSQQDRDMVLNDFRTGRVNILVATDVAARGLDVEDVKFVVNYDYPSSSEDYVHRI 442

Query 563 GRTGRMGNLGLATSFFNDKNRNLVRDLFDLIQETKQTMPSWMQRMAAETK-----QPLSS 617

GRTGR G + +FF N +DL ++QE KQT+ + + K +P S+

Sbjct 443 GRTGRSQRTGTSYTFFTTGNARQAKDLISVLQEAKQTLNPRLLELCEMAKCGVFGKPRST 502

Query 618 RRGGK 622

R GK

Sbjct 503 RFAGK 507

Protein arginine methyltransferase 7 (PRMT)

>TRINITY_DN4183_c0_g1_i2 len=1538 path=[0:0-715 2:716-1537]

CATGTATTTATGTTGTTAAAGATATTTTCTGCAGTAGGGGTCCCCATCTTAATAATTTAGAATGTTGATTATT
TAAAAACATGTTAGCCCTTCAAAATTTTGGAAAAATTATTAAAACAGTTTTAGTCGAAGGGAAACAGTTTTT
TTCAAACATACCTCGCTATAAATATGATGAAACTTGTTATAACTTGTTAAAATCCAGGATAAAATTTTCTGG
ACCTATTACAGTGTATGATTACATGAAAGAAGTTCTAACTAATCCTGCCAGTGGTTATTATGTTACAAAAAA
TGTTATTGGAAAAGAAGGTGACTTTATCACTTCTCCTGAAATTTCTCAATTGTTGGGAGAAATGGTTGCTTT
GTGGACTTTAAATGAATGGACCAAGTTGGGCTCTCCAAAACCTCTCCAGTTAGTTGAGTTGGGACCAGGAA
AAGGATCTATGATGCATGACATTTTAAGGGTCTATAAGCAATTAAAACTTGATGAACATATCACTGTTCATC
TTGTTGAAGTTAGCAATGAGCTCAGTAAAGTTCAAGGGAACAAACTTTGTACTTCCATTTTAAAACATGAA
AATAAAACATGTTATCAAGAAGGGAAAACAGAAAATAATGTGTCCATTTATTGGCATAAGGCTATTCAGGA
TGTGCCCAAAAAATTTTCCTGTATTTTAGCTCATGAATTTTTCGATGCATTGCCTGTTCATATTCTTAAGCAA
ACAGTAGATGGTTGGCGGGAAGTCTTAATAGATCTCACTGAATGTGGTACCAAACTTCGTTTTGTTATCTCA
CCTCGTCCTACACCAGCTTGTGTGTTTAGAAAATACTCAAATGGTCGAGATTATTTTGAAATTAGTCCACAG
TCAGGTCTGATACTTGAGCACATCGCCTGCACATTAGAGGAATATGGTGGCTTTGCCTTAATTATAGACTAT
GGTCATGATGGAGAAAAGAAAGATACATTCAGGGGTTTCAGAAACCATCAATTAGTGGATCCATTAGAAG
CTCCAGGCACCTCAGATTTGACCGCAGATGTCGATTTCGCTTTTCTTAAAAATGTTACTAAGGATAAGCTAA
TTTCTTTTGGTCCTGTTTCACAAAGGTCTTTTCTGAAACAATTGCACATAGATGTTCGCTTACAGGTACTCTT
AAAGAGCTGTAAGAATGAAAAAGAAAAAAATGACATCTTGTCTGGTTACCACATGATCATGGATTCTGATA
AGATGGGGAAATGTTTCAAAGTGATGGCATTATATCCTGCTGTTCTTAAAGAGTTCTTCAATGATTTTCCAG
TAGAAGGATTTGCAAATAAATAACCATTTATTAGTTTTTTAAGTATTTAGGATACAATAGAGTGTTTTTCTC
TCTTTATTTACTATTAAAAATATACTTATATATTAAATATTGTAATATTAAAATTTTAATTTTCTTCCAAGAT
TTGTATTTTTCAAATTTAGGCAGCCTCCTGTATTTCATTTATCCTTAAAGCATCCAGAGAAAGTTGTTTCTGA
AACAACAATGACATGTTAGTACTGTAC

Protein

RF: +3

ORF: 81 -> 1316

Length: 411 aa

>lcl|ORF4_TRINITY_DN4183_c0_g1_i2:80:1315 unnamed protein product

MLALQNFGKIIKTVLVEGKQFFSNIPRYKYDETCYNLLKSRIKFSGPITVYDYMKEVLTNPASGYYVTKNVIGKE
GDFITSPEISQLLGEMVALWTLNEWTKLGSPKPLQLVELGPGKGSMMHDILRVYKQLKLDEHITVHLVEVSNEL
SKVQGNKLCTSILKHENKTCYQEGKTENNVSIYWHKAIQDVPKKFSCILAHEFFDALPVHILKQTVDGWREVLI
DLTECGTKLRFVISPRPTPACVFRKYSNGRDYFEISPQSGLILEHIACTLEEYGGFALIIDYGHDGEKKDTFRGFRN
HQLVDPLEAPGTSDLTADVDFAFLKNVTKDKLISFGPVSQRSFLKQLHIDVRLQVLLKSCKNEKEKNDILSGYH
MIMDSDKMGKCFKVMALYPAVLKEFFNDFPVEGFANK

Conserved Domains

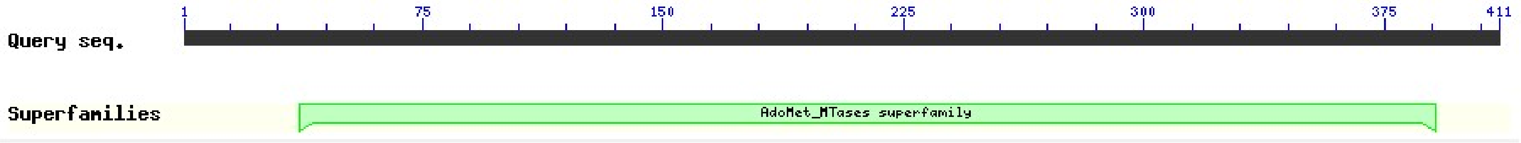

BLASTp

XP_014292128.1 protein arginine methyltransferase NDUFAF7, mitochondrial [*Halyomorpha halys*]

Score:749 bits

E-value: 0.0

Query 1 MMFLQGLRKIIKTVLTERKIFFSSIPLNKSDETCFNLLKSRINFAGPLTVYDYMKEVLTN 60

M+ LQ KIIKTVL E K FFS+IP K DETC+NLLKSRI F+GP+TVYDYMKEVLTN

Sbjct 1 MLALQNFGKIIKTVLVEGKQFFSNIPRYKYDETCYNLLKSRIKFSGPITVYDYMKEVLTN 60

Query 61 PVSGYYVTKNVIGKQGDFITSPEISQLLGEMVALWTLNEWTKLGSPKPFQLVELGPGKGS 120

P SGYYVTKNVIGK+GDFITSPEISQLLGEMVALWTLNEWTKLGSPKP QLVELGPGKGS

Sbjct 61 PASGYYVTKNVIGKEGDFITSPEISQLLGEMVALWTLNEWTKLGSPKPLQLVELGPGKGS 120

Query 121 MMHDILRVCKQLKLDEHINVHFVEVSDELSKAQGDKLCTSVLKHDNKSYYQEGKTENNVP 180

MMHDILRV KQLKLDEHI VH VEVS+ELSK QG+KLCTS+LKH+NK+ YQEGKTENNV

Sbjct 121 MMHDILRVYKQLKLDEHITVHLVEVSNELSKVQGNKLCTSILKHENKTCYQEGKTENNVS 180

Query 181 IYWHKAIQDVPKNFTCILAHEFFDALPVHILKQTVDGWREVLIDLTECCSKLRYVISPAP 240

IYWHKAIQDVPK F+CILAHEFFDALPVHILKQTVDGWREVLIDLTEC +KLR+VISP P

Sbjct 181 IYWHKAIQDVPKKFSCILAHEFFDALPVHILKQTVDGWREVLIDLTECGTKLRFVISPRP 240

Query 241 TPACVFSKYSNGRDYFEISPQSGLILEHIACTLEEHGGFVLIIDYGHDGEKKDTFRGFRN 300

TPACVF KYSNGRDYFEISPQSGLILEHIACTLEE+GGF LIIDYGHDGEKKDTFRGFRN

Sbjct 241 TPACVFRKYSNGRDYFEISPQSGLILEHIACTLEEYGGFALIIDYGHDGEKKDTFRGFRN 300

Query 301 HQLVDPLVSPGTSDLTADVDFAFLKNVTKDKLLSFGPVPQRSFLKELHIDVRLQVLLKNC 360

HQLVDPL +PGTSDLTADVDFAFLKNVTKDKL+SFGPV QRSFLK+LHIDVRLQVLLK+C

Sbjct 301 HQLVDPLEAPGTSDLTADVDFAFLKNVTKDKLISFGPVSQRSFLKQLHIDVRLQVLLKSC 360

Query 361 KNEKEKNDILSGYHMIMDSDKMGECFKVMSLFPSVLKELFNDYPVVGFVNK 411

KNEKEKNDILSGYHMIMDSDKMG+CFKVM+L+P+VLKE FND+PV GF NK

Sbjct 361 KNEKEKNDILSGYHMIMDSDKMGKCFKVMALYPAVLKEFFNDFPVEGFANK 411

Gawky

>TRINITY_DN12193_c0_g2_i6 len=4865 path=[0:0-54 2:55-2669 3:2670-2795 4:2796-3782 5:3783-3905 6:3906-4864]

AGATGTGCGACAACTCCGATTTGGCTTCCTCGTTCGGCCATTGCCTGCTCTAAGTATTTCTGCCACAATGAG
AAAAGCCCATGATAATTAATTAGTCTGTAAGACTTTGGTTTTTAAATTTTGCCTGCCAAAGACGTACTGAGA
GCCAATGTTTCGAAACAATTCTAGTTCAAATGAGATTTCTTCTAAAACTACTAATGCCTTTGTACAAAATAA
AGACGAGGAGGACAAATCTGAGAGCTTGTTAAGAGGTATGGCGCAGCCTCCCAAGCCCACAAGTCCTACT
CTTCAAGTGCCTGATAAAAGGGACGTAATGGTGGTAGATATTGGGGTGAGAGAAGAAGATGGCCCCGTCC
TGACTGTGATAACCAACCATCCGTCCCAAGCGCCCGCCAAGATTTCCTCATCAGAAATTGGTGAATGTGAA
TCTGACGGTTCTTCCAAAATGCCACTAGCAGAGACACAAGGAACGGGTGCTCTTTGCTTAGATAGCATTAA
GTCTATTAGTGTTAATGAATCATTTAGTGTTAAGGATAAATTTATTTACCCGAGCAAGAGTTCAATTCTGCC
GCCAAACATTCCAAAAACCAACGATGATACCGATCAAGATGTTAAAAGCTTTAAAATCTGTGATTATTATA
CTCGGTGGGGAATACCACGAAACTTCAAACTGTTAGGAGGAGGAGAGAGTTCTCTTACTACGGGAACTACT
GGTTGGGGGTCACCACCTTCAAATCAAGGTGGAAGTTCAGGATGGAACAGTGCAACTACTAGTGGAAGTA
ATAGTTCTTCCGGACAAGGACAAGCGGGTGCTGGACAAAGTCCAGCTCCTGCCTCTGCTGGACAAACTTGG
GGTAGTACCCAAAACAATACCAACAACAGCAATAGCAATAGTAACAATAATAATGGATCTCGTAATGCAG
TCAGCCAGCAAGGTGGAAGCACACAACAGCAATCAGGAGGGGGGCCACCTAGTCAGTCCACGGCTCCACC
TGTAGCAGCTGTGTCTACATCAACTGTTACGACTGCTCCAGCCTCTTCAGCAACAAATACGTCCAATATAAA
TACTGCTACAACATCATCTTCTCAACAAAATGGTTCAAGTTCTGGCAATCAAGTAGTAGGAAGTGGTTCTAC
TTGGGCGACTGCAGTTGGCAAAGGACTTCCTCCGACTAGCACAGCTACAACTCCCACCTCAAGTGGAAGCA
CATCTACTAAACAACAAATGGAACAACTCAACACAATGAGGGAAGCTCTCTACAGTCAAGATGGATGGGG
TGGTCAAAATGTCAACCAAGACAGCAATTGGGATATACCAGGTTCTCCAGAACCAGGCACAAAAGACAGC
AACAATGCAGCCCCTGTTCCTCTTTGGAAACTACCTATTAATAATGGCACTGATCTTTGGGAGGCTAATCTA
CGAAACGGAGGTGTTCCTCCACCCGTGAGCCAGCAGAGCCAGAAAACACCTTGGGTTCACACTCCCAGCAC
TAACATCGGTGGTACGTGGGGCGAAGATGATGAAGGTGATGCCTCCAACGTATGGACTGGTGTTCCTCAAG
CTCAGACTGGATGTGGTCCCCAGTGGCCTGCTCAACCACCTCCCATTTGGCCTGCTACCAAAAAAGAAGGA
GATTGGGGAGGACCTAACTGGAATGATCAACGTGACACAAGAGACCTTCGTCACAGCGATATGAGACAAA
TAATGGATGCTAGAGAACATATGAGACCAACTTCTATTGATCACAGATCAATGGGTGGGAATGATGTGATT
ATGAGAGGTGACCCACGAGGAATTAGTGGTCGTCTTAATGGAGTGACCAGTGAGGCAATGTGGCCTGGCCC
AGGGCCCCATCATCACATACCTCATCATCAAGGAAAATTACCATCTCAACCTAACCAACCAGTTAATCAAT
GGAGTAGTACTGGGCCTCCAATGAAGGACATGTCTGGTCTCGGTGGTAAATCAACTGGTTGGGAAGAACCT
TCGCCTCCAGCTCAACGAAGGAATATGCCCAACTATGATGATGGAACATCGCTTTGGGGCCCACAGCATCC
CAGGCCCTCAATCCAAGGCCAGAACAAGGTTTCTCATTGGAAAGAAATGCCTACTCCTGGTATAGGTCGTG
GTGGCTTACAGTGTCCTCCTGGTCGTGCAAACCCTACTATGAAACCTGATCAACCTTTATGGCCTCATCATC
CAAGGAATGAACGTGGATGGGAAGGAGGAATGGATAGCGGTCCATGGGGAGATGAAAAACCAACACCTA
CCGCTGCTCCTTGGATGGACCAAGGACTAGCTCCTTCTTCATGGCAAGGCGGACCAAAACATAAACCTGCC
TGGGATGGCTCTGATTTAGATCCCACTTCTTGGGTTCACTCGAAACAGCCTTCAAAGTCTGTTTCAAAAGAA
TTTATCTGGACAAGCAAGCAATTTAGAATTTTATCAGAAATGGGCTTTAAGAAGGAAGATATTGAAAGTGC
ATTAAGAAGTTCCGGTATGAGTCTTGAAGATGCATTGGATCAGTTGAACACCAACAGCAGGGGACTAAGCG
CTGGAGGTGGCGGTGAGAGATGGCCTCGTCATGGTGATTTAGATCCTGATCATGCTGCTATTATGAATGCAT
TTCCTTCACCTCAACAAACCATTTGTCTTGCTCCATATCCACAGGGTGGAGGTGGTGGCGGAAGTGGAAGT

GGTCCTGGAGGTGGCCCTACCTTAGCGACCATAACACCAGCTGTAATGCAGAAACTTCTTGCACAGCAACC
TCCACAGCAGCAACCTTTTGCTCAGCAATCTTCAAGAACACAGCAAACTCAGCAGCCATCAGCTCAGCAGC
TTAGGATGTTAGTTCAGCAAATTCAAATGGCAGTTCAAACTGGTTACCTCAGCCCTCAGATTTTAAACCAAC
CATTAGCTCCACAAACACTTATTTTATTGAATCAACTTCTACAACAGATTAAGAATCTACAACAGCTTATGC
AACACCATACTGTCATGCAAGTTAATCCTCTTGGTAAACCAAGCTCAAATCACCTATTACAATTATCAGTGC
AGATCACCAAGACTAAGCAACAAATAACCAATCTTCAGAATCAGATAGCTGCTCAACAAGCTGTGTATGTT
AAGCATCAACAACATACACCGCCTACGTCTGAATTTTTTAAGAGTTCATTACATGAACCGATTTCTGCACTT
CATCCAAATTTCTCTGATCTTTCTCTCAAAGATCCTCCGACTAGTGGAACAAGTCAACAGTCACGATTAAAT
CAGTGGAAGTTACCTGCACTAGAAAAAGACTCTGATATTGGATCCGGTGAATTTTCAAGAGCTCCTGGCAC
AACTTCTAAATCAGCTCAAGGCTCATCTTCACCCAATACCAATCTATTACTTGGGCAGGCTGATGGTACCTG
GTCTTCTGTAAATCGCGAGTCCAGTTGGCCTGATTCCTCAGGAGATGATGCATCTGGTAAGGATTGGCCGA
ATTCCAGCCAACCTCCATCTCAAGCATTCTCAGATCTTGTTCCCGAATTTGAACCAGGCAAACCTTGGAAGG
GAAACCCATTGAAAAGCATCGAGGATGACCCAAGCCTTACACCAGGTTCTGTTGTAAGGTCTCCTCTTTCG
CTGCCTTCAATAAAGGATACACATATTCTCTCAACTAGTACCGGAGCTGGTAAAGCCTCACCTACTACCAGT
TCCTCCTTGGATATCATTCCTTCTCTTGGTTTATCATCCTCTACTTGGAGCTTTAATCCACCACCTTCTTCATC
CAACACCAGTGTGAAGCTGAACTCTAGTGGAGCGGGTGGAGGTGGTAGTGGATCAACATCAAATAATGGT
GGAGGCAAAAATAGTGCTTCAACTTGGGAAACTAATTCATCTGAATTGTGGGCTCCTAAAAGAGGGCCTCC
CCCAGGTTTACCAGCCAAACCTTCTGGTAGCTCCAGTGGTGGGCAGGCTACTAACGGTTGGGGACCATTGT
CTAGTAGCGGCCGTTGGTCTACTGGGCAAGGTTGGCCTGGGCCGAATCAGGCGGCTGCAACTCAGCCAGGA
TCAACTTGGTTGTTATTGCGCAATCTTACTCCTCAGATTGATGGTTCGACTTTGAAAACTCTGTGTTTACAAC
ATGGGCCTCTATCTAATTTTCATCTCTACCTAAACCATGGCATTGCTCTTGCTAAATATGCTTCTCGTGAAGA
AGCCAACAAGGCCCAGGGTGCTCTAAACAATTGTGTCCTTGGCAACACTACAATCTTTGCCGAGAGCCCCA
GTGAGACGGATGTACTATCATTACTTCAGCACCTCGGTGGGCAGGGGAGCGCTGCCAGCGGCAGCTCCGCC
TGGCGTGGCAAGGAGGCCTGGGGCAACTCTCAGCTGTGGGGAGCCACAGGAGCCAGCTCCACTGCTGCTTC
CCTCTGGGCAGGAGATAGTGACCAGCATCGCAACACTCCATCCTCAATTAACTCATATTTGCCAGGTGACC
TTCTTGGTGGTGAGTCCATTTAGGCAAATCCTCTTCATTCTTCTCTCAATACTTCACCAAATTCTTCTCGATC
TATAAATACGTCAATCAAAACTATTGAACAAAAAAAATATAAAAAAACAAAAAAAAAACAAATACTTTGA
TCTCAAAAACACCACATGACCTTTTATTATAAATATATATGATATGAAGTATATGCAATTAATTATTTGTA
CCAGGAACTTATATCTTATTATTAATATAATTATTATTATTGTATTTTAAAGAATGAATTTGATAATGAGTTT
TAAATTATATAACGACATGTGTAGTTCCTGTCGAACAAAATCAGACTGTTTTGAGTATTTGTTAAAAACGAT
TCAATATTACTAAGTAAT

Protein

<u>RF:</u> +2

<u>ORF:</u> 149 -> 4510

<u>Length</u>: 1453 aa

>lcl|ORF5_TRINITY_DN12193_c0_g2_i6:148:4509 unnamed protein product

MFRNNSSSNEISSKTTNAFVQNKDEEDKSESLLRGMAQPPKPTSPTLQVPDKRDVMVVDIGVREEDGPVLTVITN
HPSQAPAKISSSEIGECESDGSSKMPLAETQGTGALCLDSIKSISVNESFSVKDKFIYPSKSSILPPNIPKTNDDTDQ
DVKSFKICDYYTRWGIPRNFKLLGGGESSLTTGTTGWGSPPSNQGGSSGWNSATTSGSNSSSGQGQAGAGQSPA
PASAGQTWGSTQNNTNNSNSNSNNNNGSRNAVSQQGGSTQQQSGGGPPSQSTAPPVAAVSTSTVTTAPASSAT
NTSNINTATTSSSQQNGSSSGNQVVGSGSTWATAVGKGLPPTSTATTPTSSGSTSTKQQMEQLNTMREALYSQD
GWGGQNVNQDSNWDIPGSPEPGTKDSNNAAPVPLWKLPINNGTDLWEANLRNGGVPPPVSQQSQKTPWVHTP
STNIGGTWGEDDEGDASNVWTGVPQAQTGCGPQWPAQPPPIWPATKKEGDWGGPNWNDQRDTRDLRHSDMR
QIMDAREHMRPTSIDHRSMGGNDVIMRGDPRGISGRLNGVTSEAMWPGPGPHHHIPHHQGKLPSQPNQPVNQW
SSTGPPMKDMSGLGGKSTGWEEPSPPAQRRNMPNYDDGTSLWGPQHPRPSIQGQNKVSHWKEMPTPGIGRGGL
QCPPGRANPTMKPDQPLWPHHPRNERGWEGGMDSGPWGDEKPTPTAAPWMDQGLAPSSWQGGPKHKPAWD
GSDLDPTSWVHSKQPSKSVSKEFIWTSKQFRILSEMGFKKEDIESALRSSGMSLEDALDQLNTNSRGLSAGGGGE
RWPRHGDLDPDHAAIMNAFPSPQQTICLAPYPQGGGGGGSGSGPGGGPTLATITPAVMQKLLAQQPPQQQPFAQ

QSSRTQQTQQPSAQQLRMLVQQIQMAVQTGYLSPQILNQPLAPQTLILLNQLLQQIKNLQQLMQHHTVMQVNP
LGKPSSNHLLQLSVQITKTKQQITNLQNQIAAQQAVYVKHQQHTPPTSEFFKSSLHEPISALHPNFSDLSLKDPPTS
GTSQQSRLNQWKLPALEKDSDIGSGEFSRAPGTTSKSAQGSSSPNTNLLLGQADGTWSSVNRESSWPDSSGDDA
SGKDWPNSSQPPSQAFSDLVPEFEPGKPWKGNPLKSIEDDPSLTPGSVVRSPLSLPSIKDTHILSTSTGAGKASPTT
SSSLDIIPSLGLSSSTWSFNPPPSSSNTSVKLNSSGAGGGGSGSTSNNGGGKNSASTWETNSSELWAPKRGPPPGLP
AKPSGSSSGGQATNGWGPLSSSGRWSTGQGWPGPNQAAATQPGSTWLLLRNLTPQIDGSTLKTLCLQHGPLSNF
HLYLNHGIALAKYASREEANKAQGALNNCVLGNTTIFAESPSETDVLSLLQHLGGQGSAASGSSAWRGKEAWG
NSQLWGATGASSTAASLWAGDSDQHRNTPSSINSYLPGDLLGGESI

Conserved Domains

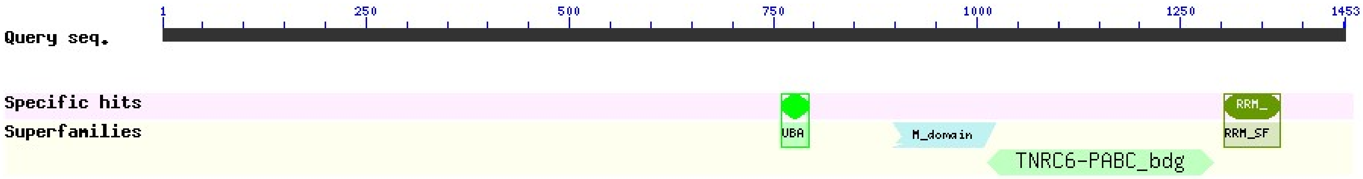

BLASTp

XP_014288686.1 protein Gawky isoform X1 [*Halyomorpha halys*]

Score:2830 bits

E-value: 0.0

Query 1 MFRNNSSSNEISSKTTNAFVQNKDEEDKSESLLRGMAQPPKPISPTLQVPEKRDVMVVDI 60

MFRNNSSSNEISSKTTNAFVQNKDEEDKSESLLRGMAQPPKP SPTLQVP+KRDVMVVDI

Sbjct 1 MFRNNSSSNEISSKTTNAFVQNKDEEDKSESLLRGMAQPPKPTSPTLQVPDKRDVMVVDI 60

Query 61 GVREEDGPVLTVITNHPSQAPAKISSSEIDECESDGSSKMPLAETHGTGALCLDSIKSIS 120

GVREEDGPVLTVITNHPSQAPAKISSSEI ECESDGSSKMPLAET GTGALCLDSIKSIS

Sbjct 61 GVREEDGPVLTVITNHPSQAPAKISSSEIGECESDGSSKMPLAETQGTGALCLDSIKSIS 120

Query 121 VNKSFSVKDKFIYPSKSSILPPNIPKTNDDTDQDVKSFKICDYYTRWGIPRNFKLLGGGE 180

VN+SFSVKDKFIYPSKSSILPPNIPKTNDDTDQDVKSFKICDYYTRWGIPRNFKLLGGGE

Sbjct 121 VNESFSVKDKFIYPSKSSILPPNIPKTNDDTDQDVKSFKICDYYTRWGIPRNFKLLGGGE 180

Query 181 SSLTTGTTGWGSPPSNQGGSSGWNSASTTSGNNSSSGQGQAGAGQSPAPASAGQTWGSSQ 240

SSLTTGTTGWGSPPSNQGGSSGWNSA TTSG+NSSSGQGQAGAGQSPAPASAGQTWGS+Q

Sbjct 181 SSLTTGTTGWGSPPSNQGGSSGWNSA-TTSGSNSSSGQGQAGAGQSPAPASAGQTWGSTQ 239

Query 241 NNTNNSNSSSNNNNGSRNSVSQQGGGSTQQQPGGGPPSQSTAPPVATVSTPTVTTAPASS 300

NNTNNSNS+SNNNNGSRN+VSQQGG STQQQ GGGPPSQSTAPPVA VST TVTTAPASS

Sbjct 240 NNTNNSNSNSNNNNGSRNAVSQQGG-STQQQSGGGPPSQSTAPPVAAVSTSTVTTAPASS 298

Query 301 ATNTSNINTATTSSSQQNGSTTGNQVVGSGSTWATAVGKGLPPTSTATTPTSSGSTSTKQ 360

ATNTSNINTATTSSSQQNGS++GNQVVGSGSTWATAVGKGLPPTSTATTPTSSGSTSTKQ

Sbjct 299 ATNTSNINTATTSSSQQNGSSSGNQVVGSGSTWATAVGKGLPPTSTATTPTSSGSTSTKQ 358

Query 361 QMEQLNTMREALYSQDGWGGQNVNQDSNWDIPGSPEPGTKDSNNAAPVPLWKLPINNGTD 420

QMEQLNTMREALYSQDGWGGQNVNQDSNWDIPGSPEPGTKDSNNAAPVPLWKLPINNGTD

Sbjct 359 QMEQLNTMREALYSQDGWGGQNVNQDSNWDIPGSPEPGTKDSNNAAPVPLWKLPINNGTD 418

Query 421 LWEANLRNGGVPPPVSQQSQKTPWVHTPSTNIGGTWGEDDEGDASNVWTGVPQAQTGCGP 480

LWEANLRNGGVPPPVSQQSQKTPWVHTPSTNIGGTWGEDDEGDASNVWTGVPQAQTGCGP

Sbjct 419 LWEANLRNGGVPPPVSQQSQKTPWVHTPSTNIGGTWGEDDEGDASNVWTGVPQAQTGCGP 478

Query 481 QWPAQPPPIWPATKKEGDWGGPNWNDQRDTRDLRHSSDMRQMMDARDHMRP-SIDHRSMG 539

QWPAQPPPIWPATKKEGDWGGPNWNDQRDTRDLRHS DMRQ+MDAR+HMRP SIDHRSMG

Sbjct 479 QWPAQPPPIWPATKKEGDWGGPNWNDQRDTRDLRHS-DMRQIMDAREHMRPTSIDHRSMG 537

Query 540 GNDVIMRGDPRGISGRLNGVTSEAMWPGPGPHHHIPHHQGKLPSQPNQPVNQWSSTGPPM 599

GNDVIMRGDPRGISGRLNGVTSEAMWPGPGPHHHIPHHQGKLPSQPNQPVNQWSSTGPPM

Sbjct 538 GNDVIMRGDPRGISGRLNGVTSEAMWPGPGPHHHIPHHQGKLPSQPNQPVNQWSSTGPPM 597

Query 600 KDMTGLGGKSTGWEEPSPPAQRRNMPNYDDGTSLWGPQHPRPTIQGQNKVSHWKEMPTPG 659

KDM+GLGGKSTGWEEPSPPAQRRNMPNYDDGTSLWGPQHPRP+IQGQNKVSHWKEMPTPG

Sbjct 598 KDMSGLGGKSTGWEEPSPPAQRRNMPNYDDGTSLWGPQHPRPSIQGQNKVSHWKEMPTPG 657

Query 660 IGRGGLQCPPGRANPTMKPEQPLWPHHPRNERGWEGGMDSGPWGDEKPTPTAAPWMDQGL 719

IGRGGLQCPPGRANPTMKP+QPLWPHHPRNERGWEGGMDSGPWGDEKPTPTAAPWMDQGL

Sbjct 658 IGRGGLQCPPGRANPTMKPDQPLWPHHPRNERGWEGGMDSGPWGDEKPTPTAAPWMDQGL 717

Query 720 APSSWQGGPKHKPAWDGSDLDPTSWVHSKQPSKSVSKEFIWTSKQFRILSEMGFKKEDIE 779

APSSWQGGPKHKPAWDGSDLDPTSWVHSKQPSKSVSKEFIWTSKQFRILSEMGFKKEDIE

Sbjct 718 APSSWQGGPKHKPAWDGSDLDPTSWVHSKQPSKSVSKEFIWTSKQFRILSEMGFKKEDIE 777

Query 780 SALRSSGMSLEDALDQLNTN-RGLSGGGGGGERWPRHGDLDPEHAAIMNAFPSPQQTICLA 838

SALRSSGMSLEDALDQLNTN RGLS GGGGERWPRHGDLDP+HAAIMNAFPSPQQTICLA

Sbjct 778 SALRSSGMSLEDALDQLNTNSRGLSAGGGGERWPRHGDLDPDHAAIMNAFPSPQQTICLA 837

Query 839 PYPQGGGGGGSGSGPGGGPTLATITPAVMQKLLAQQPPQQQPFAQQSSRTQQTQQPSAQQ 898

PYPQGGGGGGSGSGPGGGPTLATITPAVMQKLLAQQPPQQQPFAQQSSRTQQTQQPSAQQ

Sbjct 838 PYPQGGGGGGSGSGPGGGPTLATITPAVMQKLLAQQPPQQQPFAQQSSRTQQTQQPSAQQ 897

Query 899 LRMLVQQIQMAVQTGYLSPQILNQPLAPQTLILLNQLLQQIKNLQQLMQHHTVMQVNPLG 958

LRMLVQQIQMAVQTGYLSPQILNQPLAPQTLILLNQLLQQIKNLQQLMQHHTVMQVNPLG

Sbjct 898 LRMLVQQIQMAVQTGYLSPQILNQPLAPQTLILLNQLLQQIKNLQQLMQHHTVMQVNPLG 957

Query 959 KPSSNHLLQLSVQITKTKQQITNLQNQIAAQQAVYVKHQQHTPPTTEFFKSSLHEPISGL 1018

KPSSNHLLQLSVQITKTKQQITNLQNQIAAQQAVYVKHQQHTPPT+EFFKSSLHEPIS L

Sbjct 958 KPSSNHLLQLSVQITKTKQQITNLQNQIAAQQAVYVKHQQHTPPTSEFFKSSLHEPISAL 1017

Query 1019 HPNFSDLSLKDPPTSGTSQQSRLNQWKLPALEKDSDIGSGEFSRAPGTTAKSAQGSSSPN 1078

HPNFSDLSLKDPPTSGTSQQSRLNQWKLPALEKDSDIGSGEFSRAPGTT+KSAQGSSSPN

Sbjct 1018 HPNFSDLSLKDPPTSGTSQQSRLNQWKLPALEKDSDIGSGEFSRAPGTTSKSAQGSSSPN 1077

Query 1079 TNLLLGQADGTWSSVNRESSWPDSAGDDASGKDWPNSSQPPSQAFSDLVPEFEPGKPWKG 1138

TNLLLGQADGTWSSVNRESSWPDS+GDDASGKDWPNSSQPPSQAFSDLVPEFEPGKPWKG

Sbjct 1078 TNLLLGQADGTWSSVNRESSWPDSSGDDASGKDWPNSSQPPSQAFSDLVPEFEPGKPWKG 1137

Query 1139 NPLKSIEDDPSLTPGSVVRSPLSLPSIKDTHILSTSTGAGKASPTTSSSLDIIPSLGLSS 1198

NPLKSIEDDPSLTPGSVVRSPLSLPSIKDTHILSTSTGAGKASPTTSSSLDIIPSLGLSS

Sbjct 1138 NPLKSIEDDPSLTPGSVVRSPLSLPSIKDTHILSTSTGAGKASPTTSSSLDIIPSLGLSS 1197

Query 1199 STWSFNPPPSSSNTSVKLNSSGAGGGGSGSTSNNGGGKNSASTWETNSSELWAPKRGPPP 1258

STWSFNPPPSSSNTSVKLNSSGAGGGGSGSTSNNGGGKNSASTWETNSSELWAPKRGPPP

Sbjct 1198 STWSFNPPPSSSNTSVKLNSSGAGGGGSGSTSNNGGGKNSASTWETNSSELWAPKRGPPP 1257

Query 1259 GLPAKPTGGSSGGQATNGWGPLSSSGRWSTGQGWPGPNQAAATQPGSTWLLLRNLTPQID 1318

GLPAKP+G SSGGQATNGWGPLSSSGRWSTGQGWPGPNQAAATQPGSTWLLLRNLTPQID

Sbjct 1258 GLPAKPSGSSSGGQATNGWGPLSSSGRWSTGQGWPGPNQAAATQPGSTWLLLRNLTPQID 1317

Query 1319 GSTLKTLCLQHGPLSNFHLYLNHGIALAKYASREEANKAQGALNNCVLGNTTIFAESPSE 1378

GSTLKTLCLQHGPLSNFHLYLNHGIALAKYASREEANKAQGALNNCVLGNTTIFAESPSE

Sbjct 1318 GSTLKTLCLQHGPLSNFHLYLNHGIALAKYASREEANKAQGALNNCVLGNTTIFAESPSE 1377

Query 1379 TDVLSLLQHLGGQGSAASGSSAWRGKEAWGNSQLWGATGASSTAASLWAGDSDQHRNTPS 1438

TDVLSLLQHLGGQGSAASGSSAWRGKEAWGNSQLWGATGASSTAASLWAGDSDQHRNTPS

Sbjct 1378 TDVLSLLQHLGGQGSAASGSSAWRGKEAWGNSQLWGATGASSTAASLWAGDSDQHRNTPS 1437

Query 1439 SINSYLPGDLLGGESI 1454

SINSYLPGDLLGGESI

Sbjct 1438 SINSYLPGDLLGGESI 1453

Similar to fragile X mental retardation syndrome related protein 1 (FXMR1)

>TRINITY_DN1626_c0_g1_i4 len=2706 path=[0:0-828 2:829-1919 4:1920-2705]

TGTATATGACTTTTCAAATCTTTAACAACCCGATTTATTAATCTCCTATGAATAAATAAGTCAGAAACTATT
AAAAATAAACAAATTTAAATCAGTAAATAAGGACAGGTTATGATAAGAACAATTATTTCTACCTTATGAGC
TTGTTAGGAGTGTGATCTTCGGCATCGTAATATTCATCTGAATCACTGCTACCAGACATTTTAAATTTGAAA
ACAATTAATGAAGTATTTTATGACATTTTTAAGAAATAAGATTAAATAAATGAACGTAAGTTAATCCACAG
CCTTCTACATCTTGTATGAACACTCCTACCAATGGGAAAGGAGAGAACCAAGTTTATGGGATTCTATTGGTT
GTAGTAAAGTTATACGAGGCCCGTTGATATTTTCATAGCAATAGCAAGATGGATGACCTAGAAGTGGAAGT
ATGCGGCGAAAACGGTGCTTATTATAAGGCTTATGTCACTGATGTGTTTGAGAAAGAAGTGTCAGTTGCCTT
TGAAAATGAATGGCAAGCTGAGTCCAGGTTTACCTTTAATCTGGTACGATTACCTCCTCCTAAGGATGCCCC
TAAAACAGAATTCAAGGAGAATGAAGAGGTTGAAGTACTCTCTCGGTCGAATGACCATGAGTCATGTGGAT

GGTGGAGAGCTGTCATTAAGATGATGAAGGGAGATTTTTAGTTTGGAATACCTTGGCTGGGAAACAACT
TACACGGAAATTGTCCCTTCTGACAGGTTAAGGCATAAAAACACCAATCCTCCAATCACAGCACAAACATT
CACCAAGTTCGAAATAGAAGTTCCAGAAGAGCTGCGGGAATATGCGAAGAAGGAAAATGTTCATAAAGAG
TTCCAAAAAGCCATAAATGCTTCAATCTGTCGTTATATACCTGAAAGTGGAGTCCTCAGCTTTATTTCGAGG
TGTGAAGGAGCCAAGAAAAGAGCTACTATGCTTCAGGAAATGCATTTTCGGAATTTGCATCAAAAACTGTT
ACTCTTGAAACGAACAGAAGAAGCCCAAAAACAGCTGGAATCCACCAAGCTCCAGAACACCGGAGGGTTT
TCTGATGAATTTTCTGTCAAAGAGGATCTAATGGGCTTAGCTATTGGTGCTCATGGAGTAAATATACAGCAG
GCGAGAAAGTTGGATGGTATCACCAATATTGAGTTGGAAGAGGGTTGCACTTTCAAGATATATGGTGAAAC
TAGTGAAGCTGTTAAAAAGGCAAGAACCATGCTGGAATACAGTGAGGAGTCAATGCAAGTGGAAAGAAGT
TTAGTTGGAAAAGTTATAGGAAAAAATGGCCGTATAATTCAAGAAATAGTTGATAAAAGTGGAGTTGTTCG
GGTGAAGATTGAGGGAGACAATGAGCCATCTCCGTCGGTACCTAGAGAAGAAGGTCTAGTCCCATTCATGT
TCGTTGGTACTCTTGAGTCTATTACCAATGCTAAAATTCTCCTTGAATATCACCTTGCCCACCTCAAGGAGG
TCGATAAGCTGAGACAAAATAAAGAAGCCATTGAAAGCCAGGTTCGGAACATGCAGGGAACTAACATGGG
CTCGATGATGGGATTCCCCATACAGAGGCGCAACGAGAGAGGTTACAATTCCGATATGGAAGTTGGTGGTG
GACGAGGCAGGGGAGGTCCTCCTAGGGGACGTGGAGGAAGAGGTAGAGGTGGACCTGGTAGGCACGACTC
CAGGTACCAAGGTTCCCGACACCAAACTCCTGACACCGATGAAAGAGTTAAGGAAGGAGGAAGAGGTTAT
GGTGGAGGCGGCCGAGGTGGTGGAAATTACATGCAAAGGAGGGACAGGGGAGACAGGGGGGGCCCTCCA
AGGGCGAACAACCCTCGAGTAAGGCATGACGAAGACCAACCACCTCCACAGGATTTCGAAACAGGAGACT
CTGCTGCCATAAACAGTGCACCTGTAGAGAATTCCTCGAGTAAAGAAGCTGTTAACAGTACTGAAGGACCT
AAAGGCCCACGCAGAAACAACAGGCAGAAGAGGACTGAGAAGGTGAAGGAGCTGTACAACGGGACGACC
TAAAGAGGAAAAGATGCGGGGGAGGAGGATCTAGTCACCATGGCTCACTGCCTGTTGCTGAAACAAGGAC
ACTAACCGATATTTCCACTTCTCCCTCGTCCGCATTACTTGCTACTTTATTTGTGCCGTGCCAATGGCTACA
GGCAGTGGGCAAAGGAAGAACTAGGTTTCACCATCGCTGGCAATATCTCTTTCCTCATTTTTCTTTTCTTTTT
TCTCTCTTTTTTTCATCTCTATCTCTCGGAAACAAAAAATCTCTTTGTTATATCTAATTCTGAGTTAAACTTCA
AGCGTTTGACTAGAAACCCCTCCTCATATTTATTTGTAAGACATTGTAGATATATTGACTTTTCTTTTCTTTT
TTTCCCTGATTATGAATATCTTGCTCAGATAGGTTAATGAACAGATTTTCTTTCTGTTTTCTCTTGCTATACA
ATTATTGTGTTTGTGTTATAATGTGTTATTCGCTTAATATTTTGGCTAACACCCATTTACAAATATTGCTATC
TGCAAATAAAGGATTTAAAATCCAGAACGATACTTTTATTTTTATTTTTTGCTTTTTTTTTTTTTTATATATAT
ATATTTTTTTCTTTAACATCAAGAACTTCATTTGGAGGGGCGCTTACTGTACTTATTTCAAATTATT

Protein

RF: +2

ORF: 407 -> 2059

Length: 550 aa

>lcl|ORF2_TRINITY_DN1626_c0_g1_i4:406:2058 unnamed protein product

MDDLEVEVCGENGAYYKAYVTDVFEKEVSVAFENEWQAESRFTFNLVRLPPPKDAPKTEFKENEEVEVLSRSN
DHESCGWWRAVIKMMKGDFLVLEYLGWETTYTEIVPSDRLRHKNTNPPITAQTFTKFEIEVPEELREYAKKENV
HKEFQKAINASICRYIPESGVLSFISRCEGAKKRATMLQEMHFRNLHQKLLLLKRTEEAQKQLESTKLQNTGGFS
DEFSVKEDLMGLAIGAHGVNIQQARKLDGITNIELEEGCTFKIYGETSEAVKKARTMLEYSEESMQVERSLVGK
VIGKNGRIIQEIVDKSGVVRVKIEGDNEPSPSVPREEGLVPFMFVGTLESITNAKILLEYHLAHLKEVDKLRQNKE
AIESQVRNMQGTNMGSMMGFPIQRRNERGYNSDMEVGGGRGRGGPPRGRGGRGRGGPGRHDSRYQGSRHQT
PDTDERVKEGGRGYGGGGRGGGNYMQRRDRGDRGGPPRANNPRVRHDEDQPPPQDFETGDSAAINSAPVENS
SSKEAVNSTEGPKGPRRNNRQKRTEKVKELYNGTT

Conserved Domains

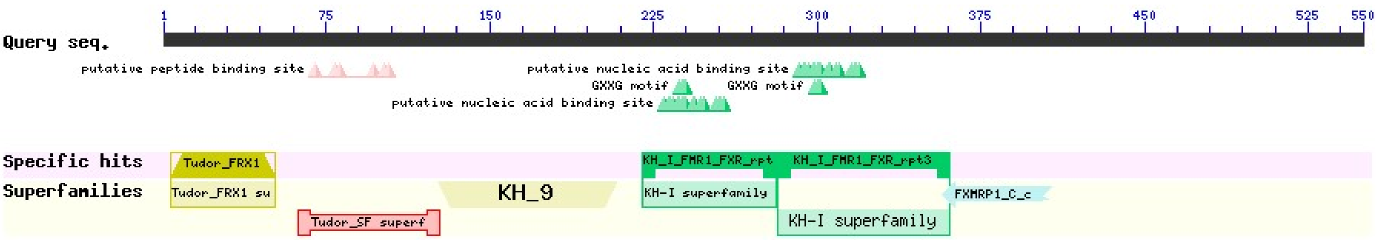

BLASTp

XP_969396 PREDICTED: fragile X mental retardation syndrome-related protein 1 isoform X2 [Tribolium castaneum]

Score:613 bits

E-value: 0.0

Query 28 MEDLAVEVCGENGALYKGYVVDVFEDSVLIHFEDEWQPDSKFPFSQVRLPPKPD-PKVEF 86

M+DL VEVCGENGA YK YV DVFE V + FE+EWQ +S+F F+ VRLPP D PK EF

Sbjct 1 MDDLEVEVCGENGAYYKAYVTDVFEKEVSVAFENEWQAESRFTFNLVRLPPPKDAPKTEF 60

Query 87 TENMEVEVYSRANHQEAYGWWKSRIKMMKGDFYVLEYVGWDTTYTEIVSDDRLRVKNSNP 146

EN EVEV SR+N E+ GWW++ IKMMKGDF VLEY+GW+TTYTEIV DRLR KN+NP

Sbjct 61 KENEEVEVLSRSNDHESCGWWRAVIKMMKGDFLVLEYLGWETTYTEIVPSDRLRHKNTNP 120

Query 147 PIDSSMFVKFEIEVPEDVREYAKIENAHKEFQNAIGASLIRYVPEKGVLVVISRNESSRR 206

PI + F KFEIEVPE++REYAK EN HKEFQ AI AS+ RY+PE GVL ISR E +++

Sbjct 121 PITAQTFTKFEIEVPEELREYAKKENVHKEFQKAINASICRYIPESGVLSFISRCEGAKK 180

Query 207 CARLVQDMHFRSLSQKVLLLKRTEEAARQLESTKLATIGGFSDEFNVREDLMGLAIGAHG 266

A ++Q+MHFR+L QK+LLLKRTEEA +QLESTKL GGFSDEF+V+EDLMGLAIGAHG

Sbjct 181 RATMLQEMHFRNLHQKLLLLKRTEEAQKQLESTKLQNTGGFSDEFSVKEDLMGLAIGAHG 240

Query 267 ANIQQARKVDGITNIELEENSCTFKIYGETDEAVKKARSMLEYSEESLQVPRALVGKVIG 326

NIQQARK+DGITNIELEE CTFKIYGET EAVKKAR+MLEYSEES+QV R+LVGKVIG

Sbjct 241 VNIQQARKLDGITNIELEE-GCTFKIYGETSEAVKKARTMLEYSEESMQVERSLVGKVIG 299

Query 327 KNGRIIQEIVDKSGVVRVKIEGDNEPQPTIPREEGQVPFVFVGTVESISNAKVLLKYHLA 386

KNGRIIQEIVDKSGVVRVKIEGDNEP P++PREEG VPF+FVGT+ESI+NAK+LL+YHLA

Sbjct 300 KNGRIIQEIVDKSGVVRVKIEGDNEPSPSVPREEGLVPFMFVGTLESITNAKILLEYHLA 359

Query 387 HLKEVEQLRQEKLEIDQQLRSIHGNALGSMQSLSMSRRNDRGYNSDMD 434

HLKEV++LRQ K I+ Q+R++ G +GSM + RRN+RGYNSDM+

Sbjct 360 HLKEVDKLRQNKEAIESQVRNMQGTNMGSMMGFPIQRRNERGYNSDME 407

Gemin 3 homolog

>TRINITY_DN55620_c0_g1_i1 len=2684 path=[0:0-2683]

TATTAATATACATTTTTAAATGATCTTGATTAAGTTAACCAATCCTACAAAGGTCCGCCATGTTTTGAAATA
AGTTACTAGGTGTTCTGTGTTGTTATTATTTGATTAGTTGGTGAAATTTTAAGAATAAGACGAAAATGGTAG
CTCACGATTTAGAAAACAAAGAGAGGACTAAAGATATAGCGATAGAAGATGGTGTTACGTTTCTACTATG
TTGTTACCGGATTTCATTTTAAATGGACTGAATTCAGCCGGTTTTAAAAAACCATCACCGATCCAACTTGCT
GCGATTCCTTTAGCAAGATGTGGTTTGGATCTCATAGTACAGGCCAAGTCAGGCACTGGCAAAACTCTTGT
GTTTGCTATCACTGCTTTGGAATCTATCAGTTTAAAGATCAATAGAGTTCAGGTCATTATTTTAGCTCCTACG
AGAGAGATAGCTTTACAAATAACAGATGTTATTAATTGTATTGGAAGTTCTTTGAAAGGTTTGAAGGTTGGT
ACATTTGTTGGTGGACGATCGACAAATTCTGATAAACCTGTAATGAAAGGATGTCACATAGCTGTCGGCTC
GCCAGGTAGAATAAGACACCTGATTGAACTGAATATTTTGGACTCTAAAGCAGTTAGATTATTTGTGCTAG
ATGAAGCTGATAAACTGTTTGAAAAAGATTACGATTATCAACATGATTTGAATTTTATTTTTAATAATTGTG
GTGAAAGTAAGCAAGTGCTTGCCCTGAGTGCCACTTATCCCCCCGAATTATTGGAATTCACAAAAAAATAC
ATGAGGGATGGTCAGCATGTTACACCTAGTGGTGGGCCTACCCCAGTACTTCTTGGTCTGAAGCAGATGGT
GTTGATATCCCGAGATCATCCTGACTTTGCCGCAAGATTGGCCATCAAAGAGAAAGATTTGATTAACATTCT
AGAAACTGTATCTTTTGATCAGTGTTGTGTTTTTCTGAACTACGCCACTAGGGCAGAGAGCATAAACTATTT
ATTGAAAAAGAAAGGTTTTGCATCTACATGTCTCACTGGGAAACAGGATATGGATACGAGAATAGAAGCTA
TTACCACATTCAAGTCTGGTGGTTGTAGAATTCTTGTTACCACTGACTTAGCAGCTAGAGGAATTGATGCTT
CTTGCATTAATTTGGTGATCAACTTAGATCTTCCATGGGATGCTGCTACTTACCTGCATAGAATGGGACGTG
CTGGCAGATATGGTTCTCATGGAATCTGTATTTCAATAATCTCAGATGGTATTGAGTTGGAAAAGTTCAGAA
GGCTTTTGGGCTCGATTGGTGGTAAATCAATGGAAGTGAATGTTATACCTCGGTTAGGCAAAGTTGACCTTT
GGACCGAAGATGTAGGTAATTTCGATAAGATCCAAGGCATTATTGAATCATCTAGTGAAGAAGAGACTTTA
GAAGAGAAATTGAAGAAAAATTTCCAGACTGCAAATGAATATCTTCAAAGTAGAGGCAAAGAGGAAAGTA
TTCATCTTTTTGAACAGGATCAATGTGATGAAGTTTCTACTAGCATTGGTGTTAGTAGTGAAGCAACCCAAA
CTATTTCCATGAAAGAATTAGAAGATGAATTGAATAATTATCTTTTAGAGACAGTTCAACAAAAACAACAA
AAAGAGATTCATCAGAAAGTATCAACTCCCCGTAAATCAACCCCTAAAAAAGTGCCCACTTATTCAGAAAT
TTTGACGAATACCCGAGGAAAGAATTCTCCAGCTAAAAAATCCCTTTCAGGTGCTTTAAGTGGCAAGTTTTT
AGCTAATGCGGTAATTAAGTCTGAAAAAGTAATTGAGAAGAAAGACCAAACTATTAGTGATAAATTATTCA
AATTAACTGAGGATGTTGCTAAACCAGATAAGTCGTTTAAGAGCGTAATTGTCCTAAGTAATAAAGATAAT
GGGACTAGCTGTACTGATTCCAACACTGATAGGACTATTGTTAATGAACCAGCAAATATAGACATAGCTGC
TAACATACCATCAAGTGATCCTATTAGGAAAATTACTAATGAAACATGCTCTAAAAGGAAACGTACCGTCA
GTTCTGCCAGTTCTTCTGAGTCTGATGATTTTGACAGCTATACATATACAGTTGATTCTTATGATGATTATGA
AACCATTGACGATGTTACCTCAGTTTCTGGAATTGATTTGAATTCCAATCATGATGAATTTGAATCTCTGTC
AGATATATCTTCTGCGGAAGAGGAGAGTGAAGTTGATTATCCATCAGAAATATTCCATGTTTTGGAATCTG
ATTATGAGGAACCCGAACAGTCTCCTCCAACTGATTGGTTTGCAGTTATTTCTAAAGAAATTCAAAATTATG

TTGTATATTCTAGGCAGCACATGAATGCTCACCGTAGCTGTGTCAGCCATACGGGTTAAGTAACTACATTTT
TCTAATTATCTAATGTTTTAGAAGTTAAATTTAAAGACTGAATTGTTATTACTCTTGTAATATTCTGTAAATT
GCTTGTTTTGGTATTTTTAAATGTTATGCCTATGTTTTGTTATTGTATTTATATTGTTGTTTTCGATTACATTA
ATCTTTTGTAACTTATTTGCATTTTTGTTTTATAAATGTAATTATACTATTGTGATATAAATTACCAAATAAT
TTTGATTAATGTTAATACAAATACAGTCTGG

Protein

RF: +3

ORF: 138 -> 2420

Length: 760 aa

>lcl|ORF6_TRINITY_DN55620_c0_g1_i1:137:2419 unnamed protein product

MVAHDLENKERTKDIAIEDGVTFSTMLLPDFILNGLNSAGFKKPSPIQLAAIPLARCGLDLIVQAKSGTGKTLVFA
ITALESISLKINRVQVIILAPTREIALQITDVINCIGSSLKGLKVGTFVGGRSTNSDKPVMKGCHIAVGSPGRIRHLIE
LNILDSKAVRLFVLDEADKLFEKDYDYQHDLNFIFNNCGESKQVLALSATYPPELLEFTKKYMRDGQHVTPSGG
PTPVLLGLKQMVLISRDHPDFAARLAIKEKDLINILETVSFDQCCVFLNYATRAESINYLLKKKGFASTCLTGKQD
MDTRIEAITTFKSGGCRILVTTDLAARGIDASCINLVINLDLPWDAATYLHRMGRAGRYGSHGICISIISDGIELEK
FRRLLGSIGGKSMEVNVIPRLGKVDLWTEDVGNFDKIQGIIESSSEEETLEEKLKKNFQTANEYLQSRGKEESIHL
FEQDQCDEVSTSIGVSSEATQTISMKELEDELNNYLLETVQQKQQKEIHQKVSTPRKSTPKKVPTYSEILTNTRGK
NSPAKKSLSGALSGKFLANAVIKSEKVIEKKDQTISDKLFKLTEDVAKPDKSFKSVIVLSNKDNGTSCTDSNTDR
TIVNEPANIDIAANIPSSDPIRKITNETCSKRKRTVSSASSSESDDFDSYTYTVDSYDDYETIDDVTSVSGIDLNSNH
DEFESLSDISSAEEESEVDYPSEIFHVLESDYEEPEQSPPTDWFAVISKEIQNYVVVYSRQHMNAHRSCVSHTG

Conserved Domains

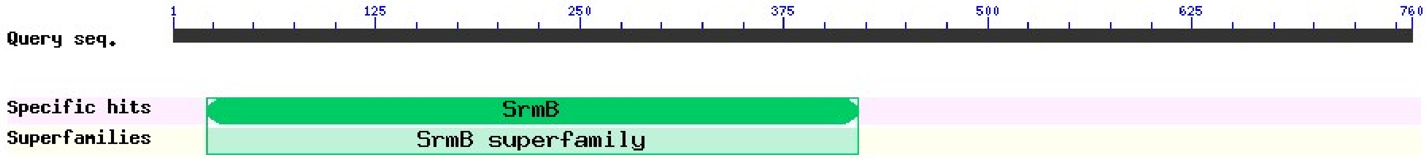

BLASTp

EFA00789 ATP-dependent RNA helicase WM6-like Protein [Tribolium castaneum]Score:1321
Score : 427 bits

E-value: 5e-144

Query 5 IAHDLDAKERTKDVILDENISFASLLLPDDIKQGLSVSGFKKPSPIQFKAIPLGRCGFDL 64

+AHDL+ KERTKD+ +++ ++F+++LLPD I GL+ +GFKKPSPIQ AIPL RCG DL

Sbjct 2 VAHDLENKERTKDIAIEDGVTFSTMLLPDFILNGLNSAGFKKPSPIQLAAIPLARCGLDL 61

```
Query  65   IVKSKSGTGKTLVFSTIALETVNTAKDHLQVLILVPTREIAVQIEDVLRSVGCHVNGLKI  124
            IV++KSGTGKTLVF+  ALE+++   + +QV+IL PTREIA+QI DV+  +G  + GLK+
Sbjct  62   IVQAKSGTGKTLVFAITALESISLKINRVQVIILAPTREIALQITDVINCIGSSLKGLKV  121

Query  125  ESFIGGRPLEDDLKKSSKCHIAVGAPGRVKHLLKMGALTTNLVKLFVLDEADKLMEE--S  182
             +F+GGR    D      CHIAVG+PGR++HL+++   L +  V+LFVLDEADKL E+
Sbjct  122  GTFVGGRSTNSDKPVMKGCHIAVGSPGRIRHLIELNILDSKAVRLFVLDEADKLFEKDYD  181

Query  183  FQSDINEIYNSLPPRKQMIVSSATYPQELDTFLANYMQSPTHVT-SENETPLLLGLKQFA  241
            +Q D+N I+N+    KQ++  SATYP EL  F   YM+   HVT S   TP+LLGLKQ
Sbjct  182  YQHDLNFIFNNCGESKQVLALSATYPPELLEFTKKYMRDGQHVTPSGGPTPVLLGLKQMV  241

Query  242  AMLRPGLNSVQQMKIKNDLLITILTKVSFVQCLVFTNYQSRTETVSNYLNQKGWDSVFIS  301
             + R   +   ++ IK   LI IL  VSF QC VF NY +R E+++  L +KG+ S  ++
Sbjct  242  LISRDHPDFAARLAIKEKDLINILETVSFDQCCVFLNYATRAESINYLLKKKGFASTCLT  301

Query  302  AAQKQTERLEAIDNLKKFKNRILLSTDLTSRGIDAPNVDLVINYDLPCDAVTYLHRMGRA  361
              Q    R+EAI   K    RIL++TDL +RGIDA  ++LVIN DLP DA TYLHRMGRA
Sbjct  302  GKQDMDTRIEAITTFKSGGCRILVTTDLAARGIDASCINLVINLDLPWDAATYLHRMGRA  361

Query  362  GRYGSGGLCINFVSEGPEVTKLQHILGAIGG-NLSIAKLPPLEGVDLWQVDLKTLEQIRG  420
            GRYGS G+CI+ +S+G E+ K + +LG+IGG ++ +  +P L  VDLW  D+   ++I+G
Sbjct  362  GRYGSHGICISIISDGIELEKFRRLLGSIGGKSMEVNVIPRLGKVDLWTEDVGNFDKIQG  421

Query  421  VV  422
            ++
Sbjct  422  II  423
```

p68 Helicase

>TRINITY_DN10378_c0_g1_i5 len=3350 path=[1:0-1390 2:1391-1391 4:1392-1392 6:1393-2541 8:2542-2685 10:2686-3349]

CCCGTACTTGTTAACTAGAGTTGTTTTTCGTGTTAAGTGGAAGCATAATTTAGAAGTTAGGAAATTTGCCGG
ACTGAGAGAATATCGGTATATTTTCGCTCCCAAACCTTACAGTATTAAGATTACTTTAGTCAGAAATGGGTG
GACGTGACGGCAGAAGAGGAGGTGCCAGAGGAGGCAGGGGTGGAAGAGGTGCTGGAAGTGGAGGAGGTC
GTGGTGGCAGGGATTTTGGCCGTGGTGGATTTGATCGCAGAGATGGTTTTGGAAGTTTAAAAGGAAAACAG
CCTGGTGGACGCCTAAGAAAGCCTAGTTGGAATATGGCAAATCTAGAGCCATTCAAGAAAGATTTTTATGT
TCCTCATCCAAATGTCTTAAACAGGACAGAAGATGATATTGAAAAATTTAGAGAAAGCAAATCAATTACCA
TTAAAGGGGACAGAATCCCACCACCAATAATGCGATTTGAAGATGTTAATTTCCTGACTATGTTATGAATG
AAGTGAAAAAACAGGGCTATGATGAACCTACACCAATTCAGGCCCAAGGCTGGCCAATAGCATTAAGTGG
GAGGGATATGGTTGGAATTGCTCAGACTGGTTCGGGTAAAACTTTAGCATATATTCTTCCTGCTATAGTCCA
TATAAATAATCAACCGAGGTTAAGACCTAGAGACGGTCCTATTGCTCTTATTTTAGCCCCTACTCGTGAGTT
GGCACAGCAAATTCAGCAAGTTGCCAATGACTTTGGATCTCATACATCAGTTCGAAATACTTGTATTTTTGG
TGGTGCGCCTAAAGGAGGGCAGGCGAGAGACTTGGAAAGAGGTGTTGAAATTGTCATTGCTACTCCTGGA
AGGCTTATAGATTTTCTTGAGAAAAATACAACTAATTTGAAAAGATGTACATACTTAGTATTAGACGAAGC
AGATAGAATGTTGGATATGGGTTTTGAACCTCAAATTAGAAAAATTATTGAACAAATACGTCCTGATAGAC
AAGTATTGATGTGGTCTGCTACTTGGCCTAAGGAAGTTCGTAATTTAGCTGAAGAATTTCTTCATAATTATA
TCCAATTAAATATTGGATCATTAGAATTGTCTGCTAATCATAATATTCAACAATATGTTGATATTTGTGATG
AATATGAAAAACCTGACAAACTTTGTGACCTCTTGACAGACATATGTAGAGAAGCAGAATCTAAAACTATC
ATTTTCACAGAGACTAAGCGAAGCGTTGATGAAATTGTTAGAACAATAAATCGAAGGGGATGGAAAGCCA
GTGGAATACATGGTGACAAATCTCAACAGGACCGTGATATGGTTTTAAATGATTTTCGCACTGGCAGGGTT
AACATCCTCGTAGCAACAGATGTTGCTGCCCGAGGTTTGGATGTTGAAGATGTAAAATTTGTTGTCAATTAT
GACTATCCTTCTTCATCGGAGGATTATGTCCATCGAATAGGACGTACTGGAAGATCTCAGAGAACAGGAAC
GTCATATACATTTTTTACTACTGGAAATGCTCGTCAAGCTAAGGATCTTATATCTGTTTTACAAGAGGCCAA
GCAGACTTTAAACCCTCGCCTTCTAGAGCTTTGTGAAATGGCTAAGTGTGGAGTTTTTGGAAAACCAAGAA
GCACAAGATTTGCTGGTAAAGAGAGGGGCAGTAAAGGTGGAAGAGGAGGAAGGGGTGGTAGAGGAGGCA
GTGAAAGGAGAGGGCGACCCGAAAGGAGAGGACCTAGTAGTGGATCTGAAAGGAGGGGTAGTGATCGAA
GCGGTGGTGGTAGGGGAGATCGTAGCTCTGAGAGGAGAGGTGGAGGAAGTAGTGACAGGTGGGGAAGAG
ATTCGGCTCCGCCATCAGGCAGAGGTGGTGGTTTTGGAAATAGAATCGGTTCTGGTGGAGGATTCGACAGT
GGTGGCCGTGGTGCTCTTGGCGGCAGCGCAGGATTTGGAGAAAGAGATCCTCCAAGGAACAGTGGTTTCGG
CAATGATGGTGGCAGAGCAGGTGGTTTTAGACCGAAACCTCCTTCTTTAATGCAGGCTGAAACTTTTGGTG
ATAGGAATGGTCCAAGAGGTGGAAACTACTCAGAAGGTTTTGGAAGCGATAGTGGTAGGGGTTTTGGAGA
TGCTGGAGGTGGTAGAGGATTCGGTGGTGACAGCAATCGAGGTGGGTTTGCCACAGAAAATGGTGGTCGG
AGAGGATTTGGTGGGGAAAGTAGAGGAGGATTCGGAGGAGATAGTGGAAGAGGAGGATTTGGTGGAGAT
GAAGATGATGGATTTGGAAGAGGAAGGGATTCAAGTAGCTCAAAAATAGGTGGAAGGGGTGGAGGTAGTG
GAGCAGGAGGTAGGGGTGGTATGTCCAGAGGAATGAGTGGCAGTAGTGGAGGAGCCCCTCCAAGAGGAG
GAGGGCGAGGAGGTGGTGGGCTAGATAGGGGCGGCAGGGGTGCTCCTGCAGGCGCTGCTGGCAGGAACAG
TTACAGCTCGGCTCCAGCTCCCACTTCTAGGGGGACCTACGGTCAGGACAGGCTTCCTAGTGGAGGTTATG
AAACCAGGCAGGCGAATAGCAACAATCTTGGATATGACCGGCGAAGTGATCGAAGGTCTGACTGGGCCTC
AGAGAGTCCAGTGCCAGAAAAAAGATCTAGATTTCTGACAATACTTCGAGTACCGCAGCTCCGCCCCCAC
CCCCTCTCATGAGTACTACCAGCGCTGTTACAACCAAGGCAGCTCAAATGCCTGCCAACTTCAGTCAGCCTC
CACCAAATTACAGTAAGCCTCCTCCCAACTATCACACTGCTGTTCCTCAACCTGCACAGGCTTATCAGGCAG
CTCAACAGCAACCTACTAACATGATGCAGTACCCCCAGCACATGTATCAGCAACAAAACTACATGATGTAT
CAGCAAATGTACCAGCATCAAATGAATGGGTACCAGCAGCAACAACAGACTCAGCAGCAGACCTACCAAC
AGCAACCACCACTTCCAAAATGATGAAAAAACTTCATTCCATAAAAGCTTAACTGTCATTCGAATATATAC
ATATATATATAATTTTTTTATATAATTATACATATTTATATATGTGTATAAACCTTTGTGATTCTATTGGATG
TATTGCACCGAGTAATTTAACAAGTTTATTGTGATTTTATAATTTTAGCAAATGAGATGTTATGTTTCCTTTA
TTATTTTTTTCTTTGTTTATTTGTTTTTAATTTATTTTACTAGGAGAACATTAAAATATTGTGACAACATTTAT
TTTAATAAGTTATTTTCCCTAATTTTAATTTTTTTTTTTTTTTTTTTTTTTTTTTTTAAATAATATTTATATCGTA
ATAGAGTAGT

Protein

RF: +3

ORF: 138 -> 2996

Length: 952 aa

lcl|ORF6

MGGRDGRRGGARGGRGGRGAGSGGGRGGRDFGRGGFDRRDGFGSLKGKQPGGRLRKPSWNMANLEPFKKDF
YVPHPNVLNRTEDDIEKFRESKSITIKGDRIPPPIMRFEDVNFPDYVMNEVKKQGYDEPTPIQAQGWPIALSGRD
MVGIAQTGSGKTLAYILPAIVHINNQPRLRPRDGPIALILAPTRELAQQIQQVANDFGSHTSVRNTCIFGGAPKGG
QARDLERGVEIVIATPGRLIDFLEKNTTNLKRCTYLVLDEADRMLDMGFEPQIRKIIEQIRPDRQVLMWSATWPK
EVRNLAEEFLHNYIQLNIGSLELSANHNIQQYVDICDEYEKPDKLCDLLTDICREAESKTIIFTETKRSVDEIVRTIN
RRGWKASGIHGDKSQQDRDMVLNDFRTGRVNILVATDVAARGLDVEDVKFVVNYDYPSSSEDYVHRIGRTGR
SQRTGTSYTFFTTGNARQAKDLISVLQEAKQTLNPRLLELCEMAKCGVFGKPRSTRFAGKERGSKGGRGGRGG
RGGSERRGRPERRGPSSGSERRGSDRSGGGRGDRSSERRGGGSSDRWGRDSAPPSGRGGGFGNRIGSGGGFDSG
GRGALGGSAGFGERDPPRNSGFGNDGGRAGGFRPKPPSLMQAETFGDRNGPRGGNYSEGFGSDSGRGFGDAGG
GRGFGGDSNRGGFATENGGRRGFGGESRGGFGGDSGRGGFGGDEDDGFGRGRDSSSSKIGGRGGGSGAGGRG
GMSRGMSGSSGGAPPRGGGRGGGGLDRGGRGAPAGAAGRNSYSSAPAPTSRGTYGQDRLPSGGYETRQANSN
NLGYDRRSDRRSDWASESPVPEKRSRFSDNTSSTAAPPPPPLMSTTSAVTTKAAQMPANFSQPPPNYSKPPPNYH
TAVPQPAQAYQAAQQQPTNMMQYPQHMYQQQNYMMYQQMYQHQMNGYQQQQQTQQQTYQQQPPLPK

Conserved Domains

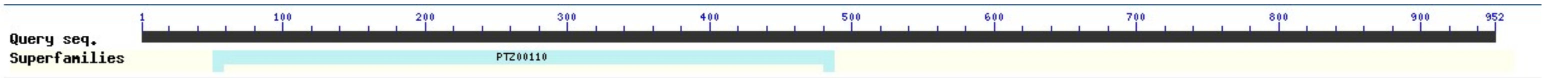

BLASTp

XP_024217656.1 ATP-dependent RNA helicase dbp2-like isoform X2 [Halyomorpha halys]

Score:1302 bits

E-value: 0.0

Query 44 SLKGKQPGGRLRKPSWNMANLEPFKKDFYVPHPNVLNRTEDDIEKFRESKSITIKGDRIP 103
SLKGKQPGGRLRKPSWNM NLEPFKKDFYVPHPNVLNRTEDDIEKFRESKSITIKGDRIP
Sbjct 44 SLKGKQPGGRLRKPSWNMVNLEPFKKDFYVPHPNVLNRTEDDIEKFRESKSITIKGDRIP 103

Query 104 PPIMRFEDVNFPDYVMNEVKKQGYDEPTPIQAQGWPIALSGRDMVGIAQTGSGKTLAYIL 163
PPIM+FEDVNFPDYVMNEVKKQGYDEPTPIQAQGWPIALSGRDMVGIAQTGSGKTLAYIL
Sbjct 104 PPIMQFEDVNFPDYVMNEVKKQGYDEPTPIQAQGWPIALSGRDMVGIAQTGSGKTLAYIL 163

Query 164 PAIVHINNQPRLRPRDGPIALILAPTRELAQQIQQVANDFGSHTSVRNTCIFGGAPKGGQ 223
PAIVHINNQPRLRPRDGPIALILAPTRELAQQIQQVANDFGSHTSVRNTCIFGGAPKGGQ
Sbjct 164 PAIVHINNQPRLRPRDGPIALILAPTRELAQQIQQVANDFGSHTSVRNTCIFGGAPKGGQ 223

Query 224 ARDLERGVEIVIATPGRLIDFLEKNTTNLKRCTYLVLDEADRMLDMGFEPQIRKIIEQIR 283
ARDLERGVEIVIATPGRLIDFLEKNTTNLKRCTYLVLDEADRMLDMGFEPQIRKIIEQIR

```
Sbjct  224  ARDLERGVEIVIATPGRLIDFLEKNTTNLKRCTYLVLDEADRMLDMGFEPQIRKIIEQIR  283

Query  284  PDRQVLMWSATWPKEVRNLAEEFLHNYIQLNIGSLELSANHNIQQYVDICDEYEKPDKLC  343
            PDRQVLMWSATWPKEVRNLAEEFLHNYIQLNIGSLELSANHNIQQYVDICDEYEKPDKLC
Sbjct  284  PDRQVLMWSATWPKEVRNLAEEFLHNYIQLNIGSLELSANHNIQQYVDICDEYEKPDKLC  343

Query  344  DLLTDICREAESKTIIFTETKRSVDEIVRTINRRGWKASGIHGDKSQQDRDMVLNDFRTG  403
            DLLTDICRE+ESKTIIFTETKRSVDEIVRTINRRGWKASGIHGDKSQQDRDMVLNDFRTG
Sbjct  344  DLLTDICRESESKTIIFTETKRSVDEIVRTINRRGWKASGIHGDKSQQDRDMVLNDFRTG  403

Query  404  RVNILVATDVAARGLDVEDVKFVVNYDYPSSSEDYVHRIGRTGRSQRTGTSYTFFTTGNA  463
            RVNILVATDVAARGLDVEDVKFVVNYDYPSSSEDYVHRIGRTGRSQRTGTSYTFFTTGNA
Sbjct  404  RVNILVATDVAARGLDVEDVKFVVNYDYPSSSEDYVHRIGRTGRSQRTGTSYTFFTTGNA  463

Query  464  RQAKDLISVLQEAKQTLNPRLLELCEMAKCGVFGKPRSTRFAGKERGSKGGRGGGRGGRGG  523
            RQAKDLISVLQEAKQTLNPRLLELCEMAKCGVFGKPRSTRFAGKERGSKGGRG  GGRGG
Sbjct  464  RQAKDLISVLQEAKQTLNPRLLELCEMAKCGVFGKPRSTRFAGKERGSKGGRGRGGGRGG  523

Query  524  SERRGRPERRGPSSGSERRGSDRSGGGRGDRSSERRGGGSSDRWGRDSAPPSGRGGGFGN  583
            SERRGR   R   SGSERRGSDRSGGGRGDRSSERRGGGSSDRWGRDSAPPSGRGGGFGN
Sbjct  524  SERRGR-PERRGPSGSERRGSDRSGGGRGDRSSERRGGGSSDRWGRDSAPPSGRGGGFGN  582

Query  584  RI-GSGGGFDSGGRGALGGSAGFGERDPPRNSGFGND-GGRAGGFRPKPPSLMQAETFGD  641
            R+  GG   G  G     GFGER+P RNSGF ND GGR GGFR KPP LM  ETF +
Sbjct  583  RMGSGGGFDSGGRSGLGNSGGGFGEREPQRNSGFANDAGGRGGGFRMKPPGLMGDETFSE  642

Query  642  RNGPRGGNYSEG---FGSDSGRGFGDAGGGRGFGGDSNRGGFATENGGRRGFGGESRGGF  698
            RNGPRGGNY+EG   FGSD+GRGFG++GGGRGFG D+NRGGFATENG R    G    G
Sbjct  643  RNGPRGGNYTEGGVAFGSDNGRGFGNSGGGRGFGSDNNRGGFATENGRRG--FGGESRGG  700

Query  699  GGDSGRGGFGGDEDDGFGRGRDSSSSKIGGRGGGSGAGGRGGMSRGMSGSSGGAPPRGGG  758
             G  G  G  GD+D+GFGRGRDSS+S+IGGRGG SGAGGRGG      G+  GAPPRGGG
Sbjct  701  FGGEGGRGGFGDDDEGFGRGRDSSNSRIGGRGGSSGAGGRGGRGMSSGGNGSGAPPRGGG  760

Query  759  RGGGGLDRGGRGAPAGAAGRNS-----YSSAPAPTSRGTYGQDRLPS----GGYETRQAN  809
            RGGGGLDRGGRGAP G+AGR +     Y S PAP+SRGTYGQDRL S    GGYETRQA+
Sbjct  761  RGGGGLDRGGRGAPGGSAGRGTSNRSGYGSGPAPSSRGTYGQDRLSSGGTAGGYETRQAD  820

Query  810  SNNLGYDRRSDRRSDWASESPVPEKRSRFSDNTSSTAAPPPPPLMS-TTSAVTTKAAQMP  868
             N+LGY+RRSDRRSDWASE+PVPEKRSRFSDNTSS AAPPPPPLMS TT+ V+TK+AQMP
Sbjct  821  GNSLGYERRSDRRSDWASENPVPEKRSRFSDNTSSAAAPPPPPLMSATTNVVSTKSAQMP  880

Query  869  ANFSQPPPNYSKPPPNYHTAVPQPAQAYQAAQQQPTNMMQYPQHMYQQQNYMMYQQMYQH  928
            ANFSQPPPNYSKPPPNYHTA PQ  QAYQA QQQ TNMMQYPQHMYQQQNY+MYQQMYQH
```

Sbjct 881 ANFSQPPPNYSKPPPNYHTAAPQVTQAYQAPQQQTTNMMQYPQHMYQQQNYLMYQQMYQH 940

Query 929 QMNGYQQQQTQQQTYQQQPPLPK 952

QMNGYQQQQ QQ QQQPPLPK

Sbjct 941 QMNGYQQQQAQQQTY-QQQPPLPK 963

Uptake genes

Scavenger

>TRINITY_DN11492_c2_g2_i1 len=3020 path=[0:0-3019]

AAGAGTTAGTCGGCCGTGAGCTGGTGATCTCTCGACTTCTCGGTGGATCAGTGACCCGGGTGTTCTGTGATC
GCTTCTCCAACTTCTTTTCTTCAACTTTAAACTTCACTTCTTCCTCTTTGCTTTCGGGCATCGAACCCACCGCC
ATGCACAACAAGAGGAAGCTTTGTGACAAGCTCACCAGCAGTTTTCTCAGGAAGTGGTGGATAGTGATCGT
TGTGTCACTGATTCTAATATTCGCTGGAATCTTCATAGCTATATTTTTTAGCAATATTGTTAACCTAGTCATA
GATAAGGAAATCACCCTCCGAGAAGGCGGTCAAGTGTTCGACATGTGGAGGAAGCCCCCGGTGGAACCAG
TGTTCAGGGTATACGTCTACAACGTCACCAACGCCGATGACTTCCTTAACCGAGGGGACAAGCCCATTCTT
GATGAGCTAGGTCCATTCGTCTACGTTGAAACCTGGGAAAAAGTGAATTTAACATTCAACGACAATGGAAC
TGTTACATTTCAACAACAGAAAATTTATAAGTTTGATCCTGATCAGTCCGTGGGTGATGAGAGTGATATGGT
CGTCGTTCCTAATATTCCTATGTTGAGTGCCACTTCGCAGAGTAAACATGCAGCTAGATTTCTCAGGCTGGC
AATGGCTTCCATTATGGATATTTTAAAAATAAAACCTTTTGTGGAAGTATCAGTCGGCCAACTGCTTTGGGG
ATATGAGGATCCTCTTTTGAAATTAGCAAAGGATGTAGTGCCTAAAGAACAGAAGCTTCCATATGAAGAAT
TTGGACTTTTGTATGGGAAAAATGGAACTTCTAAAGATATAGTGACTGTATTCACTGGTGCAAATGACATTA
CAAAATACGGTCTTGTTGATCGATTCAACGGCATGTCTCATTTGCCACATTGGAAAAGCGAACATTGTAAC
AGCCTTTTAGGAAGTGATGGATCAATCTTTCCACCCCACATCACCAAAAATACTACACTTTATGTATTCGAC
AAGGATCTCTGTAGACTACTTCCTCTTAAATTCAAGGAAGAAGTTGACACTCCAGGAAATGTTCTCGGGTA
CAGATTCACCCCACCTGATAATGTATTTGCTGATGTTGAGAAAAACCCAGAAAATGAATGTTTCTGCCCATC
TGGACCTCCTTGTGCACCAGATGGATTGTTCAACGTTTCTCTTTGCCAATATGATTCTCCAATCATGCTTTCA
TTCCCTCATTTCTATCTGGCGGATCCAAAACTGAGGACTGCTGTCGAAGGAATTTCTGAACCTGATCCAGAA
AAGCATGGATTTTGGCTTGATGTTCAACCAACAATGGGAGCAGGTTTAAGAGCACAAGCAAGAATTCAAAT
CAATTTGGCTGTAAGTCAAGTTGTAGACATTAAGCAAGTGGCGACGTTCCCAGACATAGTTTTTCCCATCAT
GTGGTTTGAAGATGGAATCAGCGGTCTGCCTGAGAGTGTAACCAAGCTTATGAACCTCGCTACAACACTTC
CACCCGTTTTAAAAACGGTTCTTCTTTATATTTCTTTATCTGTGGTGTCTTTTTCTTCATATTGTCAGCATTC
TGCCTCGTTAGGAACTCCCAACGGCAAGAGACCCTCAGTCTGGAAGGAACAGCTCACTACGCAGCCAACG
ATGAAGCAAACAAGAAAGCAAAGCAACAAGCCAACGGCCAGACCAACGGACACAAATATCCAGACACTA
AGACGAATCCAGCTTTTGTTGGTGATCAATCATAACATATAATTTCGAATTTTTTAATTTTATTTTTGGGAGG
ACAGACTTTACCGCAGTACAAATTTAAACTGGAAAGCGGTAATGGACATTTTAGCAGCTGATAAATTAAGT
AGTGAACTTCAATTAAGATCATTGAATGAATATCTATGACTAGCTGCCTAAGCATTGCTCAATTTCTTTTTA
TTTTTTTATTGTTGTTTTAATTGTTGAACATCGAAAAAATATATATTTTTGAATTTAAATAATCACCTGTTTT
GTGATATGAATATATTTCTTTTTGTATGCCGTTTATCTAAAATAGGCTTTTCAAATAATTTGTATTTCTTGTT
GTTTGTAGTGTATGTTTATCTAAACATTCATTATAAACATTTGTAACATATCCGTCCAGTCAAGAAGTACTT
AGTATTTTTTTACATAAATTGTGTATATAAATTGTATAGTGGTCATTATATTTCTGCATTAAGATTGTTTATG
AGGATTTAGTTGTAGAAATATTCAAAGCCTTGTGCCTGATGTTAAATATTAGATATTATTTGAGATTTTTGT
ATAAAAATGGTTAAAATTTGTGATGAGGTGCATTATTTTACCAACTTATCGAGAATTTATTGTTGTTAGTTTT
TCAGAAATATTTTTATTATTTGTTCATTCTTTTTTTTTTTTTTTTTTAATAAATATATTATTTTATGTATTATAG
AGAGGATGTGGAAATCAGGCATTTATTAAAAGATCCCTCACATTAAATAAGAAATGTAAGGGTTAAATATT
TATTACTTATTTTATTGTATAAACTGTATAAAATCATGTGAAGTGTATTAAGTGTGATATTTAGTAATTTGTA
TTTGCTTCCTGTTATTATTTACTACACACCAGTGCCATCATTCAAGTCCATGTCACTAGTTAGTGATGAACTT
GAACTCTCTATGAGAGTTGAATTTTCTCTATTTTTCTTTTAAATCTATTGATAATAATATAATGTGCAAATTA
TTCAAAAGGCATTTGATAGTTCTGGTGTCTTGTAAATAAACAGGGGAGGAAAATGATGATTACTCATTTCTT
GGAGAAAGCTGAAATAAGAGTGAATTTAGTAAAATGTACAATACATTTTATATTCAAAGAAACGATGTATT

GTTTTTTGAAATGATGTTTTTCAGACATTCCATTTATTGTAATTGAATAAATATAATTTTAAATCAAAGTTTT
ACTAATCTATAAATGAATTTAAACCTCCATTTTCTTATTATCCACAAATATCTTGTTAACAATGATCC

Protein

RF: +3

ORF: 147 -> 1754

Length: 535 aa

>lcl|ORF9

MHNKRKLCDKLTSSFLRKWWIVIVVSLILIFAGIFIAIFFSNIVNLVIDKEITLREGGQVFDMWRKPPVEPVFRVYV
YNVTNADDFLNRGDKPILDELGPFVYVETWEKVNLTFNDNGTVTFQQQKIYKFDPDQSVGDESDMVVVPNIPM
LSATSQSKHAARFLRLAMASIMDILKIKPFVEVSVGQLLWGYEDPLLKLAKDVVPKEQKLPYEEFGLLYGKNGT
SKDIVTVFTGANDITKYGLVDRFNGMSHLPHWKSEHCNSLLGSDGSIFPPHITKNTTLYVFDKDLCRLLPLKFKE
EVDTPGNVLGYRFTPPDNVFADVEKNPENECFCPSGPPCAPDGLFNVSLCQYDSPIMLSFPHFYLADPKLRTAVE
GISEPDPEKHGFWLDVQPTMGAGLRAQARIQINLAVSQVVDIKQVATFPDIVFPIMWFEDGISGLPESVTKLMNL
ATTLPPVLKTVLLYIFFICGVFFFILSAFCLVRNSQRQETLSLEGTAHYAANDEANKKAKQQANGQTNGHKYPDT
KTNPAFVGDQS

Conserved Domains

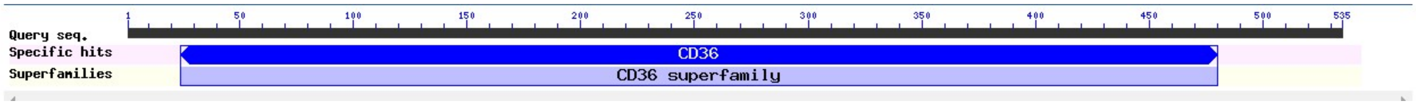

BLASTp

XP_024218066.1 scavenger receptor class B member 1, partial [*Halyomorpha halys*]

Score:1026 bits

E-value: 0.0

Query 1 VVIIVSLILIFVGIFIAIFFSSIVNLVIDKEITLREGGQVFDMWRKPPVEPIFRVYVYNV 60

+VI+VSLILIF GIFIAIFFS+IVNLVIDKEITLREGGQVFDMWRKPPVEP+FRVYVYNV

Sbjct 21 IVIVVSLILIFAGIFIAIFFSNIVNLVIDKEITLREGGQVFDMWRKPPVEPVFRVYVYNV 80

Query 61 TNADDFLNRGDKPILDELGPFVYVETWEKVNLTFNDNGTVTFQQQKIYKFDPDQSVGDES 120

TNADDFLNRGDKPILDELGPFVYVETWEKVNLTFNDNGTVTFQQQKIYKFDPDQSVGDES

Sbjct 81 TNADDFLNRGDKPILDELGPFVYVETWEKVNLTFNDNGTVTFQQQKIYKFDPDQSVGDES 140

Query 121 DMVVVPNIPMLSATSQSKHAARFLRLAMASIMDILKIKPFVEVSVGQLLWGYEDPLLKLA 180

DMVVVPNIPMLSATSQSKHAARFLRLAMASIMDILKIKPFVEVSVGQLLWGYEDPLLKLA

Sbjct 141 DMVVVPNIPMLSATSQSKHAARFLRLAMASIMDILKIKPFVEVSVGQLLWGYEDPLLKLA 200

Query 181 KDVVPKEQKLPYEEFGLLYGKNGTSKDIMTVFTGANDITKYGLVDRFNGMSHLPHWKSEE 240

KDVVPKEQKLPYEEFGLLYGKNGTSKDI+TVFTGANDITKYGLVDRFNGMSHLPHWKSE

Sbjct 201 KDVVPKEQKLPYEEFGLLYGKNGTSKDIVTVFTGANDITKYGLVDRFNGMSHLPHWKSEH 260

Query 241 CNRLAGSDGSIFPPHITKNTTLYVFDKDLCRLLPLQFKKEVNTPGDVLGYRFSPPENVFA 300

CN L GSDGSIFPPHITKNTTLYVFDKDLCRLLPL+FK+EV+TPG+VLGYRF+PP+NVFA

Sbjct 261 CNSLLGSDGSIFPPHITKNTTLYVFDKDLCRLLPLKFKEEVDTPGNVLGYRFTPPDNVFA 320

Query 301 EIEKNPDNECFCPSGPPCAPHGLFNVSLCQYDSPVMLSFPHFYLADPKLRTAVEGISEPD 360

++EKNP+NECFCPSGPPCAP GLFNVSLCQYDSP+MLSFPHFYLADPKLRTAVEGISEPD

Sbjct 321 DVEKNPENECFCPSGPPCAPDGLFNVSLCQYDSPIMLSFPHFYLADPKLRTAVEGISEPD 380

Query 361 PEKHGFWLDVQPTMGAGLRAQARIQINLAVSQVVDIKQVATFPDIVFPIMWFEDGISGLP 420

PEKHGFWLDVQPTMGAGLRAQARIQINLAVSQVVDIKQVATFPDIVFPIMWFEDGISGLP

Sbjct 381 PEKHGFWLDVQPTMGAGLRAQARIQINLAVSQVVDIKQVATFPDIVFPIMWFEDGISGLP 440

Query 421 ESVTKLMNLATTVPPVLKMVLLYIFFICGFFFFILSAFCLVRNSQRQETLSLEGTAHYAA 480

ESVTKLMNLATT+PPVLK VLLYIFFICG FFFILSAFCLVRNSQRQETLSLEGTAHYAA

Sbjct 441 ESVTKLMNLATTLPPVLKTVLLYIFFICGVFFFILSAFCLVRNSQRQETLSLEGTAHYAA 500

Query 481 NDEANKKAKQQANGQTNGHKYPDTKTNPAFVGDQS 515

NDEANKKAKQQANGQTNGHKYPDTKTNPAFVGDQS

Sbjct 501 NDEANKKAKQQANGQTNGHKYPDTKTNPAFVGDQS 535

CG4966 = orthologous to the Hermansky-Pudlak Syndrome4

>TRINITY_DN9038_c0_g1_i1 len=1583 path=[0:0-1582]

AATTTTGTAACCTCTTCCAAACAAATTAATATGCACTCCTCTATGTTATGTAATCTGGTGCTCTTTTGACTTA
AATGGGTTCTTGTACATATTATTGAATATTTCTGTGATGGCAAAGGAATTATTGATAGTTTTATATATGATT
GCGAATCCTGTAAGAAGGAAGAAGATGACCCACAAGACGCAATCATTTATTTTTACCCTACTTGGGTCAAC
AACGAACAGAGACATGCTCTGTGCAGTCAGTTAATGGGCGTCACTCAGTTTTGTGCATCATCCTTCTCTTTA
CCGAACATCATCTCTCTGCAAAGTGGAAAGTTCGCATTGAAAAAGTTGGGCAGATTTGCTTTGTGCGTGGG
GACAGATAGAAACATTCCAGATTTAGTATTGGAAACACGTGCGAGCATATTATATAAGTTGTTGAGGCTGT
TTCATTTCAGTATTGAAAATTTACAAGAATCGTCAGGTGAAGAAGATATTTCCGATAAATTGGCCGAACTCC
TCCAAGTATACTTGCCCATATTGCAATATGCCACTAACACATTCGGAAACATTCCGGTATTAAAAATCCCTA
AGAGCACGAGTACTCTTTACTTGGAAGCGAGGCAAACTTTACAGGGCTTTCAAGAAATTCATGGTGTTCTT
GGTGGTGTGCTGCTCTATCAAAACAAAGTTGTTGCTACTCAATTAAATAAACCATTTACGAAGCAGCTTGTG
GTGACTGACCCTTATAGAATTAAGCTTCCTGCAGAAACATTTGTAACTTCTTTTCATCTACCCTTTGGAGTG
CAGCTGATTAAGGTTTATGTTCCGTCATCTGAGGTTAAACGGCTTCGGCATGAAAATGACGAATTGCTGGA
CGCTTTAAAGGATGTGAGCAATTGGAGGAAAGAGCTACAAAACTCTCAAAATCAGAACACGAATAAGAAT
AGTTGTGTGAAGCCTCCTGAATCCTTTGCCTCCGTGTGTGGTATGAAAAGGGATGTCTCTCGCATCTTCACC
GTCCTCGAGGAGGAAGGAAACGAAAACGTGGAAACGAGAGACCAGACAGACATTCCTGATGTCGTGAGAG
ACGCCGTGAAGGCTAGACACCTCGCCAGGATTGAGGCCGTCGTTCCTAATAACTTTATCATTCCAGAATCCT
CCATAAAATCTCGATCAGCTTTGACACCTGATGATGTATCGACCAGGAAAATTCCTATTAGATATTACAGC
ATCGGCCTGCCGCAGGTTAATTCCGAATGGTGTGATTCTCCGCCCAAGTCTAAAAATCCTAGGCCATATTAT
AACACCATATGCGATCCCAAGTATCCTCTATTTAAATACGATGGACTTCCTGCTTCTGTTTCCCTTTCGGAAT
CTCGAATCATGAAACAGTTTGAAGTGTTAAAAGATGAAAAATACTCGTCTAAAAGCTTGAATTTCGAAAAA
CCTCAGAATGGTAATATTAAAATGGAAAATGGGAATATTAAGATGGAAAATGGTAATATTAAAATGGAAA
ATGGTAATATTAAAATGGAAACATCTGAGAAACCTCCCGAAGTAAAGCCGCAACAAAAAATCAAGTCTTC
CTTTACGTTAC

Protein

<u>RF:</u> +2

<u>ORF:</u> 110 -> 1582

<u>Length</u>: 490 aa

>lcl|ORF1_TRINITY_DN9038_c0_g1_i1:109:1581 unnamed protein product, partial

MAKELLIVFIYDCESCKKEEDDPQDAIIYFYPTWVNNEQRHALCSQLMGVTQFCASSFSLPNIISLQSGKFALKKL
GRFALCVGTDRNIPDLVLETRASILYKLLRLFHFSIENLQESSGEEDISDKLAELLQVYLPILQYATNTFGNIPVLKI
PKSTSTLYLEARQTLQGFQEIHGVLGGVLLYQNKVVATQLNKPFTKQLVVTDPYRIKLPAETFVTSFHLPFGVQL
IKVYVPSSEVKRLRHENDELLDALKDVSNWRKELQNSQNQNTNKNSCVKPPESFASVCGMKRDVSRIFTVLEEE
GNENVETRDQTDIPDVVRDAVKARHLARIEAVVPNNFIIPESSIKSRSALTPDDVSTRKIPIRYYSIGLPQVNSEWC
DSPPKSKNPRPYYNTICDPKYPLFKYDGLPASVSLSESRIMKQFEVLKDEKYSSKSLNFEKPQNGNIKMENGNIK
MENGNIKMENGNIKMETSEKPPEVKPQQKIKSSFTL

Conserved Domains

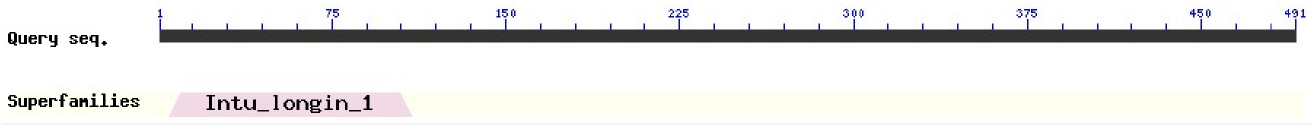

BLASTp

XP_014288755.1 uncharacterized protein LOC106688690 [*Halyomorpha halys*]

Score:897 bits

E-value: 0.0

```
Query  1    MAKELLIVFIYDCECCKKEEDDPQDAIIYFYPTWVNNEQRHALCSQLMGVTQFCASSFSL  60
            MAKELLIVFIYDCE CKKEEDDPQDAIIYFYPTWVNNEQRHALCSQLMGVTQFCASSFSL
Sbjct  1    MAKELLIVFIYDCESCKKEEDDPQDAIIYFYPTWVNNEQRHALCSQLMGVTQFCASSFSL  60

Query  61   PSIISLQSGKFALKKLGRYALCVGTDRNIPDLVLETRASILYKLLRLFHYSIENLQESSI  120
            P+IISLQSGKFALKKLGR+ALCVGTDRNIPDLVLETRASILYKLLRLFH+SIENLQESS
Sbjct  61   PNIISLQSGKFALKKLGRFALCVGTDRNIPDLVLETRASILYKLLRLFHFSIENLQESSG  120

Query  121  DEDISDKLAELLQVYLPILQYATNTFGNIPVLKIPKSTSTLYLEARQTLQGFQEIHGVLG  180
            +EDISDKLAELLQVYLPILQYATNTFGNIPVLKIPKSTSTLYLEARQTLQGFQEIHGVLG
Sbjct  121  EEDISDKLAELLQVYLPILQYATNTFGNIPVLKIPKSTSTLYLEARQTLQGFQEIHGVLG  180

Query  181  GVLLYQNKVVATQLNKAFTKQLVVTDPYRIKLPAETVVTSFHLPFGVQLIKVYVPSAEIK  240
            GVLLYQNKVVATQLNK FTKQLVVTDPYRIKLPAET VTSFHLPFGVQLIKVYVPS+E+K
Sbjct  181  GVLLYQNKVVATQLNKPFTKQLVVTDPYRIKLPAETFVTSFHLPFGVQLIKVYVPSSEVK  240

Query  241  RLREENHELLDALKDVSNWRKELQNSQSQNVNKNSCVKPPESFVSMCGMKRDVSRIFTVL  300
            RLR EN ELLDALKDVSNWRKELQNSQ+QN NKNSCVKPPESF S+CGMKRDVSRIFTVL
Sbjct  241  RLRHENDELLDALKDVSNWRKELQNSQNQNTNKNSCVKPPESFASVCGMKRDVSRIFTVL  300

Query  301  EEEGNENLETRDQTDIPDVVRDAVKARHLARIEAIVPNNFIIPESSIKIRPALTPDDIST  360
            EEEGNEN+ETRDQTDIPDVVRDAVKARHLARIEA+VPNNFIIPESSIK R ALTPDD+ST
Sbjct  301  EEEGNENVETRDQTDIPDVVRDAVKARHLARIEAVVPNNFIIPESSIKSRSALTPDDVST  360

Query  361  RKIPIRYYSIGLPQVNSEWCDSPPKPRSPRPYYNTICDPKYPIFKHDGLPASVSLSESRI  420
            RKIPIRYYSIGLPQVNSEWCDSPPK ++PRPYYNTICDPKYP+FK+DGLPASVSLSESRI
Sbjct  361  RKIPIRYYSIGLPQVNSEWCDSPPKSKNPRPYYNTICDPKYPLFKYDGLPASVSLSESRI  420
```

Query 421 MKQFEVLKEEKHLPKSLNIGKTQNGNI----------------NGNNKIETADKPPEVK 463

MKQFEVLK+EK+ KSLN K QNGNI NGN K+ET++KPPEVK

Sbjct 421 MKQFEVLKDEKYSSKSLNFEKPQNGNIKMENGNIKMENGNIKMENGNIKMETSEKPPEVK 480

Query 464 PQQKIKSSFTL 474

PQQKIKSSFTL

Sbjct 481 PQQKIKSSFTL 491

F-box protein 11 (FBX011)

>TRINITY_DN19510_c0_g1_i1 len=2901 path=[0:0-2668 1:2669-2900]

CGGTGATTGGGTTTGTGCTTCACGAAATAGTTTTTGACCATAGTGTACCTTAGAGTTTAAAATGCCGAGTGC
TTCTTATGGCTCTTCAAGATATTATCCACGAAGATCGAGACGTAAAGGAAATAACAGAGTATCCGTATCGA
ACCGAAACAATACTGCCGAAACCTGCGCCTCTGGAAGCAGTTCAAATGGAATACCTACTACTGTTATGAAC
TCACAACCCCCTCACAATCCTCCATATGATCTGCGCAGAAAATCACCCCCTTACCCAATTTCAGAAAATGGC
TTTCCTAATCTGAGGAAAAGGCCTCGCAGGTCATGTTCTGTCAATACAGATATATTCTCACCCCCTGCTGCC
CATTACCTTCAATATGAACTGCCTGATGAGGTACTTTTAACTATATTCAGTTATTTGCTAGAAAAAGATTTA
TGTCGTCTTTCTCAAGTTTGTAAGAGATTTCAAACCATTGCCAATGATAATGGATTATGGAAAAGACTTTAC
CATAATATCTTCGAATATGACATCCCGTTGTTTAATCCTGAACCATGTAAATTTGAATTCATACCACCAAAT
GATTGTGAATATGCAAATCCTTGGAAAGAAAGCTTCCGGCAGTTATATCGAGGGATTCACGTTCGACCTGG
TCAGGGTTCACGGTATAAAGCTAGAGGTAGATCTCTAGCTCATTTTGATACTGTTCAGACAGCTTTAGATCA
TGCTGATGAAAATCCAACAGCAGGCATTCCTTCTATTGTTTTTCTTCATGCTGGTACTTATAGAGGTGAATT
CTTGGTGATAGATACTGATGTTGCCCTTATAGGAGCTGCTCCAGGGAATGTTGCTGAATCAGTAATTTTAGA
AAGGCAGTCTGGTTCTACTATGATGTTTGGTGATGGTGCTAAGTCAGCCTATGCTGGTCACCTCACTTTAAA
GTTTACTCCTGATGTTGCATCTACTGTCCTGCACCATAAACACTACTGTCTAGAAGTAGGAGAGAATTGTAG
TCCAACTATTGATCACTGTATTATTAGAAGCACCTCAGTTGTTGGAGCTGCTGTTTGTGTCAGTGGCCAAGG
AGCTGCACCCATTATAAAACATTGTGACATTAGTGATTGTGAAAATGTTGGTCTATATGTAACTGACTATGC
ACATGGCACATATGAAGACAATGAAATTTCAAGAAATGCACTTGCTGGTATTTGGGTAAAGAATTATGCCA
ACCCTATTATGAGAAGAAATCATATACACCATGGAAGAGATGTTGGAATTTTTACTTTTGACAATGGTTTAG
GATTCTTTGAGGCAAATGATATTCATAACAATAGAATAGCTGGATTTGAAGTTAAAGCTGGGGCTAATCCA
ACTGTAGTTCAATGTGAAATACATCATGGTCAGACAGGTGGTATCTATGTACATGAAAATGGGCTAGGTCA
ATTTATTGACAACAGAATCCACTCAAATAATTTTGCTGGAGTTTGGATAACATCCAATAGCAACCCTACAAT
TAGACGAAATGAAATATATAATGGGCAACAAGGTGGTGTGTATATATTTGGCGAAGGGCGAGGTCTCATTG
AACACAATAACATTTATGGAAATGCTTTGGCTGGGATACAAATCCGTACTAACAGTGACCCAATTGTTAGG
CATAACAAAATACATCATGGTCAACATGGTGGAATTTATGTACATGAAAAGGGTGAAGGACTGATAGAAG
AGAATGAAGTCTATGCTAACACTTTGGCTGGGGTTTGGATCACTACTGGATCAACACCAGTCCTCCGTCGG
AATCGAATTCATTCTGGCAAACAAGTTGGTGTATATTTTTATGATAATGGGCATGGAAAACTCGAGGATAA
TGATATATTTAATCATTTATATTCCGGAGTACAGATCAGGACTGGAAGTAATCCTGTTATACGTGGTAATAA
GATCTGGGGAGGTCAAAATGGTGGAGTTTTGGTTTATAATGGTGGTCTTGGTTTATTGGAACAAAATGAGA
TATTTGACAATGCAATGGCTGGTGTATGGATCAAAACTGACTCTAATCCAACCTTGAAGCGCAACAAAATT
TTTGATGGAAGAGATGGAGGAATTTGTATTTTTAATGGTGGAAAAGGTATTTTGGAGGAAAATGATATATT
CAGGAATGCTCAAGCAGGAGTTTTAATTTCAACTCAAAGTCATCCCGTCTTGAGACATAATAGAATATTTG
ATGGATTAGCAGCAGGTATTGAAATAACTTTGAATGCCACAGCAACATTGGAAAATAATCAAATTTTTAAC
AATAGGTTTGGGGGACTTTGTCTTGCTACTGGTGTCAATCCTATTGTACGATGTAATAAAATCTTTAGTAAC
CAAGATGCCGTAGAAAAAGCTGTTAGGAATGGGCAATGCCTGTATAAAATCTCTAGTTACACTTCATTCCC
CATGCATGATTTTTACCGTTGCCAAACTTGCAATACCACTGGTCGAAATGCCATCTGTGTAAATTGTATTAA

AACTTGTCATGCTGGACATGAAGTAGAATTTGTAAGACATGATAGGTTTTTCTGTGATTGTGGCGCTGGTAC
ATTATGTAATCAGTGCCAGCTGCAAGGTGAACCAGCCCAAGATACTGATACGTTATATGATTCTGCCGAGC
CAATGGAATCACACACTCTAATGGTTAATTGAACCCTTTGGCGTTACCAATATTTATTGAAATTATACTCTT
TCTATTGTTCAGAATTGTCTTGTTATTATAGAAATAATTAGCATATGTGTTGATGAGTTGGTAAGAATGTTT
GGCGGATAAAGAAAGAATGCACAAACACATCACATACACTCAATTACTTATGTTTTTATGCTAATGGAAAG
TTTTTTTATTTTATTATACTAAGTTCAAGGTAAAAATAATG

Protein

RF: +2

ORF: 62 -> 2677

Length: 871 aa

>lcl|ORF1_TRINITY_DN19510_c0_g1_i1:61:2676 unnamed protein product

MPSASYGSSRYYPRRSRRKGNNRVSVSNRNNTAETCASGSSSNGIPTTVMNSQPPHNPPYDLRRKSPPYPISENG
FPNLRKRPRRSCSVNTDIFSPPAAHYLQYELPDEVLLTIFSYLLEKDLCRLSQVCKRFQTIANDNGLWKRLYHNIF
EYDIPLFNPEPCKFEFIPPNDCEYANPWKESFRQLYRGIHVRPGQGSRYKARGRSLAHFDTVQTALDHADENPTA
GIPSIVFLHAGTYRGEFLVIDTDVALIGAAPGNVAESVILERQSGSTMMFGDGAKSAYAGHLTLKFTPDVASTVL
HHKHYCLEVGENCSPTIDHCIIRSTSVVGAAVCVSGQGAAPIIKHCDISDCENVGLYVTDYAHGTYEDNEISRNA
LAGIWVKNYANPIMRRNHIHHGRDVGIFTFDNGLGFFEANDIHNNRIAGFEVKAGANPTVVQCEIHHGQTGGIY
VHENGLGQFIDNRIHSNNFAGVWITSNSNPTIRRNEIYNGQQGGVYIFGEGRGLIEHNNIYGNALAGIQIRTNSDPI
VRHNKIHHGQHGGIYVHEKGEGLIEENEVYANTLAGVWITTGSTPVLRRNRIHSGKQVGVYFYDNGHGKLEDN
DIFNHLYSGVQIRTGSNPVIRGNKIWGGQNGGVLVYNGGLGLLEQNEIFDNAMAGVWIKTDSNPTLKRNKIFDG
RDGGICIFNGGKGILEENDIFRNAQAGVLISTQSHPVLRHNRIFDGLAAGIEITLNATATLENNQIFNNRFGGLCLA
TGVNPIVRCNKIFSNQDAVEKAVRNGQCLYKISSYTSFPMHDFYRCQTCNTTGRNAICVNCIKTCHAGHEVEFV
RHDRFFCDCGAGTLCNQCQLQGEPAQDTDTLYDSAEPMESHTLMVN

Conserved Domains

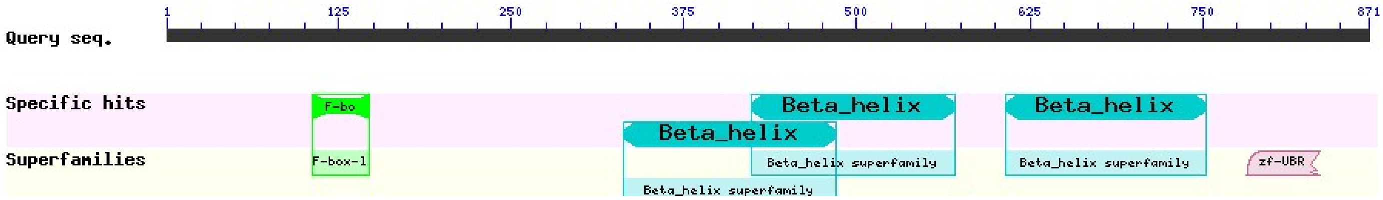

BLASTp

XP_014287303.1 F-box only protein 11 [*Halyomorpha halys*]

Score:1799 bits

E-value: 0.0

Query 1 MPSASYGSSRYYPRRSRRKGNNRVSVSNRNNTAETCASGSSSNGIPTTVMNSQPPHNPPY 60

MPSASYGSSRYYPRRSRRKGNNRVSVSNRNNTAETCASGSSSNGIPTTVMNSQPPHNPPY

Sbjct 1 MPSASYGSSRYYPRRSRRKGNNRVSVSNRNNTAETCASGSSSNGIPTTVMNSQPPHNPPY 60

Query 61 DLRRKSPPYPILENGFPNLRKRPRRSCSVNTDIFSPPAAHYLQYELPDEVLLTIFSYLLE 120

DLRRKSPPYPI ENGFPNLRKRPRRSCSVNTDIFSPPAAHYLQYELPDEVLLTIFSYLLE

Sbjct 61 DLRRKSPPYPISENGFPNLRKRPRRSCSVNTDIFSPPAAHYLQYELPDEVLLTIFSYLLE 120

Query 121 KDLCRLSQVCKRFQTIANDNGLWKRLYHNIFEYDIPLFNPEPCKFEFIPPNDCEYANPWK 180

KDLCRLSQVCKRFQTIANDNGLWKRLYHNIFEYDIPLFNPEPCKFEFIPPNDCEYANPWK

Sbjct 121 KDLCRLSQVCKRFQTIANDNGLWKRLYHNIFEYDIPLFNPEPCKFEFIPPNDCEYANPWK 180

Query 181 ESFRQLYRGIHVRPGQGSRYKARGRSLAHFDTVQTALDHADENPTSGVPSIVFLHAGTYR 240

ESFRQLYRGIHVRPGQGSRYKARGRSLAHFDTVQTALDHADENPT+G+PSIVFLHAGTYR

Sbjct 181 ESFRQLYRGIHVRPGQGSRYKARGRSLAHFDTVQTALDHADENPTAGIPSIVFLHAGTYR 240

Query 241 GEFLVIDTDVALIGAAAGNVAESVVLERQSGSTMMFGDGAKSAYAGHLTLKFTPDVASTV 300

GEFLVIDTDVALIGAA GNVAESV+LERQSGSTMMFGDGAKSAYAGHLTLKFTPDVASTV

Sbjct 241 GEFLVIDTDVALIGAAPGNVAESVILERQSGSTMMFGDGAKSAYAGHLTLKFTPDVASTV 300

Query 301 PHHKHYCLEVGENCSPTIDHCIIRSTSVVGAAVCVSGQGAAPVIKHCDISDCENVGLYVT 360

HHKHYCLEVGENCSPTIDHCIIRSTSVVGAAVCVSGQGAAP+IKHCDISDCENVGLYVT

Sbjct 301 LHHKHYCLEVGENCSPTIDHCIIRSTSVVGAAVCVSGQGAAPIIKHCDISDCENVGLYVT 360

Query 361 DYAHGTYEDNEISRNALAGIWVKNYANPIMRRNHIHHGRDVGIFTFDNGLGFFEANDIHN 420

DYAHGTYEDNEISRNALAGIWVKNYANPIMRRNHIHHGRDVGIFTFDNGLGFFEANDIHN

Sbjct 361 DYAHGTYEDNEISRNALAGIWVKNYANPIMRRNHIHHGRDVGIFTFDNGLGFFEANDIHN 420

Query 421 NRIAGFEVKAGANPTVVQCEIHHGQTGGIYVHENGLGQFIDNRIHSNNFAGVWITSNSNP 480

NRIAGFEVKAGANPTVVQCEIHHGQTGGIYVHENGLGQFIDNRIHSNNFAGVWITSNSNP

Sbjct 421 NRIAGFEVKAGANPTVVQCEIHHGQTGGIYVHENGLGQFIDNRIHSNNFAGVWITSNSNP 480

Query 481 TIRRNEIYNGQQGGVYIFGEGRGLIEHNNIYGNALAGIQIRTNSDPIVRHNKIHHGQHGG 540

TIRRNEIYNGQQGGVYIFGEGRGLIEHNNIYGNALAGIQIRTNSDPIVRHNKIHHGQHGG

Sbjct 481 TIRRNEIYNGQQGGVYIFGEGRGLIEHNNIYGNALAGIQIRTNSDPIVRHNKIHHGQHGG 540

Query 541 IYVHEKGEGLIEENEVYANTLAGVWITTGSTPVLRRNRIHSGKQVGVYFYDNGHGKLEDN 600

IYVHEKGEGLIEENEVYANTLAGVWITTGSTPVLRRNRIHSGKQVGVYFYDNGHGKLEDN

Sbjct 541 IYVHEKGEGLIEENEVYANTLAGVWITTGSTPVLRRNRIHSGKQVGVYFYDNGHGKLEDN 600

Query 601 DIFNHLYSGVQIRTGSNPVIRGNKIWGGQNGGVLVYNGGLGLLEQNEIFDNAMAGVWIKT 660

DIFNHLYSGVQIRTGSNPVIRGNKIWGGQNGGVLVYNGGLGLLEQNEIFDNAMAGVWIKT

Sbjct 601 DIFNHLYSGVQIRTGSNPVIRGNKIWGGQNGGVLVYNGGLGLLEQNEIFDNAMAGVWIKT 660

Query 661 DSNPTLKRNKIFDGRDGGICIFNGGKGILEENDIFRNAQAGVLISTQSHPVLRHNRIFDG 720

DSNPTLKRNKIFDGRDGGICIFNGGKGILEENDIFRNAQAGVLISTQSHPVLRHNRIFDG

Sbjct 661 DSNPTLKRNKIFDGRDGGICIFNGGKGILEENDIFRNAQAGVLISTQSHPVLRHNRIFDG 720

Query 721 LAAGIEITLNATATLENNQIFNNRFGGLCLATGVNPIVRCNKIFSNQDAVEKAVRNGQCL 780

LAAGIEITLNATATLENNQIFNNRFGGLCLATGVNPIVRCNKIFSNQDAVEKAVRNGQCL

Sbjct 721 LAAGIEITLNATATLENNQIFNNRFGGLCLATGVNPIVRCNKIFSNQDAVEKAVRNGQCL 780

Query 781 YKISSYTSFPMHDFYRCQTCNTTGRNAICVNCIKTCHAGHEVEFVRHDRFFCDCGAGTLC 840

YKISSYTSFPMHDFYRCQTCNTTGRNAICVNCIKTCHAGHEVEFVRHDRFFCDCGAGTLC

Sbjct 781 YKISSYTSFPMHDFYRCQTCNTTGRNAICVNCIKTCHAGHEVEFVRHDRFFCDCGAGTLC 840

Query 841 NQCQLQGEPAQDTDTLYDSAEPMESHTLMVN 871

NQCQLQGEPAQDTDTLYDSAEPMESHTLMVN

Sbjct 841 NQCQLQGEPAQDTDTLYDSAEPMESHTLMVN 871

Clathrin heavy chain (Chc)

>TRINITY_DN2469_c0_g1_i1 len=7836 path=[0:0-7835]

GTAGTTCCACGGAACACTGTTCTGCTTGCTAGTGCATTTTTCGTCTAGTTTATTCTTGTTGGGTTGGATCATT
GACAGTTATAAGATCGGAAGACGTTAACAAACACATTTCGTCTCTAACCCTTTCAATAAAAGTGGGCGTAA
GGTTATTCTTATGTGAATCTGGTGTAGCCATGTTGAATTAAAGATTGATAATATCCATGGTGTTGGAGGTGG

AAGTGATTGTTTAGCTTTCCTGAGGGGTCAATAGGGTAATTTAAGGAAGATTAAGTATTTAGTTGAAGCTAT
TTTATATTATCCAAGATGTCTCAAATATTACCGATACGCTTCCAAGAACATTTACAGCTTTCGAGTGTAGGA
ATAAACCCAACTAGTGTAGGCTTTAACACATTAACCATGGAATCTGACAAGTTCATATGCGTTAGAGAAAA
AGTTGGAGAAACTTCTCAAGTTGTGATAATAGATTTAAATGATACCGCTAATCCAATAAGAAGACCTATTT
CAGCGGATTCAGCTATCATGAACCCAGCTAGTAAAGTTATAGCATTGAAGGGAAAAGCTGGCACTGATGG
AACAACCACCCAGAAAACATTGCAGATTTTTAATATCGAAATGAGAAGTAAGATGAAAGCACATACTATG
ACTGATGAAGTAGTTTTTTGGAAATGGATATCCCTTAATACACTTGCGTTAGTCACTGAAACGTCTGTTTAT
CATTGGAGCATGGAGGGTGACTCTCAACCAGTGAAAATGTTCGATAGACATTCTAGCCTGAATGGTTGTCA
AATTATAAATTACAGAACAGATCCCAAGCAAACCTGGCTTTTACTTATTGGAATCTCTGCTCAGCATAATCG
TGTTGTTGGAGCTATGCAATTATATTCAGTAGAGCGCAAATGCTCACAGCCGATTGAAGGACATGCTGCAT
CATTTGCTCAGTTCAAAATGGAAGGAAACCCTGACGTATCTACTTTGTTTTGCTTTGCAGTTCGTACACCGA
CAGGAGGAAAGCTTCATATAATTGAGGTTGGACAGCCCCCCTCAGGAAACCAGCCATTCGCTAAAAAAGC
AGTTGATGTATTTTTTCCGGTAGAAGCCCAAAATGATTTTCCAGTTGCTATGCAGGTTAGCTCTAAGTATGA
TGTAATCTATCTAATAACAAAGTATGGATATATTCATCTGTATGATTTGGAAACAGCTACATGCATTTACAT
GAATCGTATTAGCATTGATACTATATTTGTCACTGCACCTCATGAATCAACTGGAGGTATCATTGGGGTGAA
TAGAAAAGGCCAGGTGTTATCAGTGAGTGTTGAAGAGGACCACATAATCCCATATATCAATAATATATTAC
AAAATCCAGATCTAGCCTTACGCATGGCAGTTAGAAATAATCTAGCTGGAGCTGAAGATTTGTTTGTTAAA
AAATTCAATATGCTTTTCCAGAATGGGCAGTATGCTGAGGCTGCTAAAGTGGCAGCCAATGCTCCCAAGGG
AATACTGAGAACCCCACAAACCATTCAGCAGTTCCAACAAATACCAAATCCGCAAGGGCAAACATCACCTC
TTTTGCAATATTTTGGAATTTTGCTTGATCAGGGTCAATTGAACAAGTATGAATCCTTAGAATTATGCAGAC
CTGTTCTGGCTCAAGGAAGAAAACAGTTAATGGAAAAATGGCTGAAAGAAGATAAACTAGAGTGCTCTGA
AGAACTCGGAGATCTAGTAAAACAAACAGACCCAACTCTTGCACTCTCTGTTTATTTAAGAGCTAATGTTCC
AAACAAGGTTATACAATGCTTTGCTGAAACTGGACAATTCCAGAAGATTGTACTATATGCAAAAAAAGTAG
GTTATTCTCCTGACTACATTTTCCTGCTCCGAAATGTAATGCGTGTTAATCCTGATCAAGGTGTTAGCTTTGC
TCAAATGCTTGTTCAAGATGAAGAACCACTGGCTGATATAAACCAGATTGTTGATATACTTATGGAACAAA
ATATGGTGCAGCAGTGCACTGCTTTTCTTTTGGATGCCCTTAAGAATAATCGGCCTTCAGAAGGTCCATTAC
AGACCAGGCTACTTGAAATGAATTTGATTTCTGCACCTCAGGTTGCTGATGCTATACTTGGGAACCAAATGT
TTACACATTATGATAGAGCCCACATTGCCCAGCTTTGTGAGAAGGCTGGTTTACTCCAGAGAGCGTTGGAA
CATTACACAGATCTTTACGATATTAAACGAGCTGTTGTTCACACTCACCTCCTTGGCCCTGATTGGCTTGTG
GGATACTTTGGTACCCTTTCTGTTGAAGATTCACTTGAATGTTTGAAAGCCATGCTAACTGCAAATATTCGG
CAGAACTTGAATATATGTGTTCAGATTGCTACCAAGTACCATGAACAACTAACAACCAAGGCTTTAATAGA
TCTTTTTGAATCATTCAAAAGTTATGAGGGATTGTTTTATTTCCTTGGCTCAATAGTCAATTTCAGCCAAGAT
CAAGAAGTTCATTTTAAATACATCCAGGCAGCCTGTAAAACAGGGCAGATAAAAGAAGTTGAACGAATTT
GCCGTGAATCTAATTGCTATAATCCAGAGAGAGTTAAAAATTTTTGAAGGAAGCAAAGTTGACAGATCAA
TTACCACTTATTATTGTTTGTGATCGGTTTGATTTTTGTCATGATTTGGTTTTATATTTATACCGAAACAATTT
GCAAAAGTACATTGAAATTTATGTGCAAAAGGTCAATCCATCTCGGTTGCCAGTTGTGATAGGAGGATTAT
TAGATGTTGATTGTTCTGAAGACATAATTAAAAATTTGATGATGGTTGTTAGAGGTCAATTTTCAACTGAGG
AGCTTGTTGCAGAAGTAGAAAAGCGAAATCGATTAAAACTTCTGCTTCCTTGGCTGGAAACTCGTGTTCAT
GAAGGCTGTGTTGAGCCTGCTACTCATAACGCTTTAGCAAAGATATACATTGATAGCAATAATAATCCAGA
ACGATTCTTGAGGGAAAATCAGTATTATGATAGCTGTGTTGTAGGAAAGTACTGTGAAAAACGAGATCCAC
ATTTGGCATGTGTAGCCTATGAAAGAGGTCAATGTGATAGAGAGCTCATCAATGTCTGTAATGAAAATTCT
CTCTTTAAATCAGAAGCAAGGTACTTGGTTAGACGTAAAGACCCTGAATTGTGGGCTGAAGTTCTTAATGA
AAACAATCCTTACAAGAGACCATTAATTGATCAGGTTGTTCAAACAGCTCTTTCGGAAACTCAAGATCCTG
AAGATATCAGTGTGACTGTCAAAGCTTTCATGACAGCTGATTTACCCAATGAACTTATTGAACTCCTAGAA
AAAATAGTTCTAGACAATTCAATATTTAGTGATCACCGCAATCTTCAAAATCTTCTCATTCTTACTGCTATA
AAAGCTGACCGTTCAAGAGTGATGGAATACATTAACCGTTTAGATAATTATGATGCACCTGATATAGCAAA
TATTGCTATAAATTATCAATTATATGAAGAAGCATTTGCAATATTTAAAAAGTTCGACGTTAATACTTCTGC
TATCCAGGTTCTAATTGAAAATGTGAACAATCTCGATAGAGCCTATGAATTCGCAGAACGCTGTAACGAGC
CAGCTGTGTGGAGTCAATTAGCCAAAGCTCAACTGCAACATGGCCTTGTAAAAGAAGCAATAGATTCATTC
ATAAAAGCTGACGACCCTTCTGCATATGTTGATGTGGTGGAAACAGCTCACAAAACAGAAAGCTGGGAGG
ATTTAGTCCGCTATTTACAAATGGCAAGGAAAAAGGCTAGAGAATCTTACATTGAAAGTGAGCTGATTTAT
GCTTATGCTCGCACTAACCGATTGGCTGATCTTGAAGAATTTATCTCAGGTCCGAATCATGCTGATATTCAA
AAGATTGGTGATAGGTGTTTTGATGACAAGATGTATGAACCAGCTAAACTGCTTTACAACAATGTTTCAAA
CTTTGCTAGACTAGCTATAACACTAGTTCATTTAAAAGAATTTCAAGGAGCTGTGGACAGTGCAAGGAAAG
CCAACAGTACAAGAACTTGGAAAGAAGTCTGTTTTGCTTGTGTGGATAGTGAGGAATTCAGATTAGCTCAG
ATGTGTGGTTTGCATATTGTGGTTCACGCAGATGAACTTGAAGACCTAATCAATTATTATCAGGACCGAGG
ATACTTTGAAGAGCTTATCAATTTATTGGAAGCTGCACTGGGGTTAGAGCGTGCTCATATGGGAATGTTCAC
AGAATTAGCTATACTCTATTCCAAGTATAAACCAGCAAAAATGAGGGAACATTTAGAATTATTTGGTCTC

GAGTTAATATTCCCAAGGTCTTGAGAGCTGCTGAACAAGCTCATTTATGGGCAGAACTTGTTTTCTGTATG
ATAAGTATGAAGAGTATGATAATGCTGTGTCTGCAATGATGAATCACCCTACTGAGGCTTGGAGAGAAGGA
CATTTTAAGGACATAATTACCAAGGTTGCCAACATTGAACTATATTACAAAGCAATACAATTTTATTTGGAT
TATAAACCTCTCCTATTGAATGATATGTTATTGGTATTAGCCCCAAGAATGGATCATACAAGAGCAGTAAA
CTTCTTTGCCAAGGTTAACCATTTACAATTGGTTAAGCCTTATCTTCGCTCAGTACAATCATTAAACAATAA
AGCAATAAATGAAGCTCTAAACAATCTTCTCATTGAAGAGGAAGACTTCCAGGGTGTCAGAACATCAATAG
ATGCTTTTGATAATTTCGACAATATTGTATTAGCTCAAAAGCTTGAAAAGCATGAGTTGATCGAATTCAGGA
GGATAGCAGCTTATCTATATAAAGGAAATAACCGATGGAAGCAGAGTGTTCAGTTATGCAAGAAAGATAG
ATTGTTCAAGGATGCAATGGAGTATGCTGCAGAATCAAAGAACTCAGAAGTTGCCGAAGAACTTTTAGCAT
GGTTCTTAGAAAAAGGAAATCATGACTGTTTTGCTGCTTGTCTGTTCCAATGTTATGATCTTCTTCATCCTGA
TGTTATTCTAGAACTTGCATGGCGACATAATATCATGGACTTCGCCATGCCTTATATCATTCAAGTGACAAG
AGAGTACATTTCCAAGGTTGATAAATTGGAAGAATCTGAAGCAAGAAGGTTAGAAGAAAGAGCAGAACAG
GATCACAAACCTATGATGATGCCTGAGCCACAGCTAATGTTAACTGCTGGCCCAGGAATGATCGGACCTGG
TTTTTCACCTGCTTATCCTAACACTTTTACCACTGGCATGCCTTATCAAGGTTATGGCATGTAGGTAATATGA
AATGTGATCAACTTCCCCGTATTAAGCAACTAAAGTGAATGGTCTAACATCCATCCAACCAGAAAGTGCAA
AGTGAAATGAATGTGAAATAAATTTTTCTATAGTTGCTAAAGAGATTGTTTTTGTTTTCTCAACACATGATC
TAATTCAAGGCCTCTTATACTGTGGTTCTGTCTTCATTAAAAAATAAATTGTATAAAGTTTAATACTGCCTTC
ATAAGTGTGCTTTTAATGAATATCATGCGCATATAAACTTTGTAAAAATCTATATGTATGTTATGATATTTCT
GTTGATATTCATAAATCTTATTTAGTCCTGAATGGTAAAGTTAGCATTAACTTATGATTAGATAACGGATAG
TCTGTTCCGTTACTACATATATTTTTTATAGTATTTAATGAATTTGTTAACTATTTAAGTTTATGTTATGCATT
CCATTTCTTCACACATATACACATACCTAGACTCACATTTACACCTAATGTGTATGTGTGGAATTATGTGTT
CTAGAGAATAGAAACACTATTTATTGTGGTATTATCTTGTTAGCTTGTGTGAATATATGTTTTATTAGTAAA
ATGATTTACTGCTACATTTGAAAAATTGTTTATATATTTAAATTATGAATTTATCTAATTTCTGTGTTTCTAT
GTTATGTTACTTTTTTTTTTATTAGTTATCTCTTGAATAATGTTATTTAGGTGTATTATTATTATTATTATTATT
ATTATTAGTACCAACATGCAATATATGCACTATAGTTCAACATATAAATAGACATTATGGAAATATGTAAA
GATTGAAAAATACACTCTTTATTATTAGAATCAAATTGTATTTAAAAAATATATAAGTAATTGTTAATGATT
ATTATATCTTATGGATGTAATGAGTCCAAAGAGGAGAAACATCAGTTATGTAGAGCAAAACTCTGGCTTCA
TATCCAGATATGTATCTGTATCATATAAGTTACAATGCTTTCATTATGTTTTTGTTTTCTTTTAAAATATATA
AATGTTGCTGGATATGAAGGTAGAGTCAAAAGTTGAATTAAATAGTTTTAATTTTTCAAAATAAACATAAT
ATTTTGTTTTATCAGTGGGTAGATTAACTTGATTTATTGGCATGTGCCTTATTTAAATGAACATTTATTCCAT
CTATTGATGGGTGTGAATATCATAAAGCCTGCATAACATTTATTATTAACATCATGAAAAAAGTACTCTCAT
TTATTTTAAATTTATATTATTTATGCAATACATTAAAAAAGTAAAAAATAATTTATTTTCATTCATTAAAATT
TTAATTGATTATTTATTTCAGTTAAAATCCATTATTTTTTGTCATCCCATATATCTAATGATTAACCTTCTTAG
GTTAAGATGTCATTGTTTAATTACAGTTGTTACATCACCCTTTGGCATTACTATGATTACAAAGGATGTAGC
AATGTGCCAAACAATTAAATACCATATATGAATATGGCTAACAATAAATTAAATTTTAAGATATATAATAT
CTTCCATCTTCCATTTCTATATCTAGTTATTTCAAGAAATGAACATTCACAGTAATCCTTTCCCTTGCCTGTT
TTATTTAAATGTGGTGATTTTTTTAGTTTATTTTGTTGCAAGCTTTTTCATCGTATGTAAGTTTTCAACTGATT
AATGTTCATAAAATGAAAATTAATATAAATTATAGATTTTAGTTATTTAACTGATAACCTGGTCTTTTCATCT
GTTTTTATGTCCAATTTTACCATAAAGATACATGTGTTTCATTAAAATCTTCTTTTCTTTTTTTTAATTAATAA
GTGAACTGCCGTTTTTTTGCTTCTTAGAGGTTATGCCAATGATATAAATGTCTGATTTAAAGATTTTACTGTA
CTCAACATTATAGAGTGGTTAGTAATAAAAGGCTTAACAAGATATAAGATTCAATATCGATCCCACCATTC
GCTGTTTATGCCTCTATCTAAACCCAAATCCGCAAGGTTAGCTTCACTAGAATGCTCCCATCTTAAGCTCAG
TCTACCCGCTCTGACATTCACCCGTTGTTTATGTACTAATTAAATTTTAAGGATTTGTTTGCAACTTAGCACA
ACATTTACAAGCTCATTTTGATAATATTCCAATATGTTTTCATGGCACACTTACCGAAATTCAACTTCATAA
AGCCTATAAACTTCTAAAAAATATTTTGTATTGTTTTTCAGGTTTTCACATCCTTTCCCCACCAGTAATGGTA
AATAGTGTTCCCTCAATTATTTCCATCATATTTTACCTTGTTTACTATTATAGATTCTCAGGTTTTCTGTCAAA
AAGACTTTTTTTTTTAAATATTGAGTCTAGTTTTTATTACAATCCTATATACATTACTCAAACACATGGGCCA
CCATAAGAACTACAATAGATCAGAGAGATTATTAAGTATGATTGAAACAATAAAAGTGTTACATTTTGATA
GAAGTTAAAAAAAAA

Protein

<u>RF:</u> +1

ORF: 304 -> 5346

Length: 1680 aa

>lcl|ORF1_TRINITY_DN2469_c0_g1_i1:303:5345 unnamed protein product

MSQILPIRFQEHLQLSSVGINPTSVGFNTLTMESDKFICVREKVGETSQVVIIDLNDTANPIRRPISADSAIMNPASK
VIALKGKAGTDGTTTQKTLQIFNIEMRSKMKAHTMTDEVVFWKWISLNTLALVTETSVYHWSMEGDSQPVKM
FDRHSSLNGCQIINYRTDPKQTWLLLIGISAQHNRVVGAMQLYSVERKCSQPIEGHAASFAQFKMEGNPDVSTLF
CFAVRTPTGGKLHIIEVGQPPSGNQPFAKKAVDVFFPVEAQNDFPVAMQVSSKYDVIYLITKYGYIHLYDLETAT
CIYMNRISIDTIFVTAPHESTGGIIGVNRKGQVLSVSVEEDHIIPYINNILQNPDLALRMAVRNNLAGAEDLFVKKF
NMLFQNGQYAEAAKVAANAPKGILRTPQTIQQFQQIPNPQGQTSPLLQYFGILLDQGQLNKYESLELCRPVLAQ
GRKQLMEKWLKEDKLECSEELGDLVKQTDPTLALSVYLRANVPNKVIQCFAETGQFQKIVLYAKKVGYSPDYI
FLLRNVMRVNPDQGVSFAQMLVQDEEPLADINQIVDILMEQNMVQQCTAFLLDALKNNRPSEGPLQTRLLEMN
LISAPQVADAILGNQMFTHYDRAHIAQLCEKAGLLQRALEHYTDLYDIKRAVVHTHLLGPDWLVGYFGTLSVE
DSLECLKAMLTANIRQNLNICVQIATKYHEQLTTKALIDLFESFKSYEGLFYFLGSIVNFSQDQEVHFKYIQAACK
TGQIKEVERICRESNCYNPERVKNFLKEAKLTDQLPLIIVCDRFDFCHDLVLYLYRNNLQKYIEIYVQKVNPSRLP
VVIGGLLDVDCSEDIIKNLMMVVRGQFSTEELVAEVEKRNRLKLLLPWLETRVHEGCVEPATHNALAKIYIDSN
NNPERFLRENQYYDSCVVGKYCEKRDPHLACVAYERGQCDRELINVCNENSLFKSEARYLVRRKDPELWAEVL
NENNPYKRPLIDQVVQTALSETQDPEDISVTVKAFMTADLPNELIELLEKIVLDNSIFSDHRNLQNLLILTAIKADR
SRVMEYINRLDNYDAPDIANIAINYQLYEEAFAIFKKFDVNTSAIQVLIENVNNLDRAYEFAERCNEPAVWSQLA
KAQLQHGLVKEAIDSFIKADDPSAYVDVVETAHKTESWEDLVRYLQMARKKARESYIESELIYAYARTNRLAD
LEEFISGPNHADIQKIGDRCFDDKMYEPAKLLYNNVSNFARLAITLVHLKEFQGAVDSARKANSTRTWKEVCFA
CVDSEEFRLAQMCGLHIVVHADELEDLINYYQDRGYFEELINLLEAALGLERAHMGMFTELAILYSKYKPAKM
REHLELFWSRVNIPKVLRAAEQAHLWAELVFLYDKYEEYDNAVSAMMNHPTEAWREGHFKDIITKVANIELYY
KAIQFYLDYKPLLLNDMLLVLAPRMDHTRAVNFFAKVNHLQLVKPYLRSVQSLNNKAINEALNNLLIEEEDFQG
VRTSIDAFDNFDNIVLAQKLEKHELIEFRRIAAYLYKGNNRWKQSVQLCKKDRLFKDAMEYAAESKNSEVAEEL
LAWFLEKGNHDCFAACLFQCYDLLHPDVILELAWRHNIMDFAMPYIIQVTREYISKVDKLEESEARRLEERAEQ
DHKPMMMPEPQLMLTAGPGMIGPGFSPAYPNTFTTGMPYQGYGM

Conserved Domains

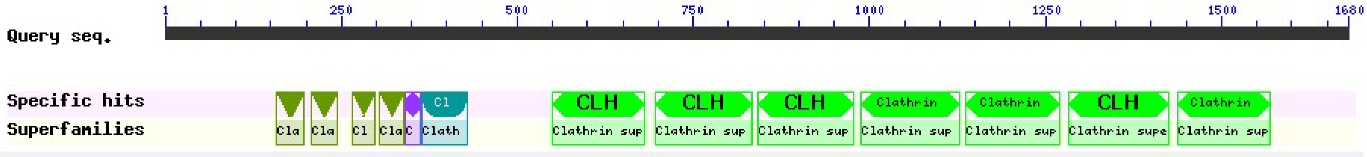

BLASTp

XP_014287090.1 clathrin heavy chain [*Halyomorpha halys*]

Score:3485 bits

E-value: 0.0

Query 1 MSQILPIRFQEHLQLSSVGINPTSVGFNTLTMESDKFICVREKVGETSQVVIIDLNDTAN 60

MSQILPIRFQEHLQLSSVGINPTSVGFNTLTMESDKFICVREKVGETSQVVIIDLNDTAN

Sbjct 1 MSQILPIRFQEHLQLSSVGINPTSVGFNTLTMESDKFICVREKVGETSQVVIIDLNDTAN 60

Query 61 PIRRPISADSAIMNPASKVIALKGKAGPDGTTTQKTLQIFNIEMRSKMKAHTMTDEVVFW 120

PIRRPISADSAIMNPASKVIALKGKAG DGTTTQKTLQIFNIEMRSKMKAHTMTDEVVFW

Sbjct 61 PIRRPISADSAIMNPASKVIALKGKAGTDGTTTQKTLQIFNIEMRSKMKAHTMTDEVVFW 120

Query 121 KWISLNTLALVTETSVYHWSMEGDSQPVKMFDRHSSLNGCQIINYRTDPKQTWLLLIGIS 180

KWISLNTLALVTETSVYHWSMEGDSQPVKMFDRHSSLNGCQIINYRTDPKQTWLLLIGIS

Sbjct 121 KWISLNTLALVTETSVYHWSMEGDSQPVKMFDRHSSLNGCQIINYRTDPKQTWLLLIGIS 180

Query 181 AQLNRVVGAMQLYSVERKCSQPIEGHAASFAQFKMEGNPDVSTLFCFAVRTLQGGKLHII 240

AQ NRVVGAMQLYSVERKCSQPIEGHAASFAQFKMEGNPDVSTLFCFAVRT GGKLHII

Sbjct 181 AQHNRVVGAMQLYSVERKCSQPIEGHAASFAQFKMEGNPDVSTLFCFAVRTPTGGKLHII 240

Query 241 EVGQPPTGNQPFSKKAVDVFFPVEAQNDFPVAMQVSSKYDVIYLITKYGYIHLYDLETAT 300

EVGQPP+GNQPF+KKAVDVFFPVEAQNDFPVAMQVSSKYDVIYLITKYGYIHLYDLETAT

Sbjct 241 EVGQPPSGNQPFAKKAVDVFFPVEAQNDFPVAMQVSSKYDVIYLITKYGYIHLYDLETAT 300

Query 301 CIYMNRISIDTIFVTAPHESTGGIIGVNRKGQVLSVSVEEDHIIPYINNILQNPDLALRM 360

CIYMNRISIDTIFVTAPHESTGGIIGVNRKGQVLSVSVEEDHIIPYINNILQNPDLALRM

Sbjct 301 CIYMNRISIDTIFVTAPHESTGGIIGVNRKGQVLSVSVEEDHIIPYINNILQNPDLALRM 360

Query 361 AVRNNLAGAEDLFVKKFNMLFQNGQYAEAAKVAANAPKGILRTPQTIQQFQQIPNPQGQT 420

AVRNNLAGAEDLFVKKFNMLFQNGQYAEAAKVAANAPKGILRTPQTIQQFQQIPNPQGQT

Sbjct 361 AVRNNLAGAEDLFVKKFNMLFQNGQYAEAAKVAANAPKGILRTPQTIQQFQQIPNPQGQT 420

Query 421 SPLLQYFGILLDQGQLNKYESLELCRPVLAQGRKQLLEKWLKEDKLECSEELGDLVKQTD 480

SPLLQYFGILLDQGQLNKYESLELCRPVLAQGRKQL+EKWLKEDKLECSEELGDLVKQTD

Sbjct 421 SPLLQYFGILLDQGQLNKYESLELCRPVLAQGRKQLMEKWLKEDKLECSEELGDLVKQTD 480

Query 481 PTLALSVYLRANVPNKVIQCFAETGQFQKIVLYAKKVGYSPDYIFLLRNVMRVNPDQGVS 540

PTLALSVYLRANVPNKVIQCFAETGQFQKIVLYAKKVGYSPDYIFLLRNVMRVNPDQGVS

Sbjct 481 PTLALSVYLRANVPNKVIQCFAETGQFQKIVLYAKKVGYSPDYIFLLRNVMRVNPDQGVS 540

Query 541 FAQMLVQDEEPLADINQIVDILMEQNMVQQCTAFLLDALKNNRPSEGPLQTRLLEMNLIS 600

FAQMLVQDEEPLADINQIVDILMEQNMVQQCTAFLLDALKNNRPSEGPLQTRLLEMNLIS

Sbjct 541 FAQMLVQDEEPLADINQIVDILMEQNMVQQCTAFLLDALKNNRPSEGPLQTRLLEMNLIS 600

Query 601 APQVADAILGNQMFTHYDRAHIAQLCEKAGLLQRALEHYTDLYDIKRAVVHTHLLGPDWL 660

APQVADAILGNQMFTHYDRAHIAQLCEKAGLLQRALEHYTDLYDIKRAVVHTHLLGPDWL

Sbjct 601 APQVADAILGNQMFTHYDRAHIAQLCEKAGLLQRALEHYTDLYDIKRAVVHTHLLGPDWL 660

Query 661 VGYFGTLSVEDSLECLKAMLTANIRQNLNICVQIATKYHEQLTTKALIDLFESFKSYEGL 720

VGYFGTLSVEDSLECLKAMLTANIRQNLNICVQIATKYHEQLTTKALIDLFESFKSYEGL

Sbjct 661 VGYFGTLSVEDSLECLKAMLTANIRQNLNICVQIATKYHEQLTTKALIDLFESFKSYEGL 720

Query 721 FYFLGSIVNFSQDQEVHFKYIQAACKTGQIKEVERICRESNCYNPERVKNFLKEAKLTDQ 780

FYFLGSIVNFSQDQEVHFKYIQAACKTGQIKEVERICRESNCYNPERVKNFLKEAKLTDQ

Sbjct 721 FYFLGSIVNFSQDQEVHFKYIQAACKTGQIKEVERICRESNCYNPERVKNFLKEAKLTDQ 780

Query 781 LPLIIVCDRFDFCHDLVLYLYRNNLQKYIEIYVQKVNPSRLPVVIGGLLDVDCSEDIIKN 840

LPLIIVCDRFDFCHDLVLYLYRNNLQKYIEIYVQKVNPSRLPVVIGGLLDVDCSEDIIKN

Sbjct 781 LPLIIVCDRFDFCHDLVLYLYRNNLQKYIEIYVQKVNPSRLPVVIGGLLDVDCSEDIIKN 840

Query 841 LMMVVRGQFSTEELVAEVEKRNRLKLLLPWLETRVHEGCVEPATHNALAKIYIDSNNNPE 900

LMMVVRGQFSTEELVAEVEKRNRLKLLLPWLETRVHEGCVEPATHNALAKIYIDSNNNPE

Sbjct 841 LMMVVRGQFSTEELVAEVEKRNRLKLLLPWLETRVHEGCVEPATHNALAKIYIDSNNNPE 900

Query 901 RFLRENQYYDSCVVGKYCEKRDPHLACVAYERGQCDRELINVCNENSLFKSEARYLVRRK 960

RFLRENQYYDSCVVGKYCEKRDPHLACVAYERGQCDRELINVCNENSLFKSEARYLVRRK

Sbjct 901 RFLRENQYYDSCVVGKYCEKRDPHLACVAYERGQCDRELINVCNENSLFKSEARYLVRRK 960

Query 961 DPELWAEVLNENNPYKRPLIDQVVQTALSETQDPEDISVTVKAFMTADLPNELIELLEKI 1020

DPELWAEVLNENNPYKRPLIDQVVQTALSETQDPEDISVTVKAFMTADLPNELIELLEKI

Sbjct 961 DPELWAEVLNENNPYKRPLIDQVVQTALSETQDPEDISVTVKAFMTADLPNELIELLEKI 1020

Query 1021 VLDNSIFSDHRNLQNLLILTAIKADRSRVMEYINRLDNYDAPDIANIAINYQLYEEAFAI 1080

VLDNSIFSDHRNLQNLLILTAIKADRSRVMEYINRLDNYDAPDIANIAINYQLYEEAFAI

Sbjct 1021 VLDNSIFSDHRNLQNLLILTAIKADRSRVMEYINRLDNYDAPDIANIAINYQLYEEAFAI 1080

Query 1081 FKKFDVNTSAIQVLIENVNNLDRAYEFAERCNEPAVWSQLAKAQLQHGLVKEAIDSFIKA 1140

FKKFDVNTSAIQVLIENVNNLDRAYEFAERCNEPAVWSQLAKAQLQHGLVKEAIDSFIKA

Sbjct 1081 FKKFDVNTSAIQVLIENVNNLDRAYEFAERCNEPAVWSQLAKAQLQHGLVKEAIDSFIKA 1140

Query 1141 DDPSAYVDVVETAHKTESWEDLVRYLQMARKKARESYIESELIYAYARTNRLADLEEFIS 1200

DDPSAYVDVVETAHKTESWEDLVRYLQMARKKARESYIESELIYAYARTNRLADLEEFIS

Sbjct 1141 DDPSAYVDVVETAHKTESWEDLVRYLQMARKKARESYIESELIYAYARTNRLADLEEFIS 1200

Query 1201 GPNHADIQKIGDRCFDDKMYEPAKLLYNNVSNFARLAITLVHLKEFQGAVDSARKANSTR 1260

GPNHADIQKIGDRCFDDKMYEPAKLLYNNVSNFARLAITLVHLKEFQGAVDSARKANSTR

Sbjct 1201 GPNHADIQKIGDRCFDDKMYEPAKLLYNNVSNFARLAITLVHLKEFQGAVDSARKANSTR 1260

Query 1261 TWKEVCFACVDSEEFRLAQMCGLHIVVHADELEDLINYYQDRGYFEELINLLEAALGLER 1320

TWKEVCFACVDSEEFRLAQMCGLHIVVHADELEDLINYYQDRGYFEELINLLEAALGLER

Sbjct 1261 TWKEVCFACVDSEEFRLAQMCGLHIVVHADELEDLINYYQDRGYFEELINLLEAALGLER 1320

Query 1321 AHMGMFTELAILYSKYKPGKMREHLELFWSRVNIPKVLRAAEQAHLWAELVFLYDKYEEY 1380

AHMGMFTELAILYSKYKP KMREHLELFWSRVNIPKVLRAAEQAHLWAELVFLYDKYEEY

Sbjct 1321 AHMGMFTELAILYSKYKPAKMREHLELFWSRVNIPKVLRAAEQAHLWAELVFLYDKYEEY 1380

Query 1381 DNAVSAMMNHPTEAWREGHFKDIITKVANIELYYKAIQFYLDYKPLLLNDMLLVLAPRMD 1440

DNAVSAMMNHPTEAWREGHFKDIITKVANIELYYKAIQFYLDYKPLLLNDMLLVLAPRMD

Sbjct 1381 DNAVSAMMNHPTEAWREGHFKDIITKVANIELYYKAIQFYLDYKPLLLNDMLLVLAPRMD 1440

Query 1441 HTRAVNFFAKVNHLQLVKPYLRSVQSLNNKAINEALNNLLIEEEDFQGVRTSIDAFDNFD 1500

HTRAVNFFAKVNHLQLVKPYLRSVQSLNNKAINEALNNLLIEEEDFQGVRTSIDAFDNFD

Sbjct 1441 HTRAVNFFAKVNHLQLVKPYLRSVQSLNNKAINEALNNLLIEEEDFQGVRTSIDAFDNFD 1500

Query 1501 NIVLAQKLEKHELIEFRRIAAYLYKGNNRWKQSVQLCKKDRLFKDAMEYAAESKNSEVAE 1560

NIVLAQKLEKHELIEFRRIAAYLYKGNNRWKQSVQLCKKDRLFKDAMEYAAESKNSEVAE

Sbjct 1501 NIVLAQKLEKHELIEFRRIAAYLYKGNNRWKQSVQLCKKDRLFKDAMEYAAESKNSEVAE 1560

Query 1561 ELLAWFLEKGNHDCFAACLFQCYDLLHPDVILELAWRHNIMDFAMPYIIQVTREYISKVD 1620

ELLAWFLEKGNHDCFAACLFQCYDLLHPDVILELAWRHNIMDFAMPYIIQVTREYISKVD

Sbjct 1561 ELLAWFLEKGNHDCFAACLFQCYDLLHPDVILELAWRHNIMDFAMPYIIQVTREYISKVD 1620

Query 1621 KLEESEARRLEERAEQDHKPMMMPEPQLMLTAGPGMIGPGFSPAYPNTFTTGMPYQGYGM 1680

KLEESEARRLEERAEQDHKPMMMPEPQLMLTAGPGMIGPGFSPAYPNTFTTGMPYQGYGM

Sbjct 1621 KLEESEARRLEERAEQDHKPMMMPEPQLMLTAGPGMIGPGFSPAYPNTFTTGMPYQGYGM 1680

AP2U (AP50)

>TRINITY_DN5859_c0_g1_i2 len=3782 path=[1:0-258 2:259-2371 4:2372-2961 6:2962-3781]

GTAGGTGTTCCTTATTGATTAAGATGTGGACCGCATTTTCTCTACTATTTTAAATAAAATCTTTATATTCGTA
CGTTTTTGTGTTTCCGAATCGTCAAATTTAGAGTGGTAATGCTTATTTGCACATCTCTTTTAGAACTGACCCA
TAAACAATGCAGGGTTTAACGTTAACTGATAAACGATTCGCCTGTAGTTCAGCTACATATCTGATTTAATAA
TGACGTTATTTTAACTCTTGAGACATTTAGAATGAAATAAAGGATCTCATTAATACTTAACAAAATGATTGG
AGGATTGTTTGTGTACAATCATAAAGGAGAAGTGCTTATTTCTCGAGTGTACAGGGATGATATTGGCCGCA
ATGCAGTTGATGCGTTCCGAGTCAATGTCATTCATGCCAGGCAACAGGTGAGATCTCCTGTTACAAACATT
GCTAGAACATCTTTCTTCCATATAAAGAGGGCAAATATATGGCTCGCAGCAGTCACTAAGCAAAATGTCAA
TGCAGCAATGGTTTTTGAATTTCTTCTCAAAGTTATTGATGTTATGCAGTCTTACTTTGGAAAAATATCAGA
AGAAAACATCAAAAACAATTTTGTTCTTATCTATGAACTCCTTGATGAAATCTTGGACTTTGGTTATCCGCA
AAACTCCGACACTGGCGTATTAAAGACATTCATTACACAACAAGGCGTTAAATCTCAGTCGAAAGAAGAGC
AGGCACAGATCACATCTCAAGTTACCGGGCAGATTGGCTGGCGCAGGGAAGGGATTAAGTACCGCAGGAA
TGAACTATTTTTAGACGTCTTGGAATTCGTCAATCTTCTCATGTCTCCTCAAGGTCAAGTGCTGTCTGCTCAT
GTCGCCGGTAAGGTTGTTATGAAGTCTTACCTTTCAGGGATGCCAGAATGCAAATTCGGAATCAATGATAA
GATTGTAATGGAATCAAAAGGTAAAAGTAGCGGGAGTGGTATATCTGTGGCTGGCGGAGACGATGGTGCA
CGTTCCGGTAAGCCAGTGGTTGTTATTGATGATTGCCAGTTCCATCAGTGTGTCAAGTTATCGAAATTCGAA
ACCGAACATTCCATCTCTTTCATTCCTCCTGATGGAGAATTTGAGCTAATGAGGTACAGAACGACTAAGGA
CATATCCCTGCCATTCCGTGTGATCCCCCTGGTGAGGGAGGTGGGGAGGACCAGGATGGAGGTGAAGGTCG
TCCTCAAGTCCAACTTCAAGCCCTCCCTCCTCGGGCAGAAGATGGAAGTGCGCATCCCGACGCCGCTCAAC
ACCTCCGGCGTCCAGTTGATCTGCCTCAAGGGGAAGGCAAAGTACAAGGCTTCGGAGAATGCTATTGTCTG
GAAGATAAAACGAATGGCTGGTATGAAGGAAACTCAGTTATCTGCTGAAATTGAATTGTTAGAAACTGACA
CTAAGAAGAAATGGACACGTCCTCCAATATCCATGAATTTCGAGGTACCATTTGCTCCATCCGGGTTCAAA
GTCCGATATTTGAAGGTATTCGAGCCGAAGCTCAACTATTCCGACCACGACGTTATCAAGTGGGTGAGGTA
CATCGGACGAAGTGGTCTCTACGAAACCCGTTGCTGAATCCTCAACATCTCCGTGCGCCTCTCTCTGTCCCT
CTTCTCCAATCACCTTTCTTCTTTTTTTGTTTTTATACTCAGGTATCCATTACGTTGTTTCACACCTTATCAT
AAGCCTCATCGGGTGAATATCGAATAGTTTACAGCCATTTTTTTTATTAGTTTAGGTTATAGATACCTGTAT

TATATTACGCACTTAACCTGAAGATTGATAGTAGAGAGGAATACATCTTGCAATTCTTATTGCACAAAACCT
TTGCCGAGACAATAACTGTTGTAATTATATTAATTTTTATAGGGGAATAGGAAGTGAAGAATATCGTAGTC
GAAACATTTGGGGGAAGTTATATCATCTTCCTTATGTTTCCTATTATATTCAACGTCTGTTTCCAAGACTGCC
ATGGCATTCCTAAAAGGTCTCAAAAGTAACAATTAAAACATTAATATTCTATTAGCTGTTAATGAAATTACT
CGGCATTCCTTTCTTTAAAAAAATTACCATTTTATTTATTTCTATTGGAACAATTAATTCCAATATGTTGATC
TTCTTTTATTTTTATTATTTAATTTATACTCCATTTATTTTTATTTATCATGTTACTCCATTTGTGTTATTGGAT
TTTAATAATGAAATAATATTTAAATAATGGGTTAAAAATTGATTTTTGTATTATTTTAAACTATTTTGGCAGT
CGAAATTATTCATGCTTTATGTACTCAAAAATGATTTTTATTGTTATTATTAAATCAAGGATGGAAAAAAAA
AGTTTTAAGAAATGTACAAATAAGATGGGAAACGTGCCCAGAAAGTTCATTAATATGAGAAGCTCTCCCAT
GACCAAATATCGATTTTTAATAAGTATCAGTATACCAAGTGTATTGAGATTATTTTTCTTTTCTTTTCTTTTTT
TTTTTTTTCAAGTTTTAGATTATATAATCTATTTATTTTTTTCTTTAGTATATGTTCTTGCAGGTTAAGAACGA
AATAAATAAATAAAAAGCCATTAATGCACTACTGTTGTAAAAAAAATAAATAAAATGAATCTCTTACCTTA
ATATTATTATTATCATTTAATTTATATGAACTTTTCATAAAGTCGTAGATGTTAATTGATAACTTATTTAAAA
TACTTAGGAATAAAACACATTTTCTTTCGTATTCAGTATTAAATTACAAAGAGTTTTATATTTTTCCCCATCC
GATATTAATAAAATATTTACAGAAAATAATTAAAGATAAATGTTAGTGATAAATTAGTAAGCTTTGTGATG
TGGAAGAATATTATCATCGATTCTTTGTTTTGTTTTTTAAATTTCCGTGTGTAAAATCAGCTGTTTAGGAAGA
AATATATATATGTATTTGTAGGAAGAATTGATGATGCTTTATTATCTTCTGAAAAATTGTTAATTGTTGTGTT
TAATTGAATATTTATCAATTTATGATTTATTACGATGATTATGCATGGAAATTTTATGTTTTATATTTGTTAT
ATTATATACAAGAAAAAGTTATATCTGTATATGTATATATTATCAAGATCTGTACTTTATTATTGCGGGAGT
ACGATGAATTCTTTCGTCTCTCTGTGGGTATTAATTTGGACATTGTCACAAATTTGAAAGACAAATATTACT
CAATAGGCAAATATTTATAGAAAATTATATAATTTATGTGATTTGAATTATATTTAATGTGAAAATAAGAAA
AAAAAACTTAGTATTTAGGAAATATACCTACTTGTAAATGGTCACCCAACCAAGATATCCTTTTTTTTTTTTC
TTTTTTAGTTGAAAACTATTCCTGTTTTCAAATTATTCACTCACCAACAATTTTTAAAGATTCATTTAGAAAT
ATGATTTCAGTTTTAATAGTTAGGTGTTTCATGGGTGTGATTAAATAATGATGACTATTAAATAGCCTTGCA
CTAGAAGTGTGTCAAAAATATAATTACTGGGGATTCTTTTTTTTTATGATCGATTTATATTGAAAAATTATA
CCTTATAATTTAGGGTGCCATTATGCCTGTATCATTTTAATTGGTATTATTTGTTAATGATATGTATTATTCT
ATCCCAATGTAATATCACAGTTTATCTATTCCTATGTAAATTAATCTGACTGCTTTTCGTAACCAAATATAAT
TTAATTATTTTCAAAAAAAAAACAACAAAAAAT

Protein

RF: +1

ORF: 283 -> 1608

Length: 441 aa

>lcl|ORF1

MIGGLFVYNHKGEVLISRVYRDDIGRNAVDAFRVNVIHARQQVRSPVTNIARTSFFHIKRANIWLAAVTKQNVN
AAMVFEFLLKVIDVMQSYFGKISEENIKNNFVLIYELLDEILDFGYPQNSDTGVLKTFITQQGVKSQSKEEQAQIT
SQVTGQIGWRREGIKYRRNELFLDVLEFVNLLMSPQGQVLSAHVAGKVVMKSYLSGMPECKFGINDKIVMESK
GKSSGSGISVAGGDDGARSGKPVVVIDDCQFHQCVKLSKFETEHSISFIPPDGEFELMRYRTTKDISLPFRVIPLVR
EVGRTRMEVKVVLKSNFKPSLLGQKMEVRIPTPLNTSGVQLICLKGKAKYKASENAIVWKIKRMAGMKETQLS
AEIELLETDTKKKWTRPPISMNFEVPFAPSGFKVRYLKVFEPKLNYSDHDVIKWVRYIGRSGLYETRC

Conserved Domains

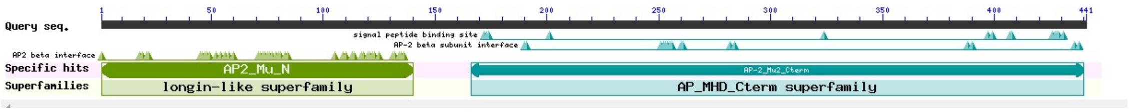

BLASTp

NP_001280510.AP-2 complex subunit mu [Tribolium castaneum]

Score:866 bits

E-value: 0.0

Query 1 MIGGLFVYNHKGEVLISRVYRDDIGRNAVDAFRVNVIHARQQVRSPVTNIARTSFFHIKR 60
MIGGLFVYNHKGEVLISRVYRDDIGRNAVDAFRVNVIHARQQVRSPVTNIARTSFFHIKR
Sbjct 1 MIGGLFVYNHKGEVLISRVYRDDIGRNAVDAFRVNVIHARQQVRSPVTNIARTSFFHIKR 60

Query 61 ANIWLAAVTKQNVNAAMVFEFLLKVIDVMQSYFGKISEENIKNNFVLIYELLDEILDFGY 120
ANIW+AAVTKQNVNAAMVFEFLLK+I+VMQSYFGKISEENIKNNFVLIYELLDEILDFGY
Sbjct 61 ANIWIAAVTKQNVNAAMVFEFLLKIIEVMQSYFGKISEENIKNNFVLIYELLDEILDFGY 120

Query 121 PQNSDTGVLKTFITQQGVKSQSKEEQAQITSQVTGQIGWRREGIKYRRNELFLDVLEFVN 180
PQN+DTGVLKTFITQQG+KS +KEEQAQITSQVTGQIGWRREGIKYRRNELFLDVLE+VN
Sbjct 121 PQNTDTGVLKTFITQQGIKSATKEEQAQITSQVTGQIGWRREGIKYRRNELFLDVLEYVN 180

Query 181 LLMSPQGQVLSAHVAGKVVMKSYLSGMPECKFGINDKIVMESKGKSSGSGISVAGGDDGA 240
LLMSPQGQVLSAHVAGKVVMKSYLSGMPECKFGINDKIVME+KGK G+ D
Sbjct 181 LLMSPQGQVLSAHVAGKVVMKSYLSGMPECKFGINDKIVMEAKGK---GGLGSTSDSDQT 237

Query 241 RSGKPVVVIDDCQFHQCVKLSKFETEHSISFIPPDGEFELMRYRTTKDISLPFRVIPLVR 300
RSGKPVVVIDDCQFHQCVKLSKFETEHSISFIPPDGEFELMRYRTTKDISLPFRVIPLVR
Sbjct 238 RSGKPVVVIDDCQFHQCVKLSKFETEHSISFIPPDGEFELMRYRTTKDISLPFRVIPLVR 297

Query 301 EVGRTRMEVKVVLKSNFKPSLLGQKMEVRIPTPLNTSGVQLICLKGKAKYKASENAIVWK 360
EVGRT+MEVKVVLKSNFKPSLLGQK+EV+IPTPLNTSGVQLICLKGKAKYKASENAIVWK
Sbjct 298 EVGRTKMEVKVVLKSNFKPSLLGQKIEVKIPTPLNTSGVQLICLKGKAKYKASENAIVWK 357

Query 361 IKRMAGMKETQLSAEIELLETDTKKKWTRPPISMNFEVPFAPSGFKVRYLKVFEPKLNYS 420
IKRMAGMKETQLSAEIELLETDTKKKWTRPPISMNFEVPFAPSGFKVRYLKVFEPKLNYS
Sbjct 358 IKRMAGMKETQLSAEIELLETDTKKKWTRPPISMNFEVPFAPSGFKVRYLKVFEPKLNYS 417

Query 421 DHDVIKWVRYIGRSGLYETRC 441
DHDVIKWVRYIGRSGLYETRC
Sbjct 418 DHDVIKWVRYIGRSGLYETRC 438

ADP-Ribosylation factor-like protein 1 (ARL1)

>TRINITY_DN8242_c0_g1_i1 len=1355 path=[0:0-351 2:352-569 4:570-1354]

CTGTAAACAACCGAAATGTCAAAATGTTCTGTTATCAGCAAAGGTGCCTGAATAGTATTATTAAGCTCTTCA
ACCACGTCAGATAATTCGAATTTGTGAGATAATTAGAAGAACAATCTCGATTTTCATCTAAGTTCATTATTT
AAAATATTCCGTTTTATAGAATGATAATATAATCATATTCTTTGTTGAATTCATGGTCAATTGAAAATGAAA
GTTTTCAAGATCTTTGAACCAAGTGCGTTACTCAGGTAATCATGTGGTGCATTGATAATCTTTTAGGAAAGA
GGTGTCAGTGTTTCGCCTTTTGAATTGATGTTATACATATCGTAGACTTATCTCATTAGCCATGGGTGGATTA
TTAAGTTACTTTAGAAACCTTTTAGGAAGCCGAGAAATGAGAATATTAATCTTAGGTTTAGATGGTGCAGG
TAAAACCACAATTTTGTATCGTTTACAAGTTGGTGAAGTTGTTACGACTATTCCGACTATTGGTTTCAATGT
GGAACAAGTGACTTACAAAAATCTCAAGTTCCAAGTTTGGGATCTCGGTGGTCAAACGAGTATAAGACCTT
ATTGGCGGTGTTATTTTCAAATACAGATGCAATAATTTATGTGGTGGATTCTGCTGATAAGGATCGAATAG
GAATATCTAAGGATGAACTTTTCTATATGTTAAAAGAAGAAGAACTGCAAGGCGCTATTCTGGTTGTTCTTG
CCAACAAGCAGGATATGGAAGGTTGCTTGAGTGTTGCAGAGGTTCATCAGGCCCTCGGTTTAGACCAATTG
AAGAATAGAACATTCCAAATTTTCAAGACTTCTGCAGTTAAAGGAGAAGGATTAGATTCTGCAATGGACTG
GCTTTCAAATGCACTTCAAAATAAAAAGTGAAATTTTTAATAAGTGGATAAAGACAAAATTAAGTCTTAAA
AATAAATGGACTATTTTTTACTTTGGGGAGGAAGTTGAACAGAGCAGCTCATGGCTCTATTTCTCTTTGTTA
TGACTATAAAGTGTACAGTAATAATAGTCTTAGTTAAATTGGCGAACCTCTCGACCCTATCTAGGGAAAAG
GCTGATACAATTGAATTTTCATGTTGTAGAACATGAATCCATTATAAGATTTTTATTTAGTGGTTTTAATTGC
TCTTATATATACATATATATTTATGACAAATAATTATAAATTTTTTCTATTTATTATGGTGTTCAATATACAT
ATATCTATACATAAATATATATTTATATATTTATTTATAAATATATTTTCCCCTACATTATTATGCCACAATA
TACATAATAAACATAGATTAGAAAAACCACTAAGCTTCTTGTGCATATGTCTATTTCGTGC

Protein

<u>RF:</u> +2

<u>ORF:</u> 350 -> 892

<u>Length</u>: 180 aa

>lcl|ORF2

MGGLLSYFRNLLGSREMRILILGLDGAGKTTILYRLQVGEVVTTIPTIGFNVEQVTYKNLKFQVWDLGGQTSIRP
YWRCYFSNTDAIIYVVDSADKDRIGISKDELFYMLKEEELQGAILVVLANKQDMEGCLSVAEVHQALGLDQLK
NRTFQIFKTSAVKGEGLDSAMDWLSNALQNKK

Conserved Domains

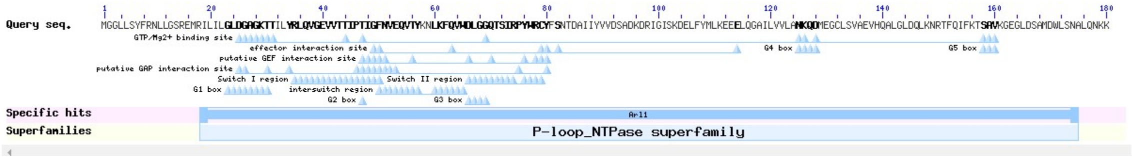

BLASTp

XP_973025.1ADP-ribosylation factor-like protein 1 [Tribolium castaneum]

Score:323 bits

E-value: 8e-120

```
Query  1    MGGLLSYFRNLLGSREMRILILGLDGAGKTTILYRLQVGEVVTTIPTIGFNVEQVTYKNL  60
            MG L SYF++LLG+REMRILILGLDGAGKTTILYRLQVGEVVTTIPTIGFNVEQV YKNL
Sbjct  1    MGVLFSYFKSLLGAREMRILILGLDGAGKTTILYRLQVGEVVTTIPTIGFNVEQVQYKNL  60

Query  61   KFQVWDLGGQTSIRPYWRCYFSNTDAIIYVVDSADKDRIGISKDELFYMLKEEELQGAIL  120
             FQVWDLGGQTSIRPYWRCY+SNTDAIIYVVDSADK+R+GISKDELF+ML+EEEL  AIL
Sbjct  61   NFQVWDLGGQTSIRPYWRCYYSNTDAIIYVVDSADKERMGISKDELFHMLREEELGDAIL  120

Query  121  VVLANKQDMEGCLSVAEVHQALGLDQLKNRTFQIFKTSAVKGEGLDSAMDWLSNALQNKK  180
            VVLANKQD+ GC+S+ EVHQALGL+ LKNRTFQIFKTSA KGEGLD AM+WL+NAL+NKK
Sbjct  121  VVLANKQDIPGCMSLKEVHQALGLEALKNRTFQIFKTSATKGEGLDMAMEWLANALKNKK  180
```

Epsin 2 (Epn2)

>TRINITY_DN2686_c2_g1_i4 len=1957 path=[2:0-633 3:634-666 4:667-1129 6:1130-1480 7:1481-1757 8:1758-1956]

TATCGCCTGGCTTTGCTGATTTGTCGAAATTGAATGAGTACTGTCATTCTTATCTTTATGTGTGTAAATTGTT
TTTAAAAACTCAGAAAAGGTTAAAATCTTAAAATCCAAGATGCGGCGGCGTGAAGACATGCAGGTGAATG
TGGCAGGGCTTCGGCGGAATATAAAAAATTTAGCCCATAATTACTCTGATGCTCAGATAAAGGTACGAGAG
GCAACGAGTAACGATCCATGGGGTCCTAGTAGCACGCTCATGTCTGAAATTGCTGACCTAACATACAACGT
AGTCGCTTTCACAGAAATAATGCAAATGATATGGAAACGCCTGAACGATCACGGTAGAAATTGGAGACAT
GTGTATAAAGCGCTATTACTCTTGGAGTATCTCATTAAAACTGGTTCCGAAAAGGTCGGCCAGCAGTGTAA
AGAAAATATTTTCGCAATCCAAACTCTTCGGGATTTCCAGTATTCGGACGAGGGTAAAGATCAAGGGGTCA
ACGTAAGAGAAAAGGCAAAGCAGCTGGTGTCACTTTTAAAAGATGAAGAAAGATTACGCAATGAGCGTGC
ACGAGCGCTCAAAGCTAAAGAACGATTCGCTCAAACGGCGTCAGGATTCGGCAGTGACACAACCTTGGAT
GGTGCTTCACCTTCCAGCCCTACGTTTCAATCGAGATCAGCTTGGAGTTCCAGTGATGTTGAGACTCCCAGT
AAAGTAGATATTATGTGTGCGAGGCCGACAACAGCTGGCGAAGAAGAATTGCAGCTTCAGCTGGCTCTTGC
GATGTCGAGGGAAGAAGCTGAACAAGAGGAACAGAAAAGGAGGAGTGACGATGTTCGCCTCCAACTAGC
AATCTCTCAGAGTCAGAATGAATTCAAACAACAGCAACAGGAGGTTGAAAAGGAGCCGAGCGGTAGCCAC
ATGCTGGACCTCCTTGACGTGAATCTGGGCCCCATCGATAGCGGCCCGACCACCTCCGCCCCCACCCCTCTC
CACGACCCCTGGGGGATGCCCCTCCCTCCTTCCCGTCCCCAGGTTGATCCATGGAGTGTTTCATCTTCAACG
GCAACAAACCCTTCCGTTGCCGATCCTTGGTCTCCCGCTCCAACCGTTTCTGCGCCTCCTAAGCCAAACGAG
GCTGCCTCTGTGGACCCGTGGAAGGCAGTGAAGCCGCAGAACGATCCTTGGTCCCCCGCGACGGAGCCGA
GCCCAGCGCTATCATCTCCGTTAAATGACCTTGACGAGTTTGACATTATAACTAATAGGAATAACATCAAC
AATAATAATAAAACATGCATTAGCCCTAATCCTTTTGACATGAGAGGGCTTGGAGAGAGCCTGAGTGATAG
GAATGGACTGGGCTCATCCAAGAGGAGGAGTCCCCACGCGTTCTTAGGCGAGAATTCTGGATTGGTCAATC
TAGACAATCTTGTCACAAAACCTACTCAGCCTCAACCGACTCAGCAGCCACCGTTACGACCAGTGGCAGCA
AACCCTTTCTCGCCAGTGGGTGCCCCGTTGCAGCCAGTTCGCAACACACCCGCCTTTGCACCTCAGGCAATA
TCTCAACCTCTCGTACCTCAACCTAATCCTTTCTTGTCATAGGGGCGTCATTTCACCCTGCGTCGAAACCAA
CACAACGGTCAACTTTCAAAAATCCATTACAATCACCAAACTATTCTCTTTTTTTCAATTTAAAAAAAAAAA

AAAAGTATAAGCTACTATTTTCCACGTGAGTGAAAGCAATAAAAATAAAAAGCAAAAAAAAAAAAAAAAA
GGAGAAAAAAAAAGAAAGCAAAGTCTGGGAAACAATCTGTTTTCTTTGGTCACTCGCAAAACGTATTATCAA
TCTCTGTGCCTCGTAATATAAATGTTATATATATATATATATATATCCAATGTTTGTCGCCCGTTTATATTTG
AGTTGTATTAATTAGCTCTTTTAGAATGGTGCAGTATT

Protein

RF: +2

ORF: 113 -> 1603

Length: 496 aa

>lcl|ORF2_TRINITY_DN2686_c2_g1_i4:112:1602 unnamed protein product

MRRREDMQVNVAGLRRNIKNLAHNYSDAQIKVREATSNDPWGPSSTLMSEIADLTYNVVAFTEIMQMIWKRL
NDHGRNWRHVYKALLLLEYLIKTGSEKVGQQCKENIFAIQTLRDFQYSDEGKDQGVNVREKAKQLVSLLKDEE
RLRNERARALKAKERFAQTASGFGSDTTLDGASPSSPTFQSRSAWSSSDVETPSKVDIMCARPTTAGEEELQLQL
ALAMSREEAEQEEQKRRSDDVRLQLAISQSQNEFKQQQQEVEKEPSGSHMLDLLDVNLGPIDSGPTTSAPTPLH
DPWGMPLPPSRPQVDPWSVSSSTATNPSVADPWSPAPTVSAPPKPNEAASVDPWKAVKPQNDPWSPATEPSPAL
SSPLNDLDEFDIITNRNNINNNNKTCISPNPFDMRGLGESLSDRNGLGSSKRRSPHAFLGENSGLVNLDNLVTKPT
QPQPTQQPPLRPVAANPFSPVGAPLQPVRNTPAFAPQAISQPLVPQPNPFLS

Conserved Domains

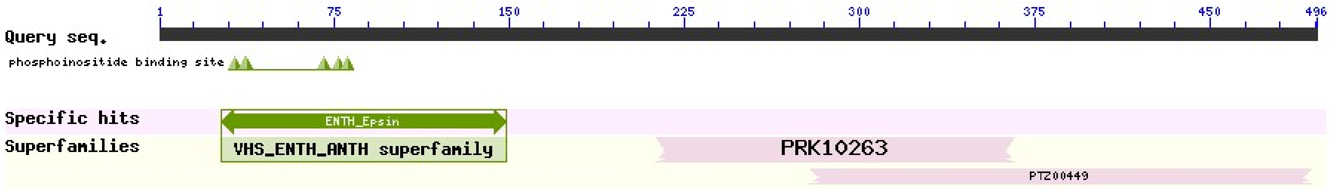

BLASTp

XP_014270392.1 epsin-2 isoform X5 [*Halyomorpha halys*]

Score:916 bits

E-value: 0.0

Query 14 MRRREDMQVNVAGLRRNIKNLAHNYSDAQIKVREATSNDPWGPSSTLMSEIADLTYNVVA 73

MRRREDMQVNVAGLRRNIKNLAHNYSDAQIKVREATSNDPWGPSSTLMSEIADLTYNVVA

Sbjct 1 MRRREDMQVNVAGLRRNIKNLAHNYSDAQIKVREATSNDPWGPSSTLMSEIADLTYNVVA 60

Query 74 FTEIMQMIWKRLNDHGRNWRHVYKALLLLEYLIKTGSEKVGQQCKENIFAIQTLRDFQYS 133

FTEIMQMIWKRLNDHGRNWRHVYKALLLLEYLIKTGSEKVGQQCKENIFAIQTLRDFQYS

Sbjct 61 FTEIMQMIWKRLNDHGRNWRHVYKALLLLEYLIKTGSEKVGQQCKENIFAIQTLRDFQYS 120

Query 134 DEGKNQGLNVREKAKQLVSLLKDEEKLRNERARALKAKERFAQTASGFGSDTTLDGASPS 193

DEGK+QG+NVREKAKQLVSLLKDEE+LRNERARALKAKERFAQTASGFGSDTTLDGASPS

Sbjct 121 DEGKDQGVNVREKAKQLVSLLKDEERLRNERARALKAKERFAQTASGFGSDTTLDGASPS 180

Query 194 SPTFQSRSAWSSSDVETPSKVDIMCARPTTAGEEELQLQLALAMSREEAEQEEQKRRSDD 253

SPTFQSRSAWSSSDVETPSKVDIMCARPTTAGEEELQLQLALAMSREEAEQEEQKRRSDD

Sbjct 181 SPTFQSRSAWSSSDVETPSKVDIMCARPTTAGEEELQLQLALAMSREEAEQEEQKRRSDD 240

Query 254 VRLQLAISQSQNEFKQQHHEVQKDQGGSHMLDLLDVNLGPLDGGPTTSAPVPLQDPWGMP 313

VRLQLAISQSQNEFKQQ EV+K+ GSHMLDLLDVNLGP+D GPTTSAP PL DPWGMP

Sbjct 241 VRLQLAISQSQNEFKQQQQEVEKEPSGSHMLDLLDVNLGPIDSGPTTSAPTPLHDPWGMP 300

Query 314 IPPSRPQTLDLRLNNWSSVDPWSVSSSTATNPSAADPWSPAPTASAPPKPNEAASVDPWK 373

+PPSRPQ   VDPWSVSSSTATNPS ADPWSPAPT SAPPKPNEAASVDPWK

Sbjct 301 LPPSRPQ-----------VDPWSVSSSTATNPSVADPWSPAPTVSAPPKPNEAASVDPWK 349

Query 374 TVKPQNDPWSPATEPSPALSSPLNDLDEFDIITNRNNINNNNKTCISPNPFDMRGLGESL 433

VKPQNDPWSPATEPSPALSSPLNDLDEFDIITNRNNINNNNKTCISPNPFDMRGLGESL

Sbjct 350 AVKPQNDPWSPATEPSPALSSPLNDLDEFDIITNRNNINNNNKTCISPNPFDMRGLGESL 409

Query 434 NDRNGLGSSKRRSPHAFLGENSGLVNLDNLVTKPTQPQQNQQQPLRPVAANPFSPVGAPL 493

+DRNGLGSSKRRSPHAFLGENSGLVNLDNLVTKPTQPQ QQ PLRPVAANPFSPVGAPL

Sbjct 410 SDRNGLGSSKRRSPHAFLGENSGLVNLDNLVTKPTQPQPTQQPPLRPVAANPFSPVGAPL 469

Query 494 QPVRSTPAFAPQAISQPLVPQPNPFLS 520

QPVR+TPAFAPQAISQPLVPQPNPFLS

Sbjct 470 QPVRNTPAFAPQAISQPLVPQPNPFLS 496

Gap Junction protein (Innexin2)

>TRINITY_DN2653_c0_g1_i1 len=1747 path=[0:0-1746]

GCGAGGTGTGGTACTAACAGTCCATCAGTGCAGGTTTGACTGAGAGAGTGCTTGTGTGTGTTCTAGTGGCTC
CAGTATGTTTGACGTGTTCGGCTCCCTCAAGGGGCTTCTGCGGATAGACACAGTATGCATCGACAACAACG
TCTTCCGGCTTCACTACAAAGCCACCGTCATCATCCTAGTGGCCTTCTCCCTGTTGGTCACCTCCAGGCAAT
ACATCGGAGACCCCATCGACTGCATCGTCGATGACATTCCCCTGAGCGTAATGGACACCTATTGTTGGATCT
ATTCGACTTTCACGATTCCGAACCGACTCACAGGTACAGTGGGAAAAGACGTCGTTCAACCTGGTGTTGCA
GGCCATGTCGAAGGTGAGGACGAAGTCAAATATCATAAATATTACCAGTGGGTGTGCTTCGTATTGTTTTTC
CAAGCGATACTGTTCTATGTGCCTCGTTACCTCTGGAAAACATGGGAAGGAGGAAGGATCAAGATGTTGGT
ACTCGATCTCAATTGCCCCGTTATAAATGAAGAATGTAAAGCCGACAGAAAGAAGCTTCTAGTCGACTATT
TCACATCCAATCTTCACACCCAGAATTTCTACGCCATCAGATTCTTTATTTGTGAAGCTCTGAATTTCATCAA
TGTTTTGGTTCAGATTTATTTCATGGACTTCTTTCTAGACGGAGAGTTTACAACATATGGATCGGACGTCGT
CAGGTTCACCGAAATGGAACCGGAAGAAAGAGAAGATCCTATGTCGAGAGTGTTTCCAAAAGTTACCAAA
TGTACCTTCCACAAATACGGTCCATCAGGATCCGTGCAGAAGTTTGATGGCTTGTGCGTCCTCCCGTTGAAC
ATCGTCAACGAAAAGATCTATGTCTTCTTGTGGTTTTGGTTTATCATCCTCACCATCCTATCCGCATTAGCGC
TGGTCTATCGCGTGGCAGTGGTCTGCGGGCCCCAGCTTCGCCTCTACCTCCTCCGCGCCAGGTCCAGGTTGT
CGCCCCAGGGCCAGATAGAGACAATAGCCAAGCACTGCCACCTTGGCGACTGGTTTGTTCTATACCAATTG
GGTAAGAATATTGACCCTCTCGTATTTAAAGAACTGATTGCAGATCTGGCGAAGAAGTTTGAAGGTAAAGA
GTCAGTTTAAGATATATTTGGTTTATTGTATTTTTGTAATACTAGTGCCTTAACTATGCCCTCTCGAGTAGGG
CTCTGTCCAGACGAATTAATGTGTTCAAAATGTGCATTAATTTCATTTTTGTAAATTGACAACATCCTTCTTT
TGGGGGCTTCAGGCACATGTGATTTTGCTTATTATTATATTTTGTTAATTTTTATCAAATGAAGTAATACTCC
TCCATAAATGCACTGCCAAAGCTATATGTCTTTCATTCATAGCTAGGAAGTAGCATATTATGAGAGCTAAC
GCACACATTTTATTTACAATTTTTGAAGACAATAATATACATTCCAAATTGAAACAATTGGCAATATTTAGG
TGTTCAAATTGTAAAATAATGCTCATGTTACTTTTAAAAGACTGTATTAAACTTTAACTGCTTCCTTATTGTT
TATAAAATTAATTTTTTATTACATATAAAGATTTTAAGATATTGTAGAAGTTAATATAATGTATCACTTCTAT
ATTTAAAATTAAGGTGCATACTATTTTTTTTTTTTTTTTTTAATATCGTATTGTGTAAGTACTACCTATTAGT
TGGTAGAACGAAAAGATTT

Protein

<u>RF:</u> +2

<u>ORF:</u> 77 -> 1156

<u>Length</u>: 359 aa

>lcl|ORF2_TRINITY_DN2653_c0_g1_i1:76:1155 unnamed protein product

MFDVFGSLKGLLRIDTVCIDNNVFRLHYKATVIILVAFSLLVTSRQYIGDPIDCIVDDIPLSVMDTYCWIYSTFTIP
NRLTGTVGKDVVQPGVAGHVEGEDEVKYHKYYQWVCFVLFFQAILFYVPRYLWKTWEGGRIKMLVLDLNCP
VINEECKADRKKLLVDYFTSNLHTQNFYAIRFFICEALNFINVLVQIYFMDFFLDGEFTTYGSDVVRFTEMEPEER
EDPMSRVFPKVTKCTFHKYGPSGSVQKFDGLCVLPLNIVNEKIYVFLWFWFIILTILSALALVYRVAVVCGPQLR
LYLLRARSRLSPQGQIETIAKHCHLGDWFVLYQLGKNIDPLVFKELIADLAKKFEGKESV

Conserved Domains

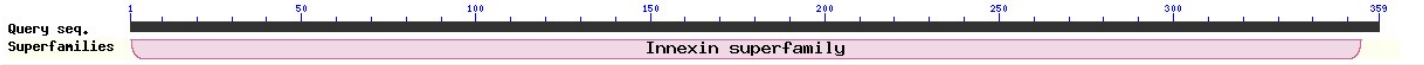

BLASTp

_XP_014292574.1 innexin inx2 [*Halyomorpha halys*]

Score:736 bits

E-value: 0.0

Query 1 MFDVFGSLKGLLRIDTVCIDNNVFRLHYKATVIILVAFSLLVTSRQYIGDPIDCIVDDIP 60

MFDVFGSLKGLLRIDTVCIDNNVFRLHYKATVIILVAFSLLVTSRQYIGDPIDCIVDDIP

Sbjct 1 MFDVFGSLKGLLRIDTVCIDNNVFRLHYKATVIILVAFSLLVTSRQYIGDPIDCIVDDIP 60

Query 61 LSVMDTYCWIYSTFTIPNRLSGTVGKDVVQPGVAGHVEGEDEVKYHKYYQWVCFVLFFQA 120

LSVMDTYCWIYSTFTIPNRL+GTVGKDVVQPGVAGHVEGEDEVKYHKYYQWVCFVLFFQA

Sbjct 61 LSVMDTYCWIYSTFTIPNRLTGTVGKDVVQPGVAGHVEGEDEVKYHKYYQWVCFVLFFQA 120

Query 121 ILFYVPRYLWKTWEGGRIKMLVLDLNCPVINEECKADRKKLLVDYFTSNLHTQNFYAIRF 180

ILFYVPRYLWKTWEGGRIKMLVLDLNCPVINEECKADRKKLLVDYFTSNLHTQNFYAIRF

Sbjct 121 ILFYVPRYLWKTWEGGRIKMLVLDLNCPVINEECKADRKKLLVDYFTSNLHTQNFYAIRF 180

Query 181 FICEALNFINVLVQIYFMDFFLDGEFTTYGSDVVRFTEMEPEEREDPMSRVFPKVTKCTF 240

FICEALNFINVLVQIYFMDFFLDGEFTTYGSDVVRFTEMEPEEREDPMSRVFPKVTKCTF

Sbjct 181 FICEALNFINVLVQIYFMDFFLDGEFTTYGSDVVRFTEMEPEEREDPMSRVFPKVTKCTF 240

Query 241 HKYGPSGSVQKFDGLCVLPLNIVNEKIYVFLWFWFIILTILSALALVYRVAVVCGPQLRL 300

HKYGPSGSVQKFDGLCVLPLNIVNEKIYVFLWFWFIILTILSALALVYRVAVVCGPQLRL

Sbjct 241 HKYGPSGSVQKFDGLCVLPLNIVNEKIYVFLWFWFIILTILSALALVYRVAVVCGPQLRL 300

Query 301 YLLRARSRLSPQGQIETIAKNCHLGDWFVLYQLGKNIDPLVFKELIADLAKKFEGKESV 359

YLLRARSRLSPQGQIETIAK+CHLGDWFVLYQLGKNIDPLVFKELIADLAKKFEGKESV

Sbjct 301 YLLRARSRLSPQGQIETIAKHCHLGDWFVLYQLGKNIDPLVFKELIADLAKKFEGKESV 359

Nucleases genes

Exoribonuclease 1 (Eri1)

>TRINITY_DN6568_c0_g1_i4 len=4573

ATGGGTTTTTAGTAGGAAATGATTTCATCCCAAATCTTCCTAAATTTCACATCGCTAACAATGCTCTTCATA
AATTGTATCAAGCATATATGGAGATTCTTCCTACTTTAGGAGGATACATTAATGATTATGGAACTTTAAATT
TAGAAAGGTTTGAAAAATACTTAACGAAACTCGCTGAAATTGAATTAGAAAATTTTAGGGATATACAAGCC
GACATTAAGTATTTAGAAAGTAAAACGGGAAAGAATCTTCTTTGTGATGATGAAACTGATGAAGGCATATC
TAAATTTGACGATGATTTAATGCGCCTTGTAGAAGACTCTAATAAAATGTTTGAAGGTGAGGATGATACGG
TATATGATAGCTCTGATGATGAACAACTTATACAGGATGAGTTCAGACATCATAAGGCTCGTTTTTATATGG
AAAAATTGAACTACGAGAATGTTGATAGCGAGGTTTTGAAGAGCCAAGCTGAATGCTACGTGCGAGCAAT
ACAGTGGAATTTGTTCTACTATTACACAGGATGCCCATCCTGGTCTTGGTTTTATCCTCATCACTATGCGCC
ATATGTATCAGATATTAAAGGATTCACAGATTTTGATTTTAAATTTGATTTGTCGGCTCCATTTCAGCCATTC
CAGCAACTTCTTGCGGTCTTACCCTCTGCCAGCAAAAATATTGTGCCCGAACCCTTTCAGAACCTCATGGCT
TCACCCGATTCACCTATAATACAATTTTATCCTCCTGAATTCGAGACCGATTTAAATGATAAGATGAACGAT
TGGGAAGCAGTGGTTTTGATTCCATTCATCGAGGAGAAAAGCCTTCTTGATGCAATGCAACCTTACTATAA
GGACTTAACAGAAGAAGAAAATAGGAGGAATTCTGTACAGCCTATGGAACTTTATGAATATACTTCAGATA
ATTTAGGCCGTTGTGAAGCTCCTGAGTTCTTTCCACCGATTGAAAATAATCATGCTCAAATGACTGCTCTCT
CAAGATCAGATATTGCTGTCGATCCCAGAAAAGTTGTAAAGGGTATACTTCCTGGAGCTCTTTCAGGTTCA
AGATATGGATTTCCACAACTTCGGGGTATTTCTTTTAAGGTGGAATTGAAGATGGCAAAGGTTAAAGTTTTT
GAACATCCTAGTCGAGGAGAAAATATGATTCTGAAAATAGAAAGAATAGAATTCACTAGTGAAGAAGTAC
TTTATTCAATGGCTGAGAAACTGCGCAACAAAGTCATTTACGTCGGTTGGCCCCATATTCAGAGAGGAAGG
GCTACGGCTATTTCATCAGAGCAATGTCAGTTCAAAATTGAACACAATAAACCTCCTGTTAAATATGATGA
TAAAACCCGACATAAGCTGGATGCTGATGAGCTTCAGCAAAGACATTTGTTGAACTTGGGAATAGATTTAG
GAAAAGTGGATATCATAATTTATGTTAAAACTGTTGAAGACATAAGAGAACAATTTGATGAAAGTGATTCT
TCCAAAACTCAGGGAAAAAGGACTTACATGTGGAGCGAAACTTGTTATCCATTTCCTTATCAGACTTATCTA
GACCTTAATAATACTGATTTACTTGATAATGATGAGGAATTGGAACAAATCAAGGATTTGAACGAAATTTT
CCCAATTGGAAAACACGTCTTTCTACTGGACTCATTGAACTATGGATTGTTTGGAAAAGTAAAATCTATTGA
AAATGCTAAAATGGTGGAAGTCACTGTTAATGTGGAATCCAATTTGGATCTTTCTGGAATTTTTGAACTATC
GCACAAAACAGAACTTAGGTATTATCCAGGTAGCATAGCCGCTCAGAGGCTTGGTATTGGTAGCCATCTTT
TCTCCCGTTTGACCGGATCTATATTTATCAAGAATGCAGATCGTGATGAAAGAATCAATATTGGTCTTGGAC
TAAAATTTACCAAACAAGGAGAAGAGCTCATTGGCTATACAAGGAGAGAAGGCAACGTTTGGTATTATTCC
GAAAAAACGATTGCACTTCTTGATGAATATATGGCTAAATTTCCTGAATTGTTTGAAAAACTTCAAAACATG
AAAAGTGACGGCGACATGTTCCTCAATAAGGACGTTTTTCCAGATAACACGGATGAAAAAGTGGCTGAAGT
GAGGGCTTGGCTCAAAGCACAGGCTCACACGAGTGCTGAAAGGAGGCCATCAGGCTCGCAACTTTTGACTC
CCGAAGCTATGAAAGAAATTGAAAAACGTGTCACTGAATATTGTGCTAATAAATTAAACAAAACTATTGAC
ATTATGATCAAACCTTCAAGTATATATATCCCAAAGCCAAGACTTAGCAAGTTACCTCCTGATCCAAATACC
ACTGTCAGCATGCTTGATCGTATTGTTTCTGTACGAGAAAACCATTGTATACCACTGGGTTTAATGGGGACT
GTTGTTGGCGTTGACAGGCCTGGCAACAGCGCCAATGCACTGTATGATATCTTATTTGACAGCCCTGTGATT
GGTGGATCATGCCGTTCCTGTGACAAGCCTCGAATTTATAGGCTTCCAAGTCAATCATTTATTAATATCTCC
TATGGAGCAAGAAAGTCAGGAAAAATAAATGTTTCTCTTGCTCCTAAGATGGCTAATGTGAATCAGACCAA
TGTTGGAGAATCTTGGAGGGGATCAAATTACAATAATAGTCCTCGAAATGAGAACAAGTTCTATCAGATTC
CAAGAAACAGAGAACTTAATCAGCCTCTTACTCCTCAAGATGAAAACAGGTTCTATCAGATTCCAAGGAAC
AGAGAACTTAATCAACCTACTACTCCTCAAGGTAATGACTCAGTGAAGCAGTTTCTTCAAAATTTTCATCCT
GTTAAACAGGCAGCATTTCCTGCCAACCATGTACCTTCTGGTAATAGGCCACTAGGCGCAGATTCGGAATT
CCAAGCTATGTGGACTATCCTTCAGAATCAGAAAGTATCTGATGAAAAGTCTTTGCGACCTTGTGTTCCTAC
TCCAAAGGCCACTCCTGTAGCACAAGTAGAATCAGCTGTAGTTGAGCAAACTATGGCACTTCGCAAGTTAC
TCAAGTTGAACGAAACTTCGACACTGCCAGTTAATAAACATTTTACCCCCGATTTTCAGCCTCAATTGATAC
AGCACGCCAGTAACAGCAATCGTAGAGCAGCCAATATGACTCCTCTCATGAATACCAATAATTTTATTTTC
AATCATCAAGGGAGTGTGATGAGGCCAAAGACTTGCAGCCATATTAATTGTGACTTGGAGTTAAGAAATAT
ATGCCTCAATAAAGGTATTCAAATCCCTAAATATAATTATAACCACTTGGCATCTTCTGGAGAGATAACTTG
CATCATAACATTTTCAAATGATATTGGAAAAGTTGTTGGAGATTCTTGTAGGAGTCAAGAAGAGGCTGCTG
AAAACGCTTCTTGTAAGGCTATCAAATTAATATTGAAGAGGGAAGAAAATGTAAGAAGTCCATTAGGTTAT
CGAAGCGTTCATCGACTTCCTGAGTCGCAGCCTATGATGATGCAGCCGCAGCAGTCCACTTCAAGGACAGG
AATGAACCAGCAACTCCCTACTCCTCCTGTTCAATGGTGTTCTAGCAGAACGGGTGTCATTCCAGAAATGA
CGTTCACGCCAACTATGAGGAAACTCTCTGGTAGACCATCTCAGGAACAGTTAAGATCGCCTCAAAAAGTA
CTGAGCCCAGTCATTCCCAACTCTCCGCTGGAAGAATCACGAGGTTACGGACCAAACGCTGGTAAGAATGA
AGAGAGCCATCTGCTAGGAGGCGATGAAGGGAAACAAAAACCTAAGCCTAACCAAGCTCGAAGACCTGCA
GCAAAACCAAGAAGTAGAATCGCTGCTAATTTTTCAGTTCCATTACCTGATAGCAAATAGTTTTAGCTGTTT

GTGTATAATACTTAAATTAATTAATATAGATTTATATAAATATTATATATATTTATAATTGTGTACATTTTGA
AAGAAACAGAAAATATCTGTTTTCTATTAAATATTTGTACATATATTGCGTACCTCATATCATTTACCCTGTT
TTAGGAAAGTTATCTATGTAATAACGAAAGTGATTAATTTAACTCTTCAGCTTTTAACAAAATTGTCTAAAT
CTCTATTCAGCTCATTAATTGTTGACAATTGTGACTAGATAGATAGGTTAATTTGTAAATTAAGAAATTACT
CCTTGTGATAAACTTTATGTTTTAATTTTAAATAACCTTGATATTGTAAAAAAAACTTTTGAATATTTTATGT
ATTTTGAACGATGGTTCTTTCATACGTTTGTATTTTAAATATTTTATGTTATAACTGAATTATGATGACTTGG
TTGGCAAAATTGAAAGTACAATAAGATAATGCTTATGACCTATTTTTTCTTACAATGGAGACATTTTTTATG
GTTATGAAAACAATTTTATGATATTATTCTTTTGTTATTGTTGTTTTCTTCTGTGTAAACATTTTATCTTAATA
GAAACTAAAAGAGAGATACGTAAAAAAGAAACATTTTAAAATCTAATTCATTAAAAAATATGCC

Protein

RF: +1

ORF: 1 -> 3915

Length: 1305 aa

>lcl|ORF1_TRINITY_DN6568_c0_g1_i4:0:3914 unnamed protein product, partial

MGFLVGNDFIPNLPKFHIANNALHKLYQAYMEILPTLGGYINDYGTLNLERFEKYLTKLAEIELENFRDIQADIKY
LESKTGKNLLCDDETDEGISKFDDDLMRLVEDSNKMFEGEDDTVYDSSDDEQLIQDEFRHHKARFYMEKLNYE
NVDSEVLKSQAECYVRAIQWNLFYYYTGCPSWSWFYPHHYAPYVSDIKGFTDFDFKFDLSAPFQPFQQLLAVLP
SASKNIVPEPFQNLMASPDSPIIQFYPPEFETDLNDKMNDWEAVVLIPFIEEKSLLDAMQPYYKDLTEEENRRNSV
QPMELYEYTSDNLGRCEAPEFFPPIENNHAQMTALSRSDIAVDPRKVVKGILPGALSGSRYGFPQLRGISFKVELK
MAKVKVFEHPSRGENMILKIERIEFTSEEVLYSMAEKLRNKVIYVGWPHIQRGRATAISSEQCQFKIEHNKPPVK
YDDKTRHKLDADELQQRHLLNLGIDLGKVDIIIYVKTVEDIREQFDESDSSKTQGKRTYMWSETCYPFPYQTYL
DLNNTDLLDNDEELEQIKDLNEIFPIGKHVFLLDSLNYGLFGKVKSIENAKMVEVTVNVESNLDLSGIFELSHKTE
LRYYPGSIAAQRLGIGSHLFSRLTGSIFIKNADRDERINIGLGLKFTKQGEELIGYTRREGNVWYYSEKTIALLDEY
MAKFPELFEKLQNMKSDGDMFLNKDVFPDNTDEKVAEVRAWLKAQAHTSAERRPSGSQLLTPEAMKEIEKRV
TEYCANKLNKTIDIMIKPSSIYIPKPRLSKLPPDPNTTVSMLDRIVSVRENHCIPLGLMGTVVGVDRPGNSANALY
DILFDSPVIGGSCRSCDKPRIYRLPSQSFINISYGARKSGKINVSLAPKMANVNQTNVGESWRGSNYNNSPRNENK
FYQIPRNRELNQPLTPQDENRFYQIPRNRELNQPTTPQGNDSVKQFLQNFHPVKQAAFPANHVPSGNRPLGADSE
FQAMWTILQNQKVSDEKSLRPCVPTPKATPVAQVESAVVEQTMALRKLLKLNETSTLPVNKHFTPDFQPQLIQH
ASNSNRRAANMTPLMNTNNFIFNHQGSVMRPKTCSHINCDLELRNICLNKGIQIPKYNYNHLASSGEITCIITFSN
DIGKVVGDSCRSQEEAAENASCKAIKLILKREENVRSPLGYRSVHRLPESQPMMMQPQQSTSRTGMNQQLPTPP
VQWCSSRTGVIPEMTFTPTMRKLSGRPSQEQLRSPQKVLSPVIPNSPLEESRGYGPNAGKNEESHLLGGDEGKQK
PKPNQARRPAAKPRSRIAANFSVPLPDSK

Conserved Domains

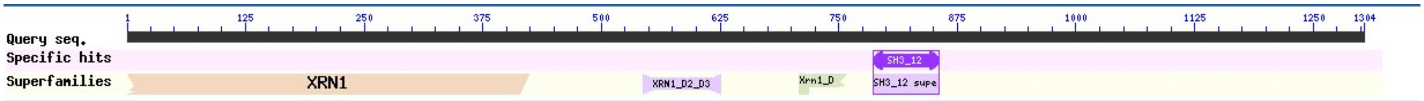

BLASTp

XP_014290344.1 PREDICTED: 5'-3' exoribonuclease 1 [*Halyomorpha halys*]

Score:2280 bits

E-value: 0.0

Query 283 MGFLVGNDFIPNLPKFHIANNALHKLYQAYMEILPTLGGYINDYGTLNLERFEKYLTKLA 342

MGFLVGNDFIPNLPKFHIANNALHKLYQAYMEILPTLGGYINDYGTLNLERFEKYLTKLA

Sbjct 1 MGFLVGNDFIPNLPKFHIANNALHKLYQAYMEILPTLGGYINDYGTLNLERFEKYLTKLA 60

Query 343 EIELDNFRDIQADIKYLESKSGKKLLSD-ETDEAISKFDDELMRLVEDSNKMFEGEDDMA 401

EIEL+NFRDIQADIKYLESK+GK LL D ETDE ISKFDD+LMRLVEDSNKMFEGEDD

Sbjct 61 EIELENFRDIQADIKYLESKTGKNLLCDDETDEGISKFDDDLMRLVEDSNKMFEGEDDTV 120

Query 402 FDSSDDEQLIQDEFRHHKARFYMEKLNYENVDSEVLMSQAECYVRAIQWNLFYYYTGCPS 461

+DSSDDEQLIQDEFRHHKARFYMEKLNYENVDSEVL SQAECYVRAIQWNLFYYYTGCPS

Sbjct 121 YDSSDDEQLIQDEFRHHKARFYMEKLNYENVDSEVLKSQAECYVRAIQWNLFYYYTGCPS 180

Query 462 WSWFYPHHYAPYVSDIKGFTDFDFKFDLSAPFQPFQQLLAVLPSASKNIVPGPFQDLMAS 521

WSWFYPHHYAPYVSDIKGFTDFDFKFDLSAPFQPFQQLLAVLPSASKNIVP PFQ+LMAS

Sbjct 181 WSWFYPHHYAPYVSDIKGFTDFDFKFDLSAPFQPFQQLLAVLPSASKNIVPEPFQNLMAS 240

Query 522 PDSPIIQFYPPDFETDLNDKMNDWEAVVLIPFIEEKSLLDAMQPYYKDLTEDENRRNSVQ 581

PDSPIIQFYPP+FETDLNDKMNDWEAVVLIPFIEEKSLLDAMQPYYKDLTE+ENRRNSVQ

Sbjct 241 PDSPIIQFYPPEFETDLNDKMNDWEAVVLIPFIEEKSLLDAMQPYYKDLTEEENRRNSVQ 300

Query 582 PMEVYEYTSDNLGRCEAPEHFPPIENNHAQVTTLSRSDIAIDPRKVVKGILPGALSGARY 641

PME+YEYTSDNLGRCEAPE FPPIENNHAQ+T LSRSDIA+DPRKVVKGILPGALSG+RY

Sbjct 301 PMELYEYTSDNLGRCEAPEFFPPIENNHAQMTALSRSDIAVDPRKVVKGILPGALSGSRY 360

Query 642 GFPQLRGISFKVELKLAKVKVFEHPSRGENMILKIDRIEFESEEILYSMAEKLRNKIIYV 701

GFPQLRGISFKVELK+AKVKVFEHPSRGENMILKI+RIEF SEE+LYSMAEKLRNK+IYV

Sbjct 361 GFPQLRGISFKVELKMAKVKVFEHPSRGENMILKIERIEFTSEEVLYSMAEKLRNKVIYV 420

Query 702 GWPHIQKGKATAISSEQCQFRIEHNKPPFKYDDKNRHKLDAADLQQRYLLNLGIDLGKVD 761

```
          GWPHIQ+G+ATAISSEQCQF+IEHNKPP KYDDK RHKLDA +LQQR+LLNLGIDLGKVD

Sbjct  421   GWPHIQRGRATAISSEQCQFKIEHNKPPVKYDDKTRHKLDADELQQRHLLNLGIDLGKVD  480


Query  762   IIIYVKTVVDIRVQFDENDSSQTQGKRTYMWSETHYPFPYQTYLDLNNFTLIDNDEELEQ  821

          IIIYVKTV DIR QFDE+DSS+TQGKRTYMWSET YPFPYQTYLDLNN  L+DNDEELEQ

Sbjct  481   IIIYVKTVEDIREQFDESDSSKTQGKRTYMWSETCYPFPYQTYLDLNNTDLLDNDEELEQ  540


Query  822   MKDLNEIFPIGKQVFLMDLLNYGMFGKVKSIDDNKRVEVIINVEPNLDLSGILELSHKTD  881

          +KDLNEIFPIGK VFL+D LNYG+FGKVKSI++ K VEV +NVE NLDLSGI ELSHKT+

Sbjct  541   IKDLNEIFPIGKHVFLLDSLNYGLFGKVKSIENAKMVEVTVNVESNLDLSGIFELSHKTE  600


Query  882   LRYYPGSIAAQRLGIGSHLFSRLTGSIFIKNADRDERINVGLGLKFTKQGEELIGYTRRE  941

          LRYYPGSIAAQRLGIGSHLFSRLTGSIFIKNADRDERIN+GLGLKFTKQGEELIGYTRRE

Sbjct  601   LRYYPGSIAAQRLGIGSHLFSRLTGSIFIKNADRDERINIGLGLKFTKQGEELIGYTRRE  660


Query  942   GNIWYYSEKTIALLDEYMDKFPELFEKLHSTKSDGDMYLNKDIFPDNTDEKMSEIRTWLK  1001

          GN+WYYSEKTIALLDEYM KFPELFEKL + KSDGDM+LNKD+FPDNTDEK++E+R WLK

Sbjct  661   GNVWYYSEKTIALLDEYMAKFPELFEKLQNMKSDGDMFLNKDVFPDNTDEKVAEVRAWLK  720


Query  1002  AQSHSSAERRPSGSLLLTSEAMKEIEKRVIELCANRANKT--LAVNPSSIYIPKPRLSKL  1059

          AQ+H+SAERRPSGS LLT EAMKEIEKRV E CAN+ NKT  + + PSSIYIPKPRLSKL

Sbjct  721   AQAHTSAERRPSGSQLLTPEAMKEIEKRVTEYCANKLNKTIDIMIKPSSIYIPKPRLSKL  780


Query  1060  PPDPNTTVNMLDRVIFVRENHCIPLGLMGTVVGVDKPANSGNALYDILFDCPVIGGSCRS  1119

          PPDPNTTV+MLDR++ VRENHCIPLGLMGTVVGVD+P NS NALYDILFD PVIGGSCRS

Sbjct  781   PPDPNTTVSMLDRIVSVRENHCIPLGLMGTVVGVDRPGNSANALYDILFDSPVIGGSCRS  840


Query  1120  CDQPRIYRLPNQSFINISYGARKSGKVNVSAPSKMANVNQTNVGESWRGSNYNNSPRNEN  1179

          CD+PRIYRLP+QSFINISYGARKSGK+NVS  KMANVNQTNVGESWRGSNYNNSPRNEN

Sbjct  841   CDKPRIYRLPSQSFINISYGARKSGKINVSLAPKMANVNQTNVGESWRGSNYNNSPRNEN  900


Query  1180  K--------------------FYQIPRNRELSQPGTPQGNDSVKQFLQNFHPAKQGAFP  1218
```

K FYQIPRNREL+QP TPQGNDSVKQFLQNFHP KQ AFP

Sbjct 901 KFYQIPRNRELNQPLTPQDENRFYQIPRNRELNQPTTPQGNDSVKQFLQNFHPVKQAAFP 960

Query 1219 ANHVPHPNRSLGADSEFQAMWNFLQNQKVPEEKSLRPGVPPQQKVPVSPVE--VVEQTIA 1276

ANHVP NR LGADSEFQAMW LQNQKV +EKSLRP VP + PV+ VE VVEQT+A

Sbjct 961 ANHVPSGNRPLGADSEFQAMWTILQNQKVSDEKSLRPCVPTPKATPVAQVESAVVEQTMA 1020

Query 1277 LRKFLKLNETPLPPVNQHFTPEFQPQLIQHANNSSRRAGNMAPL-NTNNFLFN-QGSVMR 1334

LRK LKLNET PVN+HFTP+FQPQLIQHA+NS+RRA NM PL NTNNF+FN QGSVMR

Sbjct 1021 LRKLLKLNETSTLPVNKHFTPDFQPQLIQHASNSNRRAANMTPLMNTNNFIFNHQGSVMR 1080

Query 1335 PKTCSHINCDLELRNLCVNKNIQIPRYNYKHLASSGEITCIITFSNDIGKAVGDSCRNQE 1394

PKTCSHINCDLELRN+C+NK IQIP+YNY HLASSGEITCIITFSNDIGK VGDSCR+QE

Sbjct 1081 PKTCSHINCDLELRNICLNKGIQIPKYNYNHLASSGEITCIITFSNDIGKVVGDSCRSQE 1140

Query 1395 EAAENASCKAIKLILKREEIVRSPLGYRNVHR--PAQPMAMQPQQSTSRIGMNQQLPIPP 1452

EAAENASCKAIKLILKREE VRSPLGYR+VHR +QPM MQPQQSTSR GMNQQLP PP

Sbjct 1141 EAAENASCKAIKLILKREENVRSPLGYRSVHRLPESQPMMMQPQQSTSRTGMNQQLPTPP 1200

Query 1453 VQWCSSRTNMIPDTQFTPPMRN-----KQDQIRSPQKVLSPVVPNSPQEESRGYGQKTVK 1507

VQWCSSRT +IP+ FTP MR Q+Q+RSPQKVLSPV+PNSP EESRGYG K

Sbjct 1201 VQWCSSRTGVIPEMTFTPTMRKLSGRPSQEQLRSPQKVLSPVIPNSPLEESRGYGPNAGK 1260

Query 1508 NEETHPLSAEEGNQKPKPNQSHRRSAAKAKSRIAANFSVPLPDSK 1552

NEE+H L +EG QKPKPNQ+ RR AAK +SRIAANFSVPLPDSK

Sbjct 1261 NEESHLLGGDEGKQKPKPNQA-RRPAAKPRSRIAANFSVPLPDSK 1304

Small RNA degrading nuclease 1 (SDN1-like)

>TRINITY_DN36580_c0_g1_i1 len=2254 path=[1:0-812 2:813-2253]

ATTTTGAGAGTCTTGGTTTTATACTGTACAAACATAAATTAGGTTTTTGATTGCGTTTTTGGAAGAATAATTT
TATTTTGGGCAAGAATGCCAAAAAAAAATGACATAGCAAAAGTTTTCATTTTACACTTTTGACGAAGTTAATT
TCACATGAAAACAAAATTTTTAAGTGATTTCTGAGTTAGTGAATTAACTCGTTGGCAGAACTTTAACAACAT
AGTTAGCGGGAGGTAATGTTTTGGTGTTGGGGGTTATCTATCCACGTGATAGATGAATTTTAACACCTCTAA
TTTATATATGTATTCGACGGATAATTACATCTTTGAACAGTTTTAATGATGCTAGCTATTTTCCATCAAGAA
AATAGCGCTTTATTGCTAACGCTAGTATCTGTTTTCAGTTAAAGTTTTTATAACCCAAAAAAAAAAATTTAAA
AAGATCTAAATTAATGGTTTTCCAGTATGTTTGAGCATAACAAGCGTAATCTAAAAAAACTTAGAAGAAAG
GAAAATAAAAAGAAGAAATTAAAAGCATTGTTGGAAATAAGTAGTTTGAATGATCAAGATCGTAAGAAGT
CAAGTGTGAATTCAGCTTCTGTTGGACCACCTCAGAGAGATCCATCTAATAAAAAATCCCAAAAAAACTTT
GAGGAACCAACTCCAAAACGAATGAAGCGAGATTTTCTCTTTCCACTCAATGGTGCTGCAAGGAGTGACGA
AAATGAAGATCAGGCTAAAAAGAGTAGAAGTCTTCTCACTGAGGAACAACTTGCTGAATTGAACAAGCTA
CTTAAAATGAGAAAAAAACTAAGACATGAAAGACCTTTTCTGGAGCTGAATGAAGTTGGTGAAGCCGCAT
CTCTTAGTTTGCCAGAGTCTCAAAGAGTGCCGTTGCCGTTTGATGATTTCCAACACTTCATTTTAACCTCACT
TTTTGCTCATATGAGCTCGAAAGGTCCCAGTAGGTGGATAAATTGTAAAAAGCACCAAAAATTAGAGCAGA
GGATATTTATGATTATCGAGGGTGCTGGTCTAAAAGATTATTTAGGAAAGGAAGCAGACTTGAAAACACTT
TCACAATTTGAGAAGCAGTTGGAAGTCCTATCACCGTTACAGTACGGCGGAGATTTGATTAGTGAACTGGC
GCTTGTTTCGTTAACTAACACCCAAAGGCAAAAGCTTGCTTCTGAATATGGATCGTTAAAAGAAGCTTACA
GTTCTGGTGACATATTTAATACTTTCAGAACTATTTTCCCAATAACTGCCAAATGTGAAAAAGACGCTGAAG
ATAAAACTGATCAGAAAGAAGATATACCAGCGATTAAACCGGAACCGAAGATGTTCGAAGAAAGGCCAAA
GGATAAGTTCTCGAGGAAGCAGCTACTTCTGAGCCCTTGGGACATGATAGAAGAAAATTATCCGCTTCCTT
TATCGGGAGAACTCGGGCGGAGATACTCTGACTTTGTTTTCACGCGTGATAGTTACACTGAAGTGACCGAG
AATTCACCCATGTGGTCTCTTGATTGTGAGATGTGTCTTACAACTGATGGTAGTGAATTAACTCGCATCTGC
ATTGTTGACGAGGATTGCAACATCGTCTATGAAACTTTAGTGAAGCCGTATAACCCTATTAAAAACTATCTT
ACTAAATTCTCTGGTATCACTCCTTCGATGATGAAGCCAGTGCAGACTCGATTAGAAGATGTGCAAGCAGA
TATTCGTGCCATCTGCCCCCCTGATCTTATTCTTATAGGTCAGTCATTGAACATGGATTTGATGGCGATGAA
GATGATGCACCCTTACGTCATAGACACATCAGTTATTTTCAACATGTCCGGGATCAGGCAGAGAAAGACAA
AGCTGAGGATTCTCGTCGAGGAGTTCTTGTCGTTGAGGATTCAAACCAACGACAGCCTTGGCCACAGCCCG
ATTGAGGATGCTCTTGCTGCTATGAAACTAGTCCAAGCCAAACTGTATCACTCTATCAGTTGGGGAAACGC
AGTACTAGGAGGCCAGAGATCTAAAGAAGAATCTAATTCTGCAAGAATCCTCCTGGAGTCGAGGGAGGCC
GCTGGGATGGCACGGTTATCCGTCGCCGAATCTAAACGACTCTCATGGCCACAATTGAAAAGAGAGAGGG
ATCGGAACGTTGATAACTTGCATAAGTCCCGAAAGGGTTTTCGTCCAGACACGCAGCAGTTTGAGAATATT
AGTGCCACTTCACTTTTCTCCCACTTGGTCCGATGTGATCGAACTG

Protein

RF: +1

ORF: 460 -> 2253

Length: 597 aa

>lcl|ORF1_TRINITY_DN36580_c0_g1_i1:459:2252 unnamed protein product, partial

MFEHNKRNLKKLRRKENKKKKLKALLEISSLNDQDRKKSSVNSASVGPPQRDPSNKKSQKNFEEPTPKRMKRD
FLFPLNGAARSDENEDQAKKSRSLLTEEQLAELNKLLKMRKKLRHERPFLELNEVGEAASLSLPESQRVPLPFDD
FQHFILTSLFAHMSSKGPSRWINCKKHQKLEQRIFMIIEGAGLKDYLGKEADLKTLSQFEKQLEVLSPLQYGGDLI
SELALVSLTNTQRQKLASEYGSLKEAYSSGDIFNTFRTIFPITAKCEKDAEDKTDQKEDIPAIKPEPKMFEERPKD
KFSRKQLLLSPWDMIEENYPLPLSGELGRRYSDFVFTRDSYTEVTENSPMWSLDCEMCLTTDGSELTRICIVDED
CNIVYETLVKPYNPIKNYLTKFSGITPSMMKPVQTRLEDVQADIRAICPPDLILIGQSLNMDLMAMKMMHPYVID
TSVIFNMSGIRQRKTKLRILVEEFLSLRIQTNDSLGHSPIEDALAAMKLVQAKLYHSISWGNAVLGGQRSKEESNS
ARILLESREAAGMARLSVAESKRLSWPQLKRERDRNVDNLHKSRKGFRPDTQQFENISATSLFSHLVRCDRT

Conserved Domains

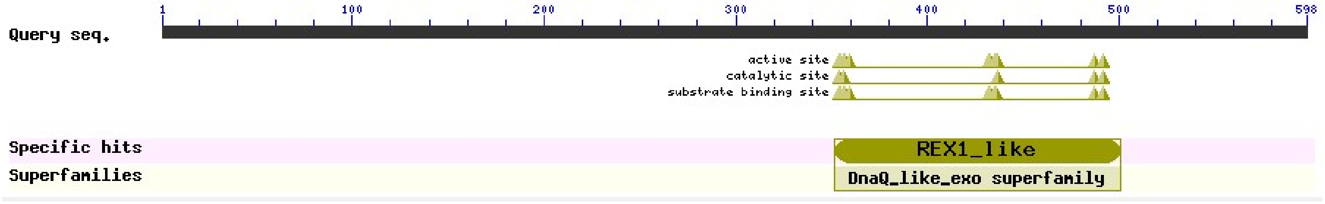

BLASTp

XP_014279339.1 uncharacterized exonuclease C637.09 isoform X1 [*Halyomorpha halys*]

Score:871 bits

E-value: 0.0

Query 7 MLEHNKRNLKKLQRKENKKKKLKALLEISSLNDQDRKKSSINSASSGLPVKDPSSKKFQK 66

M EHNKRNLKKL+RKENKKKKLKALLEISSLNDQDRKKSS+NSAS G P +DPS+KK QK

Sbjct 1 MFEHNKRNLKKLRRKENKKKKLKALLEISSLNDQDRKKSSVNSASVGPPQRDPSNKKSQK 60

Query 67 NFEEPTPKRMKRDFLFPLNGIAGNDDENVQIRQDRSLLTEEQISELNKLLKMRKRIRHER 126

NFEEPTPKRMKRDFLFPLNG A +D+ Q ++ RSLLTEEQ++ELNKLLKMRK++RHER

Sbjct 61 NFEEPTPKRMKRDFLFPLNGAARSDENEDQAKKSRSLLTEEQLAELNKLLKMRKKLRHER 120

Query 127 PFLELNEVGEAASLSLPGSQRVPLPFDDFQHFILTSLFAHMSTKGPSRWINCKKYQKLER 186

PFLELNEVGEAASLSLP SQRVPLPFDDFQHFILTSLFAHMS+KGPSRWINCKK+QKLE+

Sbjct 121 PFLELNEVGEAASLSLPESQRVPLPFDDFQHFILTSLFAHMSSKGPSRWINCKKHQKLEQ 180

Query 187 RIFMIIEGAGLKEYLEKEAELKTLSQFEKKLEVLTPLQYGGDLVRELALVSLTNSQKQKL 246

RIFMIIEGAGLK+YL KEA+LKTLSQFEK+LEVL+PLQYGGDL+ ELALVSLTN+Q+QKL

Sbjct 181 RIFMIIEGAGLKDYLGKEADLKTLSQFEKQLEVLSPLQYGGDLISELALVSLTNTQRQKL 240

Query 247 VSEYGSLKEAYKSGDVFKTFKTIFPITAKCNKAAE--SIRKEDIQANNTESTMY-KRQKD 303

SEYGSLKEAY SGD+F TF+TIFPITAKC K AE + +KEDI A E M+ +R KD

Sbjct 241 ASEYGSLKEAYSSGDIFNTFRTIFPITAKCEKDAEDKTDQKEDIPAIKPEPKMFEERPKD 300

Query 304 KFSRKLLLLSPWEMAEEDFPLLLDEERQQRFADFVFTRDSYSEVSDNSPMWAVDCEMCST 363

KFSRK LLLSPW+M EE++PL L E +R++DFVFTRDSY+EV++NSPMW++DCEMC T

Sbjct 301 KFSRKQLLLSPWDMIEENYPLPLSGELGRRYSDFVFTRDSYTEVTENSPMWSLDCEMCLT 360

Query 364 AYGHELTRICIVDENSNIVYETLVKPYNPIINYLTQHSGITPSMLKNVNTRLEDVQADIR 423

G ELTRICIVDE+ NIVYETLVKPYNPI NYLT+ SGITPSM+K V TRLEDVQADIR

Sbjct 361 TDGSELTRICIVDEDCNIVYETLVKPYNPIKNYLTKFSGITPSMMKPVQTRLEDVQADIR 420

Query 424 AICPPDVILIGQSLSNDLKAMQMMHPYVIDTSVIFNLSGVKYRKTKLKVLVEEFLSLRIQ 483

AICPPD+ILIGQSL+ DL AM+MMHPYVIDTSVIFN+SG++ RKTKL++LVEEFLSLRIQ

Sbjct 421 AICPPDLILIGQSLNMDLMAMKMMHPYVIDTSVIFNMSGIRQRKTKLRILVEEFLSLRIQ 480

Query 484 TEDKLGHCPIEDAVAAMKLVQEKLRHTPTWGNAVLAGPRSKEESRSARVLLESREAAGMG 543

T D LGH PIEDA+AAMKLVQ KL H+ +WGNAVL G RSKEES SAR+LLESREAAGM

Sbjct 481 TNDSLGHSPIEDALAAMKLVQAKLYHSISWGNAVLGGQRSKEESNSARILLESREAAGMA 540

Query 544 RLSLTNSERITWTQLMNKKRDDQNDDGVY----NFHPYSDNQYED-NSTSLFAHLV 594

RLS+ S+R++W QL K+ D+N D ++   F P + Q+E+ ++TSLF+HLV

Sbjct 541 RLSVAESKRLSWPQL--KRERDRNVDNLHKSRKGFRPDT-QQFENISATSLFSHLV 593

DNA/RNA non-specific endonuclease isoform 1

>TRINITY_DN4766_c0_g1_i2 len=1504 path=[1:0-162 5:163-1503]

TGTGGACTAGCTCTATTGACACAACTGTTCGCGCTCGACTATAAAACCCGCGTGCAAGGTTCTGACAATGTGACAGTTCT
TCAGGATGGTCGCGTTTCTTCTTGCATCTCTAGCGGCGTGTCTTCTGGCGGCCGAAGCCAGGGTCGTCTCCAGGGTAGAC
CCTGCAGGAGGCGCATGTATTCTGGACTTGAATAATGACCTGCCGAAGAAAAATGAACCTCTCTTCCTCCAGAAGACAG
CTTCCGGTTCTCTCGACCTCGTCCTTCCGGAAATGGAAGGAACAAGAGGAGTCATCGCCCTGAGGGAAGGAGAGCAAAT
CGTGGTCTCTTGTCCCGGTAAGAAGAACCACATCGCAGCGACTAATACCGAGTTCTCAGGCGCATCTTGCCGCAGCGGT
ACCAAGCTGGCCATCGACGGCTCGGTCTTCAACTCATTGGACCTCGACTGCAGCTCCAGGGCAGGATCCACCACCAGGC
CCACCCAGAAGAAGTGCGCAGGAGGAAAGGGTATCATCGTTGAGCTTGGTTTTGATGTCGGCAACTCCTGGATCCCGAT
GATCGAGTCCTGCCACGACGTAGCCAACAGCAACACCTTCTACTCCACCCACACCATCCACGGAGCCATCATGGGAGGC
AAGGTCTACAGGACGACCGCCAGGCCAGTCTTCGCCAGGGGAGATAGCATCTTCTTCAAGGGATTCAACCCGGAACAG
GCTTACACTCAGAAAAACCAACAAACCGTCCTCTCCCGCGAACTGGGAGCCGCCAGCGCCGGCAAACACTTCGACGCC
AAGAAGACCTTCTTTCTGGCCAGAGGTCACCTCGCCCCCGACGCGGACTTCCTCTTCAGCGCCCACCAGTTCTTGACCTA
CTTCTATGTCAACGTGGCTCCGCAATGGCAGTCCATCAACGCCGGCCACTGGCTCAAGGTTGAAGACAACACCAGGAGG
ATCGCCAAGAGTCTCGGCGCTGACCTCCAAGTGGTGACAGGAACGGAAGGCGTCCTCAGCCTTCCAGCAGCCAGAGGC
GAGAAGCAGATCAGACTCCAGGACTCCAGGCTTCCGGTGCCCGAGCACTTCTGGAAGGTCGTCAGGAACACCCAGGAC
GACTCCTGCATCGCCTTCGTCTCCACCAACAACCCCTTCATCACCAAGCCACCCAAGGCCATCTGCCCAGACGTCTGCTC
CCAGAACGGATGGCCGCTCCTCCAGAATGACCTCTCCAAGGGCTACGTCTACTGCTGCAGGTACCAGGACATCAAGAAG
GCCATCCCTGACATGCCCAACCTCAACTGCAAGTCCGTCCTCAGGTGGAAATGAGCTGTATGACTTGTCTAATAGACGA
TTTGTAAATAAATACAATGCCAAACTCTATCTAGATCAGTCTACACAAACCTATTAATCATCAGTATAAAATAAGTTTAA
TTAAATTTAGAGTAATATGAAATTATAATTAAGAGGCGGAGTAGAAGAAATGGAGGAGAGTGGTTCCTCCTGAAATAA
AAA

Protein

RF: +2

ORF: 86- 1318

Length: 410 aa

>lcl|ORF1_TRINITY_DN4766_c0_g1_i2:85:1317 unnamed protein product

MVAFLLASLAACLLAAEARVVSRVDPAGGACILDLNNDLPKKNEPLFLQKTASGSLDLVLPEMEGTRGVIALRE
GEQIVVSCPGKKNHIAATNTEFSGASCRSGTKLAIDGSVFNSLDLDCSSRAGSTTRPTQKKCAGGKGIIVELGFDV
GNSWIPMIESCHDVANSNTFYSTHTIHGAIMGGKVYRTTARPVFARGDSIFFKGFNPEQAYTQKNQQTVLSRELG
AASAGKHFDAKKTFFLARGHLAPDADFLFSAHQFLTYFYVNVAPQWQSINAGHWLKVEDNTRRIAKSLGADLQ
VVTGTEGVLSLPAARGEKQIRLQDSRLPVPEHFWKVVRNTQDDSCIAFVSTNNPFITKPPKAICPDVCSQNGWPL
LQNDLSKGYVYCCRYQDIKKAIPDMPNLNCKSVLRWK

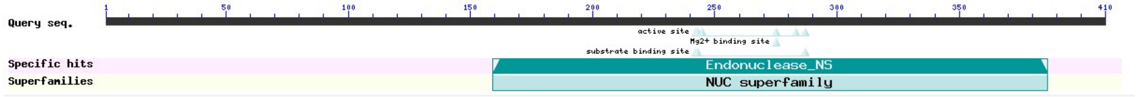

Query 138 GGKGIIVELGFDVGNSWIPMIESCHDVANSNTFYSTHTIHGAIMGGKVYRTTARPVFARG 197

G +G+I +VG+SWIPMIESCHDV NSNTFYSTHT+HGAIMGGKVYRTTARP+F+RG

Sbjct 90 GNRGVIALREGEVGDSWIPMIESCHDVENSNTFYSTHTVHGAIMGGKVYRTTARPLFSRG 149

Query 198 DSIFFKGFNPEQAYTQKNQQTVLSRELGAASAGKHFDAKKTFFLARGHLAPDADFLFSAH 257

SIFFKGFNPE AYTQKNQQ VL+R+LG A+A K+FD+KKTF+LARGHLAPDADFLFSAH

Sbjct 150 LSIFFKGFNPETAYTQKNQQAVLARDLGTANANKYFDSKKTFYLARGHLAPDADFLFSAH 209

Query 258 QFLTYFYVNVAPQWQSINAGHWLKVEDNTRRIAKSLGADLQVVTGTEGVLSLPAARGEKQ 317

QFLTYFYVNVAPQWQSINAG+WL+VEDNTR+IAKSLGADLQ+VTGTEG+L+LP+ +GEK+

Sbjct 210 QFLTYFYVNVAPQWQSINAGNWLRVEDNTRKIAKSLGADLQIVTGTEGILTLPSTKGEKE 269

Query 318 IRLQDSRLPVPEHFWKVVRNTQDDSCIAFVSTNNPFITKPPKAICPDVCSQNGWPLLQND 377

IRLQ S+LPVPEHFWKV+RNTQDDSCIAFVSTNNPF+T PK +C DVCSQNGWP+LQND

Sbjct 270 IRLQSSKLPVPEHFWKVLRNTQDDSCIAFVSTNNPFLTSAPKTLCQDVCSQNGWPVLQND 329

Query 378 LSKGYVYCCRYQDIKKAIPDMPNLNCKSVLRWK 410

LSKGYVYCCRYQDIKKAIP+MPNL CKSVL+ K

Sbjct 330 LSKGYVYCCRYQDIKKAIPEMPNLTCKSVLKGK 362

DNA/RNA non-specific endonuclease isoform 3

>TRINITY_DN14109_c0_g1_i4 len=1531 path=[0:0-292 2:293-887 4:888-1530]

CACCGAAAAGCACTTCTGCATTTAACCAAATTATTAATTGATGAAGAAAAGTTTGATTAGATAAAGTCACT
AAAACCGGCGCTAAGTCTTCAGTAAGGATGGCAGTAGCTAAGGAACAAACATCTTTTTTCCGCAGTTTATC
TGTGGAGCCTGCTGTCTTTCTCTACTCTTTCGCTATATATTGGTACAATATTATGATACCGAATCTCATTCAA
CAGAAAGTTTGCAGTCCAGAACTTCCACCGTCGGTTGGGTTCGTCTGCAAAGACATAGATCTGCTCGCTGT
AGCCACAGACATTGTTACTACTCGATCAACACTAAGGGATGCTCTCCCAGCTATGATTATACTTTTTGCTGG
TTGCTGGCGTGACGTAACATCTCTCAATAAGCCAATAATGTATTTTTCAATAGTTTCGGAAATTATTGGAAT
CTCTATTCAGATATTTGCTTATATAAACTGGACAATTTCACCTTGGGTAGCTATCTTTATTGAAGGGATTATC
AATGGCTTAGGAGGAGCCAGCAAGCTATTTTTTATTGGGGCTACCTGTGCAATCACTGATATTACCAATACC
GAAAACAGGACAAAACGTCTCACACTGATTTCTGCTATGATATCTTTTGGAGCCTGTTTCGCTTCTGCAACA
TCTGGATATGGCCTATCATACATGGGCTACACATGGTATTTGATTTTGGCAATCGTTTTCCAAGTAGCTAGT
TTTATATTGGTTATCATTTTTGTTAAGGATAAAAAACCAAAGGAAGGAAAAGTAGACATTTATGAAATGCT
CAAACAGCTTAAAATAACTGCTTTGAAAAGAAGAACAAACAACATAATAATTTGGTTAATGGTTTTCTCAT
CTTGCTCAATGACTGTTATAGCCATAGCTGAGGGAAACATTTTTGTTTATTATCTTCAGAGAGGTTTTGATTT
CACAATTGAGGAAGTAGGAGTTTATAATTCATATCGACAAATGGTTGGGGCAGTGAGTACTCTACTAGTTC
TTCCCATACTTACTAATATATTAAAATGGCAAGATTTCAAAATTGGTATTGTGGCATGTACAGTGACAATTA
TCTCATGTATTTCAATGGTTTATGCCAAGACTAAGTGGGAATTAATAATATTTGCTTTATTCGAGTTCATGA
GGACTTTACTTTTTGGCCTTCCAAAGTCAGTCATTTCAAAATGTGTAAACGAAAATGAAATTGGTTTGTTTA
TCAGCTTTTGCTTTATCGGAGAATGTATTTTACCAATTGGTATGTTTTATCTTTATGACTATATCTATACTCA
GACCTCTACAGCGCTCCCTGGTGCATTCTATTTTGCAAGTGCTGCTACCATTGCAGTTTCATTGATATTTTAC
AGCTTTTCTTCTTGTATCTACACCCCTCTTGAAGTTTGTGATAAAGTAATTAAAAGTGATCTAGAAAAGTTT
GAAAAGAAGTCTCAGCTGCAAGAAAAGATAAAAAATGATTCTGTGTTCAAAATGATAAATAAATGAAATT
TTTTCAAAGGTCAAAAAAAAAA

Protein

<u>RF:</u> +3

<u>ORF:</u> 99 -> 1505

<u>Length</u>: 468 aa

>lcl|ORF3_TRINITY_DN14109_c0_g1_i4:98:1504 unnamed protein product

MAVAKEQTSFFRSLSVEPAVFLYSFAIYWYNIMIPNLIQQKVCSPELPPSVGFVCKDIDLLAVATDIVTTRSTLRD
ALPAMIILFAGCWRDVTSLNKPIMYFSIVSEIIGISIQIFAYINWTISPWVAIFIEGIINGLGGASKLFFIGATCAITDIT
NTENRTKRLTLISAMISFGACFASATSGYGLSYMGYTWYLILAIVFQVASFILVIIFVKDKKPKEGKVDIYEMLKQ
LKITALKRRTNNIIIWLMVFSSCSMTVIAIAEGNIFVYYLQRGFDFTIEEVGVYNSYRQMVGAVSTLLVLPILTNIL
KWQDFKIGIVACTVTIISCISMVYAKTKWELIIFALFEFMRTLLFGLPKSVISKCVNENEIGLFISFCFIGECILPIGM
FYLYDYIYTQTSTALPGAFYFASAATIAVSLIFYSFSSCIYTPLEVCDKVIKSDLEKFEKKSQLQEKIKNDSVFKMI
NK

Conserved Domains

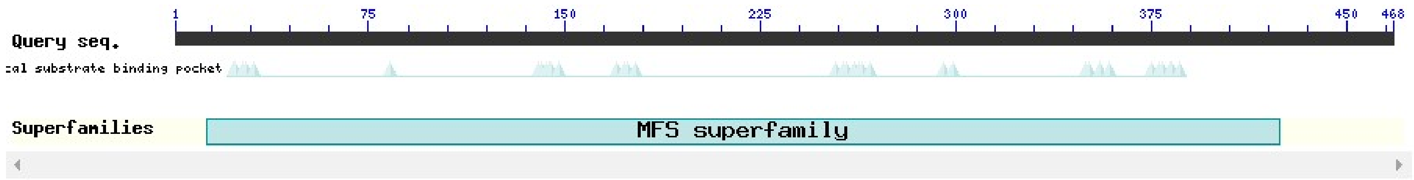

BLASTp

XP_014293261.1 uncharacterized protein LOC106691872 [*Halyomorpha halys*]

Score:687 bits

E-value: 0.0

Query 1 MAVAKEQTSFFRSLSVEPAVFLYSFAIYWYNIMIPNLIQQKVCSPELPPSVGFVCKDIDL 60

M+ AKEQTSF SLS EPA+F Y+FA+ WYN ++PNL+QQKVCSPE PP V F C D L

Sbjct 1 MSAAKEQTSFLNSLSSEPAIFFYAFAVLWYNGILPNLVQQKVCSPEFPPPVEFACNDTKL 60

Query 61 LAVATDIVTTRSTLRDALPAMIILFAGCWRDVTSLNKPIMYFSIVSEIIGISIQIFAYIN 120

LA ATDIVTTR TLRDALPAMI+LF+GCWRD TSLNKPIMYF+I+SEIIGISI+IFAY+N

Sbjct 61 LAEATDIVTTRVTLRDALPAMILLFSGCWRDATSLNKPIMYFAIISEIIGISIEIFAYLN 120

Query 121 WTISPWVAIFIEGIINGLGGASKLFFIGATCAITDITNTENRTKRLTLISAMISFGACFA 180

WTISPW A+F +GIINGLGG SKLF+IGATCAITD T TE+RT RLTLISAMI FG C A

Sbjct 121 WTISPWAAVFADGIINGLGGGSKLFYIGATCAITDNTGTEDRTTRLTLISAMIMFGCCLA 180

Query 181 SATSGYGLSYMGYTWYLILAIVFQVASFILVIIFVKDKKPKEGKVDIYEMLKQLKITALK 240

S TSGYGL YMGYTW+LILAI+FQ+ S +LV+I VKDKK ++GK DI +M KQLKITALK

Sbjct 181 SVTSGYGLKYMGYTWFLILAIIFQIVSLLLVMILVKDKKRRKGKADILKMFKQLKITALK 240

Query 241 RRTNNIIIWLMVFSSCSMTVIAIAEGNIFVYYLQRGFDFTIEEVGVYNSYRQMVGAVSTL 300

RR NNIIIWLMVFSSCS+TV+AIAEGN+ +YYL++GF FTIEE GVY+SYRQM+GAV T+

Sbjct 241 RRPNNIIIWLMVFSSCSLTVVAIAEGNVTLYYLEKGFGFTIEEAGVYSSYRQMIGAVGTI 300

Query 301 LVLPILTNILKWQDFKIGIVACTVTIISCISMVYAKTKWELIIFALFEFMRTLLFGLPKS 360

LV PIL+ +L W+D IGIV+C VTI+ I+MV AKTK ELIIFALF+FMRTLLF LPKS

Sbjct 301 LVPPILSKLLGWKDSIIGIVSCAVTIVMYITMVIAKTKLELIIFALFDFMRTLLFSLPKS 360

Query 361 VISKCVNENEIGLFISFCFIGECILPIGMFYLYDYIYTQTSTALPGAFYFASAATIAVSL 420

VISKCV+ENEIG+F+SFC IGECILPIG+FYLYDYIYT TST LPGAF+ ASA   AVSL

Sbjct 361 VISKCVDENEIGVFVSFCIIGECILPIGIFYLYDYIYTATSTTLPGAFFLASAVIAAVSL 420

Query 421 IFYSFSSCIYTPLEVCDKVIK---SDLEKFEKKSQLQEK 456

IFYS ++CIYTPL V D+ +K   SDLEK EK+SQLQEK

Sbjct 421 IFYSITACIYTPLNVNDEEVKKISSDLEKCEKESQLQEK 459

Exosome

>TRINITY_DN30464_c0_g1_i1 len=3293 path=[2:0-1905 3:1906-2672 4:2673-3292]

GTTATGTTTGTTGTATACTCACGTGGGTAGCATTCGTGTTGAGTGAAGTCCCATCTTTTGTTAAACATGTTAA
CCAAGAAGGTTTTTATAAACAGAGCAAAAAGGGGACAATTTTAAAGATTGTCAGGGAACATTACCTGCGG
AAAGACATTTGGTGTGGTTGCAAAGGTTGTACAAAATGTGCTCTTGAAGAAAAAGATATGATCTTAGATGC
TTGTCCTATTTCTAAATGTTCCTTATTATCAGAGCCTCATTATATTTTTTTGGACACTAATGCTGTCTTAGATC
AGATTGATGCTATTGAGGATCCCTATTTGGAAAATATTATTATTCCTCAGACTGTATTAGATGAAGTTAAAC
ACAGGAGCTCTTCTGTATTCAAAAGGCTCAAAGAAGTCATTTTTGACAAAAAAAGGAAAATATACGTATTT
GTGAATGAACATCTTAAAGCGACGTACATCGAAAGGAAAGTCGGAGAGACAGTGAATGACAGAAATGACC
GAGCGATCAGAAGAGCGGCACTCTGGTTCCAGGAGCATACCGGGGTCAAATCGGTCCTGCTTTCTGATGAT
GTCGCTAATAGGGAGAAAGCTATTGAGAAAGGCGTTGTTGCGTTTTCATTGAGTGATTATGCAGAGGCAAC
TGATTCAGCTACGCTGAGTGAGAGGCTTTGTGCCAAGCGATGGAGCGAAAGAAGAGGAGAAGATTCGACT
GACGTGTTCCCGGCTCACCTTCCTCCAGCCGCCGTTCACCACGGCCTCAAAGCTGGCCTCCTCCTCCAGGGG
GTCTTCTACGCCTCCAAGGAAAACTGTCTGGAGGCCACCGTCATCACCGACAAGCATAATGTATTAATTCA
AGGAAGAGCGAGCTTGAACCGGGCAATAGATGGTGACACAGTCGCCGTTGAGTTGTTACCAAAAGAAGAT
TGGGTAGCTCCGTCGAATCTCGTCATCCAATCAGAAGAGGCTGAGGACGAGGTGAAAGTTGAAAGAGAAA
TAAATGAAGAAGAGAGGCAAGTCACGGGCAAAATTGTTGCCATAATTCACAGAAAGTGGCAGCAATACTG
CGGCATTTTGCAGCTCTCTCCAATCAAAGGGCATACTCATCATTTATTTGTTCCTGCTAAAAGACACATTCC
TAAAGTGAGAATCGAAACGAGGCAAGGGGAAAAGCTCGCCAGCCAAAGACTAGTCGTCGCTATAGATGCC
TGGCCGAGGCACTCCAGGTATCCACAGGGCCATTTTGTTCGAGCGCTGGGGAAGATCGGTGACAAGGAGA
CTGAGAATGAGGTGCTCCTGTTGGAACATGACATCCCGTGCTCCAAGTTCTCTGATGCTGTTCTGGCCGACC
TGCCCAGCAATGATTGGGTCATAACTCCAGAGGAACGAGCAAAGCGAGTCGACCTTCGAAACATTGTGATT
TGCTCAGTGGATCCACCTGGTTGCACTGACATCGATGACGCATTGCATTGCTTCAAACTTCCCAATGGAAAT
TACGAGGCGGGAGTGCATATTGCGGACGTAAGCCACTTCATCCGACCGGGCACAGCTCTCGACCGAGAGG
CAGCGCTCAGGGCGACAACCGTCTACCTCGTCGACAAGAGGATCGACATGGTCCCCGACTTGCTGAGTTCA
AACCTGTGCTCGCTGCGTGGCGGAGAGGAGAGGCTTGCCTTCTCCTGTATCTGGGAGCTGACGCCTGAGGC
AGAGATCGTTTCCACCAGGTTCCACAAGTCAGTGATCAAGTCCAAATCCGCAATGACTTACGAAGCAGCTC
AAGCCATCATCGACGACGCCTCGGATGACAGTGTTCTGGCGCAGTCCCTGCGCGGCCTCAACTGTCTTGCC
AAGGTTCTCAAGAAGCGCAGGATGGACAATGGGGCTCTGTCCCTCGCCTCTCCAGAGATCAGGTTTCGAGT
TGATTCTGAAACCCACGAGCCGCTAGAAGTAGTTTCAAAGAAGATGCGAGAAACCAATTCTATGGTTGAAG
AGTTCATGCTCCTTGCTAACGTGTCGACCGCAGAGAAGATATTGGAAGAATTCCCAGAAGTGGCGCTTCTT
CGACGCCACCCAGAACCCCCTCCGCCCAACTTTGATCCTCTTGTCAAAGCTGCTCACCACCAGGGTTTCGAA
ATAGACATCTCCAACAGCAAGAAACTCTCGGAGAGCCTGGATAAAGCAGTGAAAGCTAATGACAGCTACT
TCAACGTGATGCTGCGTATCTTGGCCACTCGCTGCATGCTACAAGCAGTTTACTTCGTCAGCGGCACGACCC

AGAGGTCAGAGTTCTTCCATTATGGGCTCGCCGCGCCGCTGTACACACACTTTACTTCCCCGATCAGGAGGT
ACTCTGATGTCATCGTGCACAGGCTCCTAGCAGCTTGTATCGGTGCTGACGCAACTTACCCAGATCTCATGG
ATAAGAGGAAGACAGATAAACTCTGTCAAAACTTGAACTACAGGAAGAGAATGGCCCAGTACGCTGGAAG
GGCTTCCGTCGCCTTGCACACTCACCTGTTCTTCCGAGACAGAATAGAGAACGAGGAAGGCTACGTCCTCT
TCGTGAGGAAGAACGCGCTTCAAGTGCTGATCCCCAAGTACGGGCTCGAGGGCACCCTCCTCCTCTCATCA
AAGGACAAGAACAACCCCAGCCCGTTTCTTTTCAACAACGATGATCACACGCAGCGAGCAGGGGATATTGT
TTTCCACGCGTTTGATCGTGTTGTTGTGAGGCTCTACGTCGACCGTTCTAACGTTCAACACGAGAGGCTCAT
CATGAATTTAGTTGAGCCTTCCATTCCCGGGTTTTCGGTTTGTTCTTCGGGAGACCTAGTGCCTACTGAAGA
GGAACAAGTTCCTCCTTCTTCGGGAGACCTAGTGCCTACTGAATAGGAACAACTTCCTCCTTCTCAAGTTAC
TTCTTAATGCAATCTTATATATATGTTTTTTTTTTGAAATTGTAAATATCTCTGTAAATACATTTCCTTCAAT
CTGACCATTATAACTCATAAACATTTTTTGTAAATATAAGTTTTTATCATATTTTTCTAATGCTGTTCATATA
ACTTGCCTGTTATTTATCTTTCTTTTCTTGAATAAACAAATATCTAAAAAATGGATTTGAACTTCATAACGAA
ATCTGGTGTGACATCACGGAAGTTTCCTACTTGAATAAATTTTAAAATTTTATATTGAAATGGACACAGTTA
TGTTCAATCAATAATTATGTGTGTAACAAAGCCAACATAGATCTGTGTTCTATATACATCCATATTTTAGCC
GACACATCAAG

Protein

RF: +1

ORF: 67 -> 2892

Length: 941 aa

>lcl|ORF1

MLTKKVFYKQSKKGTILKIVREHYLRKDIWCGCKGCTKCALEEKDMILDACPISKCSLLSEPHYIFLDTNAVLDQ
IDAIEDPYLENIIIPQTVLDEVKHRSSSVFKRLKEVIFDKKRKIYVFVNEHLKATYIERKVGETVNDRNDRAIRAAL
WFQEHTGVKSVLLSDDVANREKAIEKGVVAFSLSDYAEATDSATLSERLCAKRWSERRGEDSTDVFPAHLPPA
AVHHGLKAGLLLQGVFYASKENCLEATVITDKHNVLIQGRASLNRAIDGDTVAVELLPKEDWVAPSNLVIQSEE
AEDEVKVEREINEEERQVTGKIVAIIHRKWQQYCGILQLSPIKGHTHHLFVPAKRHIPKVRIETRQGEKLASQRLV
VAIDAWPRHSRYPQGHFVRALGKIGDKETENEVLLLEHDIPCSKFSDAVLADLPSNDWVITPEERAKRVDLRNIV
ICSVDPPGCTDIDDALHCFKLPNGNYEAGVHIADVSHFIRPGTALDREAALRATTVYLVDKRIDMVPDLLSSNLC
SLRGGEERLAFSCIWELTPEAEIVSTRFHKSVIKSKSAMTYEAAQAIIDDASDDSVLAQSLRGLNCLAKVLKKRR
MDNGALSLASPEIRFRVDSETHEPLEVVSKKMRETNSMVEEFMLLANVSTAEKILEEFPEVALLRRHPEPPPPNF
DPLVKAAHHQGFEIDISNSKKLSESLDKAVKANDSYFNVMLRILATRCMLQAVYFVSGTTQRSEFFHYGLAAPL
YTHFTSPIRRYSDVIVHRLLAACIGADATYPDLMDKRKTDKLCQNLNYRKRMAQYAGRASVALHTHLFFRDRIE
NEEGYVLFVRKNALQVLIPKYGLEGTLLLSSKDKNNPSPFLFNNDDHTQRAGDIVFHAFDRVVVRLYVDRSNVQ
HERLIMNLVEPSIPGFSVCSSGDLVPTEEEQVPPSSGDLVPTE

Conserved Domains

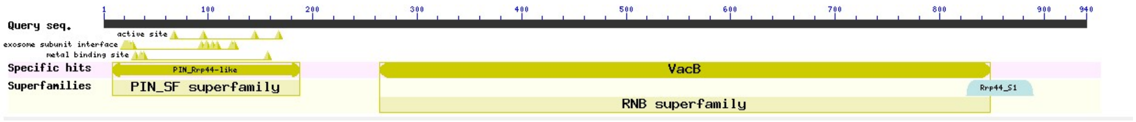

BLASTp

XP_014280828.1 exosome complex exonuclease RRP44 [*Halyomorpha halys*]

Score:1859 bits

E-value: 0.0

```
Query  1    MLTKKVFYKQSKKGTILKIVREHYLRKDIWCGCKGCTKCALEEKDMILDACPISKCSLLS  60
            MLT+KVFYKQSKKGTILKIVREHYLRKDIWCGCKGC KCALEEKD++LDACP+SKCSLLS
Sbjct  1    MLTRKVFYKQSKKGTILKIVREHYLRKDIWCGCKGCLKCALEEKDVVLDACPVSKCSLLS  60

Query  61   EPHYIFLDTNAVLDQIDAIEDPYLENIIIPQTVLDEVKHRSSSVFKRLKEVIFDKKRKIY  120
            EPHYIFLDTNAVLDQIDAIEDPYLENI+I QTVLDEVKHRSSSVFKRLKEVIFDKKRK+Y
Sbjct  61   EPHYIFLDTNAVLDQIDAIEDPYLENIVISQTVLDEVKHRSSSVFKRLKEVIFDKKRKVY  120

Query  121  VFVNEHLKATYIERKVGETVNDRNDRAIRRAALWFQEHTGVKSVLLSDDVANREKAIEKG  180
            VFVNEHLKATYIER+VGETVNDRNDRAIR+AA W+QEHTGVKSVLLSDD+ANREKA+EKG
Sbjct  121  VFVNEHLKATYIERRVGETVNDRNDRAIRKAASWYQEHTGVKSVLLSDDIANREKALEKG  180

Query  181  VVAFSLSDYAEATDSATLSERLCAKRWSERRGEDSTDVFPAHLPPAAVHHGLKAGLLLQG  240
            + AFSLSDYAEATDS TLSERLCAKRWSERRGEDSTDVFPAHLPPAAVHHGLKAGLLLQG
Sbjct  181  IFAFSLSDYAEATDSVTLSERLCAKRWSERRGEDSTDVFPAHLPPAAVHHGLKAGLLLQG  240

Query  241  VFYASKENCLEATVITDKHNVLIQGRASLNRAIDGDTVAVELLPKEDWVAPSNLVIQSEE  300
            V+YASKENCLEATV+TDKHN+LIQGRASLNRAIDGDTVAVELLPKEDWVAPSNLVI+SEE
Sbjct  241  VYYASKENCLEATVVTDKHNILIQGRASLNRAIDGDTVAVELLPKEDWVAPSNLVIESEE  300

Query  301  AEDEVKVEREINEEERQVTGKIVAIIHRKWQQYCGILQLSPIKGHTHHLFVPAKRHIPKV  360
            AEDEVKVEREINE+ERQVTGKIVAIIHRKWQQYCGILQLSPIKGHTHHLFVPAKRHIPKV
Sbjct  301  AEDEVKVEREINEQERQVTGKIVAIIHRKWQQYCGILQLSPIKGHTHHLFVPAKRHIPKV  360

Query  361  RIETRQGEKLASQRLVVAIDAWPRHSRYPQGHFVRALGKIGDKETENEVLLLEHDIPCSK  420
            RIETRQGEKLASQRLVVAIDAWPRHSRYPQGHFVRALGKIGDKETENEVLLLEHDIPCSK
Sbjct  361  RIETRQGEKLASQRLVVAIDAWPRHSRYPQGHFVRALGKIGDKETENEVLLLEHDIPCSK  420

Query  421  FSDAVLADLPSNDWVITPEERAKRVDLRNIVICSVDPPGCTDIDDALHCFKLPNGNYEAG  480
            FSDAVLADLP+NDW ITPEE AKRVDLRNI++CSVDPPGCTDIDDALHCFKLPNGNYEAG
Sbjct  421  FSDAVLADLPNNDWTITPEELAKRVDLRNIIVCSVDPPGCTDIDDALHCFKLPNGNYEAG  480

Query  481  VHIADVSHFIRPGTALDREAALRATTVYLVDKRIDMVPDLLSSNLCSLRGGEERLAFSCI  540
            VHIADVSHFIRPGTALD+EAALRATTVYLVDKRIDMVPDLLSSNLCSLRGGEERLAFSCI
Sbjct  481  VHIADVSHFIRPGTALDKEAALRATTVYLVDKRIDMVPDLLSSNLCSLRGGEERLAFSCI  540

Query  541  WELTPEAEIVSTRFHKSVIKSKSAMTYEAAQAIIDDASDDSVLAQSLRGLNCLAKVLKKR  600
            WELTP AEIVSTRFHKSVI+SK+AMTYEAAQAIIDD SD+S LAQSLRGLN LAK+LKKR
```

Sbjct 541 WELTPNAEIVSTRFHKSVIQSKAAMTYEAAQAIIDDVSDNSELAQSLRGLNTLAKILKKR 600

Query 601 RMDNGALSLASPEIRFRVDSETHEPLEVVSKKMRETNSMVEEFMLLANVSTAEKILEEFP 660
R DNGALSLASPEIRFRVDSETHEPLEVVSKKMRETNSMVEEFMLLANVSTAEKILEEFP
Sbjct 601 RTDNGALSLASPEIRFRVDSETHEPLEVVSKKMRETNSMVEEFMLLANVSTAEKILEEFP 660

Query 661 EVALLRRHPEPPPPNFDPLVKAAHHQGFEIDISNSKKLSESLDKAVKANDSYFNVMLRIL 720
EVALLRRHPEPPP NFDPL+KAAHHQGFE+D +NSKKLSESLDKA+K NDSYFNVMLRIL
Sbjct 661 EVALLRRHPEPPPANFDPLIKAAHHQGFELDTANSKKLSESLDKAIKPNDSYFNVMLRIL 720

Query 721 ATRCMLQAVYFVSGTTQRSEFFHYGLAAPLYTHFTSPIRRYSDVIVHRLLAACIGADATY 780
ATRCMLQAVYFVSG TQRSEFFHYGLAAPLYTHFTSPIRRYSDVIVHRLLAACIGADATY
Sbjct 721 ATRCMLQAVYFVSGVTQRSEFFHYGLAAPLYTHFTSPIRRYSDVIVHRLLAACIGADATY 780

Query 781 PDLMDKRKTDKLCQNLNYRKRMAQYAGRASVALHTHLFFRDRIENEEGYVLFVRKNALQV 840
PDLMDKRKTDKLCQNLNYRKRMAQYAGRASVALHTHLFFRDRIENEEGY+LFVRKNALQV
Sbjct 781 PDLMDKRKTDKLCQNLNYRKRMAQYAGRASVALHTHLFFRDRIENEEGYILFVRKNALQV 840

Query 841 LIPKYGLEGTLLLSSKDKNNPSPFLFNNDDHTQRAGDIVFHAFDRVVVRLYVDRSNVQHE 900
LIPKYGLEGTLLLSSKDK NPSPF+FNNDDHTQRAG+IVFHAFDR+VVRLYVDRSNVQHE
Sbjct 841 LIPKYGLEGTLLLSSKDKKNPSPFVFNNDDHTQRAGNIVFHAFDRLVVRLYVDRSNVQHE 900

Query 901 RLIMNLVEPSIPGFSVCSSGDLVPTEEEQVPPS 933
RLIMNLVEPSIPGFSVCSSGDLVPTEEEQVPPS
Sbjct 901 RLIMNLVEPSIPGFSVCSSGDLVPTEEEQVPPS 933

Nibbler

>TRINITY_DN76599_c0_g1_i1 len=3136 path=[0:0-3135]

ATTAAGTATTGGGACATGCGTCTTCAAGTTGAAAATTTGTTGCTTTGACAAAAATCAAACAGACGTAAATT
ATTATTTAAAAAATGGTGGTTATACTTAGGGTGTGTTTTTCTTATTGAGAAATGCTTAGATTGAAACCACAG
TCGGCAAAATGCATCCTCTCGCAAAGGTGGAACACTTCCAACCCAGACCGTTCATTAATACTGATAACATA
ACTGTTATCAAACAGTGGGAACTGGAAGAATGAGATCTCTACAGTAATTTTTAACTATGAAAACATAATAG
AGCTTTGGTCTGATTCAATGGATGGGATGGCAGGCTCTTCTACAGATTTTAGGAGCACCTTGGCTAAAGTTT
GGAATCAGTGTAAAAGATGTGATGCATTGTACCGAATGCTTCAACACCATTTTGAAAGTAGTGGGAATCCT
TATGAAAGCGTATTAGTTTTAATCAGGCAGTCCCCTGACTATTGCCTCTCTCGACCAAATGGTCTTTCATTT
ACCATTATTGAACGATTTGCCAGTTGGATTAATTATAGAAGAGAAAATTACAAACACTGCCTTACGGAAGA
TGTGAAACGTGAAGCTTTTCAAGTAGCCTCCAGACAAAGAAATAAAGCTATAAGCAAGAATATTATCGATG
TTTATCTTCTTAAAGACTACAAGCATATGCTTGTGAAATCCCTGAATGACAATCTTCAGCTTCATATGTACA
AAGAAGTTTGCGAATGGTCTATGATGTTGGAATTGGAATCTGAATTTGGTATTTTTGATTTTATTGTTCCACT
TATTTTCCAAGATAAACTTACGATTGTTGACGAATTTCTCAAGAAAAACCGTTCTCATCATCTAGAACTGAT
AAAATTTCTAGATAACGTTCTGGCCCAGAGATGTATTTCGAACTTCATGGATCAACTAATAATAGACTTGAA
AATAGAAAACGTGAAGCATGGTTCCCTGCCCAAGCTGTCTCAGACAAAGCCCTTGTGTAAGCTGCTAAAGA

GGTTTGCCTCGATCCACAATCTCCCCCCCGACAGTATGCCCAACCTCACACTCAGGAATGGCATAGGCACT
GTTAACTTCCTCATCCGCAAAAGATATTATGACCGCAGCTTAAGCAAAGAAGCATTCCGAGAGCTGGTAAA
GGATGCTTGCGGGTCAAGAAGAGAAGTGATGATCCACTTGGTCAACAGGCTCGACGAAGAGGATGATGCC
ACCGAGGCCTTGATCTGTGCCCGCCTTTTCTCTCTTCCTCCTTCTGACATGCCTCAGAACGTCAGATCTCTTG
ATGAGAATGGCTGGAGGGAAGACGATTCACCGATTTCGGACAAAGGAGTGGAAGAAGGGCTAGACCTCAG
TGATGAATATCACAAATTACCCCTGCAACTTTCTTGTATACACATTATTGACACAGAAGCAGCTTTTTCTTC
TCTCATTGATTCCTACATTGACGGAGCTACTACGATAGGGATCGACTTGGAGTGGAAGCCTACGATGGTGG
CACCCGCCGGTGAGCTGGCCCTGCTCCAGCTGGCCAAGGAGGACAAGGTCTTCCTCGTGGACGTCCTCTCT
CTAGCCAACTCCCATCACCTATGGGGGCAGTTCGCACACGTCTTTCTTAATAACCATGACATTCTCAAAATA
GGTTTTGCTATGGGCGCTGATTCAGCCATGTTGGCGCAATGTTTTCCCATTGACTTCGCTTTTCGAATGTCTG
GGCTTGGCTTTTTGGATCTCTCCTCTCTCTGGAATAAACTTGTGAAGGATTACAATTTTGAGTTTCCATATGA
AGCTAACGAAACCTGTTCGAGCTGTAGCTTGAGCCAACTTGTCGCACTTTGTATTGGAAAGCCACTCAACA
AGAGAGAACAGTTTTCCAATTGGGAGACGCGTCCATTAAGGCTGACCCAAAAGGTTTATGCTGCATTGGAT
GCTTACTGTCTAATTGAAGTGTATGAAGTAATCAAAAGGCTGTGCTTGGAAAGGAACATCCCCCTCGATGA
TATTGTTCTAGGGATGATGGCGAGGACTGAACAAGGGAAGGCCAAGAAATCAAAATCAAACAAAAAAAGT
AAAGAAGCCATGAGATACAGATCTACAGCTACAGATGCCAAAGAGATGAAGCTGGTGTGCGAGCCTAGCC
TAGCAATGCTGGCCAGCGTACTGCGCAGGCACGGCATAGACACTGTTCTGGCTGATCTCGAGGACATTGCT
GACATCGCCGAGTCTGAAAACAGGATAGCGCTATCTTCTTCCTGGCCTTTCAAAACGATTTCCGAGCAGTTG
CCGAAAGATAAATGCTACTTGGTTCGGTCATGCGCCTCCCCCGACCAGTACAGGGAAGTCGTTAGGCAGCT
CCACATCATTGTTAAGCCAGAGTACATTCTCTCAAGGTGCCAGGAATGCAACAGTGAGCACGTAGTGATGG
TAAGCAAGAAAACTGTCGAAGATTGGTTCGTTGAGAAAGTGAAGCCTAAGGCTGTGAACTGCGTCCCCGCT
GGCTACTCTGACAATGAGTTCAGCGACTCGGAAGAGGATGAGTATTACAGGCTCACCGTGACTGAGTGTCC
TCCTGCCACGGTCGAAAACGGTTCAAAGTTCTGCCCGACTACCTCAACAGGAGTTCCAATCAGGATGGACC
TCATTGTGGAAGAGCAAGTTAAATCTGCGAGGCTGGTCTGCCTCTGCGAGGATTGCGGGCGGGTGTTCTGG
CAAGCAGAGGCCAAATAGAAGCCTCCTTTGCGAGAATAAACTCTAACCACCAAATTGCTCTTATAACATTT
CTTCCCGTTCCCATATTATTTGTGATTAATCCCATCTGTGTTCCTTTTGATATCGACGTTCTCATTATAGGAG
GAGAAAGAAATGAAAAGTATAATCTATATATATATATATTTAATTATAAGTGAAGTTATATAACTTTTTAAG
ACAATAAGATTATTTAATTAAAATATCAACTGTATTTTAATAGAGTTTTAAGAATTTTATTTACTAAAAATT
ATATTTTTTTGTTACACTGTTTGTTCTTTTATTCTTTGTTCTGTAAAATATTTTTAATGTTTTGTAAACATTTTT
CGGAAATCGAAAAATTAAATTTGAATTTATTTACAAATTATTTTAAATATAATAGCACATCG

Protein

RF: +3

ORF: 303 -> 2729

Length: 808 aa

>lcl|ORF8

MDGMAGSSTDFRSTLAKVWNQCKRCDALYRMLQHHFESSGNPYESVLVLIRQSPDYCLSRPNGLSFTIIERFAS
WINYRRENYKHCLTEDVKREAFQVASRQRNKAISKNIIDVYLLKDYKHMLVKSLNDNLQLHMYKEVCEWSM
MLELESEFGIFDFIVPLIFQDKLTIVDEFLKKNRSHHLELIKFLDNVLAQRCISNFMDQLIIDLKIENVKHGSLPKLS
QTKPLCKLLKRFASIHNLPPDSMPNLTLRNGIGTVNFLIRKRYYDRSLSKEAFRELVKDACGSRREVMIHLVNRL
DEEDDATEALICARLFSLPPSDMPQNVRSLDENGWREDDSPISDKGVEEGLDLSDEYHKLPLQLSCIHIIDTEAAF
SSLIDSYIDGATTIGIDLEWKPTMVAPAGELALLQLAKEDKVFLVDVLSLANSHHLWGQFAHVFLNNHDILKIGF
AMGADSAMLAQCFPIDFAFRMSGLGFLDLSSLWNKLVKDYNFEFPYEANETCSSCSLSQLVALCIGKPLNKREQ
FSNWETRPLRLTQKVYAALDAYCLIEVYEVIKRLCLERNIPLDDIVLGMMARTEQGKAKKSKSNKKSKEAMRY
RSTATDAKEMKLVCEPSLAMLASVLRRHGIDTVLADLEDIADIAESENRIALSS

SWPFKTISEQLPKDKCYLVRSCASPDQYREVVRQLHIIVKPEYILSRCQECNSEHVVMVSKKTVEDWFVEKVKP
KAVNCVPAGYSDNEFSDSEEDEYYRLTVTECPPATVENGSKFCPTTSTGVPIRMDLIVEEQVKSARLVCLCEDCG
RVFWQAEAK

Conserved Domains

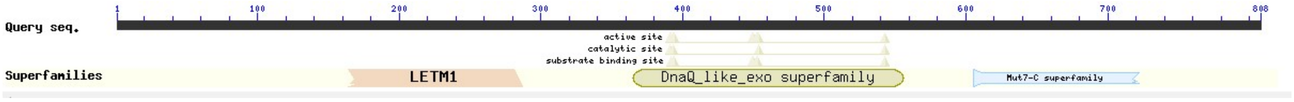

BLASTp

XP_024216394.1 exonuclease mut-7 homolog [Halyomorpha halys]

Score:1436 bits

E-value: 0.0

Query 1 MDGMAGSSTDFRSTLAKVWNQCKRCDALYRMLQHHFESSGNPYESVLVLIRQSPDYCLSR 60
MDG+AG S DF STLAKVWNQCKRCDAL RMLQHHF+S+ NPYESVLVLIRQSPDY LSR
Sbjct 1 MDGIAGCSADFSSTLAKVWNQCKRCDALDRMLQHHFDSNKNPYESVLVLIRQSPDYSLSR 60

Query 61 PNGLSFTIIERFASWINYRRENYKHCLTEDVKREAFQVASRQRNKAISKNIIDVYLLKDY 120
PNGLSFTII++FASWI+YRR NYKHCLTE+VKREAFQVASRQRNK ISKNIIDVYLLKDY
Sbjct 61 PNGLSFTIIKQFASWISYRRANYKHCLTEEVKREAFQVASRQRNKTISKNIIDVYLLKDY 120

Query 121 KHMLVKSLNDNLQLHMYKEVCEWSMMLELESEFGIFDFIVPLIFQDKLTIVDEFLKKNRS 180
K + VKSLNDNLQLHMYKEVCEWSMML+LESEFGI DFIVPLIFQDKLT+VDEFLKKNR+
Sbjct 121 KQLFVKSLNDNLQLHMYKEVCEWSMMLDLESEFGISDFIVPLIFQDKLTVVDEFLKKNRT 180

Query 181 HHLELIKFLDNVLAQRCISNFMDQLIIDLKIENVKHGSLPKLSQTKPLCKLLKRFASIHN 240
HHLELIKFLDN+L+QR ISNF+D LIIDLKIEN+KH SLPKLSQTKPLCKLLKRFASIHN
Sbjct 181 HHLELIKFLDNILSQRSISNFIDHLIIDLKIENIKHASLPKLSQTKPLCKLLKRFASIHN 240

Query 241 LPPDSMPNLTLRNGIGTVNFLIRKRYYDRSLSKEAFRELVKDACGSRREVMIHLVNRLDE 300
+PPDSMPNLTLRNGIGTVNFLIRKRYYDRSLSKEAFRELVKDACGSRREVMIHLVNRLDE
Sbjct 241 IPPDSMPNLTLRNGIGTVNFLIRKRYYDRSLSKEAFRELVKDACGSRREVMIHLVNRLDE 300

Query 301 EDDATEALICARLFSLPPSDMPQNVRSLDENGWREDDSPISDKGVEEGLDLSDEYHKLPL 360
E D TEALICARLFS+P SDMP +VR+LDENGWRED+ ISD+ VEE LD SDEYHK PL
Sbjct 301 EGDPTEALICARLFSVPSSDMPPSVRALDENGWREDNLAISDELVEERLDHSDEYHKFPL 360

Query 361 QLSCIHIIDTEAAFSSLIDSYIDGATTIGIDLEWKPTMVAPAGELALLQLAKEDKVFLVD 420
QLS I IIDTEAAFSSLIDS IDGATTIGIDLEWKPTMVAP GELALLQLAKEDKVFL+D

Sbjct 361 QLSSIFIIDTEAAFSSLIDSSIDGATTIGIDLEWKPTMVAPTGELALLQLAKEDKVFLID 420

Query 421 VLSLANSHHLWGQFAHVFLNNHDILKIGFAMGADSAMLAQCFPIDFAFRMSGLGFLDLSS 480
VLSLANSHHLWGQFA+ FLNNHDILKIGFAMGADS MLAQCFPIDFAFRMSGLGFLDLSS
Sbjct 421 VLSLANSHHLWGQFANAFLNNHDILKIGFAMGADSTMLAQCFPIDFAFRMSGLGFLDLSS 480

Query 481 LWNKLVKDYNFEFPYEANETCSSCSLSQLVALCIGKPLNKREQFSNWETRPLRLTQKVYA 540
LWNKLVKDYNFEFPYEANETCSSCSLSQLVALCIGKPLNKREQFSNWETRPLRLTQ+VYA
Sbjct 481 LWNKLVKDYNFEFPYEANETCSSCSLSQLVALCIGKPLNKREQFSNWETRPLRLTQRVYA 540

Query 541 ALDAYCLIEVYEVIKRLCLERNIPLDDIVLGMMARTEQGKAKKSKSNKKSKEAMRYRSTA 600
ALDAYCLIEVY+VIKRLC ERNIPLDDI+ MM RTEQGKAKKSK +KKSKEAM++RS
Sbjct 541 ALDAYCLIEVYQVIKRLCAERNIPLDDIIQDMMTRTEQGKAKKSKKDKKSKEAMKHRSVP 600

Query 601 TDAKEMKLVCEPSLAMLASVLRRHGIDTVLADLEDIADIAESENRIALSSSWPFKTISEQ 660
T+AK+MKLVCEPSLAMLA++LRRHG+DT+LA+L DIADIA SENRIALSSSWPFKTISEQ
Sbjct 601 TEAKKMKLVCEPSLAMLATILRRHGVDTILAELHDIADIAVSENRIALSSSWPFKTISEQ 660

Query 661 LPKDKCYLVRSCASPDQYREVVRQLHIIVKPEYILSRCQECNSEHVVMVSKKTVEDWFVE 720
LPKDKCYLVRSCAS DQYREV+RQL+IIVKPE ILSRCQ+CNS+HV M++KKTVEDWF++
Sbjct 661 LPKDKCYLVRSCASSDQYREVLRQLYIIVKPEDILSRCQDCNSDHVTMINKKTVEDWFLQ 720

Query 721 KVKPKAVNCVPAGYSDNEFSDSEEDEYYRLTVTECPPATVENGSKFCPTTSTGVPIRMDL 780
K K KA NCVPAG+SDNE +DSEED+Y+ L ++C P +ENGSKF PTT TGVPIR+DL
Sbjct 721 KGKSKAENCVPAGFSDNELTDSEEDDYFTLDTSDCTPNAIENGSKFYPTTMTGVPIRLDL 780

Query 781 IVEEQVKSARLVCLCEDCGRVFWQAEAK 808
+ EEQ+K A L+ LCE CGRVFWQAE K
Sbjct 781 VAEEQIKLASLISLCEKCGRVFWQAETK 808

dna/rna non specific endonuclease PolyApolymerase

>TRINITY_DN5556_c0_g1_i1 len=2513 path=[2:0-133 11:134-134 12:135-135 16:136-276 18:277-295 19:296-1103 21:1104-1865 22:1866-1922 24:1923-2070 25:2071-2512]

GTCTGGTAAGCTGTAGGATTGTCAACAACATAATTCAGAACTGTAGACAAAGCTATTTTTAGCTACTTTAG
TGTGGATTGTAGTTTGTTAACTTTAATATATTGCTCGGTCTTAGCTGGTGCTTCCTGAACTGGGGAGCTTCAT
AATTCTTCGTTAATTGAAGGACCTTTCTTTATCGGTGAGGCAATGAACACAGTACTGATTTAGAGTTTCATC
TGAATCGAGCATCATTTAACATAAGGAGTGTCGAAGATAACAAAACAAGTGGAAGTCTGAGTGCAAGGTG
CAAGTTCAAATGGGGAATTAAAGAACAGGACACCTAATGACATGAAAAGACTTCTGTAATGAACCCTACC
CACTGAACATGCTTACTGGCCGCTGTATTGAGGAAAGGTATGTGGTCGTCGCAGGGCTCAGCGCAACCTGC
TCCACAGCAGCAGCACCAGCAACAACAGCGCACTCTAGGGCTTACATCAGCCATTACTCTTGCTGGTCCCA
AGCCTATCGACTTCCAGAAGACTAATGAACTTATAGAGACCCTTAAACCATACAATGTTATGGAAACTGAG
GAGGAACTGAATCACAGGATGGAAATTTTAAGCAAACTTAACAGTCTAGTAAAGGAATGGATTAGAGAGG
TGAGCATAAGCCGTAACATGCCTGAAAGTGTTGCTAATTCAGTTGGTGGCAAGATCTACACATTTGGCTCA
TATCGCCTTGGTGTCCATCATAAAGGTGCCGATATTGATGCTCTCTGTGTAGCCCCCAGGCATATTGACAGG

TCTGATTACTTCACATCATTCTTCGAAAAGTTAAAGTTGCATACCGAAGTAACTGACTTGAGGGCAGTGGA
AGAAGCTTTTGTTCCTGTGATAAAAATGAATTTTGATGGAATTGAAATAGATATGCTGTTTGCTCGGCTTGC
TCTAAAAGAAATTCCTGATTCAATGGATCTCAAAGATGACATGTTGCTCAAGAATCTTGATCAAAAATGTG
TGAGAAGTCTGAATGGCTGCAGGGTCACTGATGAGATATTACGATTAGTACCTGATATAGAAAATTTCCGC
CTCACTCTTAGAACCATAAAGCTGTGGGCAAAAAAACATGGTGTATACAGCAATGTGATGGGATATTTAGG
TGGAGTTAGTTGGGCTATGCTGGTAGCTCGAACATGCCAGTTGTATCCCAATGCTGTCGCAGCAACCCTTGT
TCACAAGTTTTTCTTGGTTTTTCCCAATGGAAGTGGCCGCAGCCTGTTCTGCTAAAACAGCCAGACCAGGT
CAATCTTGGATTTCCAGTGTGGGATCCTCGAGTGACTGTTTCGGACCGATACCATCTTATGCCTATCATCAC
TCCTGTATACCCTCAGCAGAATTCAACTTTTAATGTTTCTGCATCTACAAGAACCATTCTGCAGGAAGCTTT
TAATAATGGTCTATCAATCACGGATGAAATATTTAATGGGAAGGCTTCATGGGACAAATTGTTTGAAGCAC
CCAATTTTTTCTTAAAGTACAAACACTTTATTGTATTATTGGCTAAGTCTGCAAGCCAAGAAGAACAGTTGG
AATGGTGTGGATTAATAGAATCTAAGGTGCGCCATTTAGTTGGTAATCTTGAACGCAACCAGTATATTAGC
CTGGCCCATGTCAACCCGGAATGTTTTCCTGGACCACCAACACCCCCGGGAGAACCACTAGTACCCACTTC
AATGTGGTTTATTGGCATTGTCTTCAGGAAGTTGGAAAATCTCAATGTTGACCTCACCTATGATATTCAGTC
TTTCACCACTTCTGTTCATCGACAAGCTTCAGCCATTAAGATGATTAAGGATTCCATGAAAATTGAGGCTAG
GCATGTTAAAAGAAAACAGCTTAGTGCTTACCTGCCGGCGCATATTTTGAAGCGAGAAAAAAAGGTTGCAG
CAGCCAGAAACAGTACCTCTCCAATTCGAAGAAATGGTGTGTCTCAACTCCAGGTTGGTGATGCCAACAAA
AAGCGTCATTCTGACTCCTCTCTCGACTTGTTGCAGAAGAAACAGAAGCTCCATCAAGAACTACCGGAAAT
GGATGGGGATCCACCAATGAATAGGATTGTTGTTTCATGTGGTGAAGATTCAAATTCATCACTATCTGCCGA
TGAACATAATAGAAACCACGAGATACTTGACTCTAGTAAATCAACTTTAGAGAACAAGAAACCTGACAGT
ATACAGGAAGAGGTTATCTGTTTATGAAAAGGATATATGGAATCAGAATTACCATAGTTGACTGAGGTGGT
TTCACCACTGGGGGTCTATATATATGTATCTTTTATATATAGATAATGCTACCTCTGTACCTGCTGAGGTGA
AAGAGTTGCTCAGACCTTTATCTCTAGTGACGGTAAGTATGATGGGGTTCTGAACAATGGATGGCATCGGA
CAATTTCAATATTATAATGTTCTATACTTTTTCTTTTTGTAATTATGTGTATATATATATATATATGTGTGTGT
GTGTGTGTGTGTGT

Protein

RF: +1

ORF: 397 -> 2238

Length: 613 aa

>lcl|ORF5_TRINITY_DN5556_c0_g1_i13:235:2076 unnamed protein product

MWSSQGSAQPAPQQQHQQQQRTLGLTSAITLAGPKPIDFQKTNELIETLKPYNVMETEEELNHRMEILSKLNSLV
KEWIREVSISRNMPESVANSVGGKIYTFGSYRLGVHHKGADIDALCVAPRHIDRSDYFTSFFEKLKLHTEVTDLR
AVEEAFVPVIKMNFDGIEIDMLFARLALKEIPDSMDLKDDMLLKNLDQKCVRSLNGCRVTDEILRLVPDIENFRL
TLRTIKLWAKKHGVYSNVMGYLGGVSWAMLVARTCQLYPNAVAATLVHKFFLVFSQWKWPQPVLLKQPDQV
NLGFPVWDPRVTVSDRYHLMPIITPVYPQQNSTFNVSASTRTILQEAFNNGLSITDEIFNGKASWDKLFEAPNFFL
KYKHFIVLLAKSASQEEQLEWCGLIESKVRHLVGNLERNQYISLAHVNPECFPGPPTPPGEPLVPTSMWFIGIVFR
KLENLNVDLTYDIQSFTTSVHRQASAIKMIKDSMKIEARHVKRKQLSAYLPAHILKREKKVAAARNSTSPIRRNG
VSQLQVGDANKKRHSDSSLDLLQKKQKLHQELPEMDGDPPMNRIVVSCGEDSNSSLSADEHNRNHEILDSSKST
LENKKPDSIQEEVICL

Conserved Domains

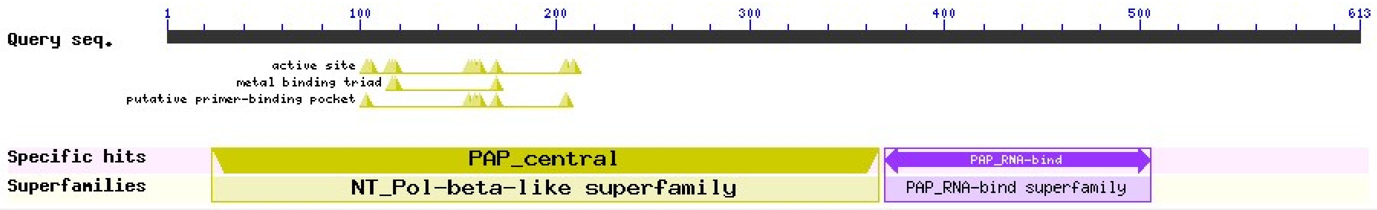

BLASTp

EFA00912 Poly(A) polymerase gamma-like Protein [Tribolium castaneum]

Score:777 bits

E-value: 0.0

```
Query  1    MWSSQPVNNGTQNNKENVTQKNDTKIPTLGMTSAISTAPPKPSDLLKTQELEEALKPFGV  60
            MWSSQ    G+        +  +  TLG+TSAI+ A PKP D  KT EL E LKP+ V
Sbjct  1    MWSSQ----GSAQPAPQQQHQQQQR--TLGLTSAITLAGPKPIDFQKTNELIETLKPYNV  54

Query  61   FESEQELNHRMVILGKLYSLVKQWIKDVSISKNMPESVAENVGGKIYTFGSYRLGVHNRG  120
             E+E+ELNHRM IL KL SLVK+WI++VSIS+NMPESVA +VGGKIYTFGSYRLGVH++G
Sbjct  55   METEEELNHRMEILSKLNSLVKEWIREVSISRNMPESVANSVGGKIYTFGSYRLGVHHKG  114

Query  121  ADIDALCVAPRHISRNDFFGSFYELLKKQPEVTDLRAVEEAFVPVIKMNFDGIEIDMLFA  180
            ADIDALCVAPRHI R+D+F SF+E LK   EVTDLRAVEEAFVPVIKMNFDGIEIDMLFA
Sbjct  115  ADIDALCVAPRHIDRSDYFTSFFEKLKLHTEVTDLRAVEEAFVPVIKMNFDGIEIDMLFA  174

Query  181  RLLLKEIPDSMDLRDDLLLKNLDQKCVRSLNGCRVTDEILRLVPNVDNFRLALRAIKLWA  240
            RL LKEIPDSMDL+DD+LLKNLDQKCVRSLNGCRVTDEILRLVP+++NFRL LR IKLWA
Sbjct  175  RLALKEIPDSMDLKDDMLLKNLDQKCVRSLNGCRVTDEILRLVPDIENFRLTLRTIKLWA  234

Query  241  KRHGIYSNALGYLGGVSWAMLVARTCQLYPNAAPATLVHKFFLVFSQWKWPQPVLLKQPS  300
            K+HG+YSN +GYLGGVSWAMLVARTCQLYPNA  ATLVHKFFLVFSQWKWPQPVLLKQP
Sbjct  235  KKHGVYSNVMGYLGGVSWAMLVARTCQLYPNAVAATLVHKFFLVFSQWKWPQPVLLKQPD  294

Query  301  NVNLGFAVWDPRVNIQDRYHLMPIITPAYPQQNSTFNVSGSTRQIIMEEFKLGLQLTDDI  360
             VNLGF VWDPRV + DRYHLMPIITP YPQQNSTFNVS STR I+ E F  GL +TD+I
Sbjct  295  QVNLGFPVWDPRVTVSDRYHLMPIITPVYPQQNSTFNVSASTRTILQEAFNNGLSITDEI  354

Query  361  MLSKQTWDKLFEPPLFFMKYKHFIVLLVSAESPEDHLEWCGLVESKFRLLIGTLERNQHI  420
              K +WDKLFE P FF+KYKHFIVLL  + S E+ LEWCGL+ESK R L+G LERNQ+I
Sbjct  355  FNGKASWDKLFEAPNFFLKYKHFIVLLAKSASQEEQLEWCGLIESKVRHLVGNLERNQYI  414

Query  421  TLAHINPESFSLLESQRESN-THCSMWFIGLEFAKSENLNVNLTFDIQQFTETVQNHALN  479
```

```
      +LAH+NPE F    +         SMWFIG+ F K ENLNV+LT+DIQ FT +V   A
Sbjct  415  SLAHVNPECFPGPPTPPGEPLVPTSMWFIGIVFRKLENLNVDLTYDIQSFTTSVHRQASA  474

Query  480  ISMLKEGMKLEARHVKRKQLYQYLSPSLLKRERKTSITVKSQSN-----------GTDSK  528
      I M+K+ MK+EARHVKRKQL  YL   +LKRE+K +    S S          G  +K
Sbjct  475  IKMIKDSMKIEARHVKRKQLSAYLPAHILKREKKVAAARNSTSPIRRNGVSQLQVGDANK  534

Query  529  KRLSDPGNSDSDNPNKKIRLSEEMHSTYDDSSNNSTSLNCNEDSNSNISLTE  580
      KR SD   S  D   KK +L +E+    D   N   ++C EDSNS++S  E
Sbjct  535  KRHSD---SSLDLLQKKQKLHQELPEMDGDPPMNRIVVSCGEDSNSSLSADE  583
```

Antiviral genes

Ars

>TRINITY_DN4735_c0_g1_i1 len=2848 path=[0:0-567 2:568-2847]

ATTGTTTTAGAAGGCTGTGTCCTAATCACAGTTTACGTTCGACGATTCTCAGGAGGCGTTTCGTTTACAGTG
TCGGTACTTTTCATTTGCTCGTTGCAAATAGAAAGTATAAGTCATTATGGGAGATAGTGAAGACGAATATG
ACAAAAAAAGGCGAGATAAATTCCAAGGTGAAAGAAGTGATTCATATAGGACGGATAAACGCAAAGACG
ATGACTGGGGAAGACCGAGGATGAGAAATGATTATCGCGATAGGTACCAGTATCAAGGTGATTTGCCACC
CACAAAGCGGATGAGATATGACTCGGATGATATTAGGAGGATGAGGTATAATGATCCTGGGTACGGTCCAT
ACAACAGTTGGGGCCCTGAACCACCCTATCCTGGAAATTCAAGGCTAGGTGAAATGGATACACAGCCTCCT
ATCATGACTTTCAAAGCTTTTCTTCAATCTCAAGATGACAACATAACTGACGAAGAAGCTATTGCCAAGTAT
GCCGAGTACAAACTTGAATTTAGGCGTCAACAGCTGAATGAATTTTTTGTGGCTCATAAGGAAGAAGAATG
GTTCAAATTAAAATATCATCCTGAAGAATCTCTTAAGAGAAAAGAGGAGCTTAACAATGCACTGAAGAAA
AGATGTGATGTTTTTCTTGAAATGTTGGAATCTAAACGCATGGATGATATAAGAGTAGATACTGAACAAGG
AGATGAAGTTGTTAAACTTCTAGATTCTGTTGTCATTAGGCTTGAAGGAGGAACTGACCTGGATTTAACTAT
TCTTGACCAACAAGAAGGACAGAAAGATGAGCAAAAAGAAAAGGATAAGAGTGATGACAAACCTAAAGA
AAATAACAAATCAGATGAAGAAGAAGGAAAAAAGAAGGAAAATGAAGAGAATAAAGACGAAATAGTAAT
TGACGAAGATGATGAGCGTAAAGAGGAAGAAGAAAAGAATGAAGTTCATGAATTATCTGAAGATGAAAGA
CCACCAGGAGTTGATCCTGAGAGTGATATTGAAAAAGAAGATACGCCTGTTGAGAAAGAATCACAATCTG
AAAAAGAGCCGCAACCTGAAGAAGAAAAAAGGGAATCATCGCCTGAGAAGGAAAAACAAGAGGAAGTAA
AGAAATCTAAAGATGACCAAGATTCTGATAAGGATCATGATGAAGTTGTTCCATTAACACCAAGAGCTCTA
CACAAAACTTCATCGATTTTTCTTCGGACTATTTCTCCTAGAGTTACCAAGGCTGAAATTGAATCGATTTGT
AAAAAGTATCCAGGATTTCTCAGAGTATCCCTTTCTGAGCCTCAAGCAGAGCAGCACTGGTATAGAAGAGG
ATGGGTTACGTTTAGGCGTGATGTAAATATAAAAGAAATCTGTTGGAATTTGAATAACATAAGGGTAAAAG
AGTGTGAAATGGGAGCAATAGTGAATCGGGATCTATCACGTAGAGTTAGATCAGTGAGTGGACTTTCAGGA
CACAAACAGGTTATCTTGGCTGACTTAAAATTAGCTGCAAAAATCATACAAGAATTGGACTCGAGAGCTGG
TTTCTGCACTCCTGATCAGAGTGAACCTTTTGGATTGGAATCAAGAAATGCTGTCTTTAAAGGTATCACTGA
ATATCTGGTAGAAGAAGCCCCTGCAGAAGAAGAAGAACTTCTGGGTCAAGGAATAGTCACTGATGAATCT
AAGTCTAGAATAGCAGGTGCAATCAAGATTTTAGATAAACTGCTTCTATATTTGAGGATTGTCCATTCTGTT
GATTACTACAACACTTCACAGTACACATCAGAAGACGAAATGCCTAATAGATGTGGCATAATGCACTTAAG
AGGACTACCTTCTAGTACGGAGGTTTCTCCTCAAGAAATACAAGAATATATAGAAGGTTATAAAACAAAAC
TTGAGCCCTTGTATACTCCAGTGCCGACTGTTTCTGAACAAGAACTATCATCATTAGGTGCTAAGGATAGAG
ATACTGAAGTCGAAAAATTCATACAAGCTAATACCCAGGAATTAGCCAAAGACAAGTGGTTATGTCCTTTA
TCAGGAAAAAAATTCAAAGGACCAGAATTTGTCAGGAAACATATTTACAATAAGTTTTCTCAAGAACTTGA
AGAAGTAAAAAAGGAGGTTGATTATTTTAATAATTATCTCAGAGATCCGAAAAGGCCTCAGTTAGCTGAAC
ACCCTGGAAATAGAGGTGGCAAAAAGGAGCCTGAATCTCCTTACCATTACCAGTATGGTGGTGGTTTTAAA
CGTGGATTTGGCCATTTTGGTGGTGGTGGTGGACATGGTGGTTTCAACCGTGGGCGAGGAGGTTTCGGAAG
AGGAAGAGGTATGGACTACAGACCTATTATTACTTATAGGGACTTGGATGCCCCATGTGAGCCAGATGAAA

TTATATAAATGCAGTCTTACTTTTATTTATTATAACCTTAATTATCTATATGTGTAATAAACATGAATACAAT
TGTGATTTGCATTTATACATTTATCATTGTGCATTAAAACAATTTTTTATTAAATCGACTAAATTATATTTG
CTTTTGGTTTCTTTTGTTCCTTTTAAAATAATATTTCAATTTTATAGTTTAAAAAAATTAATGATATAAGAGT
AGAGATGCAAAAGACCAAAGCATTATTATTGCCCACCATGTAGAAGTTTCATTATTATATGTTTTGTTTTTT
GTAAGAAATTTTAACTTTTGAAAAATAAAATTTACTTATTGAAAATTGTTTCATTTACTAAGTATACGTAAT
TCTATTTTTATTTTTTATTTTATTTTTTTAATTTCTGTTGTCATTGTAATTTTCACTAATTGATTATAAAATATC
GG

Protein

RF: +2

ORF: 119 -> 2416

Length: 765 aa

>lcl|ORF2

MGDSEDEYDKKRRDKFQGERSDSYRTDKRKDDDWGRPRMRNDYRDRYQYQGDLPPTKRMRYDSDDIRRMR
YNDPGYGPYNSWGPEPPYPGNSRLGEMDTQPPIMTFKAFLQSQDDNITDEEAIAKYAEYKLEFRRQQLNEFFVA
HKEEEWFKLKYHPEESLKRKEELNNALKKRCDVFLEMLESKRMDDIRVDTEQGDEVVKLLDSVVIRLEGGTDL
DLTILDQQEGQKDEQKEKDKSDDKPKENNKSDEEEGKKKENEENKDEIVIDEDDERKEEEEKNEVHELSEDERP
PGVDPESDIEKEDTPVEKESQSEKEPQPEEEKRESSPEKEKQEEVKKSKDDQDSDKDHDEVVPLTPRALHKTSSIF
LRTISPRVTKAEIESICKKYPGFLRVSLSEPQAQHWYRRGWVTFRRDVNIKEICWNLNNIRVKECEMGAIVNRDL
SRRVRSVSGLSGHKQVILADLKLAAKIIQELDSRAGFCTPDQSEPFGLESRNAVFKGITEYLVEEAPAEEEELLGQ
GIVTDESKSRIAGAIKILDKLLLYLRIVHSVDYYNTSQYTSEDEMPNRCGIMHLRGLPSSTEVSPQEIQEYIEGYKT
KLEPLYTPVPTVSEQELSSLGAKDRDTEVEKFIQANTQELAKDKWLCPLSGKKFKGPEFVRKHIYNKFSQELEEV
KKEVDYFNNYLRDPKRPQLAEHPGNRGGKKEPESPYHYQYGGGFKRGFGHFGGGGGHGGFNRGRGGFGRGR
GMDYRPIITYRDLDAPCEPDEII

Conserved Domains

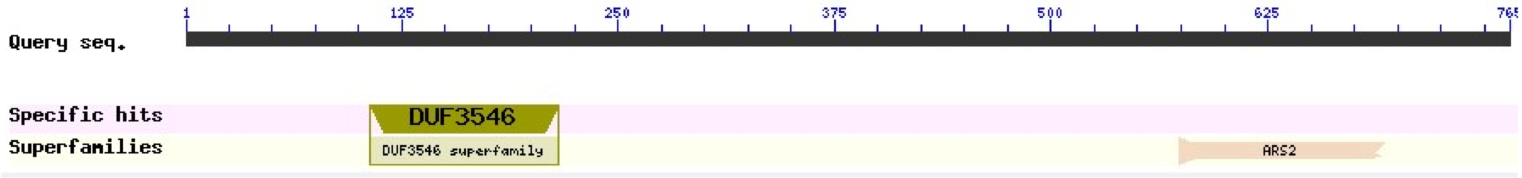

BLASTp

XP_014277995.1 serrate RNA effector molecule homolog isoform X1 [*Halyomorphahalys]*

Score:1507 bits

E-value: 0.0

Query 1 MGDSEDEYDKKRRDKFQGERSDSYRTDKRKDDDWGRPRMRNDYRDRYQYQGDLPPTKRMR 60

MGDSEDEYDKKRRDKFQGERSDSYRTDKRKDDDWGRPRMRNDYRDRYQYQGDLPPTKRMR

Sbjct 1 MGDSEDEYDKKRRDKFQGERSDSYRTDKRKDDDWGRPRMRNDYRDRYQYQGDLPPTKRMR 60

Query 61 YDSDDIRRMRYNDPGYGPYNSWGPEPPYPGNSRLGEMDTQPPIMTFKAFLQSQDDNITDE 120

YDSDDIRRMRYNDPGYGPYNSWGPEPPYPGNSRLGEMDTQPPIMTFKAFLQSQDDNITDE

Sbjct 61 YDSDDIRRMRYNDPGYGPYNSWGPEPPYPGNSRLGEMDTQPPIMTFKAFLQSQDDNITDE 120

Query 121 EAIAKYAEYKLEFRRQQLNEFFVAHKEEEWFKLKYHPEESLKRKEELNNALKKRCDVFLE 180

EAIAKYAEYKLEFRRQQLNEFFVAHKEEEWFKLKYHPEESLKRKEELN+ALKKRCDVFLE

Sbjct 121 EAIAKYAEYKLEFRRQQLNEFFVAHKEEEWFKLKYHPEESLKRKEELNSALKKRCDVFLE 180

Query 181 MLESKRMDDIRVDTEQGDEVVKLLDSVVIRLEGGTDLDLTILDQQEGQKDEQKEKDKSDD 240

MLES RMDDIRVDTEQGDEVVKLLDSVVIRLEGGTDLDLTILDQQEGQKDEQKEKDKSDD

Sbjct 181 MLESHRMDDIRVDTEQGDEVVKLLDSVVIRLEGGTDLDLTILDQQEGQKDEQKEKDKSDD 240

Query 241 KPKENNKSDEEEGKKKENEENKDEIVIDEDDERKEEEEKNEVHELSEDERPPGVDPESDI 300

KPKENNKSDEEE K+KENEENKDEIVIDEDDERKEEEEKNEVHELSEDERPPGVDPESDI

Sbjct 241 KPKENNKSDEEEVKQKENEENKDEIVIDEDDERKEEEEKNEVHELSEDERPPGVDPESDI 300

Query 301 EKEDTPVEKESQSEKEPQPEEEKRESSPEKEKQEEVKKSKDDQDSDKDHDEVVPLTPRAL 360

EKED P EKES SEKE QPEE +ESSPEKEK+EEVKKSKDDQDSDKDHDEVVPLTPRAL

Sbjct 301 EKEDVPAEKESPSEKETQPEE--KESSPEKEKKEEVKKSKDDQDSDKDHDEVVPLTPRAL 358

Query 361 HKTSSIFLRTISPRVTKAEIESICKKYPGFLRVSLSEPQAEQHWYRRGWVTFRRDVNIKE 420

HKTSSIFLRTISPRVTKAEIESICKKYPGFLRVSLSEPQAEQHWYRRGWVTFRRDVNIKE

Sbjct 359 HKTSSIFLRTISPRVTKAEIESICKKYPGFLRVSLSEPQAEQHWYRRGWVTFRRDVNIKE 418

Query 421 ICWNLNNIRVKECEMGAIVNRDLSRRVRSVSGLSGHKQVILADLKLAAKIIQELDSRAGF 480

ICWNLNNIRVKECEMGAIVNRDLSRRVRSVSGLSGHKQV+L DLKLAAKIIQELDSRAGF

Sbjct 419 ICWNLNNIRVKECEMGAIVNRDLSRRVRSVSGLSGHKQVVLGDLKLAAKIIQELDSRAGF 478

Query 481 CTPDQSEPFGLESRNAVFKGITEYLVEEAPAEEEELLGQGIVTDESKSRIAGAIKILDKL 540

C PDQSE FGLE+RNAVFKGITEYLVEEAPAEEEELLGQGIV DESKSRIAGAIKILDKL

Sbjct 479 CNPDQSETFGLETRNAVFKGITEYLVEEAPAEEEELLGQGIVADESKSRIAGAIKILDKL 538

Query 541 LLYLRIVHSVDYYNTSQYTSEDEMPNRCGIMHLRGLPSSTEVSPQEIQEYIEGYKTKLEP 600

LLYLRIVHSVDYYNTSQYTSEDEMPNRCGIMHLRGLPSSTEVSPQEIQEYI+GYKTKLEP

Sbjct 539 LLYLRIVHSVDYYNTSQYTSEDEMPNRCGIMHLRGLPSSTEVSPQEIQEYIDGYKTKLEP 598

Query 601 LYTPVPTVSEQELSSLGAKDRDTEVEKFIQANTQELAKDKWLCPLSGKKFKGPEFVRKHI 660

LYTPV TV+EQEL+SLGAKDRDTEVEKFIQANTQELAKDKWLCPLSGKKFKGPEFVRKHI

Sbjct 599 LYTPVQTVTEQELTSLGAKDRDTEVEKFIQANTQELAKDKWLCPLSGKKFKGPEFVRKHI 658

Query 661 YNKFSQELEEVKKEVDYFNNYLRDPKRPQLAEHPGNRGGKKEPESPYHYQYGGGFKRGFG 720

YNKFSQELEEVKKEVDYFNNYLRDPKRPQLAEHPGNRGGKKEPESPYHYQYGGGFKRGFG

Sbjct 659 YNKFSQELEEVKKEVDYFNNYLRDPKRPQLAEHPGNRGGKKEPESPYHYQYGGGFKRGFG 718

Query 721 HFGGGGGHGGFNRGRGGFGRGRGMDYRPIITYRDLDAPCEPDEII 765

HF GGHGGFNRGRGGFGRGRGMDYRPIITYRDLDAPCEPDEII

Sbjct 719 HF---GGHGGFNRGRGGFGRGRGMDYRPIITYRDLDAPCEPDEII 760

Nina C

>TRINITY_DN11848_c0_g1_i1 len=3502 path=[0:0-3328 2:3329-3501]

GCAATAGCACTGCTCTGCATAAATATCTGGAACAGGAAAAAAAAAATTAATCATGGAGCGCGGTCTAGGC
AACGAAGGCCTGGACTTCGATAAATTAGAAGATCCTGGAGAGAAATATGACCTTCAAGATATTTTAGGATC
AGGAGTAAATGCTAAAGTGTATGCAGCTACGGATAAGAATTCAGGACACAAGGTTGCTATAAAAGTTCAA
AAGGTTACAAATGAAAATAAATCGGCCGTTGAAGAAGAATACAAGATCCTTCGAGATCTTTCAAATCACCC
AAACCTTCCAGACTTCTATGGAGTATACAAGCGCTCTGAAGGAAATAAAAACTACGTTTGGTTTGTAATGG
AGCTTTGTGAAGGTGGCCCTGTCATCGATTTGATTCGCGCTCTTCACAGACAAGCTAAAAAAATGAACGAA
TTGCATATAGCTTTTATACTGAACGAGACAATTAAGGCTGTTCAAAAATTACACGAGAATCACGTAATTCAT
CGAGATATAAAAGGAAGCAACATACTTCTTACAAAAAATGGTGAAGTGAAGTTGGTGGATTTTGGAATATC
AAAGGAGCTATCAAGTACATTAGGAAGATGCTTGACATCGATTGGTTCTCCATCTTGGATGGCACCAGAGG
TCGTTGAATGTAAAGGCAACAAAACAGCTTATGACAACAGGGCTGATGTTTGGGCACTGGGAATTACAGCC
ATAGAGTTAGGAGATGGTAAAGCACCATTCCAAGACATGCATCCGACAAGTGCTCTCTTCCAAATCGTCCG
AAACCCACCTCCGACTCTCTATCGACCAGCAAACTGGTCACAAACTTACAATGACTTTATAGCCGAGTGCC
TCGAGAAAAATCCTGAACATAGGCCTTATTTAGTTGAGTTAATGGAGCACCCTTTCTTGACACAATTGCCAG
AAAATGATTTTCATCTAAATGCAGAACTCAAATCATTACTTGAAAATGTAACTACTGAAGATTTAGCCAAT
AGATCAGCAGAAATCAGTATTCGAAAAGGGTATTTAAAGAAAGGGCCAAGCCTAGATGAAGAACCAATGT
GTGTGGCAGATATGGCTGCTCTAGAAAAAATCACTGAAGATAACATTATTGAACAGCTTGAAGCAAGATAT

AGGAAGAATGATACATACACATTTATTGGAGATGTCTTATTATTTCTTAACCCAAATAAAACACTTGATATT
TATGGATATCAGTTTTACAGCAAATATAAATTCAAATCCAGATCTGATAATGAGCCCCACATATTTGCAATA
GCTGACAGTGCTTATCAAAATATGTTACACCACAATACCCCTCAGCATATTGTCATCTCAGGAGAAATCAT
GTCAGGAAAGACACAGCAGTATAAGCATGTCGTGAATCATCTACTCTTCCTTGGCTGGAATGGTAAGCAAA
TTAGTGATAAAATAAAAAAAGCTGTTGAAATAATCCAAGCATTCGGAAATGCTGCTACACCCCTTCATGAT
AATTCTACAAGACATGCCCTTTATACACAAGTAACATTTTCAAATTCAGGCAAAGTCTCGGGAGCTATATTT
TGGCTCTATCAGCTGGAAAAGTGGAGAGTAACAGGGAACAGGTCACCATATCATGCTAACTTTCACATATT
TTATTACTTATATGATGGCTTATCTTCAGAAGGAAATCTAGAAACATATATGCTAGAAAGAGAAAAATCCT
ACTGTTACTTTCGAAGAGAAATTTCAGAGGAAGATGTCGATAACAAAGCTCCAATGGGTCCTAGAGACAGA
CCTGAAATTAACGCGGCTTGCTTCAGGAAATTAAAAGAAAGTCTAACAGCACTCCAGTTTGAGGAATCAGA
ACAAGATCTCATTTGGAAGATTCTTGCAGCCATAATTTTATTAGGAGAGATTGACTATAAAGAAGATGAAG
ACGGCAATGCAGATGTTAAAGATGTGGATGTTGTTGATAAAGTGGCCACTTTGTTAGAAATTGATGGCAAA
AGATTGACTTGGGCACTTTGTAATTATTGTGTTATTGAAAAAGATACTGCAGCGAGAAGAAAGCATTCTATT
CCAGAGGCCATTGCAGCAAGAAATGTTCTAGCACAAACATTATATGCAAGAGTAGTTGATTGGGTTATTAA
TTTGATTAATTACAAGATGTCACTTTTAAGAGCTGTATATGGAGACAACCATTTCATTGGAATACTAGATAT
GTTTGGTTTTGAATGCTATGATGAAAATGGACTTGAGCAACTTTTTGTAAATTCACTGAATGAACAACTCCA
GTACTACTACAACCAAAAAATATTTATATCTGAAATCGAAGAAGAAGAGGAAGAAGAAATACAACTAAAA
AAGTTTCAATTTTACAATAACAGGGAAACAATGGATGAACTATTCAACAAACCTAATGGTTTAATGCATAT
TTTAGATGAAGCAAACAAGCTCAATATGGATTCAGAATACATAATAGATGCTCTGGACAAGAAAGGTGAA
GGATCTCGCATACTCTCATGTGGAGAGGAAGAATTTTTTGTTGCTCATTACACAGGAAAGGTGCGATATCA
AACGACTACCATGTGTACAAAAAATCGAGACTTCCTACCACCAGAGCTAACAGAAATTCTGAGATGTTCGG
CAAACAATAATATAAAACAACTCTTTACCAACAAATTAAATAGAACAGGGAATCTTACAATCAACACAGAT
AATATTATAACAGGTGCTGGTGTTGTTAAGAAGAAAAGTTGGGGATCAGCATTACTAGCTGATACAAACAG
AACACCAAGGCCTTATAATACAGCTTCAAAAGGAGAGTTTTCTCAAACGAGAGGTATAAGAACAGCCGCA
GCCATATTTAAATCAACATCTCTTGAAATATTGAAATCTTTAGCATCGGGTAGTTCCTATTTTGTTCGATGTA
TCCGAACAGATCTTCAAGGTACTCCAGGTGGTTTTCAGCAAGGTATCATCAGGCAACAGTTACGAGCGTTA
TCAGTAATTGATACAGCAAAAGCAAGGCAGCTGGGCTATTCTCACAGGATTACATTCGCACAATGTTTGGA
CCGATATCAGTTTTTGGCTTTTGACTTTGATGAAGAAGTTGAAAAAACAAGAGATAACTGCCGCCTGTTGAT
GATTCGCTTGAAGCTAGAAGGATGGTACTTAGGAAATTCTAAAGTTTTCCTCAAGTATTATAATGAAGAAT
ACTTATCAAGAATGTATGAAACTCAAGTGAAGAAGATCATAAAGGTCCAGTCGATGCTTCGATCTTTCTTA
GCAAAAAGAAATGTAGCCAATAAAAAGCTGAAATCAACACCTTCAATCACTGAGTCTGGTAATGACGACA
TCCAAGAGGAATAATTCAAAAATACTTTTATTGTTATTCAACAAAAATTTTAATCTATTAGTAGTCTGATTA
AAATATCCTGAAATTGTTTAAATAATCATTATGTAAAAAAAAAAAAAAAAAATGTTATTGTTCTAAAATATA
AGCAATACAATGCTATTG

Protein

RF: +2

ORF: 53 -> 3355

Length: 1100 aa

>lcl|ORF3_TRINITY_DN11848_c0_g1_i1:52:3354 unnamed protein product

MERGLGNEGLDFDKLEDPGEKYDLQDILGSGVNAKVYAATDKNSGHKVAIKVQKVTNENKSAVEEEYKILRDL
SNHPNLPDFYGVYKRSEGNKNYVWFVMELCEGGPVIDLIRALHRQAKKMNELHIAFILNETIKAVQKLHENHVI
HRDIKGSNILLTKNGEVKLVDFGISKELSSTLGRCLTSIGSPSWMAPEVVECKGNKTAYDNRADVWALGITAIEL
GDGKAPFQDMHPTSALFQIVRNPPPTLYRPANWSQTYNDFIAECLEKNPEHRPYLVELMEHPFLTQLPENDFHL
NAELKSLLENVTTEDLANRSAEISIRKGYLKKGPSLDEEPMCVADMAALEKITEDNIIEQLEARYRKNDTYTFIG
DVLLFLNPNKTLDIYGYQFYSKYKFKSRSDNEPHIFAIADSAYQNMLHHNTPQHIVISGEIMSGKTQQYKHVVNH

LLFLGWNGKQISDKIKKAVEIIQAFGNAATPLHDNSTRHALYTQVTFSNSGKVSGAIFWLYQLEKWRVTGNRSP
YHANFHIFYYLYDGLSSEGNLETYMLEREKSYCYFRREISEEDVDNKAPMGPRDRPEINAACFRKLKESLTALQF
EESEQDLIWKILAAIILLGEIDYKEDEDGNADVKDVDVVDKVATLLEIDGKRLTWALCNYCVIEKDTAARRKHSI
PEAIAARNVLAQTLYARVVDWVINLINYKMSLLRAVYGDNHFIGILDMFGFECYDENGLEQLFVNSLNEQLQY
YYNQKIFISEIEEEEEEEIQLKKFQFYNNRETMDELFNKPNGLMHILDEANKLNMDSEYIIDALDKKGEGSRILSC
GEEEFFVAHYTGKVRYQTTTMCTKNRDFLPPELTEILRCSANNNIKQLFTNKLNRTGNLTINTDNIITGAGVVKK
KSWGSALLADTNRTPRPYNTASKGEFSQTRGIRTAAAIFKSTSLEILKSLASGSSYFVRCIRTDLQGTPGGFQQGII
RQQLRALSVIDTAKARQLGYSHRITFAQCLDRYQFLAFDFDEEVEKTRDNCRLLMIRLKLEGWYLGNSKVFLKY
YNEEYLSRMYETQVKKIIKVQSMLRSFLAKRNVANKKLKSTPSITESGNDDIQEE

Conserved Domains

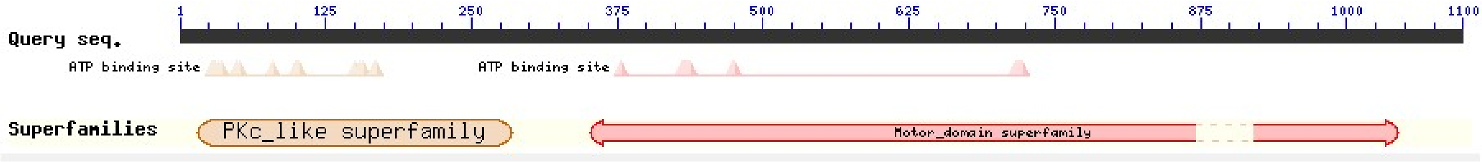

BLASTp

XP_014281724.1 neither inactivation nor afterpotential protein C [*Halyomorpha halys*]

Score:1097 bits

E-value: 0.0

Query 1 MERGLGNEGLDFDKLEDPGEKYDLQDILGSGVNAKVYAATDKNSGHKVAIKVQKVTNENK 60

MERGLGNEGLD DKLEDPGEKY+LQ+ILG+GVNAKVYAATDKNSGHKVAIKVQKVTNENK

Sbjct 1 MERGLGNEGLDIDKLEDPGEKYELQEILGTGVNAKVYAATDKNSGHKVAIKVQKVTNENK 60

Query 61 SAVEEEYKILRDLSNHPNLPDFYGVYKRSEGNKNYVWFVMELCEGGPVIDLIRALHRQAK 120

S+VEEEYKILRDLSNHPNLPDFYGVYK SEGNKNYVWFVMELC+GGP+IDLIRALHRQAK

Sbjct 61 SSVEEEYKILRDLSNHPNLPDFYGVYKHSEGNKNYVWFVMELCDGGPIIDLIRALHRQAK 120

Query 121 KMNELHIAFILNETIKAVQKLHENHVIHRDIKGSNILLTKNGEVKLVDFGISKELSSTLG 180

KMNELHIAFIL ETIKA+QKLHENHVIHRDIKGSNILLTKNGEVKLVDFGISKELSSTLG

Sbjct 121 KMNELHIAFILKETIKAIQKLHENHVIHRDIKGSNILLTKNGEVKLVDFGISKELSSTLG 180

Query 181 RCLTSIGSPSWMAPEVVECKGNKTAYDNRADVWALGITAIELGDGKAPFQDMHPTSALFQ 240

RCLTSIGSPSWMAPEVVECKGNKTAYDNRADVWALGITAIELGDGKAPFQDMHPTSALFQ

Sbjct 181 RCLTSIGSPSWMAPEVVECKGNKTAYDNRADVWALGITAIELGDGKAPFQDMHPTSALFQ 240

Query 241 IVRNPPPTLYRPANWSQTYNDFIAECLEKNPEHRPYLVELMEHPFLTQLPENDFHLNAEL 300

IVRNPPPTLYRPANWSQ YNDFIAECLEKNPEHRPYL+E+MEHPF+TQLPENDFHLNAEL

Sbjct 241 IVRNPPPTLYRPANWSQNYNDFIAECLEKNPEHRPYLMEIMEHPFITQLPENDFHLNAEL 300

Query 301 KSLLENVTTEDLANRSAEISIRKGYLKKGPSLDEEPMCVADMAALEKITEDNIIEQLEAR 360

KSLLENVT++DL NRSAEISIRKGYLKKGPSLDEEPMCVADMAALEKITEDNIIEQLEAR

Sbjct 301 KSLLENVTSDDLTNRSAEISIRKGYLKKGPSLDEEPMCVADMAALEKITEDNIIEQLEAR 360

Query 361 YRKNDTYTFIGDVLLFLNPNKTLDIYGYQFYSKYKFKSRSDNEPHIFAIADSAYQNMLHH 420

YRKNDTYTFIGDVLLFLNPNK+LDIYGYQFYSKYKFKSRSDNEPH+FAIADSAYQNMLHH

Sbjct 361 YRKNDTYTFIGDVLLFLNPNKSLDIYGYQFYSKYKFKSRSDNEPHVFAIADSAYQNMLHH 420

Query 421 NTPQHIVISGEIMSGKTQQYKHVVNHLLFLGWNGKQISDKIKKAVEIIQAFGNAATPLHD 480

NTPQHIVISGEIMSGKTQQ+KH+VNHLLFLGWNGKQISDKIKK+VEIIQAFGNAATPLHD

Sbjct 421 NTPQHIVISGEIMSGKTQQFKHIVNHLLFLGWNGKQISDKIKKSVEIIQAFGNAATPLHD 480

Query 481 NSTRHALYTQVTFSNSGKVSGAIFWLYQLEKWRVTGNRSPYHANFHIFYYLYDGLSSEGN 540

NSTRHALYTQ+TFSNSGK+SGAIFWLYQLEKWRVTGNRSPYHANFHIFYYLYDGLSSEGN

Sbjct 481 NSTRHALYTQITFSNSGKISGAIFWLYQLEKWRVTGNRSPYHANFHIFYYLYDGLSSEGN 540

Query 541 LETYMLEREKSYCYFRREISEEDVDNKAPMGPRDRPEINAACFRKLKESLTALQFEESEQ 600

LE YMLEREKSY YFRR+ISEEDVD KAP+GPRDRPE NAACFRKLKESLTALQFEES+Q

Sbjct 541 LEKYMLEREKSYYYFRRQISEEDVDQKAPLGPRDRPETNAACFRKLKESLTALQFEESDQ 600

Query 601 DLIWKILAAIILLGEIDYKEDEDGNADVKDVDVVDKVATLLEIDGKRLTWALCNYCVIEK 660

DLIWKILAAIILLGEI+YKEDEDGNAD+KD D+VDKVATLLEIDGKRLTWALCNYCVIEK

Sbjct 601 DLIWKILAAIILLGEIEYKEDEDGNADLKDTDIVDKVATLLEIDGKRLTWALCNYCVIEK 660

Query 661 DTAARRKHSIPEAIAARNVLAQTLYARVVDWVINLINYKMSLLRAVYGDNHFIGILDMFG 720

DTAARRKHSIPEAIAARNVLAQTLYARVVDW+INLINYKMSLLRAVYGD+HFIGILDMFG

Sbjct 661 DTAARRKHSIPEAIAARNVLAQTLYARVVDWIINLINYKMSLLRAVYGDHHFIGILDMFG 720

Query 721 FECYDENGLEQLFVNSLNEQLQYYYNQKIFISEIEEEEEEEIQLKKFQFYNNRETMDELF 780

FECYDENGLEQLFVN+LNEQLQYYYNQKIFISEIEEEEEEEIQLKKFQFYNNR+TMDELF

Sbjct 721 FECYDENGLEQLFVNTLNEQLQYYYNQKIFISEIEEEEEEEIQLKKFQFYNNRDTMDELF 780

Query 781 NKPNGLMHILDEANKLNMDSEYIIDALDKKGEGSRILSCGEEEFFVAHYTGKVRYQTTTM 840

NKPNGLMHILDEANKLN+DSE+IIDALDKK EGSRILSCGEEEFFVAHYTGKVRYQTT M

Sbjct 781 NKPNGLMHILDEANKLNVDSEHIIDALDKKSEGSRILSCGEEEFFVAHYTGKVRYQTTAM 840

Query 841 CTKNRDFLPPELTEILRCSANNNIKQLFTNKLNRTGNLTINTDNIITGAGVVKKKSWGSA 900

CTKNRDFLPPELTEILRCSANNNIKQLFTNKLNRTGNLTINTD +IT GV+KKKSWGSA

Sbjct 841 CTKNRDFLPPELTEILRCSANNNIKQLFTNKLNRTGNLTINTDTVITSTGVIKKKSWGSA 900

Query 901 LLADTNRTPRPYNTASKGEFSQTRGIRTAAAIFKSTSLEILKSLASGSSYFVRCIRTDLQ 960

LLAD NRT RPYNTASKGEFSQTRGIRTAAAIFKSTSLEILKSLASGSSYFVRCIRTDLQ

Sbjct 901 LLADQNRTARPYNTASKGEFSQTRGIRTAAAIFKSTSLEILKSLASGSSYFVRCIRTDLQ 960

Query 961 GTPGGFQQGIIRQQLRALSVIDTAKARQLGYSHRITFAQCLDRYQFLAFDFDEEVEKTRD 1020

TPGGFQQGIIRQQLRALSVIDTAKARQLGYSHRITFAQCLDRYQFLAFDFDEEVEKTRD

Sbjct 961 STPGGFQQGIIRQQLRALSVIDTAKARQLGYSHRITFAQCLDRYQFLAFDFDEEVEKTRD 1020

Query 1021 NCRLLMIRLKLEGWYLGNSKVFLKYYNEEYLSRMYETQVKKIIKVQSMLRSFLAKRNVAN 1080

NCRLLMIRLKLEGWYLGNSKVFLKYYNEEYLSRMYETQVKKIIKVQSMLRSFLAKRNVAN

Sbjct 1021 NCRLLMIRLKLEGWYLGNSKVFLKYYNEEYLSRMYETQVKKIIKVQSMLRSFLAKRNVAN 1080

Query 1081 KKLKSTPSITESGNDDI 1097

KKLKSTPSITES N+ I

Sbjct 1081 KKLKSTPSITESANELI 1097

Beta 1,4-mannosyltransferase (egh)

>TRINITY_DN10121_c0_g1_i1 len=2786 path=[0:0-2785]

CATGGGTGTTGCCATTTTGCTGTAGTAGATTTTGAATTGTCTCGGTTGTTGAATTACAATGAATTACTTATAA
TGATTGATTAAATTCATTACGGATTATGTTATTTTAAAATTCGTTTTCACCAGTTATATTCTAAGAAATTGTA
ATGCACTTGACCATAGGAAGATAACATAAAAGTATCAGATATGTTAAACAGCAAAACTAAGCATGCTCTCC
ACTGTGCACTATTGTTTTCGGTTATCATTATTTTTGAACTCTTAACTGGAGGACTGAAATTATCAAGTGATG
AAACAATTATAGATCCATGGGCAAAATATGGATTCATTTTTACAATAGTCCTGTATATTCTGAGATTTCTGG
CATTCTTACCATTGCCACAAATTCTTCTGAATTTTGCAGGCCTCACATTTTACAATGCCTTTCCTGACAATGT
TATTTTAAAAGGTAGTCCAATTTTAGCACCATTTATATGTATAAGGATAGTTACTCGTGGCGATTATCCACA
GTTAGTGAAGAACAATGTAAGCCGTAATTTATCCAAGTGTTTGAGTGCCGGTTTAGAACATTTTGTAATTGA
AGTTGTGACTGATAAACCTATACATCTCTTGAAAGACAGAAGAGTTATAGAAACTGTTGTACCAGGAAATT
ATAAACCTAAGTCTGGTGCTCTATTTAAAGCTCGTGCCCTACAGTATTGTTTAGAAGAAGGTGTTAATACTT
TATCCGATTCTGATTGGATTGTGCATCTTGATGAAGAAACTCTCTTGACTGAAAATTCAATTAGAGGTATAT
TGAATTTTGTAATAGATGGAAAACACTCTTTTGGTCAAGGGCTGATAACGTATGCCAATGAAGAAGTTGTC
AACTGGATAACCACACTTGCTGATACATTTAGGGTTGCAGATGATATGGGGAAGCTTCGTTTTCAATTTTTA
ATGTTTCATAAACCCCTTCTAAGTTGGAAAGGCTCTTACGTTGTCACTCAGGTGAAGGCAGAACGTGATGTT
ACATTTGATAATGGATTGGATGGTTCAGTTGCTGAAGACTGTTTTTTTGCTATGAAAGCATATAAAATGGGT
TATACATTCAATTTTATTGAAGGAGAAATGTGGGAAAAATCACCTTTCACTCTTGGTGATTTCTTGCAACAA
AGGAAACGTTGGCTCCAAGGCATTCTTTTGGTCGTACGTTCGAAAAAAATTCCTTTGAAAAATAAAATATTT
CTCAGTATTTCATGTTATTCTTGGGTCACAATGCCTCTTTCAACATCAAATATTTTATTAGCTTCACTCTGTC
CAATTCCTTGTCCTCCATTAATAGATTTCTTATGTGCTTTTATTGGTGCTGTTAGCATATATATGTATATTTTT
GGGGTCATTAAATCATTTTCATTATATCGTTTTGGTATCATCAAATTTAGCCTTTGTATTTTTGGTGCACTAG
CTACGATTCCAGTCAATGTTATTATTGAAAATGTTGCTGTAATTTGGGGTTTATTAGGAAAAAAACATAAAT
TTTATGTTGTAAACAAAGATAATCGACCACCAGTGACTGTTTAAATATTACATGAAGAAGTTATTGTCTAGT
TAGAATAATAATAATTGATATTAATTTCAAATGAGTTGTACATAACAAACAATTTTTGTTCTTATAATTTTGT
AGGATTGTAGGGTCCCTGATAGTAATGACAGTCTTCATCCATACTATTGCATTGTATACATTGTAAATTTAT
TCAGATGGTAAATATTTTTAGCAATTAATTAAGTTATTGCTGTCAAGATTATGTTTTCATAATTATTTTAAAA
TAATATTTTTTTTTGGGGTATCTAAGCATAAATTGTATGAAATCATTGTGATTTAAAATTACACTAAAATAT
AAGGACATCTGATTTATAATTTATTCACTTTTTTCTTGTATTCTTGTTCCAGCAACAGATGACTTTGATTGTT
AGTTAACTGTTATGATTTCAATTATTTATTTGTTTATGTTAGCTGTTCCTTAGTCAGTTATTTTAAGTTTTACT
TGTCCATAATATAGGAGAACATATTAGATAAAGTTGGCTGTGTAATGTCCATTTATTATATAGTTGAACCTC
TTAGTTGTGTTTTAAAGTTGTATTTATAATTCTTAACATCACCGAAAATGAGCTCTTCTACTTAATTAGTTTT
ATAGCACAAGAAGTGTAAATGATATAGTAAGACTGAACAGTTTATATTTTATTTTCCTTATACTATAATATT
TTTGTCACATAATCATGTAGAAATCTTAGTTGAAATTAGTTCTATTTGTATCTTTTTATTGTGTATAATTCAA
TATTTCCTTAAACAAATACCCTTCCACTAGGTTGTTGTTTGTTGTAAACCGATTCCTTTATTGTCACTACTAG
TCACATACCTATTTACATATATACATTTTAAAATGTTCTATCAAATATCAAATGCAGAAAAATGTGATTACC
GATTAATTATGTTATAATCTCTATGTCTTTACGTTATTTTTCTTTCATTATGTCTTTTCCCTGATCATTATTAT
AATGCATATCTTATATTTCATGTTCCAATTTTATGTATGTATATTATTATTGAGTGTATGTCCCTATTTCCAAT
TTATATTTATTGATTTCTCCAAGTTGGTTAGTTATTAATTTTTGTGTAAGATACATAACATTAATCAGCAATT
ACCTTAATTATCTTATACAGAAACAGTAACTGCATGCATATCATTCATTGGAATTAATCAATTTTTAAAAAT
AGATATTTGGTTGCAATCTTTGATTAATTTGGT

Protein

RF: +1

ORF: 187 -> 1560

Length: 601 aa

>lcl|ORF1_TRINITY_DN10121_c0_g1_i1:186:1559 unnamed protein product

MLNSKTKHALHCALLFSVIIIFELLTGGLKLSSDETIIDPWAKYGFIFTIVLYILRFLAFLPLPQILLNFAGLTFYNAF
PDNVILKGSPILAPFICIRIVTRGDYPQLVKNNVSRNLSKCLSAGLEHFVIEVVTDKPIHLLKDRRVIETVVPGNYK
PKSGALFKARALQYCLEEGVNTLSDSDWIVHLDEETLLTENSIRGILNFVIDGKHSFGQGLITYANEEVVNWITTL
ADTFRVADDMGKLRFQFLMFHKPLLSWKGSYVVTQVKAERDVTFDNGLDGSVAEDCFFAMKAYKMGYTFNF
IEGEMWEKSPFTLGDFLQQRKRWLQGILLVVRSKKIPLKNKIFLSISCYSWVTMPLSTSNILLASLCPIPCPPLIDFL
CAFIGAVSIYMYIFGVIKSFSLYRFGIIKFSLCIFGALATIPVNVIIENVAVIWGLLGKKHKFYVVNKDNRPPVTV

Conserved Domains

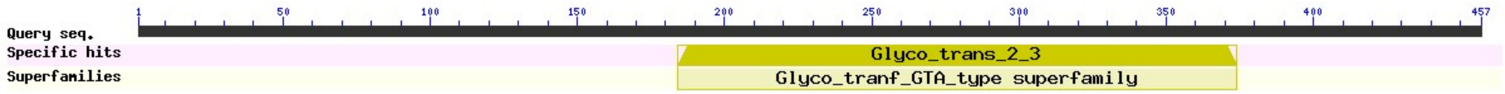

BLASTp

XP_014283435.1 beta-1,4-mannosyltransferase egh isoform X1 [*Halyomorpha halys*]

Score:918 bits

E-value: 0.0

Query 1 MLNSKTKHALHCALLFSVIIIFELLTGGLKLSSDETIIDPWAKYGFIFTIVLYILRFLAF 60

MLNSKTKHALHCALLFSVIIIFELLTGGLKLSSDET+IDPW KYGF+ TIVLY LRFLAF

Sbjct 1 MLNSKTKHALHCALLFSVIIIFELLTGGLKLSSDETVIDPWVKYGFVITIVLYALRFLAF 60

Query 61 LPLPQILLNFAGLTFYNAFPDNVILKGSPILAPFICIRIVTRGDYPQLVKNNVSRNLSKC 120

LPLPQILLNFAGLTFYNAFPDNVILKGSP+LAPFIC+RIVTRGDYPQLVKNNVSRNLSKC

Sbjct 61 LPLPQILLNFAGLTFYNAFPDNVILKGSPLLAPFICVRIVTRGDYPQLVKNNVSRNLSKC 120

Query 121 LSAGLEHFVIEVVTDKPIHLLKDRRVIETVVPGNYKPKSGALFKARALQYCLEEGVNTLS 180

LSAGLEHFVIEVVTDKP+HL+KDRRVIETVVPGNYK KSGALFKARALQYCLEEGVNTLS

Sbjct 121 LSAGLEHFVIEVVTDKPLHLMKDRRVIETVVPGNYKAKSGALFKARALQYCLEEGVNTLS 180

Query 181 DSDWIVHLDEETLLTENSIRGILNFVIDGKHSFGQGLITYANEEVVNWITTLADTFRVAD 240

DSDWIVHLDEETLLTENSIRGILNFVIDGKHSFGQGLITYANEEVVNWITTLADTFRVAD

Sbjct 181 DSDWIVHLDEETLLTENSIRGILNFVIDGKHSFGQGLITYANEEVVNWITTLADTFRVAD 240

Query 241 DMGKLRFQFLMFHKPLLSWKGSYVVTQVKAERDVTFDNGLDGSVAEDCFFAMKAYKMGYT 300

DMGKLRFQFLMFHKPLLSWKGSYVVTQVKAERDVTFDNGLDGSVAEDCFFAMKAYKMGYT

Sbjct 241 DMGKLRFQFLMFHKPLLSWKGSYVVTQVKAERDVTFDNGLDGSVAEDCFFAMKAYKMGYT 300

Query 301 FNFIEGEMWEKSPFTLGDFLQQRKRWLQGILLVVRSKKIPLKNKIFLSISCYSWVTMPLS 360

FNFIEGEMWEKSPFTLGDFLQQRKRWLQGILLVVRSKKIPLKNKIFLSISCYSWVTMPLS

Sbjct 301 FNFIEGEMWEKSPFTLGDFLQQRKRWLQGILLVVRSKKIPLKNKIFLSISCYSWVTMPLS 360

Query 361 TSNILLASLCPIPCPPLIDFLCAFIGAVSIYMYIFGVIKSFSLYRFGIIKFSLCIFGALA 420

TSNILLASLCPIPCPPLIDFLCAFIGAVSIYMYIFGVIKSFSLYRFG+IKFSLCIFGALA

Sbjct 361 TSNILLASLCPIPCPPLIDFLCAFIGAVSIYMYIFGVIKSFSLYRFGVIKFSLCIFGALA 420

Query 421 TIPVNVIIENVAVIWGLLGKKHKFYVVNKDNRPPVTV 457

TIP+NVIIEN AVIWGLLGKKHKFYVVNKDNRPP+TV

Sbjct 421 TIPINVIIENAAVIWGLLGKKHKFYVVNKDNRPPLTV 457

CG4572

>TRINITY_DN91529_c0_g1_i1 len=1547 path=[0:0-1546]

ATTCAGTATCAATTTTGTTACCAGAGAAGGCGGGCAGTTATATAAGATGAAATCTTATTTAGTGTTACTATG
GGTGTACTATTGTGTTAGTTTTACAAGTGGCTTTTGGATGGGAAAGTTTAGCAATGTTCCACACATTGCTGG
TGGTAACGTTGGAGAACCGTTATTTTTAACACCATTAATAGAAAATGGGTCTATTGCAGTAGCACAATCGG
CTGCATCTGTCAAGCCAGTAAAAGCTAATATTAAAAGTTATGCAGGTTTCTTGACAGTCAATAAGCAATAC
AATTCCAATATGTTTTTTTGGTATTTTCCAGCGGAAAACAACTCAACAACTGCACCTGTTGTACTTTGGCTG
CAAGGGGGGCCTGGTGCGTCATCCCTATATGGTTTATTTAATGAAAATGGGCCATTTTACGTCAAGAAGGA
AAGGGGTCTTAAGTCTAGAAAATATTATTGGTCGCAGATACTGAATGTTATTTACATTGATAACCCAGTTGG
TACAGGGTTTAGTTTTACAGAAAATGATAACGGTTATGTCAAAAATGAAGATGGTGTAGGAAACGACCTTT
ACTCGGCACTAATACAATTCTTTAAATTATTTCCAGACCTTCAAAAAAATGATTTCTTTGTCGCTGGAGAAT
CATATGCGGGAAAATACGTACCTGCAATTGCTTATAAAATTCACGTTAACAATGAGAAAAAATTACCAAAA
ATTAACTTAAAGGGAATTTCCATTGGCAACGGATTATCAGACCCGGAAAATATGATGAATTATGGAGACTA
CTTATACCAAATTGGATTGATTGATAGTAATACAAGAATGGTGTTTCAAAGGAAGCAAGATGACATAATTA
AAAATATTCAAGCAAAGAATTATTTAAAAGCATTTGAGGGGTTCGATGCTTTACTCAATGGTGATTTAACTC
CTTATAAAAGTTTTTTTTATAATCAGACTGGATTCAACTTTTACTTTAATTATTTGCACAGTGAAGACGATAG
TCCTTATGGAGATATGGCAGCATATGTTCAAAAAGATATAATGAGAAGAAGTATACATGTTGGTAATTTAA
CTTTCCATACAGACAGTAAAGTAGAGCAATATTTAAAGCAGGATGTAATGCAGTCAGTTAAACCATTGGTT
GAAAAACTAGTTGAGAAATATAAAGTATTGTTTTATAATGGCCAGTTGGACATAATTGTTCCGTATCCCTTA
ACAATAAACTTTTTACAGCGATTGAAATGGAGTGGGGCAAATATATATAAAACCGTTCCCAGAAAAAAATG
GATGATTGGTAATGAGCTTGCTGGTTACTCAAAATCTGTTAAAGGATTTACTGAAGTACTGGTTCGAAATGC
AGGGCATATGGTGCCTGGAGACCAGCCGAAATGGGCACTTGATTTAATTTCACGTTTTGTGTTCAATAAAC
CATTTTGAATCTTTGAAAATGAAATAACATATTTTACCATTTAGTTTATGGCAATGTATTTTATCATAAAATA
AATAATTTTGATTGAAAAAAAAAAAATATCGGAATAGCGTCGTG

Protein

RF: +2

ORF: 47 -> 1438

Length: 463 aa

>lcl|ORF1_TRINITY_DN91529_c0_g1_i1:46:1437 unnamed protein product

MKSYLVLLWVYYCVSFTSGFWMGKFSNVPHIAGGNVGEPLFLTPLIENGSIAVAQSAASVKPVKANIKSYAGFL
TVNKQYNSNMFFWYFPAENNSTTAPVVLWLQGGPGASSLYGLFNENGPFYVKKERGLKSRKYYWSQILNVIYI
DNPVGTGFSFTENDNGYVKNEDGVGNDLYSALIQFFKLFPDLQKNDFFVAGESYAGKYVPAIAYKIHVNNEKK
LPKINLKGISIGNGLSDPENMMNYGDYLYQIGLIDSNTRMVFQRKQDDIIKNIQAKNYLKAFEGFDALLNGDLTP
YKSFFYNQTGFNFYFNYLHSEDDSPYGDMAAYVQKDIMRRSIHVGNLTFHTDSKVEQYLKQDVMQSVKPLVE
KLVEKYKVLFYNGQLDIIVPYPLTINFLQRLKWSGANIYKTVPRKKWMIGNELAGYSKSVKGFTEVLVRNAGH
MVPGDQPKWALDLISRFVFNKPF

Conserved Domains

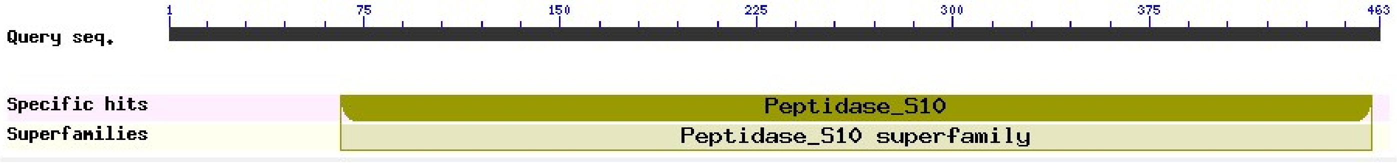

BLASTp

XP_014280828.1 venom serine carboxypeptidase-like [*Halyomorpha halys*]

Score:870 bits

E-value: 0.0

Query 4 YLVLLWVYYCVSFTSGFWMGKFSNVPHIAGGNVGEPLFLTPLIENGSIAVAQSAASVKPV 63

YLVL+ VYYCV+FTSGF M K+ N P+I G NVGEPLFLTP IENGSIA QSAASVKPV

Sbjct 14 YLVLICVYYCVNFTSGFGM-KYRNFPYITGDNVGEPLFLTPFIENGSIAEGQSAASVKPV 72

Query 64 KANIKSYAGFLTVNKQYNSNMFFWYFPAENNSTTAPVVLWLQGGPGASSLYGLFNENGPF 123

KAN+KSYAGF TVNKQYNSNMFFWYFPAENNSTTAPVVLWLQGGPGASSLYGLFNENGPF

Sbjct 73 KANVKSYAGFFTVNKQYNSNMFFWYFPAENNSTTAPVVLWLQGGPGASSLYGLFNENGPF 132

Query 124 YVKKERGLKSRKYYWSQILNVIYIDNPVGTGFSFTENDNGYVKNEDGVGNDLYSALIQFF 183

YVKKERGLKSRKYYWSQILNVIYIDNPVGTGFSFT+NDNGYVKNEDGVGNDLYSAL QFF

Sbjct 133 YVKKERGLKSRKYYWSQILNVIYIDNPVGTGFSFTDNDNGYVKNEDGVGNDLYSALTQFF 192

Query 184 KLFPDLQKNDFFVAGESYAGKYVPAIAYKIHVNNEKKLPKINLKGISIGNGLSDPENMMN 243

KLFP+L+KNDFFVAGESYAGKYVPAIAYKIH +NE+ LPKINLKGISIGNGLSDPENM+N

Sbjct 193 KLFPELRKNDFFVAGESYAGKYVPAIAYKIHTSNEQNLPKINLKGISIGNGLSDPENMLN 252

Query 244 YGDYLYQIGLIDSNTRMVFQRKQDDIIKNIQAKNYLKAFEGFDALLNGDLTPYKSFFYNQ 303

YGDYLYQIGLIDS+TRM FQRKQDDI+KNIQAKNYLKAFEGFDALLNGDLTPYKSFFYNQ

Sbjct 253 YGDYLYQIGLIDSSTRMAFQRKQDDIVKNIQAKNYLKAFEGFDALLNGDLTPYKSFFYNQ 312

Query 304 TGFNFYFNYLHSEDDSPYGDMAAYVQKDIMRRSIHVGNLTFHTDSKVEQYLKQDVMQSVK 363

TGF+FYFNYLH+EDDSPYGDM YVQKD+MRRSIHVGNLTFHTDSKVEQYLKQDVMQSVK

Sbjct 313 TGFSFYFNYLHNEDDSPYGDMGEYVQKDVMRRSIHVGNLTFHTDSKVEQYLKQDVMQSVK 372

Query 364 PLVEKLVEKYKVLFYNGQLDIIVPYPLTINFLQRLKWSGANIYKTVPRKKWMIGNELAGY 423

P +EKLVEKYKVLFYNGQLDIIVPYPLTINFLQRLKWSGANIYKTVPRKKWM+GNELAGY

Sbjct 373 PWIEKLVEKYKVLFYNGQLDIIVPYPLTINFLQRLKWSGANIYKTVPRKKWMVGNELAGY 432

Query 424 SKSVKGFTEVLVRNAGHMVPGDQPKWALDLISRFVFNKPF 463

SK+VKGFTEVLVRNAGHMVPGDQPKWALDLI+RFV+NKPF

Sbjct 433 SKTVKGFTEVLVRNAGHMVPGDQPKWALDLITRFVYNKPF 472

Intracellular transport genes

Vtype_proton_ATPase_catalytic_subunit

>TRINITY_DN3993_c2_g1_i1 len=3048 path=[0:0-3047]

GAGAAATCTTAGAGTTATATTTATAATATCATGGTCCAAGGTTTCTACTGATATAAGTTGCAGCTATAGATA
CAGCTGTTAGTTCTTGTCTCAGCTGTAGGTGGGTGAGGTTGAAGTGGTATACATTTACGTGACTAGGAGGA
AATTTTTTGCAAAATGGCCTTGCCCAGGATAAAAGACGAAGATCAGGAGTCGAAGTTTGGATATGTCTTTG
GTGTTTCTGGTCCTGTGGTCACTGCTGAAAAGATGTCAGGATCAGCTATGTACGAGCTAGTAAGAGTCGGTT
ATTTCGAATTGGTCGGTGAAATTATCCGTCTTGAAGGTGACATGGCTACAATTCAGGTATATGAAGAAACTT
CTGGCGTAACAGTTGGCGATCCTGTCTTACGAACAGGTAAACCTCTCTCTGTGGAATTGGGTCCTGGTATCC
TCGGAAGTATCTTTGATGGTATCCAGCGACCCCTGAAGGATATTAACGAAATCTCTAACAGTATTTACATCC
CGAAGGGTGTCAACATCCCTGCGTTGTCGAGAACTGCTGCTTGGGAGTTTCAGCCCACTAACATCAAGGTC

GGAAGCCACATTACCGGAGGTGATCTTTATGGAGTTGTCCATGAGAACACCCTCGTCAAACACAAAATGAT
CCTGCCGCCGAGAGCTAAGGGCACAGTGACCTATCTCGCTGCCCCCGGTAACTACACTGTCGATGATGTCG
TCCTTGAAACAGAGTTTGATGGTGAGAAAACTAAGTTCACTATGTTGCAAGTGTGGCCTGTGCGTCAGCCA
CGACCAGTCACTGAGAAACTCCCAGCCAACTACCCGTTGTTGACCGGCCAGCGTGTATTGGATGCACTCTT
CCCTTGTGTCCAAGGAGGTACCACTGCTATCCCAGGTGCTTTCGGTTGTGGTAAAACTGTCATCTCACAAGC
TCTGTCAAAGTATTCAAACTCAGATGTCATCATTTACGTAGGTTGCGGAGAGAGAGGAAATGAAATGTCTG
AGGTATTGAGAGATTTCCCAGAATTATCAGTTGAGATTGATGGTGTGACAGAATCCATCATGAAGAGAACA
GCGCTGGTCGCCAACACCTCTAACATGCCTGTAGCCGCTCGAGAGGCCTCCATCTATACTGGTATCACTCTG
TCTGAATACTTCAGAGACATGGGTTACAACGTTTCCATGATGGCTGACTCTACTTCAAGATGGGCCGAGGC
CTTGAGAGAAATTTCTGGTCGTTTGGCTGAAATGCCTGCCGACAGTGGTTACCCCGCCTACTTGGGAGCCCG
ATTGGCCTCTTTCTACGAGCGTGCCGGACGAGTGAAGTGCCTTGGCAATCCAGAGAGAGAAGGATCTGTAT
CCATTGTCGGAGCTGTGTCTCCTCCTGGTGGTGACTTCTCAGACCCTGTCACCTCTGCCACCCTCGGTATTGT
CCAAGTCTTCTGGGGTTTGGACAAGAAGCTCGCCCAGAGGAAGCATTTCCCGTCCATCAACTGGCTTATCTC
CTACAGTAAATACATGAGAGCGTTGGACGACTTTTATGACAAGAACTTCCCCGAATTTGTTCCCCTGAGAA
CCAAAGTGAAGGAGATTCTTCAGGAAGAGGAAGATCTTTCAGAAATTGTGCAGCTGGTCGGTAAGGCTTCC
CTCGCCGAGTCTGATAAGATCACCCTCGAGATTGCCAAGCTACTCAAGGACGACTTCCTCCAACAGAACAG
CTACTCACCTTACGATCGGTTCTGCCCATTCTACAAGACGGTCGGTATGCTGAAGAACATGATTTCCTTCTA
CGACTTGGCACGACACGCAGTCGAATCCACGGCCCAGAGCGAGAAGAAGATAACTTGGGCCGTCATCAAA
GAGAGCATGGGTAACATCCTTTACCAGATGTCGTCTATGAAGTTCAAGGACCCCGTGAAAGACGGTGAATC
CAAGATCAGGTCAGACTTCGAGCAGCTCCAAGAGGACATACAGCAGGCGTTCAGGAACCTCGAGGACTAA
CAACTGTACTGTCGTCGTACTGTTGTTTATTCTTCTCCACTTGTATTTTATCTCTCCCTACTGTTTATATATAC
ATCGGTGTTCTCACCTCTGCGGAATGTCATTATTTCTTCATTTTCTATGTATATAATATATGTTTACATAATA
TAATAACAACAAAATGTATAATTATAAATTTGAAAATGTGTATGATAATTCTTTAAGCAATGACATTAAAT
GAAAAACAAAAAAAGTATTAAAAAAAAAAAAAAAATTGAAACAGCTGATGAAATAGATTTAGATAACCACA
TTTAAAGTTAAGATGGGGTCTTCGGGAACTAGTTATTCGGTCATTAGATGTTTTAATGATATTTTCAGATTTT
ATTTAAGATATTTATTAGGTGAGTAATTTCTTAGGACCTAGGTAGCTAAAATGACACTTCGGAGTGAAAAC
AGCAGTACTGGGGAGTCATATCAGTCCCGAGAATTTTAAATGTAATTTACTTGTAATTGTTTAATTTTTTTTT
TTGTTATTGATTGTTGTAAAAGTTTTTTTTTCTTTTGTTATATCTCTTCTGAATCATTCCGTTTGTTGTATGTA
AACAAAAAAAAAAAAAAGTTTGAGATAAAATGTAAAAAGTATAAAAATATATATCATTGCAGTTATATATAT
AAAATGAGGTTTAAAAAGTAAAAATTTTAAAAAAATAAAGAGTACTTATTTAAAAAAAAAGGATTTTTTAA
AAAAAGTTTTATCATGGTACTACTGAAAAATTGTATTTAATTAAAATATTATAAAGCAATTGTTATTATTTT
CTGAAATAATAAAAATATTATTTATGGTTATTTTATGAACAGGAATAAATTATTTGTTATTTGTTATTCCATT
CTATAAGAGGAGAAATCAAATTTCTCTCCCGTCTTATGGCAAGTGTGTTCATTTGTAAATTATGTTCTTTCA
AAAGTAATATTTTATTTTATCAAATTATTGGCTAGAAAAAAATTAAAAAAAAAAAAAAAGATATATATATAT
ATATATATATATATATATATATATATATATATATATATATATA

Protein

RF: +1

ORF: 157 -> 1998

Length: 613 aa

>lcl|ORF1:157:1998

MALPRIKDEDQESKFGYVFGVSGPVVTAEKMSGSAMYELVRVGYFELVGEIIRLEGDMATIQVYEETSGVTVGD
PVLRTGKPLSVELGPGILGSIFDGIQRPLKDINEISNSIYIPKGVNIPALSRTAAWEFQPTNIKVGSHITGGDLYGVV
HENTLVKHKMILPPRAKGTVTYLAAPGNYTVDDVVLETEFDGEKTKFTMLQVWPVRQPRPVTEKLPANYPLLT
GQRVLDALFPCVQGGTTAIPGAFGCGKTVISQALSKYSNSDVIIYVGCGERGNEMSEVLRDFPELSVEIDGVTESI
MKRTALVANTSNMPVAAREASIYTGITLSEYFRDMGYNVSMMADSTSRWAEALREISGRLAEMPADSGYPAYL
GARLASFYERAGRVKCLGNPEREGSVSIVGAVSPPGGDFSDPVTSATLGIVQVFWGLDKKLAQRKHFPSINWLIS
YSKYMRALDDFYDKNFPEFVPLRTKVKEILQEEEDLSEIVQLVGKASLAESDKITLEIAKLLKDDFLQQNSYSPY
DRFCPFYKTVGMLKNMISFYDLARHAVESTAQSEKKITWAVIKESMGNILYQMSSMKFKDPVKDGESKIRSDFE
QLQEDIQQAFRNLED

Conserved Domains

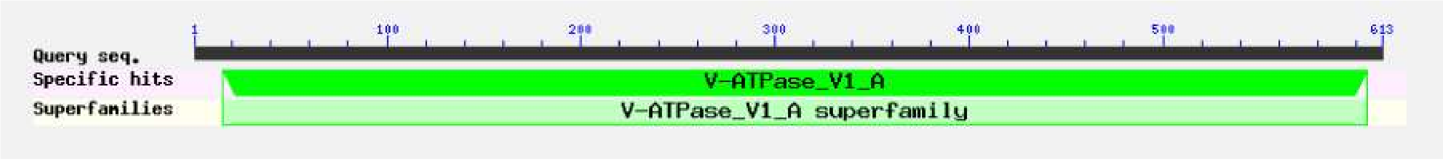

BLASTp

XP_014272529.1 V-type proton ATPase catalytic subunit A [*Halyomorpha halys*]

Score:1321 bits

E-value: 0.0

E-value 0.0 score 1256

Query 1 MALPRIKDEDQESKFGYVFGVSGPVVTAEKMSGSAMYELVRVGYFELVGEIIRLEGDMAT 60

MALPRIKDEDQESKFGYVFGVSGPVVTAEKMSGSAMYELVRVGYFELVGEIIRLEGDMAT

Sbjct 1 MALPRIKDEDQESKFGYVFGVSGPVVTAEKMSGSAMYELVRVGYFELVGEIIRLEGDMAT 60

Query 61 IQVYEETSGVTVGDPVLRTGKPLSVELGPGILGSIFDGIQRPLKDINEISNSIYIPKGVN 120

IQVYEETSGVTVGDPVLRTGKPLSVELGPGILGSIFDGIQRPLKDINEISNSIYIPKGVN

Sbjct 61 IQVYEETSGVTVGDPVLRTGKPLSVELGPGILGSIFDGIQRPLKDINEISNSIYIPKGVN 120

Query 121 IPALSRTAAWEFQPTNIKVGSHITGGDLYGVVHENTLVKHKMILPPRAKGTVTYLAAPGN 180

IPALSR+AAWEFQPTNIKVGSHITGGDLYGVVHENTLVKHKMILPPRAKGTVTYLAAPGN

Sbjct 121 IPALSRSAAWEFQPTNIKVGSHITGGDLYGVVHENTLVKHKMILPPRAKGTVTYLAAPGN 180

Query 181 YTVDDVVLETEFDGEKTKFTMLQVWPVRQPRPVTEKLPANYPLLTGQRVLDALFPCVQGG 240

YTVDDVVLETEFDGEKTKFTMLQVWPVRQPRPVTEKLPANYPLLTGQRVLDALFPCVQGG

Sbjct 181 YTVDDVVLETEFDGEKTKFTMLQVWPVRQPRPVTEKLPANYPLLTGQRVLDALFPCVQGG 240

Query 241 TTAIPGAFGCGKTVISQALSKYSNSDVIIYVGCGERGNEMSEVLRDFPELSVEIDGVTES 300

TTAIPGAFGCGKTVISQALSKYSNSDVIIYVGCGERGNEMSEVLRDFPELSVEIDGVTES

Sbjct 241 TTAIPGAFGCGKTVISQALSKYSNSDVIIYVGCGERGNEMSEVLRDFPELSVEIDGVTES 300

Query 301 IMKRTALVANTSNMPVAAREASIYTGITLSEYFRDMGYNVSMMADSTSRWAEALREISGR 360

IMKRTALVANTSNMPVAAREASIYTGITLSEYFRDMGYNVSMMADSTSRWAEALREISGR

Sbjct 301 IMKRTALVANTSNMPVAAREASIYTGITLSEYFRDMGYNVSMMADSTSRWAEALREISGR 360

Query 361 LAEMPADSGYPAYLGARLASFYERAGRVKCLGNPEREGSVSIVGAVSPPGGDFSDPVTSA 420

LAEMPADSGYPAYLGARLASFYERAGRVKCLGNPEREGSVSIVGAVSPPGGDFSDPVTSA

Sbjct 361 LAEMPADSGYPAYLGARLASFYERAGRVKCLGNPEREGSVSIVGAVSPPGGDFSDPVTSA 420

Query 421 TLGIVQVFWGLDKKLAQRKHFPSINWLISYSKYMRALDDFYDKNFPEFVPLRTKVKEILQ 480

TLGIVQVFWGLDKKLAQRKHFPSINWLISYSKYMRALDDFYDKNFPEFVPLRTKVKEILQ

Sbjct 421 TLGIVQVFWGLDKKLAQRKHFPSINWLISYSKYMRALDDFYDKNFPEFVPLRTKVKEILQ 480

Query 481 EEEDLSEIVQLVGKASLAESDKITLEIAKLLKDDFLQQNSYSPYDRFCPFYKTVGMLKNM 540

EEEDLSEIVQLVGKASLAESDKITLEIAKLLKDDFLQQNSYSPYDRFCPFYKTVGMLKNM

Sbjct 481 EEEDLSEIVQLVGKASLAESDKITLEIAKLLKDDFLQQNSYSPYDRFCPFYKTVGMLKNM 540

Query 541 ISFYDLARHAVESTAQSEKKITWAVIKESMGNILYQMSSMKFKDPVKDGESKIRSDFEQL 600

I+FYDLARHAVESTAQSEKKIT+AVIKESMGNILYQMSSMKFKDPVKDGESKIR+DFEQL

Sbjct 541 ITFYDLARHAVESTAQSEKKITFAVIKESMGNILYQMSSMKFKDPVKDGESKIRADFEQL 600

Query 601 QEDIQQAFRNLED 613

QEDIQQAFRNLED

Sbjct 601 QEDIQQAFRNLED 613

Vacuolar H+ ATPase sub unit C (vha16)

>TRINITY_DN1028_c0_g3_i1 len=2707 path=[0:0-2706]

CCAATGAGAAGCAACCTTACATAGAGTCCTTGGTCGAAAATCCTTGCAATCACATGATCGTTCAGCAAGTC
TAGGGGTGTAAAGACAGCTTTTGGTCCATTTCTATTTCTGTCATCTGAGGTACTTGCTCCTGCTCAGGAATT
AACCCACCTAAAATGTCAACATCCGAGAACCCAATTTACGGACCATTTTTTGGAGTTATGGGAGCCGCTTC
AGCTATGATATTTAGCGCTCTCGGTGCTGCCTATGGAACTGCAAAATCAGGTACGGGTATCGCAGCCATGT
CTGTTATGCGACCCGAGCTGATCATGAAATCTATTATTCCTGTTGTCATGGCTGGTATTATCGCTATTTATGG
CTTGGTAGTTGCTGTACTGATTGCAGGAGCTCTTGACCCTCCTGCTAAATATTCACTATACAAGGGTTTCAT
GCATCTTGGAGCTGGTTTAGCAGTTGGTTTCAGTGGTCTTGCTGCTGGCTTTGCCATTGGAATAGTTGGTGA
CGCAGGTGTAAGGGGAACTGCTCAACAACCACGATTGTTTGTCGGAATGATTCTAATTTTGATTTTTGCTGA
AGTATTAGGTCTCTACGGACTTATTGTGGCAATCTATTTGTACACAAAATAAATCATTTGTTTGGTATTCTTT

GTGGGCTCAACAGCTACCCTTTTTTGACCAGTATATCCTTGTAGACAATATAGTTAATCATTTTCCAATCAT
GATTTCTCTTTTATTTTCCACCCTCATAAGTATCCCTTTTTTTTTTTCTTTTTGTTTTAATTTTAAATTTCATTT
AATCATTCTGAGCTGTAGAATCAATTTGTGTTATAAAGTGATTGGGGAATGATTGCACCTTTTATTTTCTTTC
TTTTTTTTTTTTTTTTTATTTTTTTTTTTTTTTTTTTTTTTTCCTGTAAGAAGACAAATATTTAGTCGAGATTAA
ATGGAGGATATAATATTAAAATAAAGTGGGTAATAAAAAACAGAGATCTAGCGCGAAATTATTTAGTGAG
TAAACTTATATATTTTCTATTAGAGAGAGTTACAAGTGTATTAGTTAACTAAATGCGGGAGACAATTGGTTT
AATATTCATTGCCAACGAAAAAACACAGACAAAAGATTTAAAAATATATGTATATCGCATTAGTTTGATAC
CTAATTAGAAAATGTATGTTATTTCCTTGTATATCTTTAAATTGTAATTTTAGTTGTGTCTTTTTATATAATTT
GTGTCATTTGTAAATATTCGATTGTTTGGATTTTATTTTAATCCATTTATTATAAATATATATATGTATGTGT
GAAATCTCGAAAACTTATTTTGAAAGAGGGCATATTGAAAGCTACTAAATGTATTTATTTAAAAAAAAACAA
AAAAACTGTTACGTTACTGATTGTTACTACGAGATTGTACTATATAATTATGAAAATTAGTCATTAAGTATT
ATTTAATGATATAGGTTCTTATAGGGACATAACCTATATATATAGCATATATATATAAAAAACATATTGGTA
TATAAGTACGTATTAAGAACATGGTTAAATTGATTAATCTCTTATCACAAGAGCAAGACAATATTATGTTTA
AGTCTTCTAAGAATTTCACCTCATCAATATCATTATTTAAATTTCAATTCTTTTAATCGACATTTTAATAAAT
TTCAATGTTAATTTAGATATTATTTTATGGATATAGAATGTATTAGAGGAAAAAAATGTATAAGTATTAATAA
AAATGTGAACATATTGTTTTGGAGGGGAGACTTTTAAATGTTCACGTATTGCTATTCATCAAATTATACAAC
GCTATTTTATATATTTTATTGCAAAAGTTATTTTTGATTTGTGCACACATTTTCTTCCAAGAAATATATCAAA
AGCTCCTTATTATATAAAATAAAATACTTATTGACCAATAGATGTGAAGAATGAGTGCGTGATAGTGTAAG
CTACTCTATATCCCCCGTCGCCTCCTCATAATGTCTAAGGCATGTGATATAATTACACAGGTATTTATTTGA
GGCAGATCTTTAGTGTGTTTATGGCCCCTGTGTATGGAGTATATGTTGCGGTGCGGAAGGAAAGAGATTGA
TTGGCAAACATGTCAAGATTATCTACTCATCACAAATCTTACTAAGTAATAGTTATCTTGGAACAGAAAATA
TTCACAAAGATAGGAAAAAAAAAGGGAAAAAAAAGTAAAAAGAAAAAAAATTACTGCAACAAACAAACTA
GTTTTAGTTAGAATTTTAGGAGACTGATGAAGGAGACTGCCCCATGGAAACTATTTCTGTTCCTAAGTCCCG
TTGGCATGTGTGTCTTTCTCTTTGGCCTTCCCACCCCTTGTCTTTTTAGGTTATAGATACAGTCTGTGGTTAC
GTATATATATTATGTATGTAGAATGCTTTCTATTCATTTTTTTAATTTTATTTTAAATTTTGTATTAAAGCACA
TATCGTTATTTTGGAGAGCTTGTTATGTTTTTAAAGTTAATCTGTTTTATTTATTACAAATTGTTAATATTAT
TCATATCACATTCCACTAATTGTTTCCTTTAAAGATTTAATGTTTTGAAAATTATTCTTTTGATTATAAATTTT
AAAAATTGTTTATCACCAACTTTGTTGTGAATGTATGCTTGCTGTTCACTTATCTTGTTTAACAGTTATGTTT
GGACACTAGATTATTATATTTGTATTTATTTCG

Protein

<u>RF:</u> +3

<u>ORF:</u> 156 -> 626

<u>Length</u>: 156 aa

>lcl|ORF9_TRINITY_DN1028_c0_g3_i1:155:625 unnamed protein product

MSTSENPIYGPFFGVMGAASAMIFSALGAAYGTAKSGTGIAAMSVMRPELIMKSIIPVVMAGIIAIYGLVVAVLIA
GALDPPAKYSLYKGFMHLGAGLAVGFSGLAAGFAIGIVGDAGVRGTAQQPRLFVGMILILIFAEVLGLYGLIVAI
YLYTK

Conserved Domains

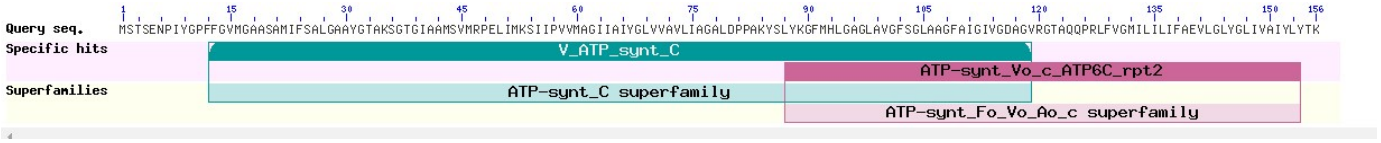

Query 1 MSTSENPIYGPFFGVMGAASAMIFSALGAAYGTAKSGTGIAAMSVMRPELIMKSIIPVVM 60

MS+++NPIYGPFFGVMGAASAMIFSALGAAYGTAKSGTGIAAMSVMRPELIMKSIIPVVM

Sbjct 1 MSSTDNPIYGPFFGVMGAASAMIFSALGAAYGTAKSGTGIAAMSVMRPELIMKSIIPVVM 60

Query 61 AGIIAIYGLVVAVLIAGALDPPAKYSLYKGFMHLGAGLAVGFSGLAAGFAIGIVGDAGVR 120

AGIIAIYGLVVAVLIAGALDPPAKYSLYKGFMHLGAGLAVGFSGLAAGFAIGIVGDAGVR

Sbjct 61 AGIIAIYGLVVAVLIAGALDPPAKYSLYKGFMHLGAGLAVGFSGLAAGFAIGIVGDAGVR 120

Query 121 GTAQQPRLFVGMILILIFAEVLGLYGLIVAIYLYTK 156

GTAQQPRLFVGMILILIFAEVLGLYGLIVAIYLYTK

Sbjct 121 GTAQQPRLFVGMILILIFAEVLGLYGLIVAIYLYTK 156

Small Rab GTPases

>TRINITY_DN3137_c0_g1_i1 len=3162 path=[1:0-222 3:223-407 4:408-3161]

ATAATACAAGTGAACAAGTACCATATATTTTATTTTAAATTCATTCTATACATTAACATAATATTGACTTCC
ATTATGCAATTCACTATAAATCATATTACACAGTAGCCATAAATTGTTTAAAATTTTGTTTTTGAATAATAA
TTGTGAACATTGCCCTATACTTATAATGTTTTAATTTGTATTAAATACAACAAACCACCATCTGAATCAATA
ACCAGTTCTTAAAGATGACATCGAGGAAGAAAGTTCTATTGAAAGTTATCATTCTTGGTGATTCTGGAGTTG
GTAAAACTTCACTCATGAACCAATATGTTAATAAAAAATTTTCAAACCAGTACAAGGCTACAATCGGTGCA
GATTTCCTCACAAAAGAAGTGTTAGTTGATGATAGAGTTGTTACGATGCAGATATGGGACACAGCAGGCCA
AGAAAGATTTCAATCATTAGGAGTTGCATTTTACCGAGGGGCTGATTGTTGTGTACTTGTATTTGATGTGTC
TGCGCCGACAACTTTTAAATCACTTGACTCTTGGCGTGATGAGTTTCTCATACAAGCTTCTCCTAGAGATCC
TGAGAACTTTCCTTTTGTTCTGCTGGGAAACAAAGTTGACCTGGAGAACAGAGCGGTTTCTGCCAAAAGAG
CGCAGCACTGGTGCCAGTCCAAGAACAATATTCCTTACTATGAAACAAGTGCAAAAGAAGCCATAAATGTT
GAGCAAGCATTTCTTACTATTGCTAAAAACGCCCTCGCTCAAGAATCGGAAGCAAACCTGTATAATGAGTT
CCCTGACCAAATAAAGCTAACTGGAGACCCCAAAGTTCAAATGCCAGATCAGTGCGCCTGTTAGTTGAAGA
ATGTTTTCTGTGATTTCATCGTTTGTGCTTTTGATGGAGCACATTGCATCTACTCAGCTCCGCATTACCGGTT
TTTAATTGGGGGGGTCTCAGGGTAACAAATCTAATGATACGTTAATATATTTATATATATATGTTTGCATAT
ATATAAACTGAAAAATATACATTATTTATATATAGACATATAAAATATAGAATTAATTATACATAAACCTAT
CATATGCTGGAATATTATGTATTTACTGTTCTTTTACAGTAAATATAATTTTTTATCAATGTAATATTAGATT
TATTATGTCTGTTGTCTTATTTTGTAAATACATGATTATGTGATAAAACATACAAATTATATGAAGTGCACC
TTCAAACTGTATAGTCATGACATATACTTAATTATTTGAGATTTGTAAATTAATTGTGTTTTTGTATTTAAAT
CAGTTCAGCCTCATTAGTCAGTATTGATGTTCGTTTTTAATGCACTTCTTATCAATCTATATAATTTGTAATT
AAAACTTTGTGCTGGCAGTACTTTCAAAAGTTTCATTTTCAGTTTGACATTATTTATTTAGTTTATTTCACTA
ATATATATAGTTATAATGTATAATATGTATGTATATTAGATGAACTATATTGCATTTTATGATGTTTTATTTG
AAGGTCTGCTTTCTCTAGGCGTATTATATTAAACTAATGTTTTCAGAGTTATTTTTTTGTAAATTAGTAATAA
TAGATACAGGCAGTCTTAAACTTTTTAAATTGTTGTTTTCCATATTTTATTAAAGAACTATAGCTTGGTGTAT
TTAAACTGTATTATACATCAAGTAGAGATACCATTGAATTTAGAACTAAACAACTATTTTATAACTGTTTTA
AAGTTTTAATGAATTTTTTTTTCTGGAGTTGTGATACAAATTATTTTGTCAATCAGATTAGTATAAAATTATG
CTGTTTGTATAGTTATGACATGTTTAATATAGTTTGTATGCAATTATAAAATGTATTAAATTCTATACATGTA
TAGATTCAAGAAGGTGGTTTTTGTAACGCATATGAAGAAAAGTGCTTTGTGTATAGCAGTTCAGTTCACTTT
GTCATCGAATAAATAGATTGAATATTACTATATTTATATTCTCCTGGTTTTTAATTTTGATTTAAACATCATA
ATATAATTATTAAATTAACTAATAACATTTTTCCTTATATATCTAAAAAAACATTGATCTTTACACTACAGT

AATTCATTATTATGCCAGAAATTATCAAAATGGAACATACCATAGATCTTAATCACTTATTATAAGCCAACA
TTTAGACTTACATTGTTGGTCTTCATCAGGGCTTTGAATATAGTTATACGCAATAAGTATGAACTCAATGTA
CACTTATAGATAAATGTAAATATGCTTGAAATAAATATGGTAAAAGTGAAATACACAACACAACATCTGAG
TTGTAAAAAAAGTTTTCCTTCCAATCCCTGCAAAGTATATTTTGTAAGCAAATATAACTGTCTTTATCAAAA
CTGTCTCATTCTTTTAAGATCACTTTTTTTTATATCATATTCATCTTATAAGGACAACAATCTCAGTTATATTT
GTTAAATTTATGAAACTACTGATTTTTATCTTGAAATTCATTGCAATATGAGTAGAGGATTTCATCTGATCA
TTAAACTGACCCAAAAGGTAATTTTGTGTATCATGTTGCTTTAGTGTTGACACAACTTGACCAATCATAAAA
ATCTTGGTTTAGTAAAGTGAGCCAGTTTAAATTAATTTTTAATTTAAAAATAATAGGTGATAAGAGTGGTAA
ATCTGATAAAATAAATACTGTTGTAATGTTTTATATACAGTAAGTCTCATTATACAATGATGTATATAATGA
TTAGGCAGATGTAATCCCTTATGATGCTACCACTGAGTTGATAGTGTTTGAAACAGATGAAATTAAAAATA
GATATTTTGTTTTAAAACGTAATATTAGTTTAAATTTGACTTAATAATTATGTTATTAACAAAATAGTGTAA
GAAAAATTGGCTGAAATAGTTTCTATATTCATTAGTAAACACAAATAATAATTTTTGTCTATCTTCATACTT
GTCTCTCATTTTGAGATATTAAGCATTTTTGATGATTTGCATTTATACTGTTTATACTTTGAACTTTATCATG
AAAATTTAAATATGAAGTCTGAATGTGATCTTATTCCGTGATTGTGTGTTACTTTGTAGGTGTTTTGTTACCA
TTAAAGCTTTGGATCTTCAAAGCCTGGTGACATGAACTAAATGATTCACCTAATTACAG

Protein

RF: +3

ORF: 231 -> 851

Length: 106 aa

>lcl|ORF6_TRINITY_DN3137_c0_g1_i1:230:850 unnamed protein product

MTSRKKVLLKVIILGDSGVGKTSLMNQYVNKKFSNQYKATIGADFLTKEVLVDDRVVTMQIWDTAGQERFQSL
GVAFYRGADCCVLVFDVSAPTTFKSLDSWRDEFLIQASPRDPENFPFVLLGNKVDLENRAVSAKRAQHWCQSK
NNIPYYETSAKEAINVEQAFLTIAKNALAQESEANLYNEFPDQIKLTGDPKVQMPDQCAC

Conserved Domains

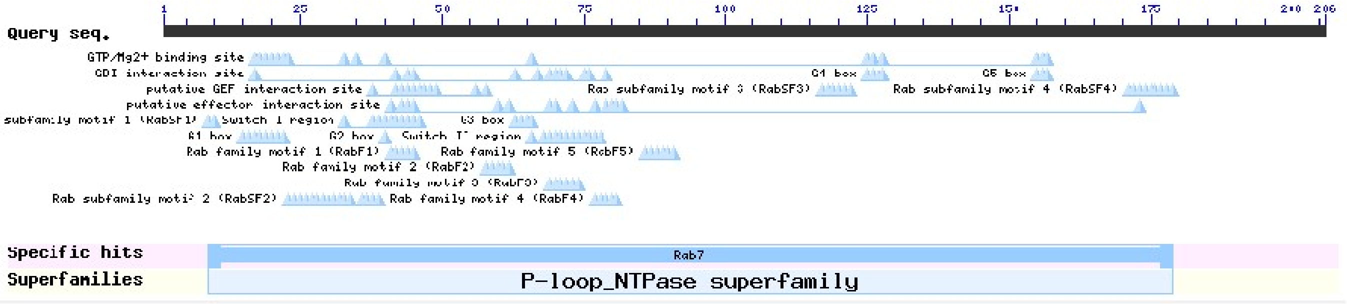

BLASTp

XP_014286452.1 ras-related protein rab7 [*Halyomorpha halys*]

Score:425 bits

E-value: 4.24e-154

```
 Query  1    MTSRKKVLLKVIILGDSGVGKTSLMNQYVNKKFSNQYKATIGADFLTKEVLVDDRVVTMQ  60
             MTSRKKVLLKVIILGDSGVGKTSLMNQYVNKKFSNQYKATIGADFLTKEVLVDDRVVTMQ
Sbjct  1    MTSRKKVLLKVIILGDSGVGKTSLMNQYVNKKFSNQYKATIGADFLTKEVLVDDRVVTMQ  60

Query  61   IWDTAGQERFQSLGVAFYRGADCCVLVFDVSAPTTFKSLDSWRDEFLIQASPRDPENFPF  120
             IWDTAGQERFQSLGVAFYRGADCCVLVFDVSAPTTFKSLDSWRDEFLIQASPRDPENFPF
Sbjct  61   IWDTAGQERFQSLGVAFYRGADCCVLVFDVSAPTTFKSLDSWRDEFLIQASPRDPENFPF  120

Query  121  VLLGNKVDLENRAVSAKRAQHWCQSKNNIPYYETSAKEAINVEQAFLTIAKNALAQESEA  180
             VLLGNKVDLENRAVSAKRAQHWCQSKNNIPYYETSAKEAINVEQAFLTIAKNALAQESEA
Sbjct  121  VLLGNKVDLENRAVSAKRAQHWCQSKNNIPYYETSAKEAINVEQAFLTIAKNALAQESEA  180

Query  181  NLYNEFPDQIKLTGDPKVQMPDQCAC  206
             NLYNEFPDQIKLTGDPKV  PDQCAC
Sbjct  181  NLYNEFPDQIKLTGDPKVPAPDQCAC  206
```

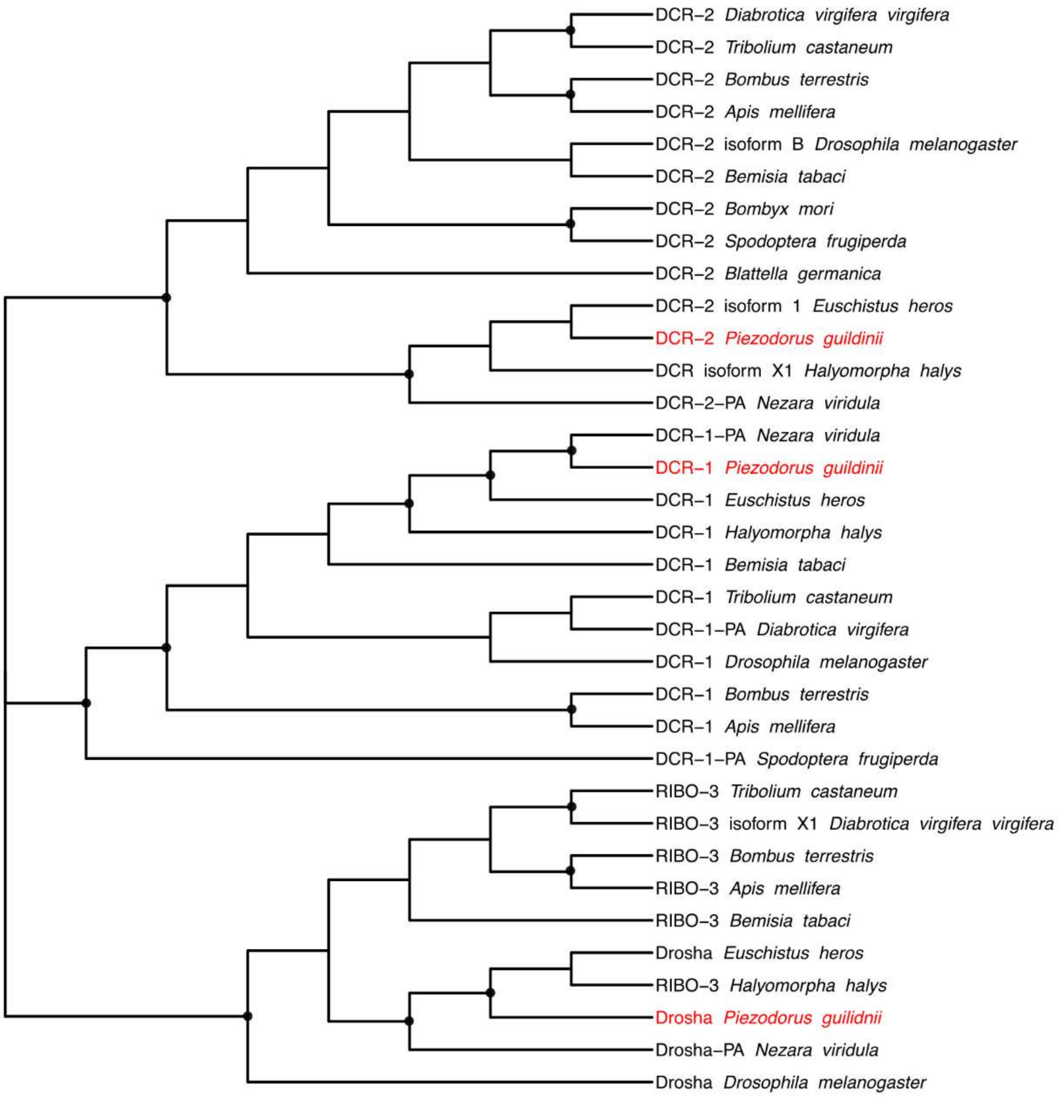

Figure S1 - Phylogenetic tree of *P. guildinii* Dicer 1 (DCR-1), Dicer 2 (DCR-2) and Drosha.
Proteins were aligned using MUSCLE and tested using the Neighbor-Joining Tree. Black dots at each node represent values > 70 calculated by bootstrap analysis (1,000 replicates).
Species and accession numbers used: **Hemiptera***: Euschistus heros (*sequences from Cagliari et al 2020)*, Halyomorpha halys (DCR-1 XP_014270680, RIBO-3 XP_014278529.1), Nezara viridula (DCR-1 AVK59457.1*, DCR-2 AVK59458.1, Drosha AVK59456.1), *Bemisia tabaci* (DCR-1 AHY18681.1, DCR-2 XP_018899828.1, RIBO-3 XP_018900133.1) **Blatodea**: *Blatella germanica (DCR-2 CCF23094.1),* **Hymenoptera** *Apis mellifera (DCR-1 NP_001116485.2, DCR-2 XP_016773223.2, RIBO-3 XP_016766928.1), Bombus terrestris (DCR-1 XP_020723074.1, DCR-2 XP_012163127.1, RIBO-3 XP_003394274.1)* **Diptera** *Drosophila melanoganster (DCR-1 AAF56056.1, DCR-2 NP_001286540., Drosha NP_477436.1)*, **Lepidoptera** *Spodoptera frugiperda (DCR-1 AVK59441.1,DCR-2 XP_035450081.1), Bombix mori (DCR-2 NP_001180543.1*) and **Coleoptera** *Tribolium costanacearum (DCR-1 XP_008199045.1, DCR-2 NP_001107840.1, RIBO-3 XP_008199088.1) Diabrotica virgifera (DCR-1 AVK59428.1, DCR-2 AUM60046.1, RIBO-3 XP_028140268.1)*.

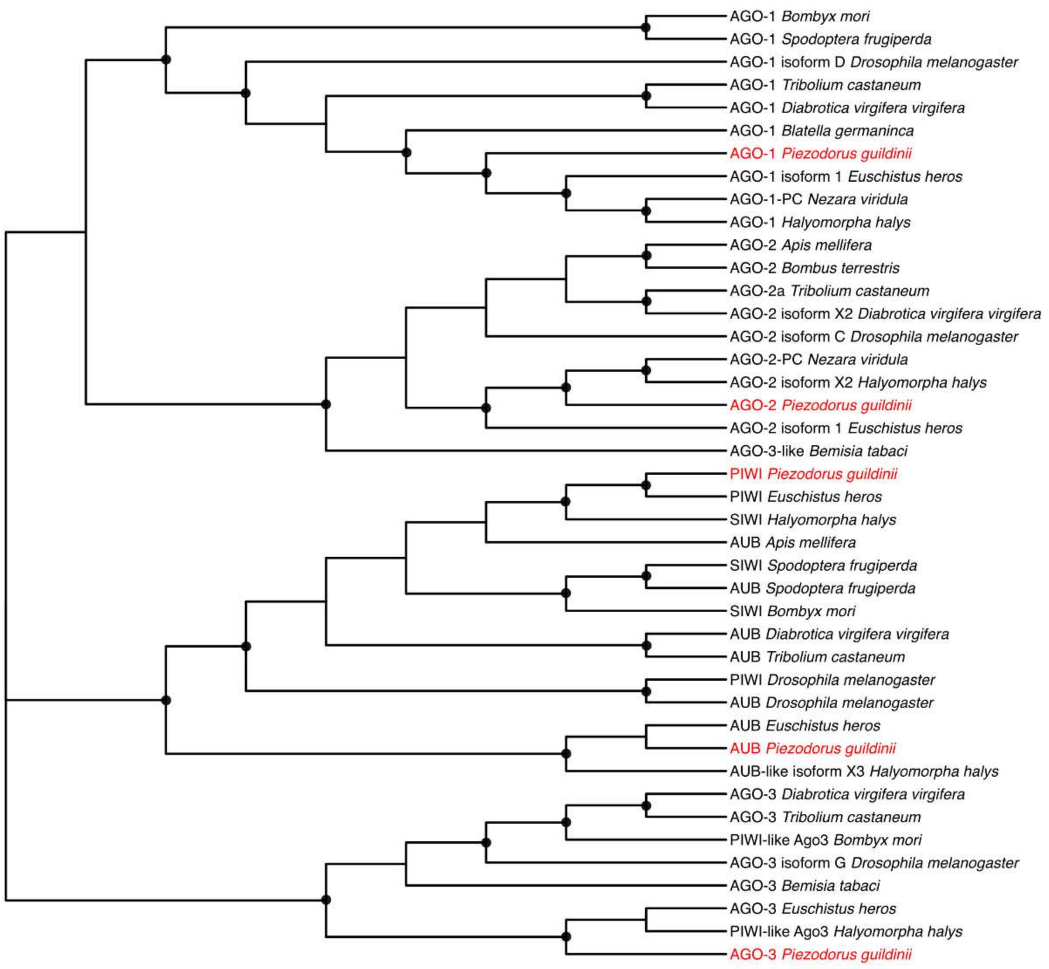

Figure S2 - *Phylogenetic tree of P. guildinii Argonaute 1 (AGO-1), Argonaute 2 (AGO-2), Argonaute 3 (AGO-3), Aubergine (AUB) and Piwi*. Proteins were aligned using MUSCLE and tested using the Neighbor-Joining Tree. Black dots at each node represent values > 70 calculated by bootstrap analysis (1,000 replicates). Species and accession numbers used: **Hemiptera:** *Euschistus hero (*sequences from Cagliari et al 2020)*, Halyomorpha halys (AGO-1 KAE8573837.1, AGO-2 XP_024214272.1 AGO-3, XP_014276831.1 AUB XP_014275927 SIWI XP_014270559.1) Nezara viridula* (AGO-1 AVK59466.1, AGO-2 AVK59468.1), *Bemisia tabaci* (AGO-2 XP_018905192.1, AGO-3 XP_018917778.1) **Blatodea:** *Blatella germanica (AGO-1 CCV01212.1),* **Hymenoptera**: *Apis mellifera: (AGO-2 XP_395048.4*, AUB NP_001159378.1), *Bombus terrestris* (AGO-2 XP_012168271.1) **Diptera**: *Drosophila melanoganster (AGO-1 NP_001246314.1, AGO-2 NP_730054.1, AGO-3 NP_001163498.1, AUB CAA64320.1, PIWI AGL81535)* **Lepidoptera:** *Spodoptera frugiperda (AGO-1 AVK59453.1, AUB AGS40930.1, PIWI XP_035434065.), Bombix mori* (AGO-1 NP_001095931.1, AGO-3 NP_001098067.2, PIWI NP_001098066.2) and **Coleoptera:** *Tribolium costanacearum (*AGO-1 EFA09197.2, AGO.2 NP_001107842.1, AGO-3 EFA02921.1, AUB XP_015837420.1*) Diabrotica virgifera* (AGO-1 AUM60041.1, AGO-2 XP_028131557.1, AGO-3 AUM60043.1, AUB AUM60044.1).

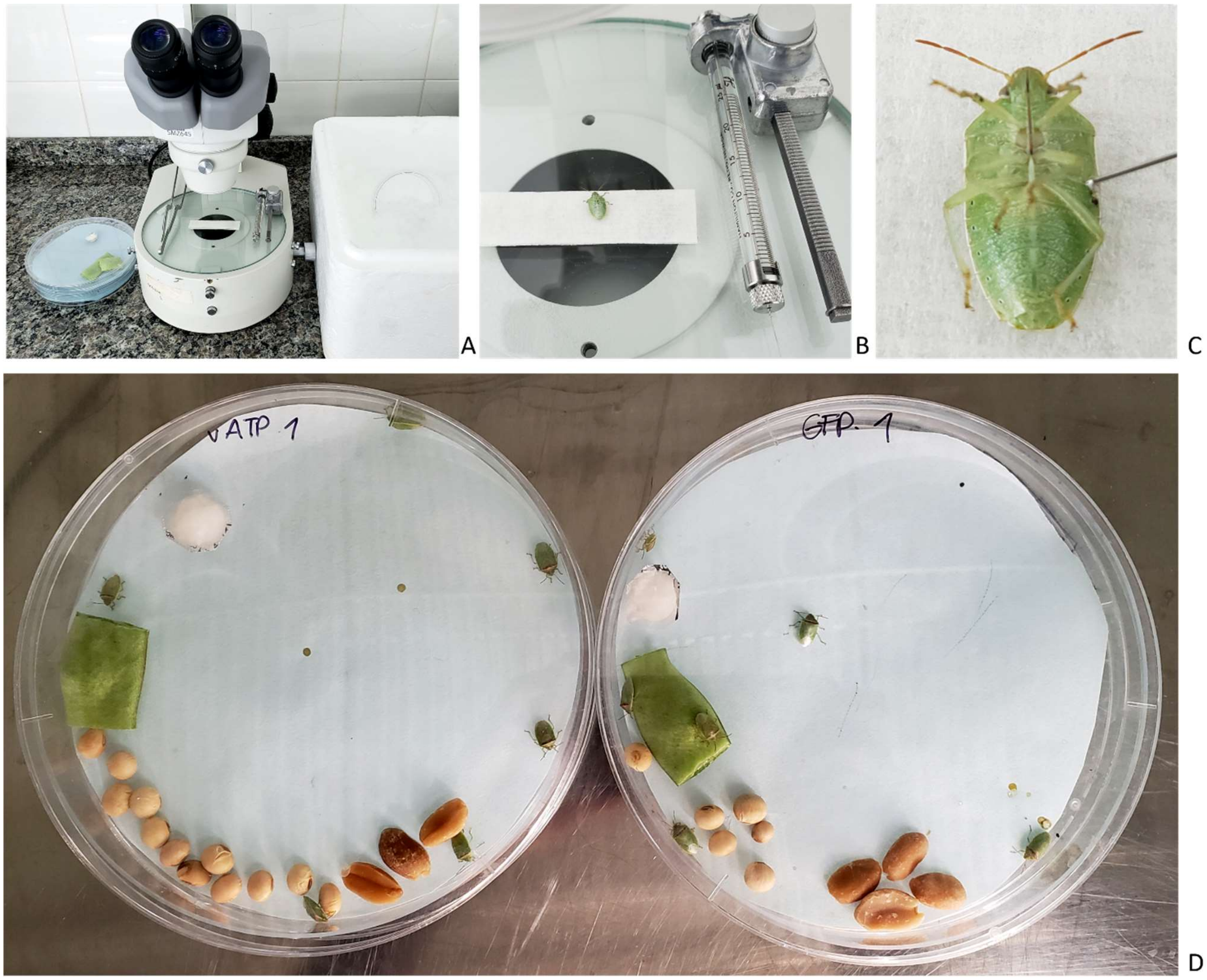

Figure S3. ***Piezodorus guildinii*** **dsRNA injection bioassay. A.** Microinjection setup consisting (from Right to left), anesthetizer ice, binocular microscope (2x) and petri dishes for insect recovery. B. Insects were immobilized ventral side up and injected with a Hamilton syringe with a 33G needle coupled to a PB600 repeater, C. Site of injection, ventral septum between the thoracic and abdominal segments indicated with 33G needle, D. Insects injected were maintained in petri dishes in rearing conditions and mortality was evaluated every day for 14 days.
